# Proteomic Analysis of the Acidocalcisome, an Organelle Conserved from Bacteria to Human Cells

**DOI:** 10.1371/journal.ppat.1004555

**Published:** 2014-12-11

**Authors:** Guozhong Huang, Paul N. Ulrich, Melissa Storey, Darryl Johnson, Julie Tischer, Javier A. Tovar, Silvia N. J. Moreno, Ron Orlando, Roberto Docampo

**Affiliations:** 1 Center for Tropical and Emerging Global Diseases and Department of Cellular Biology, University of Georgia, Athens, Georgia, United States of America; 2 Department of Biology, Georgia State University, Atlanta, Georgia, United States of America; 3 Complex Carbohydrate Research Center, University of Georgia, Athens, Georgia, United States of America; University of Dundee, United Kingdom

## Abstract

Acidocalcisomes are acidic organelles present in a diverse range of organisms from bacteria to human cells. In this study acidocalcisomes were purified from the model organism *Trypanosoma brucei*, and their protein composition was determined by mass spectrometry. The results, along with those that we previously reported, show that acidocalcisomes are rich in pumps and transporters, involved in phosphate and cation homeostasis, and calcium signaling. We validated the acidocalcisome localization of seven new, putative, acidocalcisome proteins (phosphate transporter, vacuolar H^+^-ATPase subunits *a* and *d*, vacuolar iron transporter, zinc transporter, polyamine transporter, and acid phosphatase), confirmed the presence of six previously characterized acidocalcisome proteins, and validated the localization of five novel proteins to different subcellular compartments by expressing them fused to epitope tags in their endogenous loci or by immunofluorescence microscopy with specific antibodies. Knockdown of several newly identified acidocalcisome proteins by RNA interference (RNAi) revealed that they are essential for the survival of the parasites. These results provide a comprehensive insight into the unique composition of acidocalcisomes of *T. brucei*, an important eukaryotic pathogen, and direct evidence that acidocalcisomes are especially adapted for the accumulation of polyphosphate.

## Author Summary

Acidocalcisomes are acidic organelles conserved from bacteria to human cells that are rich in polyphosphate, a polymer of orthophosphate units linked by high-energy phospho-anyhidride bonds. We found here that acidocalcisomes from *Trypanosoma brucei*, belonging to the group of organisms that produces African sleeping sickness and nagana, are rich in pumps, channels, and transporters involved in phosphate and cation homeostasis, and calcium signaling. Proteomic analysis of acidocalcisome fractions and expression of genes with epitope tags validated the presence of a number of novel transporters, and RNA interference demonstrated the essentiality of these organelles.

## Introduction

Acidocalcisomes were originally observed in bacteria and unicellular eukaryotes and named metachromatic [1] or volutin [2] granules. Later, when polymers of orthophosphate called polyphosphate (polyP) were identified at high levels within these organelles, acidocalcisomes were also called polyphosphate granules [3]. The length of polyP varies from as few as three to as many as thousands of residues [4]. The discovery of a diverse array of transporters established that acidocalcisomes are real organelles present from bacteria to human cells [5]. Acidocalcisomes have been well described in some species of bacteria [6], [7], trypanosomatids [8]–[10], apicomplexan parasites [11]–[13], fungi [14], [15], algae [16], [17], insect eggs [18], [19], sea urchin eggs [20], and chicken eggs [21]. Additionally, these organelles are also present in mammalian cells such as human platelets [22] and mast cells and basophils [23], where they belong to the group of organelles known as lysosome-related organelles (LROs). However, the name *acidocalcisome* was first used to describe these organelles in trypanosomatids [8], [9], and acidocalcisomes have been most extensively studied in these organisms.

*Trypanosoma brucei* belongs to a group of organisms responsible for human African trypanosomiasis (sleeping sickness), and nagana, a cattle disease in Africa. The two best-studied life stages of *T. brucei* are the procyclic forms (PCF), which grow in the intestine of the *tse tse* fly vector, and the bloodstream forms (BSF), which replicate in the blood of the mammalian host. Both stages can be grown in the laboratory and possess acidocalcisomes, although these are more abundant in the PCF [24]. Knowledge of the protein composition of acidocalcisomes will facilitate understanding of the physiological roles of these organelles. Among the proteins localized to acidocalcisomes of *T. brucei* so far is the vacuolar proton pyrophosphatase (TbVP1), which has been used as an acidocalcisome marker for subcellular fractionation studies [24]. In this work, we used iodixanol gradient centrifugation to obtain TbVP1-enriched fractions and examine the acidocalcisome proteome. We validated localization and essentiality of a selected group of proteins by *in situ* epitope tagging and immunofluorescence assays with specific antibodies, and RNA interference (RNAi) experiments, respectively. The results support the important role of these organelles in phosphate and cation homeostasis, and calcium signaling.

## Results

We isolated acidocalcisomes by a modification of isolation procedures described previously [17], [25]. After grinding with silicon carbide to break the cells, the lysates were fractionated by differential centrifugation followed by density-gradient ultracentrifugation using high-density solutions of iodixanol that were specially prepared by condensing the commercial iodixanol solution [17] (S1A Figure). Fractions were collected from the upper layers of the gradients. Composition of each fraction was confirmed using enzymatic and western blot analyses for organellar markers and microscopic observation.

**Figure 1:**
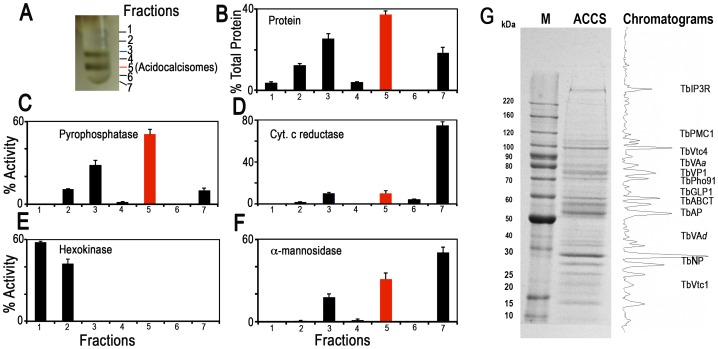
Distribution on iodixanol gradients of organellar markers from PCF trypanosomes. (A) Photograph showing bands obtained after the second iodixanol gradient centrifugation. Fraction 5 corresponds to the purified acidocalcisomes. (B) Protein distribution. (C) TbVP1 activity (measured as the AMDP-sensitive P_i_ release) is concentrated in fractions 3 and 5. (D) Mitochondrial marker distribution, succinate cytochrome c reductase. (E) Glycosomal marker distribution, hexokinase. (F) Lysosomal marker distribution, α-mannosidase. In (B–F) the *y-*axis indicates relative distribution; the *x-*axis indicates fraction number; *bars* show means ± SD (as a percentage of the total recovered activity) from two or three independent experiments. (G) SDS-PAGE of Fraction 5 from a representative acidocalcisome (ACCS) fractionation stained with Coomassie brilliant blue. The relative intensities of the bands were obtained from a bitmap file of the gel image and is shown on the right. Background was subtracted. The approximate localization of the acidocalcisome proteins identified in Table 2 is shown. BenchMark protein markers are shown at the left.

We analyzed the proteome using acidocalcisomes obtained via two different strategies. First, we utilized the pellet fraction from the first iodixanol gradient containing acidocalcisomes [25] (S1A Figure). Second, we used acidocalcisome samples obtained from fraction 5 of the second ultracentrifugation step of our iodixanol gradient protocol (Fig. 1A). Similar enzyme activity profiles were obtained in more than three independent fractionations. Since the vacuolar pyrophosphatase (TbVP1) activity (measured as aminomethylenediphosphonate (AMDP)-sensitive pyrophosphatase activity [24], [26] was highly enriched in fraction 5 of the second iodixanol gradient ultracentrifugation, we are reporting the proteomic results of this purified fraction from two of the experiments, although most acidocalcisome proteins described here were also detected in the acidocalcisome pellet obtained after the first iodixanol gradient centrifugation (results not shown).

Fig. 1 illustrates protein abundance (Fig. 1B) as well as distribution of markers for acidocalcisomes (TbVP1) (Fig. 1C), mitochondria (succinate cytochrome c reductase) (Fig. 1D), glycosomes (hexokinase) (Fig. 1E), and lysosomes (α-mannosidase) (Fig. 1F) as percentage of the total recovered activity from two to three independent experiments using double iodixanol gradient centrifugation of PCF lysates. Fractions 3 and 5 of PCF showed the highest TbVP1 activity (Fig. 1C) and were less contaminated with glycosome (Fig. 1E) or mitochondrial (Fig. 1D) markers than the other fractions. Very similar results to the enzymatic activities were obtained by western blot analyses of the different fractions (F1–F7) (S2A–B Figure) using antibodies against proteins localized to acidocalcisomes (TbVP1), mitochondria (voltage-dependent anion channel, TbVDAC), glycosomes (pyruvate, phosphate dikinase, TbPPDK), and lysosomes (Tbp67) (S2B Figure).

We also evaluated our purification method by comparing marker enzymes and activities in the 15,000×*g* fraction applied to the iodixanol gradient and the acidocalcisome fraction from the first and second iodixanol gradients (S1A Figure and Table 1). The pyrophosphatase yield was ∼10 and ∼5.0%, whereas the yield of protein was only 0.14 and 0.05%, a 70 and 99-fold purification, respectively. The only other organelles that were enriched to any extent in the acidocalcisome preparation after the first iodixanol gradient were glycosomes and lysosomes, as evidenced by a 3- and 2-fold purification of hexokinase and α-mannosidase, respectively. However, this purification was greatly reduced with the second gradient isolation. Mitochondria (marked by succinate cytochrome c reductase) were not enriched in these fractions. The acidocalcisomes obtained after two iodixanol gradients were therefore enriched by this technique >60-fold more than these other cell compartments.

**Table 1:** Purification of acidocalcisomes on iodixanol step gradients.

	Yield (%)		Purification-fold	
	1^st^	2^nd^	1^st^	2^nd^
Protein (mg)	0.14 (3)	0.05 (3)		
Pyrophosphatase*	9.81 (3)	4.96 (3)	70	99
Succinate cytochrome c reductase	0.12 (3)	0.02 (3)	1	0.2
Hexokinase	0.46 (3)	0 (3)	3	0
α-mannosidase	0.27 (3)	0.08 (3)	2	1.6

Yield values are percentages relative to the 15,000×*g* pellet fraction and represent averages from number of preparations in parentheses.

*Pyrophosphatase activities in the 15,000 g pellet, and the 1^st^ and 2^nd^ gradient acidocalcisome preparations were 0.22±0.09, 15.6±3.2, and 22.6±2.1 µmol min^−1^ mg^−1^ protein, respectively (mean ± SD).

Electron microscopy of PCF acidocalcisome fraction (fraction 5) (S1B Figure) showed round organelles of various sizes up to 200 nm in diameter, in some cases containing electron-dense material (*arrows* and *arrowheads*) and with the same appearance as acidocalcisomes isolated using Percoll gradients [24]. When fixed, acidocalcisomes lose their electron-dense content to a variable extent, resulting in a heterogeneous appearance. In contrast to the purity of fraction 5, electron microscopy of the 15,000×g pellet used to load the first gradient showed the presence of mitochondria, glycosomes, and flagella (S2C Figure), while that of the pellet of the first gradient showed some contamination with glycosomes (S2D Figure).

After SDS-PAGE of different pellets and gradient centrifugation bands, and enzymatic digestion with trypsin, peptides were analyzed by LC-MS/MS (see Materials and Methods). Fig. 1G shows a typical Coomassie brilliant blue-stained gel of proteins present in fraction 5 and the approximate positions in the gel that some of the putative acidocalcisome proteins studied in this work would have. A similar pattern of bands was obtained in three other fractionations (S3 Figure). S3 Figure also shows western blot analyses of these preparations with antibodies against known acidocalcisome proteins, such as TbVP1 [24] (S3A Figure, *arrowheads*), inositol-1,4,5-trisphosphate receptor (TbIP_3_R) [27] (S3B Figure, *arrowhead*), and vacuolar soluble pyrophosphatase (TbVSP) [28] (S3C Figure, *arrow*). Antibodies against TbVP1 reveal the presence of two bands as previously reported [29], antibodies against *T. cruzi* VSP show extra cross-reacting bands, and one of them (*arrowhead*) probably corresponds to the soluble inorganic pyrophosphatase (Tb927.3.2840; MW 28.7 kDa). Antibodies against TbIP_3_R show lower molecular mass bands that are probably hydrolysis products of this very high molecular weight protein.

### Protein identification

We identified a total of 580 proteins (1% false discovery rate, protein probabilities >0.95) from fraction 5 of the first (ACCS1) and second (ACCS2) experiments. The ACCS1 and ACCS2 datasets included 520 and 340 protein identifications, respectively (proteins are reported in S1 Table; peptides in S2 Table). When variants of similar proteins are indistinguishable from peptide data, the ProteinProphet [30] algorithm utilized by the ProteoIQ software treats these identifications as a single protein (a protein “group”). For example, two virtually identical isoforms (Tb927.4.4380 and Tb927.8.7980) of vacuolar-H^+^-pyrophosphatase (TbVP1) are present in *T. brucei* and vary in only 6 of 826 residues. Peptides from these proteins were unequivocally identified in our acidocalcisome datasets, and we report them as a single identification. In these instances, one or both of the proteins may be present. Two hundred nineteen are annotated as “hypothetical” in the *T. brucei* genome, and five were not represented in proteomic data available in TriTrypDB.org (downloaded May 28, 2014). Of the five with no prior mass spectrometry evidence, three were annotated as hypothetical. The remaining two proteins for which we provide novel expression evidence are annotated as frame-shift pseudogenes for a retrotransposon hot spot protein and a variant surface glycoprotein. Approximately 21% (120) of our 580 proteins have predicted transmembrane domains (S3 Table), consistent with estimates of representation in other organisms [31]. Of 40 identifications (6.9% of total), with predicted signal peptides, 22 also possessed putative transmembrane domains.

Annotated proteins in our proteomic dataset span a broad range of metabolic groups. Transport-related proteins accounted for ∼15%. Among these were transporters and pumps, vacuolar-H^+^-pyrophosphatase, an acidocalcisomal marker, was identified in our dataset. Other well-represented metabolic groups in our dataset were energy metabolism (∼14%), protein, lipid, carbohydrate, and nucleic acid metabolism (∼36%), and cell structure and organization (∼18%).

Subcellular localizations of each protein were predicted (S4 Table) using a series of algorithms (pTARGET, targetP, WoLF-PSORT, and SLP-LOCAL). Both plant and non-plant-optimized predictions were performed as a means of comparison, but we report here non-plant, targeting predictions. Approximately 20% of our identifications are nuclear, 17% are cytosolic, and ∼9% are mitochondrial. Plasma membrane and secretory predictions represent ∼5% and 1%, respectively. Table 2 shows proteins with known localization to acidocalcisomes of *T. brucei* and those established in this work (see below) and other proteins that we selected for localization studies. Table 2 indicates which of these markers were not present in our proteomic datasets (labeled with *asterisks*). Of the proteins identified by proteomic analysis of the subcellular fractions, we selected several proteins, some previously tested, for further validation (Table 2). Additionally, we selected other targets for validation based on properties that could justify acidocalcisome localization (Table 2).

**Table 2:** Identification of acidocalcisome protein candidates in *T. brucei*, showing localization and essentiality in BSF or PCF.

TriTrypDB Gene ID	Annotation (protein name)	MW (kDa)	TMD	Localization	Required for growth in BSF or PCF	Confidence (peptides)	Ref.
Tb927.4.4380 Tb927.8.7980	Vacuolar H^+^-PPase (TbVP1)	86	14	Ac	BSF, PCF	0.99 (8)	[29]
Tb927.8.1180	Vacuolar-Ca^2+^-ATPase (TbPMC1)	121	8	Ac	BSF, PCF	0.96 (8)	[36]
Tb927.8.2770	IP_3_ receptor (TbIP_3_R)	345	5	Ac	BSF, PCF	1 (22)	[27] this study
Tb927.7.3900	Vacuolar transporter chaperone 1 (TbVtc1)*	20	3	Ac	PCF	-	[40] this study
Tb927.11.12220	Vacuolar transporter chaperone 4 (TbVtc4)	91	3	Ac	BSF, PCF	1 (8)	[38], [39] this study
Tb927.11.7060 Tb927.11.7080	Vacuolar soluble PPase (TbVSP)*	47	0	Ac	BSF, PCF	-	[28]
Tb927.11.10650	Adaptor protein 3 subunit beta (TbAP-3β)*	100	0	Ac, Golgi, Endosomes	BSF, PCF	-	[63]
Tb927.5.3610	Adaptor protein 3 subunit delta (TbAP-3δ)*	125	1	Ac, Golgi, Endosomes	BSF, PCF	-	[63]
Tb927.5.1300	Vacuolar H^+^-ATPase subunit *a* (TbVA*a*)	89	6	Ac, Lysosome, Golgi	BSF, PCF	1 (5)	This study
Tb927.5.550	Vacuolar H^+^-ATPase subunit *d* (TbVA*d*)	42	0	Ac, Lysosome, Golgi	BSF, PCF	1 (1)	This study
Tb927.3.800	Vacuolar iron transporter (TbVIT1)*	30	3	Ac	BSF, PCF	-	This study
Tb927.4.4960	Zinc transporter (TbZnT)*	50	5	Ac	-	-	This study
Tb927.11.11160	Phosphate transporter (TbPho91)	81	10	Ac	-	1 (1)	This study
Tb927.10.7020	Acid phosphatase (TbAP)	49	0	Ac	-	1 (9)	This study
Tb927.9.10340	Polyamine transporter 1 (TbPOT1)*	54	11	Ac, Lysosome, Endosome	-	-	This study
Tb927.11.6680	Polyamine transporter 2 (TbPOT2)*	56	10	Lysosome	-	-	This study
Tb927.8.1870	Golgi/lysosome glycoprotein 1 (TbGLP1)	68	1	Lysosome, Golgi	-	1 (1)	This study
Tb927.11.540	ABC transporter (TbABCT)	76	6	Mitochondria	-	1 (1)	This study
Tb927.10.3640	Nuclear protein (TbNP)	31	6	Nucleus	-	1 (1)	This study
Tb927.11.840.1	Cation/proton antiporter (TbFTP)	81	15	Flagellar tip	-	-	This study

MW, molecular weight; TMD, transmembrane domains; Ac, acidocalcisome; IP_3_, inositol 1,4,5-trisphosphate; PPase, pyrophosphatase; -, not tested.

*Proteins for which peptides were not found in the acidocalcisome proteome.

### Proteins involved in Ca^2+^ signaling

**Figure 2:**
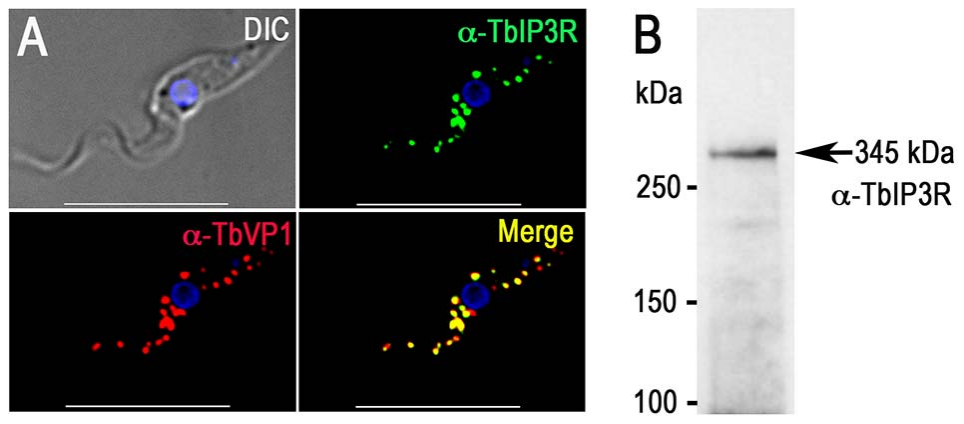
Immunofluorescence microscopy analysis of TbIP_3_R. (A) TbIP_3_R co-localized with TbVP1 in acidocalcisomes of PCF trypanosomes (Pearson's correlation coefficient of 0.8399). *Yellow* in merge images indicates co-localization. *Scale bars* = 10 µm. (B) Western blot analysis of TbIP_3_R expressed in PCF trypanosomes using polyclonal anti-TbIP_3_R antibody. Lysate containing 30 µg of protein from PCF trypanosomes was subjected to SDS/PAGE on 4–15% polyacrylamide gel, and transferred to a nitrocellulose membrane. Molecular weight markers at *left* and *arrow* shows the band corresponding to TbIP_3_R.

The acidocalcisomes in trypanosomatids serve as large acidic calcium stores [5], [32], and a number of proteins in these organelles can mediate Ca^2+^ signaling in the cell. The localization of the inositol 1,4,5-trisphosphate receptor (IP_3_R) in trypanosomatids has been controversial, but endogenous tagging of the IP_3_R of *T. brucei* with a 3× HA epitope tag demonstrated specific localization to the acidocalcisomes in this species [27]. The IP_3_R-HA did not co-localize with TbBiP, an ER marker [33] with a clear reticular labeling. Proteomic analysis of acidocalcisome fractions (unpublished) and contractile vacuole complex fractions [34] of *T. cruzi* also supported the presence of IP_3_R in these organelles. These results corroborate the punctate vacuolar localization in *T. cruzi* reported for TcIP_3_R by other authors [35]. These authors suggested an endoplasmic reticulum (ER) localization of TcIP_3_R, but no clear co-localization with TbBiP antibodies was presented [35]. To confirm the acidocalcisome localization of TbIP_3_R, we generated an antibody against the IP_3_ binding region of TbIP_3_R. Immunofluorescence analysis using this antibody confirmed the acidocalcisome localization, as determined by co-localization with antibodies against TbVP1 in *T. brucei* (Fig. 2A). Western blot analysis confirmed specificity of these antibodies (Fig. 2B and S3B Figure).

The acidocalcisome localization of the vacuolar Ca^2+^-ATPase (TbPMC1, Tb927.8.1180) [36] was also confirmed in our proteomic analysis (Table 2). Peptides from other Ca^2+^-ATPases (Tb927.3.3400, annotated as sarcoplasmic-endoplasmic reticulum-type Ca^2+^-ATPase; and Tb927.8.1160, annotated as vacuolar-type Ca^2+^-ATPase) were also detected (S3 Table), although they probably indicate similarity of peptides from different ATPases or contamination with other subcellular membrane fractions.

### Proteins involved in phosphate and polyP metabolism

**Figure 3:**
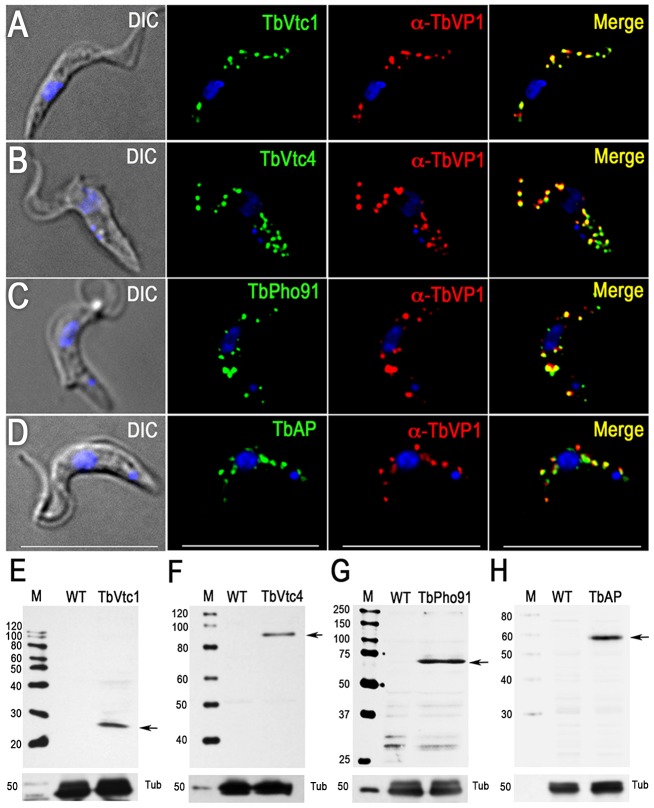
Immunofluorescence microscopy and western blot analysis of proteins involved in phosphorus metabolism. TbVtc1 (A), TbVtc4 (B), TbPho91 (C), and TbAP (D) were 3×HA epitope-tagged in situ and co-localized with TbVP1 in acidocalcisomes of PCF trypanosomes (Pearson's correlation coefficients of 0.873, 0.734, 0.728, and 0.680, respectively). *Yellow* in merge images indicate co-localization. *Scale bars* for (A–D) = 10 µm. Western blot analyses with monoclonal anti-HA showing labeling of TbVtc1 (E), TbVtc4 (F), TbPho91 (G), and TbAP (H) in PCF trypanosomes. Molecular weight markers at *left*, and *arrows* show the corresponding bands identified. Tubulin (*Tub*) was used as a loading control.

The vacuolar transporter chaperone complex (VTC complex) is involved in polyP synthesis in yeast [37] and trypanosomes [38], [39]. Homologues of the yeast proteins (Vtc1p to Vtc4p) are present in the genomes of trypanosomatids, apicomplexan, fungi, and algae but absent in mammalian cells. GFP-tagged *T. brucei* vacuolar transporter chaperone 1 (TbVtc1) localized to acidocalcisomes and the ER, although ER localization was attributed to an artifact of protein overexpression [40]. Although we did not detect peptides for this protein in the acidocalcisome proteome, we re-examined its localization and avoided pitfalls of overexpression and abnormal distribution by expressing 3× HA-tagged TbVtc1 in its endogenous locus under wild-type regulation. TbVtc1 perfectly co-localized with TbVP1 to acidocalcisomes (Fig. 3A). TbVtc4, which was positively identified in the acidocalcisome proteome (S1 Table), also co-localized to acidocalcisomes with TbVP1 (Fig. 3B), as reported previously [38]. Western blot analyzes confirmed the expression of the tagged proteins (Fig. 3E and 3F).

A putative phosphate transporter (TcPho1, TcCLB.508831.60) in *T. cruzi*, which was originally annotated as a sodium/sulphate symporter, localizes to the contractile vacuole and intracellular membranes of epimastigotes of *T. cruzi* [34]. The product of the *T. brucei* homologue (TbPho91, Tb927.11.11160) co-localized with TbVP1 in acidocalcisomes (Fig. 3C). Expression of the tagged protein was confirmed by western blot analysis (Fig. 3G).

Previous work [28] has indicated the presence of a vacuolar soluble pyrophosphatase in acidocalcisomes of *T. brucei* (TbVSP, Tb927.11.7060 and Tb927.11.7080). Although peptides corresponding to this protein were not identified in the proteome, antibodies against this protein reacted with a band of ∼50 kDa corresponding to the apparent molecular mass of the protein in the acidocalcisome fraction (S3C Figure, *arrow*).

We also investigated the localization of a putative acid phosphatase (Tb927.10.7020; TbAP), which was present in our acidocalcisome fractions (S1 Table). The presence of an acid phosphatase activity in *T. rangeli* acidocalcisomes was detected by cytochemical methods [41], and early work in *T. brucei rhodesiense* also localized an acid phosphatase activity to lysosome-like vesicles that probably correspond to acidocalcisomes [42]. We found that TbAP co-localized with TbVP1 to acidocalcisomes (Fig. 3D). Western blot analysis confirmed the expression of the tagged protein (Fig. 3H).

### Proton pumps

**Figure 4:**
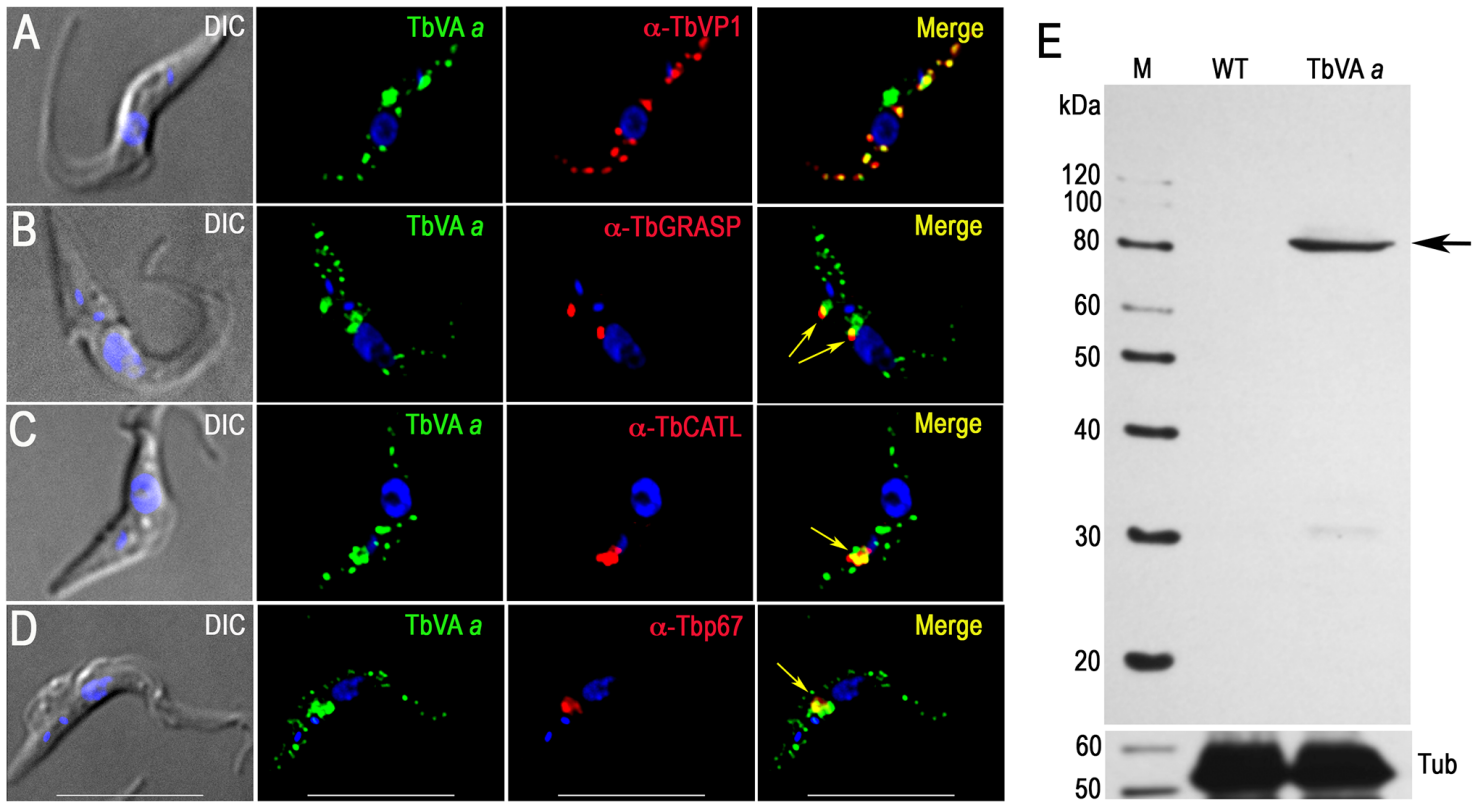
Immunofluorescence microscopy and western blot analysis of V-H^+^-ATPase subunit *a* in PCF trypanosomes. Epitope-tagged V-H^+^-ATPase subunit *a* co-localizes with TbVP1 to the acidocalcisomes (A), with TbGRASP to the Golgi complex (B) with TbCATL (C) and with p67 (D) and to lysosomes (Pearson's correlation coefficients of 0.631, 0.539, 0.804, and 0.754, respectively). *Yellow* in merge images indicate co-localization (also shown with *arrows* in (B–D)). *Scale bars* for (A–D) = 10 µm. (E) Confirmation of tagging by western blot analyses with monoclonal anti-HA in PCF trypanosomes. HRP-conjugated goat anti-mouse was used as a secondary antibody. Magic Mark XP (Invitrogen) was used as a molecular weight marker and *arrow* shows band corresponding to TbVA*a*. Tubulin (Tub) was used as a loading control (*bottom panel*).

Proton pumps maintain a low pH inside acidocalcisomes. We identified both TbVP1 and vacuolar proton ATPase (V-H^+^-ATPase) in our proteomic analysis (Table 2). Early physiological studies using bafilomycin A_1_, a specific inhibitor of V-H^+^-ATPase [43], demonstrated V-H^+^-ATPase activity in permeabilized *T. brucei* PCF trypanosomes [8]. This finding was later confirmed in experiments with intact cells [44] and isolated acidocalcisomes [24]. All putative subunits of this pump are present in the *T. brucei* genome (TriTrypDB.org, S5 Table), and two of the subunits, the putative H^+^-translocating subunit *a* (TbVA*a*) and the putative H^+^ transporting subunit *d* (TbVA*d*), were found in our acidocalcisome proteomic analysis (Table 2). We tagged subunits *a*, and *d* with a 3× HA tag and found excellent co-localization with TbVP1 (Fig. 4A, and S4A Figure). Additional punctate staining of the *a* and *d* subunits that did not co-localize with TbVP1 could correspond to labeling of the Golgi complex and endocytic pathway, where the V-H^+^-ATPase also localizes in most eukaryotic cells. In agreement with that additional localization, we found that part of the antibody reaction against these subunits co-localizes with the Golgi marker Golgi reassembly and stacking protein (TbGRASP) [45] (Fig. 4B and S4B Figure) and with the lysosomal markers cathepsin L (TbCATL), a luminal lysosomal cysteine peptidase, and p67, a lysosomal membrane glycoprotein [46] (Figs. 4C and 4D, and S4C Figure and S4D Figure, respectively). Western blot analyses confirmed the expression of these proteins (Fig. 4E and S4E Figure).

### Other transporters

**Figure 5:**
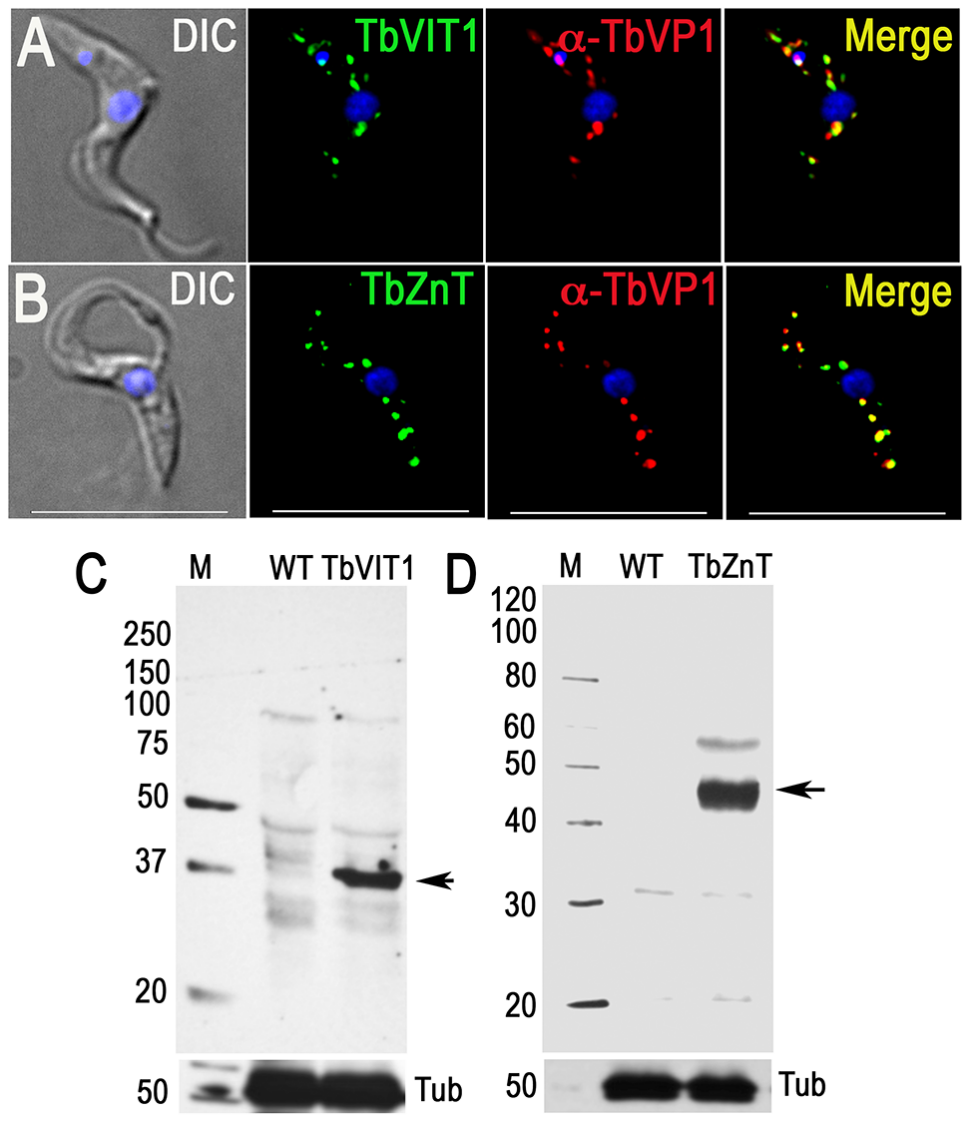
Immunofluorescence microscopy and western blot analyses of metal ion transporters. Epitope-tagged TbVIT1 (A) and TbZnT (B) and co-localize with TbVP1 to the acidocalcisomes (Pearson's correlation coefficients of 0.6879 and 0.7604, respectively). *Yellow* in merge images indicate co-localization. *Scale bars* for A–B = 10 µm. Tagging with HA was confirmed by western blot analyses of TbVIT1 (C) and TbZnT (D) using anti-HA antibodies. Markers are at the *left* side, and *arrows* show the corresponding bands. Tubulin (*Tub*) was used as a loading control (*bottom panel*).

Several acidocalcisome proteins of other trypanosomatids or with potential localization to acidocalcisomes were also investigated. Since iron has been detected in acidocalcisomes of *T. cruzi* [47], *Phytomonas spp.* [48], [49], and *Leishmania amazonensis* [50], we tagged a hypothetical protein (Tb927.3.800) with similarity to vacuolar iron transporters (VIT). This protein co-localized with TbVP1 (Fig. 5A), and western blot analysis of PCF trypanosome lysates showed a single band using anti-HA antibodies (Fig. 5C). We also tagged a putative metal-ion (zinc) transporter (Tb927.4.4960) as an homologue in *T. cruzi* (TcCLB.511439.50) occurs in acidocalcisomes [51]. Fig. 5B shows that HA-tagged Tb927.4.4960 co-localized with TbVP1, and western blot analyses (Fig. 5D) confirmed its expression.

### Proteins in the proteome that do not localize to acidocalcisomes

Several proteins enriched in the acidocalcisome proteome possess transmembrane domains (TMD), and some have homologues present in acidocalcisomes of other species. For example, Tb927.10.3640 has six predicted TMD and is annotated in TriTrypDB.org as a hypothetical protein. The C terminus was tagged with a 3× HA tag using homologous recombination with the endogenous locus. Surprisingly, the protein showed nuclear membrane localization (S5A Figure), and western blot analysis identified a single band of ∼35 kDa (predicted molecular mass, 32 kDa, S5C Figure). Interestingly, this protein was previously identified in a nuclear proteome of *T. brucei* [52]

An ABC transporter was identified in the acidocalcisomes of *Cyanidoschyzon merolae* [17] and Tb927.11.540, listed as a putative ABC transporter with six predicted TMD, was enriched in the *T. brucei* acidocalcisome proteome (S1 Table). However, antibodies against HA co-localized with MitoTracker in the mitochondrion of PCF (S5B Figure), and western blot analysis showed a strong band of ∼75 kDa compatible with the predicted molecular mass of 76 kDa. A second band at ∼60 kDa, may be due to cleavage of a mitochondrial targeting signal of 97 amino acids (S5D Figure).

Tb927.8.1870 is a Golgi/lysosome glycoprotein 1 (TbGLP1) reported to localize in the Golgi complex, multivesicular lysosomes, and in unidentified small vesicles [53]. As we detected localization of other acidocalcisome proteins in Golgi and lysosomes (Fig. 4 and S4 Figure) we tagged the C terminus of TbGLP1 with 3× HA. The small vesicles previously described [53] are apparently not the acidocalcisomes as TbGLP1 does not co-localize with TbVP1 (S6A Figure). Consistent with this, antibodies against HA co-localized with TbGRASP (S6B Figure), TbCATL (S6C Figure) and p67 (S6D Figure). Western blot analysis showed a band of ∼90 kDa, close to the apparent molecular mass of the native protein [53] (S6E Figure).

### Proteins of potential acidocalcisome localization

Acidocalcisomes are rich in basic amino acids, and potentially polyamines to balance anionic charges of polyphosphate, as occurs in the yeast vacuole [54]. We investigated the localization of HA-tagged putative polyamine transporters TbPOT1 (Tb927.9.10340) and TbPOT2 (Tb927.11.6680). TbPOT1 partially co-localizes with acidocalcisomes (S7A Figure), and with lysosomes (S7B–C Figure). TbPOT2, in contrast, did not co-localize with Golgi complex (S8A Figure) and showed an exclusive lysosomal localization (S8B–C Figure). Western blot analyses confirmed the expression of the tagged proteins (S7E Figure and S8E Figure, respectively).

Biochemical evidence for the presence of a Na^+^/H^+^ exchanger in acidocalcisomes of different trypanosomatids [55] including *T. brucei* PCF [56], [57] has been presented. We therefore investigated the localization of Tb927.11.840.1, which has 15 predicted TMD and is annotated as a putative cation/proton antiporter in TriTrypDB.org, and as a potential Na^+^/H^+^ exchanger in TransportDB. Interestingly, HA-tagged TbFTP localizes to the distal tip of the flagellum of PCF, and does not co-localize with acidocalcisomes (S7D Figure). Western blot analysis identified one band absent in wild type cells (S7F Figure). Few proteins, among them adenylyl cyclases [58], a calpain-like protein TbCALP.1.3 [59], the kinesin motor Kif13-2 [60], an unknown antigen, and the flagellar protein FLAM8 [61], have previously been reported to exhibit localization to the flagellar tip of *T. brucei*. In addition, a cation channel does occur in the distal tip of the flagellum *T. cruzi* [62] and the presence of channels and exchangers at this localization may be compatible with the proposed role of the flagellum as an environmental sensor.

### Requirement of newly discovered acidocalcisome proteins for normal growth

**Figure 6:**
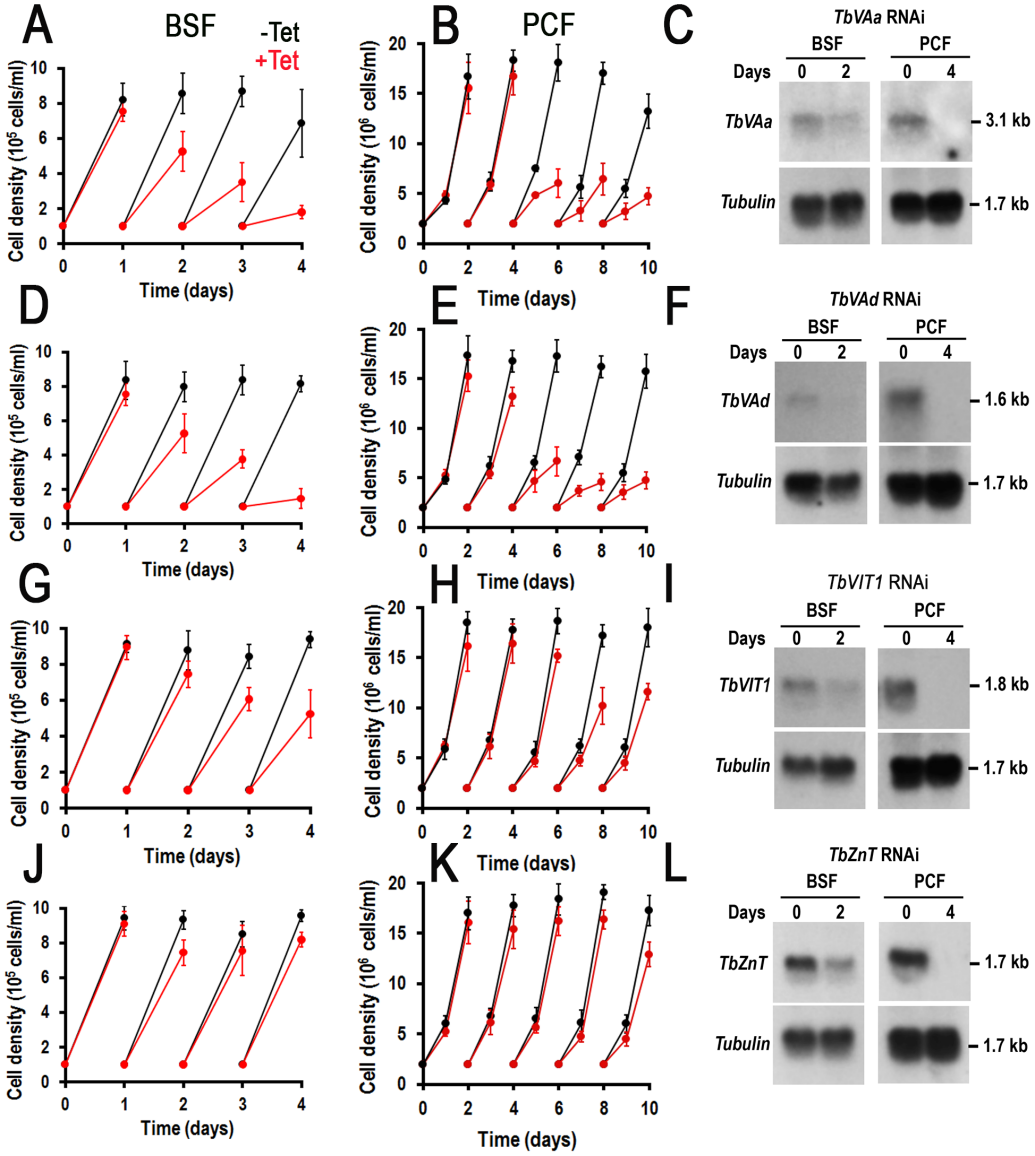
Effect of inhibition of expression of four acidocalcisome genes by tetracycline-induced RNAi on cell growth. (A–B), (D–E), (G–I), and (J–L) show growth of BSF (*left panels*) and PCF (*right panels*) trypanosomes of *TbVAa*, *TbVAd*, *TbVIT1*, and *TbZnT* RNAi in the absence (−*Tet*, *black lines*) or presence (+*Tet*, *red lines*) of 1 µg/ml tetracycline for the indicated number of days, respectively. Values are means ± SD (n = 3–4). (C), (F), (I) and (L) show northern blot analyses of *TbVAa*, *TbVAd*, *TbVIT1*, and *TbZnT* RNAi in the absence (0) or presence (2 or 4 days) of tetracycline, respectively. Tubulin is shown as a loading control. Markers are shown on the *right*.

We have reported before that a number of genes encoding acidocalcisome proteins such as TbVP1 [29], TbPMC1 [36], TbIP_3_R [27], TbVtc1 [40], TbVtc4 [38], [39], TbVSP [28], and AP-3 β and δ subunits [63] are essential for the growth of BSF and/or PCF trypanosomes (Table 2). We therefore selected some of the newly identified acidocalcisome proteins to investigate their requirement for growth. Knockdown of *TbVAa* or *TbVAd* by induction of double-stranded RNA resulted in growth defects in both BSF and PCF trypanosomes (Fig. 6A, 6B, and 6D, and 6E, respectively), with an 81±4% and 69±3% reduction in the number of cells, respectively. Northern blots (analysis performed with ImageJ software) showed that mRNA was down-regulated by 73–96% after 2 and 4 d of RNAi in BSF and PCF trypanosomes, respectively (Fig. 6C and 6F).

Knockdown of *TbVIT1* in both BSF and PCF trypanosomes (Fig. 6G and 6H) resulted in growth defects with a 44±6% and 41±3% reduction in the number of cells after 2 and 4 d of tetracycline addition to BSF and PCF trypanosomes, respectively. Knockdown of *TbZnT* only weakly affected the growth of PCF trypanosomes (Fig. 6J and 6K). Northern blot analyses showed that the mRNA was downregulated in all cases (Fig. 6I and 6L).

## Discussion

We report here the proteomic analysis of subcellular fractions enriched in acidocalcisomes from *T. brucei*. These fractions are enriched in proteins previously demonstrated to localize to acidocalcisomes like TbVP1 [24], TbPMC1 [36], TbVtc4 [38], and TbIP_3_R [27]. Our protocol yields fractions well resolved from organelle markers for mitochondria (succinate cytochrome c reductase, TbVDAC), glycosomes (hexokinase, TbPPDK) and lysosomes (α-mannosidase, Tbp67). We made 580 identifications in fractions highly enriched in TbVP1 activity. Membrane proteins are challenging for proteomic analysis, but our dataset includes a relatively high representation of membrane proteins (21% in fraction 5). A published plasma membrane proteome of *T. brucei* contains a lower proportion of membrane proteins (16.1% of 1,536 proteins, [64], suggesting that our fractionation successfully enriched proteins with potential, membrane-related functions. Additionally, our proteomic analysis confirmed expression of five proteins previously undetected in whole cell analyses of *T. brucei* (data from TriTrypDB.org, accessed May 28, 2014). This confirms the relevance of subcellular proteomics as a method of choice for the identification of larger numbers of proteins than whole cell proteomics [51].

Subcellular fractionation only partially purifies cellular components from contaminants. This contamination is due in part to the abundance of some proteins, the adhesive properties of others, and also because there are junctions that connect organelles with each other [65]. In this regard we previously discussed [66] the close association of acidocalcisomes with mitochondria of trypanosomes [67], an association that is important for Ca^2+^ signaling, and could explain the contamination of our fractions with mitochondrial membrane proteins. It is therefore essential that mass spectrometric analysis be validated with *in vivo* expression of tagged proteins. Only few studies to date [34], [51], [61], [68] have implemented such a method to verify proteomes of trypanosomatid parasites. To validate our dataset, we expressed a number of proteins in the acidocalcisome proteome as HA-fusion proteins. We complemented this set of proteins with selected proteins with known localizations to the acidocalcisomes in other species, and with proteins that could potentially be present in the acidocalcisomes on the basis of our knowledge of the organelle. Interestingly, several proteins previously localized to acidocalcisomes were absent in our dataset. These notable absences from our dataset suggest very low expression levels.

**Figure 7:**
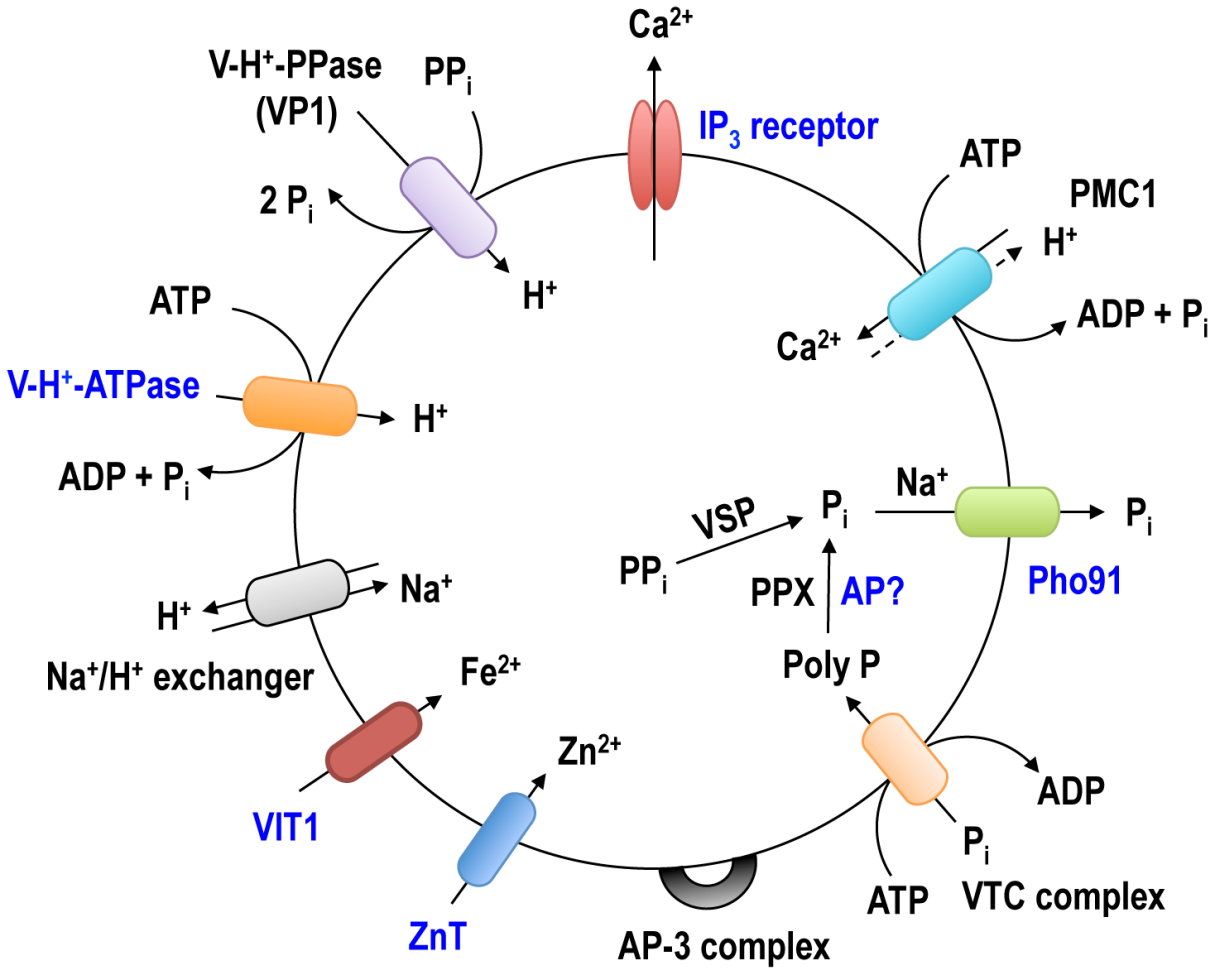
Schematic representation of the acidocalcisome of *T. brucei*. The identified acidocalcisome proteins corresponding to the pumps, exchangers, transporters or protein complexes in Table 2 are shown in this model. The newly identified acidocalcisome proteins in this study are marked in blue.

The proteins we localized to the acidocalcisomes (Fig. 7) belong to three groups: proteins involved in Ca^2+^ signaling, phosphate homeostasis, and membrane transport. The acidocalcisome localization of the IP_3_R [27] was confirmed using antibodies against the IP_3_ binding region of the receptor, which recognized a band of 345 kDa that corresponds to the apparent molecular mass of the receptor (343 kDa). The antibody marked an additional band at ∼80 kDa that likely corresponds to a hydrolysis product, as this band is very weak in immunoblots of total cell lysates. Although TbIP_3_R in *T. cruzi* was suggested to localize to the ER [35], the IFA results from *T. cruzi* were not convincing given that endogenously tagged *T. brucei* IP_3_R localizes to acidocalcisomes [27]. Further work is necessary to confirm this localization in other trypanosomatids. The identification of a mechanism for Ca^2+^ uptake (TbPMC1) and Ca^2+^ release (TbIP_3_R) in acidocalcisomes underscore the relevance of these organelles in Ca^2+^ signaling.

The acidocalcisome localization of two components of the VTC complex involved in synthesis of polyP [38], [40] was confirmed, and the excellent co-localization of TbVtc1 and TbVtc4 with TbVP1 in acidocalcisomes suggest that previously reported localization of TbVtc1 in the ER [40] was the consequence of its overexpression from an exogenous locus.

A phosphate transporter (TbPho91) annotated as sulfate/sodium symporter, and encoding for a putative *Saccharomyces cerevisiae* Pho91p orthologue (S9A Figure) was localized to the acidocalcisomes. Pho91p, is localized to the vacuole and proposed to be involved in exporting P_i_ from the vacuole to the cytosol [69]. The orthologue identified in *T. cruzi* (TcCLB.508831.60) shares 65% amino acid identity to TbPho91, and has been localized to the contractile vacuole and other membranes of that parasite [34]. The ORF of TbPho91 encodes a predicted, 728 amino acid protein with an apparent molecular weight of 81 kDa, nine transmembrane domains, an *N*-terminal regulatory SPX domain and an anion-permease domain that is also present in other anion transporters. The recognized polypeptide had an apparent molecular mass of ∼70 kDa and, since *T. brucei* Pho91 possesses ten transmembrane domains, a size discrepancy between the expected (99 kDa) and the observed molecular mass could be attributed to the usual anomalous migration of hydrophobic proteins on SDS gels [70]. If TbPho91 functions as its orthologue in *S. cerevisiae* [69], it could be involved in the release of P_i_ from the acidocalcisomes.

The acid phosphatase (TbAP) is the first soluble enzyme identified at the molecular level in acidocalcisomes of trypanosomatids. The gene (Tb927.10.7020) encodes a 50 kDa protein that has a signal peptide and belongs to the histidine phosphatase superfamily (TriTrypDB.org). Catalytic activity in the superfamily centers on phosphorylation and dephosphorylation of a histidine residue that follows the first β-strand of the protein. A conserved Arg-His-Gly (RHG) triad has been proposed to contain the phosphorylated histidine [71] and is conserved in TbAP. The LTXXG motif in the region between β1 and β2 is also conserved [71]. It is interesting to note that some acid phosphatases, like the tartrate-resistant or purple acid phosphatase (S9B Figure) have exopolyphosphatase activity [72] and further work will be needed to investigate whether the exopolyphosphatase activity detected in acidocalcisomes [73] is due to this enzyme.

The presence of a V-H^+^-ATPase activity was one of the defining properties that led to the identification of acidocalcisomes in trypanosomes [8], [9]. The enzyme activity was later localized to acidocalcisomes of different unicellular eukaryotes [5], but this is the first work studying the localization of the enzyme using epitope-tagged subunits. V-H^+^-ATPases are multisubunit proton pumps composed of two subcomplexes. The peripheral V_1_ complex consists of eight subunits (A to H) and is responsible for ATP hydrolysis, whereas the membrane-integral V_0_ complex (a, c, c′, c″, d, and e subunits) is responsible for proton translocation from the cytosol into the lumen of endomembrane compartments [74]. Epitope tagging of two membrane integral V_0_ complex subunits (*a* and *d*) identified the localization of this multisubunit complex to acidocalcisomes, lysosomes, and Golgi complex. This is in contrast with *T. cruzi* in which a P-type H^+^-ATPase is involved in acidification of the endocytic pathway [75]. As occurs with most organisms studied to date, the enzyme is essential for parasite growth and survival. It is also quite interesting that there is some heterogeneity in TbVP1 stain compared to some of these markers, which may well suggest that there is more than one class of compartment or at least differential compositions. This could indicate either functional differences or maturation/degradation of these compartments.

Two new metal ion transporters were identified. Tb927.3.800 is an orthologue to the vacuolar iron transporter (VIT1) originally described in *Arabidopsis thaliana* [76] and to the yeast Ca^2+^-sensitive cross-complementer 1 (CCC1) [77] (S9C Figure). These transporters are localized to the plant and yeast vacuole, respectively, and have been involved in iron and manganese sequestration into the vacuoles. The present of an iron transporter is in agreement with the detection of iron in acidocalcisomes of different species [78].

Tb9274.4960 is a member of the cation diffusion facilitator (CDF) family [79], which includes mammalian zinc transporters such as ZnT4 [80], *S. cerevisiae* ZRC1 [81], *A. thaliana* metal tolerance protein 1 (AtMTP1) [82], and *Escherichia coli* YiiP (EcYiiP) [83] (S10A Figure). These transporters function as antiporters of Zn, Cd, Co and/or Ni with protons. All known CDF domains proteins contain 6 TMD and share characteristic motifs, such as a CDF family-specific signature sequence at the start of the second membrane-spanning helix (TM2), and a long C-terminus [82]. The presence of this zinc transporter is in agreement with the abundant presence of zinc in the acidocalcisomes, as detected by X-ray microanalyses of different prokaryotes and eukaryotes [5], [78].

We also report the localization of some proteins not previously investigated, such as a mitochondrial ABC transporter (Tb927.11.540) (TbABCT), a flagellar cation/proton antiporter (Tb927.11.840.1) or flagellar tip protein (TbFTP), a nuclear periphery protein (Tb927.10.3640) (TbNP), a lysosome/acidocalcisome putative polyamine transporter (Tb927.9.10340) (TbPOT1) (S10B Figure), and a lysosomal putative polyamine transporter (Tb927.11.6680) (TbPOT2). We also confirmed the Golgi and lysosomal localization of TbGLP1 [53].

Finally, we report the requirement for growth of two subunits of the V-H^+^-ATPase (TbVA*a* and TbVA*d*), and of an orthologue of a vacuolar iron transporter (TbVIT1) in both PCF and BSF trypanosomes, supporting the role of acidocalcisomes in parasite growth and survival.

The identification of novel acidocalcisome proteins provides useful insights into the biogenesis of these organelles. A common feature of all the acidocalcisome proteins validated by endogenous expression with HA-tags in this study is the presence of one or more tyrosine-based, sorting signals with the YXXØ (Ø corresponds to an hydrophobic amino acid) consensus motif (see S7 Table). The μ subunits of at least four of the adaptor protein (AP) complexes bind to this motif [84]. In this regard, AP-3 is required for the biogenesis of the acidocalcisomes [63]. All of the proteins we validated by expression also possess generic N-glycosylation motifs, phosphothreonine modules binding FHA domains with large aliphatic amino acids at the pT+3 position as well as casein kinase 2 (CK2), glycogen synthase kinase β (GSK3β) and NEK2 (never in mitosis (NimA)-related kinases 2) phosphorylation sites (see S7 Table). A variety of kinases such as GSK3β localize to the Golgi and regulate post-Golgi membrane trafficking [85]. These findings will help guiding future studies on the biogenesis of these organelles.

In summary, in addition to validate the expression at the protein level of a number of important genes and identify the localization of proteins not previously studied, we identified several new acidocalcisome proteins using a strategy complementing subcellular proteomics and bioinformatics with their localization using *in situ* epitope-tagged proteins or specific antibodies, and RNAi for functional validation. Four of these proteins are newly identified acidocalcisome proteins, and their identification will facilitate further studies to elucidate the roles of this organelle in *T. brucei* physiology.

## Materials and Methods

### Ethics statement

Mice experiments in this work followed a reviewed and approved protocol by the Institutional Animal Care and Use Committee (IACUC). Animal protocols followed the US Government principles for the Utilization and Care of Vertebrate animals. The University of Georgia IACUC approved the animal protocol (Protocol number A2012-3-010).

### Cell culture

*T. brucei* PCF trypanosomes (wild type and 29-13 strains) and BSF (single marker (SM) strains) were used. PCF 29-13 (*T7RNAP NEO TETR HYG*) co-expressing T7 RNA polymerase and *Tet* repressor were a gift from Dr. George A. M. Cross (Rockefeller University, NY) and were grown in SDM-79 medium [86], supplemented with hemin (7.5 µg/mL) and 10% heat-inactivated fetal bovine serum, and at 27°C in the presence of G418 (15 µg/ml) and hygromycin (50 µg/ml) to maintain the integrated genes for T7 RNA polymerase and tetracycline repressor, respectively [87]. BSF trypanosomes (single marker strain) were also a gift from Dr. G.A.M. Cross and were grown at 37°C in HMI-9 medium [88] supplemented with 10% fetal bovine serum (FBS), 10% serum plus (JRH Biosciences, Inc.), and 2.5 µg/ml G418.

### Chemicals and reagents

TRIzol reagent, *Taq* polymerase, Magic Marker protein standards, BenchMark protein ladder, Mito-Tracker Red, and Alexa-conjugated secondary antibodies were purchased from Life Technologies (Carlsbad, CA). The expression vector pET32 EK/Lic was purchased from Novagen (Madison, WI). *E. coli* OverExpression C43 (DE3) strain was purchased from Lucigen (Middleton, WI). [α-^32^P]dCTP (3,000 Ci mmol^−1^) was from Perkin Elmer (Waltham, Massachusetts). Rabbit antibodies against *T. brucei* vacuolar H^+^-pyrophosphatase (TbVP1) [29] were a gift from Dr. Norbert Bakalara (Ecole Nationale Supérieure de Chimie de Montpellier, Montpellier, France). Mouse monoclonal antibody against HA (purified HA.11 clone 16B12) was purchased from Covance Inc. (Princeton, NJ). Rat monoclonal antibody against HA (clone 3F10) and Complete, EDTA-free protease inhibitor cocktail tablets were purchased from Roche Applied Science (Indianapolis, IN). The pMOTag4H vector [89] was a gift from Dr. Thomas Seebeck (University of Bern, Bern, Switzerland). The p2T7^Ti^ vector [90] was a gift from Dr. John Donelson (University of Iowa, Iowa City, IA). Antibody against GRASP [45] was a gift Dr. Graham Warren (Max F. Perutz Laboratories, Vienna, Austria), and antibodies against p67 and TbCATL [46] were a gift from Dr. James Bangs (University of Wisconsin, Madison, WI). Rabbit polyclonal antibody against TbVDAC was a gift from Dr. Minu Chadhuri (Meharry Medical College, TN). Anti *T. brucei* pyruvate, phosphate dikinase (PPDK)-producing mouse hybridoma culture supernatant was a gift from Dr. Frédéric Bringaud (University of Bordeaux, France). The enhanced chemiluminescence (ECL) detection kit was purchased from Amersham Biosciences (GE Healthcare Life Sciences, Piscataway, NJ), and Pierce ECL Western blotting substrate was from Thermo Fisher Scientific Inc. (Rockford, IL). The Bradford protein assay reagent, Precision Plus Protein WesternC pack, 4–15% polyacrylamide Ready gels, Zeta-Probe GT Genomic Testing blotting and nitrocellulose membranes were from Bio-Rad (Hercules, CA). AMAXA Human T-cell Nucleofector kit was purchased from Lonza (Koln, Germany). Prime-a Gene Labeling System was from Promega (Madison, WI). QIAquick gel extraction kit and MinElute PCR purification kit, Ni-NTA agarose, and Protein G Agarose Resins were from Qiagen (Valencia, CA). The primers were purchased from Integrated DNA Technologies (Coralville, IA). All other reagents of analytical grade were from Sigma (St. Louis, MO).

### Subcellular fractionation of acidocalcisomes and 1-D gel electrophoresis

Fractions enriched in acidocalcisomes were isolated and purified using two iodixanol gradient centrifugations (S1 Figure). PCF trypanosomes (3–4 g wet weight) were washed twice with Buffer A (116 mM NaCl, 5.4 mM KCl, 0.8 mM MgSO_4_, 50 mM Hepes, pH 7.2) with 5.5 mM glucose. The parasites were washed once in cold isolation buffer (125 mM sucrose, 50 mM KCl, 4 mM MgCl_2_, 0.5 mM EDTA, 20 mM Hepes, 3 mM dithiothreitol (DTT) supplied with Complete, EDTA-free, protease inhibitor cocktail (Roche) prior to lysis with silicon carbide in isolation buffer. Silicon carbide and cell debris were eliminated by a series of low speed centrifugations (100 *g* for 5 min, 300 *g* for 10 min, and 1,200 *g* for 10 min). The supernatant was centrifuged at 15,000 *g* for 10 min, and the pellet was resuspended in 1 ml isolation buffer and applied to the 34% step of a discontinuous gradient with 4 ml steps of 20, 24, 28, 34, 37 and 40% iodixanol (diluted in isolation buffer). The gradient was centrifuged at 50,000 *g* in a Beckman JS-24.38 rotor for 60 min at 4°C, and fractions were collected from the top. The pellet was resuspended in 700 µl isolation buffer and applied to the 27% step of another discontinuous gradient of iodixanol, with 1.4 ml of isolation buffer containing 10% w/v sucrose over-layered on the top and 1 ml steps of 27, 62 and 80% iodixanol, which were diluted from 90% w/v iodixanol with isolation buffer. To prepare 90% w/v iodixanol, 60% w/v iodixanol solution (Optiprep) was dried completely at 70°C and resuspended with isolation buffer. After the second gradient centrifugation at 50,000 *g* for 60 min at 4°C, fractions were collected from the top, washed twice with isolation buffer by centrifugation at 20,000 g for 15 min at 4°C, and analyzed by various organelle marker enzyme assays. The protein concentration was quantified by Bradford assay using a SpectraMax Microplate Reader. After washing fraction 5, containing the highest vacuolar-H^+^-pyrophosphatase (PPase) activity (Fig. 1A), it was resuspended in 200-µl isolation buffer. Aliquots of the purified acidocalcisome suspension were separated on 4–15% SDS-PAGE gels and stained with Coomassie brilliant blue, immunoblotted with several acidocalcisome markers, precipitated for electron microscopy, or used for proteomic analysis. Chromatograms of protein bands in the SDS-PAGE gels were obtained after background subtraction using ImageJ (National Institute of Health, Bethesda, MD).

### In-gel tryptic digestion

Gel lanes were washed twice in ddH_2_O for 15 min and cut into 10 equal slices. Proteins were reduced in a 10 mM dithiothreitol (DTT)/100 mM ammonium bicarbonate solution at 65°C for 1 h and carboxyamidomethylated with 55 mM iodoacetamide/100 mM ammonium bicarbonate for 1 h at room temperature in the dark. Enzymatic digestion was performed with porcine trypsin (1∶50, Promega, Madison, WI) at 37°C overnight. Tryptic peptides were extracted two times with 100 µl of 50% acetonitrile/0.1% formic acid. Combined extracts were evaporated to dryness and stored at −20°C until mass spectrometry analysis.

### Mass spectrometry

Peptides were resuspended in 20 µl of 2% acetonitrile/0.1% formic acid. Data was acquired using an Agilent 1100 Capillary LC system (Palo Alto, CA) with a 0.2×150 mm Halo Peptide ES-C18 capillary column packed with 2.7 µm diameter superficially porous particles (Advanced Materials Technology, Inc., Wilmington, DE). On-line MS detection used the Thermo-Fisher LTQ ion trap (San Jose, CA) with a Michrom (Michrom Bioresources, Auburn, CA) captive spray interface. Sample analysis utilized the LTQ divert valve fitted with an EXP Stem Trap 2.6 µL cartridge packed with Halo Peptide ES-C18 2.7 µm diameter superficially porous particles (Optimize Technologies, Oregon City, OR). Sample injection volume was 8 µl. Gradient conditions increased the concentration of mobile phase B from 6% to 75% B over 90 min. Mobile phase A consisted of 99.9% water, 0.1% formic acid and 10 mM ammonium formate. Mobile phase B contained 80% acetonitrile, 0.1% formic acid and 10 mM ammonium formate. Mobile phases used formic acid, ammonium formate and acetonitrile from Sigma-Aldrich (St. Louis, MO).

Raw tandem mass spectra were converted to mzXML files, then into mascot generic files (MGF) via the Trans-Proteomic Pipeline (Seattle Proteome Center, Seattle, WA). MGF files were searched using Mascot (Matrix Scientific Inc, Boston, MA) against separate target and decoy databases obtained from the National Center for Biotechnology Information (NCBI). The target database contained all *T. brucei* protein sequences and the decoy database contained the reversed sequences from the target database. Mascot settings were as follows: tryptic enzymatic cleavages allowing for up to 2 missed cleavages, peptide tolerance of 1000 parts-per-million, fragment ion tolerance of 0.6 Da, fixed modification due to carboxyamidomethylation of cysteine (+57 Da), and variable modifications of oxidation of methionine (+16 Da) and deamidation of asparagine or glutamine (+0.98 Da). Mascot files were loaded into ProteoIQ (NuSep, Bogart, GA), where a 1% false discovery rate and a 0.9 peptide probability were applied for confirmation of protein identifications. The ProteinProphet algorithm utilized by ProteoIQ software combines hit proteins with degenerate peptide fingerprints into a single identification (a protein “group”) and generates a group probability. In these cases, one or more of the individual proteins may actually be present in the sample.

### Bioinformatic analysis of mass spectrometry results

Subcellular fractionation protocols enrich samples for target organelles but produce somewhat heterogeneous preparations containing material from other cell compartments that are readily detected by exquisitely sensitive tools such as mass spectrometry. To identify likely contaminants from non-acidocalcisomal compartments in our proteomic dataset, we used a series of subcellular prediction algorithms: TargetP 1.1 [91], pTARGET [92], SLP-LOCAL [93], and WoLF-PSORT [94]. Data from each of these algorithms was processed using Perl scripts and a MySQL database to screen for proteins with prediction confidence thresholds of 80%. Final consensus predictions of subcellular localization for individual protein hits were assigned when two or more algorithms agreed. In the event when the mass spectrometry data identified a protein group with more than one member, consensus predictions for individual proteins were combined into a group consensus prediction when predictions between at least two individual proteins agreed. The membrane topology and presence of signal peptides and was predicted using the following tools: SignalP3 [95], TMHMM2.0c [96], HMMTOP2.1 [97] and PolyPhobius [98], [99] (accessed May 28, 2014). In addition, we also used published data for annotated proteins to validate our data.

### Enzyme assays

Pyrophosphatase (PPase) activity (acidocalcisome marker) was assayed by measuring phosphate (P_i_) release using the malachite green assay [100] with some modifications. Briefly, reactions contained 130 mM KCl, 2 mM MgCl_2_, 10 mM Hepes, pH 7.2, 100 µM PP_i_, 0.5 µg of gradient fraction with or without 40 µM aminomethylenediphosphonate (AMDP). After incubation at 30°C for 10 min, the reaction was stopped by the addition of an equal volume of freshly prepared mixture of three parts of 0.045% malachite and one part of 4.2% ammonium molybdate. The absorbance (*A*) at 660 nm was read using the microplate reader. The amount of P_i_ released was determined by comparison with a standard curve. AMDP was used to distinguish between vacuolar (sensitive) and soluble (insensitive) PPase activities. The specific activity of TbVP1 was defined as µmol P_i_ released/min×mg of protein.

Succinate-cytochrome C reductase activity (mitochondria marker) was assayed as described previously [101], using 3 mM succinate (pH 7.2) as the substrate and following the reaction containing 0.1 mM cytochrome C (Cyt C), 0.3 mM KCN, 40 mM Hepes pH 7.5, and 10 µl of gradient fraction at 30°C at 550-540 nm in the microplate reader. Hexokinase (glycosome marker) was assayed as described previously [102]. The reaction mixtures (100 µl) contained 10 mM D-glucose, 0.6 mM ATP, 0.6 mM NADP^+^, 10 mM MgCl_2_, 2.5 units/ml glucose-6-phosphate dehydrogenase, and 50 mM potassium Hepes, pH 7.8. The oxidation of NADP was monitored at 30°C in the microplate reader at 340–430 nm.

Alpha-mannosidase activity (lysosome marker) was assayed using p-nitrophenyl-α-D-mannopyranoside (pNP-Man) as substrate as described previously [103]. The reaction mixtures contained 200 mM sodium acetate buffer (pH 4.6), 0.6 mM pNP-Man and 10 µl of gradient fraction in a total volume of 100 µl. The mixture was incubated at 30°C for 30 min, and the reaction was terminated by the addition of 160 µl of 1 M Na_2_CO_3_. Two hundred microliter of the final mixture was transferred to a microtitre plate and read at 405 nm using the micro plate reader. 1 unit of activity corresponds to the hydrolysis of 1 µmol of substrate/min at 30°C. The α-mannosidase activity was expressed as µmol/min×mg protein.

### Electron microscopy

Aliquots (25 µl) of the 15,000×g pellet fraction, the pellet of the first gradient and fraction 5 of the second gradient (Fig. 1 and S1 Figure) were precipitated by centrifugation at 20,000 *g* for 15 min at 4°C. The pellets were fixed in 2.5% glutaraldehyde and 4% paraformaldehyde in 0.1 M sodium cacodylate buffer (pH 7.4) at room temperature for 1 h. The supernatants were carefully replaced with fresh fixative without disturbing the pellets and then stored 4°C. Samples were processed for transmission electron microscopy at the Electron Microscopy Laboratory at the University of Georgia College of Veterinary Medicine.

### Generation of epitope tagging cassettes and RNAi constructs

The one-step epitope-tagging protocol reported by Oberholzer et al. [89] was used to produce 14 C-terminal HA-tagging cassettes (TriTrypDB gene ID numbers listed in Table 2) for transfection of *T. brucei* PCF trypanosomes. In brief, the PCR forward and reverse primers included terminal 100–120 nucleotides of each ORF before its stop codon and the reverse complement of the first 100–120 nucleotides of the 3′UTR, respectively, followed in frame by the 21–26 nucleotides of the backbone sequences of pMOTag vector series [89]. The HA-tagging cassettes containing a hygromycin resistant gene as a selection marker were generated for cell transfection by PCR using pMOTag4H as template with the corresponding PCR primers of the gene.

To knockdown the expression of the *TbVAa*, *TbVAd*, *TbVIT*, or *TbZnT* genes (TriTrypDB gene ID numbers listed in Table 2) by double-stranded RNA expression, the inducible T7 RNA polymerase-based protein expression system and the p2T7^Ti^ vector with dual-inducible T7 promoters were employed. A cDNA fragment (ranging from 566 to 757 bp) of the genes targeted to nucleotides (*TbVAa*: 310–876, *TbVAd*: 364–1121, *TbVIT*: 125–755, *TbZnT*: 620–1241) of the open reading frames (ORFs) was amplified using the forward and reverse primers listed in S6 Table, digested with restriction enzymes (BamHI and HindIII), and cloned into p2T7^Ti^ vector. The recombinant constructs were confirmed by sequencing at the DNA Analysis Facility at Yale University (New Heaven, CT), NotI-linearized, and purified with QIAGEN's DNA purification kit for cell transfections.

### Cell transfection

Mid-log phase PCF (∼5×10^6^ cells/ml) were harvested by centrifugation at 1,000 g for 7 min, washed with Cytomix buffer (2 mM EGTA, 3 mM MgCl_2_, 120 mM KCl, 0.5% glucose, 0.15 mM CaCl_2_, 0.1 mg/ml BSA, 10 mM K_2_HPO_4_/KH_2_PO_4_, 1 mM hypoxanthine, 25 mM Hepes, pH 7.6) and resuspended in 0.45 ml of the same buffer at a cell density of 2.5×10^7^ cells/ml. The washed cells were mixed with 50 µl of NotI-linearized plasmid DNA or purified PCR products (10 µg) in a 0.4-cm electroporation cuvette and subjected to two pulses from a Bio-Rad Gene Pulser electroporator set at 1.5 kV and 25 µF. The stable transformants were obtained in SDM-79 medium supplemented with 15% FBS plus appropriate antibiotics (5 µg/ml phleomycin, 50 µg/ml hygromycin and 15 µg/ml G418).

For the BSF, 10 µg of NotI-linearized plasmid DNA (<10 µl) were used per 4×10^7^ mid-log phase cells in 100 µl AMAXA Human T-cell Nucleofector solution. Electroporation was performed using 2 mm gap cuvettes with program X-001 of the AMAXA Nucleofector. Following each transfection, stable transformants were selected and cloned by limiting dilution in HMI-9 medium containing 15% FBS with appropriate antibiotics (2.5 µg/ml phleomycin and 2.5 µg/ml G418) in 24-well plates. Antibiotic-resistant clones were further characterized as described below. The correct epitope-tagging of the target genes was confirmed by PCR followed by sequencing and Western blot analyses. RNAi was induced with 1 µg/ml fresh tetracycline when the cells were at a density of 2×10^6^ PCF or 1×10^5^ BSF/ml.

### Anti-TbIP_3_R antibodies

The cDNA fragment of *TbIP_3_R* encoding a putative IP_3_ binding domain (amino acids 329–804) [27] was amplified by PCR using primers TbIP3BD-F and TbIP3BD-R (S6 Table) and cloned in frame into the expression vector pET32 EK/Lic (Novagen) to generate pET32(*TbIP_3_R-BD*). The correct plasmid pET32(*TbIP_3_R-BD*) was confirmed by sequencing and then transformed into *E. coli* OverExpress C43 (DE3) strain (Lucigen, WI). His-tagged TbIP_3_R-BD fusion protein was affinity purified with Ni-NTA agarose (Qiagen) based on the manufacturer's protocol. The purified protein was used to immunize mice and polyclonal antibodies were purified from anti-serum with Protein G Agarose Resins (Qiagen).

### Immunofluorescence microscopy

When Mitotracker Red CMXRos (Invitrogen) was used, live cells were labeled for 30 min with the red-fluorescent dye at 50 nM in trypanosome culture medium. PCF trypanosomes were washed with PBS and then fixed with 4% paraformaldehyde in PBS at room temperature for 1 h. The fixed parasites were washed twice with PBS, allowed to adhere to poly-L-lysine-coated coverslips, and permeabilized with 0.3% Triton X-100/PBS for 3 min for PCF. After blocking with PBS containing 3% BSA, 1% fish gelatin, 50 mM NH_4_Cl and 5% goat serum for 1 h, trypanosomes were stained in 3% BSA/PBS with the polyclonal rabbit antibody against TbVP1 (1∶500), mouse polyclonal antibody against TbIP_3_R-BD (1∶100), purified HA.11 clone 16B12 mouse monoclonal antibody against HA (1∶50), rat monoclonal antibody against HA (1∶100) (Roche), rabbit anti-GRASP antibody (1∶100), mouse anti-p67 monoclonal antibody (1∶200), rabbit anti-trypanopain (TbCATL) antibody (1∶600) for 1 h. After thoroughly washing with PBS containing 3% BSA, cells were incubated with Alexa 488-conjugated goat anti-mouse or anti-rat antibody, and Alexa 546-conjugated goat anti-rabbit or anti-mouse antibody at 1∶1,000 for 1 h. The cells were counterstained with DAPI before mounting with Gold ProLong Gold antifade reagent (Molecular Probes). Differential interference contrast (DIC) and fluorescent optical images were captured using an Olympus IX-71 inverted fluorescence microscope with a Photometrix CoolSnap^HQ^ CCD camera driven by DeltaVision software (Applied Precision, Seattle, WA). Images were deconvolved for 15 cycles using Softwarx deconvolution software. Pearson's correlation coefficients (PCC) were calculated using the Softwarx software by measuring the images of whole cells or specific cell-staining regions.

### Western blot analyses

The cells were harvested and washed twice in PBS. The washed cells or aliquots of purified acidocalcisome suspension were lysed with RIPA buffer (150 mM NaCl, 20 mM Tris/HCl, pH 7.5, 1 mM EDTA, 1% SDS, and 0.1% Triton X-100) containing protease inhibitor tablet (Roche) in ice for 1 h. The protein concentration was determined by using Pierce BCA protein assay kit with the microplate reader. The total cell lysates were mixed with 2× Laemmli sample buffer (BioRad) at 1∶1 ratio (volume/volume) and directly loaded. The separated proteins were transferred onto nitrocellulose membranes using a Bio-Rad transblot apparatus. The membranes were blocked with 10% non-fat milk in PBS containing 0.5% Tween-20 (PBS-T) at 4°C overnight. The blots were incubated with rabbit antibodies against TbVP1 (1∶5,000), rabbit antibodies against TbVDAC (1∶2,000), mouse antibodies against TbPPDK (1∶200), mouse antibodies against Tbp67 (1∶3,000), rabbit antibodies against TcVSP (1∶5,000), mouse antibodies against TbIP_3_R (1∶1,000), mouse antibodies against HA (1∶1,000), or mouse antibodies against tubulin (1∶20,000) for 1 h. After five washings with PBS-T, the blots were incubated with horseradish peroxidase conjugated anti-mouse or anti-rabbit IgG (H+L) antibody at a dilution of 1∶20,000 for 1 h. After washing five times with PBS-T, the immunoblots were visualized using Pierce ECL Western blotting substrate according to the manufacturer's instructions.

### Northern blot analysis

Total RNA was isolated with TRIzol reagent and treated with DNA-*free* following the manufacturer's instructions. RNA samples (10 µg/lane) were fractionated on 1% agarose/formaldehyde gels, transferred to Zeta-Probe nylon membranes by capillary action, and fixed onto the membranes by baking at 80°C for 1 h. The probes for *TbVAa*, *TbVAd*, *TbVIT* and *TbZnT* were generated by PCR using the same set of primers (S6 Table) from the corresponding RNAi constructs in p2T7^Ti^ as described above and labeled with [α-^32^P]-dCTP using a Prime-a-Gene Labeling System according to the manufacturer's protocol. The [α-^32^P]-dCTP-labeled probe of *Tb-β-tubulin* gene (GeneDB Tb927.1.2390) was generated from *T. brucei* genomic DNA by PCR using gene-specific primers TbTubb-F and TbTubb-R (S6 Table). RNA-bound membranes were hybridized with the ^32^P-labeled probes in 0.5 M Na_2_HPO_4_, pH 7.4 and 7% SDS at 65°C overnight with agitation. After hybridization, the membranes were washed twice for 10 min each at 68°C with 1× SSC and 0.1% SDS and twice for 30 min at 65°C with 0.1× SSC and 0.1% SDS. Northern blots were visualized by autoradiography, and quantified by using ImageJ (National Institute of Health, Bethesda, MD).

## Supporting Information

S1 Figure **Subcellular fractionation of acidocalcisomes.** (A) Trypanosome lysates were obtained by grinding with silicon carbide, decanted by low speed centrifugation to eliminate debris and silicon carbide, and centrifuged at 15,000 g for 10 min to isolate the organellar fraction that was applied to the 34% step of a discontinuous iodixanol gradient. After centrifugation at 50,000 g for 1 h, the pellet was resuspended and applied to the 27% step of a second iodixanol gradient and centrifuged at 50,000 g for 1 h. Aliquots from each fraction were used for enzymatic assays. (B) Electron microscopy of acidocalcisome fraction prepared by the iodixanol procedure (fraction 5). *Scale bar* = 0.2 µm. *Arrows* and *arrowheads* show electron-dense material inside acidocalcisomes.
(TIF)

S2 Figure **SDS-PAGE, immunoblots, and electron micrographs of subcellular fractions.** (A–B) SDS-PAGE and immunoblot analyses of the 15,000×g pellet (P1, 30 µg), the first gradient pellet (P2, 2 µg), and the second gradient fractions (F1 to F7, 2 µg each). The SDS-PAGE gel (A) was stained with Coomassie brilliant blue. BenchMark protein molecular markers (M) are shown at the left. Western blot analyses (B) were done using antibodies against acidocalcisome marker TbVP1, mitochondrial marker voltage-dependent anion channel (TbVDAC), glycosomal marker pyruvate, phosphate dikinase (TbPPDK), and lysosome marker Tbp67. M, Magic Marker protein standards. (C–D) Electron microscopy of the 15,000×g pellet or P1 (C) and the pellet obtained after the first gradient centrifugation or P2 (D). Arrows indicate electron-dense acidocalcisomes, and other organelles. M, mitochondria; G, glycosome, Ac, acidocacisome (note electron-dense material in some of them). Scale bar = 0.5 µm.
(TIF)

S3 Figure **Proteins present in fraction 5.** SDS-PAGE (*left panels*) and western blot analyses (*right panels*) of fraction 5 from three representative fractionations. The SDS-PAGE gels were stained with Coomassie brilliant blue. BenchMark protein molecular markers are shown at the *left* for all gels. Western blot analyses were done using antibodies against TbVP1 (A), TbIP_3_R (B), and TcVSP (C), as described under Materials and Methods. *Arrowheads* in A and B, and *arrow* in C show the reactions of antibodies with the bands of expected size. *Arrowhead* in C probably corresponds to the reaction with a soluble pyrophosphatase.
(TIF)

S4 Figure **Immunofluorescence microscopy and western blot analysis of V-H^+^-ATPase subunit** ***d*** **(TbVA** ***d*** **) in PCF trypanosomes.** V-H^+^-ATPase subunit *d* co-localize with TbVP1 to the acidocalcisomes (A), with TbGRASP to the Golgi complex (B), and with TbCATL (C) and p67 (D) to lysosomes (Pearson's correlation coefficients of 0.625, 0.561, 0.785, and 0.796 respectively). *Yellow* in merge images indicate co-localization (also shown with *arrows* in (B–D)). *Scale bars* for A–D = 10 µm. (E) Confirmation of tagging by western blot analyses with monoclonal anti-HA in PCF trypanosomes. HRP-conjugated goat anti-mouse was used as a secondary antibody. Precision Plus Protein WesternC marker (Bio-Rad) was used for the molecular weight markers. *Arrow* indicates band corresponding to TbVA *d*. Tubulin (*Tub*) was used as a loading control (*bottom panel*).
(TIF)

S5 Figure **Localization of other proteins.** (A) Epitope-tagged TbNP localizes to the nuclear membrane. (B) TbABCT co-localizes with MitoTracker (Pearson's correlation coefficient of 0.688). *Yellow* in merge images indicate co-localization. *Scale bars* for A–B = 10 µm. (C–D) Tagging with HA was confirmed by western blot analyses using anti-HA antibodies. Markers are at the *left* side and *arrows* indicate the corresponding bands. Equivalent amounts of wild type cell (WT) proteins were loaded as evidenced by the similar background to the test lanes.
(TIF)

S6 Figure **Localization of other proteins.** Epitope-tagged TbGLP1 does not co-localize with TbVP1 (A) but co-localizes with TbGRASP to the Golgi complex (B), and with TbCATL (C) and p67 (D) to the lysosome (Pearson's correlation coefficients of 0.5369, 0.8050 and 0.8426, respectively). *Yellow* in merge images indicate co-localization (also shown with *arrows* in (B–D)). *Scale bars* for A–D = 10 µm. (E) Tagging with HA was confirmed by western blot analyses using anti-HA antibodies. Markers are at the left side and arrow shows the band corresponding to TbGLP1. Equivalent amounts of wild type cell (WT) proteins were loaded as evidenced by the similar background to the test lanes.
(TIF)

S7 Figure **Immunofluorescence microscopy and western blot analysis of polyamine transporters TbPOT1.** Epitope-tagged TbPOT1 partially co-localizes with TbVP1 to the acidocalcisomes (A) and co-localizes with TbCATL (B) and p67 (C), to the lysosomes (Pearson's correlation coefficients of 0.4064, 07191, and 0.6710, respectively). *Arrrows* in merge images show the co-localization. (D) A putative cation/proton antiporter localizes to the falgellar tip (*white arrow*) and was named flagellar tip protein (TbFTP). (E, F) Tagging with HA was confirmed by western blot analyses using anti-HA antibodies. Markers are at the *left* side and *arrows* shows the band corresponding to TgGLP1, and TbFTP, respectively. Equivalent amounts of wild type cell (WT) proteins were loaded as evidenced by the similar background to the test lanes.
(TIF)

S8 Figure **Immunofluorescence microscopy and western blot analysis of polyamine transporters TbPOT2.** Epitope-tagged TbPOT2 does not co-localize with TbGRASP to the Golgi complex (A) but it co-localizes with TbCATL (B) and p67 to the lysosomes (C) (Pearson's correlation coefficients of 0.8806, 0.8404, respectively). *Scale bars* for A–C = 10 µm. (D) Tagging with HA was confirmed by western blot analyses using anti-HA antibodies. Markers are at the left side. Arrow indicate band corresponding to TbPOT2. Equivalent amounts of wild type cell (WT) proteins were loaded as evidenced by the similar background to the test lanes.
(TIF)

S9 Figure **Comparison of three newly identified acidocalcisome proteins and their homologues with known functions from other organisms.** Multiple protein sequence alignments of (A) phosphate transporters from *S. cerevisiae* (ScPho91p, accession number CAY82206), *T. cruzi* (TcPho91, TcCLB.508831.60), and *T. brucei* Pho91 (TbPho91, Tb927.11.11160). (B) Acid phosphatases from *Homo sapiens* (HsTRAP, P13686) and *T. brucei* (TbAP, Tb927.10.7020). (C) Vacuolar iron transporters from *A. thaliana* (AtVIT1, NP_178286), *S. cerevisiae* (ScVIT1, DDA09536), and *T. brucei* (TbVIT1, Tb927.3.800). The protein sequences were analyzed *via* ClustalW2 at the EMBL-EBI website (http://www.ebi.ac.uk/Tools/msa/clustalw2/). The symbols “*”, “:”, and “.” represent identical, conserved, or semi-conserved amino acid (aa) substitutions, respectively. *Red*: small and hydrophobic aa (AVFPMILW); *blue*: acidic aa (DE); *magenta*: basic aa (RK); and *green*: hydroxyl, amine, and basic aa (STYHCNGQ).
(TIF)

S10 Figure **Comparison of two newly identified acidocalcisome proteins and their homologues with known functions from other organisms.** Multiple protein sequence alignments of (A) Zinc transporters from *Mus musculus* (MmZnT4, AAB82593), *S. cerevisiae* (ScZRC1, CAA88653.1), *A. thaliana* (AtMTP1, NP_850459), *E. coli* (EcYiiP, P69380.1), *T. cruzi* (TcZnT, TcCLB.511439.50), and *T. brucei* (TbZnT, Tb927.4.4960). (B) Polyamine transporters from *S. cerevisiae* (ScTPO1, Q07824) and *T. brucei* (TbPOT1, Tb927.9.10340). Analysis was done as in Fig. S9.
(TIF)

S1 Table ***T. brucei*** **proteins identified with high confidence (1% false discovery rate, protein probability ≥0.95) from fraction 5 datasets (ACCS1 and ACCS2).** Proteins with degenerate peptide (peptides shared among all members of the protein cluster) fingerprints are reported in a single protein “group” as described in the Methods.
(PDF)

S2 Table **Peptide list for all high confidence identifications (1% false discovery rate, protein probability ≥0.95) from the acidocalcisome data sets (ACCS1 and ACCS2).** When high-confidence identifications to similar proteins were identified but peptide degeneracy limited discrimination, peptides matched to these indistinguishable proteins are represented in a single protein “group” as described in the Methods.
(PDF)

S3 Table **Signal peptide (SP) and transmembrane domain (TMD) predictions of high confidence protein identifications (1% false discovery rate, protein probability ≥0.95) from** ***T. brucei*** **acidocalcisome datasets (ACCS1 and ACCS2).** Predictions of TMD and SP for individual proteins were based on consensus of two or more algorithms. If the predicted number of TMD varied among the different predictions packages, we report the median number of TMD. If a protein group contained more than one protein hit (see Methods for explanation of protein grouping based on degenerate peptide fingerprints), the number of TMD and presence of SP were assigned if predicted in at least two members.
(PDF)

S4 Table **Predicted subcellular locations for high-confidence protein groups (protein probability: 1% false discovery rate, p≥0.95,) identified in the** ***T. brucei*** **acidocalcisome fractions (ACCS1 and ACCS2) from our prediction servers using non-plant based algorithms.** Individual protein predictions are based upon agreement between at least two analysis packages. Consensus for identifications with multiple protein hits (see Methods for explanation of protein grouping based on degenerate peptide fingerprints) is given when prediction for a compartment agrees among two of more members of the group. S, secreted. C, cytosol. M, mitochondrion. N, nucleus. PM, plasma membrane. G, Golgi complex. ER, endoplasmic reticulum. P, peroxisome. L, lysosome. CYKS, cytoskeleton. Threshold probabilities and confidences used to screen our predictions with poor reliability: targetP (RC = 1). pTarget ≥80%, SLP-LOCAL ≥2, WoLfPsort ≥∼80%. WoLFPSORT thresholds for each subcellular location were derived from empirical prediction confidence statistics (wolfpsort.org/empiricalConfidenceByNumNeighbors/index.html, updated August 15, 2007).
(PDF)

S5 Table **Subunits of the vacuolar H^+^-ATPase present in the genome of** ***T. brucei*** **.**
(PDF)

S6 Table **Primers used to generate probes for blotting and constructs for antibody production or RNAi (the underlined nucleotides indicate the primer extension sequences for ligation independent cloning or the introduced HindIII and BamHI sites).**
(PDF)

S7 Table **Common features of confirmed acidocalcisome proteins identified by the ELM server.**
(PDF)
