# Supplementary figures and images for: Proteomic Analysis of the Acidocalcisome, an Organelle Conserved from Bacteria to Human Cells
Source: PLoS Pathog. 2014 Dec 11;10(12):e1004555. doi: 10.1371/journal.ppat.1004555 (PMC4263762; doi:10.1371/journal.ppat.1004555)

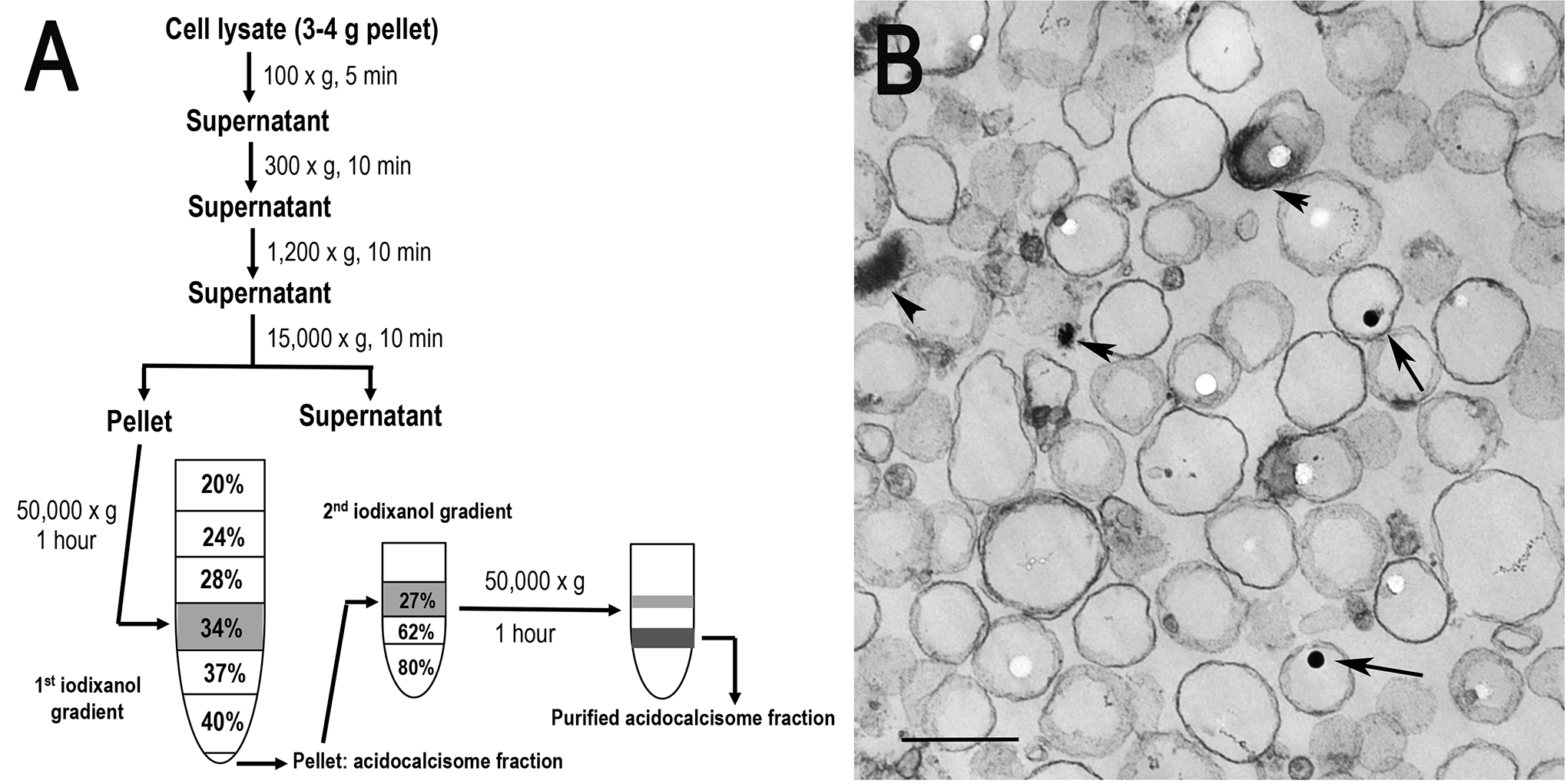

Supplement: S1 Figure — Subcellular fractionation of acidocalcisomes. (A) Trypanosome lysates were obtained by grinding with silicon carbide, decanted by low speed centrifugation to eliminate debris and silicon carbide, and centrifuged at 15,000 g for 10 min to isolate the organellar fraction that was applied to the 34% step of a discontinuous iodixanol gradient. After centrifugation at 50,000 g for 1 h, the pellet was resuspended and applied to the 27% step of a second iodixanol gradient and centrifuged at 50,000 g for 1 h. Aliquots from each fraction were used for enzymatic assays. (B) Electron microscopy of acidocalcisome fraction prepared by the iodixanol procedure (fraction 5). Scale bar = 0.2 µm. Arrows and arrowheads show electron-dense material inside acidocalcisomes. (TIF) [file ppat.1004555.s001.tif]

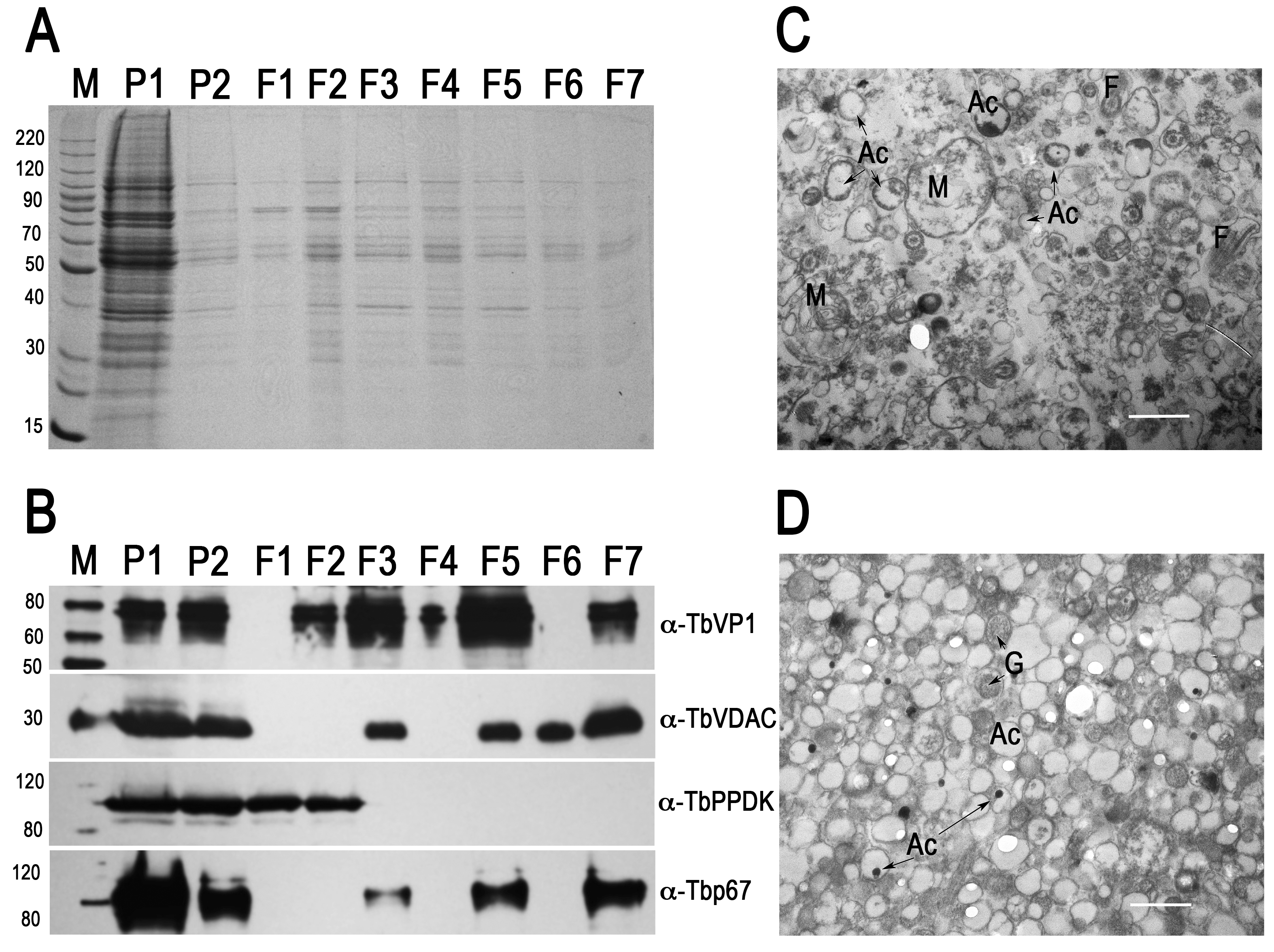

Supplement: S2 Figure — SDS-PAGE, immunoblots, and electron micrographs of subcellular fractions. (A–B) SDS-PAGE and immunoblot analyses of the 15,000×g pellet (P1, 30 µg), the first gradient pellet (P2, 2 µg), and the second gradient fractions (F1 to F7, 2 µg each). The SDS-PAGE gel (A) was stained with Coomassie brilliant blue. BenchMark protein molecular markers (M) are shown at the left. Western blot analyses (B) were done using antibodies against acidocalcisome marker TbVP1, mitochondrial marker voltage-dependent anion channel (TbVDAC), glycosomal marker pyruvate, phosphate dikinase (TbPPDK), and lysosome marker Tbp67. M, Magic Marker protein standards. (C–D) Electron microscopy of the 15,000×g pellet or P1 (C) and the pellet obtained after the first gradient centrifugation or P2 (D). Arrows indicate electron-dense acidocalcisomes, and other organelles. M, mitochondria; G, glycosome, Ac, acidocacisome (note electron-dense material in some of them). Scale bar = 0.5 µm. (TIF) [file ppat.1004555.s002.tif]

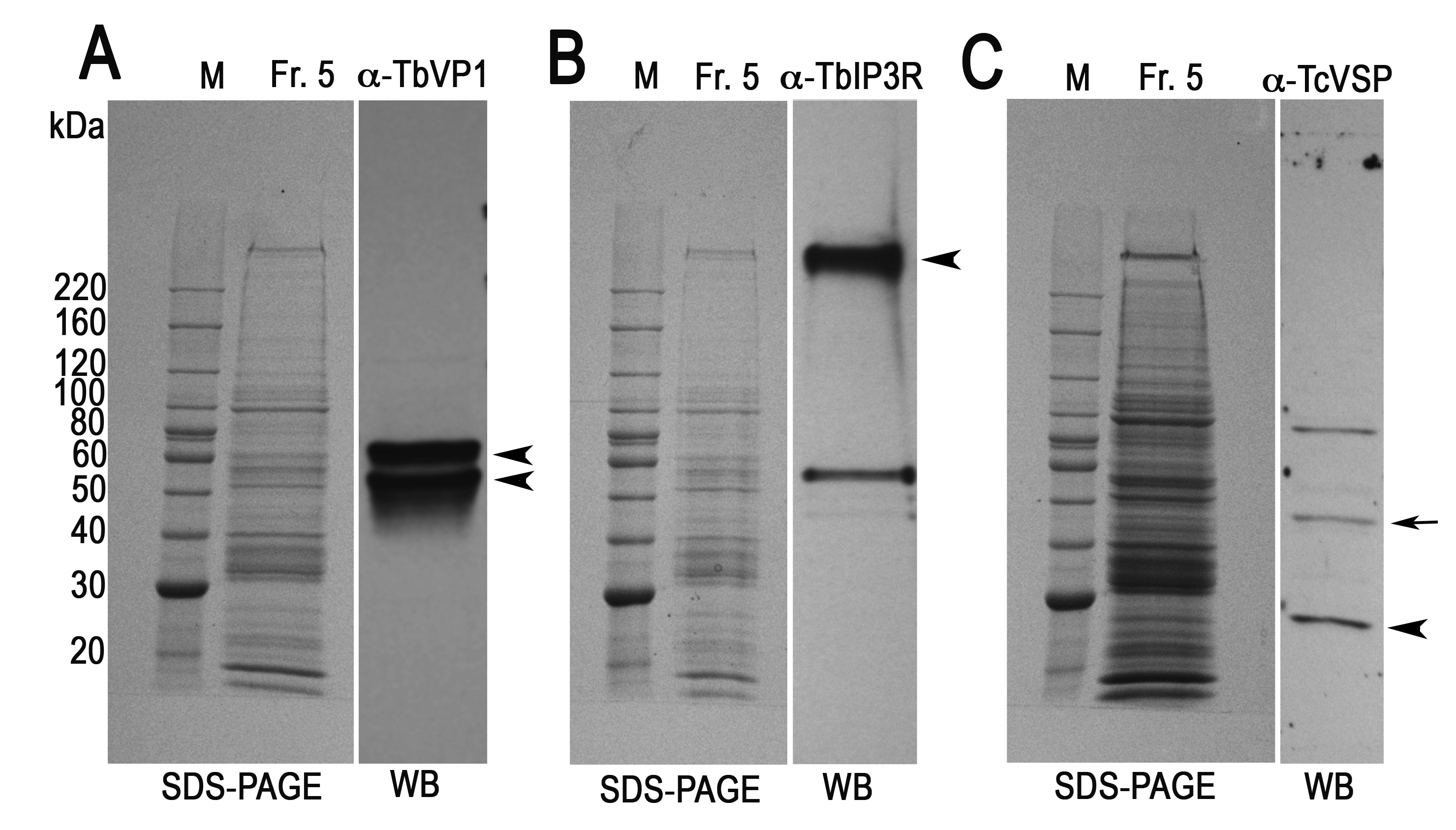

Supplement: S3 Figure — Proteins present in fraction 5. SDS-PAGE (left panels) and western blot analyses (right panels) of fraction 5 from three representative fractionations. The SDS-PAGE gels were stained with Coomassie brilliant blue. BenchMark protein molecular markers are shown at the left for all gels. Western blot analyses were done using antibodies against TbVP1 (A), TbIP3R (B), and TcVSP (C), as described under Materials and Methods. Arrowheads in A and B, and arrow in C show the reactions of antibodies with the bands of expected size. Arrowhead in C probably corresponds to the reaction with a soluble pyrophosphatase. (TIF) [file ppat.1004555.s003.tif]

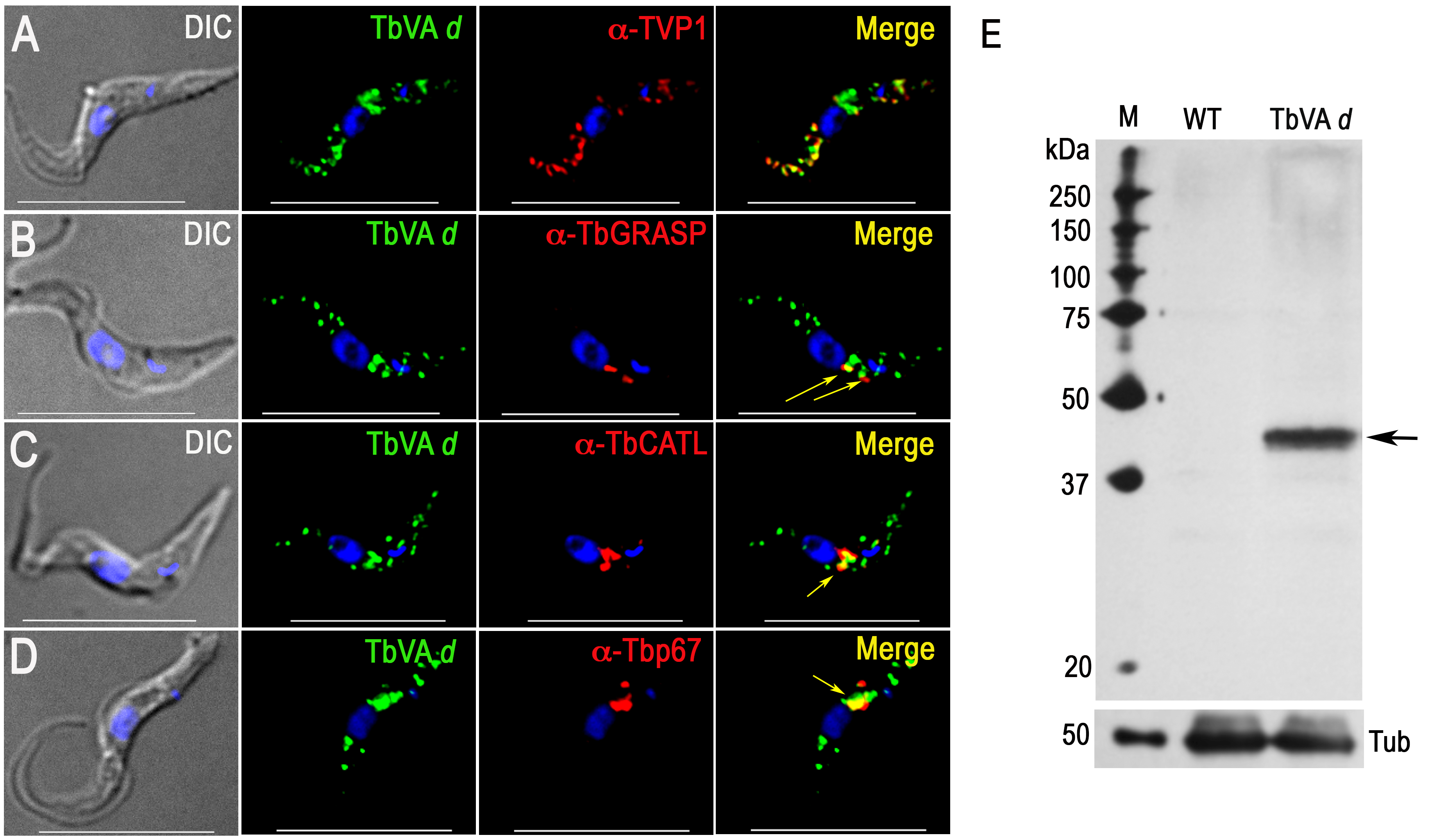

Supplement: S4 Figure — Immunofluorescence microscopy and western blot analysis of V-H+-ATPase subunit d (TbVA d ) in PCF trypanosomes. V-H+-ATPase subunit d co-localize with TbVP1 to the acidocalcisomes (A), with TbGRASP to the Golgi complex (B), and with TbCATL (C) and p67 (D) to lysosomes (Pearson's correlation coefficients of 0.625, 0.561, 0.785, and 0.796 respectively). Yellow in merge images indicate co-localization (also shown with arrows in (B–D)). Scale bars for A–D = 10 µm. (E) Confirmation of tagging by western blot analyses with monoclonal anti-HA in PCF trypanosomes. HRP-conjugated goat anti-mouse was used as a secondary antibody. Precision Plus Protein WesternC marker (Bio-Rad) was used for the molecular weight markers. Arrow indicates band corresponding to TbVA d. Tubulin (Tub) was used as a loading control (bottom panel). (TIF) [file ppat.1004555.s004.tif]

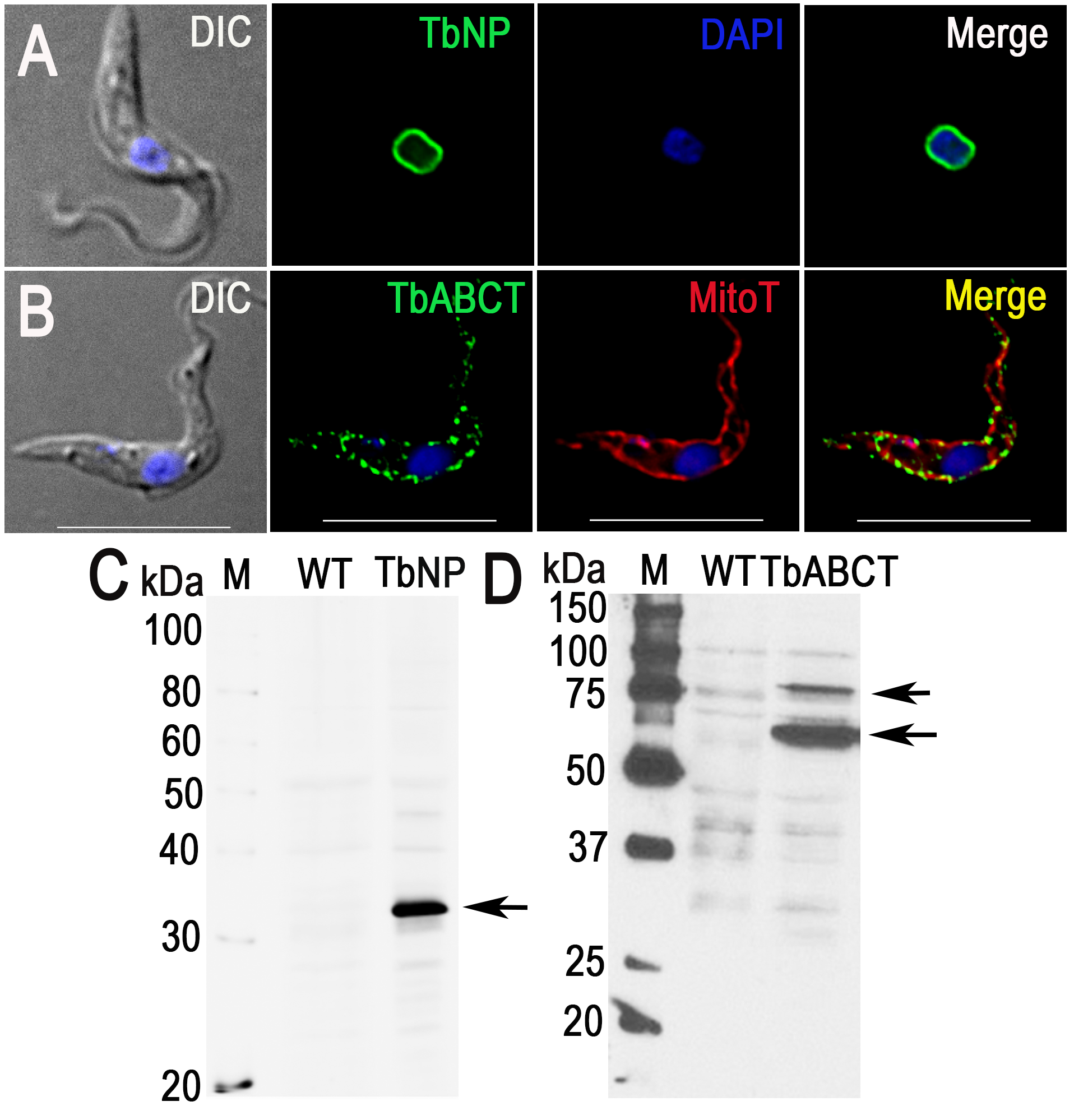

Supplement: S5 Figure — Localization of other proteins. (A) Epitope-tagged TbNP localizes to the nuclear membrane. (B) TbABCT co-localizes with MitoTracker (Pearson's correlation coefficient of 0.688). Yellow in merge images indicate co-localization. Scale bars for A–B = 10 µm. (C–D) Tagging with HA was confirmed by western blot analyses using anti-HA antibodies. Markers are at the left side and arrows indicate the corresponding bands. Equivalent amounts of wild type cell (WT) proteins were loaded as evidenced by the similar background to the test lanes. (TIF) [file ppat.1004555.s005.tif]

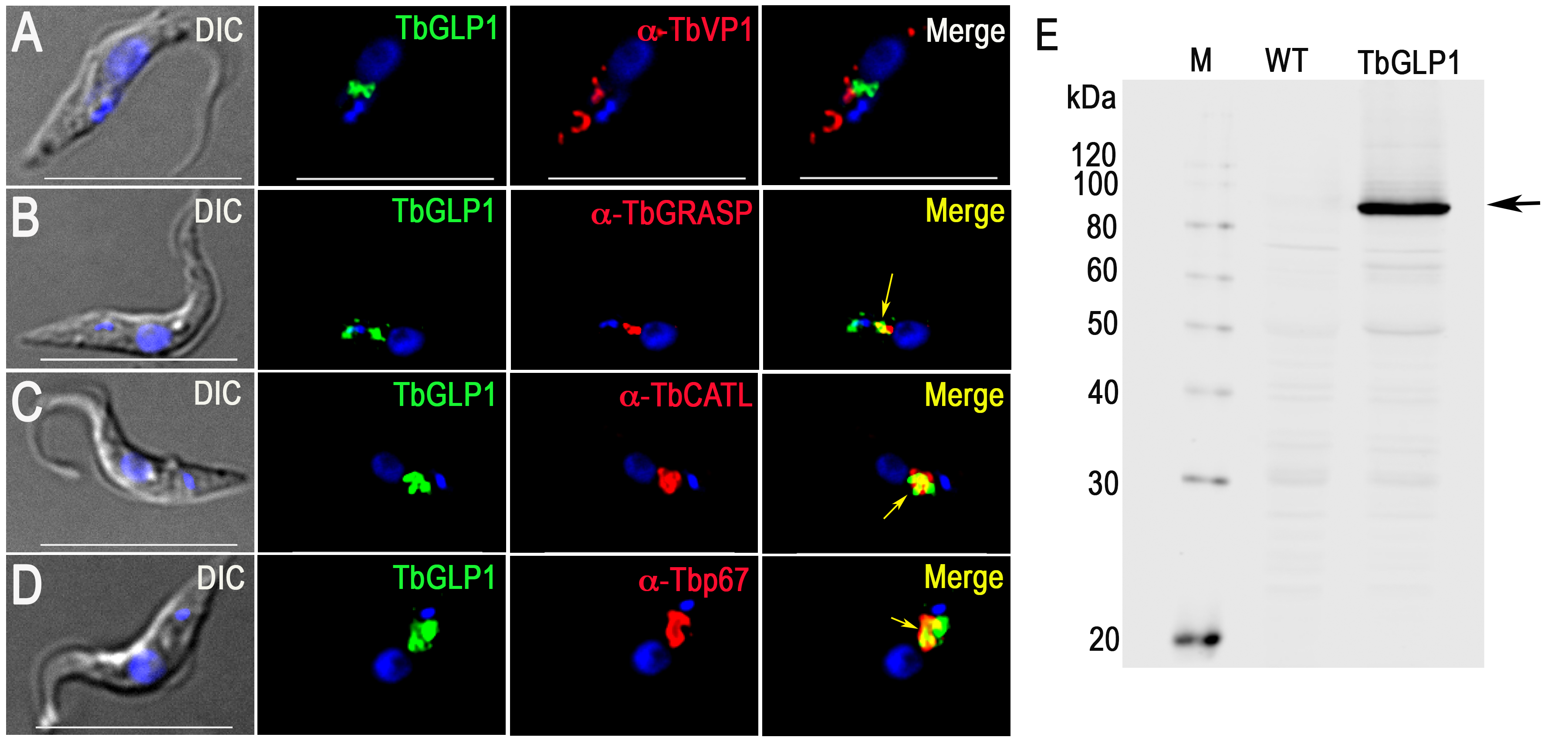

Supplement: S6 Figure — Localization of other proteins. Epitope-tagged TbGLP1 does not co-localize with TbVP1 (A) but co-localizes with TbGRASP to the Golgi complex (B), and with TbCATL (C) and p67 (D) to the lysosome (Pearson's correlation coefficients of 0.5369, 0.8050 and 0.8426, respectively). Yellow in merge images indicate co-localization (also shown with arrows in (B–D)). Scale bars for A–D = 10 µm. (E) Tagging with HA was confirmed by western blot analyses using anti-HA antibodies. Markers are at the left side and arrow shows the band corresponding to TbGLP1. Equivalent amounts of wild type cell (WT) proteins were loaded as evidenced by the similar background to the test lanes. (TIF) [file ppat.1004555.s006.tif]

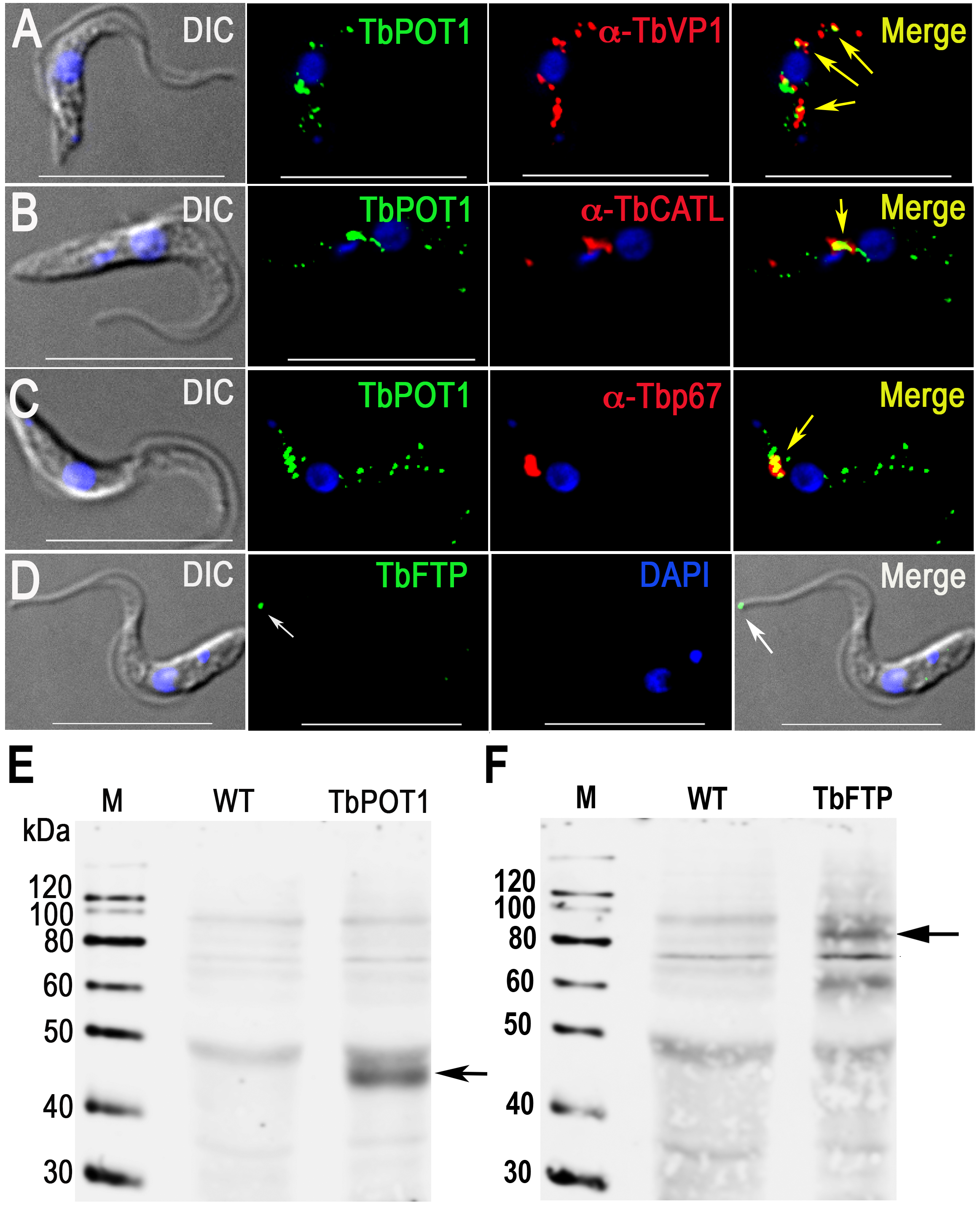

Supplement: S7 Figure — Immunofluorescence microscopy and western blot analysis of polyamine transporters TbPOT1. Epitope-tagged TbPOT1 partially co-localizes with TbVP1 to the acidocalcisomes (A) and co-localizes with TbCATL (B) and p67 (C), to the lysosomes (Pearson's correlation coefficients of 0.4064, 07191, and 0.6710, respectively). Arrrows in merge images show the co-localization. (D) A putative cation/proton antiporter localizes to the falgellar tip (white arrow) and was named flagellar tip protein (TbFTP). (E, F) Tagging with HA was confirmed by western blot analyses using anti-HA antibodies. Markers are at the left side and arrows shows the band corresponding to TgGLP1, and TbFTP, respectively. Equivalent amounts of wild type cell (WT) proteins were loaded as evidenced by the similar background to the test lanes. (TIF) [file ppat.1004555.s007.tif]

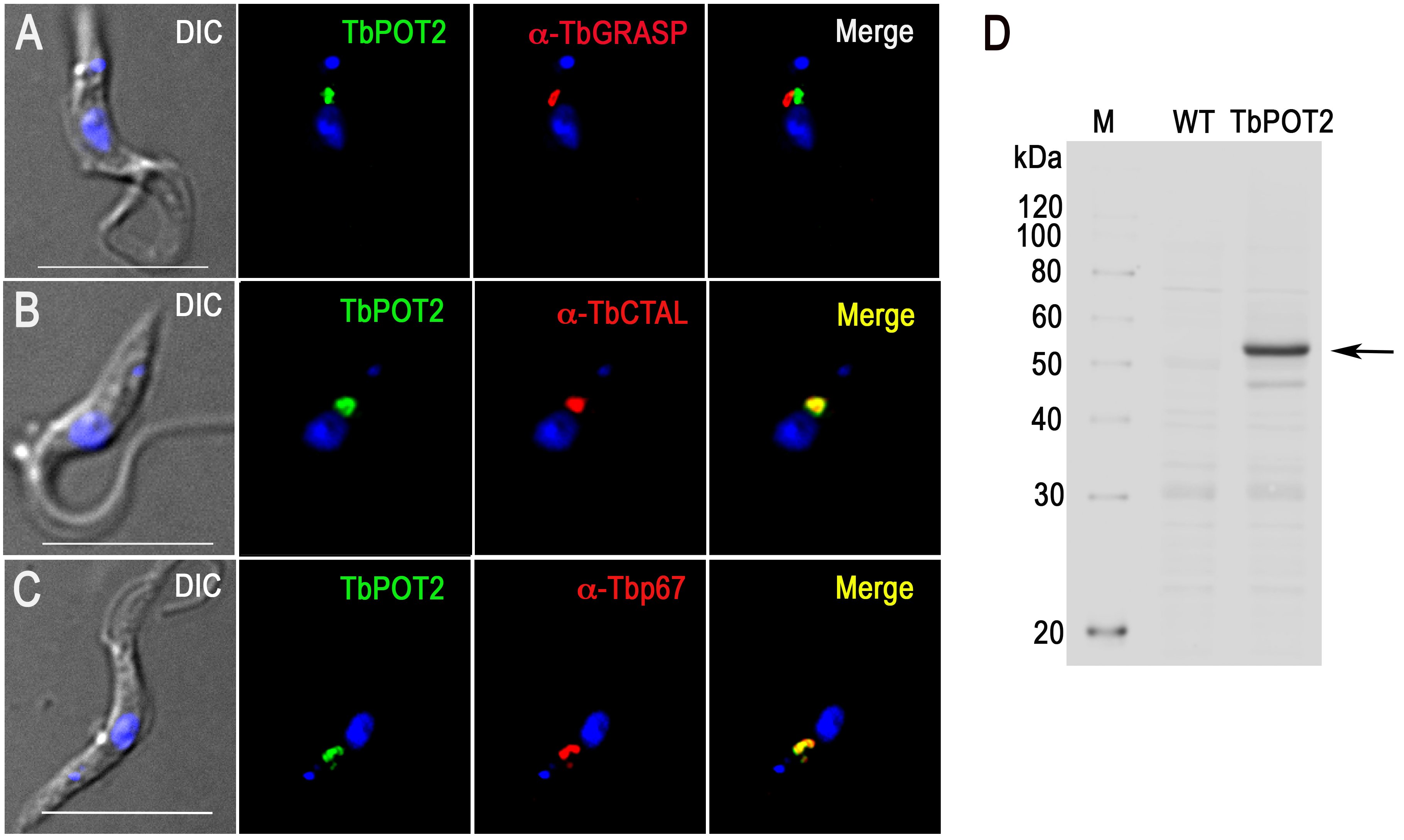

Supplement: S8 Figure — Immunofluorescence microscopy and western blot analysis of polyamine transporters TbPOT2. Epitope-tagged TbPOT2 does not co-localize with TbGRASP to the Golgi complex (A) but it co-localizes with TbCATL (B) and p67 to the lysosomes (C) (Pearson's correlation coefficients of 0.8806, 0.8404, respectively). Scale bars for A–C = 10 µm. (D) Tagging with HA was confirmed by western blot analyses using anti-HA antibodies. Markers are at the left side. Arrow indicate band corresponding to TbPOT2. Equivalent amounts of wild type cell (WT) proteins were loaded as evidenced by the similar background to the test lanes. (TIF) [file ppat.1004555.s008.tif]

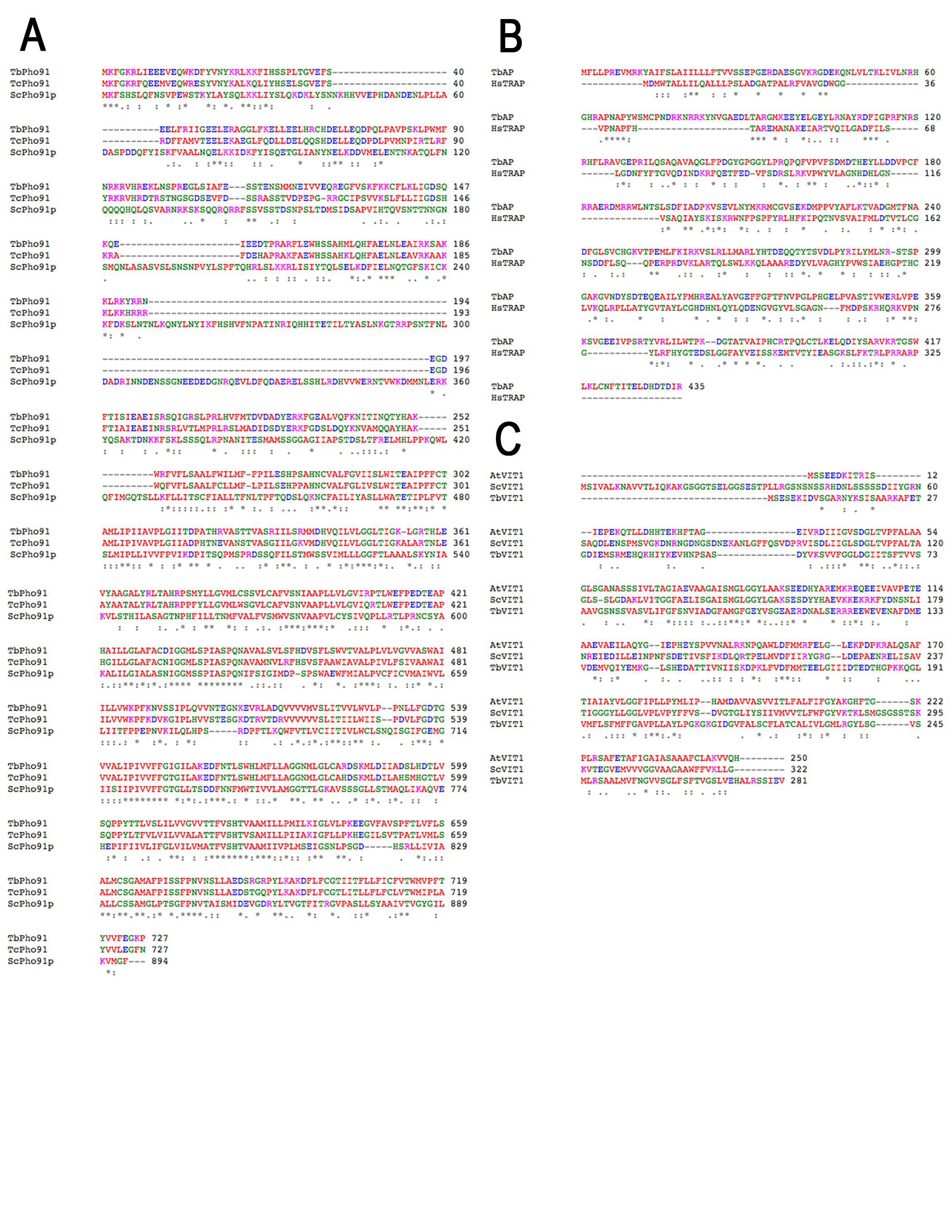

Supplement: S9 Figure — Comparison of three newly identified acidocalcisome proteins and their homologues with known functions from other organisms. Multiple protein sequence alignments of (A) phosphate transporters from S. cerevisiae (ScPho91p, accession number CAY82206), T. cruzi (TcPho91, TcCLB.508831.60), and T. brucei Pho91 (TbPho91, Tb927.11.11160). (B) Acid phosphatases from Homo sapiens (HsTRAP, P13686) and T. brucei (TbAP, Tb927.10.7020). (C) Vacuolar iron transporters from A. thaliana (AtVIT1, NP_178286), S. cerevisiae (ScVIT1, DDA09536), and T. brucei (TbVIT1, Tb927.3.800). The protein sequences were analyzed via ClustalW2 at the EMBL-EBI website (http://www.ebi.ac.uk/Tools/msa/clustalw2/). The symbols “*”, “:”, and “.” represent identical, conserved, or semi-conserved amino acid (aa) substitutions, respectively. Red: small and hydrophobic aa (AVFPMILW); blue: acidic aa (DE); magenta: basic aa (RK); and green: hydroxyl, amine, and basic aa (STYHCNGQ). (TIF) [file ppat.1004555.s009.tif]

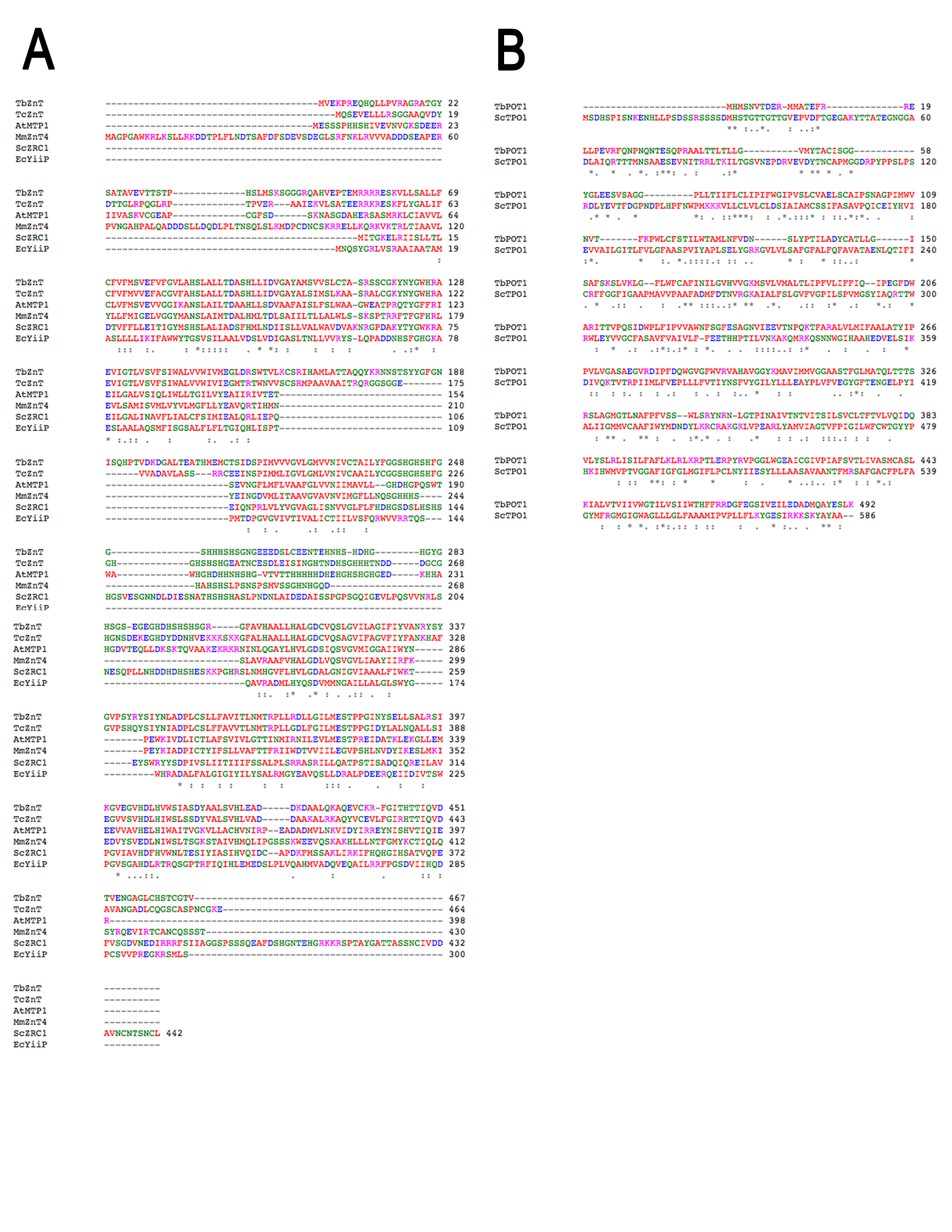

Supplement: S10 Figure — Comparison of two newly identified acidocalcisome proteins and their homologues with known functions from other organisms. Multiple protein sequence alignments of (A) Zinc transporters from Mus musculus (MmZnT4, AAB82593), S. cerevisiae (ScZRC1, CAA88653.1), A. thaliana (AtMTP1, NP_850459), E. coli (EcYiiP, P69380.1), T. cruzi (TcZnT, TcCLB.511439.50), and T. brucei (TbZnT, Tb927.4.4960). (B) Polyamine transporters from S. cerevisiae (ScTPO1, Q07824) and T. brucei (TbPOT1, Tb927.9.10340). Analysis was done as in Fig. S9. (TIF) [file ppat.1004555.s010.tif]
