# Supplementary material for: Proteomic Analysis of the Acidocalcisome, an Organelle Conserved from Bacteria to Human Cells
Source: PLoS Pathog. 2014 Dec 11;10(12):e1004555. doi: 10.1371/journal.ppat.1004555 (PMC4263762; doi:10.1371/journal.ppat.1004555)
Supplement: S1 Table — T. brucei proteins identified with high confidence (1% false discovery rate, protein probability ≥0.95) from fraction 5 datasets (ACCS1 and ACCS2). Proteins with degenerate peptide (peptides shared among all members of the protein cluster) fingerprints are reported in a single protein “group” as described in the Methods. (PDF) [file ppat.1004555.s011.pdf]

Table S1. *T. brucei proteins identified with high confidence (1% false discovery rate, protein probability ≥ 0.95) from fraction 5 datasets (ACCS1 and ACCS2). Proteins with degenerate peptide (peptides shared among all members of the protein cluster) fingerprints are reported in a single protein "group" as described in the Methods.*

					ACCS1						ACCS2					
Protein Group	Description	Length	Length	Mass	Probability	Total Score	Total Peptides	Peptides (Paris Guidelines)	Spectra	% coverage	Probability	Total Score	Total Peptides	Peptides (Paris Guidelines)	Spectra	% coverage
1	Tb927.6.3740	heat shock 70 kDa protein, mitochondrial precursor, putative	657	71.4	1	4164	83	52	393	74	1	3981	79	41	347	67
	Tb927.6.3750	heat shock 70 kDa protein, mitochondrial precursor, putative	657	71.4												
	Tb927.6.3800	heat shock 70 kDa protein, mitochondrial precursor, putative	657	71.4												
2	Tb927.11.6280	pyruvate phosphate dikinase	913	100.4	1	3628	80	54	336	61	1	3407	76	49	274	67
3	Tb927.1.2330	beta tubulin	442	49.7	1	3320	72	32	484	71	1	2987	67	30	344	71
	Tb927.1.2350	beta tubulin	442	49.7												
	Tb927.1.2370	beta tubulin	442	49.7												
	Tb927.1.2390	beta tubulin	442	49.7												
4	Tb927.10.6400	chaperonin HSP60, mitochondrial precursor	562	59.4	1	3190	65	44	338	73	1	2754	58	37	183	68
	Tb927.10.6510	chaperonin HSP60, mitochondrial precursor	562	59.4												
5	Tb927.3.1380	ATP synthase beta chain, mitochondrial precursor,ATP synthase F1, beta subunit	519	55.7	1	2841	52	31	191	61	1	2923	52	30	272	61
6	Tb927.2.4210	glycosomal phosphoenolpyruvate carboxykinase	525	58.5	1	2459	54	38	328	65	1	2769	59	39	245	74
7	Tb927.11.7460	glucose-regulated protein 78, putative,luminal binding protein 1 (BiP), putative	653	71.4	1	2234	46	37	147	59	1	1768	36	29	143	58
	Tb927.11.7510	glucose-regulated protein 78, putative,luminal binding protein 1 (BiP), putative	653	71.4												
8	Tb927.10.5620	fructose-bisphosphate aldolase, glycosomal	372	41.0	1	2914	55	39	222	72	1	1548	32	21	94	51
9	Tb927.1.2340	alpha tubulin	451	49.7	1	2567	52	32	578	65	1	2475	49	29	518	64
	Tb927.1.2360	alpha tubulin	451	49.7												
	Tb927.1.2380	alpha tubulin	451	49.7												
	Tb927.1.2400	alpha tubulin	451	49.7												
10	Tb927.6.4280	glyceraldehyde 3-phosphate dehydrogenase, glycosomal	359	39.0	1	2540	52	35	405	88	1	2259	48	27	402	81
	Tb927.6.4300	glyceraldehyde 3-phosphate dehydrogenase, glycosomal	359	39.0												
11	Tb927.8.3530	glycerol-3-phosphate dehydrogenase [NAD], glycosomal	354	37.8	1	2050	39	31	283	87	1	2514	46	25	300	84
12	Tb927.11.11330	heat shock protein 70	690	75.3	1	1306	28	21	63	40	1	1083	25	21	55	49
13	Tb927.11.9980	2-oxoglutarate dehydrogenase E1 component, putative	1005	112.9	1	1560	41	33	116	44	1	1144	26	24	67	35
14	Tb927.10.15410	glycosomal malate dehydrogenase	323	33.7	1	1708	35	24	163	68	1	1979	38	23	315	78
15	Tb927.9.12570	glycerol kinase, glycosomal	512	56.3	1	1499	30	24	102	55	1	1142	22	17	81	49
16	Tb927.5.930	NADH-dependent fumarate reductase	1142	123.6	1	1269	33	28	125	34	1	1367	31	27	78	33
17	Tb927.11.3980	mitochondrial processing peptidase alpha subunit, putative,metallo-peptidase, Clan ME, Family M16	518	57.0	1	1416	30	20	90	52	1	922	21	16	66	49
18	Tb927.2.4230	NUP-1 protein, putative	3647	406.6	1	1426	31	18	131	6	1	1210	22	13	78	4
19	Tb927.3.2230	succinyl-CoA synthetase alpha subunit, putative	301	31.4	1	723	21	12	68	42	1	922	18	8	52	39
20	Tb927.5.1060	mitochondrial processing peptidase, beta subunit, putative,metallo-peptidase, Clan ME, Family M16	489	54.1	1	1466	26	24	156	53	1	818	17	15	66	37
21	Tb927.11.5520	triosephosphate isomerase	250	26.8	1	1196	26	13	128	47	1	1516	28	14	134	51
22	Tb927.4.2070	antigenic protein, putative	4455	511.0	1	1118	25	13	66	5	1	221	6	4	14	1
23	Tb927.11.11680	2-oxoglutarate dehydrogenase E2 component, putative	383	41.1	1	1416	29	21	89	43	1	633	17	13	38	32
24	Tb927.10.8030	hypothetical protein, conserved	255	28.8	1	992	20	15	85	58	1	1159	23	14	89	62
25	Tb927.10.7410	succinyl-CoA ligase [GDP-forming] beta-chain, putative-with=GeneDB:LmjF36.2950	419	44.9	1	895	19	15	66	42	1	886	19	16	58	54
26	Tb927.9.5900	glutamate dehydrogenase	992	112.0	1	776	20	17	68	28	1	1263	26	19	67	31
27	Tb927.5.2930	hypothetical protein, conserved	370	43.3	1	1069	22	17	101	47	1	830	19	15	62	40
28	Tb927.5.2080	guanosine monophosphate reductase, putative	491	52.2	1	813	18	16	57	42	1	622	13	12	28	36
29	Tb927.9.5320	nucleolar RNA binding protein, putative	501	55.0	1	953	19	13	47	29	1	704	12	9	26	27
30	Tb927.8.2770	inositol 1,4,5-trisphosphate receptor	3099	342.2	1	636	18	15	71	8	1	1308	26	22	97	13
31	Tb927.10.3210	delta-1-pyrroline-5-carboxylate dehydrogenase, putative	561	62.0	1	827	19	14	69	31	1	650	16	11	33	25
32	Tb927.11.1020	ribokinase, putative	333	35.2	1	1213	20	10	62	39	1	1270	21	10	94	37
33	Tb927.11.2690	succinyl-coA:3-ketoacid-coenzyme A transferase, mitochondrial precursor, putative	493	53.0	1	583	9	4	20	14	1	766	12	6	21	17
34	Tb927.11.1450	2-oxoglutarate dehydrogenase E1 component, putative	1008	113.3	1	813	23	21	75	31	1	1034	24	22	59	34
35	Tb927.10.14820	mitochondrial carrier protein, putative,ADP/ATP translocase 1, putative	307	34.0	1	990	21	15	216	46	0.99	737	13	9	148	36
	Tb927.10.14830	mitochondrial carrier protein, putative,ADP/ATP translocase 1, putative	307	34.0												
	Tb927.10.14840	mitochondrial carrier protein, putative,ADP/ATP translocase 1, putative	307	34.0												
36	Tb927.10.14550	ATP-dependent DEAD/H RNA helicase, putative	660	71.3	1	836	16	10	50	20	1	687	13	10	52	24
37	Tb927.10.2090	elongation factor 1-alpha,EF-1-alpha	348	37.8	1	1018	22	12	179	43	0.97	591	15	10	54	43
	Tb927.10.2100	elongation factor 1-alpha,EF-1-alpha	449	49.1												
	Tb927.10.2110	elongation factor 1-alpha	449	49.1												
38	Tb927.7.7420	ATP synthase alpha chain, mitochondrial precursor,ATP synthase F1, alpha subunit	584	63.4	1	1032	25	16	79	23	0.84	247	10	8	24	16
	Tb927.7.7430	ATP synthase alpha chain, mitochondrial precursor,ATP synthase F1, alpha subunit	584	63.4												
39	Tb927.10.2020	hexokinase	471	51.1	1	534	12	11	27	35	1	618	15	13	38	42
40	Tb927.10.1510	NOT1	2326	258.9	1	661	18	15	42	11	1	601	13	12	30	10
41	Tb927.2.1560	cyclophilin-type peptidyl-prolyl cis-trans isomerase, putative	196	21.1	0.98	742	10	6	20	67						
42	Tb927.3.3580	lipophosphoglycan biosynthetic protein, putative	773	87.7	1	680	14	12	58	22	1	798	19	15	52	22
43	Tb927.3.1790	pyruvate dehydrogenase E1 beta subunit, putative	348	37.5	1	881	17	16	70	41	1	567	13	11	35	46
44	Tb927.10.7570	dihydrolipoamide acetyltransferase E2 subunit, putative	451	48.1	1	560	15	13	39	43	1	549	11	10	27	38
45	Tb927.5.3400	calcium-translocating P-type ATPase,calcium pump	1011	110.2	1	813	21	16	47	21	1	353	9	9	15	11
46	Tb927.8.3750	nucleolar protein, putative	483	54.3	1	806	17	13	45	42	1	435	10	9	27	39
47	Tb927.1.4100	cytochrome oxidase subunit IV	353	40.5	1	656	19	11	45	42	1	729	18	9	64	40
48	Tb927.8.2540	3-ketoacyl-CoA thiolase, putative	438	46.2	1	671	11	10	38	33	1	341	7	5	26	22
49	Tb927.8.5640	hypothetical protein, conserved	333	37.0	1	726	15	12	42	50	1	890	15	10	43	48
50	Tb927.10.1170	intraflagellar transport protein IFT172, putative	1747	196.5	1	540	15	14	41	13	1	600	12	12	28	10
51	Tb927.10.12700	pyruvate dehydrogenase E1 alpha subunit, putative	378	42.5	1	791	18	13	50	45	1	557	11	7	35	27
52	Tb927.10.16120	inosine-5'-monophosphate dehydrogenase,IMP dehydrogenase	447	48.4	1	661	14	11	41	42	1	339	9	7	25	28
53	Tb927.9.11580	Gim5A protein,glycosomal membrane protein	243	26.4	1	780	15	7	185	37	1	515	9	6	60	37
	Tb927.9.11600	Gim5B protein,glycosomal membrane protein	241	25.9												
54	Tb927.10.2010	hexokinase	471	51.2	0.99	407	10	9	21	31	0.98	410	10	10	25	34
55	Tb927.8.1740	hypothetical protein, conserved	587	62.1	1	350	9	8	26	18	1	391	8	5	15	16
56	Tb927.8.6110	hypothetical protein, conserved	499	54.6	1	414	10	10	31	41	1	237	7	7	19	21
57	Tb927.10.2560	mitochondrial malate dehydrogenase	318	33.1	1	521	12	10	37	44	1	737	16	10	69	51
58	Tb927.10.4560	elongation factor 2	846	94.3	1	506	14	12	58	20	0.99	444	10	9	20	17
	Tb927.10.4570	elongation factor 2	846	94.3												

59	Tb927.6.2230	hypothetical protein, conserved	775	86.6		1	315	8	7	18	13		1	122	3	3	5	10
60	Tb927.10.8940	hypothetical protein, conserved	411	45.4		1	277	9	9	24	40		1	152	5	4	11	14
61	Tb927.10.7020	acid phosphatase, putative	435	49.9		1	370	8	8	31	29		1	418	11	9	25	33
62	Tb927.6.4440	hypothetical protein, conserved	352	37.7		1	809	15	12	61	35		1	234	6	6	18	23
63	Tb927.8.6060	2-amino-3-ketobutyrate coenzyme A ligase, putative,glycine acetyltransferase, putative	404	43.7		1	504	11	10	31	32		1	462	10	7	42	26
64	Tb927.6.2790	L-threonine 3-dehydrogenase, putative	332	36.9		1	614	11	10	47	32		1	714	12	10	106	38
65	Tb927.4.1300	hypothetical protein, conserved	374	42.0		1	512	14	12	38	49		1	329	9	8	42	30
66	Tb927.10.4310	prohibitin 2, putative	295	32.2		1	493	11	9	24	38		1	495	10	6	20	26
67	Tb927.9.14160	rieske iron-sulfur protein, mitochondrial precursor	297	33.6		1	464	12	9	47	28		1	665	17	10	45	33
68	Tb927.1.120	retrotransposon hot spot protein 4 (RHS4), putative	856	97.9		1	406	13	12	23	15		1	332	8	8	16	10
69	Tb927.10.2350	pyruvate dehydrogenase complex E3 binding protein, putative	260	27.3		1	663	14	11	45	42		1	429	10	8	23	33
70	Tb927.10.180	ATP synthase F1 subunit gamma protein, putative	305	34.3		1	260	8	8	24	32		1	155	3	2	6	19
71	Tb927.3.3900	carnitine O-palmitoyltransferase II, putative	621	67.3		1	508	11	8	21	18		1	505	11	10	25	24
72	Tb927.2.3030	ATP-dependent Clp protease subunit, heat shock protein 78 (HSP78), putative,serine peptidase, putative	812	90.6		1	505	15	14	26	27		1	360	9	9	23	21
73	Tb927.3.3270	ATP-dependent phosphofructokinase	487	53.5		1	523	12	12	23	24		1	319	8	7	23	21
74	Tb927.10.2240	hypothetical protein, conserved	588	66.3		1	464	10	8	22	19		1	233	5	5	9	12
75	Tb927.11.16730	dihydrolipoyl dehydrogenase	477	50.4		1	498	13	11	35	28		1	404	9	7	27	22
76	Tb927.9.12510	ATP-dependent DEAD/H RNA helicase, putative	735	82.7		1	418	11	10	66	19		1	459	10	8	69	16
77	Tb927.3.5050	60S ribosomal protein L4	374	41.8		1	429	13	11	40	40		1	62	3	3	4	11
78	Tb927.4.4380	vacuolar-type proton translocating pyrophosphatase 1, putative	826	85.9		0.96	270	6	5	30	9		0.99	560	11	8	94	13
	Tb927.8.7980	vacuolar-type proton translocating pyrophosphatase 1,V-type H( )-translocating pyrophosphatase	826	85.9														
79	Tb927.9.5150	ribosomal protein S6, putative,NHP2/RS6-like protein	126	13.5		1	556	12	7	26	80							
80	Tb927.4.3950	cytoskeleton-associated protein CAP5.5, putative,cysteine peptidase, Clan CA, family C2, putative	853	94.6		1	357	10	9	22	18		1	322	7	5	16	12
81	Tb927.3.3130	hypothetical protein, conserved	1642	178.0		1	438	13	10	27	9		1	209	6	3	7	4
82	Tb927.9.6100	TFIIF-stimulated CTD phosphatase, putative	343	39.5		1	354	9	8	26	20		1	157	4	4	13	17
83	Tb927.8.6170	transketolase, putative	669	72.5		1	387	11	10	29	25		1	433	14	13	44	32
84	Tb927.3.930	dynein heavy chain, putative	4639	531.1		1	305	10	9	27	3		1	136	6	6	6	2
85	Tb927.10.14750	fibrillarin, putative	304	31.6		1	468	12	11	32	46		0.74	107	3	3	3	13
86	Tb927.9.6090	TFIIF-stimulated CTD phosphatase, putative	343	39.5		1	315	9	8	22	19		0.87	112	4	4	10	16
87	Tb927.11.2650	heat shock protein 84, putative	753	84.1		1	388	8	6	24	14		1	321	8	7	14	18
88	Tb927.11.13180	hypothetical protein, conserved	560	61.2		1	286	5	4	19	16		1	366	8	7	20	24
89	Tb927.9.10770	Polyadenylate-binding protein 2 (Poly(A)-binding protein 2) (Poly(A)-binding protein II) (PABII) (Polyadenylate-binding nuclear protein 1) (Nuclear poly(A)-binding protein 1) (PABP2), putative	555	62.1		1	353	9	8	38	22		1	303	7	6	24	19
90	Tb927.8.6580	succinate dehydrogenase flavoprotein, putative	609	66.8		1	444	8	6	28	16		1	365	7	7	12	16
91	Tb927.11.7380	glycerol-3-phosphate dehydrogenase (FAD-dependent), mitochondrial	603	66.9		1	338	7	7	27	11		1	278	6	6	14	17
92	Tb927.9.2470	nucleolar protein	757	85.8		1	345	12	11	24	18		1	277	6	6	18	15
93	Tb927.8.1160	vacuolar-type Ca2 -ATPase, putative	1102	121.1		0.65	104	3	3	7	5		0.99	572	9	9	40	15
94	Tb927.7.2700	NADH-cytochrome b5 reductase, putative	287	31.8		1	366	9	9	29	38		1	176	4	3	5	12
95	Tb927.10.470	choline dehydrogenase, putative	525	56.9		1	335	8	7	22	25		1	158	4	4	16	18
96	Tb927.8.1180	vacuolar-type Ca2 -ATPase 1	1106	121.5		0.6	89	2	2	6	4		0.96	534	8	8	38	14
97	Tb927.11.13280	mitochondrial RNA binding protein 2	224	25.1		1	221	6	6	18	36		1	311	8	5	13	29
98	Tb927.5.1210	short-chain dehydrogenase, putative	311	33.8		1	387	9	6	29	23		1	232	5	4	11	16
99	Tb927.6.2170	co-chaperone GrpE, putative	222	23.8		1	343	9	9	31	58		1	149	4	4	10	41
100	Tb927.7.6360	histone H2A variant	179	18.6		1	459	10	5	43	40							
101	Tb927.10.12500	P-type H -ATPase, putative	912	100.1		1	230	7	5	25	14		1	200	5	4	10	10
102	Tb927.5.3810	orotidine-5-phosphate decarboxylase/orotate phosphoribosyltransferase, putative,OMPDCase-OPRTase, putative	458	49.9		1	414	8	8	29	25		1	347	7	7	21	21
103	Tb927.10.13510	zinc metallopeptidase, putative	569	62.7		1	329	7	7	20	11		1	231	6	6	18	23
104	Tb927.5.520	stomatin-like protein, putative	531	55.9		1	499	10	7	25	20		1	219	5	4	11	12
105	Tb927.11.3250	dynein heavy chain, putative	4658	530.8		1	412	11	9	29	4		1	94	3	3	5	1
106	Tb927.10.11310	intraflagellar transport protein IFT55/IFT57, putative	413	46.3		0.95	39	1	1	1	5		1	210	6	5	8	27
107	Tb927.8.3060	cytosolic leucyl aminopeptidase, putative,metallo-peptidase, Clan MF, Family M17	671	71.3		1	401	9	7	38	18		1	425	7	6	33	19
108	Tb927.8.1330	60S ribosomal protein L7a, putative	276	30.8		1	570	11	11	38	42		0.93	237	5	5	7	30
	Tb927.8.1340	60S ribosomal protein L7a, putative	276	30.8														
109	Tb927.10.6910	Sterol methyltransferase, putative	359	40.1		0.97	355	8	6	29	29		0.96	342	7	4	24	24
	Tb927.10.6950	sterol 24-c-methyltransferase, putative	359	40.1														
110	Tb927.9.9660	hypothetical protein, conserved	351	38.3		1	309	7	5	46	20		1	300	7	4	32	16
111	Tb927.2.2510	voltage-dependent anion-selective channel	270	29.2		1	473	10	9	36	37		0.98	343	7	5	23	25
	Tb927.2.2520	voltage-dependent anion-selective channel	270	29.2														
112	Tb927.11.6460	hypothetical protein, conserved	954	107.3		1	241	6	5	14	11		1	305	7	6	8	14
113	Tb927.7.6670	hypothetical protein, conserved	3030	332.7		1	443	11	11	29	7		1	87	3	3	3	2
114	Tb927.3.4630	UDP-glucose:glycoprotein glucosyltransferase, putative	1675	186.7		1	430	9	7	21	8		1	159	3	3	6	4
115	Tb927.8.1510	ATP-dependent DEAD/H RNA helicase, putative	569	62.4		0.98	40	1	1	4	7		1	203	4	4	10	9
116	Tb927.11.11520	glycosomal membrane protein, putative	218	24.0		1	557	12	11	45	29		0.91	44	1	1	3	6
117	Tb927.3.2600	ATP-dependent DEAD/H RNA helicase, putative	2170	244.8		1	323	7	7	18	6		1	234	5	4	8	4
118	Tb927.8.5200	hypothetical protein, conserved	1788	201.4		1	198	6	6	11	5		1	142	3	3	6	4
119	Tb927.9.5730	nucleosome assembly protein-like protein	422	47.5		1	280	6	5	17	19		1	138	4	4	10	16
120	Tb927.9.12730	chaperone protein DNAj, putative	451	48.5		1	433	10	7	29	27		1	165	5	4	14	14
121	Tb927.8.3380	electron transfer protein, putative	242	27.2		1	229	6	5	14	29		1	414	7	5	14	32
122	Tb927.11.11010	hypothetical protein, conserved	348	41.6		1	435	13	12	32	38		1	56	3	3	4	13
123	Tb927.4.1500	RNA editing associated helicase 2	2167	241.7		1	171	6	6	9	4							
124	Tb927.3.3310	60S ribosomal protein L13, putative	218	25.4		0.99	455	9	9	37	31		0.41	32	1	1	4	7
	Tb927.3.3320	60S ribosomal protein L13, putative	218	25.4														
125	Tb927.11.5450	malic enzyme, putative	571	63.1		1	186	5	4	12	13		1	287	5	5	20	18
126	Tb927.2.450	retrotransposon hot spot protein 4 (RHS4), putative	857	97.9		0	109	4	4	6	5		0.96	189	4	4	8	6
127	Tb927.10.13800	hypothetical protein, conserved	323	34.4		1	256	4	4	10	26		1	83	5	5	7	22
128	Tb927.10.14600	40S ribosomal protein S2, putative	266	28.6		0.97	229	6	6	17	25							
129	Tb927.9.3170	cytochrome oxidase subunit V	196	22.2		1	420	10	9	36	48		0	4	1	1	1	6
130	Tb927.10.520	hypothetical protein, conserved	396	46.7		1	494	10	7	49	21		1	209	4	2	14	9
131	Tb927.11.1090	calpain-like protein, putative,cytoskeleton assocociated protein, putative	5795	657.8		1	248	8	7	31	2		0.39	53	2	2	5	0
132	Tb927.8.7530	3,2-trans-enoyl-CoA isomerase, mitochondrial precursor, putative	358	39.7		1	150	4	4	7	17		1	344	7	6	13	18
133	Tb927.10.450	hypothetical protein, conserved	585	65.6		1	104	4	4	9	14		0.99	41	1	1	2	5
134	Tb927.11.13090	elongation factor 1 gamma, putative	404	46.3		0.99	342	9	7	29	21		0.75	111	2	2	7	9
	Tb927.11.13190	elongation factor 1 gamma, putative	532	60.4														

135	Tb927.7.1730	60S ribosomal protein L7, putative	242	27.7		1	433	8	8	29	39		0.86	244	7	6	17	30
136	Tb927.6.1520	aquaporin 3, putative	321	35.0		1	173	3	1	7	10		1	243	5	1	6	10
137	Tb927.11.6250	hypothetical protein, conserved	269	27.6		1	190	5	4	20	23		1	237	3	2	9	16
138	Tb927.11.3590	40S ribosomal protein S4, putative	273	30.6		0.97	268	8	7	25	33		0.75	115	2	2	6	11
	Tb927.11.3600	40S ribosomal protein S4, putative	273	30.6														
139	Tb927.7.3550	hypothetical protein, conserved	1241	138.1		1	330	9	8	26	9		0.27	33	2	2	2	3
140	Tb927.3.4760	dynamin, putative,vacuolar sortin protein 1, putative	660	73.1		1	346	7	7	17	15		1	210	5	4	8	8
141	Tb927.9.10310	mitochondrial carrier protein, putative,mitochondrial phosphate transporter, putative	317	34.3		1	219	4	4	22	23		1	212	6	3	10	18
142	Tb927.9.10400	hypothetical protein, conserved	495	53.6		1	315	6	6	21	10		1	111	2	2	7	5
143	Tb927.10.14500	hypothetical protein, conserved	337	37.0		1	324	7	7	25	23		0	20	2	2	2	12
144	Tb927.7.1300	protein disulfide isomerase, putative	377	41.9		1	251	5	5	19	18		1	90	2	2	6	6
145	Tb927.10.3940	40S ribosomal protein S3A, putative	256	29.4		1	371	9	9	27	33							
146	Tb927.2.4710	RNA-binding protein, putative	441	49.9		1	257	6	5	23	17		1	258	4	4	31	12
147	Tb927.11.2060	60S acidic ribosomal subunit protein, putative	324	34.6		0.98	286	8	7	25	35		0.85	134	4	3	5	14
	Tb927.11.2050	60S acidic ribosomal subunit protein, putative	324	34.6														
148	Tb927.4.2450	thioredoxin, putative	411	44.4		1	381	7	5	16	15		1	64	1	1	3	5
149	Tb927.4.4210	ATP-dependent zinc metallopeptidase, putative,metallo-peptidase, Clan MA(E) Family M41	877	96.1		1	292	7	7	17	11		0.92	48	2	2	3	5
150	Tb927.10.7500	fibrillarin	300	31.6		1	238	5	4	12	21		1	160	3	2	6	13
151	Tb927.11.3490	hypothetical protein, conserved	798	87.7		1	287	6	6	17	9		1	141	2	2	6	4
152	Tb927.11.2670	Nucleoporin	545	58.8		1	271	5	5	13	12		1	125	3	2	3	6
153	Tb927.10.3650	NADH-dependent fumarate reductase, putative	1232	133.4		0.89	242	6	5	11	5		1	200	5	5	8	5
154	Tb927.5.1300	vacuolar proton translocating ATPase subunit A, putative	783	89.6		0.48	31	1	1	2	3		1	361	7	5	18	12
155	Tb927.3.4970	hypothetical protein, conserved	1063	115.7		0.98	102	5	5	6	11		0.91	40	2	2	4	6
156	Tb927.8.4810	prohibitin 1	277	31.4		1	245	5	5	23	17		1	119	3	3	4	16
157	Tb927.10.3990	DHH1	406	46.4		1	163	5	4	16	16		1	237	6	4	14	21
158	Tb927.11.8260	carbonic anhydrase-like protein	422	45.7		1	64	1	1	4	3		1	197	4	3	12	10
159	Tb927.8.900	splicing factor TSR1	328	37.4		1	301	7	7	35	19		1	127	3	3	7	12
160	Tb927.9.2900	hypothetical protein, conserved	2631	287.0		1	212	6	5	13	3		0	14	1	1	1	1
161	Tb927.9.4210	fatty acyl CoA synthetase 3	702	77.8		1	259	8	8	22	13		1	214	6	5	13	12
162	Tb927.3.1120	GTP-binding nuclear protein rtb2, putative	217	24.4		1	211	4	3	18	32		1	57	1	1	2	9
163	Tb927.5.3230	hypothetical protein, conserved	2181	240.8		1	218	4	4	20	4		0	12	1	1	1	1
164	Tb927.11.1270	hypothetical protein, conserved	169	20.2		1	240	4	3	10	17							
165	Tb927.2.3780	translation initiation factor IF-2, putative	833	94.4		1	104	5	5	10	9		1	268	7	6	21	13
166	Tb927.11.13500	par1	592	68.3		1	239	6	6	18	16		1	97	3	2	8	8
167	Tb927.11.4480	radial spoke protein RSP4/6, putative	585	66.8		1	280	6	6	14	20		0	4	1	1	1	3
168	Tb927.5.1470	NADH-cytochrome b5 reductase, putative	294	32.7		1	210	6	6	10	32		1	124	4	4	8	22
169	Tb927.8.1890	cytochrome c1, heme protein, mitochondrial precursor	258	30.0		1	185	4	3	6	29		1	167	3	1	7	8
170	Tb927.11.2410	hypothetical protein, conserved	222	24.5		1	221	5	4	14	23		1	150	5	5	8	28
171	Tb927.11.14730	metalloprotease, putative,cell division protein FtsH homologue, putative	677	74.4		1	333	8	7	22	13		1	201	5	4	16	9
172	Tb927.6.4090	chaperonin HSP60, mitochondrial precursor, putative	541	58.3		1	295	5	3	17	8		1	285	5	4	13	10
173	Tb927.3.3490	high mobility group protein, putative	271	30.8		1	150	4	4	9	16		1	49	1	1	3	5
174	Tb927.1.4310	hypothetical protein, conserved	1638	183.6		1	334	7	6	18	7		1	116	2	1	2	2
175	Tb927.7.1790	Adenine phosphoribosyltransferase, putative	230	25.6		1	257	5	5	22	37		1	142	2	2	8	15
176	Tb927.11.11360	guanine nucleotide-binding protein beta subunit- like protein,activated protein kinase c receptor	318	34.7		0.98	300	6	6	17	25		0.96	271	6	4	14	16
	Tb927.11.11370	guanine nucleotide-binding protein beta subunit-like protein,activated protein kinase c receptor	318	34.7														
177	Tb927.9.10560	hypothetical protein, conserved	168	19.0		1	218	3	2	11	14							
178	Tb927.3.3670	RNA-binding protein, putative	623	66.9		1	261	7	7	22	13							
179	Tb927.11.12150	flagellar protein essential for flagellar pocket biogenesis	587	67.3		1	172	4	4	5	10		0.99	69	2	2	3	7
180	Tb927.10.3260	Long-chain-fatty-acid--CoA ligase 5 (EC 6.2.1.3) (Long-chain acyl-CoA synthetase 5) (LACS 5), putative	703	78.9		1	149	4	4	11	10		1	119	3	3	7	8
181	Tb927.3.2180	hypothetical protein, conserved	156	17.9		1	133	3	2	3	35							
182	Tb927.10.560	40S ribosomal proteins S11, putative	174	20.1		1	235	5	5	17	22		0.49	25	1	1	2	7
183	Tb927.7.4770	cyclophilin-type peptidyl-prolyl cis-trans isomerase, putative	173	18.6		1	345	6	4	15	36							
184	Tb927.11.550	hypothetical protein SCD6.10	280	31.3		0	18	1	1	1	10		1	56	1	1	1	6
185	Tb927.8.890	small GTP-binding protein Rab1, putative	208	22.7		1	182	4	1	6	15							
186	Tb927.7.2570	mRNA processing protein, putative	473	52.5		1	200	3	3	17	10		1	216	4	4	15	10
187	Tb927.10.15180	nucleosome assembly protein, putative	366	41.3		1	61	1	1	4	3		0	24	2	2	2	5
188	Tb927.11.1430	hypothetical protein, conserved	752	86.8		1	153	5	5	9	13		1	188	4	4	14	11
189	Tb927.9.9550	hypothetical protein, conserved	177	20.7		1	221	6	6	19	38		1	104	2	2	3	15
190	Tb927.4.5010	calreticulin, putative	395	45.0		0.97	265	5	4	23	11		0.91	211	4	4	16	14
	Tb927.8.7410	calreticulin, putative	395	45.0														
191	Tb927.7.3740	hypothetical protein, conserved	849	93.2		0.99	71	2	2	5	4		0	11	1	1	1	2
192	Tb927.10.9570	paraflagellar rod component, putative	1082	118.6		1	248	7	6	11	9		1	171	5	4	11	5
193	Tb927.8.1570	hypothetical protein, conserved	272	28.8		0.92	58	2	2	6	9		1	103	3	3	5	27
194	Tb927.10.6180	hypothetical protein, conserved	499	54.8		0.8	32	1	1	3	4		1	349	5	5	30	17
195	Tb927.10.1390	hypoxanthine-guanine phosphoribosyltransferase, putative	234	26.3		1	118	2	2	5	13		0.99	44	1	1	2	7
196	Tb927.11.12220	vacuolar transporter chaperone 4	793	91.3		1	138	5	5	10	10		1	329	8	8	27	16
197	Tb927.8.6660	paraflagellar rod component, putative	607	69.0		1	174	5	5	16	16		1	191	4	3	22	11
198	Tb927.8.4780	hypothetical protein, conserved	4151	467.8		1	199	6	6	17	2		0	8	1	1	1	1
199	Tb927.3.3460	hypothetical protein, conserved	483	53.2		1	233	5	4	10	15		0.36	33	2	2	6	8
200	Tb927.11.9080	hypothetical protein, conserved	1216	136.2		1	199	4	3	9	4		0	7	1	1	1	2
201	Tb927.11.14130	ribosomal protein L18, putative	179	20.9		0.96	294	8	6	17	40							
202	Tb927.10.4880	hypothetical protein, conserved	224	25.9		1	151	3	3	13	11		0.99	56	2	1	2	8
203	Tb927.9.11220	hypothetical protein, conserved	356	41.5		1	195	6	5	13	17		1	132	3	3	10	14
204	Tb927.8.3870	hypothetical protein, conserved	1205	127.6		1	181	4	4	15	4		1	91	2	2	2	2
205	Tb927.2.5160	chaperone protein DNAj, putative	400	44.3		1	148	3	3	8	16							
206	Tb927.10.240	peroxin 14, putative	366	39.9		1	177	7	7	14	23		1	136	4	3	8	13
207	Tb927.10.8230	protein disulfide isomerase,bloodstream- specific protein 2 precursor	497	55.5		1	337	7	7	20	19		1	73	2	2	5	6
208	Tb927.6.4210	aldehyde dehydrogenase, putative	595	64.5		1	254	5	4	15	16		1	215	5	4	9	11
209	Tb927.6.3150	Hydin,flagellar component	4521	500.8		1	218	8	8	19	3							
210	Tb927.10.7090	alternative oxidase	329	37.5		0.75	40	2	2	2	7		1	262	8	5	17	16
211	Tb927.11.1710	mitochondrial RNA binding protein 1,gBP21, MRP1	206	23.2		1	210	5	5	14	31							
212	Tb927.3.1810	hypothetical protein, conserved	326	36.1		1	63	2	2	5	16		1	178	4	2	5	17

213	Tb927.9.9450	hypothetical protein, conserved,zinc finger protein family member, putative	1030	114.7		1	190	4	3	33	5		0.77	64	3	3	5	4
214	Tb927.11.8160	dynein heavy chain, putative	4152	473.5		1	120	5	4	10	1		0	14	1	1	1	0
215	Tb927.4.3590	translation elongation factor 1-beta, putative	261	28.3		1	124	4	4	8	31		0.75	121	3	3	7	28
216	Tb927.9.2450	electon transport protein SCO1/SCO2, putative	323	37.4		1	149	3	2	4	11		1	88	3	3	4	16
217	Tb927.3.2440	serine/threonine-protein kinase, putative,protein kinase, putative	406	45.8		1	168	4	2	6	9							
218	Tb927.9.4190	fatty acyl CoA syntetase 1	698	78.9		1	131	4	4	7	10		1	165	4	4	13	9
219	Tb927.4.4070	mevalonate kinase, putative	329	35.5		1	167	4	4	7	17		1	203	4	3	9	16
220	Tb927.10.2530	adenylate kinase, putative	222	24.9		1	223	6	5	14	32							
221	Tb927.5.500	hypothetical protein, conserved	1403	153.5		1	239	5	5	12	7		0.32	42	2	2	2	2
222	Tb927.2.3800	mRNA processing protein, putative	492	55.4		1	171	3	2	4	12		1	61	1	1	3	6
223	Tb927.7.2170	hypothetical protein, conserved	1481	168.6		1	177	5	5	12	3							
224	Tb927.8.1990	peroxidoxin	226	25.6		1	188	4	4	12	16		0.25	22	1	1	1	5
225	Tb927.10.12840	mitochondrial carrier protein, putative,mitochondrial 2-oxoglutarate/malate carrier protein, putative	304	33.1		1	153	4	4	13	16		1	233	4	3	23	16
226	Tb927.7.2390	hypothetical protein, conserved	951	102.8		1	114	3	2	3	4		0	5	1	1	1	2
227	Tb927.4.1330	DNA topoisomerase IB, large subunit	696	79.2		1	262	5	5	30	8		1	101	3	3	6	4
228	Tb927.11.12230	heat shock protein HslVU, ATPase subunit HslU, putative,ATP-dependent hsl protease ATP-binding subunit hslU, putative	496	54.8		1	99	2	2	2	5		0.77	32	1	1	1	4
229	Tb927.8.3690	isocitrate dehydrogenase [NADP], mitochondrial precursor, putative	437	48.5		1	197	4	4	10	11		0	10	1	1	1	3
230	Tb927.11.15040	chaperonin HSP60, mitochondrial precursor, putative,heat shock protein 60	594	64.1		1	229	5	5	22	11		1	145	4	4	7	9
231	Tb927.6.3050	aldehyde dehydrogenase family, putative	543	59.6		1	118	4	4	7	11		1	52	1	1	3	3
232	Tb927.8.5120	cytochrome c	114	12.3		0.99	183	6	5	8	54							
233	Tb927.6.1500	alkyl-dihydroxyacetone phosphate synthase	613	69.0		1	145	5	5	12	13		0.86	53	2	2	3	7
234	Tb927.2.5660	adenylate kinase, putative	260	29.3		1	113	3	2	5	18		0	12	1	1	1	12
235	Tb927.10.5770	valosin-containing protein homolog,Transitional endoplasmic reticulum ATPase, putative	780	85.8		1	184	4	4	9	10		1	53	1	1	4	2
236	Tb927.11.5290	mitochondrial carrier protein, putative	282	29.8		1	146	3	3	9	14		0.88	39	2	2	2	10
237	Tb927.9.5690	60S acidic ribosomal protein, putative	113	11.1		1	114	3	3	6	58		0	13	1	1	2	29
238	Tb927.11.6140	40S ribosomal protein S15A, putative	130	14.6		1	124	2	2	6	17							
239	Tb927.11.600	hypothetical protein, conserved	106	12.1		1	173	4	3	10	42		0	4	1	1	1	22
240	Tb927.2.5800	sedoheptulose-1,7-bisphosphatase	332	35.5		1	240	5	5	19	21		1	165	4	4	7	19
241	Tb927.4.2080	hypothetical protein, conserved	906	104.8		1	203	4	4	11	6		1	197	5	5	8	9
242	Tb927.10.8780	hypothetical protein, conserved	2169	244.7		1	114	3	2	6	2							
243	Tb927.10.15750	hypothetical protein, conserved	1748	197.6		1	184	4	3	13	2		0.23	22	1	1	1	1
244	Tb927.9.4500	heat shock protein, putative,HSP70-like protein	833	90.8		1	91	2	2	5	4		0.99	56	2	2	3	4
245	Tb927.9.4680	ATP-dependent DEAD box helicase, putative	404	45.3		1	102	3	3	11	11		0.99	80	3	2	8	7
246	Tb927.4.870	dynein heavy chain, putative	4448	509.8		1	153	4	4	13	2		0.97	42	1	1	2	1
247	Tb927.10.5760	adenylate kinase, putative	215	24.3		1	199	5	5	14	27		0	10	1	1	1	7
248	Tb927.10.13180	hypothetical protein, conserved	1236	136.6		1	127	4	4	6	4		0	21	1	1	1	1
249	Tb927.11.14780	phosphomannose isomerase, putative	409	46.4		1	128	4	4	10	17		0.91	58	2	2	3	12
250	Tb927.2.2940	hypothetical protein, conserved	299	32.2		1	119	2	2	9	16		1	148	4	2	9	16
251	Tb927.10.10360	microtubule-associated protein, putative	3198	373.3		0.89	170	4	4	43	1		1	163	4	4	17	2
252	Tb927.4.1360	hypothetical protein, conserved	293	32.5		1	190	5	5	15	27							
253	Tb927.11.15150	1-acyl-sn-glycerol-3-phosphate acyltransferase, putative	271	30.3		1	98	3	3	8	11		0	7	1	1	1	7
254	Tb927.10.8200	hypothetical protein, conserved	269	30.8		0.78	50	2	2	4	9		1	195	4	3	5	21
255	Tb927.7.4900	5'-3' exonuclease XRNA, putative,exoribonuclease 1, putative	1418	158.6		1	188	4	4	11	6		1	111	3	3	3	3
256	Tb927.9.5960	succinate dehydrogenase, putative	188	21.2		1	221	5	5	17	34		0.83	32	1	1	1	7
257	Tb927.11.9400	hypothetical protein, conserved	887	95.8		1	142	4	2	7	5		1	170	3	1	5	4
258	Tb927.7.210	proline dehydrogenase	556	63.8		0.79	32	1	1	2	3		1	232	6	5	13	16
259	Tb927.11.8870	mitochondrial DEAD box protein,KREH1	546	60.6		1	109	3	2	10	6		0	14	1	1	1	3
260	Tb927.11.1980	hypothetical protein, conserved,zinc finger protein family member, putative	517	58.4		1	66	2	2	4	8		1	115	2	2	4	9
261	Tb927.4.4910	3,2-trans-enoyl-CoA isomerase, mitochondrial precursor, putative	400	45.3		1	117	4	4	9	13		0.87	35	1	1	4	5
262	Tb927.10.16150	ATP-dependent zinc metallopeptidase, putative,metallo-peptidase, Clan MA(E) Family M41	577	62.7		0.62	80	1	1	1	3		1	99	2	2	4	3
263	Tb927.11.8050	hypothetical protein, conserved	523	60.0		1	91	2	2	4	9		1	158	5	3	8	12
264	Tb927.10.2950	hypothetical protein, conserved	1867	206.7		1	103	2	2	4	1		0	7	1	1	2	1
265	Tb927.8.1550	paraflagellar rod component, putative	778	88.1		1	186	5	5	11	10		1	60	2	2	4	5
266	Tb927.5.4420	nucleolar RNA helicase II, putative,nucleolar RNA helicase Gu, putative	632	68.7		1	135	3	3	9	8		0.59	29	1	1	1	3
267	Tb927.10.770	hypothetical protein, conserved	632	69.1		1	202	4	3	8	5		1	117	2	1	5	3
268	Tb927.11.3360	hypothetical protein, conserved	909	102.8		1	99	2	2	3	2		1	60	2	2	2	2
269	Tb927.6.4980	40S ribosomal protein S14	144	15.5		1	226	6	6	19	29		0	4	1	1	1	15
270	Tb927.10.10280	microtubule-associated protein, putative	2371	267.4		1	209	5	5	51	2		0.67	112	3	3	13	2
271	Tb927.3.1840	3-oxo-5-alpha-steroid 4-dehydrogenase, putative	298	33.3		1	186	5	5	12	30		0	18	1	1	1	9
272	Tb927.11.4450	hypothetical protein, conserved	117	12.7		1	114	3	3	5	44							
273	Tb927.7.3370	hypothetical protein, conserved	596	67.4		1	102	4	3	7	9							
274	Tb927.8.4400	hypothetical protein, conserved	668	75.9		1	189	5	3	19	8		1	64	1	1	2	4
275	Tb927.11.10760	kinesin-like protein, putative	617	69.8		1	144	4	4	4	10		0.97	62	2	2	3	4
276	Tb927.10.9780	ATP-dependent DEAD/H RNA helicase, putative,ATP- dependent RNA helicase, putative	878	97.4		1	87	2	2	6	7		0.69	30	1	1	1	4
277	Tb927.11.16510	hypothetical protein, conserved	146	16.3		1	170	4	4	15	29							
278	Tb927.11.17000	hypothetical protein, conserved,leucine-rich repeat protein (LRRP), putative	1004	110.0		1	134	4	3	11	5		1	108	2	2	6	3
279	Tb927.9.15360	40S ribosomal protein S6, putative	250	28.4		1	156	3	2	9	10							
280	Tb927.10.170	pseudouridine synthase, Cbf5p	427	48.2		1	136	4	4	11	10		0.81	30	1	1	1	4
281	Tb927.3.5570	syntaxin, putative	260	29.2		1	134	3	2	5	7		1	77	2	1	2	4
282	Tb927.10.6630	ATP-dependent DEAD/H RNA helicase HEL64, putative	568	64.0		1	177	4	4	10	11		1	127	3	3	9	9
283	Tb927.8.2740	mitochondrial RNA binding protein	338	38.5		1	150	3	2	6	12		1	127	2	1	4	5
284	Tb927.10.2770	eukaryotic translation initiation factor 5, putative	382	42.9		1	197	4	4	13	11							
285	Tb927.7.3980	immunodominant antigen, putative,tc40 antigen-like	904	97.2		1	105	3	2	7	2		0.52	39	2	1	2	2
286	Tb927.3.3590	U3 small nucleolar ribonucleoprotein protein MPP10, putative	672	75.2		1	83	2	2	3	5		0	3	1	1	1	4
287	Tb927.11.11540	DNA topoisomerase II, putative	1424	162.6		1	155	4	4	13	3		0.94	36	1	1	2	1
288	Tb927.7.1290	hypothetical protein, conserved	242	27.0		1	104	4	4	9	10		0.59	28	1	1	1	6
289	Tb927.11.2790	hypothetical protein, conserved	709	80.0		1	117	2	2	6	2							
290	Tb927.8.2000	cyclophilin, putative	301	32.9		1	102	2	2	7	8							
291	Tb927.8.1860	pitrilysin-like metalloprotease,metallo-peptidase, Clan ME, Family M16C	1030	114.4		1	75	2	2	3	2		0.98	68	2	2	2	3
292	Tb927.11.6210	sterol 14-alpha-demethylase	481	54.3		1	147	4	3	9	11		1	82	2	2	2	11
293	Tb927.6.4130	hypothetical protein, conserved	104	11.7		1	150	5	4	14	38							
294	Tb927.9.13990	RNA-binding protein, putative	305	34.6		1	130	3	3	10	14		0.99	90	3	3	4	18

295	Tb927.6.2050	ribosome biogenesis regulatory protein (RRS1), putative	213	24.6	1	119	3	3	7	23						
296	Tb927.4.410	CAF 40	316	35.3	1	113	2	1	4	11	1	136	2	1	6	11
297	Tb927.7.280	cyclophilin-type peptidyl-prolyl cis-trans isomerase, putative	225	24.3	1	120	3	3	7	24						
298	Tb927.10.6050	clathrin heavy chain	1703	190.5	0.99	122	4	4	7	4	1	105	4	4	6	6
299	Tb927.9.14200	hypothetical protein, conserved	386	44.5	1	143	3	3	13	10	0.98	43	1	1	2	4
300	Tb927.3.5020	hypothetical protein, conserved	1413	155.7	1	149	4	3	57	4	0	29	2	1	10	1
301	Tb927.8.6390	lysophospholipase, putative,alpha/beta hydrolase, putative	280	30.1	1	107	3	3	8	17	0.99	45	1	1	2	4
302	Tb927.11.6050	hypothetical protein, conserved	1707	189.4	1	121	3	3	7	3	0.86	55	2	2	4	2
303	Tb927.2.4550	FtsJ cell division protein, putative	905	102.8	1	69	2	1	5	2	0.99	95	3	3	5	6
304	Tb927.9.15330	hypothetical protein, conserved	897	100.0	1	79	1	1	2	2	0	10	1	1	2	2
305	Tb927.11.5220	chaperone protein DNAj, putative	766	84.7	1	103	2	1	9	3	0.64	40	1	1	1	2
306	Tb927.11.3500	hypothetical protein, conserved	1017	115.1	1	86	2	2	7	2	1	62	2	2	4	4
307	Tb927.6.1470	hypothetical protein, conserved	223	27.5	1	131	3	3	6	9	1	174	4	3	9	9
308	Tb927.1.1700	hypothetical protein, conserved	493	55.0	1	120	3	2	6	9	0.85	101	4	2	7	9
309	Tb927.2.5130	hypothetical protein, conserved	390	43.5	1	104	2	1	3	9	0	15	1	1	1	7
310	Tb927.8.2630	kinesin, putative	750	85.1	0.99	42	1	1	4	3	1	79	2	2	4	5
311	Tb927.11.6300	40S ribosomal protein S5, putative	190	21.4	1	123	3	3	4	15						
312	Tb927.9.1750	hypothetical protein, conserved	4479	502.5	1	148	4	4	12	2						
313	Tb927.9.11000	small GTPase, putative,GTP-binding protein, putative	220	23.8	1	108	2	2	6	7	1	104	2	1	2	7
314	Tb927.9.11850	structural maintenance of chromosome 1, putative	1275	144.4	1	183	4	3	8	4	0	4	1	1	1	2
315	Tb927.10.4760	hypothetical protein, conserved	242	27.1	1	190	4	4	12	16	0	8	1	1	1	9
316	Tb927.6.3890	replication factor C, subunit 2, putative	347	38.8	1	121	4	3	8	14	1	75	2	2	3	10
317	Tb927.11.16480	enoyl-CoA hydratase/isomerase family protein, putative	362	40.2	1	88	2	2	6	8	0.73	55	3	3	5	16
318	Tb927.7.2190	hypothetical protein, conserved	266	29.2	1	56	1	1	4	8	0.76	30	1	1	2	5
319	Tb927.7.3430	cyclophilin-type peptidyl-prolyl cis-trans isomerase, putative	231	25.5	0.99	111	4	4	7	34						
320	Tb927.11.3770	hypothetical protein, conserved	262	28.9	1	122	3	2	4	12						
321	Tb927.7.3330	hypothetical protein, conserved	4334	502.3	1	122	3	3	14	1	0.9	41	2	2	2	1
322	Tb927.11.7900	mitochondrial RNA binding protein,RBP16,RNA- binding protein of 16 kDa	141	15.1	1	142	5	3	7	52						
323	Tb927.11.10240	hslVU complex proteolytic subunit, putative,hslVU complex proteolytic subunit, threonine peptidase, Clan T(1), family T1B	209	22.7	1	153	2	2	6	12						
324	Tb927.6.2640	importin alpha subunit, putative	533	58.0	0	22	1	1	1	5	1	51	1	1	5	5
325	Tb927.10.4000	methylglutaconyl-CoA hydratase, mitochondrial precursor, putative	277	29.2	1	156	3	3	12	25	1	96	2	2	8	15
326	Tb927.4.1540	hypothetical protein, conserved	508	55.2	1	114	2	2	8	5	1	80	1	1	6	4
327	Tb927.6.4630	kinetoplastid-specific phospho-protein phosphatase, putative	444	48.9	0	7	1	1	1	5	1	210	3	3	9	18
328	Tb927.11.5600	hypothetical protein, conserved	119	13.6	1	162	3	2	7	17	0	9	1	1	1	17
329	Tb927.10.5990	hypothetical protein, conserved	443	50.7	1	134	2	2	6	3	1	56	1	1	1	3
330	Tb927.2.3610	hypothetical protein, conserved	144	16.0	1	185	3	3	10	10						
331	Tb927.11.10150	hypothetical protein, conserved	1211	137.5	0.97	41	1	1	1	1	1	80	2	2	2	4
332	Tb927.7.640	hypothetical protein, conserved	334	37.3	1	130	2	2	15	5	1	115	3	2	5	5
333	Tb927.7.5940	Protein Associated with Differentiation	605	67.1	1	68	1	1	4	6						
334	Tb927.10.7380	hypothetical protein, conserved	349	39.7	1	172	3	2	7	16	1	100	1	1	7	12
335	Tb927.11.4680	hypothetical protein, conserved	346	40.0	1	122	3	3	6	17						
336	Tb927.9.2320	hypothetical protein, conserved	367	41.5	1	89	2	2	3	11	1	142	3	1	3	8
337	Tb927.10.11520	hypothetical protein, conserved	117	13.4	1	134	3	3	5	27						
338	Tb927.10.3040	hypothetical protein, conserved	135	15.5	1	171	4	4	18	20						
339	Tb927.10.14700	hypothetical protein, conserved	342	37.7	1	117	3	3	6	10						
340	Tb927.7.680	chaperone protein DNAj, putative	774	88.6	1	121	2	2	5	5	1	117	2	2	5	5
341	Tb927.5.4330	dihydrolipoamide branched chain transacylase, putative	439	47.6	1	112	4	4	8	12	0.99	50	2	2	3	7
342	Tb927.8.610	hypothetical protein, conserved	881	96.1	1	127	3	2	6	3	0	13	1	1	1	3
343	Tb927.4.4700	hypothetical protein, conserved	269	30.7	0.96	63	2	2	3	7						
344	Tb927.10.12590	hypothetical protein, conserved	933	105.8	1	131	2	2	6	5						
345	Tb927.7.3500	glutathione-S-transferase/glutaredoxin, putative	312	35.4	1	67	2	2	4	13	1	135	3	2	12	13
346	Tb927.11.900	isocitrate dehydrogenase, putative	413	46.7	1	131	3	3	14	13	0	12	1	1	1	5
347	Tb927.5.2790	mitochondrial DNA polymerase beta-PAK	813	87.2	1	143	5	4	8	9						
348	Tb927.5.4470	hypothetical protein, conserved	845	95.7	1	121	3	2	6	4	0.99	48	2	2	2	3
349	Tb927.3.5430	hypothetical protein, conserved	271	31.2	1	142	3	3	9	16						
350	Tb927.10.4080	hypothetical protein, conserved	1183	127.0	1	103	2	2	20	2	0.41	35	1	1	2	1
351	Tb927.10.7810	hypothetical protein, conserved	712	80.2	1	95	2	2	6	6						
352	Tb927.11.10690	hypothetical protein, conserved	408	45.2	0.78	30	1	1	1	2	0.99	73	2	2	6	10
353	Tb927.10.5840	translation elongation factor 1-beta, putative	201	21.9	0	17	2	2	2	18	1	135	4	4	5	45
354	Tb927.3.750	hypothetical protein, conserved	334	37.7	1	149	3	2	6	10						
355	Tb927.8.1270	hypothetical protein, conserved	562	62.0	1	90	3	3	6	7	0.97	39	1	1	2	3
356	Tb927.4.1920	GPI transamidase, putative	686	75.7	1	70	2	1	3	3	0.97	66	2	2	2	7
357	Tb927.7.3050	hypothetical protein, conserved	1165	131.5	0	18	1	1	1	2	1	66	1	1	2	2
358	Tb927.11.880	cyclophilin a,cyclophilin type peptidyl-prolyl cis-trans isomerase	177	18.7	1	70	2	2	3	19						
359	Tb927.1.2100	calpain-like cysteine peptidase, putative,cysteine peptidase, Clan CA, family C2, putative	1146	126.1	1	87	3	3	6	4						
360	Tb927.5.800	casein kinase I, isoform 2	332	38.3	1	89	3	3	4	15						
361	Tb927.10.8590	flagellum transition zone component	1278	134.4	1	129	2	1	6	3						
362	Tb927.10.7230	hypothetical protein, conserved	2521	284.5	0.99	60	2	2	5	1	0	11	1	1	1	1
363	Tb927.11.9530	14-3-3-I protein	262	30.3	1	91	2	2	3	16	1	128	3	2	5	16
364	Tb927.4.3450	hypothetical protein, conserved	114	13.7	1	78	1	1	4	21	0	5	1	1	1	21
365	Tb927.11.2430	dynein heavy chain, putative	4307	484.6	1	56	1	1	3	0	0	35	2	2	2	1
366	Tb927.10.1660	hypothetical protein, conserved	295	33.2	0.99	63	2	2	5	12	0.95	66	2	2	4	9
367	Tb927.11.1620	hypothetical protein, conserved	192	21.6	1	110	2	2	3	17						
368	Tb927.1.2670	flagellar protein PF16	513	56.1	1	89	2	2	5	4	1	49	1	1	1	2
369	Tb927.11.9570	hypothetical protein, conserved	298	32.9	1	135	3	2	4	14	0.44	66	2	1	2	6
370	Tb927.10.14980	intraflagellar transport protein IFT52, putative	655	72.3	0.4	25	1	1	1	2	1	73	2	2	5	6
371	Tb927.11.15550	NADH-cytochrome b5 reductase, putative	308	33.8	1	63	1	1	4	6	1	114	1	1	7	6
372	Tb927.3.4650	C-8 sterol isomerase, putative	221	24.5	0.99	68	3	2	7	7						
373	Tb927.7.2710	NADH-cytochrome b5 reductase, putative	287	31.8	1	88	1	1	4	6	1	93	1	1	3	6
374	Tb927.5.1710	ribonucleoprotein p18, mitochondrial precursor, putative	188	21.2	1	119	2	2	9	22						
375	Tb927.3.5370	hypothetical protein, conserved	313	34.2	1	87	2	2	5	12	0.26	24	1	1	1	7
376	Tb927.4.470	snoRNP protein GAR1, putative	229	21.8	0.99	59	2	2	5	17	0.96	64	2	2	7	17

377	Tb927.11.2610	hypothetical protein, conserved	461	50.8	0.97	85	3	3	5	8	1	82	2	2	3	6
378	Tb927.11.4880	hypothetical protein, conserved	274	30.2	1	138	4	4	8	18	0	22	1	1	1	6
379	Tb927.3.2310	flagellar component,PACRGA	300	33.7	1	66	2	2	4	7						
380	Tb927.11.5420	hypothetical protein, conserved	330	37.4	0.96	75	2	2	2	10						
381	Tb927.9.6870	RNA-binding protein, putative	148	17.5	1	64	2	2	6	19	0.26	22	1	1	2	9
382	Tb927.10.6220	5'-3' exoribonuclease XRND, putative,5'-3' exoribonuclease 2, putative	801	90.9	1	112	3	3	5	7	1	58	1	1	2	2
383	Tb927.7.1670	hypothetical protein, conserved	186	20.8	1	87	2	2	5	19						
384	Tb927.10.11760	pumilio/PUF RNA binding protein 6	843	93.0	0.99	81	3	2	8	3	0.99	43	1	1	2	2
385	Tb927.4.560	dynein heavy chain, putative	4232	479.5	1	46	1	1	7	1						
386	Tb927.5.2850	radial spoke protein RSP2, putative	518	57.5	0.99	99	3	2	6	10	1	126	2	1	5	7
387	Tb927.5.2780	mitochondrial DNA polymerase beta	407	46.1	1	119	2	2	7	12	1	49	1	1	4	8
388	Tb927.10.9920	hypothetical protein, conserved	410	44.1	1	138	4	3	8	12	0	4	1	1	1	5
389	Tb927.11.16830	Rab-like 5, small G protein	219	24.4	0.84	54	2	2	3	18	1	64	1	1	2	13
390	Tb927.7.920	dynein heavy chain, putative	4112	467.8	1	71	1	1	2	0						
391	Tb927.11.5180	hypothetical protein, conserved	250	27.6	1	90	2	2	9	8						
392	Tb927.10.15250	paraflagellar rod component, putative	485	57.8	1	52	1	1	2	3	0.99	45	1	1	2	3
393	Tb927.7.6660	chaperone protein DNAj, putative	257	29.8	1	93	2	2	7	12						
394	Tb927.3.3330	heat shock protein 20, putative	141	15.8	1	99	1	1	4	11						
395	Tb927.1.3950	D-alanine aminotransferase	504	55.7	1	60	1	1	4	3						
396	Tb927.8.4230	hypothetical protein, conserved	1082	118.9	0.99	48	1	1	2	2						
397	Tb927.8.7040	hypothetical protein, conserved	189	21.3	1	128	3	3	15	24						
398	Tb927.5.550	vacuolar ATP synthase, putative	383	42.8	1	97	1	1	4	7	1	83	1	1	12	7
399	Tb927.2.4110	mitochondrial processing peptidase alpha subunit, putative,metallo-peptidase, Clan ME, Family M16	469	52.0	1	109	2	2	7	5						
400	Tb927.9.10580	3-demethylubiquinone-9 3-methyltransferase, putative	288	31.2	0.99	68	2	2	3	9	0.37	44	1	1	1	3
401	Tb927.11.3290	hypothetical protein, conserved	1501	166.1	0.99	58	2	2	3	2	0.71	31	1	1	1	1
402	Tb927.11.4000	hypothetical protein, conserved	581	62.8	1	98	2	2	5	11						
403	Tb927.4.590	hypothetical protein, conserved	818	88.0	1	55	2	1	7	2	1	102	3	2	10	6
404	Tb927.4.600	hypothetical protein, conserved	317	34.4	0.99	56	2	2	5	8	0	14	1	1	1	8
405	Tb927.1.1580	cytochrome c oxidase assembly factor, putative,electron transport protein SCO1/2, putative	301	33.5	1	78	2	1	9	5	0.99	65	2	1	2	5
406	Tb927.6.2560	hypothetical protein, conserved	309	34.1	0.97	38	1	1	2	13	1	65	1	1	5	13
407	Tb927.11.4980	ATP-dependent DEAD/H RNA helicase, putative,ATP- dependent RNA helicase, putative	601	65.2	0.86	33	1	1	2	5	1	55	1	1	1	3
408	Tb927.11.13510	hypothetical protein, conserved	180	20.1	0.98	63	2	2	5	22						
409	Tb927.11.7780	hypothetical protein, conserved	419	46.2	0.99	87	3	3	7	10						
410	Tb927.11.7290	pantothenate kinase subunit, putative	1467	162.0	1	124	3	3	8	3						
411	Tb927.9.9860	hypothetical protein, conserved	720	79.8	1	93	2	2	3	3						
412	Tb927.11.2510	hypothetical protein, conserved	1292	145.6	0.98	81	2	2	5	2						
413	Tb927.7.3630	TPR-repeat-containing chaperone protein DNAJ, putative	528	60.1	0.93	36	1	1	57	5	1	83	2	2	92	12
414	Tb927.3.3450	ADP-ribosylation factor-like protein 3A, putative	178	19.9	1	70	2	1	7	9						
415	Tb927.10.15000	hypothetical protein, conserved	422	48.0	1	51	1	1	7	5	0	21	1	1	2	5
416	Tb927.11.10140	hypothetical protein, conserved	269	30.3	1	79	1	1	4	7	0.93	37	1	1	1	7
417	Tb927.11.3710	hypothetical protein, conserved	515	61.1	1	120	3	3	7	8	0.8	31	1	1	2	4
418	Tb927.10.11300	paraflagellar rod component, putative	126	14.3	0.99	79	2	1	7	20						
419	Tb927.7.4550	60S ribosomal protein-like	183	19.4	1	134	2	2	5	22						
420	Tb927.11.5820	hypothetical protein, conserved	302	32.2	1	80	2	1	3	3						
421	Tb927.10.3500	RNA-binding protein, putative	878	96.5	1	67	2	2	6	3	1	51	1	1	5	2
422	Tb927.10.9810	hypothetical protein, conserved	301	33.8	0.99	65	2	2	6	9						
423	Tb927.10.15170	hypothetical protein, conserved	695	77.3	1	103	3	3	3	5						
424	Tb927.9.2650	hypothetical protein, conserved	606	66.5	1	112	3	3	12	9	0	12	1	1	1	4
425	Tb927.11.6440	hypothetical protein, conserved	441	48.8	1	64	1	1	7	2	0.97	39	1	1	1	2
426	Tb927.5.1520	heat shock protein HslVU, ATPase subunit HslU, putative	475	52.5	0.98	49	1	1	2	3	0.9	55	2	2	3	9
427	Tb927.4.4690	hypothetical protein, conserved	278	31.3	1	74	2	2	4	10	1	96	2	1	2	6
428	Tb927.10.13720	RNA-binding protein, putative	377	40.6	1	75	2	2	15	8	0.94	85	3	2	6	8
429	Tb927.3.2660	hypothetical protein, conserved	729	78.7	0.97	75	2	2	4	6	1	75	1	1	1	4
430	Tb927.7.320	hypothetical protein, conserved	180	20.2	1	87	2	2	3	14						
431	Tb927.9.9060	p21 antigen protein, putative	190	21.0	1	53	1	1	1	13						
432	Tb927.11.10660	hypothetical protein, conserved	4083	450.0	0.99	41	1	1	2	1						
433	Tb927.11.14090	hypothetical protein, conserved	480	51.6	0.97	45	1	1	4	3	0	17	1	1	1	3
434	Tb927.9.8740	RNA-binding protein	327	36.9	1	114	2	1	4	5						
435	Tb927.7.7500	thymine-7-hydroxylase, putative	320	36.9	0	14	1	1	2	5	1	122	2	2	2	15
436	Tb927.9.8160	chaperone protein DNAj, putative	430	47.8	1	75	1	1	10	5	1	63	1	1	11	5
437	Tb927.2.100	retrotransposon hot spot protein 1 (RHS1), putative	829	94.9	0.99	102	3	3	9	6						
438	Tb927.11.10780	hypothetical protein, conserved	279	30.9	1	63	1	1	3	5						
439	Tb927.2.2970	mitochondrial carrier protein, putative	312	33.9	0	22	1	1	2	8	0.99	44	1	1	1	5
440	Tb927.10.4050	serine palmitoyltransferase, putative	557	61.3	1	85	1	1	6	5						
441	Tb04.24M18.150	hypothetical protein, conserved	162	18.0	0.99	57	2	2	5	30						
442	Tb927.6.4990	ATP synthase, epsilon chain, putative	182	20.1	1	58	1	1	6	8						
443	Tb927.10.9830	hypothetical protein, conserved	157	17.2	1	50	1	1	3	8						
444	Tb927.10.10140	paraflagellar rod component, putative	331	37.0	0	23	2	1	3	9	0.99	113	3	1	3	9
445	Tb927.6.1570	2-hydroxy-3-oxopropionate reductase, putative	300	31.7	1	64	1	1	3	3						
446	Tb927.8.6240	hypothetical protein, conserved	266	30.0	0.7	37	2	2	6	7	0.96	38	1	1	3	4
447	Tb927.11.1670	cysteine desulfurase	437	48.1	0.99	44	1	1	1	3	0	6	1	1	1	3
448	Tb927.10.1100	60S ribosomal protein L9, putative	189	21.8	1	62	1	1	2	5						
449	Tb927.3.2880	hypothetical protein, conserved	104	12.6	1	65	2	2	5	15	0	12	1	1	1	15
450	Tb927.11.14700	hypothetical protein, conserved	547	61.9	0.94	37	1	1	1	3	0.96	35	1	1	1	5
451	Tb927.10.7350	hypothetical protein, conserved	1794	197.7	0.98	55	2	2	5	2						
452	Tb927.2.270	retrotransposon hot spot protein (RHS, pseudogene), putative,retrotransposon hot spot protein 3 (RHS3), frameshift	581	65.7	1	71	2	2	4	6						
453	Tb927.10.11390	60S ribosomal protein L6, putative	192	21.2	0	13	1	1	1	9	1	68	2	2	2	22
454	Tb927.8.3290	DNA polymerase zeta catalytic subunit, putative	1958	216.7	0.98	65	2	1	8	1	0	15	1	1	1	1
455	Tb927.10.15360	hypothetical protein, conserved	1769	191.9	1	58	1	1	4	1						
456	Tb927.10.6640	COP-coated vesicle membrane protein erv25 precursor, putative,ER--golgi transport protein erv25 precursor, putative	253	28.2	0	16	1	1	1	11	1	103	2	1	4	11
457	Tb927.10.4040	3-keto-dihydrosphingosine reductase	348	38.0	1	62	1	1	3	6	0.96	56	2	1	2	6
458	Tb927.10.4130	NADH-ubiquinone oxidoreductase complex I subunit, putative,NDUFA5/B13 subunit, putative	265	31.0	1	53	1	1	2	3						

459	Tb927.3.1410	cytochrome oxidase subunit VII	165	19.2	1	71	2	2	11	12						
460	Tb927.11.4650	hypothetical protein, conserved	1522	167.8	1	60	2	1	3	2	0	15	1	1	1	2
461	Tb927.11.6230	pretranslocation protein, alpha subunit, putative,SEC61-like (pretranslocation process) protein, putative	481	53.6	1	100	2	1	4	3	0.37	24	1	1	1	3
462	Tb927.8.2020	agmatinase, putative	331	36.5	1	56	1	1	3	5						
463	Tb927.2.5270	dynein heavy chain, putative	4246	485.4	1	47	1	1	3	1						
464	Tb927.10.10010	60S acidic ribosomal protein, putative	226	25.6	1	99	2	2	7	9	0.94	37	1	1	1	5
465	Tb927.4.3890	ATP-dependent RNA helicase, putative	1093	122.7	1	68	2	2	5	3						
466	Tb927.11.10090	hypothetical protein, conserved	415	46.3	0.99	67	2	2	4	5						
467	Tb927.4.3070	hypothetical protein, conserved	448	51.4	0.99	71	2	2	6	8	0	20	1	1	1	6
468	Tb927.7.6350	NADH-ubiquinone oxidoreductase, mitochondrial, putative	273	30.9	0.99	46	1	1	1	4						
469	Tb927.8.4930	hypothetical protein, conserved	305	33.9	1	63	1	1	4	5	0.98	43	1	1	2	5
470	Tb927.1.90	retrotransposon hot spot protein (RHS, pseudogene), putative	592	67.2	0.93	46	2	2	2	6	1	49	1	1	2	2
471	Tb927.9.6920	hypothetical protein, conserved	362	41.3	0.97	36	1	1	2	7	0	8	1	1	1	7
472	Tb927.6.4320	hypothetical protein, conserved	399	44.4	0.97	38	1	1	1	3						
473	Tb927.7.6260	hypothetical protein, conserved	322	36.4	0	14	1	1	1	4	0.98	42	1	1	1	4
474	Tb927.11.4920	hypothetical protein, conserved	272	30.7	0.99	63	2	2	5	13						
475	Tb927.11.15850	hypothetical protein, conserved	754	86.7	1	56	1	1	1	4						
476	Tb927.10.510	hypothetical protein, conserved	334	37.0	0.99	54	2	2	5	11	1	83	2	2	3	11
477	Tb927.9.10520	hypothetical protein, conserved	253	29.6	0.98	64	2	2	6	10	0.91	35	1	1	3	5
478	Tb927.10.15710	mitochondrial carrier protein, putative	323	35.8	1	71	1	1	4	6						
479	Tb927.4.3840	nucleolar protein, putative	525	59.0	1	74	2	2	3	5						
480	Tb927.11.13890	hypothetical protein, conserved	268	30.7	1	65	1	1	3	6						
481	Tb927.7.5790	protein disulfide isomerase, putative	135	15.4	1	50	1	1	2	7						
482	Tb927.7.7260	kinesin, putative	1041	113.3	0.99	73	2	1	17	1	0.92	56	2	1	9	1
483	Tb927.4.1280	hypothetical protein, conserved	398	43.8	0.96	40	1	1	2	5						
484	Tb927.3.2080	hypothetical protein, conserved	260	30.6	0.99	67	2	2	5	6						
485	Tb927.11.7520	hypothetical protein, conserved	364	40.5	0.99	46	1	1	2	3						
486	Tb927.5.3800	glutamine hydrolysing (not ammonia-dependent) carbomoyl phosphate synthase, putative	1833	203.9	0.97	39	1	1	2	0						
487	Tb927.11.13310	hypothetical protein, conserved	302	34.5	1	56	1	1	2	6	0	10	1	1	1	6
488	Tb927.7.7210	hypothetical protein, conserved	696	77.5	0	9	1	1	1	3	1	84	1	1	4	3
489	Tb927.11.16750	hypothetical protein, conserved	234	25.8	1	74	2	2	9	15	0	17	1	1	1	6
490	Tb927.10.830	adenylate kinase, putative	260	29.7	1	84	1	1	3	5						
491	Tb927.7.6990	hypothetical protein, conserved	300	32.7	0.97	64	2	2	6	9						
492	Tb927.11.5250	hypothetical protein, conserved	1584	177.2	1	82	2	2	5	2						
493	Tb927.10.8980	hypothetical protein, conserved	169	17.6	0.97	39	1	1	5	7						
494	Tb927.11.12040	hypothetical protein, conserved	159	18.3	0.96	37	1	1	4	13						
495	Tb927.11.540	ABC transporter, putative	691	76.2	0.7	30	1	1	1	4	1	80	1	1	4	4
496	Tb927.11.6430	hypothetical protein, conserved	1259	134.6	0.98	65	2	2	4	3	0	11	1	1	2	1
497	Tb927.1.3450	hypothetical protein, conserved	791	84.8	1	79	1	1	2	2						
498	Tb927.8.6640	hypothetical protein, conserved	604	67.4	1	78	1	1	6	3						
499	Tb927.11.610	hypothetical protein, conserved	356	40.5	1	75	2	2	2	11						
500	Tb927.8.1870	Golgi/lysosome glycoprotein 1	616	67.5	1	44	1	1	4	5	0	9	1	1	1	5
501	Tb927.8.6050	hypothetical protein, conserved	253	27.3	1	77	1	1	4	11						
502	Tb927.6.2870	hypothetical protein, conserved	465	52.1	0.99	67	2	1	3	3	0.97	41	1	1	3	3
503	Tb927.6.630	hypothetical protein, conserved	730	78.7	0.96	38	1	1	1	4	1	66	1	1	3	4
504	Tb927.7.4290	hypothetical protein, conserved	297	33.7	1	52	1	1	4	4						
505	Tb927.5.4040	hypothetical protein, conserved	817	94.2	0.8	29	1	1	4	2	0.99	38	1	1	4	2
506	Tb927.10.12960	ras-related protein rab-5,small GTPase, putative	230	24.5	1	76	1	1	2	9	1	72	1	1	2	9
507	Tb927.10.2550	malate dehydrogenase-related	357	39.0	1	69	1	1	7	6	1	61	1	1	1	6
508	Tb927.3.1820	hypothetical protein, conserved	245	25.4	0.29	24	1	1	1	8	1	59	1	1	4	8
509	Tb927.6.3670	paraflagellar rod component, putative	3222	359.0	1	50	1	1	3	1	0	16	1	1	1	1
510	Tb927.5.1680	hypothetical protein, conserved	1751	194.6	1	72	1	1	9	1						
511	Tb927.8.4450	RNA-binding protein, putative	428	47.2	0.98	43	1	1	1	5						
512	Tb927.6.4080	hypothetical protein, conserved	205	24.7	0.99	44	1	1	1	7						
513	Tb927.6.1870	eukaryotic translation initiation factor 4e, putative	427	46.5	1	71	1	1	3	3						
514	Tb927.11.6660	hypothetical protein, conserved	935	103.5	1	64	1	1	4	2						
515	Tb927.9.14420	cyclophilin-like protein, putative	384	42.6	1	51	1	1	3	4	0	9	1	1	1	4
516	Tb927.10.9820	mitochondrial intermediate peptidase, putative,metallo-peptidase, Clan MA(E) Family M3	675	76.7	0.98	43	1	1	2	3	0	17	1	1	1	3
517	Tb927.11.10960	hypothetical protein, conserved	650	71.7	1	67	1	1	3	3						
518	Tb927.11.8800	hypothetical protein, conserved	277	30.6	1	57	1	1	2	5						
519	Tb927.5.2530	hypothetical protein, conserved	1039	114.8	0.98	39	1	1	2	1	0.99	44	1	1	3	1
520	Tb927.5.3390	ADG1, pseudogene	118	13.1	1	65	1	1	4	9						
521	Tb927.7.5680	deoxyribose-phosphate aldolase, putative	280	30.0	1	64	1	1	2	5						
522	Tb927.10.6610	chaperone protein DNAj, putative	274	31.2	1	58	1	1	2	4						
523	Tb927.11.9560	oxidoreductase, putative	544	58.6	1	63	1	1	1	3						
524	Tb927.10.3640	hypothetical protein, conserved	284	31.5	1	62	1	1	2	4	0.76	41	1	1	2	4
525	Tb927.6.4070	hypothetical protein, conserved	261	29.3	1	56	1	1	2	8						
526	Tb927.11.7100	hypothetical protein, conserved	203	22.7	1	48	1	1	2	5						
527	Tb927.11.1680	vesicular-fusion protein SEC18, putative	888	97.8	1	44	1	1	2	1	0.53	31	1	1	1	1
528	Tb927.8.5860	50S ribosomal protein L17, putative	301	35.0	1	60	1	1	1	4						
529	Tb927.10.14770	protein kinase, putative	640	70.5	0.99	47	1	1	2	2						
530	Tb927.7.4460	hypothetical protein, conserved	842	91.1	1	60	1	1	4	2						
531	Tb927.2.2160	paraflagellar rod component, putative	329	37.7	0.59	28	1	1	2	5	0.99	47	1	1	1	5
532	Tb927.10.360	hypothetical protein, conserved	303	33.8	1	56	1	1	2	5						
533	Tb927.7.1090	hypothetical protein, conserved	1541	171.0	1	56	1	1	2	2						
534	Tb927.11.8030	hypothetical protein, conserved	417	46.4	1	56	1	1	1	3						
535	Tb927.8.3050	hypothetical protein, conserved	567	61.9	1	55	1	1	2	2						
536	Tb927.11.10080	hypothetical protein, conserved	189	21.5	1	55	1	1	1	13						
537	Tb927.11.10210	hypothetical protein, conserved	960	107.1	0.99	45	1	1	2	1						
538	Tb927.6.5090	hypothetical protein, conserved	819	92.8	1	54	1	1	2	2						
539	Tb927.11.460	hypothetical protein, conserved,predicted WD40 repeat protein	948	103.6	0.99	41	1	1	5	1						
540	Tb927.11.11250	cytosolic malate dehydrogenase	328	35.1	0.99	47	1	1	1	7	0.26	24	1	1	1	7

541	Tb927.6.1550	hypothetical protein, conserved,leucine-rich repeat protein (LRRP), putative	353	38.8	0.99	45	1	1	4	3						
542	Tb927.7.540	chaperone protein DNAj, putative	457	50.6	1	46	1	1	2	5						
543	Tb927.7.5320	hypothetical protein, conserved	746	83.4	1	53	1	1	2	2						
544	Tb927.10.14720	peroxin 13	391	42.1	1	53	1	1	4	3						
545	Tb927.4.1600	hypothetical protein, conserved	234	27.0	1	52	1	1	1	5						
546	Tb927.9.3370	thioredoxin	107	12.1	0.98	44	1	1	1	10						
547	Tb927.10.10800	hypothetical protein, conserved	452	49.9	1	46	1	1	2	8	0.44	27	1	1	1	8
548	Tb927.7.3810	hypothetical protein, conserved	381	41.6	1	49	1	1	3	4						
549	Tb927.10.7700	ABC transporter, putative	668	75.0	1	48	1	1	2	2						
550	Tb927.8.8120	hypothetical protein, conserved	268	30.0	0.99	48	1	1	1	6	0.49	27	1	1	1	6
551	Tb927.7.2670	hypothetical protein, conserved,zinc finger protein family member, putative	461	49.6	0.99	44	1	1	2	2						
552	Tb927.9.10070	hypothetical protein, conserved	315	35.8	1	48	1	1	1	4						
553	Tb927.10.15950	TFIID-like protein, putative	262	28.8	1	48	1	1	1	5						
554	Tb927.5.1160	hypothetical protein, conserved	228	25.5	1	48	1	1	4	9						
555	Tb927.6.2720	calcium-binding protein, putative	359	41.6	1	48	1	1	1	3						
556	Tb927.11.15240	small GTPase, putative,ras-related protein rab-2a, putative	212	23.5	1	46	1	1	1	15						
557	Tb927.10.600	hypothetical protein, conserved	541	62.7							1	90	2	2	3	7
558	Tb927.9.15060	rRNA processing protein, putative	251	28.4							0.99	64	2	1	2	14
559	Tb927.9.5040	cAMP-specific phosphodiesterase	930	103.6							1	136	3	3	5	8
560	Tb927.8.4500	eukaryotic translation initation factor 4 gamma, putative	749	84.5							1	108	2	2	4	5
561	Tb927.9.5280	hypothetical protein, conserved	274	31.9							1	73	1	1	4	18
562	Tb927.2.4090	hypothetical protein, conserved	270	29.3							1	45	1	1	3	14
563	Tb927.8.4890	endoplasmic reticulum oxidoreductin, putative,pol-associated gene 1	442	49.2							1	59	1	1	1	8
564	Tb927.10.790	vesicle-associated membrane protein, putative,synaptobrevin, putative	239	26.5							1	56	1	1	1	7
565	Tb927.11.3240	T-complex protein 1, zeta subunit, putative	544	59.5							1	113	3	3	5	6
566	Tb927.11.10170	hypothetical protein, conserved	213	24.3							1	49	1	1	5	12
567	Tb927.10.10880	ATP-binding cassette protein, putative,ABC transporter, putative,ATPase, putative	684	77.6							0.98	66	2	2	2	7
568	Tb927.11.2570	hypothetical protein, conserved	573	63.1							1	93	1	1	1	6
569	Tb927.8.3150	T-complex protein 1, gamma subunit, putative	556	60.8							1	76	1	1	5	5
570	Tb927.11.11160	sodium/sulphate symporter, putative	728	81.4							1	54	1	1	2	2
571	Tb927.10.14030	hypothetical protein, conserved	449	50.4							1	68	1	1	1	3
572	Tb927.11.2120	hypothetical protein, conserved	788	88.8							1	58	1	1	2	3
573	Tb927.11.14960	pumilio/PUF RNA binding protein 7, putative	704	78.6							0.99	46	1	1	5	2
574	Tb927.6.4970	serine/arginine-rich protein specific kinase SRPK, putative,protein kinase, putative	723	82.6							1	55	1	1	2	3
575	Tb927.10.14390	hypothetical protein, conserved	555	60.7							1	52	1	1	3	5
576	Tb927.9.9940	PACRGB,flagellar component	312	35.3							1	52	1	1	1	9
577	Tb927.10.4430	pumillo RNA binding protein PUF1	572	63.8							1	51	1	1	2	5
578	Tb927.6.5210	variant surface glycoprotein (VSG, pseudogene), putative,variant surface glycoprotein, frameshift	523	55.8							0.99	51	1	1	1	2
579	Tb927.8.1240	electron transfer flavoprotein-ubiquinone oxidoreductase, putative	564	62.3							1	49	1	1	1	6
580	Tb927.10.12260	cytosolic nonspecific dipeptidase, putative,peptidase (M20/M25/M40 family), putative	475	51.5							0.99	48	1	1	2	4
