# Supplementary material for: Proteomic Analysis of the Acidocalcisome, an Organelle Conserved from Bacteria to Human Cells
Source: PLoS Pathog. 2014 Dec 11;10(12):e1004555. doi: 10.1371/journal.ppat.1004555 (PMC4263762; doi:10.1371/journal.ppat.1004555)
Supplement: S2 Table — Peptide list for all high confidence identifications (1% false discovery rate, protein probability ≥0.95) from the acidocalcisome data sets (ACCS1 and ACCS2). When high-confidence identifications to similar proteins were identified but peptide degeneracy limited discrimination, peptides matched to these indistinguishable proteins are represented in a single protein “group” as described in the Methods. (PDF) [file ppat.1004555.s012.pdf]

Table S2. Peptide list for all high confidence identifications (1% false discovery rate, protein probability ≥ 0.95) from the acidocalcisome data sets (ACCS1 and ACCS2). When high-confidence identifications to similar proteins were identified but peptide degeneracy limited discrimination, peptides matched to these indistinguishable proteins are represented in a single protein "group" as described in the Methods.

			ACCS1							
Protein Group	GeneID	Description	Probability	Score	Spectra	Total Peptides	Peptides (Paris Guidelines)	Coverage (%)	Peptide	Peptide Score
1	Tb927.6.3740	heat shock 70 kDa protein, mitochondrial precursor, putative	1.00	4164	393	83	52	73.8	ELVEVRNNAETQANTAER	39.1
	Tb927.6.3750	heat shock 70 kDa protein, mitochondrial precursor, putative							ELVEVRNNAETQANTAERQLTEWK	39.2
	Tb927.6.3800	heat shock 70 kDa protein, mitochondrial precursor, putative							EPFRGVNPDEAVALGAATLGGVLRGDVK	23.8
									ETAENFLGRKVSNAVVTCPAYFNDAQR Carbamidomethyl (C18)	49.5
									ETAENFLGRKVSNAVVTCPAYFNDAQR Deamidated (N13)- Carbamidomethyl (C18)	16.0
									ETAENFLGRKVSNAVVTCPAYFNDAQR Deamidated (N5)- Carbamidomethyl (C18)	42.9
									ETAENFLGRKVSNAVVTCPAYFNDAQR Deamidated (N5)- Deamidated (N13)- Carbamidomethyl (C18)	39.8
									FDDEHIQHDIKNVPYK	29.2
									FDDEHIQHDIKNVPYKIIR	13.9
									FESLADKLVQR	61.9
									GLVLLDVTPLSLGIETLGGVFTR	43.1
									GQEKLVGLAAK	31.3
									GVNPDEAVALGAATLGGVLR	35.3
									GVNPDEAVALGAATLGGVLRGDVK	35.1
									GVNPDEAVALGAATLGGVLRGDVK Deamidated (N3)	7.2
									GVPQIEVTFDIDANGICHVTAK Carbamidomethyl (C17)	17.8
									GVPQIEVTFDIDANGICHVTAK Deamidated (N14)- Carbamidomethyl (C17)	9.2
									IREAAEKAK	34.0
									KVSNAVVTCPAYFNDAQR Carbamidomethyl (C9)	63.1
									KVSNAVVTCPAYFNDAQR Carbamidomethyl (C9)- Deamidated (N14)	69.9
									KVSNAVVTCPAYFNDAQR Deamidated (N4)- Carbamidomethyl (C9)	73.5
									KVSNAVVTCPAYFNDAQRQATK Carbamidomethyl (C9)	34.5
									MIPKNTTIPTKK Oxidation (M1)	25.1
									MKETAENFLGR	59.7
									MKETAENFLGR Oxidation (M1)	56.6
									MKETAENFLGRK	41.7
									MKETAENFLGRK Oxidation (M1)	44.4
									MPKVVEAVK Oxidation (M1)	21.0
									NVPYKIIR	33.7
									QAITNPQSTFFAVKR	45.5
									QAITNPQSTFFAVKR Deamidated (Q7)	37.5
									QATKDAGTIAGLNVIR	52.0
									QATKDAGTIAGLNVIR Deamidated (N13)	35.3
									QATKDAGTIAGLNVIR Deamidated (Q1)	80.0
									QATKDAGTIAGLNVIR Deamidated (Q1)- Deamidated (N13)	63.5
									QCIKDAAVDLKEISEVVLVGGMTR Carbamidomethyl (C2)- Oxidation (M22)	10.3
									QFFGREPFRGVNPDEAVALGAATLGGVLR	13.7
									QLTEWKYVTDAEKENVR	51.2
									QLTEWKYVTDAEKENVR Deamidated (Q1)	48.3
									QYSPSQVGAFVLEK	44.8
									RFDDEHIQHDIK	29.7
									RFDDEHIQHDIKNVPYK	41.1
									RQAITNPQSTFFAVKR	42.1
									SKFESLADKLVQR	45.0
									SKFESLADKLVQR Deamidated (Q12)	22.1
									SNNGDAWVQDGNGKQYSPSQVGAFVLEK Deamidated (N12)	57.9
									SNNGDAWVQDGNGKQYSPSQVGAFVLEK Deamidated (N2)- Deamidated (N3)	47.4
									SNNGDAWVQDGNGKQYSPSQVGAFVLEK Deamidated (N3)	46.4
									SNNGDAWVQDGNGKQYSPSQVGAFVLEK Deamidated (N3)- Deamidated (N12)	64.5
									SNNGDAWVQDGNGKQYSPSQVGAFVLEK Deamidated (Q15)- Deamidated (N12)	35.5
									SNNGDAWVQDGNGKQYSPSQVGAFVLEK Deamidated (Q15)- Deamidated (N2)	58.6
									SNNGDAWVQDGNGKQYSPSQVGAFVLEK Deamidated (Q15)- Deamidated (N3)	27.9
									SNNGDAWVQDGNGKQYSPSQVGAFVLEK Deamidated (Q9)	41.0
									SQTFSTAADNQTQVGIK	29.6
									SQTFSTAADNQTQVGIK Deamidated (N10)	46.0
									TEYQQAAAANSGSSGSSSTEGQGEQQQQQASGEKKE	86.0
									TEYQQAAAANSGSSGSSSTEGQGEQQQQQASGEKKE Deamidated (N10)	5.3
									TEYQQAAAANSGSSGSSSTEGQGEQQQQQASGEKKE Deamidated (Q22)	11.7
									TLLAELRK	46.9
									TSGIDLSKER	62.2
									TTPSVVAFKGQEK	42.0
									TTPSVVAFKGQEKLVGLAAK	27.3
									VFQGEREMASDNQMMGQFDLVGIPPAPR Oxidation (M8)- Oxidation (M14)- Oxidation (M15)	30.0
									VFQGEREMASDNQMMGQFDLVGIPPAPR Oxidation (M8)- Oxidation (M14)- Oxidation (M15)- Deamidated (Q13)	21.2
									VLENTEGFR	51.2
									VLENTEGFRTTPSVVAFK	35.7
									VLENTEGFRTTPSVVAFKGQEK	47.6
									VMENPNVTKDELSASTDKLQK	31.5
									VMENPNVTKDELSASTDKLQK Oxidation (M2)	25.3
									VSNAVVTCPAYFNDAQR Carbamidomethyl (C8)	72.9
									VSNAVVTCPAYFNDAQR Carbamidomethyl (C8)- Deamidated (N13)	54.1
									VSNAVVTCPAYFNDAQRQATK Carbamidomethyl (C8)	10.4
									VSNAVVTCPAYFNDAQRQATKDAGTIAGLNVIR Carbamidomethyl (C8)	57.6
									VSNAVVTCPAYFNDAQRQATKDAGTIAGLNVIR Carbamidomethyl (C8)- Deamidated (Q18)	40.4
									VSNAVVTCPAYFNDAQRQATKDAGTIAGLNVIR Deamidated (N3)- Carbamidomethyl (C8)	72.7
									VVEAVK	30.5
									VVEAVKQFFGREPFR	33.7
									VVEAVKQFFGREPFR Deamidated (Q7)	22.9
									VVNEPTAAALAYGLDK	75.5
									VVNEPTAAALAYGLDKTK	81.4
									VVNEPTAAALAYGLDKTKDSLIAVYDLGGGTFDISVLEIAGGVFEVK	7.6
									YVTDAEKENVR	55.6
									YVTDAEKENVRTLLAELR	15.5
2	Tb927.11.6280	pyruvate phosphate dikinase	1.00	3628	336	80	54	60.8	AALDKLLPIQR	38.1
									AALDKLLPIQR Deamidated (Q10)	21.1
									AAVTADSIAQK	47.9
									AAVTADSIAQKADFFSFGTNDLTQMGCGFSR Oxidation (M25)- Carbamidomethyl (C27)	32.6
									AAVTADSIAQKADFFSFGTNDLTQMGCGFSRDDAGPFLR Carbamidomethyl (C27)	35.4
									AAVTADSIAQKADFFSFGTNDLTQMGCGFSRDDAGPFLR Deamidated (Q10)- Carbamidomethyl (C27)	37.0
									AAVTADSIAQKADFFSFGTNDLTQMGCGFSRDDAGPFLR Oxidation (M25)- Carbamidomethyl (C27)	51.2
									ADFFSFGTNDLTQMGCGFSRDDAGPFLR Carbamidomethyl (C16)	29.7
									ADFFSFGTNDLTQMGCGFSRDDAGPFLR Oxidation (M14)- Carbamidomethyl (C16)	73.6
									ADFVGILR	36.2
									AHGVGEEERR	37.8
									AMRGLPVTIR	33.0
									AMRGLPVTIR Oxidation (M2)	34.1
									ARSFGAEGVGLCR Carbamidomethyl (C12)	57.0
									CCVSGCGDMVIR Carbamidomethyl (C1)- Carbamidomethyl (C2)- Carbamidomethyl (C6)- Oxidation (M9)	39.7
									DDAGPFLRHYGNLGIYAQDPFQSIDQEGIGELVR	36.7
									EMILADSASGR Oxidation (M2)	33.2
									EMILADSASGRK	32.4
									EMILADSASGRK Oxidation (M2)	42.5
									FITMYADIVMQVGREDFEEALSR	5.0
									FITMYADIVMQVGREDFEEALSR Oxidation (M10)	36.9
									FITMYADIVMQVGREDFEEALSR Oxidation (M4)	16.5
									FITMYADIVMQVGREDFEEALSR Oxidation (M4)- Deamidated (Q11)	16.7
									FITMYADIVMQVGREDFEEALSR Oxidation (M4)- Oxidation (M10)	45.2

	ACCS2							
Protein Group	Probability	Score	Spectra	Total Peptides	Peptides (Paris Guidelines)	Coverage (%)	Peptide	Peptide Score
1	1.00	3981	347	79	41	66.5	ATNGDTHLGGEDFDLCLSDHILEEFRK Carbamidomethyl (C16)	23.9
							ATNGDTHLGGEDFDLCLSDHILEEFRK Deamidated (N3)- Carbamidomethyl (C16)	29.2
							DAGTIAGLNVIR	18.6
							ELVEVRNNAETQANTAER	56.6
							ELVEVRNNAETQANTAER Deamidated (N7)	27.6
							ELVEVRNNAETQANTAER Deamidated (N7)- Deamidated (N8)	33.2
							EPFRGVNPDEAVALGAATLGGVLRGDVK	50.0
							EPFRGVNPDEAVALGAATLGGVLRGDVK Deamidated (N7)	49.0
							ETAENFLGRKVSNAVVTCPAYFNDAQR Carbamidomethyl (C18)	47.6
							ETAENFLGRKVSNAVVTCPAYFNDAQR Carbamidomethyl (C18)- Deamidated (N23)	33.7
							ETAENFLGRKVSNAVVTCPAYFNDAQR Deamidated (N5)- Carbamidomethyl (C18)	26.1
							ETAENFLGRKVSNAVVTCPAYFNDAQR Deamidated (N5)- Deamidated (N13)- Deamidated (N23)- Carbamidomethyl (C18)	35.4
							ETAENFLGRKVSNAVVTCPAYFNDAQR Deamidated (N5)- Deamidated (N23)- Carbamidomethyl (C18)	41.1
							ETAENFLGRKVSNAVVTCPAYFNDAQR Deamidated (Q26)- Deamidated (N5)- Deamidated (N23)- Carbamidomethyl (C18)	29.4
							FDDEHIQHDIK	13.1
							GLVLLDVTPLSLGIETLGGVFTR	52.7
							GVNPDEAVALGAATLGGVLR	59.3
							GVNPDEAVALGAATLGGVLRGDVK	41.1
							GVNPDEAVALGAATLGGVLRGDVK Deamidated (N3)	19.1
							GVPQIEVTFDIDANGICHVTAK Carbamidomethyl (C17)	37.0
							GVPQIEVTFDIDANGICHVTAK Deamidated (N14)- Carbamidomethyl (C17)	20.5
							KSQTFSTAADNQTQVGIK	52.0
							KSQTFSTAADNQTQVGIK Deamidated (Q3)	81.5
							KVSNAVVTCPAYFNDAQR Carbamidomethyl (C9)	70.7
							KVSNAVVTCPAYFNDAQR Carbamidomethyl (C9)- Deamidated (N14)	63.0
							KVSNAVVTCPAYFNDAQR Deamidated (N4)- Deamidated (N14)- Carbamidomethyl (C9)	53.7
							KVSNAVVTCPAYFNDAQR Deamidated (Q17)- Deamidated (N4)- Deamidated (N14)- Carbamidomethyl (C9)	55.8
							MKETAENFLGR	26.3
							MKETAENFLGR Oxidation (M1)	56.4
							MKETAENFLGRK	46.8
							NVPYKIIR	25.6
							QAITNPQSTFFAVK	42.3
							QAITNPQSTFFAVK Deamidated (Q7)	33.3
							QAITNPQSTFFAVKR	22.0
							QATKDAGTIAGLNVIR Deamidated (Q1)	11.7
							QFFGREPFRGVNPDEAVALGAATLGGVLR	44.8
							QFFGREPFRGVNPDEAVALGAATLGGVLR Deamidated (N12)	39.7
							QLTEWKYVTDAEKENVR	56.0
							QLTEWKYVTDAEKENVR Deamidated (Q1)	58.3
							QLTEWKYVTDAEKENVR Deamidated (Q1)- Deamidated (N15)	0.3
							QYSPSQVGAFVLEK	52.5
							RFDDEHIQHDIK	28.8
							RFDDEHIQHDIK Deamidated (Q8)	30.3
							RFDDEHIQHDIKNVPYK	42.8
							RQAITNPQSTFFAVKR	35.7
							RQAITNPQSTFFAVKR Deamidated (N6)	25.7
							RQAITNPQSTFFAVKR Deamidated (Q8)	17.8
							SKFESLADKLVQR	60.4
							SNNGDAWVQDGNGKQYSPSQVGAFVLEK Deamidated (N2)	58.8
							SNNGDAWVQDGNGKQYSPSQVGAFVLEK Deamidated (N2)- Deamidated (N3)	41.3
							SNNGDAWVQDGNGKQYSPSQVGAFVLEK Deamidated (N3)	47.3
							SNNGDAWVQDGNGKQYSPSQVGAFVLEK Deamidated (N3)- Deamidated (N12)	59.9
							SNNGDAWVQDGNGKQYSPSQVGAFVLEK Deamidated (Q15)- Deamidated (N2)	78.7
							SNNGDAWVQDGNGKQYSPSQVGAFVLEK Deamidated (Q15)- Deamidated (N2)- Deamidated (N3)- Deamidated (N12)	44.9
							SNNGDAWVQDGNGKQYSPSQVGAFVLEK Deamidated (Q20)- Deamidated (N2)- Deamidated (N3)	16.9
							SNNGDAWVQDGNGKQYSPSQVGAFVLEK Deamidated (Q9)- Deamidated (N3)	35.7
							SQTFSTAADNQTQVGIK	27.0
							SQTFSTAADNQTQVGIK Deamidated (N10)	40.9
							SQTFSTAADNQTQVGIK Deamidated (Q11)	39.7
							SQTFSTAADNQTQVGIK Deamidated (Q13)	12.8
							TEYQQAAAANSGSSGSSSTEGQGEQQQQQASGEKKE	5.0
							TEYQQAAAANSGSSGSSSTEGQGEQQQQQASGEKKE Deamidated (N10)	74.7
							TEYQQAAAANSGSSGSSSTEGQGEQQQQQASGEKKE Deamidated (Q5)	91.9
							TLLAELRK	34.2
							TSGIDLSKER	40.3
							TTPSVVAFKGQEK	34.7
							TTPSVVAFKGQEKLVGLAAK	21.1
							VLENTEGFR	20.0
							VLENTEGFRTTPSVVAFKGQEK	3.8
							VMENPNVTKDELSASTDKLQK	32.7
							VMENPNVTKDELSASTDKLQK Oxidation (M2)	12.7
							VSNAVVTCPAYFNDAQR Carbamidomethyl (C8)	57.5
							VSNAVVTCPAYFNDAQR Carbamidomethyl (C8)- Deamidated (N13)	56.8
							VSNAVVTCPAYFNDAQR Deamidated (N3)- Carbamidomethyl (C8)	38.3
							VVEAVKQFFGREPFR	23.9
							VVNEPTAAALAYGLDK	11.1
							VVNEPTAAALAYGLDKTK	79.7
							VVNEPTAAALAYGLDKTKDSLIAVYDLGGGTFDISVLEIAGGVFEVK	14.8
							YVTDAEKENVR	57.6
2	1.00	3407	274	76	49	66.6	AALDKLLPIQR	37.9
							AALDKLLPIQR Deamidated (Q10)	32.4
							AAVTADSIAQK	39.4
							ADFFSFGTNDLTQMGCGFSR Carbamidomethyl (C16)	71.7
							ADFFSFGTNDLTQMGCGFSR Oxidation (M14)- Carbamidomethyl (C16)	54.2
							ADFFSFGTNDLTQMGCGFSRDDAGPFLR Carbamidomethyl (C16)	32.6
							ADFFSFGTNDLTQMGCGFSRDDAGPFLR Deamidated (N9)- Oxidation (M14)- Carbamidomethyl (C16)	27.4
							ADFFSFGTNDLTQMGCGFSRDDAGPFLR Oxidation (M14)- Carbamidomethyl (C16)	29.0
							ADFVGILR	9.9
							AHGVGEEERR	32.8
							EELSLIREEVVKTAEAVITK	24.0
							ELLGGKGANLAEMVNLGIPVPPGFTITTEACK Carbamidomethyl (C31)	20.6
							EMILADSASGR	54.1
							EMILADSASGRK	42.5
							FITMYADIVMQVGREDFEEALSR	20.3
							FITMYADIVMQVGREDFEEALSR Oxidation (M10)	33.4
							FITMYADIVMQVGREDFEEALSR Oxidation (M4)	21.1
							FVYDSYRR	17.0
							GANLAEMVNLGIPVPPGFTITTEACK Carbamidomethyl (C25)	32.9
							GANLAEMVNLGIPVPPGFTITTEACK Oxidation (M7)- Carbamidomethyl (C25)	7.5
							GANLAEMVNLGIPVPPGFTITTEACKTYQETETIPQEVADQVR Carbamidomethyl (C25)	42.0
							GANLAEMVNLGIPVPPGFTITTEACKTYQETETIPQEVADQVR Carbamidomethyl (C25)- Deamidated (Q29)	32.4
							GANLAEMVNLGIPVPPGFTITTEACKTYQETETIPQEVADQVR Deamidated (Q36)- Deamidated (Q41)- Deamidated (N3)- Deamidated (N9)- Carbamidomethyl (C25)	31.6
							GGMTSHAAVVAR	74.1

									FVYDSYRR	23.6
									GANLAEMVNLGIPVPPGFTITTEACK Oxidation (M7)- Carbamidomethyl (C25)	9.4
									GGMTSHAAVVAR Oxidation (M3)	52.0
									GLAASPGAAVGQVVFDAESAK	57.6
									GLAASPGAAVGQVVFDAESAK Deamidated (Q12)	7.0
									GLAASPGAAVGQVVFDAESAKEWSGR	59.0
									GLAASPGAAVGQVVFDAESAKEWSGR Deamidated (Q12)	12.2
									GLAASPGAAVGQVVFDAESAKEWSGRGK	37.8
									GLIYAGKLK	28.3
									HYGNLGIYAQDPFQSIDQEGIGELVR	58.4
									HYGNLGIYAQDPFQSIDQEGIGELVR Deamidated (Q10)	44.2
									HYGNLGIYAQDPFQSIDQEGIGELVR Deamidated (Q18)	7.8
									IAIDMVNEGLISREEAVLR Oxidation (M5)	14.7
									IDPYQVDHLMHPNLEPGAEKANKPIGR Oxidation (M10)	33.1
									INFIREMILADSASGR Oxidation (M7)	28.5
									INFIREMILADSASGRK	56.2
									INFIREMILADSASGRK Deamidated (N2)- Oxidation (M7)	35.6
									INFIREMILADSASGRK Oxidation (M7)	28.6
									KAALDKLLPIQR	39.1
									KAALDKLLPIQR Deamidated (Q11)	16.2
									LETSPEDLAGMDAACGILTAR Carbamidomethyl (C15)	56.5
									LETSPEDLAGMDAACGILTAR Oxidation (M11)- Carbamidomethyl (C15)	52.3
									LGITYPEIYNMQVR	23.0
									LGITYPEIYNMQVR Oxidation (M11)	24.5
									LGMPAEKVR	26.1
									LGMPAEKVR Oxidation (M3)	20.2
									LGVRTNADTPADAAK	45.1
									LGVRTNADTPADAAKAR	28.2
									LGVRTNADTPADAAKAR Deamidated (N6)	30.9
									LLCDVRK Carbamidomethyl (C3)	41.9
									LLDPPLHEFVPHDAAAQFELAQK	32.9
									LLDPPLHEFVPHDAAAQFELAQKLGMPAEK	6.9
									LLDPPLHEFVPHDAAAQFELAQKLGMPAEK Oxidation (M26)	24.5
									LLDPPLHEFVPHDAAAQFELAQKLGMPAEKVR	24.6
									MGICGEHGGDPATIGFCHK Oxidation (M1)- Carbamidomethyl (C4)- Carbamidomethyl (C17)	32.8
									NRVNALHELNPMLGHR	40.9
									NRVNALHELNPMLGHR Oxidation (M12)	29.0
									SATGVAFSR	27.0
									SFGAEGVGLCR Carbamidomethyl (C10)	55.0
									SFGAEGVGLCRTEHMFFEGSR Carbamidomethyl (C10)- Oxidation (M15)	23.9
									SGAAASMPGMMDTVLNLGLNK Oxidation (M7)- Oxidation (M10)- Oxidation (M11)	15.4
									SGAAASMPGMMDTVLNLGLNKVTVDAWVR Oxidation (M7)- Oxidation (M10)- Oxidation (M11)	11.2
									SGAAASMPGMMDTVLNLGLNKVTVDAWVR Oxidation (M7)- Oxidation (M10)- Oxidation (M11)- Deamidated (N16)	20.7
									SPSTGENFFFGEYLVNAQGEDVVAGIR	15.4
									SPSTGENFFFGEYLVNAQGEDVVAGIRTPQQINHSLSLR	132.3
									SPSTGENFFFGEYLVNAQGEDVVAGIRTPQQINHSLSLR Deamidated (N7)	120.0
									TAEAVITK	36.3
									TEHMFFEGSR Oxidation (M4)	17.4
									TNADTPADAAKAR	43.6
									TPQQINHSLSLR	33.8
									TYQETETIPQEVADQVR	29.4
									TYQETETIPQEVADQVRENVSR	16.0
									TYQETETIPQEVADQVRENVSRVEK	34.6
									VHYTVGTMIEVPR Oxidation (M8)	16.8
									VNALHELNPMLGHR	7.9
									VNALHELNPMLGHR Oxidation (M10)	38.3
3	Tb927.1.2330	beta tubulin	1.00	3320	484	72	32	71.3	AGPYGQIFRPDNFIFGQSGAGNNWAK	59.5
	Tb927.1.2350	beta tubulin							AGPYGQIFRPDNFIFGQSGAGNNWAK Deamidated (N12)	62.4
	Tb927.1.2370	beta tubulin							AGPYGQIFRPDNFIFGQSGAGNNWAK Deamidated (N22)	57.8
	Tb927.1.2390	beta tubulin							AGPYGQIFRPDNFIFGQSGAGNNWAK Deamidated (N23)	32.3
									AGPYGQIFRPDNFIFGQSGAGNNWAK Deamidated (Q17)	36.2
									AGPYGQIFRPDNFIFGQSGAGNNWAK Deamidated (Q6)	5.9
									AGPYGQIFRPDNFIFGQSGAGNNWAK Deamidated (Q6)- Deamidated (Q17)	78.2
									EAESCDCLQGFQICHSLGGGTGSGMGTLLISK Carbamidomethyl (C5)- Carbamidomethyl (C7)- Carbamidomethyl (C14)- Deamidated (Q9)- Oxidation (M25)	40.3
									EAESCDCLQGFQICHSLGGGTGSGMGTLLISK Carbamidomethyl (C5)- Carbamidomethyl (C7)- Carbamidomethyl (C14)- Oxidation (M25)	42.2
									EIVCVQAGQCGNQIGSK Carbamidomethyl (C4)- Carbamidomethyl (C10)	33.6
									EVDEQMLNVQNK Deamidated (Q5)- Oxidation (M6)	43.7
									EVDEQMLNVQNK Oxidation (M6)	47.8
									FPGQLNSDLR	30.0
									FPGQLNSDLRK	41.1
									FWEVISDEHGVDPTGTYQGDSDLQLER	36.1
									GHYTEGAELIDSVLDVCCK Carbamidomethyl (C17)- Carbamidomethyl (C18)	53.6
									GLSVPELTQQMFDAK	52.4
									GLSVPELTQQMFDAK Deamidated (Q9)	44.8
									GLSVPELTQQMFDAK Deamidated (Q9)- Oxidation (M11)	43.2
									GLSVPELTQQMFDAK Oxidation (M11)	44.7
									GLSVPELTQQMFDAKNMMQAADPR	23.2
									GLSVPELTQQMFDAKNMMQAADPR Deamidated (Q10)	15.7
									GLSVPELTQQMFDAKNMMQAADPR Deamidated (Q9)- Oxidation (M11)- Oxidation (M17)- Oxidation (M18)	17.5
									GLSVPELTQQMFDAKNMMQAADPR Oxidation (M11)- Oxidation (M17)- Oxidation (M18)	25.5
									GLSVPELTQQMFDAKNMMQAADPR Oxidation (M11)- Oxidation (M18)	16.0
									GLSVPELTQQMFDAKNMMQAADPR Oxidation (M17)- Oxidation (M18)	24.7
									HGRYLTASALFR	29.5
									IMMTFSIIPSPK Oxidation (M3)	42.6
									INVYFDEATGGR	56.5
									INVYFDEATGGR Deamidated (N2)	38.0
									INVYFDEATGGRYVPR	19.2
									INVYFDEATGGRYVPR Deamidated (N2)	17.1
									KLAVNLVPFPR	41.5
									KLAVNLVPFPR Deamidated (N5)	16.7
									LAVNLVPFPR	54.0
									LAVNLVPFPR Deamidated (N4)	37.8
									LHFFMMGFAPLTSR	44.7
									LHFFMMGFAPLTSR Oxidation (M5)	56.3
									LHFFMMGFAPLTSR Oxidation (M5)- Oxidation (M6)	26.6
									LHFFMMGFAPLTSR Oxidation (M6)	48.5
									LREQYPDR	23.5
									LREQYPDRIMMTFSIIPSPK Oxidation (M11)	17.0
									MAVTFIGNNTCIQEMFR Carbamidomethyl (C11)	11.9
									MAVTFIGNNTCIQEMFR Carbamidomethyl (C11)- Deamidated (Q13)	49.8
									MAVTFIGNNTCIQEMFR Carbamidomethyl (C11)- Oxidation (M15)	15.1
									MAVTFIGNNTCIQEMFR Deamidated (N8)- Carbamidomethyl (C11)	38.7
									MAVTFIGNNTCIQEMFR Oxidation (M1)- Carbamidomethyl (C11)	57.2
									MAVTFIGNNTCIQEMFR Oxidation (M1)- Deamidated (Q13)- Deamidated (N8)- Carbamidomethyl (C11)	59.1
									MAVTFIGNNTCIQEMFR Oxidation (M1)- Deamidated (Q13)- Deamidated (N9)- Carbamidomethyl (C11)	35.3
									MAVTFIGNNTCIQEMFR Oxidation (M1)- Oxidation (M15)- Carbamidomethyl (C11)	19.1
									MAVTFIGNNTCIQEMFRR Carbamidomethyl (C11)	38.9
									MAVTFIGNNTCIQEMFRR Oxidation (M1)- Carbamidomethyl (C11)	27.7
									MAVTFIGNNTCIQEMFRR Oxidation (M1)- Oxidation (M15)- Carbamidomethyl (C11)	18.7
									MREIVCVQAGQCGNQIGSK Carbamidomethyl (C6)- Carbamidomethyl (C12)	13.3
									MREIVCVQAGQCGNQIGSK Oxidation (M1)- Carbamidomethyl (C6)- Carbamidomethyl (C12)	42.9
									MSTKEVDEQMLNVQNK Oxidation (M1)- Oxidation (M10)	20.2
									MSTKEVDEQMLNVQNKNSSYFIEWIPNNIK Oxidation (M1)- Oxidation (M10)	46.9
									MSTKEVDEQMLNVQNKNSSYFIEWIPNNIK Oxidation (M1)- Oxidation (M10)- Deamidated (N15)	8.6
									MSTKEVDEQMLNVQNKNSSYFIEWIPNNIK Oxidation (M1)- Oxidation (M10)- Deamidated (Q14)	35.0

						GGMTSHAAVVAR Oxidation (M3)	30.1
						GLAASPGAAVGQVVFDAESAK	70.8
						GLAASPGAAVGQVVFDAESAKEWSGR	67.8
						GLAASPGAAVGQVVFDAESAKEWSGR Deamidated (Q12)	52.5
						GTKFDTDLTASDLKELCDGYLELFELK Carbamidomethyl (C17)	42.7
						HYGNLGIYAQDPFQSIDQEGIGELVR	56.3
						IAIDMVNEGLISREEAVLR	35.0
						IAIDMVNEGLISREEAVLR Deamidated (N7)	24.8
						IAIDMVNEGLISREEAVLR Oxidation (M5)	13.0
						IDPYQVDHLMHPNLEPGAEK	27.9
						IDPYQVDHLMHPNLEPGAEK Oxidation (M10)	28.7
						IDPYQVDHLMHPNLEPGAEKANKPIGR Oxidation (M10)	16.9
						INFIREMILADSASGR	21.5
						INFIREMILADSASGR Oxidation (M7)	10.0
						KAALDKLLPIQR	43.7
						KEELSLIREEVVK	34.2
						KRYPSMEEAMPENYR	3.1
						KWVYYFGGGK	31.5
						LETSPEDLAGMDAACGILTAR Carbamidomethyl (C15)	73.4
						LETSPEDLAGMDAACGILTAR Oxidation (M11)- Carbamidomethyl (C15)	68.3
						LGMPAEKVR	9.3
						LGMPAEKVR Oxidation (M3)	11.1
						LLCDVRK Carbamidomethyl (C3)	16.6
						LLDPPLHEFVPHDAAAQFELAQK	30.4
						LLDPPLHEFVPHDAAAQFELAQK Deamidated (Q17)	20.4
						LLDPPLHEFVPHDAAAQFELAQK Deamidated (Q22)	20.2
						MGICGEHGGDPATIGFCHK Carbamidomethyl (C4)- Carbamidomethyl (C17)	31.2
						MGICGEHGGDPATIGFCHK Oxidation (M1)- Carbamidomethyl (C4)- Carbamidomethyl (C17)	12.2
						NRVNALHELNPMLGHR	31.3
						NRVNALHELNPMLGHR Deamidated (N10)	36.0
						NRVNALHELNPMLGHR Oxidation (M12)	34.0
						SATGVAFSR	21.3
						SFGAEGVGLCR Carbamidomethyl (C10)	45.7
						SGAAASMPGMMDTVLNLGLNK	51.5
						SGAAASMPGMMDTVLNLGLNKVTVDAWVR	42.5
						SPSTGENFFFGEYLVNAQGEDVVAGIR	40.7
						SPSTGENFFFGEYLVNAQGEDVVAGIRTPQQINHSLSLR	118.2
						SPSTGENFFFGEYLVNAQGEDVVAGIRTPQQINHSLSLR Deamidated (N7)	83.2
						SPSTGENFFFGEYLVNAQGEDVVAGIRTPQQINHSLSLR Deamidated (N7)- Deamidated (N16)	39.3
						SPSTGENFFFGEYLVNAQGEDVVAGIRTPQQINHSLSLR Deamidated (Q18)	68.1
						TEHMFFEGSR	25.9
						TGCSFPQDPVMQLFAAIK Carbamidomethyl (C3)	23.4
						TPQQINHSLSLR	42.7
						TPQQINHSLSLR Deamidated (N6)	14.8
						TPQQINHSLSLR Deamidated (Q4)	11.0
						TYQETETIPQEVADQVR	42.9
						TYQETETIPQEVADQVRENVSR	14.6
						TYQETETIPQEVADQVRENVSRVEK	22.7
						VGLDYVSCSPFR Carbamidomethyl (C8)	38.8
						VGLDYVSCSPFRVPVAIVAAAHASIK Carbamidomethyl (C8)	42.0
						VGLDYVSCSPFRVPVAIVAAAHASIKDR Carbamidomethyl (C8)	33.7
						VHYTVGTMIEVPR Oxidation (M8)	20.4
1.00	2987	344	67	30	70.6	AGPYGQIFRPDNFIFGQSGAGNNWAK	54.9
						AGPYGQIFRPDNFIFGQSGAGNNWAK Deamidated (N12)	48.9
						AGPYGQIFRPDNFIFGQSGAGNNWAK Deamidated (N12)- Deamidated (N22)	90.7
						AGPYGQIFRPDNFIFGQSGAGNNWAK Deamidated (N12)- Deamidated (N23)	47.9
						AGPYGQIFRPDNFIFGQSGAGNNWAK Deamidated (N22)	57.9
						AGPYGQIFRPDNFIFGQSGAGNNWAK Deamidated (N23)	54.9
						AGPYGQIFRPDNFIFGQSGAGNNWAK Deamidated (Q17)	44.2
						AGPYGQIFRPDNFIFGQSGAGNNWAK Deamidated (Q17)- Deamidated (N23)	16.3
						AGPYGQIFRPDNFIFGQSGAGNNWAK Deamidated (Q6)	23.3
						AGPYGQIFRPDNFIFGQSGAGNNWAK Deamidated (Q6)- Deamidated (N12)	38.0
						AGPYGQIFRPDNFIFGQSGAGNNWAK Deamidated (Q6)- Deamidated (N22)	54.8
						AGPYGQIFRPDNFIFGQSGAGNNWAK Deamidated (Q6)- Deamidated (N22)- Deamidated (N23)	35.5
						AGPYGQIFRPDNFIFGQSGAGNNWAK Deamidated (Q6)- Deamidated (N23)	53.5
						AGPYGQIFRPDNFIFGQSGAGNNWAK Deamidated (Q6)- Deamidated (Q17)	47.9
						EAESCDCLQGFQICHSLGGGTGSGMGTLLISK Carbamidomethyl (C5)- Carbamidomethyl (C7)- Carbamidomethyl (C14)	41.5
						EAESCDCLQGFQICHSLGGGTGSGMGTLLISK Carbamidomethyl (C5)- Carbamidomethyl (C7)- Carbamidomethyl (C14)- Deamidated (Q9)	40.8
						EIVCVQAGQCGNQIGSK Carbamidomethyl (C4)- Carbamidomethyl (C10)	41.4
						EVDEQMLNVQNK	31.1
						EVDEQMLNVQNK Deamidated (Q5)- Oxidation (M6)	9.4
						EVDEQMLNVQNKNSSYFIEWIPNNIK	24.0
						FPGQLNSDLRK	32.9
						FPGQLNSDLRK Deamidated (N6)	29.9
						FWEVISDEHGVDPTGTYQGDSDLQLER	38.2
						FWEVISDEHGVDPTGTYQGDSDLQLER Deamidated (Q18)	33.8
						FWEVISDEHGVDPTGTYQGDSDLQLER Deamidated (Q24)	48.7
						GHYTEGAELIDSVLDVCCK Carbamidomethyl (C17)- Carbamidomethyl (C18)	57.3
						GLSVPELTQQMFDAK	15.2
						GLSVPELTQQMFDAK Deamidated (Q9)- Oxidation (M11)	33.7
						GLSVPELTQQMFDAKNMMQAADPR	23.0
						GLSVPELTQQMFDAKNMMQAADPR Oxidation (M11)- Oxidation (M17)- Oxidation (M18)	10.3
						IMMTFSIIPSPK	51.0
						IMMTFSIIPSPK Oxidation (M2)- Oxidation (M3)	35.9
						IMMTFSIIPSPK Oxidation (M3)	43.2
						INVYFDEATGGR	43.6
						INVYFDEATGGR Deamidated (N2)	56.2
						INVYFDEATGGRYVPR	29.2
						KLAVNLVPFPR	56.4
						KLAVNLVPFPR Deamidated (N5)	45.0
						LAVNLVPFPR	55.5
						LAVNLVPFPR Deamidated (N4)	40.7
						LAVNLVPFPRLHFFMMGFAPLTSR	9.1
						LHFFMMGFAPLTSR	19.1
						LHFFMMGFAPLTSR Oxidation (M5)- Oxidation (M6)	29.1
						LHFFMMGFAPLTSR Oxidation (M6)	25.3
						LREQYPDR	29.1
						LREQYPDRIMMTFSIIPSPK	15.1
						MAVTFIGNNTCIQEMFR Carbamidomethyl (C11)	40.5
						MAVTFIGNNTCIQEMFR Carbamidomethyl (C11)- Oxidation (M15)	31.1
						MAVTFIGNNTCIQEMFR Deamidated (N9)- Carbamidomethyl (C11)	10.0
						MAVTFIGNNTCIQEMFR Oxidation (M1)- Carbamidomethyl (C11)	5.2
						MAVTFIGNNTCIQEMFR Oxidation (M1)- Oxidation (M15)- Carbamidomethyl (C11)	11.2
						MAVTFIGNNTCIQEMFRR Carbamidomethyl (C11)	36.9
						MREIVCVQAGQCGNQIGSK Carbamidomethyl (C6)- Carbamidomethyl (C12)	41.2
						MREIVCVQAGQCGNQIGSK Oxidation (M1)- Carbamidomethyl (C6)- Carbamidomethyl (C12)	42.5
						MREIVCVQAGQCGNQIGSK Oxidation (M1)- Carbamidomethyl (C6)- Carbamidomethyl (C12)- Deamidated (Q8)	20.9
						MSTKEVDEQMLNVQNK	41.7
						MSTKEVDEQMLNVQNKNSSYFIEWIPNNIK	31.8
						NMMQAADPR	53.8
						NMMQAADPR Oxidation (M3)	0.1

									NMMQAADPR Oxidation (M3)	2.9
									NSSYFIEWIPNNIK	52.1
									NSSYFIEWIPNNIK Deamidated (N1)	49.9
									NSSYFIEWIPNNIKSSVCDIPPKGLK Carbamidomethyl (C18)	16.0
									NSSYFIEWIPNNIKSSVCDIPPKGLK Deamidated (N1)- Carbamidomethyl (C18)	31.3
									SSVCDIPPKGLK Carbamidomethyl (C4)	32.8
									SVLIDLEPGTMDSVR	44.6
									SVLIDLEPGTMDSVR Oxidation (M11)	44.9
									SVLIDLEPGTMDSVRAGPYGQIFRPDNFIFGQSGAGNNWAK Oxidation (M11)- Deamidated (Q21)	33.1
									VGEQFTLMFR	55.9
									VGEQFTLMFR Deamidated (Q4)- Oxidation (M8)	39.5
									VGEQFTLMFR Oxidation (M8)	46.7
									YLTASALFR	53.0
4	Tb927.10.6400	chaperonin HSP60, mitochondrial precursor	1.00	3190	338	65	44	73.3	AAVQEGIVPGGGAALLR	51.7
	Tb927.10.6510	chaperonin HSP60, mitochondrial precursor							AAVQEGIVPGGGAALLR Deamidated (Q4)	40.9
									ALDGLLQDQSLTADQR	68.6
									ALDGLLQDQSLTADQRTGVQIIR	41.7
									ASKALDGLLQDQSLTADQR	64.8
									ASKALDGLLQDQSLTADQR Deamidated (Q10)	87.4
									ASKALDGLLQDQSLTADQR Deamidated (Q18)	84.0
									ASKALDGLLQDQSLTADQRTGVQIIR	19.3
									ATITKDDTVLLNGGGDVAMMKER Deamidated (N12)- Oxidation (M19)- Oxidation (M20)	6.6
									ATITKDDTVLLNGGGDVAMMKER Oxidation (M19)- Oxidation (M20)	24.0
									AVEAVATTLGPK	59.3
									AVEAVATTLGPKGR	40.5
									AVEVILK	34.7
									AVEVILKNIESQSR	42.9
									AVEVILKNIESQSR Deamidated (N8)	3.7
									DRITDALCSTR Carbamidomethyl (C8)	21.8
									EGAVVVEKVLENTDAAVGYDAQLDRYVNMFEAGIIDPAR Oxidation (M29)	25.9
									ERVDLLR	27.0
									GMDRAVEVILKNIESQSR	34.2
									GMDRAVEVILKNIESQSR Oxidation (M2)	33.9
									GRNVIIEQSYGAPK	60.2
									GRNVIIEQSYGAPKITK	26.1
									GVQRAVEAVATTLGPK	76.4
									GYISPYFVTDAKTQKAELEDAFVLVSAK	21.7
									IACVKAPGFGDNK Carbamidomethyl (C3)	31.9
									ITDALCSTR Carbamidomethyl (C6)	21.0
									ITKDGVTVAK	27.8
									IVANAGR	23.7
									IVANAGREGAVVVEK	33.0
									IVANAGREGAVVVEK Deamidated (N4)	25.2
									IVANAGREGAVVVEKVLENTDAAVGYDAQLDR	33.6
									IVANAGREGAVVVEKVLENTDAAVGYDAQLDR Deamidated (N19)	12.0
									KATITKDDTVLLNGGGDVAMMK Oxidation (M20)- Oxidation (M21)	21.1
									KLNNIHTILPVLNHVVR	46.0
									KLNNIHTILPVLNHVVR Deamidated (N13)	39.8
									KLNNIHTILPVLNHVVR Deamidated (N3)	53.7
									KLNNIHTILPVLNHVVR Deamidated (N3)- Deamidated (N4)	59.9
									KLNNIHTILPVLNHVVR Deamidated (N4)	55.1
									KLNNIHTILPVLNHVVR Deamidated (N4)- Deamidated (N13)	58.5
									LAKLSGGVAVIR	32.4
									LNNIHTILPVLNHVVR	50.2
									NAVRLPAHR	37.4
									NVIIEQSYGAPK	43.5
									NVIIEQSYGAPKITK	69.4
									NVIIEQSYGAPKITKDGVTVAK	35.1
									QVCNKTNDLAGDGTTTSAVLVASIFSEGIK Carbamidomethyl (C3)	18.8
									QVCNKTNDLAGDGTTTSAVLVASIFSEGIK Carbamidomethyl (C3)- Deamidated (N7)	14.1
									SGRPLLIIADDVESEALTTMIFNKLQGK	21.9
									SGRPLLIIADDVESEALTTMIFNKLQGK Oxidation (M20)	19.5
									SIATGTNPIDMKR	43.0
									SIATGTNPIDMKR Oxidation (M11)	61.1
									SIEFKDPFENMGAQLVR	37.3
									SIEFKDPFENMGAQLVR Deamidated (N10)- Oxidation (M11)	27.1
									SIEFKDPFENMGAQLVR Oxidation (M11)	33.6
									TGVQIIR	30.5
									TNDLAGDGTTTSAVLVASIFSEGIK	18.2
									TNDLAGDGTTTSAVLVASIFSEGIKSIATGTNPIDMKR Deamidated (N2)- Oxidation (M36)	25.6
									TQKAELEDAFVLVSAK	47.5
									TVTNTENVVQVATISANGDVELGKLIGEAMEK Deamidated (N17)- Oxidation (M30)	24.5
									TVTNTENVVQVATISANGDVELGKLIGEAMEK Deamidated (Q10)	36.8
									VGGASEVEVNEKKDR	50.5
									VGKDGVITTQDGK	27.6
									VLENTDAAVGYDAQLDRYVNMFEAGIIDPAR	51.9
									VLENTDAAVGYDAQLDRYVNMFEAGIIDPAR Deamidated (N20)- Oxidation (M21)	46.6
									VLENTDAAVGYDAQLDRYVNMFEAGIIDPAR Oxidation (M21)	58.6
5	Tb927.3.1380	ATP synthase beta chain, mitochondrial precursor,ATP synthase F1, beta subunit	1.00	2841	191	52	31	60.9	AKVVSTGGNISVPVGR	70.2
									AKVVSTGGNISVPVGR Deamidated (N9)	56.5
									AKVVSTGGNISVPVGRETLGR	41.2
									ARVAQSALTMAEYFRDVEGQDVLLFIDNIFR	19.1
									AVAESGIYPAVNPLECASR Carbamidomethyl (C16)	71.4
									CIAMQTTDLLK Carbamidomethyl (C1)	37.5
									CIAMQTTDLLK Carbamidomethyl (C1)- Oxidation (M4)	44.5
									CIAMQTTDLLKLK Carbamidomethyl (C1)- Oxidation (M4)	34.9
									DVEGQDVLLFIDNIFR	50.8
									FTQANSEVSALLGRIPAAVGYQPTLAEDLGQLQER	96.0
									FTQANSEVSALLGRIPAAVGYQPTLAEDLGQLQER Deamidated (N5)	98.4
									FTQANSEVSALLGRIPAAVGYQPTLAEDLGQLQERITSTTK	33.6
									GHGGFSVFAGVGER	12.0
									GPVGEKLRMPIHAVAPK	14.3
									GPVGEKLRMPIHAVAPK Oxidation (M9)	24.2
									GSITSVQAVYVPADDITDPAPATTFSHLDATTVLDR	83.9
									GSITSVQAVYVPADDITDPAPATTFSHLDATTVLDR Deamidated (Q7)	75.2
									IFNVLGDAIDQR	71.3
									IFNVLGDAIDQR Deamidated (Q11)	63.2
									IFNVLGDAIDQRGPVGEK	75.4
									IFNVLGDAIDQRGPVGEKLR	30.9
									IFNVLGDAIDQRGPVGEKLR Deamidated (N3)	41.9
									IGLFGGAGVGKTVIIMELINNVAK Oxidation (M16)	43.3
									IMDPDVISVDHYNVAQDVVQMLTK Deamidated (Q16)- Oxidation (M21)	48.8
									IMDPDVISVDHYNVAQDVVQMLTK Oxidation (M2)	39.5
									IMDPDVISVDHYNVAQDVVQMLTK Oxidation (M2)- Oxidation (M21)	64.7
									IMDPDVISVDHYNVAQDVVQMLTK Oxidation (M21)	47.5
									IPAAVGYQPTLAEDLGQLQER	69.4
									IPAAVGYQPTLAEDLGQLQERITSTTK	32.0
									LADQAAEDAVLTTGIK	52.2
									LADQAAEDAVLTTGIKVIDLILPYCK Carbamidomethyl (C25)	50.0
									LADQAAEDAVLTTGIKVIDLILPYCKGGK Carbamidomethyl (C25)	57.3
									LADQAAEDAVLTTGIKVIDLILPYCKGGK Deamidated (Q4)- Carbamidomethyl (C25)	24.5
									LRMPIHAVAPK	25.7
									LRMPIHAVAPK Oxidation (M3)	29.6
									TVIIMELINNVAK	53.1

							NSSYFIEWIPNNIKSSVCDIPPKGLK Carbamidomethyl (C18)	18.2
							NSSYFIEWIPNNIKSSVCDIPPKGLK Deamidated (N1)- Carbamidomethyl (C18)	8.0
							NSSYFIEWIPNNIKSSVCDIPPKGLK Deamidated (N11)- Carbamidomethyl (C18)	20.2
							SSVCDIPPKGLK Carbamidomethyl (C4)	27.2
							SVLIDLEPGTMDSVR	10.4
							VGEQFTLMFR	58.7
							VGEQFTLMFR Oxidation (M8)	47.3
							YLTASALFR	39.4
4	1.00	2754	183	58	37	68.0	AAVQEGIVPGGGAALLR	45.6
							AELEDAFVLVSAK	52.8
							ALDGLLQDQSLTADQR	15.9
							ALDGLLQDQSLTADQR Deamidated (Q15)	19.7
							ASKALDGLLQDQSLTADQR	63.4
							ASKALDGLLQDQSLTADQR Deamidated (Q10)	39.4
							AVEAVATTLGPK	41.6
							AVEAVATTLGPKGR	28.2
							AVEVILK	33.1
							AVEVILKNIESQSR	58.4
							DRITDALCSTR Carbamidomethyl (C8)	18.6
							GLIERETSDYNR	20.5
							GRNVIIEQSYGAPK	46.8
							GYISPYFVTDAK	48.2
							GYISPYFVTDAKTQKAELEDAFVLVSAK	32.5
							IACVKAPGFGDNK Carbamidomethyl (C3)	45.3
							ITDALCSTR Carbamidomethyl (C6)	22.1
							ITKDGVTVAK	30.3
							IVANAGR	13.8
							IVANAGREGAVVVEK	30.9
							IVANAGREGAVVVEK Deamidated (N4)	55.9
							IVANAGREGAVVVEKVLENTDAAVGYDAQLDR	33.7
							KLNNIHTILPVLNHVVR	42.0
							KLNNIHTILPVLNHVVR Deamidated (N13)	37.7
							KLNNIHTILPVLNHVVR Deamidated (N3)	53.0
							KLNNIHTILPVLNHVVR Deamidated (N3)- Deamidated (N13)	59.6
							KLNNIHTILPVLNHVVR Deamidated (N4)	67.4
							KLNNIHTILPVLNHVVR Deamidated (N4)- Deamidated (N13)	32.4
							LNNIHTILPVLNHVVR	43.6
							LNNIHTILPVLNHVVR Deamidated (N2)	35.3
							NVIIEQSYGAPK	31.7
							NVIIEQSYGAPK Deamidated (N1)	8.5
							QVCNKTNDLAGDGTTTSAVLVASIFSEGIK Carbamidomethyl (C3)	18.1
							QVCNKTNDLAGDGTTTSAVLVASIFSEGIK Carbamidomethyl (C3)- Deamidated (N7)	13.3
							SGRPLLIIADDVESEALTTMIFNK	27.2
							SGRPLLIIADDVESEALTTMIFNKLQGK	51.5
							SGRPLLIIADDVESEALTTMIFNKLQGK Deamidated (N23)	73.5
							SGRPLLIIADDVESEALTTMIFNKLQGK Oxidation (M20)	23.1
							SIATGTNPIDMK	7.6
							SIATGTNPIDMKR	43.1
							SIATGTNPIDMKR Oxidation (M11)	62.1
							SIEFKDPFENMGAQLVR	59.6
							SIEFKDPFENMGAQLVR Deamidated (N10)	2.4
							TGVQIIR	20.2
							TNDLAGDGTTTSAVLVASIFSEGIK	54.7
							TQKAELEDAFVLVSAK	54.4
							TQKAELEDAFVLVSAK Deamidated (Q2)	17.1
							TVTNTENVVQVATISANGDVELGKLIGEAMEK	12.4
							TVTNTENVVQVATISANGDVELGKLIGEAMEK Deamidated (N17)	61.0
							TVTNTENVVQVATISANGDVELGKLIGEAMEK Deamidated (N17)- Oxidation (M30)	9.5
							VGGASEVEVNEKKDR	20.5
							VGKDGVITTQDGK	40.2
							VGKDGVITTQDGKTLTTELEVVEGMSVDR	42.8
							VGKDGVITTQDGKTLTTELEVVEGMSVDR Oxidation (M25)	19.0
							VLENTDAAVGYDAQLDR	61.5
							VLENTDAAVGYDAQLDRYVNMFEAGIIDPAR	70.8
							VLENTDAAVGYDAQLDRYVNMFEAGIIDPAR Deamidated (N20)	64.9
							VLENTDAAVGYDAQLDRYVNMFEAGIIDPAR Oxidation (M21)	35.2
5	1.00	2923	272	52	30	60.9	AKVVSTGGNISVPVGR	56.9
							AKVVSTGGNISVPVGRETLGR	38.4
							AKVVSTGGNISVPVGRETLGR Deamidated (N9)	12.6
							AVAESGIYPAVNPLECASR Carbamidomethyl (C16)	30.8
							AVAESGIYPAVNPLECASR Deamidated (N12)- Carbamidomethyl (C16)	54.5
							CIAMQTTDLLKLK Carbamidomethyl (C1)- Oxidation (M4)	8.0
							CIAMQTTDLLKLK Carbamidomethyl (C1)- Oxidation (M4)- Deamidated (Q5)	8.6
							DVEGQDVLLFIDNIFR	60.9
							DVEGQDVLLFIDNIFR Deamidated (N13)	61.6
							DVEGQDVLLFIDNIFR Deamidated (Q5)	44.3
							ELQDIIAVLGIDELSEEDKLIVDR	41.5
							ELQDIIAVLGIDELSEEDKLIVDRAR	33.1
							FTQANSEVSALLGR	72.1
							FTQANSEVSALLGR Deamidated (N5)	83.7
							FTQANSEVSALLGRIPAAVGYQPTLAEDLGQLQER	65.8
							FTQANSEVSALLGRIPAAVGYQPTLAEDLGQLQER Deamidated (N5)	77.5
							FTQANSEVSALLGRIPAAVGYQPTLAEDLGQLQER Deamidated (Q3)	73.9
							FTQANSEVSALLGRIPAAVGYQPTLAEDLGQLQER Deamidated (Q33)- Deamidated (N5)	72.6
							FTQANSEVSALLGRIPAAVGYQPTLAEDLGQLQERITSTTK	16.4
							GHGGFSVFAGVGER	62.7
							GSITSVQAVYVPADDITDPAPATTFSHLDATTVLDR	65.2
							GSITSVQAVYVPADDITDPAPATTFSHLDATTVLDR Deamidated (Q7)	45.4
							IFNVLGDAIDQR	75.2
							IFNVLGDAIDQR Deamidated (Q11)	56.7
							IFNVLGDAIDQRGPVGEK	59.0
							IFNVLGDAIDQRGPVGEK Deamidated (Q11)	30.6
							IFNVLGDAIDQRGPVGEKLR	32.0
							IGLFGGAGVGK	38.5
							IMDPDVISVDHYNVAQDVVQMLTK	59.3
							IMDPDVISVDHYNVAQDVVQMLTK Deamidated (Q16)- Oxidation (M21)	35.5
							IMDPDVISVDHYNVAQDVVQMLTK Oxidation (M2)	39.3
							IMDPDVISVDHYNVAQDVVQMLTK Oxidation (M21)	27.9
							IPAAVGYQPTLAEDLGQLQER	78.7
							IPAAVGYQPTLAEDLGQLQER Deamidated (Q19)	55.5
							LADQAAEDAVLTTGIKVIDLILPYCK Carbamidomethyl (C25)	46.7
							LADQAAEDAVLTTGIKVIDLILPYCKGGK Carbamidomethyl (C25)	50.3

									TVIIMELINNVAK Oxidation (M5)	51.7							LRMPIHAVAPK	30.8
									VAQSALTMAEYFR Oxidation (M8)	31.7							LRMPIHAVAPK Oxidation (M3)	15.5
									VAQSALTMAEYFRDVEGQDVLLFIDNIFR	40.1							MPIHAVAPK	42.3
									VAQSALTMAEYFRDVEGQDVLLFIDNIFR Deamidated (N26)	45.7							TVIIMELINNVAK	54.1
									VAQSALTMAEYFRDVEGQDVLLFIDNIFR Deamidated (Q18)	39.9							VAQSALTMAEYFR	59.3
									VAQSALTMAEYFRDVEGQDVLLFIDNIFR Deamidated (Q3)	41.2							VAQSALTMAEYFRDVEGQDVLLFIDNIFR	43.5
									VAQSALTMAEYFRDVEGQDVLLFIDNIFR Deamidated (Q3)- Oxidation (M8)	45.0							VAQSALTMAEYFRDVEGQDVLLFIDNIFR Deamidated (Q18)	27.4
									VAQSALTMAEYFRDVEGQDVLLFIDNIFR Oxidation (M8)	49.7							VAQSALTMAEYFRDVEGQDVLLFIDNIFR Deamidated (Q3)	7.7
									VAQSALTMAEYFRDVEGQDVLLFIDNIFR Oxidation (M8)- Deamidated (N26)	58.7							VAQSALTMAEYFRDVEGQDVLLFIDNIFR Oxidation (M8)	46.8
									VIDLILPYCKGGK Carbamidomethyl (C9)	32.3							VAQSALTMAEYFRDVEGQDVLLFIDNIFR Oxidation (M8)- Deamidated (Q18)	44.0
									VIDLKGESK	17.9							VIDLILPYCKGGK Carbamidomethyl (C9)	16.4
									VVSTGGNISVPVGR	37.6							VIDLKGESK	11.6
									VVSTGGNISVPVGRETLGR	41.4							VVSTGGNISVPVGR	26.3
									VVSTGGNISVPVGRETLGR Deamidated (N7)	7.2							VVSTGGNISVPVGRETLGR	28.1
									YRELQDIIAVLGIDELSEEDKLIVDR	26.5							YRELQDIIAVLGIDELSEEDKLIVDR	44.4
									YRELQDIIAVLGIDELSEEDKLIVDR Deamidated (Q5)	15.1							YRELQDIIAVLGIDELSEEDKLIVDR Deamidated (Q5)	28.9
6	Tb927.2.4210	glycosomal phosphoenolpyruvate carboxykinase	1.00	2459	328	54	38	65.1	ADPTVPGVTSTTSVALNFK	4.7	1.00	2769	245	59	39	74.5	ADPTVPGVTSTTSVALNFK	30.6
									AIGLNPETEKDIYEAVR	51.3							AIGLNPETEKDIYEAVR	48.3
									AIGLNPETEKDIYEAVR Deamidated (N5)	24.1							AIGLNPETEKDIYEAVR Deamidated (N5)	29.9
									AIGLNPETEKDIYEAVRFGAVAENCTLDR Carbamidomethyl (C25)	21.4							AIGLNPETEKDIYEAVRFGAVAENCTLDR Carbamidomethyl (C25)	18.8
									AIIDAIHDGTLDQADYEVYPGWGLHIPK	37.0							AIIDAIHDGTLDQADYEVYPGWGLHIPK	39.0
									AIIDAIHDGTLDQADYEVYPGWGLHIPKR	67.5							AIIDAIHDGTLDQADYEVYPGWGLHIPK Deamidated (Q13)	23.2
									APIIHKNLTAPELVEWALKLEK	23.2							AIIDAIHDGTLDQADYEVYPGWGLHIPKR	55.2
									ASEEMKGAVPR	53.3							AIIDAIHDGTLDQADYEVYPGWGLHIPKR Deamidated (Q13)	37.8
									ASEEMKGAVPR Oxidation (M5)	37.9							APIIHKNLTAPELVEWALKLEK	3.4
									AVAGHPKNIIFLTNDAFGVMPPVAR Oxidation (M20)	38.1							ASEEMKGAVPR	54.2
									AWTDLKQFNETTKELVTMFQK	8.7							FGAVAENCTLDR Carbamidomethyl (C8)	56.0
									AWTDLKQFNETTKELVTMFQK Oxidation (M18)	22.0							FGAVAENCTLDR Deamidated (N7)- Carbamidomethyl (C8)	61.2
									FGAVAENCTLDR Carbamidomethyl (C8)	62.6							FGAVAENCTLDRR Carbamidomethyl (C8)	22.1
									FGAVAENCTLDRR Carbamidomethyl (C8)	32.5							GILTVMFELMPR	37.0
									FGAVAENCTLDRR Deamidated (N7)- Carbamidomethyl (C8)	34.7							GVFNIEGGCYAK Carbamidomethyl (C9)	34.4
									FGAVAENCTLDRRTHEIDFNDESICK Carbamidomethyl (C8)- Carbamidomethyl (C25)	20.8							HATHYGQQLAEK	50.3
									GVFNIEGGCYAK Carbamidomethyl (C9)	36.2							HATHYGQQLAEKMEK Oxidation (M13)	15.5
									HATHYGQQLAEK	22.4							IVNTTDVTDNVDWGSVNMK	68.0
									HATHYGQQLAEKMEK Oxidation (M13)	20.8							KAWTDLKQFNETTK Deamidated (Q8)	13.0
									KAWTDLKQFNETTK	62.3							KGILTVMFELMPR	57.9
									LEKDSQLTAR Deamidated (Q6)	21.1							LEKDSQLTAR	20.3
									LTEESFEKLK	36.6							LEKDSQLTAR Deamidated (Q6)	40.4
									NIIFLTNDAFGVMPPVAR	29.8							LTEESFEKLK	45.6
									NIIFLTNDAFGVMPPVAR Oxidation (M13)	35.9							LTSAQAMFWFVMGYTANVPGVEAGSAR	20.5
									NLIGDDEHVWTDR	47.7							NIIFLTNDAFGVMPPVAR	28.8
									NLIGDDEHVWTDRGVFNIEGGCYAK Carbamidomethyl (C22)	24.3							NIIFLTNDAFGVMPPVAR Deamidated (N7)	24.5
									NLIGDDEHVWTDRGVFNIEGGCYAK Deamidated (N1)- Carbamidomethyl (C22)	24.2							NIIFLTNDAFGVMPPVAR Oxidation (M13)	18.9
									NLIGDDEHVWTDRGVFNIEGGCYAK Deamidated (N1)- Deamidated (N17)- Carbamidomethyl (C22)	16.1							NLIGDDEHVWTDR	47.8
									NLIGDDEHVWTDRGVFNIEGGCYAK Deamidated (N17)- Carbamidomethyl (C22)	25.0							NLIGDDEHVWTDRGVFNIEGGCYAK Carbamidomethyl (C22)	40.7
									NLTAPELVEWALKLEK	41.9							NLIGDDEHVWTDRGVFNIEGGCYAK Deamidated (N1)- Carbamidomethyl (C22)	6.0
									NLTAPELVEWALKLEKDSQLTAR	36.5							NLTAPELVEWALK	42.9
									NLTAPELVEWALKLEKDSQLTAR Deamidated (N1)	16.4							NLTAPELVEWALK Deamidated (N1)	38.6
									NMLIVPTLEELQSFGEPDYVIYNAGEAK	37.3							NLTAPELVEWALKLEK	50.1
									NMLIVPTLEELQSFGEPDYVIYNAGEAK Oxidation (M2)	46.7							NLTAPELVEWALKLEKDSQLTAR	48.2
									RNLIGDDEHVWTDRGVFNIEGGCYAK Carbamidomethyl (C23)	18.9							NLTAPELVEWALKLEKDSQLTAR Deamidated (N1)	25.0
									RTHEIDFNDESICKNTR Carbamidomethyl (C13)	49.4							NLTAPELVEWALKLEKDSQLTAR Deamidated (Q19)	11.7
									RVANVPASLLNPR	36.9							NMLIVPTLEELQSFGEPDYVIYNAGEAK	45.4
									RVANVPASLLNPR Deamidated (N11)	24.0							NMLIVPTLEELQSFGEPDYVIYNAGEAK Deamidated (Q12)	58.6
									SFQDRFASR	25.9							NMLIVPTLEELQSFGEPDYVIYNAGEAK Oxidation (M2)	31.8
									THEIDFNDESICK Carbamidomethyl (C12)	43.9							QFNETTKELVTMFQK	30.3
									THEIDFNDESICK Deamidated (N7)- Carbamidomethyl (C12)	34.4							QFNETTKELVTMFQK Deamidated (N3)- Oxidation (M12)	38.7
									THEIDFNDESICKNTR Carbamidomethyl (C12)	57.6							QFNETTKELVTMFQK Deamidated (Q1)	39.7
									TTLSADPRR	27.9							RNLIGDDEHVWTDR	31.1
									VANVPASLLNPR	46.0							RNLIGDDEHVWTDRGVFNIEGGCYAK Carbamidomethyl (C23)	20.3
									VANVPASLLNPR Deamidated (N10)	47.3							RTHEIDFNDESICKNTR Carbamidomethyl (C13)	40.0
									VANVPASLLNPR Deamidated (N3)	47.8							RVANVPASLLNPR	29.8
									VANVPASLLNPRK	33.6							RVANVPASLLNPR Deamidated (N11)	13.1
									VANVPASLLNPRK Deamidated (N3)	23.9							SFQDRFASR	23.1
									VARPIFSSCFGGPFLVR Carbamidomethyl (C9)	36.2							SGDVTVFFGLSGTGK	45.1
									VAYPLMHIDGALSK	52.7							THEIDFNDESICKNTR Carbamidomethyl (C12)	44.7
									VAYPLMHIDGALSK Oxidation (M6)	32.5							THEIDFNDESICKNTR Carbamidomethyl (C12)- Deamidated (N14)	45.8
									VWLLNTGYAGGR	64.3							THEIDFNDESICKNTR Deamidated (N7)- Carbamidomethyl (C12)	40.4
									VWLLNTGYAGGRADR	25.0							VANVPASLLNPR	43.9
									YVEASRL	30.6							VANVPASLLNPRK	6.1
																	VARPIFSSCFGGPFLVR Carbamidomethyl (C9)	35.4
																	VAYPLMHIDGALSK	45.2
																	VAYPLMHIDGALSK Oxidation (M6)	23.0
																	VWLLNTGYAGGR	49.3
																	VWLLNTGYAGGR Deamidated (N5)	45.6
7	Tb927.11.7460	glucose-regulated protein 78, putative,luminal binding protein 1 (BiP), putative	1.00	2234	147	46	37	59.4	AKFEELNMDLFKGTLVPVQR	55.9	1.00	1768	143	36	29	57.6	AKFEELNMDLFK	30.4
	Tb927.11.7510	glucose-regulated protein 78, putative,luminal binding protein 1 (BiP), putative							AKFEELNMDLFKGTLVPVQR Oxidation (M8)	37.6							AKFEELNMDLFKGTLVPVQR	37.7
									DAGTIAGLNVVR	78.4							AKFEELNMDLFKGTLVPVQR Oxidation (M8)	6.2
									DNRLLGKFELSGIPPAAR	29.5							DAGTIAGLNVVR	48.7
									FLDENPNAEKEEYKTALETLQSVTNPIIQK	37.7							ELNRGINPDEAVAYGAAVQAAVLTGESEVGGR	48.3
									FLDENPNAEKEEYKTALETLQSVTNPIIQK Deamidated (N7)	31.9							FLDENPNAEKEEYKTALETLQSVTNPIIQK	51.5
									GINPDEAVAYGAAVQAAVLTGESEVGGR	39.9							FLDENPNAEKEEYKTALETLQSVTNPIIQK Deamidated (N7)	25.4
									IINEPTAAAIAYGLNK	80.2							FLDENPNAEKEEYKTALETLQSVTNPIIQK Deamidated (Q21)	52.6
									IINEPTAAAIAYGLNK Deamidated (N3)	84.8							GINPDEAVAYGAAVQAAVLTGESEVGGR	48.5
									IINEPTAAAIAYGLNKAGEK	68.6							IINEPTAAAIAYGLNKAGEK	18.2
									IINEPTAAAIAYGLNKAGEK Deamidated (N3)	6.9							ITPSVVAFTDTER	48.4
									ITPSVVAFTDTER	62.7							ITPSVVAFTDTERLIGDGAK	36.3
									ITPSVVAFTDTERLIGDGAK	43.7							KKQFTPEEISAMVLQK	37.2
									ITPSVVAFTDTERLIGDGAKNQLPQNPHNTIYTIK	42.4							KYTDAAVQADKK	42.9
									ITPSVVAFTDTERLIGDGAKNQLPQNPHNTIYTIK Deamidated (N21)	30.3							LDPNDKAAVETAVAEAIR	40.1
									KYTDAAVQADKK	40.7							LGGKLDPNDKAAVETAVAEAIR	39.4
									LIERNTQIPTKK	25.9							LKKSDIHEIVLVGGSTR	53.8
									LIGDGAKNQLPQNPHNTIYTIKR	53.2							LLGKFELSGIPPAAR	37.8
									LLGKFELSGIPPAAR	39.7							LLSYEVIADRDGKPK	25.6
									LLSYEVIADRDGKPK	22.3							NQLPQNPHNTIYTIKR	23.3
									MKEIAETYLGEK Oxidation (M1)	21.9							NSLESVAYSLR	53.7
									NAVVTVPAYFNDAQR	43.5							NSLESVAYSLR Deamidated (N1)	66.4
									NAVVTVPAYFNDAQRQSTKDAGTIAGLNVVR	28.2							SDIHEIVLVGGSTR	51.2
									NQLPQNPHNTIYTIK	31.5							SQVFSTHADNQPGVLIQVYEGER	61.2
									NQLPQNPHNTIYTIKR	33.7							TYQSAGGGDKPQPMDDL Oxidation (M14)	32.2
									NSLESVAYSLR	60.0							VEVDSLTEGFDFSEK	19.2
									NSLESVAYSLR Deamidated (N1)	29.7							VEVDSLTEGFDFSEKITR	32.2
									NVDISKDQK	24.3							VKNAVVTVPAYFNDAQR	33.5
									QLSSHPEAR	17.9							VKNAVVTVPAYFNDAQR Deamidated (N13)	48.3
									QSTKDAGTIAGLNVVR	72.0							VLEDAKLKK	25.7
									QSTKDAGTIAGLNVVR Deamidated (Q1)	23.9							VPKVQQLISDFFGGKELNR	31.5
									SDIHEIVLVGGSTR	7.9							VQQLISDFFGGK	52.3
									SQVFSTHADNQPGVLIQVYEGER	41.6							VQQLISDFFGGKELNR	54.7
									SQVFSTHADNQPGVLIQVYEGERQLTK	55.8							VQQLISDFFGGKELNR Deamidated (Q3)	5.7
									SQVFSTHADNQPGVLIQVYEGERQLTKDNR	56.3							VVLVDVIPLSLGIETVGGVMTK	56.8
									TYQSAGGGDKPQPMDDL Oxidation (M14)	41.1							VVLVDVIPLSLGIETVGGVMTK Oxidation (M20)	39.5
									VEVDSLTEGFDFSEK	4.8								
									VKNAVVTVPAYFNDAQR	34.3								
									VKNAVVTVPAYFNDAQR Deamidated (Q16)	30.7								

									VLEDAK	17.1								
									VLEDAKLKK	28.6								
									VPKVQQLISDFFGGKELNR	41.2								
									VQQLISDFFGGK	45.5								
									VQQLISDFFGGKELNR	66.9								
									VVLVDVIPLSLGIETVGGVMTK	34.6								
									VVLVDVIPLSLGIETVGGVMTK Oxidation (M20)	27.7								
8	Tb927.10.5620	fructose-bisphosphate aldolase, glycosomal	1.00	2914	222	55	39	72.3	ADDDKDSQSLYVAGNTY	52.1	1.00	1548	94	32	21	50.5	ADDDKDSQSLYVAGNTY	37.6
									AKMNSLAQLGK Oxidation (M3)	36.4							ADDDKDSQSLYVAGNTY Deamidated (N15)	29.7
									AKMNSLAQLGK Oxidation (M3)- Deamidated (N4)	49.0							AKTGETFPQYLR	39.1
									AKMNSLAQLGKYNR Oxidation (M3)	61.3							AKTGETFPQYLR Deamidated (Q9)	3.6
									AKTGETFPQYLR	47.7							ALMLECEGFEQYISGVILHDETVYQK Carbamidomethyl (C6)	45.7
									AKTGETFPQYLR Deamidated (Q9)	5.2							ALMLECEGFEQYISGVILHDETVYQK Carbamidomethyl (C6)- Deamidated (Q11)	69.2
									AKTGETFPQYLRR	26.9							ALMLECEGFEQYISGVILHDETVYQK Oxidation (M3)- Carbamidomethyl (C6)	31.8
									ALMLECEGFEQYISGVILHDETVYQK Carbamidomethyl (C6)	52.4							ATAEQVAEYTVK	50.0
									ALMLECEGFEQYISGVILHDETVYQK Oxidation (M3)- Carbamidomethyl (C6)	52.6							AWGGKESGVEAGRR	23.5
									ALMLECEGFEQYISGVILHDETVYQK Oxidation (M3)- Carbamidomethyl (C6)- Deamidated (Q11)	16.8							FAGIGLSNTAEHR	57.7
									ALMLECEGFEQYISGVILHDETVYQKAK Carbamidomethyl (C6)	45.6							FAGIGLSNTAEHRR	20.9
									ALMLECEGFEQYISGVILHDETVYQKAK Oxidation (M3)- Carbamidomethyl (C6)	78.6							GLLAADESTGSCSK Carbamidomethyl (C12)	73.1
									ALQSSAIK	15.5							GLLAADESTGSCSKR Carbamidomethyl (C12)	59.7
									ALQSSAIKAWGGK	66.0							LKTPYEAELIETAK	12.9
									ALQSSAIKAWGGKESGVEAGR	60.8							LKTPYEAELIETAKK	45.3
									ATAEQVAEYTVK	53.7							RVEVLLTQLPAYNR	32.6
									ATAEQVAEYTVKTLAR	54.8							RVEVLLTQLPAYNR Deamidated (N13)	10.7
									ATAEQVAEYTVKTLAR Deamidated (Q5)	12.7							RVEVLLTQLPAYNR Deamidated (Q8)	32.8
									AWGGKESGVEAGR	36.4							RVEVLLTQLPAYNR Deamidated (Q8)- Deamidated (N13)	31.8
									AWGGKESGVEAGRR	11.9							RVEVLLTQLPAYNRLK	33.5
									FAGIGLSNTAEHR	51.6							RVEVLLTQLPAYNRLK Deamidated (N13)	21.0
									FAGIGLSNTAEHR Deamidated (N8)	49.4							TGETFPQYLR	58.7
									FAGIGLSNTAEHRR	26.0							TGETFPQYLR Deamidated (Q7)	40.7
									FAGIGLSNTAEHRR Deamidated (N8)	14.1							TPYEAELIETAK	50.9
									GLLAADESTGSCSKR Carbamidomethyl (C12)	70.3							TPYEAELIETAKK	35.7
									GVVPGIKTDCGLEPLVEGAK Carbamidomethyl (C10)	32.9							VEVLLTQLPAYNR	59.2
									GVVPGIKTDCGLEPLVEGAKGEQMTAGLDGYIK Carbamidomethyl (C10)- Oxidation (M24)	33.2							VEVLLTQLPAYNRLK	7.5
									KMTAPGKGLLAADESTGSCSK Oxidation (M2)- Carbamidomethyl (C19)	22.6							VSQHVWSEVVSALHR	53.5
									LKTPYEAELIETAK	41.8							YAILSQLCGLVPIVEPEVMIDGTHDIETCQR Carbamidomethyl (C8)- Carbamidomethyl (C29)	56.6
									LKTPYEAELIETAKK	34.4							YAILSQLCGLVPIVEPEVMIDGTHDIETCQR Carbamidomethyl (C8)- Carbamidomethyl (C29)- Deamidated (Q30)	42.1
									MNSLAQLGKYNR Oxidation (M1)	43.5							YNRADDDKDSQSLYVAGNTY	45.7
									MNSLAQLGKYNR Oxidation (M1)- Deamidated (Q6)	50.2							YNRADDDKDSQSLYVAGNTY Deamidated (N18)	38.4
									MTAPGKGLLAADESTGSCSK Carbamidomethyl (C18)	40.5								
									MTAPGKGLLAADESTGSCSK Oxidation (M1)- Carbamidomethyl (C18)	55.1								
									MTAPGKGLLAADESTGSCSKR Carbamidomethyl (C18)	64.0								
									MTAPGKGLLAADESTGSCSKR Oxidation (M1)- Carbamidomethyl (C18)	56.7								
									NVYKIQNGTVSEAVVR	69.9								
									NVYKIQNGTVSEAVVR Deamidated (N7)	66.1								
									RFAGIGLSNTAEHRR	34.5								
									RGVVPGIKTDCGLEPLVEGAK Carbamidomethyl (C11)	16.8								
									RVEVLLTQLPAYNR	43.5								
									RVEVLLTQLPAYNR Deamidated (N13)	20.2								
									RVEVLLTQLPAYNRLK	33.8								
									RVEVLLTQLPAYNRLK Deamidated (N13)	31.1								
									TDCGLEPLVEGAKGEQMTAGLDGYIKR Carbamidomethyl (C3)- Oxidation (M17)	35.8								
									TGETFPQYLR	46.6								
									TPYEAELIETAK	57.9								
									TPYEAELIETAKK	45.0								
									VEVLLTQLPAYNR	53.9								
									VEVLLTQLPAYNRLK	57.5								
									VSQHVWSEVVSALHR	45.2								
									VSQHVWSEVVSALHR Deamidated (Q3)	30.5								
									YAILSQLCGLVPIVEPEVMIDGTHDIETCQR Carbamidomethyl (C8)- Carbamidomethyl (C29)	45.1								
									YAILSQLCGLVPIVEPEVMIDGTHDIETCQR Carbamidomethyl (C8)- Carbamidomethyl (C29)- Oxidation (M19)	46.1								
									YNRADDDKDSQSLYVAGNTY	56.3								
9	Tb927.1.2340	alpha tubulin	1.00	2567	578	52	32	64.8	AVCMIANSTAIAEVFAR Carbamidomethyl (C3)	25.7	1.00	2475	518	49	29	64.3	AVCMIANSTAIAEVFAR Carbamidomethyl (C3)	26.7
	Tb927.1.2360	alpha tubulin							AVCMIANSTAIAEVFAR Carbamidomethyl (C3)- Oxidation (M4)	40.3							AVCMIANSTAIAEVFAR Carbamidomethyl (C3)- Deamidated (N7)	16.2
	Tb927.1.2380	alpha tubulin							AVCMIANSTAIAEVFAR Carbamidomethyl (C3)- Oxidation (M4)- Deamidated (N7)	23.5							AVCMIANSTAIAEVFAR Carbamidomethyl (C3)- Oxidation (M4)	66.8
	Tb927.1.2400	alpha tubulin							AVFLDLEPTVVDEVR	54.4							AVCMIANSTAIAEVFAR Carbamidomethyl (C3)- Oxidation (M4)- Deamidated (N7)	22.8
									AVFLDLEPTVVDEVRTGTYR	27.3							AVFLDLEPTVVDEVR	42.4
									AYHEQLSVSEISNAVFEPASMMTK Oxidation (M21)- Oxidation (M22)	17.2							AVFLDLEPTVVDEVRTGTYR	27.1
									CGINYQPPTVVPGGDLAK Carbamidomethyl (C1)	27.1							CGINYQPPTVVPGGDLAK Carbamidomethyl (C1)	34.2
									CGINYQPPTVVPGGDLAKVQR Carbamidomethyl (C1)	45.5							CGINYQPPTVVPGGDLAKVQR Carbamidomethyl (C1)	8.1
									DVNAAVATIK	33.2							DVNAAVATIK	23.7
									DVNAAVATIKTKR	18.6							EIVDLCLDR Carbamidomethyl (C6)	33.5
									DVNAAVATIKTKR Deamidated (N3)	34.1							EIVDLCLDRIR Carbamidomethyl (C6)	28.3
									EIVDLCLDR Carbamidomethyl (C6)	42.8							FDGALNVDLTEFQTNLVPYPR	45.0
									EIVDLCLDRIR Carbamidomethyl (C6)	29.4							FDGALNVDLTEFQTNLVPYPR Deamidated (N6)	24.0
									FDGALNVDLTEFQTNLVPYPR	55.3							GDVVPKDVNAAVATIK	31.5
									GHYTIGKEIVDLCLDR Carbamidomethyl (C13)	37.3							GHYTIGKEIVDLCLDR Carbamidomethyl (C13)	43.0
									GHYTIGKEIVDLCLDRIR Carbamidomethyl (C13)	27.3							GHYTIGKEIVDLCLDRIR Carbamidomethyl (C13)	31.1
									IDHKFDLMYSK Oxidation (M8)	19.8							IDHKFDLMYSKR	20.9
									IDHKFDLMYSKR	22.5							IHFVLTSYAPVISAEK	28.1
									IDHKFDLMYSKR Oxidation (M8)	23.6							IHFVLTSYAPVISAEKAYHEQLSVSEISNAVFEPASMMTK	27.0
									IHFVLTSYAPVISAEK	49.0							KLADNCTGLQGFLVYHAVGGGTGSGLGALLLER Carbamidomethyl (C6)	48.4
									IHFVLTSYAPVISAEKAYHEQLSVSEISNAVFEPASMMTK Oxidation (M37)- Oxidation (M38)	51.8							KLADNCTGLQGFLVYHAVGGGTGSGLGALLLER Carbamidomethyl (C6)- Deamidated (Q10)	33.9
									KLADNCTGLQGFLVYHAVGGGTGSGLGALLLER Carbamidomethyl (C6)	29.4							KLADNCTGLQGFLVYHAVGGGTGSGLGALLLER Deamidated (N5)- Carbamidomethyl (C6)	31.3
									KLADNCTGLQGFLVYHAVGGGTGSGLGALLLER Carbamidomethyl (C6)- Deamidated (Q10)	11.1							KLADNCTGLQGFLVYHAVGGGTGSGLGALLLER Deamidated (Q10)- Deamidated (N5)- Carbamidomethyl (C6)	18.9
									KLADNCTGLQGFLVYHAVGGGTGSGLGALLLER Deamidated (N5)- Carbamidomethyl (C6)	19.8							LADNCTGLQGFLVYHAVGGGTGSGLGALLLER Carbamidomethyl (C5)	51.1
									KLADNCTGLQGFLVYHAVGGGTGSGLGALLLER Deamidated (Q10)- Deamidated (N5)- Carbamidomethyl (C6)	5.3							LADNCTGLQGFLVYHAVGGGTGSGLGALLLER Carbamidomethyl (C5)- Deamidated (Q9)	55.2
									LADNCTGLQGFLVYHAVGGGTGSGLGALLLER Carbamidomethyl (C5)	65.0							LADNCTGLQGFLVYHAVGGGTGSGLGALLLER Deamidated (N4)- Carbamidomethyl (C5)	21.4
									LADNCTGLQGFLVYHAVGGGTGSGLGALLLER Carbamidomethyl (C5)- Deamidated (Q9)	32.7							LADNCTGLQGFLVYHAVGGGTGSGLGALLLER Deamidated (Q9)- Deamidated (N4)- Carbamidomethyl (C5)	18.7
									LADNCTGLQGFLVYHAVGGGTGSGLGALLLER Deamidated (N4)- Carbamidomethyl (C5)	39.3							LIGQVVSSLTASLR	44.5
									LIGQVVSSLTASLR	48.3							LIGQVVSSLTASLR Deamidated (Q4)	30.8
									LIGQVVSSLTASLR Deamidated (Q4)	33.6							LIGQVVSSLTASLRFDGALNVDLTEFQTNLVPYPR	17.0
									LIGQVVSSLTASLRFDGALNVDLTEFQTNLVPYPR	9.7							LSVDYGK	27.3
									LSVDYGK	27.3							LSVDYGKK	34.2
									LSVDYGKK	27.0							NLDIERPTYTNLNR	25.0
									LSVDYGKKSK	22.9							NLDIERPTYTNLNR Deamidated (N1)	1.3
									NLDIERPTYTNLNR	34.2							NLDIERPTYTNLNR Deamidated (N1)- Deamidated (N11)	17.2
									NLDIERPTYTNLNR Deamidated (N1)- Deamidated (N11)	7.8							NLDIERPTYTNLNR Deamidated (N11)	15.9
									QLFHPEQLISGKEDAANNYAR	40.2							QLFHPEQLISGK	19.2
									QLFHPEQLISGKEDAANNYAR Deamidated (N18)	35.5							QLFHPEQLISGKEDAANNYAR	39.1
									QLFHPEQLISGKEDAANNYAR Deamidated (Q1)	26.7							QLFHPEQLISGKEDAANNYAR Deamidated (N17)	30.0
									QLFHPEQLISGKEDAANNYAR Deamidated (Q1)- Deamidated (Q7)	37.4							QLFHPEQLISGKEDAANNYAR Deamidated (N18)	35.8
									QLFHPEQLISGKEDAANNYAR Deamidated (Q7)	28.0							QLFHPEQLISGKEDAANNYAR Deamidated (Q1)- Deamidated (N17)	38.0
									QLFHPEQLISGKEDAANNYAR Deamidated (Q7)- Deamidated (N17)	36.1							QLFHPEQLISGKEDAANNYAR Deamidated (Q7)	4.9
									RNLDIERPTYTNLNR	31.9							RNLDIERPTYTNLNR	31.1
									RNLDIERPTYTNLNR Deamidated (N2)	23.5							RNLDIERPTYTNLNR Deamidated (N2)	12.4
									RTIQFVDWSPTGFK	44.3							RTIQFVDWSPTGFK	17.4
									TGTYRQLFHPEQLISGKEDAANNYAR	38.6							RTIQFVDWSPTGFK Deamidated (Q4)	9.7
									TGTYRQLFHPEQLISGKEDAANNYAR Deamidated (N22)	55.0							TIGVEDDAFNTFFSETGAGK	75.7
									TGTYRQLFHPEQLISGKEDAANNYAR Deamidated (Q12)	37.9							TIQFVDWSPTGFK	54.7
									TIGVEDDAFNTFFSETGAGKHVPR	36.7							TIQFVDWSPTGFKCGINYQPPTVVPGGDLAK Carbamidomethyl (C14)	22.9
									TIQFVDWSPTGFK	42.8								
									TIQFVDWSPTGFK Deamidated (Q3)	35.7								
									TIQFVDWSPTGFKCGINYQPPTVVPGGDLAK Carbamidomethyl (C14)	39.5								

10	Tb927.6.4280	glyceraldehyde 3-phosphate dehydrogenase, glycosomal	1.00	2540	405	52	35	88.3	AAALNIIPSTTGAAK	34.4	1.00	2259	402	48	27	80.5	AAALNIIPSTTGAAK	35.8
	Tb927.6.4300	glyceraldehyde 3-phosphate dehydrogenase, glycosomal							AAALNIIPSTTGAAK Deamidated (N5)	25.8							AAALNIIPSTTGAAK Deamidated (N5)	37.6
									AAALNIIPSTTGAAKAVGMVIPSTQGK Oxidation (M19)	29.3							AQRNPADLPWGK	24.6
									AQRNPADLPWGK	39.2							AQRNPADLPWGKLGVEYVIESTGLFTVK	25.3
									AQRNPADLPWGKLGVEYVIESTGLFTVK	12.9							AQRNPADLPWGKLGVEYVIESTGLFTVK Deamidated (N4)	24.9
									AQRNPADLPWGKLGVEYVIESTGLFTVK Deamidated (N4)	7.6							AQRNPADLPWGKLGVEYVIESTGLFTVK Deamidated (Q2)	17.0
									AQRNPADLPWGKLGVEYVIESTGLFTVK Deamidated (Q2)	7.3							ATLQNNLPNER	41.4
									ATLQNNLPNER	31.5							ATLQNNLPNER Deamidated (N5)	52.5
									ATLQNNLPNER Deamidated (N5)	39.0							ATLQNNLPNER Deamidated (N6)	42.5
									ATLQNNLPNERR	25.0							ATLQNNLPNER Deamidated (N9)	35.4
									ATLQNNLPNERR Deamidated (N5)	18.7							ATLQNNLPNERR	18.3
									AVGMVIPSTQGK	35.4							ATLQNNLPNERR Deamidated (N5)	16.0
									AVGMVIPSTQGK Oxidation (M4)	37.3							AVGMVIPSTQGK	28.1
									AVGMVIPSTQGK Oxidation (M4)- Deamidated (Q10)	43.9							AVGMVIPSTQGK Oxidation (M4)	37.1
									DTSIKEIDAALK	45.5							DTSIKEIDAALK	44.6
									DTSIKEIDAALKR	46.4							DTSIKEIDAALKR	38.9
									EHHVVSNASCTTNCLAPLVHVLVK Carbamidomethyl (C10)- Carbamidomethyl (C14)	31.5							EGFGISTGLMTTVHSYTATQK	41.8
									EHHVVSNASCTTNCLAPLVHVLVK Carbamidomethyl (C10)- Carbamidomethyl (C14)- Deamidated (N13)	23.7							EHHVVSNASCTTNCLAPLVHVLVK Carbamidomethyl (C10)- Carbamidomethyl (C14)	46.0
									FKHSVSTTK	28.2							EHHVVSNASCTTNCLAPLVHVLVK Carbamidomethyl (C10)- Carbamidomethyl (C14)- Deamidated (N13)	33.9
									GGARKVVISAPASGGAK	44.1							EHHVVSNASCTTNCLAPLVHVLVK Deamidated (N7)- Carbamidomethyl (C10)- Carbamidomethyl (C14)	44.6
									GGRAAALNIIPSTTGAAK	45.1							IVSWYDNEWGYSHR	46.0
									ILCVKAQR Carbamidomethyl (C3)	25.3							IVSWYDNEWGYSHR Deamidated (N7)	45.4
									ILCVKAQRNPADLPWGK Carbamidomethyl (C3)	25.0							KVVISAPASGGAK	34.1
									IVSWYDNEWGYSHR	49.3							LGVEYVIESTGLFTVK	8.5
									IVSWYDNEWGYSHR Deamidated (N7)	19.9							LTGMAFRVPTADVSVVDLTFIATR	16.5
									IVSWYDNEWGYSHRVVDLVR	35.4							LTGMAFRVPTADVSVVDLTFIATR Oxidation (M4)	20.4
									KVVISAPASGGAK	43.4							MVFQALCDDGLLGNEIDVVAVVDMNTDAR Carbamidomethyl (C7)- Deamidated (N14)	9.8
									LGVEYVIESTGLFTVK	12.3							NILGYTDEELVSADFISDSR	43.8
									LTGMAFRVPTADVSVVDLTFIATR Oxidation (M4)	38.3							NILGYTDEELVSADFISDSR Deamidated (N1)	7.3
									MVFQALCDDGLLGNEIDVVAVVDMNTDAR Oxidation (M1)- Oxidation (M24)- Carbamidomethyl (C7)	16.4							NPADLPWGK	30.6
									NILGYTDEELVSADFISDSR	47.5							NPADLPWGKLGVEYVIESTGLFTVK	30.5
									SAAEGHLRGGAR	24.6							NPADLPWGKLGVEYVIESTGLFTVK Deamidated (N1)	45.7
									SKPSVAKDDTLVVNGHR	70.9							SKPSVAKDDTLVVNGHR	21.0
									SKPSVAKDDTLVVNGHR Deamidated (N14)	48.3							SKPSVAKDDTLVVNGHR Deamidated (N14)	45.4
									SKPSVAKDDTLVVNGHRILCVK Deamidated (N14)- Carbamidomethyl (C20)	22.3							SSIYDSKATLQNNLPNER	57.1
									SSIYDSK	30.7							SSIYDSKATLQNNLPNER Deamidated (N12)	4.0
									SSIYDSKATLQNNLPNER	75.3							SSIYDSKATLQNNLPNER Deamidated (N16)	42.0
									SSIYDSKATLQNNLPNER Deamidated (N12)	51.7							SSIYDSKATLQNNLPNER Deamidated (Q11)	44.4
									SSIYDSKATLQNNLPNER Deamidated (N13)	76.7							SSIYDSKATLQNNLPNERR	31.4
									SSIYDSKATLQNNLPNERR	41.0							SSIYDSKATLQNNLPNERR Deamidated (N12)	31.8
									SSIYDSKATLQNNLPNERR Deamidated (N12)	45.3							SSIYDSKATLQNNLPNERR Deamidated (N13)	35.1
									SSIYDSKATLQNNLPNERR Deamidated (N13)	71.5							SSIYDSKATLQNNLPNERR Deamidated (Q11)	51.2
									TFVMGVNHNDYNPR	44.9							TIKVGINGFGR Deamidated (N7)	43.5
									TFVMGVNHNDYNPR Oxidation (M4)	32.9							TVDGVSVKDWR	38.8
									TFVMGVNHNDYNPR Oxidation (M4)- Deamidated (N7)	33.5							TYMKNILGYTDEELVSADFISDSR Oxidation (M3)	35.9
									TIKVGINGFGR	22.0							VPTADVSVVDLTFIATR	54.6
									TIKVGINGFGR Deamidated (N7)	48.6							VVISAPASGGAK	36.3
									TVDGVSVKDWR	31.7							YFAYQMKYDSVHGK Oxidation (M6)	5.8
									TVDGVSVKDWRGGR	23.3								
									TYMKNILGYTDEELVSADFISDSR Oxidation (M3)	25.1								
									VVISAPASGGAK	40.6								
									YFAYQMKYDSVHGK Oxidation (M6)	22.6								
11	Tb927.8.3530	glycerol-3-phosphate dehydrogenase [NAD], glycosomal	1.00	2050	283	39	31	86.7	AVAEGVATAEPLER	40.6	1.00	2514	300	46	25	83.6	AALISR	15.2
									AVAEGVATAEPLERLAK	51.9							AVAEGVATAEPLER	32.8
									CWATTDVIGCEIASAMKNVLAIASGALKGLGTENNAR Carbamidomethyl (C1)- Carbamidomethyl (C10)- Deamidated (N35)	10.3							CWATTDVIGCEIASAMK Carbamidomethyl (C1)- Carbamidomethyl (C10)	81.8
									GAEIVLFVIPTQFLR	32.6							CWATTDVIGCEIASAMKNVLAIASGALK Carbamidomethyl (C1)- Carbamidomethyl (C10)	41.8
									GAVFGSGAFGTALACVLAK Carbamidomethyl (C15)	37.9							CWATTDVIGCEIASAMKNVLAIASGALKGLGTENNAR Carbamidomethyl (C1)- Carbamidomethyl (C10)	68.5
									GAVFGSGAFGTALACVLAKK Carbamidomethyl (C15)	55.8							GAEIVLFVIPTQFLR	47.0
									GFLQKNSHILR Deamidated (N6)	41.6							GAEIVLFVIPTQFLR Deamidated (Q12)	25.5
									GIERSSLLFPAQILEEFLPNYPIGVIAGPSFAIEVAK	28.3							GAVFGSGAFGTALACVLAK Carbamidomethyl (C15)	59.0
									GLLEIRDLTLALGGTGEAVFGLPGLGDLLLTCSSELSR Carbamidomethyl (C32)	17.6							GAVFGSGAFGTALACVLAKK Carbamidomethyl (C15)	35.3
									GMLTNVCTAAADINMAR Oxidation (M2)- Oxidation (M15)- Carbamidomethyl (C7)	49.1							GIERSSLLFPAQILEEFLPNYPIGVIAGPSFAIEVAK	33.6
									IMTTSDGSFR Oxidation (M2)	20.6							GIERSSLLFPAQILEEFLPNYPIGVIAGPSFAIEVAK Deamidated (N20)	35.5
									KCESVSVWHMNANEAR Carbamidomethyl (C2)- Oxidation (M10)	33.1							GLLEIRDLTLALGGTGEAVFGLPGLGDLLLTCSSELSR Carbamidomethyl (C32)	30.8
									KHNVHLPICHEVYNVLYANGCAK Carbamidomethyl (C9)- Carbamidomethyl (C21)	30.2							GMLTNVCTAAADINMAR Carbamidomethyl (C7)	56.0
									KHNVHLPICHEVYNVLYANGCAK Carbamidomethyl (C9)- Carbamidomethyl (C21)- Deamidated (N14)	21.0							GMLTNVCTAAADINMAR Carbamidomethyl (C7)- Deamidated (N14)	58.4
									KHNVHLPICHEVYNVLYANGCAK Carbamidomethyl (C9)- Carbamidomethyl (C21)- Deamidated (N19)	26.6							GMLTNVCTAAADINMAR Deamidated (N5)- Carbamidomethyl (C7)	70.4
									KHNVHLPICHEVYNVLYANGCAK Deamidated (N3)- Deamidated (N19)- Carbamidomethyl (C9)- Carbamidomethyl (C21)	15.7							GMLTNVCTAAADINMAR Oxidation (M2)- Deamidated (N5)- Carbamidomethyl (C7)	9.0
									KHNVHLPICHEVYNVLYANGCAKR Carbamidomethyl (C9)- Carbamidomethyl (C21)- Deamidated (N19)	11.7							GMLTNVCTAAADINMAR Oxidation (M2)- Oxidation (M15)- Carbamidomethyl (C7)	10.2
									KLNSCKLADEGLPALPR Carbamidomethyl (C5)	66.4							KCESVSVWHMNANEAR Carbamidomethyl (C2)- Deamidated (N11)	63.4
									KLNSCKLADEGLPALPR Deamidated (N3)- Carbamidomethyl (C5)	86.4							KHNVHLPICHEVYNVLYANGCAK Carbamidomethyl (C9)- Carbamidomethyl (C21)	35.6
									LADEGLPALPR	42.0							KHNVHLPICHEVYNVLYANGCAK Carbamidomethyl (C9)- Carbamidomethyl (C21)- Deamidated (N14)	17.1
									LAKKHNVHLPICHEVYNVLYANGCAK Carbamidomethyl (C12)- Carbamidomethyl (C24)- Deamidated (N22)	11.4							KHNVHLPICHEVYNVLYANGCAK Carbamidomethyl (C9)- Carbamidomethyl (C21)- Deamidated (N19)	18.8
									LGQGISLEEIKR	45.7							KHNVHLPICHEVYNVLYANGCAK Deamidated (N3)- Carbamidomethyl (C9)- Carbamidomethyl (C21)	26.0
									LGQGISLEEIKR Deamidated (Q3)	60.3							KHNVHLPICHEVYNVLYANGCAK Deamidated (N3)- Deamidated (N19)- Carbamidomethyl (C9)- Carbamidomethyl (C21)	42.5
									LGQGISLEEIKRTSK Deamidated (Q3)	29.7							KHNVHLPICHEVYNVLYANGCAKR Carbamidomethyl (C9)- Carbamidomethyl (C21)- Deamidated (N14)	21.6
									LNSCKLADEGLPALPR Carbamidomethyl (C4)	46.5							KHNVHLPICHEVYNVLYANGCAKR Carbamidomethyl (C9)- Carbamidomethyl (C21)- Deamidated (N19)	25.3
									NFTVGMKLGQGISLEEIKR Oxidation (M6)	8.7							KLNSCKLADEGLPALPR Carbamidomethyl (C5)	42.7
									NHVVSR	24.0							KLNSCKLADEGLPALPR Deamidated (N3)- Carbamidomethyl (C5)	49.8
									NVLAIASGALK	51.0							LADEGLPALPR	43.4
									NVLAIASGALKGLGTENNAR	66.9							LGQGISLEEIKR	34.4
									NVLAIASGALKGLGTENNAR Deamidated (N17)	84.1							LGQGISLEEIKR Deamidated (Q3)	47.2
									NVPVVMCSKGIER Carbamidomethyl (C7)	32.1							LNSCKLADEGLPALPR Carbamidomethyl (C4)	74.8
									NVPVVMCSKGIER Oxidation (M6)- Carbamidomethyl (C7)	38.7							LNSCKLADEGLPALPR Deamidated (N2)- Carbamidomethyl (C4)	13.4
									RGAVFGSGAFGTALACVLAK Carbamidomethyl (C16)	37.4							NVLAIASGALK	34.7
									SFKKLNSCK Carbamidomethyl (C8)	18.7							NVLAIASGALKGLGTENNAR	24.2
									SSLLFPAQILEEFLPNYPIGVIAGPSFAIEVAK	24.5							NVLAIASGALKGLGTENNAR Deamidated (N1)	37.5
									SSLLFPAQILEEFLPNYPIGVIAGPSFAIEVAK Deamidated (N16)	26.9							NVLAIASGALKGLGTENNAR Deamidated (N1)- Deamidated (N17)- Deamidated (N18)	49.5
									TSKAVAEGVATAEPLER	55.6							NVLAIASGALKGLGTENNAR Deamidated (N17)	43.9
									TSKAVAEGVATAEPLERLAK	49.6							NVLAIASGALKGLGTENNAR Deamidated (N18)	83.4
									VSGVTYLKR	55.3							RGAVFGSGAFGTALACVLAK Carbamidomethyl (C16)	50.3
																	SSLLFPAQILEEFLPNYPIGVIAGPSFAIEVAK	28.6
																	SSLLFPAQILEEFLPNYPIGVIAGPSFAIEVAK Deamidated (N16)	13.8
																	SSLLFPAQILEEFLPNYPIGVIAGPSFAIEVAK Deamidated (Q8)- Deamidated (N16)	38.2
																	TSKAVAEGVATAEPLER	29.0
																	VSGVTYLKR	53.2
																	VVNQKHENVYFLPGAPLPANLTFTADAEECAK Carbamidomethyl (C30)	44.8
																	VVNQKHENVYFLPGAPLPANLTFTADAEECAK Deamidated (Q4)- Carbamidomethyl (C30)	33.9
12	Tb927.11.11330	heat shock protein 70	1.00	1306	63	28	21	39.9	ARFEELCGDLFR Carbamidomethyl (C7)	37.4	1.00	1083	55	25	21	48.8	ARFEELCGDLFR Carbamidomethyl (C7)	43.5
									ARFEELCGDLFRGTLQPVER Carbamidomethyl (C7)	22.1							ATNGDTHLGGEDFDNRLVAHFTEEFKR Deamidated (N3)	29.9
									AVHDVVLVGGSTR	51.5							AVHDVVLVGGSTR	59.8
									AVVTVPAYFNDSQR	28.1							AVVTVPAYFNDSQR	33.2
									AVVTVPAYFNDSQR Deamidated (Q13)	19.6							AVVTVPAYFNDSQR Deamidated (Q13)	32.3
									AVVTVPAYFNDSQRQATKDAGTIAGLEVLR Deamidated (N10)	28.9							DAGTIAGLEVLR	64.6
									DAGTIAGLEVLR	74.8							DCHLLGTFDLSGIPPAPR Carbamidomethyl (C2)	16.4
									IINEPTAAAIAYGLDKADEGKER	31.7							IINEPTAAAIAYGLDKADEGKER	54.0
									IINEPTAAAIAYGLDKADEGKER Deamidated (N3)	37.8							LDDADKNAVTTAVEEALR	38.1
									LIGDAAKNQVAMNPTNTVFDAKR Oxidation (M12)	46.0							LIGDAAKNQVAMNPTNTVFDAKR Oxidation (M12)	11.2
									LSKADIER	40.7							MVSDAAKYEAEDKAQR	24.4
									MKEVAESYLGK Oxidation (M1)	23.1							NKGKDLSSNLR	7.3
									NKGKDLSSNLR	27.8							NQIVITNDKGR	14.1
									NQIVITNDKGR	44.9							NTINDPNVAGKLDDADKNAVTTAVEEALR	61.4
									NQIVITNDKGR Deamidated (N1)- Deamidated (N7)	44.9							NTINDPNVAGKLDDADKNAVTTAVEEALR Deamidated (N1)- Deamidated (N18)	55.0

#	Accession	Description							Peptide	Score
									NQIVITNDKGR Deamidated (N7)	32.6
									NQVAMNPTNTVFDAKR Oxidation (M5)	32.2
									NTINDPNVAGKLDDADKNAVTTAVEEALR	66.6
									QATKDAGTIAGLEVLR	66.2
									SINPDEAVAYGAAVQAFILTGGK	32.3
									TFNPEEISSMVLLK Oxidation (M10)	16.0
									TTPSYVAFTDSER	48.1
									VMQLVSDFFGGKELNK	65.3
									VMQLVSDFFGGKELNK Oxidation (M2)	29.7
									WLNDNQEASLDEYNHR	62.1
									WLNDNQEASLDEYNHR Deamidated (Q6)	39.4
									WLNDNQEASLDEYNHRQKELEGVCAPILSK Carbamidomethyl (C24)	37.1
									WLNDNQEASLDEYNHRQKELEGVCAPILSK Deamidated (N3)- Carbamidomethyl (C24)	28.8
13	Tb927.11.9980	2-oxoglutarate dehydrogenase E1 component, putative	1.00	1560	116	41	33	44.0	AFQCPIFHVNGDCPEDVAR Carbamidomethyl (C4)- Carbamidomethyl (C13)	57.0
									AFQCPIFHVNGDCPEDVAR Carbamidomethyl (C4)- Carbamidomethyl (C13)- Deamidated (N10)	15.5
									AFQCPIFHVNGDCPEDVAR Deamidated (Q3)- Carbamidomethyl (C4)- Carbamidomethyl (C13)	39.4
									AISAAPSTAYK	24.5
									AISAAPSTAYKSTHDAEERYICECVFS Carbamidomethyl (C22)- Carbamidomethyl (C24)	23.6
									AMTIKELHDHLTK	21.3
									AMTIKELHDHLTK Oxidation (M2)	27.5
									DLQSFERPLLTSPIR	40.9
									ESLQPVIDSLK	25.3
									FGCDGAESLIVGLR Carbamidomethyl (C3)	41.8
									FLQAVAEDVDTPAYSPEER	79.9
									GMKYSDELGEVTLEPTAVAR Oxidation (M2)	57.2
									GRLNTLYNVIGK Deamidated (N4)	9.1
									IEELSPFPVAEVQK	27.8
									LKAVLEQR	35.9
									LLYENDSFLSGTSAR	60.1
									LLYENDSFLSGTSAR Deamidated (N5)	56.1
									LMFTELLANPSHLEAVNPVLQGYTR	26.5
									LTLMIQAFEDYGHLAAR	5.1
									LVEEGVVTKEQQTAK	18.6
									LVMCTGQIYHILNR Oxidation (M3)- Carbamidomethyl (C4)	32.3
									MRNGKLMFTELLANPSHLEAVNPVLQGYTR Oxidation (M1)- Oxidation (M7)- Deamidated (N3)	18.1
									NGKLMFTELLANPSHLEAVNPVLQGYTR	14.1
									NGKLMFTELLANPSHLEAVNPVLQGYTR Deamidated (N1)	5.8
									NGKLMFTELLANPSHLEAVNPVLQGYTR Oxidation (M5)	48.0
									NQGAFYHVESR	30.8
									RVHATPDVFAR	28.1
									SFPVILKEFVGITAPELEPFKVQSDVK	20.5
									SSPYCSDLGR Carbamidomethyl (C5)	37.0
									STHDAEERYICECVFS Carbamidomethyl (C11)- Carbamidomethyl (C13)	7.5
									TDPLDAEDDSLHRSPQVKPR	58.8
									VGFMDQLGGAMSSSSR Oxidation (M4)- Oxidation (M11)	36.3
									VGFMDQLGGAMSSSSR Oxidation (M4)- Oxidation (M11)- Deamidated (Q6)	13.1
									VHATPDVFAR	41.0
									VPEGFVVNSK	19.7
									VQSLEKGEKIDWGAAEALAFGSLLLEGTHVR	17.9
									VQSLEKGEKIDWGAAEALAFGSLLLEGTHVR Deamidated (Q2)	23.5
									VVPASAEDKMEVAQSQDICAR Oxidation (M10)- Carbamidomethyl (C19)	25.0
									YGHNENDDPTITQPLLYKR	35.8
									YSDELGEVTLEPTAVAR	41.1
									YSEKLVEEGVVTKEQQTAK	51.0
14	Tb927.10.15410	glycosomal malate dehydrogenase	1.00	1708	163	35	24	68.4	AGRGSATLSMAEAGAR	34.0
									AGRGSATLSMAEAGAR Oxidation (M10)	24.4
									ANIEKGMNFAR	28.2
									ANIEKGMNFAR Oxidation (M7)	17.0
									ANIEKGMNFAR Oxidation (M7)- Deamidated (N8)	23.9
									FINNARHPLAVASVPVVGGHSDVTIVPLFSQLPGPLPEEGELTQIR	50.0
									GSATLSMAEAGAR Oxidation (M7)	37.4
									GVDVFVIVAGVPR	30.1
									HPLAVASVPVVGGHSDVTIVPLFSQLPGPLPEEGELTQIR	67.2
									HPLAVASVPVVGGHSDVTIVPLFSQLPGPLPEEGELTQIRK	34.2
									KRVQVAGTEVVK	24.4
									LGIYNKNKLLGVTLLDGLR	33.1
									LGIYNKNKLLGVTLLDGLR Deamidated (N7)	33.4
									LLGVTLLDGLR	78.7
									LLGVTLLDGLRATR	43.6
									LPIGPMNAVEKEMLKQSISVVK Oxidation (M6)- Oxidation (M13)	31.4
									LPIGPMNAVEKEMLKQSISVVK Oxidation (M6)- Oxidation (M13)- Deamidated (N7)	19.7
									LPPVKRDPALAELAR	15.4
									NKLLGVTLLDGLR	70.0
									NKLLGVTLLDGLR Deamidated (N1)	50.5
									QLPYGSTLSLYDVAGAPGVAADLSHVDR	76.8
									QLPYGSTLSLYDVAGAPGVAADLSHVDR Deamidated (Q1)	60.3
									QLPYGSTLSLYDVAGAPGVAADLSHVDRAGVTVK	71.4
									QLPYGSTLSLYDVAGAPGVAADLSHVDRAGVTVK Deamidated (Q1)	56.1
									QSISVVKANIEK	49.5
									QSISVVKANIEKGMNFAR Deamidated (Q1)- Oxidation (M14)	30.4
									QSISVVKANIEKGMNFAR Oxidation (M14)	48.7
									RLPIGPMNAVEKEMLK Oxidation (M14)	6.2
									RLPIGPMNAVEKEMLK Oxidation (M7)	33.2
									RVQVAGTEVVK	33.6
									VQVAGTEVVK	30.5
									VQVAGTEVVKAK	22.7
									VSVAVIGAAGGIGQSLSLLLLR	22.8
									VSVAVIGAAGGIGQSLSLLLLR Deamidated (Q14)	27.9
									YAAGKLPPVKR	23.6
15	Tb927.9.12570	glycerol kinase, glycosomal	1.00	1499	102	30	24	55.1	AALQAIALQLNDVVGSMKR	62.6
									AALQAIALQLNDVVGSMKR Deamidated (N11)	51.4
									AALQAIALQLNDVVGSMKR Oxidation (M17)	53.3
									DAGLNLSSLRVDGGLSK	53.6
									FIIFDER	22.9
									GTIVGMTLKTTR Oxidation (M6)	41.3
									KIEAIGITNQR	51.1
									KIEAIGITNQR Deamidated (Q10)- Deamidated (N9)	4.7
									KVTAELGGGDSMFASK Oxidation (M12)	36.2
									KWSPELCEK Carbamidomethyl (C7)	36.0
									LKIPMETLPEIR	43.3
									LKIPMETLPEIR Oxidation (M5)	36.0
									LRQKDASFR	22.9
									LSGGKAFVTDVTNASR	66.7
									MKYVGSIDQGTTSTR	60.9
									MKYVGSIDQGTTSTR Oxidation (M1)	67.1
									NMNLFSHITECEK Oxidation (M2)- Carbamidomethyl (C11)	54.7
									NMNLFSHITECEKLAR Oxidation (M2)- Carbamidomethyl (C11)	38.9
									NTYGTGCFLLMNVGEEAR Carbamidomethyl (C7)- Oxidation (M11)	49.6
									SNSELFGYVETDECGVAAALNER Deamidated (N2)- Carbamidomethyl (C14)	7.0
									SVPGTQGIVFVPAFSGLLAPYWDPSAR	26.0

#	Accession							Peptide	Score
								NTINDPNVAGKLDDADKNAVTTAVEEALR Deamidated (N18)	14.9
								QATKDAGTIAGLEVLR	20.1
								QKELEGVCAPILSK Carbamidomethyl (C8)	33.5
								QTEGLLLLDVAPLTLGIETAGGVMTALIKR	27.0
								SINPDEAVAYGAAVQAFILTGGK	37.4
								SKQTEGLLLLDVAPLTLGIETAGGVMTALIKR	62.3
								SQIFSTYSDNQPGVHIQVFEGER	44.3
								SQIFSTYSDNQPGVHIQVFEGER Deamidated (Q2)	44.6
								VMQLVSDFFGGKELNK	48.7
								WLNDNQEASLDEYNHR	34.6
13	Tb927.11.9980	1.00	1144	67	26	24	35.4	AFQCPIFHVNGDCPEDVAR Carbamidomethyl (C4)- Carbamidomethyl (C13)	27.7
								AISAAPSTAYK	33.6
								AISAAPSTAYKSTHDAEER	13.6
								DLQSFERPLLTSPIR	57.1
								DLQSFERPLLTSPIR Deamidated (Q3)	17.1
								FLQAVAEDVDTPAYSPEER	65.2
								GRLNTLYNVIGK	31.3
								GRLNTLYNVIGK Deamidated (N4)	26.8
								HAVLHDQNKVDTYTPLAHIR	43.9
								IDWGAAEALAFGSLLLEGTHVR	45.4
								IEELSPFPVAEVQK	43.3
								LMFTELLANPSHLEAVNPVLQGYTR	32.9
								LTLMIQAFEDYGHLAAR	48.9
								NGKLMFTELLANPSHLEAVNPVLQGYTR	41.5
								NQGAFYHVESR	39.3
								RVHATPDVFAR	28.6
								SFPVILKEFVGITAPELEPFK	17.3
								SFPVILKEFVGITAPELEPFKVQSDVK	43.4
								TDPLDAEDDSLHRSPQVKPR	14.7
								TPNESSMEQITSGGFQPVIGDPSVPPEKAR	10.0
								VHATPDVFAR	31.1
								VQSLEKGEKIDWGAAEALAFGSLLLEGTHVR	49.7
								YGHNENDDPTITQPLLYKR	35.1
								YQSEVGNIR	37.0
								YSDELGEVTLEPTAVAR	33.4
								YSEKLVEEGVVTKEQQTAK	29.8
14	Tb927.10.15410	1.00	1979	315	38	23	78.0	AGRGSATLSMAEAGAR	19.5
								AGRGSATLSMAEAGAR Oxidation (M10)	3.6
								ANIEKGMNFAR	23.7
								ANIEKGMNFAR Oxidation (M7)	15.8
								FAMFVVNAITGQSSPMVYAYVDTDGTQNCSFLAIPVVLGK Carbamidomethyl (C29)	21.1
								FINNARHPLAVASVPVVGGHSDVTIVPLFSQLPGPLPEEGELTQIR	49.7
								FINNARHPLAVASVPVVGGHSDVTIVPLFSQLPGPLPEEGELTQIR Deamidated (N4)	27.7
								FINNARHPLAVASVPVVGGHSDVTIVPLFSQLPGPLPEEGELTQIR Deamidated (Q31)	66.3
								GVDVFVIVAGVPR	42.5
								HPLAVASVPVVGGHSDVTIVPLFSQLPGPLPEEGELTQIR	68.1
								HPLAVASVPVVGGHSDVTIVPLFSQLPGPLPEEGELTQIR Deamidated (Q25)	13.8
								HPLAVASVPVVGGHSDVTIVPLFSQLPGPLPEEGELTQIR Deamidated (Q38)	74.8
								HPLAVASVPVVGGHSDVTIVPLFSQLPGPLPEEGELTQIRK	62.8
								KRVQVAGTEVVK	36.8
								LGIYNKNKLLGVTLLDGLR	39.9
								LGIYNKNKLLGVTLLDGLR Deamidated (N5)	18.7
								LLGVTLLDGLR	50.6
								LLGVTLLDGLRATR	41.1
								LPIGPMNAVEKEMLK	35.6
								LPIGPMNAVEKEMLK Deamidated (N7)	38.6
								LPIGPMNAVEKEMLKQSISVVK	34.2
								LPIGPMNAVEKEMLKQSISVVK Oxidation (M6)- Oxidation (M13)	16.4
								NKLLGVTLLDGLR	73.9
								NKLLGVTLLDGLR Deamidated (N1)	58.7
								QLPYGSTLSLYDVAGAPGVAADLSHVDR	64.2
								QLPYGSTLSLYDVAGAPGVAADLSHVDR Deamidated (Q1)	48.8
								QLPYGSTLSLYDVAGAPGVAADLSHVDRAGVTVK	75.8
								QLPYGSTLSLYDVAGAPGVAADLSHVDRAGVTVK Deamidated (Q1)	72.5
								QSISVVKANIEK	28.0
								RLPIGPMNAVEKEMLK	42.0
								RLPIGPMNAVEKEMLK Deamidated (N8)	33.0
								RLPIGPMNAVEKEMLK Oxidation (M7)	8.7
								RVQVAGTEVVK	53.2
								VQVAGTEVVK	25.1
								VQVAGTEVVKAK	17.4
								VSVAVIGAAGGIGQSLSLLLLR	45.8
								VSVAVIGAAGGIGQSLSLLLLR Deamidated (Q14)	26.7
								YAAGKLPPVKR	15.2
15	Tb927.9.12570	1.00	1142	81	22	17	49.2	AALQAIALQLNDVVGSMKR	39.5
								AALQAIALQLNDVVGSMKR Deamidated (N11)	51.4
								AALQAIALQLNDVVGSMKR Oxidation (M17)	17.6
								AFVTDVTNASR	65.2
								ETTVAWDRVTKEPLCYAPVWNDLR Carbamidomethyl (C15)	23.0
								FIIFDER	38.3
								HGLLSTVGFQVGR	32.3
								ITGLPVSTYFAAFK	38.1
								KIEAIGITNQR	61.9
								KWSPELCEK Carbamidomethyl (C7)	32.7
								LKIPMETLPEIR	36.1
								LKIPMETLPEIR Oxidation (M5)	16.0
								NTYGTGCFLLMNVGEEAR Carbamidomethyl (C7)	39.5
								NTYGTGCFLLMNVGEEAR Carbamidomethyl (C7)- Oxidation (M11)	54.7
								RGTLCFGTIDTWLMYK Carbamidomethyl (C5)	31.3
								SNSELFGYVETDECGVAAALNER Carbamidomethyl (C14)	50.7
								SNSELFGYVETDECGVAAALNER Deamidated (N2)- Carbamidomethyl (C14)	81.2
								SVPGTQGIVFVPAFSGLLAPYWDPSAR	23.7
								SVPGTQGIVFVPAFSGLLAPYWDPSARGTIVGMTLK	14.8
								TPIMGSIGDQQSALFGNMCFEKGEAK Carbamidomethyl (C19)	51.7
								VTKEPLCYAPVWNDLR Carbamidomethyl (C7)	47.6

									SVPGTQGIVFVPAFSGLLAPYWDPSARGTIVGMTLK	10.5						YVGSIDQGTTSTR	49.7
									SVPGTQGIVFVPAFSGLLAPYWDPSARGTIVGMTLK Oxidation (M33)	31.4								
									TPIMGSIGDQQSALFGNMCFEKGEAK Oxidation (M4)- Oxidation (M18)- Carbamidomethyl (C19)	48.7								
									TRKWSPELCEK Carbamidomethyl (C9)	28.7								
									TVSPSGSAMER Oxidation (M9)	37.9								
									VTAELGGGDSMFASK Oxidation (M11)	64.1								
									VTKEPLCYAPVWNDLR Carbamidomethyl (C7)	36.8								
									WSPELCEKLKIPMETLPEIR Carbamidomethyl (C6)- Oxidation (M13)	26.6								
									YVGSIDQGTTSTR	80.8								
16	Tb927.5.930	NADH-dependent fumarate reductase	1.00	1269	125	33	28	34.2	AKEVADEVLR	31.6	1.00	1367	78	31	27	32.8	AKEVADEVLR	43.8
									ALLQDSPLHTTMQYATSGLELTVPYALK	15.4						ALLQDSPLHTTMQYATSGLELTVPYALK	23.8
									ALREIALGK	24.7						ALREIALGK	42.7
									ALREIALGKER	26.0						ALREIALGKER	24.7
									APLSHSNPILGLFGAGEVTGGVHGGNR	10.7						ASIVDGGKYFER	29.2
									APLSHSNPILGLFGAGEVTGGVHGGNR Deamidated (N7)	14.8						ASIVDGGKYFERDTYK	29.5
									ASIVDGGKYFER	25.4						ATSGINGWGTR Deamidated (N6)	32.7
									ASIVDGGKYFERDTYK	23.9						DLAALIGCPVESVQQTLEEYER Carbamidomethyl (C8)	45.1
									ATSGINGWGTR	44.4						FLGPEALR	34.6
									ATSGINGWGTR Deamidated (N6)	42.2						FNLPGALQR	42.4
									ATSGINGWGTRAQAK Deamidated (N6)	40.7						FVNELDLR	25.5
									EHAPHLVNFPTTNGPWATGDGVK	20.4						GILTNHVQPPSDNLLVAICGPPVMQR Carbamidomethyl (C19)	48.2
									FVNELDLR	39.4						GYIVDYVIDNINAAGFQNVFFDWGGDCR Carbamidomethyl (C27)	41.3
									FVNELDLRSVVSK	33.7						KHEHASLDLGGVSK	38.3
									GILTNHVQPPSDNLLVAICGPPVMQR Carbamidomethyl (C19)- Oxidation (M24)- Deamidated (Q25)	34.1						LGAQLVDMDKVQLHPTGLINPKDPANPTK	39.0
									GSGGVLLNKQGKR	23.1						LGGNSLLECVVFGR Carbamidomethyl (C9)	55.8
									ISNTLPAR	19.5						LGGNSLLECVVFGR Deamidated (N4)- Carbamidomethyl (C9)	45.7
									LGGNSLLECVVFGR Carbamidomethyl (C9)	43.2						RFVNELDLR	29.3
									RISNTLPAR	23.9						RISNTLPAR	30.1
									SADAIGWLTSLGVPLTVLSQLGGHSR	47.8						SADAIGWLTSLGVPLTVLSQLGGHSR	56.4
									SADAIGWLTSLGVPLTVLSQLGGHSR Deamidated (Q20)	51.1						SADAIGWLTSLGVPLTVLSQLGGHSR Deamidated (Q20)	18.5
									SGIGGNTDPALVK	29.5						SGIGGNTDPALVK	52.6
									TFVLNRPPPLWTDGVGFIDR	7.4						SGLSLGQFIAIR	49.7
									TLGYNMNLVR Oxidation (M6)	29.1						TFVLNRPPPLWTDGVGFIDR	21.9
									TLSMKSADAIGWLTSLGVPLTVLSQLGGHSR Oxidation (M4)	19.2						VIVVGGGLAGLSAAIEAAGCGAQVVLMEK Carbamidomethyl (C20)	18.3
									VIVVGGGLAGLSAAIEAAGCGAQVVLMEKEAK Carbamidomethyl (C20)	23.5						VIVVGGGLAGLSAAIEAAGCGAQVVLMEKEAK Carbamidomethyl (C20)	43.8
									VIVVGGGLAGLSAAIEAAGCGAQVVLMEKEAK Carbamidomethyl (C20)- Deamidated (Q23)- Oxidation (M27)	4.4						VIVVGGGLAGLSAAIEAAGCGAQVVLMEKEAK Carbamidomethyl (C20)- Deamidated (Q23)- Oxidation (M27)	20.9
									VIVVGGGLAGLSAAIEAAGCGAQVVLMEKEAK Carbamidomethyl (C20)- Oxidation (M27)	32.5						VIVVGGGLAGLSAAIEAAGCGAQVVLMEKEAK Carbamidomethyl (C20)- Oxidation (M27)	27.8
									VLRFNLPGALQR	20.6						VQLHPTGLINPKDPANPTKFLGPEALR	51.0
									VQLHPTGLINPKDPANPTKFLGPEALR	11.0						VVASADTFDR	31.9
									VTGVEFTQAGSGK	41.4						VYNSSAGCFDPSTAPVAK Carbamidomethyl (C8)	71.0
									VVASADTFDRAKEVADEVLR	43.6							
									VYNSSAGCFDPSTAPVAK Carbamidomethyl (C8)	59.4							
17	Tb927.11.3980	mitochondrial processing peptidase alpha subunit, putative,metallo-peptidase, Clan ME, Family M16	1.00	1416	90	30	20	51.7	AAVELTSQCWDSSR Carbamidomethyl (C9)	36.0	1.00	922	66	21	16	48.7	ALLDQYAR	22.7
									ALLDQYAR	33.0						ASNSTQYLNPSEVLAAVR	53.1
									ASNSTQYLNPSEVLAAVR	71.1						ASNSTQYLNPSEVLAAVR Deamidated (N3)	54.0
									ASNSTQYLNPSEVLAAVR Deamidated (N3)	6.2						ASNSTQYLNPSEVLAAVR Deamidated (Q6)	67.5
									FDLNLLQDTIFTAISAPR	40.6						FDLNLLQDTIFTAISAPR	28.7
									FHEPDLER	33.5						FDLNLLQDTIFTAISAPR Deamidated (Q7)	51.3
									FRDTIDNNLK	29.1						FHEPDLER	28.8
									FRDTIDNNLKELR	54.0						FRDTIDNNLK	16.6
									GIQNVKFDNAALLEAAK	34.5						FRDTIDNNLKELR	30.7
									GIQNVKFDNAALLEAAKHR	54.1						GIQNVKFDNAALLEAAK	44.1
									GIQNVKFDNAALLEAAKHR Deamidated (N4)	37.6						GLGKPAFDITNEHSQYSGGELHEHEDRPK	50.3
									GIQNVKFDNAALLEAAKHR Deamidated (N4)- Deamidated (N9)	15.7						GLGKPAFDITNEHSQYSGGELHEHEDRPK Deamidated (N11)	45.0
									GLGKPAFDITNEHSQYSGGELHEHEDRPK	48.5						HVPEWSNGRITSK Deamidated (N7)	15.9
									HVPEWSNGRITSK	44.7						NVTASELKEVKECMMGSKPSLFGHGEMLAFPSLR Carbamidomethyl (C13)	42.5
									HVPEWSNGRITSK Deamidated (N7)	33.4						RFDLNLLQDTIFTAISAPR	34.4
									NVTASELKEVK	13.2						SFYLPYHSAGLVGFTAITSPENAVPLVK	26.6
									NVTASELKEVKECMMGSKPSLFGHGEMLAFPSLR Carbamidomethyl (C13)- Oxidation (M14)- Oxidation (M15)- Oxidation (M27)	28.2						SNTSGATSDAKRFDLNLLQDTIFTAISAPR	37.9
									RFDLNLLQDTIFTAISAPR	17.3						STPAAAGSAAQSAGIGEAKTTLLPNGTR Deamidated (Q11)- Deamidated (N25)	1.7
									SFYLPYHSAGLVGFTAITSPENAVPLVK	28.5						TTLLPNGTR Deamidated (N6)	30.4
									SNTSGATSDAKRFDLNLLQDTIFTAISAPR	51.9						WQRPAQYAIQQLETVAFYKEPLGNPR	49.1
									SNTSGATSDAKRFDLNLLQDTIFTAISAPR Deamidated (Q19)	45.5						WQRPAQYAIQQLETVAFYKEPLGNPR Deamidated (Q11)	31.9
									STPAAAGSAAQSAGIGEAK	79.8							
									STPAAAGSAAQSAGIGEAK Deamidated (Q11)	45.3							
									STPAAAGSAAQSAGIGEAKTTLLPNGTR	70.0							
									STPAAAGSAAQSAGIGEAKTTLLPNGTR Deamidated (N25)	55.6							
									STPAAAGSAAQSAGIGEAKTTLLPNGTR Deamidated (Q11)	53.1							
									TTLLPNGTR	34.2							
									TTLLPNGTR Deamidated (N6)	26.5							
									WQRPAQYAIQQLETVAFYKEPLGNPR	47.7							
									WQRPAQYAIQQLETVAFYKEPLGNPR Deamidated (Q11)	17.0							
18	Tb927.2.4230	NUP-1 protein, putative	1.00	1426	131	31	18	5.9	AALVSDVAVR	45.2	1.00	1210	78	22	13	4.0	AALVSDVAVR	48.9
									AALVSDVAVRNADTDLGTQLASALVALER	44.4						AALVSDVAVRNADTDLGTQLASALVALER	42.7
									AALVSDVAVRNADTDLGTQLASALVALER Deamidated (N11)	41.0						AALVSDVAVRNADTDLGTQLASALVALER Deamidated (N11)	53.3
									AALVSDVAVRNADTDLGTQLASALVALER Deamidated (Q19)	11.9						ASEEIFNLQNLQQQLEAALQK	69.7
									AALVSDVAVRNADTDLGTQLASALVALER Deamidated (Q19)- Deamidated (N11)	34.6						EAEEEIIQLTNKLNAAGVR	48.1
									ASEEIFNLQNLQQQLEAALQK	16.9						LAEEREAALEK	30.2
									AYQLRDDYKNEAAR	42.1						LAEEREAALEKATEMEER	10.0
									EAEQEIIQLTNKLNAAGVR	74.0						LREAEEEIIQLTNKLNAAGVR	65.0
									EAEQEIIQLTNKLNAAGVR Deamidated (Q4)	70.0						LREAEEEIIQLTNKLNAAGVR Deamidated (N13)	26.1
									EAEQEIIQLTNKLNAAGVR Deamidated (Q8)	42.5						LREAEEEIIQLTNKLNAAGVR Deamidated (N16)	40.8
									EGEAKDILLQK	26.3						LREAEEEIIQLTNKLNAAGVR Deamidated (Q10)	50.7
									LAEEREAALEK	20.3						LREAEQEIIQLTNKLNAAGVR	65.4
									LAEEREAALEKATEMEER	27.2						LREAEQEIIQLTNKLNAAGVR Deamidated (N13)	51.8
									LREAEEEIIQLTNKLNAAGVR	55.1						LREAEQEIIQLTNKLNAAGVR Deamidated (N13)- Deamidated (N16)	56.5
									LREAEEEIIQLTNKLNAAGVR Deamidated (N16)	41.0						LREAEQEIIQLTNKLNAAGVR Deamidated (N16)	21.6
									LREAEEEIIQLTNKLNAAGVR Deamidated (Q10)	23.8						LREAEQEIIQLTNKLNAAGVR Deamidated (Q10)	75.5
									LREAEQEIIQLTNKLNAAGVR	68.7						NADTDLGTQLASALVALER	79.5
									LREAEQEIIQLTNKLNAAGVR Deamidated (N16)	37.1						NADTDLGTQLASALVALER Deamidated (N1)	92.5
									LREAEQEIIQLTNKLNAAGVR Deamidated (Q10)- Deamidated (N16)	30.9						VSSEVAQHSSPPEQQIR	19.2
									NADTDLGTQLASALVALER	80.0						VSTLEEELR	44.9
									NADTDLGTQLASALVALER Deamidated (N1)	30.6						VSTLEEELRTAHSTTK	20.2
									NADTDLGTQLASALVALER Deamidated (Q9)	20.7						VSTLEEELRTAHSTTKK	35.0
									NADTDLGTQLASALVALERLAEEREAALEK Deamidated (N1)	23.0							
									NADTDLGTQLASALVALERLAEEREAALEK Deamidated (Q9)	4.6							
									NADTDLGTQLASALVALERLAEEREAALEK Deamidated (Q9)- Deamidated (N1)	14.4							
									NKLSALEASKR	23.3							
									SASSIQGGLTHETPPLLTPR	33.6							
									SLSHTQNQLQTAQER	50.7							
									VSSEVAQHSSPPEQQIR	40.5							
									VSTLEEELRTAHSTTK	21.0							
									VSTLEEELRTAHSTTKK	21.1							
19	Tb927.3.2230	succinyl-CoA synthetase alpha subunit, putative	1.00	723	68	21	12	42.2	AGSTYLNLPVFGTVAEAVK	4.3	1.00	922	52	18	8	38.9	AGSTYLNLPVFGTVAEAVK	55.8
									GKMEALEAAGAVVAHSPAQLGALMAELMK	10.1						GKMEALEAAGAVVAHSPAQLGALMAELMK	57.9
									GKMEALEAAGAVVAHSPAQLGALMAELMK Oxidation (M24)- Oxidation (M28)	41.3						GKMEALEAAGAVVAHSPAQLGALMAELMK Deamidated (Q19)	48.5
									GKMEALEAAGAVVAHSPAQLGALMAELMK Oxidation (M28)	33.5						GKMEALEAAGAVVAHSPAQLGALMAELMK Oxidation (M24)	50.1
									GKMEALEAAGAVVAHSPAQLGALMAELMK Oxidation (M3)- Oxidation (M24)- Oxidation (M28)	45.7						GKMEALEAAGAVVAHSPAQLGALMAELMK Oxidation (M24)- Oxidation (M28)	51.8
									GKMEALEAAGAVVAHSPAQLGALMAELMK Oxidation (M3)- Oxidation (M28)	26.8						GKMEALEAAGAVVAHSPAQLGALMAELMK Oxidation (M3)	38.5
									GKMEALEAAGAVVAHSPAQLGALMAELMKK Oxidation (M24)- Oxidation (M28)	11.6						GKMEALEAAGAVVAHSPAQLGALMAELMK Oxidation (M3)- Deamidated (Q19)	54.2
									GKMEALEAAGAVVAHSPAQLGALMAELMKK Oxidation (M3)- Oxidation (M24)- Oxidation (M28)	36.7						GKMEALEAAGAVVAHSPAQLGALMAELMKK	63.5
									GKMEALEAAGAVVAHSPAQLGALMAELMKK Oxidation (M3)- Oxidation (M28)	11.0						GKMEALEAAGAVVAHSPAQLGALMAELMKK Deamidated (Q19)	28.1

									HGTYHTEQAIAYGTK	41.0							GKMEALEAAGAVVAHSPAQLGALMAELMKK Oxidation (M24)- Oxidation (M28)	8.6
									KAGSTYLNLPVFGTVAEAVK	61.9							KAGSTYLNLPVFGTVAEAVK	56.2
									KAGSTYLNLPVFGTVAEAVK Deamidated (N8)	14.8							KAGSTYLNLPVFGTVAEAVK Deamidated (N8)	34.4
									KGCIGVVSR Carbamidomethyl (C3)	21.4							LIGPNCPGIIRPEECK Carbamidomethyl (C6)- Carbamidomethyl (C15)	19.0
									LIGPNCPGIIRPEECK Carbamidomethyl (C6)- Carbamidomethyl (C15)	23.7							LYLEDPGTEGIILIGEIGGTAEEEAAEFIKNHPIK	71.7
									LIGPNCPGIIRPEECK Deamidated (N5)- Carbamidomethyl (C6)- Carbamidomethyl (C15)	37.8							LYLEDPGTEGIILIGEIGGTAEEEAAEFIKNHPIK Deamidated (N31)	35.2
									MGHAGAVVSGGQGTAK Oxidation (M1)	51.4							MEALEAAGAVVAHSPAQLGALMAELMK	16.1
									MGHAGAVVSGGQGTAKGK	10.3							MGHAGAVVSGGQGTAK	32.9
									SMLLSQNKTR Oxidation (M2)	16.2							MGHAGAVVSGGQGTAK Oxidation (M1)	50.5
									VICQGITGK Carbamidomethyl (C3)	24.5								
									VKSMLLSQNKTR Oxidation (M4)	33.7								
									VKSMLLSQNKTR Oxidation (M4)- Deamidated (Q8)	29.8								
20	Tb927.5.1060	mitochondrial processing peptidase, beta subunit, putative,metallo-peptidase, Clan ME, Family M16	1.00	1466	156	26	24	52.8	AATSAFGDLQK	33.0	1.00	818	66	17	15	36.6	AATSAFGDLQK	24.3
									AATSAFGDLQKGTVTLAGVPEAR	62.9							AATSAFGDLQKGTVTLAGVPEAR	47.2
									AVSLLADVVR	51.0							AVSLLADVVR	33.0
									AVSLLADVVRNAR Deamidated (N11)	16.1							AVSLLADVVRNAR Deamidated (N11)	23.7
									CAFDSTTHGPGTPLYGTEVGTTR Carbamidomethyl (C1)	69.7							EDTERAVSLLADVVR	12.1
									EDTERAVSLLADVVR	33.8							FVGGEYKLWNLR	45.4
									FVGGEYKLWNLR	55.4							GTVTLAGVPEAR	51.1
									GTVTLAGVPEAR	59.6							IDDVTPTNVQEVLQHYFYGR	66.3
									HEPAQYAGTAR	9.2							ILHEQELAQAK	42.6
									IDDVTPTNVQEVLQHYFYGR	45.0							IPPTTLSTVGSGVR	47.3
									ILHEQELAQAK	56.3							RIPLEEMYAR	14.8
									IPPTTLSTVGSGVR	60.0							TFSSLDHSTPTNTHFNEK	33.5
									LLSNVLAKIPPTTLSTVGSGVR	52.8							TFSSLDHSTPTNTHFNEK Deamidated (N12)	34.4
									LSDEDVEVAK	41.8							VACEENPIASLATVGVWLDAGTRHEPAQYAGTAR Carbamidomethyl (C3)	56.3
									LSDEDVEVAKQAVLR	32.6							VACEENPIASLATVGVWLDAGTRHEPAQYAGTAR Carbamidomethyl (C3)- Deamidated (N6)	56.4
									QAVLRDQHDFEQRPDDICMDNLHR Carbamidomethyl (C18)- Oxidation (M19)	19.1							VAREDTERAVSLLADVVR	23.7
									RIPLEEMYAR	31.7							VVVVGSGAVNHTALER Deamidated (N10)	17.1
									SRPSSNHATTK	21.5								
									SRPSSNHATTKLLSNVLAK	45.0								
									SRPSSNHATTKLLSNVLAK Deamidated (N15)	33.7								
									TFSSLDHSTPTNTHFNEK	45.6								
									VACEENPIASLATVGVWLDAGTRHEPAQYAGTAR Carbamidomethyl (C3)	78.9								
									VAREDTERAVSLLADVVR	33.5								
									VLKTFSSLDHSTPTNTHFNEK	37.8								
									VVVVGSGAVNHTALER	70.7								
									VVVVGSGAVNHTALER Deamidated (N10)	46.7								
21	Tb927.11.5520	triosephosphate isomerase	1.00	1196	128	26	13	47.2	DVNGFLVGGASLKPEFVDIIK	43.8	1.00	1516	134	28	14	51.2	DVNGFLVGGASLKPEFVDIIK	51.3
									DVNGFLVGGASLKPEFVDIIK Deamidated (N3)	44.6							DVNGFLVGGASLKPEFVDIIK Deamidated (N3)	47.7
									DVNGFLVGGASLKPEFVDIIKATQ	38.9							DVNGFLVGGASLKPEFVDIIKATQ	39.9
									DVNGFLVGGASLKPEFVDIIKATQ Deamidated (N3)	39.8							DVNGFLVGGASLKPEFVDIIKATQ Deamidated (N3)	32.3
									FVIAAQNAIAK	61.0							FVIAAQNAIAK	47.8
									FVIAAQNAIAK Deamidated (N7)	41.9							FVIAAQNAIAK Deamidated (Q6)	57.2
									FVIAAQNAIAK Deamidated (Q6)	42.0							IGADVAGELR	77.9
									ILYGGSVNGK Deamidated (N8)	10.7							ILYGGSVNGK Deamidated (N8)	23.6
									ILYGGSVNGKNAR Deamidated (N8)	15.0							ILYGGSVNGKNAR	66.1
									ILYGGSVNGKNAR Deamidated (N8)- Deamidated (N11)	41.7							ILYGGSVNGKNAR Deamidated (N11)	46.8
									LSHPKFVIAAQNAIAK	49.3							ILYGGSVNGKNAR Deamidated (N8)	43.0
									LSHPKFVIAAQNAIAK Deamidated (N12)	53.7							ILYGGSVNGKNAR Deamidated (N8)- Deamidated (N11)	19.6
									SGAFTGEVSLPILKDFGVNWIVLGHSER	11.2							LSHPKFVIAAQNAIAK	43.4
									SGAFTGEVSLPILKDFGVNWIVLGHSERR	9.8							LSHPKFVIAAQNAIAK Deamidated (Q11)	19.3
									TLYQQRDVNGFLVGGASLKPEFVDIIK	32.9							SGAFTGEVSLPILKDFGVNWIVLGHSER	40.3
									TLYQQRDVNGFLVGGASLKPEFVDIIK Deamidated (N9)	42.1							SGAFTGEVSLPILKDFGVNWIVLGHSERR	34.4
									TLYQQRDVNGFLVGGASLKPEFVDIIK Deamidated (Q5)	17.3							TLYQQRDVNGFLVGGASLKPEFVDIIK	22.6
									TLYQQRDVNGFLVGGASLKPEFVDIIK Deamidated (Q5)- Deamidated (N9)	19.3							TLYQQRDVNGFLVGGASLKPEFVDIIK Deamidated (N9)	30.8
									TLYQQRDVNGFLVGGASLKPEFVDIIKATQ	48.3							TLYQQRDVNGFLVGGASLKPEFVDIIK Deamidated (Q5)	42.4
									TLYQQRDVNGFLVGGASLKPEFVDIIKATQ Deamidated (N9)	32.4							TLYQQRDVNGFLVGGASLKPEFVDIIKATQ	40.0
									TLYQQRDVNGFLVGGASLKPEFVDIIKATQ Deamidated (Q30)	31.7							TLYQQRDVNGFLVGGASLKPEFVDIIKATQ Deamidated (N9)	32.3
									TLYQQRDVNGFLVGGASLKPEFVDIIKATQ Deamidated (Q5)- Deamidated (Q30)	33.3							TLYQQRDVNGFLVGGASLKPEFVDIIKATQ Deamidated (Q30)	50.4
									VATPQQAQEAHALIR	54.3							TLYQQRDVNGFLVGGASLKPEFVDIIKATQ Deamidated (Q5)- Deamidated (Q30)	30.5
									VATPQQAQEAHALIR Deamidated (Q8)	19.0							VATPQQAQEAHALIR	21.4
									VVIAYEPVWAIGTGK	53.1							VATPQQAQEAHALIR Deamidated (Q6)	65.7
									VVIAYEPVWAIGTGKVATPQQAQEAHALIR	23.0							VATPQQAQEAHALIR Deamidated (Q8)	60.8
																	VVIAYEPVWAIGTGK	33.7
																	VVIAYEPVWAIGTGKVATPQQAQEAHALIR	48.3
22	Tb927.4.2070	antigenic protein, putative	1.00	1118	66	25	13	4.6	ALKEQYETLQR Deamidated (Q5)	33.3	1.00	221	14	6	4	1.0	ALKEQYETLQR Deamidated (Q5)	17.4
									ALNEQYETLQR	49.3							ALNEQYETLQR	48.4
									AVNEQYEALQR	45.7							ALNEQYETLQR Deamidated (N3)	38.9
									TNAAMEDELDSLR Deamidated (N2)- Oxidation (M5)	44.8							AVNEQYETLQR	45.0
									TNAAMEDELDSLRAANEQYETLQR Deamidated (N2)- Oxidation (M5)	12.2							TNAAMEEELDSLR	19.9
									TNAAMEDELDSLRALKEQYETLQR Oxidation (M5)- Deamidated (Q18)	41.8							TNAAMEEELDSLR Deamidated (N2)	30.5
									TNAAMEDELDSLRALNEQYETLQR	60.2								
									TNAAMEDELDSLRALNEQYETLQR Deamidated (N2)	18.2								
									TNAAMEDELDSLRALNEQYETLQR Deamidated (N2)- Oxidation (M5)	56.5								
									TNAAMEDELDSLRALNEQYETLQR Deamidated (Q23)- Deamidated (N2)	42.7								
									TNAAMEDELDSLRALNEQYETLQR Oxidation (M5)	36.0								
									TNAAMEDELDSLRALNEQYETLQR Oxidation (M5)- Deamidated (Q18)	33.6								
									TNAAMEDELDSLRAVNEQYETLQR	17.7								
									TNAAMEDELDSLRAVNEQYETLQR Deamidated (N2)	22.6								
									TNAAMEDELDSLRAVNEQYETLQR Deamidated (N2)- Oxidation (M5)	32.3								
									TNAAMEDELDSLRAVNEQYETLQR Oxidation (M5)	39.2								
									TNAAMEEELDSLR Deamidated (N2)- Oxidation (M5)	26.4								
									TNAAMEEELDSLRAANEQYETLQR Oxidation (M5)	28.6								
									TNAAMEEELDSLRALNEQYETLQR	57.2								
									TNAAMEEELDSLRALNEQYETLQR Deamidated (N2)	27.8								
									TNAAMEEELDSLRALNEQYETLQR Deamidated (Q23)	39.7								
									TNAAMEEELDSLRALNEQYETLQR Oxidation (M5)	29.7								
									TNAAMEEELDSLRAVNEQYEALQR	14.3								
									TNAAMEEELDSLRAVNEQYEALQR Deamidated (N2)	28.7								
									TNAAMEEELDSLRAVNEQYETLQR	49.3								
23	Tb927.11.11680	2-oxoglutarate dehydrogenase E2 component, putative	1.00	1416	89	29	21	43.1	AETADKPKQNAPAAAAPPK	26.7	1.00	633	38	17	13	31.6	AETADKPKQNAPAAAAPPK	1.9
									AETADKPKQNAPAAAAPPKASPTEAAPKPAPAAAPVTSR	78.0							ASPTEAAPKPAPAAAPVTSR	35.8
									APAAGVITK	30.9							DIMAVALTYDHR	54.2
									ASPTEAAPKPAPAAAPVTSR	46.9							DVQNSNFAQIEK	49.6
									DIMAVALTYDHR Oxidation (M3)	53.4							DVQNSNFAQIEKQIADFGER	16.9
									DIMAVALTYDHRLIDGSDAVTFLVK Oxidation (M3)	27.1							GLVVPVLRDVQNSNFAQIEK	36.7
									DVQNSNFAQIEK	61.5							GLVVPVLRDVQNSNFAQIEK Deamidated (N14)	22.0
									DVQNSNFAQIEKQIADFGER	65.3							GLVVPVLRDVQNSNFAQIEK Deamidated (Q17)	22.2
									DVQNSNFAQIEKQIADFGER Deamidated (N6)	41.6							GLVVPVLRDVQNSNFAQIEKQIADFGER	23.0
									GLVVPVLRDVQNSNFAQIEK	66.9							GLVVPVLRDVQNSNFAQIEKQIADFGER Deamidated (N12)- Deamidated (N14)	11.2
									GLVVPVLRDVQNSNFAQIEK Deamidated (N12)	44.1							IVLDLA	19.7
									GLVVPVLRDVQNSNFAQIEK Deamidated (N14)	43.8							KKPWVVGNSVVPR Deamidated (N8)	13.2
									GLVVPVLRDVQNSNFAQIEKQIADFGER	32.9							LIDGSDAVTFLVK	57.9
									GLVVPVLRDVQNSNFAQIEKQIADFGER Deamidated (N12)	31.0							NLIEDPARIVLDLA	49.6
									GLVVPVLRDVQNSNFAQIEKQIADFGER Deamidated (N12)- Deamidated (N14)	30.9							QNAPAAAAPPKASPTEAAPKPAPAAAPVTSR	35.4
									GLVVPVLRDVQNSNFAQIEKQIADFGER Deamidated (N14)	43.2							QNAPAAAAPPKASPTEAAPKPAPAAAPVTSR Deamidated (N2)	34.0
									GLVVPVLRDVQNSNFAQIEKQIADFGER Deamidated (Q11)	49.2							VKNLIEDPAR	50.8
									GLVVPVLRDVQNSNFAQIEKQIADFGER Deamidated (Q11)- Deamidated (N12)	3.7								
									IVLDLA	44.1								
									KKPWVVGNSVVPR	39.1								
									LGFMSPFVK Oxidation (M4)	28.4								

									LIDGSDAVTFLVK	69.8								
									NLIEDPARIVLDLA	45.8								
									QIADFGER	14.0								
									QIADFGERAR	31.5								
									QNAPAAAAPPKASPTEAAPKPAPAAAPVTSR	23.2								
									VGDAVAEDEIICQIESDKLNVDVRAPAAGVITK Carbamidomethyl (C12)	22.2								
									VKNLIEDPAR	57.3								
									VKNLIEDPARIVLDLA	46.0								
24	Tb927.10.8030	hypothetical protein, conserved	1.00	992	85	20	15	58.0	AAEEKGNFDNILSDFQK	49.5	1.00	1159	89	23	14	61.6	AAEEKGNFDNILSDFQK	46.6
									AAEEKGNFDNILSDFQK Deamidated (Q16)	50.5							AAEEKGNFDNILSDFQK Deamidated (N10)	54.7
									ADEVDYTNLPAPKPQK	39.0							AAEEKGNFDNILSDFQK Deamidated (N7)	32.8
									FLYKAAEEKGNFDNILSDFQK	24.3							AAEEKGNFDNILSDFQK Deamidated (Q16)	10.8
									IAAAASKLPIFWER	50.3							ADEVDYTNLPAPKPQK Deamidated (N8)	33.2
									IFVAPGGEKNVEEAR	69.8							GLKELADYTLVLK	39.0
									IFVAPGGEKNVEEARR	29.4							IAAAASKLPIFWER	56.4
									KLYASDFENSKYPLNIVPSDSVLFAK	43.2							IFVAPGGEKNVEEAR	17.5
									KLYASDFENSKYPLNIVPSDSVLFAK Deamidated (N9)	34.6							IFVAPGGEKNVEEARR	19.0
									KLYASDFENSKYPLNIVPSDSVLFAK Deamidated (N9)- Deamidated (N15)	29.8							KLYASDFENSKYPLNIVPSDSVLFAK	60.8
									KYLDGLSQYDMEEAKYGV Deamidated (Q8)- Oxidation (M11)	32.1							KLYASDFENSKYPLNIVPSDSVLFAK Deamidated (N9)	34.1
									KYLDGLSQYDMEEAKYGV Oxidation (M11)	25.3							LYASDFENSKYPLNIVPSDSVLFAK	33.1
									LYASDFENSKYPLNIVPSDSVLFAK	22.5							LYASDFENSKYPLNIVPSDSVLFAK Deamidated (N8)	34.4
									QAGRADEVDYTNLPAPKPQK	35.9							QAGRADEVDYTNLPAPKPQK	39.7
									QAGRADEVDYTNLPAPKPQKTVWDDNIETEVLR Deamidated (N26)	40.9							QAGRADEVDYTNLPAPKPQK Deamidated (N12)	38.0
									TAVVEKIPEFK	33.5							TAVVEKIPEFK	37.8
									TVVDRTIVK	24.0							TVVDRTIVK	34.5
									TVWDDNIETEVLRK	65.1							TVWDDNIETEVLR	62.0
									TVWDDNIETEVLRK Deamidated (N6)	67.2							TVWDDNIETEVLR Deamidated (N6)	77.6
									YLDGLSQYDMEEAKYGV Oxidation (M10)	52.8							TVWDDNIETEVLRK	43.8
																	TVWDDNIETEVLRK Deamidated (N6)	65.9
																	YLDGLSQYDMEEAKYGV	16.3
																	YLDGLSQYDMEEAKYGV Oxidation (M10)	29.4
25	Tb927.10.7410	succinyl-CoA ligase [GDP-forming] beta-chain, putative-with=GeneDB:LmjF36.2950	1.00	895	66	19	15	42.0	AAENAAEQIK	37.3	1.00	886	58	19	16	54.4	AAENAAEQIKALYNVGK	45.7
									AAENAAEQIKALYNVGK	61.1							ALLKEGGCKTEFGVACSSIEEVEAALGK Carbamidomethyl (C8)- Carbamidomethyl (C16)	42.4
									ALLKEGGCKTEFGVACSSIEEVEAALGK Carbamidomethyl (C8)- Carbamidomethyl (C16)	34.2							ALYNVGK	13.6
									ALYNVGK	26.0							CDTIAEGIVAASR Carbamidomethyl (C1)	59.3
									CDTIAEGIVAASR Carbamidomethyl (C1)	66.2							ELYLALLLDRK	71.4
									ELYLALLLDRK	48.3							ESGLPLHPAQNFEEAAALACK Carbamidomethyl (C20)	46.0
									ESGLPLHPAQNFEEAAALACK Carbamidomethyl (C20)	48.4							GMGTFVDGFKGGVHVCKDAAEALACAR Carbamidomethyl (C16)- Carbamidomethyl (C25)	26.6
									ESGLPLHPAQNFEEAAALACKLAA Carbamidomethyl (C20)	12.5							ILKESGLPLHPAQNFEEAAALACK Carbamidomethyl (C23)	27.1
									GVREVPVVVR	14.9							ILKESGLPLHPAQNFEEAAALACKLAA Carbamidomethyl (C23)	18.8
									ILKESGLPLHPAQNFEEAAALACK Carbamidomethyl (C23)	46.5							INVQEGINHDNCLTFAHELGFTGR Carbamidomethyl (C12)	43.9
									ILKESGLPLHPAQNFEEAAALACK Deamidated (Q13)- Carbamidomethyl (C23)	28.5							INVQEGINHDNCLTFAHELGFTGR Deamidated (N2)- Carbamidomethyl (C12)	42.7
									ILKESGLPLHPAQNFEEAAALACK Deamidated (Q13)- Deamidated (N14)- Carbamidomethyl (C23)	8.8							INVQEGINHDNCLTFAHELGFTGR Deamidated (Q4)- Deamidated (N2)- Carbamidomethyl (C12)	25.8
									ILKESGLPLHPAQNFEEAAALACKLAA Carbamidomethyl (C23)	31.1							LSFDDNAEFRQHDIFALADATQIDPK	36.2
									INVQEGINHDNCLTFAHELGFTGR Carbamidomethyl (C12)	51.4							LSFDDNAEFRQHDIFALADATQIDPKEVVAK	49.9
									INVQEGINHDNCLTFAHELGFTGR Deamidated (N2)- Deamidated (N11)- Carbamidomethyl (C12)	61.7							LSFDDNAEFRQHDIFALADATQIDPKEVVAK Deamidated (Q11)	48.8
									INVQEGINHDNCLTFAHELGFTGR Deamidated (N8)- Carbamidomethyl (C12)	55.9							LYVTEAISGIKR	50.1
									LYVTEAISGIKR	53.7							QHDIFALADATQIDPKEVVAK	25.2
									SKDCTMVEINPLVELENGDVMCIDAK Carbamidomethyl (C4)- Carbamidomethyl (C22)- Oxidation (M6)- Oxidation (M21)	42.9							SILVNIFGGIMR	64.1
									SQILAGGR	22.0							SKDCTMVEINPLVELENGDVMCIDAK Carbamidomethyl (C4)- Carbamidomethyl (C22)- Oxidation (M21)	8.1
26	Tb927.9.5900	glutamate dehydrogenase	1.00	776	68	20	17	28.1	ASILSSDMCENKELVR Oxidation (M8)- Carbamidomethyl (C9)	36.2	1.00	1263	67	26	19	31.3	ASLVPFLPTRPR	33.5
									ASLVPFLPTRPR	31.8							CEAEIVKLTK Carbamidomethyl (C1)	57.2
									CYTAGDSDIAVFTAK Carbamidomethyl (C1)	28.5							CYTAGDSDIAVFTAK Carbamidomethyl (C1)	4.0
									GIFSDASVEASVHQLMSR	7.2							FQTGGPDGDLGSNEILLSK	46.9
									GIFSDASVEASVHQLMSR Oxidation (M16)	14.9							FQTGGPDGDLGSNEILLSK Deamidated (Q2)	31.3
									IRPFVNPNPEKGETDIGQLASSAFLEER	80.0							GIFSDASVEASVHQLMSR	69.1
									IRPFVNPNPEKGETDIGQLASSAFLEER Deamidated (Q18)	49.8							GIFSDASVEASVHQLMSR Oxidation (M16)	7.1
									LKTPEIIFLGPDEHTAGTFPSVGSLFSK	27.7							IFLQYADALLDVVIPGEEGIVDR	28.1
									LSDSPYVR	23.3							IFLQYADALLDVVIPGEEGIVDRLK	51.0
									SFTTGKDASLGGIPHDTYAMTTR	7.8							IRPFVNPNPEKGETDIGQLASSAFLEER	70.8
									SLVSKEPNGLSR	22.1							IRPFVNPNPEKGETDIGQLASSAFLEER Deamidated (N6)	24.8
									SLVSKEPNGLSR Deamidated (N8)	28.6							LKTPEIIFLGPDEHTAGTFPSVGSLFSK	19.7
									SVFQENYNLALTQLLK	42.2							LSDSPYVR	18.1
									SVTLANVGR	12.9							SFTTGKDASLGGIPHDTYAMTTR	9.1
									TLAVLDISASLFDPEGLNKEELKR	49.0							SLVSKEPNGLSR	31.6
									TLIADSLSEKNVR	56.8							SLVSKEPNGLSR Deamidated (N8)	32.9
									TLLEVVPLETLMERVPLEYQK Oxidation (M12)	28.6							SVFQENYNLALTQLLK	46.5
									YLNTFNQPLCER Carbamidomethyl (C10)	33.6							SVFQENYNLALTQLLK Deamidated (Q4)	38.0
									YSAADVFVPCGGRPR Carbamidomethyl (C10)	25.1							SYYIATLQTVIQEIPGAVVTR	62.4
									YVLSKYTPK	25.5							TLAVLDISASLFDPEGLNKEELKR	41.6
																	TLAVLDISASLFDPEGLNKEELKR Deamidated (N18)	18.5
																	TLIADSLSEKNVR	50.1
																	TPEIIFLGPDEHTAGTFPSVGSLFSK	61.9
																	YILTLISTYPEFMKEIYEDFQVGTTHER	52.7
																	YILTLISTYPEFMKEIYEDFQVGTTHER Deamidated (Q21)	50.2
																	YLNTFNQPLCER Carbamidomethyl (C10)	29.5
27	Tb927.5.2930	hypothetical protein, conserved	1.00	1069	101	22	17	47.0	ASQDKRLDDLVDLLER	45.3	1.00	830	62	19	15	40.0	ASQDKRLDDLVDLLER	43.4
									ASQDKRLDDLVDLLER Deamidated (Q3)	23.4							ASQDKRLDDLVDLLER Deamidated (Q3)	34.4
									DRLLKEVLALQAR	32.7							DRLLKEVLALQAR	14.6
									DRLLKEVLALQAR Deamidated (Q11)	13.6							HLEGQLGVWR	18.7
									EVEAKEKLAALR	26.0							INQRDEVEPSQTEQK Deamidated (Q11)	33.1
									EVLALQAR	31.7							INWPQEYSSFIDREPVVAK	13.1
									INWPQEYSSFIDREPVVAK	21.8							INWPQEYSSFIDREPVVAK Deamidated (Q5)	21.0
									INWPQEYSSFIDREPVVAK Deamidated (Q5)	25.5							KSAEAAERNPIVTQTSTGDNAR	43.1
									INWPQEYSSFIDREPVVAKR	26.5							KSSATIEQVEKEIDALLGGAEK	53.4
									KSAEAAERNPIVTQTSTGDNAR	63.1							LDDLVDLLER	73.3
									KSSATIEQVEKEIDALLGGAEK	37.5							LLKEVLALQAR	58.0
									LLKEVLALQAR	64.6							LLKEVLALQAR Deamidated (Q9)	52.6
									LTIKHLEGQLGVWR	31.6							NPIVTQTSTGDNAR	7.3
									RREEEGLPGGPVPR	32.6							RLDDLVDLLER	30.7
									SAEAAERNPIVTQTSTGDNAR	43.3							RREEEGLPGGPVPR	35.6
									SAEAAERNPIVTQTSTGDNAR Deamidated (Q13)	46.8							SAEAAERNPIVTQTSTGDNAR	29.4
									SSATIEQVEKEIDALLGGAEK	73.9							SAEAAERNPIVTQTSTGDNAR Deamidated (N19)	40.6
									SSATIEQVEKEIDALLGGAEKLR	54.2							SSATIEQVEKEIDALLGGAEK	63.1
									SVNEVREIMSK	35.6							WVVYTPEDEVKLAQLKR	30.7
									SVNEVREIMSK Oxidation (M9)	28.7								
									WVVYTPEDEVKLAQLKR	37.6								
									YKGEVPPHYTR	33.7								
28	Tb927.5.2080	guanosine monophosphate reductase, putative	1.00	813	57	18	16	42.0	AIGAGADTVMLGNMLAGTDEAPGR Oxidation (M10)- Oxidation (M14)	41.9	1.00	622	28	13	12	36.5	AIGAGADTVMLGNMLAGTDEAPGR	81.8
									AIGAGADTVMLGNMLAGTDEAPGRVLVKDGQK Oxidation (M10)- Oxidation (M14)	44.1							AQSFLIESPR	52.1
									AQSFLIESPR	52.1							EGGIGILHR	25.2
									EGGIGILHR	40.3							ERTQDEDVFSSLVPEGVEGSVACKGPVGPIVR Carbamidomethyl (C23)	56.3
									ERTQDEDVFSSLVPEGVEGSVACKGPVGPIVR Carbamidomethyl (C23)	65.5							GVPCIADGGLR Carbamidomethyl (C4)	41.8
									GVPCIADGGLR Carbamidomethyl (C4)	43.5							IGVGPGSICITR Carbamidomethyl (C9)	44.1
									GVPCIADGGLRTSGDISK Carbamidomethyl (C4)	32.9							IILPHETAR	21.3
									IGVGPGSICITR Carbamidomethyl (C9)	24.2							LVAGAGVPQLSAVLACTR Carbamidomethyl (C16)	6.6
									IILPHETAR	28.5							RAQSFLIESPR	22.0
									LSIPIVASNMDTVCEQR Oxidation (M10)- Carbamidomethyl (C14)	38.8							TANVPIVGQNGQLLYLVTLSDVVK	39.7
									LVAGAGVPQLSAVLACTR Carbamidomethyl (C16)	40.3							TANVPIVGQNGQLLYLVTLSDVVK Deamidated (N10)	62.1
									LVAGAGVPQLSAVLACTR Deamidated (Q9)- Carbamidomethyl (C16)	5.7							TASVDIIAGNIASAEAAEALIDAGADGLK	28.5
									MTGAGLRESGSHGVAKL	16.1							TQDEDVFSSLVPEGVEGSVACKGPVGPIVR Carbamidomethyl (C21)	49.2

								MTGAGLRESGSHGVAKL Oxidation (M1)	25.0								
								RAQSFLIESPR	26.0								
								RRGVPCIADGGLR Carbamidomethyl (C6)	38.6								
								TASVDIIAGNIASAEAAEALIDAGADGLKIGVGPGSICITR Carbamidomethyl (C38)	34.8								
								TQDEDVFSSLVPEGVEGSVACKGPVGPIVR Carbamidomethyl (C21)	57.5								
29	Tb927.9.5320	nucleolar RNA binding protein, putative	1.00	953	47	19	13	28.9	AGSLLSLAKYPSSTLQILGAEK	29.3	1.00	704	26	12	9	26.6	AIKDALAIQCVHGDDTLATFR Carbamidomethyl (C10)	48.1
								AIKDALAIQCVHGDDTLATFR Carbamidomethyl (C10)	52.9							AIKDALAIQCVHGDDTLATFR Deamidated (Q9)- Carbamidomethyl (C10)	42.0
								AIKDALAIQCVHGDDTLATFR Deamidated (Q9)- Carbamidomethyl (C10)	27.4							ALRANLDDLLEDVSTEQLNQTALGLAHNLNR	75.6
								ALRANLDDLLEDVSTEQLNQTALGLAHNLNR	51.8							ANLDDLLEDVSTEQLNQTALGLAHNLNR	100.7
								ANLDDLLEDVSTEQLNQTALGLAHNLNR	66.5							ANLDDLLEDVSTEQLNQTALGLAHNLNR Deamidated (Q17)	67.1
								ANLDDLLEDVSTEQLNQTALGLAHNLNRYK	45.8							ANLDDLLEDVSTEQLNQTALGLAHNLNRYK Deamidated (N2)	60.9
								ANLDDLLEDVSTEQLNQTALGLAHNLNRYK Deamidated (Q14)	32.0							LRQFEEGVTYGR	41.5
								GPPNGNGGGAPFKR Deamidated (N4)	21.4							NFVEKQITEELAVADAK	42.9
								GPPNGNGGGAPFKR Deamidated (N6)	28.8							RGPPNGNGGGAPFKR Deamidated (N5)- Deamidated (N7)	18.9
								LIQKAGSLLSLAK	52.0							RGPPNGNGGGAPFKR Deamidated (N7)	42.2
								LIQKAGSLLSLAKYPSSTLQILGAEK	22.2							STADALATTTQVVNGELAKPVR Deamidated (N14)	31.9
								LRQFEEGVTYGR	55.5							YPSSTLQILGAEK	47.5
								QITEELAVADAKLAK	41.0								
								RGPPNGNGGGAPFKR Deamidated (N5)	22.7								
								RGPPNGNGGGAPFKR Deamidated (N5)- Deamidated (N7)	29.5								
								RGPPNGNGGGAPFKR Deamidated (N7)	33.7								
								STADALATTTQVVNGELAKPVRK	55.6								
								STADALATTTQVVNGELAKPVRK Deamidated (N14)	59.7								
								YPSSTLQILGAEK	31.1								
30	Tb927.8.2770	inositol 1,4,5-trisphosphate receptor	1.00	636	71	18	15	8.4	EAVSALAR	14.3	1.00	1308	97	26	22	12.9	ALLAYFQEHQER	36.7
								GGVVPNVSNAHEFTR	45.6							ALVFVVER	13.4
								GSNVGTGYASLLPVICACQR Carbamidomethyl (C16)- Carbamidomethyl (C18)	1.5							APNSNPLSDGVSNIYSTQQVTFGQLFVLVHAVSR	74.6
								LHVAALPSEPSERDPDCAR Carbamidomethyl (C17)	8.3							GGVVPNVSNAHEFTR	55.4
								LKHVSAENSSVITDIFTVR	2.6							GGVVPNVSNAHEFTR Deamidated (N9)	17.9
								LLQTTFSDLGATR	57.4							GSNVGTGYASLLPVICACQR Carbamidomethyl (C16)- Carbamidomethyl (C18)	47.9
								LVLAPPGEIEQTFCQFIFTPR Carbamidomethyl (C14)	33.2							HVSAENSSVITDIFTVR	79.7
								LVLKELAAVR	32.9							KGPSDYQSTVAGSAVTTQLSGSTKDVAAFLDYVK	54.8
								MALSAVLGAPEVNLSEAK	23.4							LGLHVFTK	55.8
								MALSAVLGAPEVNLSEAK Oxidation (M1)	57.3							LHVAALPSEPSERDPDCAR Carbamidomethyl (C17)	12.5
								MALSAVLGAPEVNLSEAK Oxidation (M1)- Deamidated (N13)	69.7							LKHVSAENSSVITDIFTVR	86.4
								MLLTNALTTPPSLYSSLK Oxidation (M1)	10.7							LLQTTFSDLGATR	61.8
								MLLTNALTTPPSLYSSLKVR Oxidation (M1)	36.3							LVLAPPGEIEQTFCQFIFTPR Carbamidomethyl (C14)	47.3
								NKNELSDDAEPLPLFGFETSVFQILPPTVAK	24.0							MALSAVLGAPEVNLSEAK	56.9
								NKNELSDDAEPLPLFGFETSVFQILPPTVAK Deamidated (Q23)	13.4							MALSAVLGAPEVNLSEAK Oxidation (M1)	17.9
								RADVSSTGDPFVTSPTERDALLWHK	9.3							MALSAVLGAPEVNLSEAK Oxidation (M1)- Deamidated (N13)	28.3
								SALPVTHLLEITLSYGTSYTPLVEVPRPR	9.9							MLLTNALTTPPSLYSSLK Oxidation (M1)	46.0
								SFETDYAKENAGR	52.9							NKNELSDDAEPLPLFGFETSVFQILPPTVAK	34.8
																NKNELSDDAEPLPLFGFETSVFQILPPTVAK Deamidated (Q23)	23.8
																RADVSSTGDPFVTSPTER	30.7
																RADVSSTGDPFVTSPTERDALLWHK	28.6
																SALPVTHLLEITLSYGTSYTPLVEVPRPR	52.6
																SFETDYAKENAGR	52.6
																SLVNRDQLITMEDSTDLGEPAGIMNALLLSCR Carbamidomethyl (C31)	33.0
																VAAEEGLSKGNR	35.2
																YIDGEIAALHPDGEFATK	27.5
31	Tb927.10.3210	delta-1-pyrroline-5-carboxylate dehydrogenase, putative	1.00	827	69	19	14	31.4	AIRDATEKHLR	21.2	1.00	650	33	16	11	24.6	AAIFLHAAHLISTK	57.6
								ASGSNDKPGSALFLTR	56.6							AIDTALGAAK	31.7
								ASGSNDKPGSALFLTR Deamidated (N5)	58.5							CTGAVVGQQPFGGAR Carbamidomethyl (C1)	62.8
								CGATDCPIVIGGKEYR Carbamidomethyl (C1)- Carbamidomethyl (C6)	25.0							GTLEACKR Carbamidomethyl (C6)	21.4
								CGATDCPIVIGGKEYRTDNAINR Carbamidomethyl (C1)- Carbamidomethyl (C6)	44.3							GYNATPELAQK Deamidated (N3)	25.3
								GAFEYQGQKCSATSR Carbamidomethyl (C10)	49.3							MIPSSHQQQVAK	39.1
								GTLEACKR Carbamidomethyl (C6)	24.9							MIPSSHQQQVAK Oxidation (M1)	41.2
								GYNATPELAQK	57.1							SPPAVNERMDSFQPGSDSAR Oxidation (M9)	9.7
								LRCGATDCPIVIGGK Carbamidomethyl (C3)- Carbamidomethyl (C8)	35.7							TIKENFDHSAQVSYPHQLPDYVTL	23.2
								MIPSSHQQQVAK	26.9							TIKENFDHSAQVSYPHQLPDYVTL Deamidated (Q17)	4.3
								MIPSSHQQQVAK Oxidation (M1)	25.4							YAAGNYYINDKCTGAVVGQQPFGGAR Carbamidomethyl (C12)	34.9
								MIPSSHQQQVAKGYNATPELAQK Oxidation (M1)	13.2							YAAGNYYINDKCTGAVVGQQPFGGAR Deamidated (N9)- Carbamidomethyl (C12)	25.9
								MTAALTVRGAFEYQGQKCSATSR Carbamidomethyl (C18)	35.6							YAAGNYYINDKCTGAVVGQQPFGGAR Deamidated (Q19)- Deamidated (N5)- Carbamidomethyl (C12)	55.0
								SPPAVNERMDSFQPGSDSAR Oxidation (M9)	32.6							YAAGNYYINDKCTGAVVGQQPFGGAR Deamidated (Q19)- Deamidated (N9)- Carbamidomethyl (C12)	31.1
								TIKENFDHSAQVSYPHQLPDYVTL	29.6							YALTGSIFAQDR	71.2
								YAAGNYYINDKCTGAVVGQQPFGGAR Carbamidomethyl (C12)	45.8							YALTGSIFAQDRK	75.7
								YAAGNYYINDKCTGAVVGQQPFGGAR Carbamidomethyl (C12)- Deamidated (Q19)	42.5								
								YAAGNYYINDKCTGAVVGQQPFGGAR Deamidated (N5)- Carbamidomethyl (C12)	36.1								
								YAAGNYYINDKCTGAVVGQQPFGGAR Deamidated (N9)- Carbamidomethyl (C12)	38.9								
32	Tb927.11.1020	ribokinase, putative	1.00	1213	62	20	10	39.3	ANMIAAISVQRPGTQSSYPTLDELPAEVKECKI Carbamidomethyl (C31)	75.1	1.00	1270	94	21	10	36.9	ANMIAAISVQRPGTQSSYPTLDELPAEVKECKI Carbamidomethyl (C31)	62.7
								ANMIAAISVQRPGTQSSYPTLDELPAEVKECKI Deamidated (Q10)- Carbamidomethyl (C31)	56.4							ANMIAAISVQRPGTQSSYPTLDELPAEVKECKI Deamidated (Q10)- Carbamidomethyl (C31)	62.7
								ANMIAAISVQRPGTQSSYPTLDELPAEVKECKI Oxidation (M3)- Carbamidomethyl (C31)	48.9							ANMIAAISVQRPGTQSSYPTLDELPAEVKECKI Deamidated (Q15)- Carbamidomethyl (C31)	62.0
								ANMIAAISVQRPGTQSSYPTLDELPAEVKECKI Oxidation (M3)- Deamidated (Q15)- Carbamidomethyl (C31)	45.3							ANMIAAISVQRPGTQSSYPTLDELPAEVKECKI Deamidated (Q15)- Deamidated (N2)- Oxidation (M3)- Carbamidomethyl (C31)	6.3
								GFGGKGSNQAVCAGR Carbamidomethyl (C12)	68.6							ANMIAAISVQRPGTQSSYPTLDELPAEVKECKI Oxidation (M3)- Carbamidomethyl (C31)	16.5
								GKPPRHLPSITVK	25.2							GFGGKGSNQAVCAGR Carbamidomethyl (C12)	67.2
								KQSDNYNNFFGPK	63.4							KQSDNYNNFFGPK	61.6
								KQSDNYNNFFGPK Deamidated (N7)	59.2							KQSDNYNNFFGPK Deamidated (N5)	57.0
								KQSDNYNNFFGPK Deamidated (Q2)	10.5							KQSDNYNNFFGPK Deamidated (Q2)- Deamidated (N5)	32.4
								LGAHSVVTTLGGNGYVIGEK	80.0							LGAHSVVTTLGGNGYVIGEK	51.3
								LGAHSVVTTLGGNGYVIGEK Deamidated (N13)	61.0							LGAHSVVTTLGGNGYVIGEK Deamidated (N13)	48.9
								LGAHSVVTTLGGNGYVIGEKGKPPR	82.0							LGAHSVVTTLGGNGYVIGEKGKPPR	71.0
								LGAHSVVTTLGGNGYVIGEKGKPPR Deamidated (N13)	67.8							LGAHSVVTTLGGNGYVIGEKGKPPR Deamidated (N13)	57.5
								NGVQVSNVYR	39.0							NGVQVSNVYR Deamidated (N1)	59.7
								NGVQVSNVYR Deamidated (N1)	39.8							QSDNYNNFFGPK	20.8
								NGVQVSNVYR Deamidated (Q4)	50.0							QSDNYNNFFGPK Deamidated (N4)- Deamidated (N6)- Deamidated (N7)	47.0
								QSDNYNNFFGPK	47.8							SFSKGFGGKGSNQAVCAGR Carbamidomethyl (C16)	21.7
								SFSKGFGGKGSNQAVCAGR Carbamidomethyl (C16)	47.2							YIILQNEIPVETTLDVLK	44.5
								YIILQNEIPVETTLDVLKEAHTR	32.2							YIILQNEIPVETTLDVLK Deamidated (Q5)	48.7
								YIILQNEIPVETTLDVLKEAHTR Deamidated (Q5)	29.8							YIILQNEIPVETTLDVLKEAHTR	43.3
																YIILQNEIPVETTLDVLKEAHTR Deamidated (Q5)	30.0
33	Tb927.11.2690	succinyl-coA:3-ketoacid-coenzyme A transferase, mitochondrial precursor, putative	1.00	583	20	9	4	13.8	GPGGAMDLVSCGTR Oxidation (M6)- Carbamidomethyl (C11)	69.7	1.00	766	21	12	6	17.2	GPGGAMDLVSCGTR Carbamidomethyl (C11)	18.4
								NFNVPVGQCGQTVIAEVENLVENGDIDPDEVHLPGVYVDR Carbamidomethyl (C9)	57.1							LVLKEIAEDTTVDQVK	56.2
								NFNVPVGQCGQTVIAEVENLVENGDIDPDEVHLPGVYVDR Carbamidomethyl (C9)- Deamidated (Q11)	91.4							LVLKEIAEDTTVDQVKK	54.6
								NFNVPVGQCGQTVIAEVENLVENGDIDPDEVHLPGVYVDR Deamidated (Q11)- Deamidated (N1)- Deamidated (N3)- Deamidated (N23)- Carbamidomethyl (C9)	74.2							NFNVPVGQCGQTVIAEVENLVENGDIDPDEVHLPGVYVDR Carbamidomethyl (C9)	70.0
								NFNVPVGQCGQTVIAEVENLVENGDIDPDEVHLPGVYVDR Deamidated (Q8)- Carbamidomethyl (C9)	50.8							NFNVPVGQCGQTVIAEVENLVENGDIDPDEVHLPGVYVDR Carbamidomethyl (C9)- Deamidated (N19)	119.5
								NFNVPVGQCGQTVIAEVENLVENGDIDPDEVHLPGVYVDR Deamidated (Q8)- Deamidated (N1)- Deamidated (N3)- Deamidated (N23)- Carbamidomethyl (C9)	95.3							NFNVPVGQCGQTVIAEVENLVENGDIDPDEVHLPGVYVDR Carbamidomethyl (C9)- Deamidated (N23)	116.6
								VVVPERYQTLIEHR	20.6							NFNVPVGQCGQTVIAEVENLVENGDIDPDEVHLPGVYVDR Deamidated (N3)- Carbamidomethyl (C9)	63.5
								VVVPERYQTLIEHR Deamidated (Q8)	27.5							NFNVPVGQCGQTVIAEVENLVENGDIDPDEVHLPGVYVDR Deamidated (Q8)- Deamidated (N1)- Deamidated (N3)- Deamidated (N19)- Carbamidomethyl (C9)	36.4
								YQTLIEHR	39.9							NFNVPVGQCGQTVIAEVENLVENGDIDPDEVHLPGVYVDR Deamidated (Q8)- Deamidated (N1)- Deamidated (N3)- Deamidated (N23)- Carbamidomethyl (C9)	42.2
																NFNVPVGQCGQTVIAEVENLVENGDIDPDEVHLPGVYVDR Deamidated (Q8)- Deamidated (Q11)- Deamidated (N3)- Deamidated (N23)- Carbamidomethyl (C9)	77.4
																VVVPERYQTLIEHR	21.3
																YQTLIEHR	32.4
34	Tb927.11.1450	2-oxoglutarate dehydrogenase E1 component, putative	1.00	813	75	23	21	30.9	AIESGEGAEWCLAEMLAFGATALEGTHVR Carbamidomethyl (C11)	5.8	1.00	1034	59	24	22	34.2	AIESGEGAEWCLAEMLAFGATALEGTHVR Carbamidomethyl (C11)	34.7
								FGLDGAEALVPAVIALMQEASDLGVTSFVQGMPHR	5.3							FGLDGAEALVPAVIALMQEASDLGVTSFVQGMPHR	48.1
								GRLNLLANVK	31.1							GRLNLLANVK	23.3
								HAAVTDLETNKK	38.7							HAAVTDLETNKK	37.2
								HAAVTDLETNKK Deamidated (N10)	34.7							KVLFCTGQIESIVDDR Carbamidomethyl (C5)	59.8
								HLGMAGSSDFLPYVGR	1.3							KVLFCTGQIESIVDDRR Carbamidomethyl (C5)	29.5
								HLGMAGSSDFLPYVGR Oxidation (M4)	39.1							LGDNKYHLGADR	41.9
								KVLFCTGQIESIVDDR Carbamidomethyl (C5)	60.4							LREAYETAQSCPEYTK Carbamidomethyl (C11)	47.6

									LAGEDVERGTFTQR	35.3							NGKVINFDLLCNPSHLEAMNPLVLGK Deamidated (N1)- Carbamidomethyl (C11)	50.8
									LGDNKYHLGADR	32.5							NGKVINFDLLCNPSHLEAMNPLVLGK Deamidated (N1)- Deamidated (N6)- Deamidated (N12)- Carbamidomethyl (C11)	31.5
									LREAYETAQSCPEYTK Carbamidomethyl (C11)	54.0							RGDPKPLVVFFSK	26.8
									RGDPKPLVVFFSK	25.1							SGSPDAPLVQDFTRPVK	34.8
									RVGLHVTTIPSEVKK	32.8							SLRPLVDLYSDTLVEEGVLTKEDVK	42.8
									SLRPLVDLYSDTLVEEGVLTKEDVK	21.0							SLRPLVDLYSDTLVEEGVLTKEDVKAK	33.3
									SLRPLVDLYSDTLVEEGVLTKEDVKAK	26.3							TLPIIAHGDAAISGLGMGHETMGLWDLDNYR	14.4
									VGLHVTTIPSEVK	31.3							TVRQPHRYDQAITAENEVYIEQLLK	31.6
									VGLHVTTIPSEVKK	34.4							TVRQPHRYDQAITAENEVYIEQLLK Deamidated (Q22)	31.1
									VGLHVTTIPSEVKKVHPVVER	32.5							VGLHVTTIPSEVK	49.6
									VIPNLREDAGNASEGYDLAAEKEANLPK	26.4							VGLHVTTIPSEVKK	24.2
									VKPLTDILAEFEGK	21.5							VINFDLLCNPSHLEAMNPLVLGK Carbamidomethyl (C8)	25.1
									VKPLTDILAEFEGKTHR	30.6							VIPNLREDAGNASEGYDLAAEKEANLPK	43.4
									VTAASPSTGYATVHAEEEAEIIRQALA	11.7							VKPLTDILAEFEGK	38.6
									YLQLCSDTDVVPHNFRR Carbamidomethyl (C5)	28.2							VKPLTDILAEFEGKTHR	15.9
																	YLQLCSDTDVVPHNFR Carbamidomethyl (C5)	46.1
35	Tb927.10.14820	mitochondrial carrier protein, putative,ADP/ATP translocase 1, putative	1.00	990	216	21	15	45.6	GAGANILRGIAGAGVLSGVDALKPIYVEWR	44.8	0.99	737	148	13	9	36.2	GAGANILRGIAGAGVLSGVDALKPIYVEWR	29.6
	Tb927.10.14830	mitochondrial carrier protein, putative,ADP/ATP translocase 1, putative							GIAGAGVLSGVDALKPIYVEWR	65.4							GIAGAGVLSGVDALKPIYVEWR	59.9
	Tb927.10.14840	mitochondrial carrier protein, putative,ADP/ATP translocase 1, putative							GIAGAGVLSGVDALKPIYVEWRR	35.5							GIAGAGVLSGVDALKPIYVEWRR	26.9
									LDKPYNGVVDCFRR Carbamidomethyl (C11)	20.8							LDKPYNGVVDCFRR Carbamidomethyl (C11)	11.7
									LDKPYNGVVDCFRR Deamidated (N6)- Carbamidomethyl (C11)	23.7							LDKPYNGVVDCFRR Deamidated (N6)- Carbamidomethyl (C11)	13.2
									LGFLEEFMIGGVAAGLSK	30.6							LGFLEEFMIGGVAAGLSK	43.9
									LGFLEEFMIGGVAAGLSK Oxidation (M8)	26.4							LGFLEEFMIGGVAAGLSK Oxidation (M8)	28.4
									LLVQNQGEMMK Oxidation (M10)	12.1							TISTEGVYPLWR	38.3
									LLVQNQGEMMK Oxidation (M9)- Oxidation (M10)	45.5							YFPTQALNFAFK	33.7
									RTISTEGVYPLWR	23.8							YFPTQALNFAFK Deamidated (Q5)	48.4
									TAAAPIER	22.3							YFPTQALNFAFKDK	79.4
									TAAAPIERVK	27.0							YFPTQALNFAFKDK Deamidated (Q5)	28.7
									TISTEGVYPLWR	53.7							YKNSMDCMLQVIKQEGAASLMR Carbamidomethyl (C7)	62.5
									TISTEGVYPLWRGNLSNVLR	15.8								
									VKLLVQNQGEMMK Oxidation (M11)- Oxidation (M12)	31.8								
									VKLLVQNQGEMMK Oxidation (M12)	13.6								
									YFPTQALNFAFK	47.3								
									YFPTQALNFAFK Deamidated (Q5)	25.7								
									YFPTQALNFAFKDK	59.9								
									YKNSMDCMLQVIKQEGAASLMR Oxidation (M5)- Oxidation (M8)- Oxidation (M21)- Carbamidomethyl (C7)	26.6								
									YKNSMDCMLQVIKQEGAASLMR Oxidation (M5)- Oxidation (M8)- Oxidation (M21)- Carbamidomethyl (C7)- Deamidated (Q10)	22.3								
36	Tb927.10.14550	ATP-dependent DEAD/H RNA helicase, putative	1.00	836	50	16	10	19.6	APAFGEAGHQVGYNNYGGYQQRPR	51.5	1.00	687	52	13	10	23.8	GLDIPNVGVVIQYDMPSNIDDYVHR	27.6
									APAFGEAGHQVGYNNYGGYQQRPR Deamidated (N14)	62.4							HSFLQVGR	30.5
									APAFGEAGHQVGYNNYGGYQQRPR Deamidated (Q10)	86.8							KFTYHTPVR	33.9
									APAFGEAGHQVGYNNYGGYQQRPR Deamidated (Q20)	31.0							NIVDDLIPLLNETNQVISPEVR	52.3
									FLILDEADRMLDMGFEPQIR Oxidation (M10)- Oxidation (M13)	23.5							NIVDDLIPLLNETNQVISPEVR Deamidated (Q15)	19.2
									HSFLQVGR	38.3							QALLTLLRENEGK	29.1
									MIVQGPDSDMPR Oxidation (M1)- Oxidation (M10)	38.8							SGACQVLVATDVASR Carbamidomethyl (C4)	55.4
									MIVQGPDSDMPR Oxidation (M10)	6.0							TASYLIPAINEILLNISNRPPYSPGSHSSPQALILAPTR	50.4
									NIVDDLIPLLNETNQVISPEVR	37.8							TASYLIPAINEILLNISNRPPYSPGSHSSPQALILAPTR Deamidated (N10)	59.5
									NIVDDLIPLLNETNQVISPEVR Deamidated (Q15)	26.2							TASYLIPAINEILLNISNRPPYSPGSHSSPQALILAPTR Deamidated (Q31)	42.8
									NRNIVDDLIPLLNETNQVISPEVR Deamidated (N13)	31.2							VGSTTENITQDVR	55.0
									QTLLYSATFPVEIQR	56.5							VGVAISFFNEK	54.1
									SGACQVLVATDVASR Carbamidomethyl (C4)	63.0							VGVAISFFNEKNR Deamidated (N9)	46.4
									VGVAISFFNEKNR	48.7								
									VGVAISFFNEKNR Deamidated (N9)	43.4								
									VGVAISFFNEKNRNIVDDLIPLLNETNQVISPEVR	25.0								
37	Tb927.10.2090	elongation factor 1-alpha,EF-1-alpha	1.00	1018	179	22	12	43.4	DMRQTVAVGIIK Oxidation (M2)	25.2	0.97	591	54	15	10	42.5	GPTLLEALDMLEPPVRPSDKPLRLPLQDVYK	25.9
	Tb927.10.2100	elongation factor 1-alpha,EF-1-alpha							GPTLLEALDMLEPPVRPSDKPLRLPLQDVYK Oxidation (M10)	6.6							IGGIGTVPVGR	57.5
	Tb927.10.2110	elongation factor 1-alpha							IGGIGTVPVGR	52.9							IGGIGTVPVGRVETGVMKPGDVVTFAPANVTTEVK	34.9
									IGGIGTVPVGRVETGVMKPGDVVTFAPANVTTEVK	36.6							IGGIGTVPVGRVETGVMKPGDVVTFAPANVTTEVK Deamidated (N29)	37.6
									IGGIGTVPVGRVETGVMKPGDVVTFAPANVTTEVK Oxidation (M17)	31.6							IGGIGTVPVGRVETGVMKPGDVVTFAPANVTTEVK Oxidation (M17)	18.6
									IGGIGTVPVGRVETGVMKPGDVVTFAPANVTTEVK Oxidation (M17)- Deamidated (N29)	26.1							IGGIGTVPVGRVETGVMKPGDVVTFAPANVTTEVK Oxidation (M17)- Deamidated (N29)	17.0
									MVPQKPMCVEVFNDYAPLGR Carbamidomethyl (C8)	9.4							MVPQKPMCVEVFNDYAPLGR Oxidation (M7)- Carbamidomethyl (C8)	12.2
									MVPQKPMCVEVFNDYAPLGR Oxidation (M1)- Carbamidomethyl (C8)	42.1							QTVAVGIIK	35.8
									MVPQKPMCVEVFNDYAPLGR Oxidation (M1)- Oxidation (M7)- Carbamidomethyl (C8)	25.4							SGKELEKAPK	40.0
									MVPQKPMCVEVFNDYAPLGR Oxidation (M7)- Carbamidomethyl (C8)	21.9							SIEMHHEQLAEATPGDNVGFNVK	40.4
									QTVAVGIIK	35.2							SIKSGDAAIVR	43.5
									SGDAAIVR	19.2							VETGVMKPGDVVTFAPANVTTEVK	33.8
									SGKELEKAPK	31.3							VETGVMKPGDVVTFAPANVTTEVK Oxidation (M6)	12.9
									SIEMHHEQLAEATPGDNVGFNVK	47.3							VETGVMKPGDVVTFAPANVTTEVK Oxidation (M6)- Deamidated (N18)	7.8
									SIEMHHEQLAEATPGDNVGFNVK Oxidation (M4)	44.7							VGYNVEKVR	43.0
									SIEMHHEQLAEATPGDNVGFNVK Oxidation (M4)- Deamidated (N17)	34.0								
									SIEMHHEQLAEATPGDNVGFNVK Oxidation (M4)- Deamidated (N21)	29.1								
									SIKSGDAAIVR	44.1								
									VETGVMKPGDVVTFAPANVTTEVK	26.9								
									VETGVMKPGDVVTFAPANVTTEVK Oxidation (M6)	41.6								
									VETGVMKPGDVVTFAPANVTTEVK Oxidation (M6)- Deamidated (N18)	30.1								
									VGYNVEKVR	46.0								
38	Tb927.7.7420	ATP synthase alpha chain, mitochondrial precursor,ATP synthase F1, alpha subunit	1.00	1032	79	25	16	23.5	ALKNLLYSCK Carbamidomethyl (C9)	47.6	0.84	247	24	10	8	16.1	ALKNLLYSCKR Carbamidomethyl (C9)	25.8
	Tb927.7.7430	ATP synthase alpha chain, mitochondrial precursor,ATP synthase F1, alpha subunit							ALKNLLYSCKR Carbamidomethyl (C9)	19.5							AVDTMIPIGR	19.1
									AVDTMIPIGR Oxidation (M5)	53.0							EAYPGDVFYLHSR	14.7
									EAYPGDVFYLHSR	24.9							LAADSVGGQQVQTIPMIR	11.2
									ELIVGDRQTGK	28.3							LAADSVGGQQVQTIPMIR Oxidation (M16)	8.9
									GILAEYRK	36.0							LAADSVGGQQVQTIPMIRGAR Oxidation (M16)	6.8
									LAADSVGGQQVQTIPMIR	17.7							SPVNYNLLTGFK	52.1
									LAADSVGGQQVQTIPMIR Oxidation (M16)	41.6							SPVNYNLLTGFK Deamidated (N6)	19.2
									LAADSVGGQQVQTIPMIRGAR Oxidation (M16)	29.9							VDTGAPNIVSR	50.0
									LKGILAEYRK	29.5							VGSSAQNAAMKGVAGK	13.6
									SPVNYNLLTGFK	51.1								
									SPVNYNLLTGFK Deamidated (N4)	22.2								
									SPVNYNLLTGFKAVDTMIPIGR	47.6								
									SPVNYNLLTGFKAVDTMIPIGR Deamidated (N4)- Deamidated (N6)	25.3								
									SPVNYNLLTGFKAVDTMIPIGR Deamidated (N4)- Oxidation (M17)	33.6								
									SPVNYNLLTGFKAVDTMIPIGR Deamidated (N6)- Oxidation (M17)	25.1								
									SPVNYNLLTGFKAVDTMIPIGR Oxidation (M17)	45.8								
									SPVNYNLLTGFKAVDTMIPIGRGQR	44.9								
									SPVNYNLLTGFKAVDTMIPIGRGQR Deamidated (Q24)- Deamidated (N6)	35.4								
									SPVNYNLLTGFKAVDTMIPIGRGQR Oxidation (M17)	44.7								
									VDTGAPNIVSR	38.3								
									VGSSAQNAAMKGVAGK Oxidation (M10)	24.2								
									VGSSAQNAAMKGVAGKLK Oxidation (M10)	43.0								
									VMATGQLLHIPVGAGVLGK	23.7								
									VMATGQLLHIPVGAGVLGK Oxidation (M2)	43.0								
39	Tb927.10.2020	hexokinase	1.00	534	27	12	11	34.8	AAQLSASFCCAPLVK Carbamidomethyl (C9)- Carbamidomethyl (C10)	58.3	1.00	618	38	15	13	42.3	ATIAIDGSVFEKIPSFR	4.2
									FAGMISADRMPGLQFTR Oxidation (M4)- Oxidation (M10)	21.9							ATIAIDGSVFEKIPSFRR	37.1
									FVLPTTKFDLDIDDASLNKGQQALEK Deamidated (Q21)	37.9							FVLPTTKFDLDIDDASLNKGQQALEK	68.8
									FVLPTTKFDLDIDDASLNKGQQALEK Deamidated (Q22)	53.5							GSALTPISMESGNFDSKYR Deamidated (N13)	2.2
									GSALTPISMESGNFDSKYR Oxidation (M9)	47.2							QIMTYLLYEMVEGLEGR	35.9
									IIRDVCR Carbamidomethyl (C6)	20.2							RLNNILEHISIQGNDGETVR	29.3
									RLNNILEHISIQGNDGETVR	20.4							RVLQDNINR	34.2
									RVIVHLSSINCLPAALQTALGNR Carbamidomethyl (C11)	53.2							TVPLGFTFSFPVEQTK	25.3
									VIVHLSSINCLPAALQTALGNR Carbamidomethyl (C10)	29.2							VACKEGAVVDSSTSAFKIPK Carbamidomethyl (C3)	13.2
									VLQDNINRILGPECDVR Carbamidomethyl (C14)	25.5							VCGVDVQSIEDLR Carbamidomethyl (C2)- Deamidated (Q7)	65.3
									YALEGNATDLFDFIASNVK	72.7							VIVHLSSINCLPAALQTALGNR Carbamidomethyl (C10)	28.3

									YALEGNATDLFDFIASNVKK	26.1							YALEGNATDLFDFIASNVK	57.6
																	YALEGNATDLFDFIASNVK Deamidated (N6)	12.5
																	YALEGNATDLFDFIASNVKK	53.2
																	YALEGNATDLFDFIASNVKK Deamidated (N6)	40.9
40	Tb927.10.1510	NOT1	1.00	661	42	18	15	11.0	AFLAVGGNPR	32.1	1.00	601	30	13	12	10.2	AGDPTISASLAFIEQLLK	34.9
									ANRPLQANAEPFKPKDSMVPSGGALAFEAVGPR Oxidation (M18)	19.9							ALEWQAAEEGAK	8.5
									ASPALSNLVEQHVR	58.4							ATAALNTFLASSLKDLLR	86.3
									AVYEEFNNSVPVVGLLTAYVR	32.8							AVYEEFNNSVPVVGLLTAYVR	27.7
									NAQQQVQQSTGSASTSATQGKK	45.1							AWTLLLPVIVKPTDHLVEEYGIIALEQIKPR	49.5
									NAQQQVQQSTGSASTSATQGKK Deamidated (N1)	32.7							FAVATSIVEVAEQLLDKQK	56.2
									QGVVDFAGATLSR	18.7							NAQQQVQQSTGSASTSATQGKK Deamidated (N1)	9.7
									RLVVPTLAPQNQEDAEKIR	31.7							RLVVPTLAPQNQEDAEKIR	37.4
									RPPIVITPETVR	24.8							RPPIVITPETVR	17.1
									RPPIVITPETVRVSEEEASLFGER	28.4							RPPIVITPETVRVSEEEASLFGER	49.9
									SLASNLCTVAIRDALPEPFNVQVGDLISR Carbamidomethyl (C7)	47.8							SLASNLCTVAIRDALPEPFNVQVGDLISR Carbamidomethyl (C7)	69.3
									SLASNLCTVAIRDALPEPFNVQVGDLISR Carbamidomethyl (C7)- Deamidated (N20)	44.9							SLASNLCTVAIRDALPEPFNVQVGDLISR Carbamidomethyl (C7)- Deamidated (N20)	34.9
									SLASNLCTVAIRDALPEPFNVQVGDLISR Deamidated (N5)- Carbamidomethyl (C7)	45.2							TTQQQKEVESLLSDPAVLALSTERR	15.6
									TLEDATVQHYSTGDAK	18.3								
									TTQQQKEVESLLSDPAVLALSTERR	7.9								
									VAAATLHQHDIGTLLSK	38.2								
									VAAATLHQHDIGTLLSKK	24.6								
									VTQELKPTVQR	29.3								
41	Tb927.2.1560	cyclophilin-type peptidyl-prolyl cis-trans isomerase, putative	0.98	742	20	10	6	66.8	AGKHFGPGTLSMANAGR Oxidation (M12)	9.7	0.00	0	0	0	0	0.0		
	Tb927.2.1680	cyclophilin-type peptidyl-prolyl cis-trans isomerase, putative							AGRVEMELFKDVVPK Oxidation (M6)	24.5								
									ALCTGEKGLGR Carbamidomethyl (C3)	39.4								
									IPFYPVNPSNPLVYFDISIGSQK	49.2								
									NTNGSQFFICTAATPWLDGKHVVFGQVTK Deamidated (N3)- Carbamidomethyl (C10)	69.7								
									VIPQFMCQGGDFTSGNGTGGESIYGLKFPDESFAGR Carbamidomethyl (C7)- Deamidated (Q8)	110.5								
									VIPQFMCQGGDFTSGNGTGGESIYGLKFPDESFAGR Carbamidomethyl (C7)- Deamidated (Q8)- Deamidated (N16)	122.3								
									VIPQFMCQGGDFTSGNGTGGESIYGLKFPDESFAGR Deamidated (Q4)- Deamidated (Q8)- Oxidation (M6)- Carbamidomethyl (C7)	97.2								
									VIPQFMCQGGDFTSGNGTGGESIYGLKFPDESFAGR Oxidation (M6)- Carbamidomethyl (C7)	102.3								
									VIPQFMCQGGDFTSGNGTGGESIYGLKFPDESFAGR Oxidation (M6)- Carbamidomethyl (C7)- Deamidated (N16)	56.9								
42	Tb927.3.3580	lipophosphoglycan biosynthetic protein, putative	1.00	680	58	14	12	21.9	AVFLRELISNGSDALDKIR	52.8	1.00	798	52	19	15	22.3	AVFLRELISNGSDALDKIR	0.3
									AVFLRELISNGSDALDKIR Deamidated (N10)	62.4							AVFLRELISNGSDALDKIR Deamidated (N10)	17.6
									DGGVGMTRQELEANLGSLGSSGTKR Oxidation (M6)	11.1							ELISNGSDALDKIR	60.6
									ELISNGSDALDKIR	30.4							ELISNGSDALDKIR Deamidated (N5)	41.9
									FRPLTDALTR	39.2							FRPLTDALTR	35.2
									GVEVLLMTDAIDEYVVGQVHDFANKK Oxidation (M7)	15.8							GKSIPFQAEVSK	11.5
									HPVVQQLLAR	17.1							GTEITLELKPDALDFLSPETVR	43.8
									KLINIATDSAQLDDVTDKQK	43.9							GVEVLLMTDAIDEYVVGQVHDFANK	47.1
									LINIATDSAQLDDVTDKQK	48.9							GVEVLLMTDAIDEYVVGQVHDFANKK	57.8
									LSVDPEQKTLTLR	49.2							HPVVQQLLAR	24.7
									MLDILIHSLYTNR	55.8							HPVVQQLLAR Deamidated (Q6)	14.5
									MLDILIHSLYTNR Oxidation (M1)	47.6							KLINIATDSAQLDDVTDKQK	33.2
									MLYLTTPKEPVNKDGEAPTMDIR Oxidation (M1)- Oxidation (M20)	20.5							LINIATDSAQLDDVTDKQK	58.3
									QYSEFVHFPIR	30.8							LSVDPEQK	21.0
																	LSVDPEQKTLTLR	1.1
																	MLDILIHSLYTNR	49.0
																	QYSEFVHFPIR	33.7
																	QYSEFVHFPIR Deamidated (Q1)	32.5
																	RGEEWDVLNENQPIWTR	32.1
43	Tb927.3.1790	pyruvate dehydrogenase E1 beta subunit, putative	1.00	881	70	17	16	40.8	AAEQLAKSGIEAEVINLR	35.0	1.00	567	35	13	11	46.0	AAIRDDNPVVMLEHELMYGETFKVSDEAMGEDFVIPFGK	80.8
									AKIERPGKDITMIGFSR Oxidation (M12)	25.1							AAIRDDNPVVMLEHELMYGETFKVSDEAMGEDFVIPFGK Oxidation (M29)	37.1
									ATIIQSVKK	47.9							AKIERPGKDITMIGFSR Oxidation (M12)	6.7
									ATIIQSVKKTGR	36.0							ATIIQSVKK	29.5
									DALNSAIDEELSRDKTVFVLGEEVGQYQGAYK	24.2							DALNSAIDEELSRDKTVFVLGEEVGQYQGAYK	52.7
									GLVDKYGTSR	27.6							GLVDKYGTSR	49.6
									NLEVASQPQVSDVLDVAR	62.5							NLEVASQPQVSDVLDVAR	44.6
									NLEVASQPQVSDVLDVARR	27.6							NLEVASQPQVSDVLDVARR	35.0
									SGIEAEVINLR	73.7							NLEVASQPQVSDVLDVARR Deamidated (Q9)	17.3
									SGIEAEVINLR Deamidated (N9)	56.8							SGIEAEVINLR	54.0
									SLRPLDRATIIQSVK	24.2							VFSPYSSEDAR	16.0
									SLRPLDRATIIQSVKK	29.4							VSCADCPTPYSK Carbamidomethyl (C3)- Carbamidomethyl (C6)	28.2
									VFSPYSSEDAR	23.9							VSCADCPTPYSKNLEVASQPQVSDVLDVARR Carbamidomethyl (C3)- Carbamidomethyl (C6)	20.9
									VFSPYSSEDARGMLK Oxidation (M13)	28.3								
									VSCADCPTPYSK Carbamidomethyl (C3)- Carbamidomethyl (C6)	37.0								
									VSCADCPTPYSKNLEVASQPQVSDVLDVAR Carbamidomethyl (C3)- Carbamidomethyl (C6)	59.5								
									VSCADCPTPYSKNLEVASQPQVSDVLDVARR Carbamidomethyl (C3)- Carbamidomethyl (C6)	73.9								
44	Tb927.10.7570	dihydrolipoamide acetyltransferase E2 subunit, putative	1.00	560	39	15	13	42.6	ANMLVPEANSSWQGDFIR Oxidation (M3)	34.9	1.00	549	27	11	10	37.7	ANMLVPEANSSWQGDFIR Oxidation (M3)	3.6
									EHAAPAAAPAAKPVVPVIATTPSTQNYTDIPVTNMR	67.1							EHAAPAAAPAAKPVVPVIATTPSTQNYTDIPVTNMR	59.4
									EHAAPAAAPAAKPVVPVIATTPSTQNYTDIPVTNMR Deamidated (N34)	23.5							EHAAPAAAPAAKPVVPVIATTPSTQNYTDIPVTNMR Deamidated (N26)	41.2
									EHAAPAAAPAAKPVVPVIATTPSTQNYTDIPVTNMR Oxidation (M35)	76.4							FTASFDHR	42.3
									FTASFDHR	42.8							FTASFDHRVVDGAVASQWCK Deamidated (Q17)- Carbamidomethyl (C19)	48.6
									FTASFDHRVVDGAVASQWCK Deamidated (Q17)- Carbamidomethyl (C19)	12.2							HFKDAVENPLSLLL	47.0
									GKISEWVK	44.7							IVRKDVEAAASK	32.6
									GLVDISNEMK Oxidation (M9)	9.2							KTAAELNVSLDTIEGTGGGVGR	36.8
									GLVDISNEMKVLAK Oxidation (M9)	22.5							TVDVSVAVATPTGLITPIIKDAQAR	33.2
									HFKDAVENPLSLLL	40.2							VQEVPSPTQVAPLPAGGKEAGGR	17.2
									IVRKDVEAAASK	41.2							VVDGAVASQWCK Carbamidomethyl (C11)	52.3
									KTAAELNVSLDTIEGTGGGVGR	1.9								
									TVDVSVAVATPTGLITPIIKDAQAR	21.4								
									VQEVPSPTQVAPLPAGGKEAGGR	19.0								
									VVDGAVASQWCK Carbamidomethyl (C11)	28.4								
45	Tb927.5.3400	calcium-translocating P-type ATPase,calcium pump	1.00	813	47	21	16	21.2	ADIGIAMGSGTEVAK Oxidation (M7)	1.0	1.00	353	15	9	9	11.4	EAVLTAVLFSR	70.1
									CIGFAFKPTK Carbamidomethyl (C1)	40.1							FANIKGDSAVNAFR	39.3
									EAVLTAVLFSR	66.1							FNIVSNSVTCEGR Carbamidomethyl (C10)	41.0
									FANIKGDSAVNAFR	52.3							IGEATEAALLVMSEK	49.1
									FANIKGDSAVNAFR Deamidated (N3)	64.5							IIEQLDKISGGANALR Deamidated (N13)	11.3
									GAPEEVLRR	26.9							MQLVQLLKDER	31.8
									IIEQLDKISGGANALR	29.3							QVSSPLEQDGALTK	23.4
									IIEQLDKISGGANALR Deamidated (N13)	21.9							TAVVLRDGDIK	17.3
									IIEQLDKISGGANALR Deamidated (Q4)- Deamidated (N13)	12.1							VVELHSTTLR	38.2
									KADIGIAMGSGTEVAK Oxidation (M8)	31.5								
									LGLLSSTADTTGLSYTGQELDAMTPAQKR Oxidation (M23)	31.3								
									MLPENLPTDPAAMTPAAVAAALRVDTK Oxidation (M1)	8.3								
									MLPENLPTDPAAMTPAAVAAALRVDTK Oxidation (M1)- Oxidation (M13)	22.3								
									MVLADDNFATVVK Oxidation (M1)	63.7								
									QVSSPLEQDGALTK	44.4								
									SASKMVLADDNFATVVK Oxidation (M5)	37.2								
									STHVMQDNGAVVQLNATHR Oxidation (M5)	17.7								
									STHVMQDNGAVVQLNATHR Oxidation (M5)- Deamidated (Q6)- Deamidated (N8)	31.2								
									TAVVLRDGDIK	28.6								
									VGLSSNEVEER	37.7								
									VVELHSTTLR	39.2								
46	Tb927.8.3750	nucleolar protein, putative	1.00	806	45	17	13	41.8	AQCGLGHAFSR Carbamidomethyl (C3)	58.9	1.00	435	27	10	9	38.7	AQCGLGHAFSR Carbamidomethyl (C3)	57.7
									LISKAGSLTNLAK	41.2							LLPEHQEKDILR	48.0
									LLPEHQEKDILR	43.1							LLSFAPYQSPENALEDAVCINESIVSPFLNNFLTSVLSK Carbamidomethyl (C19)	28.4
									LLSFAPYQSPENALEDAVCINESIVSPFLNNFLTSVLSK Carbamidomethyl (C19)	23.8							LNFFDTGNKPPSNR Deamidated (N2)- Deamidated (N8)	7.6
									LNFFDTGNKPPSNR	59.6							SDNMIIQSSALTEHMDKGVNLLGMR	5.8

									LNFFDTGNKPPSNRVAMEEALEQYKK	18.7							SPASTIQILGAEK	69.5
									MMLVAPNLTELMGQNIGAK	7.4							STLEERDTEEVTQQIAEILEGDEALAAR	37.8
									SPASTIQILGAEK	83.6							STLEERDTEEVTQQIAEILEGDEALAAR Deamidated (Q14)	34.1
									SPASTIQILGAEK Deamidated (Q7)	59.5							TLYLLYEGPTGYALFK	47.4
									STLEERDTEEVTQQIAEILEGDEALAAR	43.7							VLTTEEIGAGDVALQKDLQTFATFSPWVK	10.3
									STLEERDTEEVTQQIAEILEGDEALAAR Deamidated (Q13)- Deamidated (Q14)	10.4								
									STLEERDTEEVTQQIAEILEGDEALAAR Deamidated (Q14)	57.1								
									STLEERDTEEVTQQIAEILEGDEALAARVYEK	30.9								
									VALLIGSR	34.9								
									VAMEEALEQYKK Oxidation (M3)	52.5								
									VLTTEEIGAGDVALQKDLQTFATFSPWVK	36.5								
									VLTTEEIGAGDVALQKDLQTFATFSPWVK Deamidated (Q19)	40.1								
47	Tb927.1.4100	cytochrome oxidase subunit IV	1.00	656	45	19	11	41.9	ANELAEDNRLDILPGGSPNSLR	13.7	1.00	729	64	18	9	39.7	AGGDERVENAMIAR	17.5
									ANELAEDNRLDILPGGSPNSLR Deamidated (N19)	13.6							ANELAEDNRLDILPGGSPNSLR	39.4
									ANELAEDNRLDILPGGSPNSLREK	24.8							ANELAEDNRLDILPGGSPNSLR Deamidated (N19)	35.8
									ANELAEDNRLDILPGGSPNSLREK Deamidated (N19)	59.5							ANELAEDNRLDILPGGSPNSLR Deamidated (N2)- Deamidated (N19)	25.4
									ANELAEDNRLDILPGGSPNSLREK Deamidated (N2)	66.5							ANELAEDNRLDILPGGSPNSLR Deamidated (N8)	26.3
									DLREVVQELLDDVDPSR	4.8							ANELAEDNRLDILPGGSPNSLREK	26.9
									DLREVVQELLDDVDPSRHITSEAIK	20.1							ANELAEDNRLDILPGGSPNSLREK Deamidated (N19)	34.5
									FRLEKQSNTVAR	27.1							ANELAEDNRLDILPGGSPNSLREK Deamidated (N8)- Deamidated (N19)	31.8
									FRLEKQSNTVAR Deamidated (Q6)	18.9							DLREVVQELLDDVDPSR	49.6
									FTGEPPSWMR Oxidation (M9)	24.7							DLREVVQELLDDVDPSRHITSEAIK	23.2
									IFDEEHVQAEMR Oxidation (M11)	20.0							IFDEEHVQAEMR	40.6
									LTAWLPEKYHIVYVDDFQTVAANDSCAR Carbamidomethyl (C26)	58.1							IFDEEHVQAEMR Oxidation (M11)	22.4
									LTAWLPEKYHIVYVDDFQTVAANDSCAR Deamidated (Q18)- Carbamidomethyl (C26)	34.4							LTAWLPEKYHIVYVDDFQTVAANDSCAR Carbamidomethyl (C26)	37.6
									TRWNVNTELHPADRAEIASR	13.0							LTAWLPEKYHIVYVDDFQTVAANDSCAR Deamidated (N23)- Carbamidomethyl (C26)	21.6
									VVHEYNGDERILDIYSR	42.5							LTAWLPEKYHIVYVDDFQTVAANDSCAR Deamidated (Q18)- Carbamidomethyl (C26)	28.0
									VVHEYNGDERILDIYSR Deamidated (N6)	20.7							TRWNVNTELHPADRAEIASR	28.1
									WNVNTELHPADRAEIASR	29.4							VVHEYNGDERILDIYSR	24.6
									WNVNTELHPADRAEIASR Deamidated (N2)	18.3							VVHEYNGDERILDIYSR Deamidated (N6)	29.7
									WNVNTELHPADRAEIASR Deamidated (N4)	36.7								
48	Tb927.8.2540	3-ketoacyl-CoA thiolase, putative	1.00	671	38	11	10	33.3	KVTLDFCTQASDKNPNFLGNEIYKPFLR Carbamidomethyl (C7)	47.0	1.00	341	26	7	5	22.4	KVTLDFCTQASDKNPNFLGNEIYKPFLR Carbamidomethyl (C7)- Deamidated (N14)	43.9
									LGLSPNDNRLVEIKSLASAAGNLYEDSPDLTR	33.3							KVTLDFCTQASDKNPNFLGNEIYKPFLR Carbamidomethyl (C7)- Deamidated (Q9)	43.2
									QLDDFTFPCLFAR Carbamidomethyl (C9)	46.3							LGLSPNDNRLVEIK	13.1
									QRQLDDFTFPCLFAR Carbamidomethyl (C11)	15.7							SGATALDGRIPVNTGGGLLSFGHPVGATGVK	47.0
									SGATALDGRIPVNTGGGLLSFGHPVGATGVKQVLEVYR	19.7							SGATALDGRIPVNTGGGLLSFGHPVGATGVKQVLEVYR	19.0
									SGATALDGRIPVNTGGGLLSFGHPVGATGVKQVLEVYR Deamidated (N13)	42.9							SLASAAGNLYEDSPDLTR	64.8
									SLASAAGNLYEDSPDLTR	81.4							SLASAAGNLYEDSPDLTR Deamidated (N8)	57.1
									SLASAAGNLYEDSPDLTRMTTSMVAAR Oxidation (M19)- Oxidation (M23)	57.2								
									TTDCSQVSDGGAAVILASEEGLQK Carbamidomethyl (C4)	62.5								
									TTDCSQVSDGGAAVILASEEGLQKLGLSPNDNR Carbamidomethyl (C4)	46.1								
									TTDCSQVSDGGAAVILASEEGLQKLGLSPNDNRLVEIK Carbamidomethyl (C4)	82.5								
49	Tb927.8.5640	hypothetical protein, conserved	1.00	726	42	15	12	49.6	AFNVPPALLENSCRPAECLFAK Carbamidomethyl (C13)- Carbamidomethyl (C18)	31.3	1.00	890	43	15	10	48.1	AFNVPPALLENSCRPAECLFAK Carbamidomethyl (C13)- Carbamidomethyl (C18)	34.6
									AFNVPPALLENSCRPAECLFAKMDLAR Deamidated (N11)- Carbamidomethyl (C13)- Carbamidomethyl (C18)- Oxidation (M23)	9.7							AFNVPPALLENSCRPAECLFAK Deamidated (N3)- Carbamidomethyl (C13)- Carbamidomethyl (C18)	24.8
									DIGASSFDTNIFNDIANILR	24.9							AFNVPPALLENSCRPAECLFAK Deamidated (N3)- Deamidated (N11)- Carbamidomethyl (C13)- Carbamidomethyl (C18)	18.3
									FAPLIATIGSEDVTK	82.9							DIGASSFDTNIFNDIANILR	75.4
									GLSAFKQDYLDNQKVK	39.9							FAPLIATIGSEDVTK	73.1
									HRTPLDNGKLLR Deamidated (N7)	24.8							IADETLEVISR	35.6
									IADETLEVISR	77.9							ICGERNPYALR Carbamidomethyl (C2)	19.2
									ICGERNPYALR Carbamidomethyl (C2)	25.4							ILNAAVFNDGPLDDSALLFTLLEYPERGEVSVATKPVEK	122.0
									ILNAAVFNDGPLDDSALLFTLLEYPERGEVSVATKPVEK	44.2							ILNAAVFNDGPLDDSALLFTLLEYPERGEVSVATKPVEK Deamidated (N3)	37.2
									ILNAAVFNDGPLDDSALLFTLLEYPERGEVSVATKPVEK Deamidated (N3)- Deamidated (N8)	54.0							ILNAAVFNDGPLDDSALLFTLLEYPERGEVSVATKPVEK Deamidated (N3)- Deamidated (N8)	117.4
									ILNAAVFNDGPLDDSALLFTLLEYPERGEVSVATKPVEK Deamidated (N8)	64.2							ILNAAVFNDGPLDDSALLFTLLEYPERGEVSVATKPVEK Deamidated (N8)	111.4
									SRFAPLIATIGSEDVTK	44.5							LSLQVLEDMSLVGVPFDDITTK	31.4
									SVSTSAVAPPSR	45.2							NVPPVK	4.7
									TPLDNGKLLR	35.1							SRFAPLIATIGSEDVTK	35.2
									TPLDNGKLLR Deamidated (N5)	30.3							SVSTSAVAPPSR	19.2
50	Tb927.10.1170	intraflagellar transport protein IFT172, putative	1.00	540	41	15	14	12.5	AAHILESQTVGSDNDETVKGFYKNIAR	35.7	1.00	600	28	12	12	10.4	AAHILESQTVGSDNDETVKGFYK	43.9
									AVPDRVVALEEEWGDYLVSQK	14.0							AVPDRVVALEEEWGDYLVSQK	21.7
									AYIANNDWENALR	18.9							AYIANNDWENALR	57.3
									GWPDLANKR	25.3							GWPDLANKR	17.7
									HISPELEDYVKR	15.1							HISPELEDYVKR	23.6
									HLSQQQLVALFVDQAK	22.8							HLSQQQLVALFVDQAK	66.0
									ILAQLPLRPDSDLKLER	20.8							ILAQLPLRPDSDLKLER	22.1
									LVQAYRPDFLSK	11.9							KFDLAEYLAK	41.3
									NSGQDAIAFLYLNR	55.9							LVQAYRPDFLSK	39.2
									SSGGATDGYDHYAVR	51.5							NSGQDAIAFLYLNR	65.3
									TQAICWSPNNKR Carbamidomethyl (C5)	29.5							SSGGATDGYDHYAVR	81.6
									TQAICWSPNNKR Carbamidomethyl (C5)- Deamidated (N9)	32.6							VAEGYDQTAIPSILQAQGR	51.6
									VAEGYDQTAIPSILQAQGR	36.7								
									VAKEYYPDMLGEIAKR Oxidation (M9)	15.6								
									VNSLNQINELAVSEAR	53.2								
51	Tb927.10.12700	pyruvate dehydrogenase E1 alpha subunit, putative	1.00	791	50	18	13	45.2	AAYQPQMYR Oxidation (M7)	19.8	1.00	557	35	11	7	27.3	GGTIEELFAELFGR	47.4
									GGTIEELFAELFGR	44.7							GPVVLEMDSYR	42.6
									GGTIEELFAELFGREGGCSK Carbamidomethyl (C18)	25.8							QAITPLKELFTDIYCGEQYEHR Carbamidomethyl (C15)	41.0
									IPVIFCCENNHYGMGTREDR Carbamidomethyl (C6)- Carbamidomethyl (C7)- Deamidated (N10)- Oxidation (M14)	31.4							QAITPLKELFTDIYCGEQYEHR Deamidated (Q1)- Carbamidomethyl (C15)	55.0
									IPVIFCCENNHYGMGTREDR Carbamidomethyl (C6)- Carbamidomethyl (C7)- Oxidation (M14)	45.9							QAITPLKELFTDIYCGEQYEHRTTQGTVYAKP Carbamidomethyl (C15)	35.1
									IRGFCHLCIGQEAIPVGMENVLSR Carbamidomethyl (C5)- Carbamidomethyl (C8)- Oxidation (M18)- Deamidated (N20)	31.8							TVPLNPHVPFK	20.7
									QAITPLKELFTDIYCGEQYEHR Carbamidomethyl (C15)	51.8							VKENFYGGNGIVGAQAPLGAGLAWR	40.7
									QAITPLKELFTDIYCGEQYEHRTTQGTVYAKP Carbamidomethyl (C15)	9.7							VKENFYGGNGIVGAQAPLGAGLAWR Deamidated (N4)	95.9
									RGDYIPGLR	27.0							VKENFYGGNGIVGAQAPLGAGLAWR Deamidated (N9)	36.1
									TTQGTVYAKP	24.2							VKENFYGGNGIVGAQAPLGAGLAWR Deamidated (Q15)- Deamidated (N9)	11.3
									TVPLNPHVPFK	32.1							WAKEWCLAGK Carbamidomethyl (C6)	34.5
									VKENFYGGNGIVGAQAPLGAGLAWR	63.9								
									VKENFYGGNGIVGAQAPLGAGLAWR Deamidated (N4)	52.2								
									VKENFYGGNGIVGAQAPLGAGLAWR Deamidated (N9)	50.4								
									VKENFYGGNGIVGAQAPLGAGLAWR Deamidated (Q15)	85.9								
									VKENFYGGNGIVGAQAPLGAGLAWR Deamidated (Q15)- Deamidated (N9)	7.5								
									WAKEWCLAGK Carbamidomethyl (C6)	24.7								
									WAKEWCLAGKGPVVLEMDSYR Carbamidomethyl (C6)- Oxidation (M17)	36.6								
52	Tb927.10.16120	inosine-5'-monophosphate dehydrogenase,IMP dehydrogenase	1.00	661	41	14	11	42.1	ALAVGANVAMLGSMIAGTSETPGEYFFKDGMR Oxidation (M10)- Oxidation (M14)- Oxidation (M31)	88.6	1.00	339	25	9	7	28.2	AMALMGGIGVIHNNCTVEQQAR Carbamidomethyl (C15)	36.7
									AMALMGGIGVIHNNCTVEQQAR Oxidation (M2)- Oxidation (M5)- Carbamidomethyl (C15)	38.3							GVPCVADGGLRNVGDVCK Carbamidomethyl (C4)- Carbamidomethyl (C17)	38.3
									ARDYPNSSLDRNGHLLCAAATSTR Carbamidomethyl (C17)	39.1							NLIDAGADSLR	22.8
									ARDYPNSSLDRNGHLLCAAATSTR Deamidated (N6)- Carbamidomethyl (C17)	28.9							SLTAQSEGAVHSLHHYER	6.0
									ARDYPNSSLDRNGHLLCAAATSTR Deamidated (N6)- Deamidated (N12)- Carbamidomethyl (C17)	30.7							SVSPDVPVSTIR	27.2
									GVPCVADGGLR Carbamidomethyl (C4)	17.2							TYPHLEVVAGNVVTQDQAK	32.3
									GVPCVADGGLRNVGDVCK Carbamidomethyl (C4)- Carbamidomethyl (C17)	47.1							YLSENETLQVAQGVAGAVLDKGSVLK	67.8
									HGYLPVLNDKDEVVCLCSRR Carbamidomethyl (C15)- Carbamidomethyl (C17)	32.7							YLSENETLQVAQGVAGAVLDKGSVLK Deamidated (N5)	43.5
									NLIDAGADSLR	37.9							YLSENETLQVAQGVAGAVLDKGSVLK Deamidated (Q9)	23.3
									SLTAQSEGAVHSLHHYER	37.8								
									SVSPDVPVSTIR	47.9								
									YAASRGVPCVADGGLR Carbamidomethyl (C9)	5.4								
									YLSENETLQVAQGVAGAVLDKGSVLK	52.5								
									YLSENETLQVAQGVAGAVLDKGSVLK Deamidated (Q12)	50.2								
53	Tb927.9.11580	Gim5A protein,glycosomal membrane protein	1.00	780	185	15	7	36.6	ALSPNPLHASNTVR	54.2	1.00	515	60	9	6	36.6	ALSPNPLHASNTVR	44.4
	Tb927.9.11600	Gim5B protein,glycosomal membrane protein							ALSPNPLHASNTVR Deamidated (N5)	15.9							ALSPNPLHASNTVR Deamidated (N5)	24.2
									ALSPNPLHASNTVRGLLGLIASVCEFY Carbamidomethyl (C24)	22.5							DKVMAIVQFLPMALEGPAR	24.6
									DKVMAIVQFLPMALEGPAR Oxidation (M12)	29.8							GLLGLIASVCEFY Carbamidomethyl (C10)	39.8
									DKVMAIVQFLPMALEGPAR Oxidation (M4)- Oxidation (M12)	29.3							LSLLANALSKPTLTSLSKPTGDMVASR	43.3

									GLLGLIASVCEFY Carbamidomethyl (C10)	35.1							LSLLANALSKPTLTSLSKPTGDMVASR Oxidation (M23)	31.0
									LSLLANALSKPTLTSLSKPTGDMVASR	43.6							TAGCESLALSLGNLAR Carbamidomethyl (C4)	54.5
									LSLLANALSKPTLTSLSKPTGDMVASR Deamidated (N6)- Oxidation (M23)	26.0							TAGCESLALSLGNLAR Carbamidomethyl (C4)- Deamidated (N13)	31.0
									LSLLANALSKPTLTSLSKPTGDMVASR Oxidation (M23)	39.3							VMAIVQFLPMALEGPAR	49.4
									TAGCESLALSLGNLAR Carbamidomethyl (C4)	42.4								
									TAGCESLALSLGNLAR Carbamidomethyl (C4)- Deamidated (N13)	19.5								
									VMAIVQFLPMALEGPAR	54.6								
									VMAIVQFLPMALEGPAR Oxidation (M10)	53.4								
									VMAIVQFLPMALEGPAR Oxidation (M2)	25.7								
									VMAIVQFLPMALEGPAR Oxidation (M2)- Oxidation (M10)	49.7								
54	Tb927.10.2010	hexokinase	0.99	407	21	10	9	30.6	AAQLSASFCCAPLVK Carbamidomethyl (C9)- Carbamidomethyl (C10)	58.3	0.98	410	25	10	10	34.0	ATIAIDGSVFEKIPSFR	4.2
									FAGMISADRMPGLQFTR Oxidation (M4)- Oxidation (M10)	21.9							ATIAIDGSVFEKIPSFRR	37.1
									FVLPTTKFDLDIDDASLNKGQQALEK Deamidated (Q21)	37.9							FVLPTTKFDLDIDDASLNKGQQALEK	68.8
									FVLPTTKFDLDIDDASLNKGQQALEK Deamidated (Q22)	53.5							QIMTYLLYEMVEGLEGR	35.9
									GSALTPINMESGNFDSKYR Oxidation (M9)	36.1							RLNNILEHISIQGNDGETVR	29.3
									IIRDVCR Carbamidomethyl (C6)	20.2							RVLQDNINR	34.2
									RLNNILEHISIQGNDGETVR	20.4							TVPLGFTFSFPVEQTK	25.3
									RVIVHLSSINCLPAALQTALGNR Carbamidomethyl (C11)	53.2							VACKEGAVVDSSTSAFKIPK Carbamidomethyl (C3)	13.2
									VIVHLSSINCLPAALQTALGNR Carbamidomethyl (C10)	29.2							VCGVDVQSIEDLR Carbamidomethyl (C2)- Deamidated (Q7)	65.3
									VLQDNINRILGPECDVR Carbamidomethyl (C14)	25.5							VIVHLSSINCLPAALQTALGNR Carbamidomethyl (C10)	28.3
55	Tb927.8.1740	hypothetical protein, conserved	1.00	350	26	9	8	17.6	AAELTTAKSPVTAPSQR	53.7	1.00	391	15	8	5	16.0	ARPDFVAALLTQGR	50.2
									AISAPSDVPR	33.4							LVDVLLFQHFGAEPAITDTNDNNGIGPAATK Deamidated (N22)- Deamidated (N23)	62.8
									ARPDFVAALLTQGR	50.8							LVDVLLFQHFGAEPAITDTNDNNGIGPAATK Deamidated (N23)	52.5
									LVDVLLFQHFGAEPAITDTNDNNGIGPAATK Deamidated (N22)- Deamidated (N23)	7.5							LVDVLLFQHFGAEPAITDTNDNNGIGPAATKK Deamidated (N20)	55.9
									LVDVLLFQHFGAEPAITDTNDNNGIGPAATKK Deamidated (N20)	23.7							LVDVLLFQHFGAEPAITDTNDNNGIGPAATKK Deamidated (N22)- Deamidated (N23)	38.3
									LVDVLLFQHFGAEPAITDTNDNNGIGPAATKK Deamidated (N23)	29.5							LVDVLLFQHFGAEPAITDTNDNNGIGPAATKK Deamidated (N23)	18.8
									SPVTAPSQR	32.5							TALLSAGNNSGTIHPVELMNAQPVRK Deamidated (N8)	24.6
									SSGKAAELTTAKSPVTAPSQR	39.6							YIAPLLLHTESGLQGAAGSVSK	52.6
									TALLSAGNNSGTIHPVELMNAQPVRK Oxidation (M19)	44.5								
56	Tb927.8.6110	hypothetical protein, conserved	1.00	414	31	10	10	40.9	AAGSASDYRGPDFR	19.2	1.00	237	19	7	7	21.2	AAGSASDYRGPDFR	11.3
									DAFTMTNKVAK Oxidation (M5)	25.6							ALKELGMPALSR Oxidation (M7)	12.6
									GLHTGSLKDDFAADKR	22.6							GLHTGSLKDDFAADKR	28.7
									HACLGGVYALKGAAR Carbamidomethyl (C3)	21.0							IGTRNEAAFQDIGIEYYR	37.6
									IGTRNEAAFQDIGIEYYR	49.4							INLSEALGNSINLTEEQYK	64.9
									KIDITGKPMVMIGYGSGDASEAIPITPVKGWEEAAAK Oxidation (M9)- Oxidation (M11)	47.9							LILNYEVDPSR Deamidated (N4)	7.2
									LLELNTK	25.4							STAETHKTFFEELCGK Carbamidomethyl (C14)	23.1
									VLRSEKVFADFLGSK	51.3								
									VSNVVGDSFRVPSNNENAYTMAASAVLR Oxidation (M21)	59.5								
									YAEVDGGDDKLAIVVASDIAEYALGSTGEQTQGAGSVAMLVQK Oxidation (M39)	19.2								
57	Tb927.10.2560	mitochondrial malate dehydrogenase	1.00	521	37	12	10	44.0	AGVFDPARLFGITTLDVVR	35.2	1.00	737	69	16	10	51.3	AGVFDPARLFGITTLDVVR	25.8
									ALIGVVSNPVNSVVPIASEVLK	38.2							ALIGVVSNPVNSVVPIASEVLK	6.5
									ALIGVVSNPVNSVVPIASEVLK Deamidated (N8)	3.6							ALIGVVSNPVNSVVPIASEVLK Deamidated (N11)	18.2
									ALIGVVSNPVNSVVPIASEVLKK	28.7							ALIGVVSNPVNSVVPIASEVLK Deamidated (N8)	8.8
									ALIGVVSNPVNSVVPIASEVLKK Deamidated (N11)	47.4							ALIGVVSNPVNSVVPIASEVLKK	32.7
									ARTFVAEAAGK	34.6							IHKPVFDAYEQSLLEK	34.9
									AVDGADVVIIPAGTPR	27.3							LFGITTLDVVR	41.2
									GGVEKIHKPVFDAYEQSLLEK	13.2							SPYDVNVQVVGGHSGPTIIPLLSQAGVSLTEEQVK	60.1
									IHKPVFDAYEQSLLEK	32.7							SPYDVNVQVVGGHSGPTIIPLLSQAGVSLTEEQVK Deamidated (Q8)	28.4
									LFGITTLDVVR	33.1							TFVAEAAGKSPYDVNVQVVGGHSGPTIIPLLSQAGVSLTEEQVK	48.7
									TNPLVSHLSCYDIHGVTGVAADLSHICSPAK Carbamidomethyl (C10)- Carbamidomethyl (C27)	45.7							TFVAEAAGKSPYDVNVQVVGGHSGPTIIPLLSQAGVSLTEEQVK Deamidated (Q42)	61.4
									VAVLGAAGGIGQPLSLILK	64.5							TNPLVSHLSCYDIHGVTGVAADLSHICSPAK Carbamidomethyl (C10)- Carbamidomethyl (C27)	63.9
																	TNPLVSHLSCYDIHGVTGVAADLSHICSPAK Deamidated (N2)- Carbamidomethyl (C10)- Carbamidomethyl (C27)	30.6
																	VAVLGAAGGIGQPLSLILK	45.0
																	VAVLGAAGGIGQPLSLILK Deamidated (Q12)	4.0
																	VTGHLKDELHK	27.5
58	Tb927.10.4560	elongation factor 2	1.00	506	58	14	12	20.3	AYLPVAESFGFTADLR	16.4	0.99	444	20	10	9	16.9	ETVTDVSSIQCLSK Carbamidomethyl (C11)	47.4
	Tb927.10.4570	elongation factor 2							FLADKFEWDVAEAR	9.1							FLADKFEWDVAEAR	47.4
									FLPAAETLLQMIVAHLPSPK Oxidation (M11)	30.0							GTVAIGSGLQAWAFSVTRFAKMYASK Oxidation (M22)	40.4
									GLKPDIPGLDTFLDKL	19.8							GVIIGEENRPGTPIYNVR	20.8
									GVIIGEENRPGTPIYNVR	24.4							GVIIGEENRPGTPIYNVR Deamidated (N8)	20.8
									KGLKPDIPGLDTFLDKL	32.2							KGLKPDIPGLDTFLDKL	43.9
									RGVIIGEENRPGTPIYNVR	30.4							RGVIIGEENRPGTPIYNVR	22.8
									RVFYACCLTATPR Carbamidomethyl (C6)- Carbamidomethyl (C7)	29.8							STLSDSLVGAAGIIK	59.1
									SATITDDGESPHPLRDMKYSVSPVVR Oxidation (M17)	25.9							VAVEAKNPSDLPK	47.5
									VAVEAKNPSDLPK	41.9							YQEAVEDMPCGNVVGLVGVDKYIVK Carbamidomethyl (C10)	35.3
									VAVEAKNPSDLPK Deamidated (N7)	52.6								
									VNFTVDEVR	48.8								
									YQEAVEDMPCGNVVGLVGVDKYIVK Carbamidomethyl (C10)	12.4								
									YQEAVEDMPCGNVVGLVGVDKYIVK Oxidation (M8)- Carbamidomethyl (C10)	21.9								
59	Tb927.6.2230	hypothetical protein, conserved	1.00	315	18	8	7	12.7	ARVDPSTQDEKSVTLLLNCAATFR Carbamidomethyl (C19)	28.1	1.00	122	5	3	3	10.3	AIATVGFHTDEQIGELLEAVSYYRDPQK	38.9
									KTTMENFLSAVSGIPQAQKVVEVLSHQTR Oxidation (M4)	30.2							ARVDPSTQDEKSVTLLLNCAATFR Carbamidomethyl (C19)	26.2
									LEDSVGSAPPRELLK	25.0							TTMENFLSAVSGIPQAQKVVEVLSHQTR	41.7
									NRANDGKIIALSEQR	31.4								
									TTMENFLSAVSGIPQAQKVVEVLSHQTR Oxidation (M3)	44.2								
									TTMENFLSAVSGIPQAQKVVEVLSHQTR Oxidation (M3)- Deamidated (N5)	39.2								
									VAPFADAASVTTIMR Oxidation (M14)	42.6								
									VVEVLSHQTR	41.2								
60	Tb927.10.8940	hypothetical protein, conserved	1.00	277	24	9	9	39.7	AAYNNFGSGVSAMFGFQPTGLVTAIENETQGPVVTRR Deamidated (N4)- Deamidated (N5)- Oxidation (M13)	20.6	1.00	152	11	5	4	14.1	ILASNCPPPPPPPPGSPSHR Carbamidomethyl (C6)	36.7
									HKDSGNIICPEPLPK Carbamidomethyl (C9)	20.3							ILASNCPPPPPPPPGSPSHR Deamidated (N5)- Carbamidomethyl (C6)	13.5
									ILASNCPPPPPPPPGSPSHR Carbamidomethyl (C6)	59.1							SGEFDIFDPTPLPQQPTK	26.5
									KMLAPGNPPPR Oxidation (M2)	32.8							TKYRDTSEIILGPSKPSQIK	20.7
									NRSNESVDVLNLGQYSSEQLNEVER	9.2							YRDTSEIILGPSKPSQIK	16.7
									SGEFDIFDPTPLPQQPTK	24.8								
									SNESVDVLNLGQYSSEQLNEVER	33.7								
									SQEITAHGGR Deamidated (Q2)	13.0								
									VHTPANATHDIFGTGQGVEDIEPVHCK Carbamidomethyl (C26)	22.7								
61	Tb927.10.7020	acid phosphatase, putative	1.00	370	31	8	8	28.5	ILQSAQAVAQGLFPDGYGPGGYLPR	46.7	1.00	418	25	11	9	33.1	GMKEEYELGEYLR Oxidation (M2)	11.1
									LILWTPKDGTATVAIPHCR Carbamidomethyl (C18)	42.1							ILQSAQAVAQGLFPDGYGPGGYLPR	61.4
									LVPEKSVGEEIVPSR	33.6							LILWTPKDGTATVAIPHCR Carbamidomethyl (C18)	28.8
									NAYRDFIGPR	45.3							NAYRDFIGPR	39.6
									QPQFVPVFSDMDTHEYLLDDVPCFR Oxidation (M11)- Carbamidomethyl (C23)	15.6							NAYRDFIGPR Deamidated (N1)	20.7
									RKYNVGAEDLTAR	18.1							QPQFVPVFSDMDTHEYLLDDVPCFR Carbamidomethyl (C23)	18.0
									TPQLCTLKELQDIYSAR Carbamidomethyl (C5)	51.1							QPQFVPVFSDMDTHEYLLDDVPCFR Oxidation (M11)- Carbamidomethyl (C23)	18.9
									YNVGAEDLTAR	39.8							RGDEKQNLVLTK	35.7
																	RKYNVGAEDLTAR	24.5
																	SVGEEIVPSR	38.6
																	TPQLCTLKELQDIYSAR Carbamidomethyl (C5)	68.4
62	Tb927.6.4440	hypothetical protein, conserved	1.00	809	61	15	12	35.2	AAPFVVGASQTTVNVFR	34.9	1.00	234	18	6	6	23.0	AAPFVVGASQTTVNVFR Deamidated (Q10)	13.7
									AAPFVVGASQTTVNVFR Deamidated (N14)	28.2							AAPFVVGASQTTVNVFRQK	2.3
									AAPFVVGASQTTVNVFRQK	38.8							TWASLASIATK	44.5
									APSEPVGERLMFSIDHAVTDEEILTALGPLAAR	26.6							TWASLASIATKGEQRPSIK	41.2
									APSEPVGERLMFSIDHAVTDEEILTALGPLAAR Oxidation (M11)	56.1							VSPSTAAPAVTEAAVDAPTGPK	52.1
									GRVSPSTAAPAVTEAAVDAPTGPK	36.8							VVANESTANKVAPKPASPPTK Deamidated (N4)	22.9
									LMFSIDHAVTDEEILTALGPLAAR	14.9								
									TWASLASIATK	45.8								
									TWASLASIATKGEQRPSIK	34.9								
									VSPSTAAPAVTEAAVDAPTGPK	60.3								

									VSPSTAAPAVTEAAVDAPTGPKTWASLASIATK	32.8								
									VSPSTAAPAVTEAAVDAPTGPKTWASLASIATKGEQRPSIK	56.7								
									VVANESTANKVAPKPASPPTK	33.6								
									VVANESTANKVAPKPASPPTKQAPQSDSR	47.8								
									VVANESTANKVAPKPASPPTKQAPQSDSR Deamidated (N4)	36.5								
63	Tb927.8.6060	2-amino-3-ketobutyrate coenzyme A ligase, putative,glycine acetyltransferase, putative	1.00	504	31	11	10	32.4	ALGGASGGLSSGCKEIVDLQR Carbamidomethyl (C13)	67.0	1.00	462	42	10	7	26.5	ALGGASGGLSSGCKEIVDLQR Carbamidomethyl (C13)	52.1
									DALDSHGYGLASVR	15.5							EAAAAQLAAIKEAGTYKVER	50.2
									EAAAAQLAAIKEAGTYKVER	51.1							GRPYLFSNTIAPAAVGGTLK	38.6
									FICGTTDIHK Carbamidomethyl (C3)	33.8							GRPYLFSNTIAPAAVGGTLK Deamidated (N8)	8.1
									GRPYLFSNTIAPAAVGGTLK	39.7							GTPALFGVIDKVDILNTTLGK	50.8
									GTPALFGVIDKVDILNTTLGK	49.1							GTPALFGVIDKVDILNTTLGK Deamidated (N16)	37.0
									KQLQDNTHLFR	40.0							KQLQDNTHLFR	41.6
									QKGRPYLFSNTIAPAAVGGTLK Deamidated (N10)	9.6							KQLQDNTHLFR Deamidated (Q2)	21.6
									QLQDNTHLFR	20.1							QKGRPYLFSNTIAPAAVGGTLK Deamidated (N10)	16.6
									QLQDNTHLFR Deamidated (Q3)	29.9							VMELLQTTSSAR	60.0
									VMELLQTTSSAR Oxidation (M2)	67.9								
64	Tb927.6.2790	L-threonine 3-dehydrogenase, putative	1.00	614	47	11	10	32.2	CTDRTIEVEYVEGPAQK Carbamidomethyl (C1)	54.3	1.00	714	106	12	10	37.7	CTDRTIEVEYVEGPAQK Carbamidomethyl (C1)	42.8
									IANSWPDSLDDSNAR	49.6							IFIPSTIAAFGDK	45.4
									IFIPSTIAAFGDK	42.0							IFIPSTIAAFGDKCGK Carbamidomethyl (C14)	60.6
									IFIPSTIAAFGDKCGK Carbamidomethyl (C14)	59.3							QIPILHGLPSL	38.4
									IMEAPLEKLTR	40.7							QIPILHGLPSL Deamidated (Q1)	35.1
									IMEAPLEKLTR Oxidation (M2)	49.5							SVRLPGIISAATLPGGGATDYAIHMYHSALLQK	23.9
									QIPILHGLPSL	30.9							TIEVEYVEGPAQK	43.3
									TIEVEYVEGPAQK	42.4							TVYNITGFSFSPSELR	53.8
									TVYNITGFSFSPSELR	71.2							VLVTGALGQIGTDLSLALR	42.3
									VLVTGALGQIGTDLSLALR	44.0							VLVTGALGQIGTDLSLALR Deamidated (Q9)	42.9
									VLVTGALGQIGTDLSLALRDK	17.7							VLVTGALGQIGTDLSLALRDK	42.1
																	VYTELLGTWYR	41.2
65	Tb927.4.1300	hypothetical protein, conserved	1.00	512	38	14	12	48.9	EVAIAHGNGER Deamidated (N8)	5.6	1.00	329	42	9	8	30.5	EVAIAHGNGER Deamidated (N8)	3.9
									FVFTAVLPHR	45.2							FVFTAVLPHR	56.7
									LLILYPMTFSR Oxidation (M7)	26.2							LLILYPMTFSR	22.7
									QMLEDWYGLGNILPFYADVLER	18.0							REEIPKQQILK	32.8
									QMLEDWYGLGNILPFYADVLERR Deamidated (Q1)- Oxidation (M2)	60.4							SVGSLVSVPVIHGEVLPLPGELGVIEAAK	33.4
									REEIPKQQILK	42.2							SVGSLVSVPVIHGEVLPLPGELGVIEAAKVDIKDGGC Carbamidomethyl (C37)	33.1
									SKENGRVIGEDVIPYTSLVGWCGK Carbamidomethyl (C22)	33.6							VINLDESELRAFCGNAR Carbamidomethyl (C13)	9.5
									SKENGRVIGEDVIPYTSLVGWCGK Deamidated (N4)- Carbamidomethyl (C22)	27.5							VLCISHQALNALRPFKR Carbamidomethyl (C3)	31.2
									SSEQVLSIICAPENLNIPNEKRFVFTAVLPHR Carbamidomethyl (C10)	24.1							VLCISHQALNALRPFKR Carbamidomethyl (C3)- Deamidated (Q7)	11.4
									SVGSLVSVPVIHGEVLPLPGELGVIEAAK	34.8								
									SVGSLVSVPVIHGEVLPLPGELGVIEAAKVDIKDGGC Carbamidomethyl (C37)	23.4								
									VINLDESELRAFCGNAR Carbamidomethyl (C13)	29.2								
									VLCISHQALNALRPFKR Carbamidomethyl (C3)	36.2								
									VLCISHQALNALRPFKR Carbamidomethyl (C3)- Deamidated (Q7)	19.4								
66	Tb927.10.4310	prohibitin 2, putative	1.00	493	24	11	9	38.0	AVIAQFNASDLLVKRPEVSNR	53.2	1.00	495	20	10	6	25.8	AVIAQFNASDLLVK	39.7
									AVIAQFNASDLLVKRPEVSNR Deamidated (N7)	32.8							AVIAQFNASDLLVKRPEVSNR	31.1
									DLQTVNLAVR	24.1							AVIAQFNASDLLVKRPEVSNR Deamidated (Q5)	54.3
									GEAEAAKLIGMAVQKNPAFITLR Oxidation (M11)	33.7							LIGMAVQKNPAFITLR	69.3
									IGVMLAER Oxidation (M4)	39.1							LIGMAVQKNPAFITLR Oxidation (M4)	29.0
									LIGMAVQKNPAFITLR	42.2							LIGMAVQKNPAFITLR Oxidation (M4)- Deamidated (N9)	37.4
									LIGMAVQKNPAFITLR Oxidation (M4)	57.9							NRTYGEGANFAIPFLETPVVFDIR	25.5
									NKPTEVLTATGSR	52.0							TYGEGANFAIPFLETPVVFDIR	26.4
									NPAFITLR	28.1							VLYQPHVSALPDIYR	48.5
									TYGEGANFAIPFLETPVVFDIR	28.8							VLYQPHVSALPDIYR Deamidated (Q4)	38.4
									VLYQPHVSALPDIYR	34.1								
67	Tb927.9.14160	rieske iron-sulfur protein, mitochondrial precursor	1.00	464	47	12	9	27.6	DRPVNSWSDEFLKPPVSK	11.1	1.00	665	45	17	10	33.0	DRPVNSWSDEFLKPPVSK	23.7
									GKPVFVYR	29.1							IRQGPAPLNLEVPPYR	30.9
									GKPVFVYRR	25.0							IRQGPAPLNLEVPPYR Deamidated (N9)	19.0
									IRQGPAPLNLEVPPYR	31.5							LPNELEDRAR	27.7
									IRQGPAPLNLEVPPYR Deamidated (N9)	40.0							QGPAPLNLEVPPYR	18.4
									LPNELEDRAR	29.0							QGPAPLNLEVPPYR Deamidated (N7)	16.2
									QGPAPLNLEVPPYR	27.5							QGPAPLNLEVPPYR Deamidated (Q1)	18.2
									QGPAPLNLEVPPYR Deamidated (N7)	18.3							QLEGSNPLTVK	39.8
									QGPAPLNLEVPPYR Deamidated (Q1)	21.9							QLEGSNPLTVKDRPVNSWSDEFLKPPVSK	21.3
									VSLVFKQLEGSNPLTVK	62.6							QLEGSNPLTVKDRPVNSWSDEFLKPPVSK Deamidated (Q1)	20.4
									VSLVFKQLEGSNPLTVKDRPVNSWSDEFLKPPVSK	25.1							VSLVFKQLEGSNPLTVK	58.2
									WVDDKTIYLGKL	51.3							VSLVFKQLEGSNPLTVK Deamidated (N12)	65.2
																	VSLVFKQLEGSNPLTVKDRPVNSWSDEFLKPPVSK	57.1
																	VSLVFKQLEGSNPLTVKDRPVNSWSDEFLKPPVSK Deamidated (Q7)	35.5
																	WVDDKTIYLGKL	33.9
																	YSDPTLCSVDTSSEVVLNTYPEGSR Carbamidomethyl (C7)	31.4
																	YSDPTLCSVDTSSEVVLNTYPEGSR Carbamidomethyl (C7)- Deamidated (N18)	18.3
68	Tb927.1.120	retrotransposon hot spot protein 4 (RHS4), putative	1.00	406	23	13	12	15.1	AAQTAASAVEIK	20.4	1.00	332	16	8	8	10.1	AAQTAASAVEIK	35.4
									AAQTAASAVEIKGVYDSIYNAK	18.3							AAQTAASAVEIKGVYDSIYNAK	60.0
									AREEVVEGR	12.6							FVFSAPEYHER	35.6
									FVFSAPEYHER	40.5							HIVIGTPGIGK	25.0
									HIVIGTPGIGK	14.2							SILNDMASQQLR Oxidation (M6)	18.2
									IDMVGPLPR Oxidation (M3)	54.0							VFDGKPQR	32.9
									KLCVLSEEAPR Carbamidomethyl (C3)	38.7							VSSGTVNVDSYR	50.4
									NAPGLMGLAGR Oxidation (M6)	32.6							WVEKAEITPR	46.9
									SAGWQNDVVTHKLVK	12.9								
									SILNDMASQQLR	52.0								
									SILNDMASQQLR Oxidation (M6)	42.6								
									VFDGKPQR	21.1								
									WVEKAEITPR	22.8								
69	Tb927.10.2350	pyruvate dehydrogenase complex E3 binding protein, putative	1.00	663	45	14	11	41.5	AALEAITPTGR	40.0	1.00	429	23	10	8	33.5	ADFVGQPGFDYEK	23.7
									ADFVGQPGFDYEK	38.5							ADFVGQPGFDYEKAAPAPK	34.9
									ADFVGQPGFDYEKAAPAPK	46.0							ECCAENTNGAVGSSAVVK Carbamidomethyl (C2)- Carbamidomethyl (C3)	65.6
									ECCAENTNGAVGSSAVVK Carbamidomethyl (C2)- Carbamidomethyl (C3)	12.0							ECCAENTNGAVGSSAVVK Carbamidomethyl (C2)- Carbamidomethyl (C3)- Deamidated (N8)	47.6
									ECCAENTNGAVGSSAVVK Carbamidomethyl (C2)- Carbamidomethyl (C3)- Deamidated (N8)	35.5							FTKADFVGQPGFDYEKAAPAPK Deamidated (Q9)	5.6
									FTKADFVGQPGFDYEKAAPAPK	50.0							GSSSSFTNASRECCAENTNGAVGSSAVVK Deamidated (N8)- Deamidated (N19)- Carbamidomethyl (C13)- Carbamidomethyl (C14)	40.1
									GSSSSFTNASR	23.7							IAARLDKAALEAITPTGR	29.7
									IAARLDKAALEAITPTGR	43.3							LDKAALEAITPTGR	52.2
									LDKAALEAITPTGR	51.3							VSDTTLLQQLLNSMPTPK	35.2
									LDKAALEAITPTGRGGR	32.3							VSDTTLLQQLLNSMPTPK Oxidation (M14)	18.2
									VSDTTLLQQLLNSMPTPK	42.5								
									VSDTTLLQQLLNSMPTPK Deamidated (N12)- Oxidation (M14)	38.9								
									VSDTTLLQQLLNSMPTPK Oxidation (M14)	19.0								
									YGGSLDEAVAASGPGVMR Oxidation (M17)	50.0								
70	Tb927.10.180	ATP synthase F1 subunit gamma protein, putative	1.00	260	24	8	8	31.8	CIDSVVSSK Carbamidomethyl (C1)	36.5	1.00	155	6	3	2	19.3	LVAVEGQLTNISSLQQR	45.5
									LVAVEGQLTNISSLQQR	24.5							LVAVEGQLTNISSLQQR Deamidated (Q7)	46.5
									NALVYIPITTNR	43.6							YLFANALQNEEEQLIRDFFDFHAALAVLNAVGENELSEQAAR Deamidated (N5)	45.2
									NALVYIPITTNRGSCGALNSNIVR Carbamidomethyl (C15)	16.5								
									NKFWEGAVTK	40.7								
									QFGITAALIEILSAMSSLEGNAMKGVRR Oxidation (M23)	9.4								
									RNKFWEGAVTK	21.4								
									TSSLYNKTR	38.2								
71	Tb927.3.3900	carnitine O-palmitoyltransferase II, putative	1.00	508	21	11	8	17.9	IPVKDLAVNVLKLPR	46.1	1.00	505	25	11	10	24.5	IPVKDLAVNVLKLPR	43.5
									IPVKDLAVNVLKLPR Deamidated (N9)	35.1							IPVKDLAVNVLKLPR Deamidated (N9)	34.2

									KLKNAGDTQAAVGAAVVHSIAR	32.2							LAPTYEAASTGR	17.5
									LKNAGDTQAAVGAAVVHSIAR	85.6							LAVSEAASNVYGLLK	60.7
									NAGDTQAAVGAAVVHSIAR	70.5							LPIPTLEATAER	46.9
									NAGDTQAAVGAAVVHSIAR Deamidated (Q6)	33.0							NAGDTQAAVGAAVVHSIAR	36.2
									NKLAPTYEAASTGR	30.8							NKLAPTYEAASTGR	31.4
									SPSTDAGDMKLAVSEAASNVYGLLK Oxidation (M9)	29.9							VFDDKQVPLDR	29.1
									VFDDKQVPLDR	18.5							VGTLVQPLTLTFGK	21.9
									WVYNAVNKGVEVFDETRDVSPLLR	58.2							WISDIISDVEGGDGSSVSTSATSLDPAKVGTLVQPLTLTFGK	34.0
									WVYNAVNKGVEVFDETRDVSPLLR Deamidated (N4)	47.1							WVYNAVNKGVEVFDETRDVSPLLR	40.2
72	Tb927.2.3030	ATP-dependent Clp protease subunit, heat shock protein 78 (HSP78), putative,serine peptidase, putative	1.00	505	26	15	14	26.7	AWVNPQAVPPGEFLNK	33.2	1.00	360	23	9	9	20.9	DGEVFVHQNHDAVRPVDAADLGPQLTE	37.3
									DGEVFVHQNHDAVRPVDAADLGPQLTE	7.3							EIDADKQATRTENFLTHDSVTADDIAQVIAR	27.5
									DGEVFVHQNHDAVRPVDAADLGPQLTE Deamidated (Q24)	6.0							FLFDDESFICR Carbamidomethyl (C10)	34.4
									EIDADKQATRTENFLTHDSVTADDIAQVIAR	39.7							IVSGEVPESIKDKK	25.8
									GELHCVGATTLDEYKR Carbamidomethyl (C5)	32.2							LRLQQESKPEQLDSVGR	19.3
									HIEKDAALAR	13.5							RFQSVLVTEPTVEETISVLR	23.3
									IVSGEVPESIKDKK	27.3							RLPDKAIDLIDEAASR	55.2
									LQQESKPEQLDSVGR	29.3							TENFLTHDSVTADDIAQVIAR	32.2
									LRLQQESKPEQLDSVGR	35.2							VILFIDELHTLVGAGASGDGSMDAANLLKPSLAR Oxidation (M22)	11.6
									LVQSELLNR	55.5								
									RFQSVLVTEPTVEETISVLR	12.2								
									RLPDKAIDLIDEAASR	62.1								
									TAIVEGLAQR	13.4								
									TKNNPVLIGEPGVGKTAIVEGLAQR	87.5								
									VLGQDEAIATITNVVR	11.1								
73	Tb927.3.3270	ATP-dependent phosphofructokinase	1.00	523	23	12	12	24.0	AMLNSVTQEDLKVDR	9.1	1.00	319	23	8	7	21.2	DEPSSENPVSVSPLLCELAAAR Carbamidomethyl (C16)	45.9
									DEPSSENPVSVSPLLCELAAAR Carbamidomethyl (C16)	47.1							DEPSSENPVSVSPLLCELAAAR Deamidated (N7)- Carbamidomethyl (C16)	27.6
									DRLHEELAKL	43.1							DRLHEELAKL	30.8
									ELEAINR	16.1							GALVISQEAKR	26.1
									GALVISQEAK	25.8							IHFNPTETTIGIVTCGGICPGLNDVIR Carbamidomethyl (C15)- Carbamidomethyl (C19)	34.4
									GALVISQEAKR	61.3							LPGADYPNPSKK	23.4
									LPGADYPNPSKK	31.3							TFGFQTAVEK	33.3
									RELEAINR	18.6							TIDNDLSFSHR	56.5
									RGVDISVFGVPK	37.7								
									TFGFQTAVEK	41.6								
									TFGFQTAVEKAVQAIR	76.8								
									TIDNDLSFSHR	56.3								
74	Tb927.10.2240	hypothetical protein, conserved	1.00	464	22	10	8	18.7	EAWASEKNDANNGGAAPDAAAAEENVTPAEQR Deamidated (N11)	32.1	1.00	233	9	5	5	11.9	GNDIVCQLSTESAAEALLEKPPVVNECALR Carbamidomethyl (C6)- Carbamidomethyl (C27)	70.9
									GNDIVCQLSTESAAEALLEKPPVVNECALR Carbamidomethyl (C6)- Carbamidomethyl (C27)	61.2							GQNEQISAAIKR Deamidated (Q2)	27.1
									GNDIVCQLSTESAAEALLEKPPVVNECALR Deamidated (N2)- Carbamidomethyl (C6)- Carbamidomethyl (C27)	31.9							ITGEEPKKVVPFGR	39.4
									GQNEQISAAIKR	65.2							LIDAPKGQNEQISAAIKR	32.8
									GQNEQISAAIKR Deamidated (Q2)	57.8							RDAEREPR	12.5
									ITGEEPKKVVPFGR	39.3								
									KGPTDHVR	9.9								
									LIDAPK	24.2								
									LIDAPKGQNEQISAAIKR	51.3								
									NTFNNRPR	22.7								
75	Tb927.11.16730	dihydrolipoyl dehydrogenase	1.00	498	35	13	11	27.7	AAQLGLK	21.0	1.00	404	27	9	7	22.4	ETLHCDALLVSVGR Carbamidomethyl (C5)	26.6
									AAQLGLKTACVEKR Carbamidomethyl (C10)	12.5							LGSDVTVVEFAPR	74.2
									AGVAYKVGKFPFNANSR	34.3							NKVTYYKGEAGFVTPNTLNVK	34.5
									AGVAYKVGKFPFNANSR Deamidated (N13)	11.9							NTIIATGSEPTALPFLPFDEK	33.5
									AKAVATEDGFVK	46.8							NTIIATGSEPTALPFLPFDEKVVLSSTGALALQQVPK	63.2
									INVSLNER	25.7							RPYTAGLGLEK	34.8
									LGSDVTVVEFAPR	65.0							VGKFPFNANSR	23.1
									NKVTYYKGEAGFVTPNTLNVK	33.1							VGKFPFNANSR Deamidated (N7)	18.5
									NTIIATGSEPTALPFLPFDEK	24.8							VGKFPFNANSR Deamidated (N9)	24.3
									RPYTAGLGLEK	31.6								
									SVNGLTSGVEYLLKK	37.1								
									SVNGLTSGVEYLLKK Deamidated (N3)	19.7								
									VGKFPFNANSR	40.0								
76	Tb927.9.12510	ATP-dependent DEAD/H RNA helicase, putative	1.00	418	66	11	10	19.1	AGNKGVATSFYDRNNR	18.9	1.00	459	69	10	8	15.7	DIDAVESFEDLFVEPALALNIAK	43.3
									CGYKEPTPVQR Carbamidomethyl (C1)	15.5							DIDAVESFEDLFVEPALALNIAK Deamidated (N20)	18.1
									DIDAVESFEDLFVEPALALNIAK	32.4							GISLENYASIPVDIVPR	36.8
									GISLENYASIPVDIVPR	28.4							GLDIPNVAHVIQYDLPK	26.7
									GISLENYASIPVDIVPR Deamidated (N6)	32.5							GLDIPNVAHVIQYDLPKEMDDYTHR	27.4
									GISLENYASIPVDIVPRDIDAVESFEDLFVEPALALNIAK	22.2							GLDIPNVAHVIQYDLPKEMDDYTHR Oxidation (M19)	12.8
									GLDIPNVAHVIQYDLPKEMDDYTHR Oxidation (M19)	18.2							NLAVELYHYLR	49.6
									SAEQLHSALK	10.7							SAEQLHSALK	28.5
									SGITPILVATDVASR	65.9							SGITPILVATDVASR	73.4
									TAAFLVPVVHYILK	42.5							TAAFLVPVVHYILK	40.0
									TAAFLVPVVHYILKNGVSPAKDR Deamidated (N15)	23.8								
77	Tb927.3.5050	60S ribosomal protein L4	1.00	429	40	13	11	40.1	AYEKTKEAMTFLK Oxidation (M9)	20.9	1.00	62	4	3	3	10.7	GHKIENVAEVPLVVEDGVR	47.6
									FAVVSALAASSLPALVMSR Oxidation (M17)	17.0							GHKIENVAEVPLVVEDGVRAYEK	9.1
									GHKIENVAEVPLVVEDGVR	55.3							SGFMLPAPMLTSTDVTR Oxidation (M4)- Oxidation (M9)	1.9
									GHKIENVAEVPLVVEDGVRAYEK	27.8								
									IHGGGTSMSGAGAFGNMCR Oxidation (M8)- Oxidation (M17)- Carbamidomethyl (C18)	25.3								
									LSGMKHSAESWGTGR Oxidation (M4)	28.3								
									NSRQPYAVNR	27.1								
									NSRQPYAVNR Deamidated (N1)	25.6								
									RGPMLVMPDNKGTR Oxidation (M4)- Oxidation (M7)	22.8								
									SGFMLPAPMLTSTDVTR Oxidation (M4)- Oxidation (M9)	33.1								
									TVGAIDDVNRVNDSR	11.7								
									YRQPTNGIR	23.2								
									YRQPTNGIR Deamidated (N6)	27.2								
78	Tb927.4.4380	vacuolar-type proton translocating pyrophosphatase 1, putative	0.96	270	30	6	5	8.6	AADVGADLVGKVEK	80.2	0.99	560	94	11	8	12.6	AADVGADLVGKVEK	58.5
	Tb927.8.7980	vacuolar-type proton translocating pyrophosphatase 1,V-type H( )-translocating pyrophosphatase							GKGSPQHAAAVIGDTVGDPLKDTSGPALNILVK	38.2							GKGSPQHAAAVIGDTVGDPLKDTSGPALNILVK	74.7
									GSPQHAAAVIGDTVGDPLKDTSGPALNILVK	46.2							GKGSPQHAAAVIGDTVGDPLKDTSGPALNILVK Deamidated (N29)	48.1
									LMAIISVVFAPVVQSK	9.5							GKGSPQHAAAVIGDTVGDPLKDTSGPALNILVK Deamidated (Q6)	36.2
									LMAIISVVFAPVVQSK Oxidation (M2)	15.4							GSPQHAAAVIGDTVGDPLKDTSGPALNILVK	59.1
									NVYVISKR	32.7							GSPQHAAAVIGDTVGDPLKDTSGPALNILVK Deamidated (Q4)	32.6
																	LMAIISVVFAPVVQSK	10.6
																	NVYVISK	20.3
																	NVYVISKR	26.8
																	YTLAGLLPGALVSGVQVAISASNTGGAWDNAK	20.9
																	YTLAGLLPGALVSGVQVAISASNTGGAWDNAKK	19.8
79	Tb927.9.5150	ribosomal protein S6, putative,NHP2/RS6-like protein	1.00	556	26	12	7	80.2	AAQVSRNAVALAILQMENSPISAK Oxidation (M16)	6.9	0.00	0	0	0	0	0.0		
									AFPLAGDRLTQTILDIVQEASNAK	49.0								
									AFPLAGDRLTQTILDIVQEASNAKMIK Deamidated (N22)- Oxidation (M25)	14.4								
									AFPLAGDRLTQTILDIVQEASNAKMIK Oxidation (M25)	19.8								
									GIADLIVLAGDTNPIEILLHLPLLCEDKNVPYVFVPSK Carbamidomethyl (C25)	60.7								
									IQAVKLEIER	45.4								
									IQAVKLEIERLL	49.5								
									IQAVKLEIERLL Deamidated (Q2)	37.8								
									NAVALAILQMENSPISAK	59.4								
									NAVALAILQMENSPISAK Deamidated (N1)- Oxidation (M10)	32.5								
									NAVALAILQMENSPISAK Deamidated (Q9)- Deamidated (N1)- Oxidation (M10)	31.2								
									NAVALAILQMENSPISAK Oxidation (M10)	70.6								
80	Tb927.4.3950	cytoskeleton-associated protein CAP5.5, putative,cysteine peptidase, Clan CA, family C2, putative	1.00	357	22	10	9	18.1	AATAVANGLREGYSYALLSVHNFPEENITLLK	23.4	1.00	322	16	7	5	11.7	AATAVANGLREGYSYALLSVHNFPEENITLLK	11.9

									AEGNKQFVYQCSSSDPETPAEGFNFVVGR Carbamidomethyl (C11)	14.0							AATAVANGLREGYSYALLSVHNFPEENITLLK Deamidated (N7)	39.0
									CIDDSGELWPSLLQK Carbamidomethyl (C1)	13.5							AEGNKQFVYQCSSSDPETPAEGFNFVVGR Carbamidomethyl (C11)	60.3
									CTFEASETPYFVIPR Carbamidomethyl (C1)	36.7							AEGNKQFVYQCSSSDPETPAEGFNFVVGR Deamidated (Q6)- Carbamidomethyl (C11)	41.9
									ITGYVEAHNPVILSVPTGR	37.0							ITGYVEAHNPVILSVPTGR	52.2
									KAATAVANGLREGYSYALLSVHNFPEENITLLK	20.8							KAATAVANGLREGYSYALLSVHNFPEENITLLK Deamidated (N8)	34.8
									KAATAVANGLREGYSYALLSVHNFPEENITLLK Deamidated (N8)	23.7							NILVDDFLPTVGGVPCYAR Carbamidomethyl (C16)	40.8
									LDGDGRFIQPVEVR	24.3								
									NILVDDFLPTVGGVPCYAR Carbamidomethyl (C16)	42.0								
									VLLNKDGWWK	28.7								
81	Tb927.3.3130	hypothetical protein, conserved	1.00	438	27	13	10	9.0	ANAAVSTSNDVEDVRALPATLLK Deamidated (N2)	47.7	1.00	209	7	6	3	4.0	EAGEEGDRLPINLLLGFKELENSVVDAAQSIPASGGPR	27.4
									ANAAVSTSNDVEDVRALPATLLKGTPPAR	20.2							EAGEEGDRLPINLLLGFKELENSVVDAAQSIPASGGPR Deamidated (N12)	40.7
									ANAAVSTSNDVEDVRALPATLLKGTPPAR Deamidated (N2)	10.1							EAGEEGDRLPINLLLGFKELENSVVDAAQSIPASGGPR Deamidated (N22)	43.3
									ANAAVSTSNDVEDVRALPATLLKGTPPAR Deamidated (N9)	11.2							KAELTLLPR	39.8
									EAGEEGDRLPINLLLGFKELENSVVDAAQSIPASGGPR	11.3							RLAALCQEKEQLPAALQAK Carbamidomethyl (C6)	31.9
									EAGEEGDRLPINLLLGFKELENSVVDAAQSIPASGGPR Deamidated (N12)	24.8							RLAALCQEKEQLPAALQAK Carbamidomethyl (C6)- Deamidated (Q11)	19.6
									HGQVQDADVVAR	47.2								
									KAELTLLPR	39.1								
									LAALCQEKEQLPAALQAK Carbamidomethyl (C5)	38.0								
									NARPEWQTGVR	25.6								
									RLAALCQEKEQLPAALQAK Carbamidomethyl (C6)	38.0								
									RLPAEYGEPLLNKCEAVIQR Carbamidomethyl (C14)	49.4								
									SVADIADRLR	26.9								
82	Tb927.9.6100	TFIIF-stimulated CTD phosphatase, putative	1.00	354	26	9	8	19.8	ILGNVLKER	51.9	1.00	157	13	4	4	16.9	IVTDEVKYGQVGQR	51.8
									IVTDEVKYGQVGQR	62.6							LQADHALMDLIPMLR	24.1
									IVTDEVKYGQVGQRQYEGEMAK Oxidation (M20)	15.1							NGVKIEPFLPNER Deamidated (N1)	33.7
									LQADHALMDLIPMLR Oxidation (M8)- Oxidation (M13)	33.3							SAASSTDLTHELDHWR	12.9
									NGVKIEPFLPNER	35.8								
									QKILGNVLK	18.6								
									QKILGNVLKER	26.1								
									SFPLHPRNGVKIEPFLPNER	29.5								
									SFPLHPRNGVKIEPFLPNER Deamidated (N8)	35.2								
83	Tb927.8.6170	transketolase, putative	1.00	387	29	11	10	25.0	AHGAPLGDDEVAR	33.2	1.00	433	44	14	13	32.3	AHGAPLGDDEVAR	18.0
									ATPNLLVFRPSDQTETSAAWALALENTRGPSILCLSR Carbamidomethyl (C34)	26.0							ATPNLLVFRPSDQTETSAAWALALENTR	24.5
									CLAADGVQQAK Carbamidomethyl (C1)	44.4							ATPNLLVFRPSDQTETSAAWALALENTRGPSILCLSR Carbamidomethyl (C34)	17.7
									FHVQPEVYGIFGK	20.6							FHVQPEVYGIFGK	28.6
									KYTEEFPQEADALQNQLDFKLPPDWK	28.1							KASENALGALLTLTPALVGGSADLSPSNLTRPGSAQMVDFQK	20.2
									KYVGGTAPAKR	38.2							KYEALGFHVIPVSNGDSDFTALR	28.0
									LAGSEKAHGAPLGDDEVAR	34.1							KYTEEFPQEADALQNQLDFKLPPDWK	28.6
									QNTVPQPTSKLEGVAK	16.1							KYVGGTAPAKR	19.4
									SKLPLNDKSIATR	17.6							LAGSEKAHGAPLGDDEVAR	18.5
									SKLPLNDKSIATR Deamidated (N6)	27.6							SKLPLNDKSIATR	26.7
									VFPEGVPVISIEPFVSTGWER	18.9							SKLPLNDKSIATR Deamidated (N6)	21.5
																	VFPEGVPVISIEPFVSTGWER	38.5
																	VKEQFGLDPTK	23.5
																	YTEEFPQEADALQNQLDFKLPPDWK	28.8
84	Tb927.3.930	dynein heavy chain, putative	1.00	305	27	10	9	2.7	IYEPAPSMEVLQDR Oxidation (M8)	36.6	1.00	136	6	6	6	2.0	EALDESSLAINSMLSSRYCAFMR Deamidated (N11)- Oxidation (M13)- Carbamidomethyl (C19)	32.9
									LANPPALIKR	13.9							LANPPALIKR	19.7
									LLALFLPTEGEGGEPSLTER	31.3							LRLVEEQLEAVEAPR	5.5
									LRLVEEQLEAVEAPR	13.8							NMTPVAGAISWAR Oxidation (M2)	7.0
									NMTPVAGAISWAR	9.2							STSKPWEVVTDAVTLIR	64.2
									NMTPVAGAISWAR Oxidation (M2)	26.9							VVDEVVDIASSSVR	6.6
									QLASDSSTLDVLR	35.9								
									QLQEKEGLLQSK	7.5								
									SFKQLPDLITR	31.5								
									STSKPWEVVTDAVTLIR	21.5								
85	Tb927.10.14750	fibrillarin, putative	1.00	468	32	12	11	46.4	ANCIDSTADSATVFASEVQK Carbamidomethyl (C3)	70.6	0.74	107	3	3	3	13.5	ILALNAQHFLK Deamidated (N5)	36.9
									EQASLEPFERDHAVVTGYYKSPPQ	36.5							SLVPGVSIYGEKR	50.0
									GNIFQPHAR	19.6							SNIVPILEDARYPQKYR	19.9
									ILALNAQHFLK	45.0								
									ILALNAQHFLKQNGGFVISIK Deamidated (Q12)	26.0								
									LVDCIFMDVAQPDQAR Carbamidomethyl (C4)- Oxidation (M7)	41.2								
									LVDCIFMDVAQPDQAR Carbamidomethyl (C4)- Oxidation (M7)- Deamidated (Q11)	33.9								
									MRGCYLLAGKDTLATK Oxidation (M1)- Carbamidomethyl (C4)	21.8								
									SLVPGVSIYGEKR	45.2								
									SNIVPILEDARYPQK	19.5								
									SNIVPILEDARYPQKYR	17.4								
									YRMLIPRLVDCIFMDVAQPDQAR Oxidation (M3)- Oxidation (M14)- Carbamidomethyl (C11)	19.7								
86	Tb927.9.6090	TFIIF-stimulated CTD phosphatase, putative	1.00	315	22	9	8	19.2	ILGNVLKER	51.9	0.87	112	10	4	4	16.0	LQADHALMDLIPMLR	24.1
									LQADHALMDLIPMLR Oxidation (M8)- Oxidation (M13)	33.3							NGVKIEPFLPNER Deamidated (N1)	33.7
									NGVKIEPFLPNER	35.8							SAASSTDLTHELDHWR	12.9
									QKILGNVLK	18.6							VIERGEEEVAR	30.4
									QKILGNVLKER	26.1								
									SFPLHPRNGVKIEPFLPNER	29.5								
									SFPLHPRNGVKIEPFLPNER Deamidated (N8)	35.2								
									VIERGEEEVAR	28.9								
									VIVIDSDVR	20.3								
87	Tb927.11.2650	heat shock protein 84, putative	1.00	388	24	8	6	14.5	ALSDFISKR	40.7	1.00	321	14	8	7	18.5	ALSDFISKR	34.9
									ELVSNASDALEKR	62.1							FIKGAVDTESIPLNVSR	22.4
									FIKGAVDTESIPLNVSR	72.9							FVIRDTGIGMTREELAENLGTIAGSGSK	38.4
									FVIRDTGIGMTREELAENLGTIAGSGSK	25.1							FVIRDTGIGMTREELAENLGTIAGSGSK Deamidated (N18)	47.8
									FVIRDTGIGMTREELAENLGTIAGSGSK Oxidation (M10)	14.6							KIYRITGQMAGAPPK Oxidation (M9)	7.4
									FVIRDTGIGMTREELAENLGTIAGSGSK Oxidation (M10)- Deamidated (N18)	20.8							KLYTLSISPNSEEVETAGLLVEQMFDNAVIAAGLLEDPR	45.5
									HLMELSKPEEYPREEGDEAPIISITCNQSK Oxidation (M3)- Carbamidomethyl (C26)	34.1							LHYAVDAPLSIR	44.8
									LHYAVDAPLSIR	33.1							QLLDIVACSLYTEKEVFIR Carbamidomethyl (C8)	42.6
88	Tb927.11.13180	hypothetical protein, conserved	1.00	286	19	5	4	15.9	IGGGSVAYAPQGLDGAEGGDHPPTVTMR Oxidation (M27)	45.7	1.00	366	20	8	7	23.6	ADNVNLDEFKDR	33.6
									LLSEAEDDDAFTAPILSGAHEK	43.1							EFPQLGLSLTVPSPWK	49.1
									SPVCNDLSVENQLLAEALREIGAESPR Carbamidomethyl (C4)	38.0							IGGGSVAYAPQGLDGAEGGDHPPTVTMR Oxidation (M27)	10.2
									SPVCNDLSVENQLLAEALREIGAESPR Carbamidomethyl (C4)- Deamidated (Q12)	37.5							LLSEAEDDDAFTAPILSGAHEK	38.0
									SVSPSTAEPKPK	29.3							MRQEAEEGGIRDIAR	4.8
																	SPVCNDLSVENQLLAEALREIGAESPR Carbamidomethyl (C4)	61.8
																	SPVCNDLSVENQLLAEALREIGAESPR Carbamidomethyl (C4)- Deamidated (N5)	40.3
																	SVSPSTAEPKPK	35.1
89	Tb927.9.10770	Polyadenylate-binding protein 2 (Poly(A)-binding protein 2) (Poly(A)-binding protein II) (PABII) (Polyadenylate-binding nuclear protein 1) (Nuclear poly(A)-binding protein 1) (PABP2), putative	1.00	353	38	9	8	22.0	DLAGRPTVGFGYVAYATHEAAAK	35.6	1.00	303	24	7	6	19.5	AVEELDDKESPLAK	12.7
									ISYEKEESAVDAVR	21.3							DLAGRPTVGFGYVAYATHEAAAK	30.3
									LTAIGLATDEKGESR	68.3							ISYEKEESAVDAVR	26.6
									LTAIGLATDEKGESRGYAR	33.9							LTAIGLATDEKGESR	69.8
									NFDDTVTSERLK	39.1							NLDPSVDDEKLKEVFSPFGEVTSAK	37.8
									NLDPSVDDEKLKEVFSPFGEVTSAK	39.1							NLDPSVDDEKLKEVFSPFGEVTSAK Deamidated (N1)	26.8
									NLDPSVDDEKLKEVFSPFGEVTSAK Deamidated (N1)	28.8							SLGYGYVNFQNPADAEK	32.2
									SLGYGYVNFQNPADAEKALDQAGVK	6.2								
									SLGYGYVNFQNPADAEKALDQAGVKLGTK	15.4								
90	Tb927.8.6580	succinate dehydrogenase flavoprotein, putative	1.00	444	28	8	6	15.9	AAMGVAASGYNVACVSK Oxidation (M3)- Carbamidomethyl (C14)	57.5	1.00	365	12	7	7	16.3	AAMGVAASGYNVACVSK Oxidation (M3)- Carbamidomethyl (C14)	11.8
									AAMGVAASGYNVACVSK Oxidation (M3)- Deamidated (N11)- Carbamidomethyl (C14)	31.8							AFGGQSIHYGGK	51.5
									AFGGQSIHYGGK	56.8							AITLELLAGR	30.7
									AKDLASRDVVSR	32.3							AYPVIDHTFDCVVVGAGGSGLR Carbamidomethyl (C11)	62.5
									AYPVIDHTFDCVVVGAGGSGLR Carbamidomethyl (C11)	55.7							ILHNKGDIPVAR	42.6

									SDAGESSIADLDKILHNKGDIPVAR	34.8							LGANSLLDIVVFGK	61.8
									TKDGFIYQR	51.9							SCANTVIFNLTK Carbamidomethyl (C2)	67.8
									TKDGFIYQR Deamidated (Q8)	31.9								
91	Tb927.11.7380	glycerol-3-phosphate dehydrogenase (FAD-dependent), mitochondrial	1.00	338	27	7	7	11.1	ARKSEEVAHALAALESFHGQ	35.5	1.00	278	14	6	6	16.6	ARKSEEVAHALAALESFHGQ	43.6
									IIMSHQAPHLCR Oxidation (M3)- Carbamidomethyl (C11)	27.4							EDPSLLKPLHPEEPVLAADVVYSAQR	35.8
									KSEEVAHALAALESFHGQ	55.0							GRVECKPCITEELQVIGAHELETVPEEPK Carbamidomethyl (C5)- Carbamidomethyl (C8)	45.9
									LITDKTSVR	36.6							KSEEVAHALAALESFHGQ	65.6
									MAPSSGTHIIIDR Oxidation (M1)	38.1							MAPSSGTHIIIDR Oxidation (M1)	29.1
									MAPSSGTHIIIDRK Oxidation (M1)	42.1							SVVNAGGPFTAR	24.9
									SVVNAGGPFTAR	33.6								
92	Tb927.9.2470	nucleolar protein	1.00	345	24	12	11	18.5	AAIEQVPAR	37.7	1.00	277	18	6	6	15.3	ALQQQYQQLVEDEDR	25.3
									IGGDYEWSNTLAR	53.5							ENFTNLTFEGYPLEASFTTEHDKSTPKEELQALLER Deamidated (Q31)- Deamidated (N2)- Deamidated (N5)	39.7
									IKNELALVPAESLKESK	12.1							IGGDYEWSNTLAR	6.7
									KGASSQALQGAPLPVVIRR	45.5							SAGRPSEELFSLVR	28.4
									QAIAAAFTR	27.5							TLLGKEIPVYVQLQPEEVLK	45.6
									SAGRPSEELFSLVR	38.0							YGYALLGYNEAADDFFDR	50.1
									TLLGKEIPVYVQLQPEEVLK	18.1								
									TLLGKEIPVYVQLQPEEVLKMQQTVR Oxidation (M21)	24.3								
									VIGLPTSQHVR	17.6								
									VIGLPTSQHVR Deamidated (Q8)	8.3								
									YGYALLGYNEAADDFFDR	3.9								
									YGYALLGYNEAADDFFDRGGRR	9.1								
93	Tb927.8.1160	vacuolar-type Ca2 -ATPase, putative	0.65	104	7	3	3	5.4	DTSNYGTLAGR	23.8	0.99	572	40	9	9	14.5	GAEHPILLSGTVVSTAEDAYILACAVGESSFGGK Carbamidomethyl (C24)	33.8
									IGTDGAVPEEEPEGPFVWLALLGIQDPLRPEVVDAVR	43.7							GELHTLFSCVGDAKPLYEELGGVEGIAER Carbamidomethyl (C9)	37.8
									LIPVSEPSFEK	14.9							GFAIFPFTSER	47.6
																	IGTDGAVPEEEPEGPFVWLALLGIQDPLRPEVVDAVR	72.9
																	KGAEHPILLSGTVVSTAEDAYILACAVGESSFGGK Carbamidomethyl (C25)	47.0
																	LGTSITDGIDSFSVENR	55.1
																	SGDIVLLDDNFR	61.6
																	SRGFAIFPFTSER	31.5
																	TDKGNKTDQAILDFVDR	49.3
94	Tb927.7.2700	NADH-cytochrome b5 reductase, putative	1.00	366	29	9	9	38.0	AILLVSGPPGFMK Oxidation (M12)	55.7	1.00	176	5	4	3	12.2	NFLGRPSNTTK	42.0
									KVIEETMPSPNCVDKAILLVSGPPGFMK Oxidation (M7)- Oxidation (M27)- Carbamidomethyl (C12)	18.2							SPFSQSVYHSYK	29.7
									NFLGRPSNTTK	34.6							SPFSQSVYHSYK Deamidated (Q5)	47.1
									SGQYEHIGMLAGGTGVTPMYQIAR Oxidation (M9)- Oxidation (M19)	43.2							VGDSIEVQGPWK Deamidated (Q8)	49.8
									SPFSQSVYHSYK	27.5								
									SQGPLSGYLK	25.7								
									SQGPLSGYLKELGYPEAMVYKF Oxidation (M18)	24.8								
									VGDSIEVQGPWK	45.0								
									VIEETMPSPNCVDKAILLVSGPPGFMK Oxidation (M6)- Oxidation (M26)- Carbamidomethyl (C11)	19.1								
95	Tb927.10.470	choline dehydrogenase, putative	1.00	335	22	8	7	25.1	IAVIEEGEDIRRQPR	49.5	1.00	158	16	4	4	18.5	IAVIEEGEDIRRQPR	39.6
									NLACSLLSHR Carbamidomethyl (C4)	43.8							NVTHGVQIIAQLLKPK	35.5
									NLACSLLSHR Deamidated (N1)- Carbamidomethyl (C4)	32.2							NVYACDESILPSPLLGGSLPFILALGEK Carbamidomethyl (C5)	20.2
									NVYACDESILPSPLLGGSLPFILALGEK Carbamidomethyl (C5)	12.6							VLPFSNLFSELIAEHFESAGVYGGATYPAVAPETFLVK	37.5
									SWLIGDEADWTATPWDFRGDLLPR	46.5								
									VLPFSNLFSELIAEHFESAGVYGGATYPAVAPETFLVK	25.8								
									VLPFSNLFSELIAEHFESAGVYGGATYPAVAPETFLVKGTR	24.0								
									VQRLENVEGAGPHR	32.1								
96	Tb927.8.1180	vacuolar-type Ca2 -ATPase 1	0.60	89	6	2	2	4.3	DTSNYGTLAGR	23.8	0.96	534	38	8	8	14.3	GAEHPILLSGTVVSTAEDAYILACAVGESSFGGK Carbamidomethyl (C24)	33.8
									IGTDGAVPEEEPEGPFVWLALLGIQDPLRPEVVDAVR	43.7							GELHTLFSCVGDAKPLYEELGGVEGIAER Carbamidomethyl (C9)	37.8
																	GFAIFPFTSER	47.6
																	IGTDGAVPEEEPEGPFVWLALLGIQDPLRPEVVDAVR	72.9
																	KGAEHPILLSGTVVSTAEDAYILACAVGESSFGGK Carbamidomethyl (C25)	47.0
																	LGTSITDGIDSFSVENR	55.1
																	SGDIVLLDDNFR	61.6
																	SRGFAIFPFTSER	31.5
																	TDKGNKTDQAILDFVDR	49.3
97	Tb927.11.13280	mitochondrial RNA binding protein 2	1.00	221	18	6	6	35.7	AQLPPAFDVVHWNDEDISRGHLLR	27.9	1.00	311	13	8	5	29.5	AQLPPAFDVVHWNDEDISR	49.8
									FLTQALHYNTGFAR	57.4							FLTQALHYNTGFAR	49.2
									RAQLPPAFDVVHWNDEDISRGHLLR	27.1							FLTQALHYNTGFAR Deamidated (N9)	6.7
									RPSLAQQR	18.3							FLTQALHYNTGFAR Deamidated (Q4)	37.2
									VLHRDTFVVLDYHR	15.3							RAQLPPAFDVVHWNDEDISRGHLLR	23.0
									YTNATFTPNPAAPYTFTLK	42.2							RPSLAQQR	25.5
																	YTNATFTPNPAAPYTFTLK	45.0
																	YTNATFTPNPAAPYTFTLK Deamidated (N3)	32.9
98	Tb927.5.1210	short-chain dehydrogenase, putative	1.00	387	29	9	6	22.5	AGDWAVVTGGTEGIGR	56.3	1.00	232	11	5	4	16.4	AGDWAVVTGGTEGIGR	64.6
									AVALDLANR	21.7							LLPESLRGEK	18.6
									LLPESLRGEK	24.5							QTLNQLGVVNR	35.6
									NHIIIEAIAR	18.5							QTLNQLGVVNR Deamidated (N10)	29.6
									QTLNQLGVVNR	57.8							TQSKLDEVVAEIEK	64.9
									QTLNQLGVVNR Deamidated (Q1)	22.9								
									QTLNQLGVVNR Deamidated (Q5)	43.7								
									TQSKLDEVVAEIEK	34.7								
									TQSKLDEVVAEIEK Deamidated (Q2)	24.1								
99	Tb927.6.2170	co-chaperone GrpE, putative	1.00	343	31	9	9	58.1	AYGITSFGKDMLDVVDTLER Oxidation (M11)	13.8	1.00	149	10	4	4	41.4	AADAENARRIGSEDVTK Deamidated (N6)	7.1
									FDPNFHDALLKVPPTAEAPPGHISTVLK	36.0							AYGITSFGKDMLDVVDTLER	26.3
									GLEAITKLPQAEVEGHKTLSSIHTGIK	16.0							FDPNFHDALLKVPPTAEAPPGHISTVLK	45.3
									IAELKKEVLYR	42.1							GLEAITKLPQAEVEGHKTLSSIHTGIK Deamidated (Q10)	43.8
									IGSEDVTKAK	39.1								
									LSLKLLLNNLAK	36.5								
									TGYKIQDR	28.8								
									VLRASQVGVASDD	40.4								
									VPPTAEAPPGHISTVLK	21.2								
100	Tb927.7.6360	histone H2A variant	1.00	459	43	10	5	40.2	AARADLNFPVGR	50.9	0.00	0	0	0	0	0.0		
									AARADLNFPVGR Deamidated (N7)	47.3								
									ADLNFPVGR	49.1								
									CGASAAIYCAALLEYLTSEVIELAGAAAK Carbamidomethyl (C1)- Carbamidomethyl (C9)	54.5								
									GGVVPFVHK	33.9								
									HLLLAIRGDEELNQIVNATIAR	36.5								
									HLLLAIRGDEELNQIVNATIAR Deamidated (N13)	25.0								
									HLLLAIRGDEELNQIVNATIAR Deamidated (N17)	32.5								
									HLLLAIRGDEELNQIVNATIAR Deamidated (Q14)	9.1								
									HLLLAIRGDEELNQIVNATIAR Deamidated (Q14)- Deamidated (N13)	6.3								
101	Tb927.10.12500	P-type H -ATPase, putative	1.00	230	25	7	5	14.5	AISEHREGDDGSVPLVPPSKGLTSAEAEELLLK	22.4	1.00	200	10	5	4	10.0	AISEHREGDDGSVPLVPPSKGLTSAEAEELLLK	36.7
									GAPHVIIEMVHNQDEINDSVVDIIDKLASR	14.5							FQVFYLPVMMFMLITLLNDGCLMTIGYDRVVPSKLPQR Oxidation (M9)- Oxidation (M10)- Oxidation (M12)- Oxidation (M23)- Carbamidomethyl (C21)	30.5
									GAPHVIIEMVHNQDEINDSVVDIIDKLASR Oxidation (M9)	23.4							GAPHVIIEMVHNQDEINDSVVDIIDKLASR	35.3
									GAPHVIIEMVHNQDEINDSVVDIIDKLASR Oxidation (M9)- Deamidated (N17)	47.2							GAPHVIIEMVHNQDEINDSVVDIIDKLASR Deamidated (N12)	34.0
									GYTCAMTGDGVNDAPALKR Carbamidomethyl (C4)- Oxidation (M6)- Deamidated (N12)	16.4							GYTCAMTGDGVNDAPALKR Carbamidomethyl (C4)- Oxidation (M6)- Deamidated (N12)	14.5
									LGSGSAVPADCTINEGVIDVDEAALTGESLPVTMGTEHMPK Carbamidomethyl (C11)- Oxidation (M34)- Oxidation (M39)	35.3							TAATLVDKR	34.5
									TAATLVDKR	37.2								
102	Tb927.5.3810	orotidine-5-phosphate decarboxylase/orotate phosphoribosyltransferase, putative,OMPDCase-OPRTase, putative	1.00	414	29	8	8	25.1	ACAPTLWILVPGVGAQGGDLK Carbamidomethyl (C2)	20.8	1.00	347	21	7	7	20.7	ACAPTLWILVPGVGAQGGDLK Carbamidomethyl (C2)	36.8
									ADTAAAAVLECKR Carbamidomethyl (C11)	42.6							ACAPTLWILVPGVGAQGGDLKAALQAGLR Carbamidomethyl (C2)	24.4
									AVAQKLCEDINR Carbamidomethyl (C7)	45.8							ADTAAAAVLECKR Carbamidomethyl (C11)	22.5
									AVAQKLCEDINRIR Carbamidomethyl (C7)	28.6							AVAQKLCEDINR Carbamidomethyl (C7)	30.8

									AVTQAKDPR	26.4							NVGLVVGATDPVALSR	71.7
									NVGLVVGATDPVALSR	64.0							RGDIADTAEAYAK	43.4
									RGDIADTAEAYAK	52.2							TDGSGLLINVSR	58.8
									TSNKGSSELQCLQTNGQSLYETIAERAER Carbamidomethyl (C11)	52.9								
103	Tb927.10.13510	zinc metallopeptidase, putative	1.00	329	20	7	7	10.9	ELFEEARGAAPAIIFIDEIDAIGSR	22.2	1.00	231	18	6	6	23.0	ELADMTPGLSPATIATVVNEAALQSGIQEK Oxidation (M5)	4.7
									GAAPAIIFIDEIDAIGSR	50.6							GAAPAIIFIDEIDAIGSR	74.2
									GCLLTGEPGTGKTLLAK Carbamidomethyl (C2)	59.8							GCLLTGEPGTGKTLLAK Carbamidomethyl (C2)	1.3
									GLNDAYAHR	34.5							GLNDAYAHR	30.9
									GQALGYTQR	41.0							KLVEKESFLEAVDNTLVGSK	21.8
									VRELFEEAR	35.0							TINQLLAELDGLNSGSDVLIVMAATNFQDNIDKALLR	52.9
									VRELFEEARGAAPAIIFIDEIDAIGSR	26.2								
104	Tb927.5.520	stomatin-like protein, putative	1.00	499	25	10	7	19.6	NTILNIVPQGR	62.0	1.00	219	11	5	4	12.1	RSNTVVLGGGGSGLPVSDPAAFTAQALSVFR	66.4
									NTILNIVPQGR Deamidated (N5)	55.5							RSNTVVLGGGGSGLPVSDPAAFTAQALSVFR Deamidated (Q25)- Deamidated (N3)	30.6
									RSNTVVLGGGGSGLPVSDPAAFTAQALSVFR	63.6							SNTVVLGGGGSGLPVSDPAAFTAQALSVFR	30.1
									SNTVVLGGGGSGLPVSDPAAFTAQALSVFR	46.6							SVGTVANAFNASPNPQSFR	41.8
									SNTVVLGGGGSGLPVSDPAAFTAQALSVFR Deamidated (Q24)	18.4							VAEEYIEKFGELAR	9.0
									SVGTVANAFNASPNPQSFR	35.1								
									SVGTVANAFNASPNPQSFRDAVALR	13.9								
									SVGTVANAFNASPNPQSFRDAVALR Deamidated (Q16)- Deamidated (N10)	11.7								
									VAEEYIEKFGELAR	41.4								
									YATVLRAEAEAAAMALKADAVGR Oxidation (M14)	27.5								
105	Tb927.11.3250	dynein heavy chain, putative	1.00	412	29	11	9	3.7	AINESVIHSLTCVRDQLDPEWISENEGTPLVDVR Carbamidomethyl (C12)	35.2	1.00	94	5	3	3	1.1	SIVNSFIANVGAGAGLIKPLVDGR	22.3
									ALSNFSDEPWER	40.0							TVQADVLFR	4.1
									LVSGIDKIKEAGVQVAALQEVLHR	14.7							VFLSAEPSNVIPIGILQR	33.3
									LVSGIDKIKEAGVQVAALQEVLHR Deamidated (Q14)	13.8								
									SEGSPLVSRDSEDWQKYVSYVDGVVTK	16.8								
									SIKLTSEPPTGIR	37.0								
									SIVNSFIANVGAGAGLIKPLVDGR	53.7								
									TAALMETVGREK	12.4								
									TVQADVLFR	25.2								
									VFLSAEPSNVIPIGILQR	53.5								
									VFLSAEPSNVIPIGILQR Deamidated (N9)	37.7								
106	Tb927.10.11310	intraflagellar transport protein IFT55/IFT57, putative	0.95	39	1	1	1	5.3	QASGEAVLTVLSLLADHAILSK	39.2	1.00	210	8	6	5	27.4	EQTLAQQFSDQVEDYRVK	37.4
																	LRELNSSQDAANIASQSVQQLSAELNQVSGLLDQVKR Deamidated (Q19)	69.4
																	QASGEAVLTVLSLLADHAILSK	45.4
																	QASGEAVLTVLSLLADHAILSK Deamidated (Q1)	10.9
																	SGKEDTGIPVESEINADEWNLEVER	25.1
																	SRIENAAILLK Deamidated (N5)	8.7
107	Tb927.8.3060	cytosolic leucyl aminopeptidase, putative,metallo-peptidase, Clan MF, Family M17	1.00	401	38	9	7	18.2	AIAELQLPVNVVAAVGLAENAIGPSSYHPSCILTSR Carbamidomethyl (C31)	36.7	1.00	425	33	7	6	19.2	AIAELQLPVNVVAAVGLAENAIGPSSYHPSCILTSR Carbamidomethyl (C31)	24.8
									AIKGNIADLVNVPSVR	54.4							EGGSCTAAAFLSNFVEKDVK Carbamidomethyl (C5)	34.2
									AIKGNIADLVNVPSVR Deamidated (N11)	33.6							GFQPAGAPGFGVELLVDYFR	42.6
									EGGSCTAAAFLSNFVEKDVK Carbamidomethyl (C5)	49.0							SLDVLPLFSFSK	44.8
									GFQPAGAPGFGVELLVDYFR	27.7							SLPNVLYSVGDLFQPASALPAEDVAEKTVTYAVAAAYR	54.4
									GFQPAGAPGFGVELLVDYFR Deamidated (Q3)	34.3							SLPNVLYSVGDLFQPASALPAEDVAEKTVTYAVAAAYRYDR	45.1
									SLDVLPLFSFSK	48.3							SLPNVLYSVGDLFQPASALPAEDVAEKTVTYAVAAAYRYDR Deamidated (Q14)	57.6
									SLDVLPLFSFSKK	32.5								
									SSGEEVWPMPIGDEHQR	5.4								
108	Tb927.8.1330	60S ribosomal protein L7a, putative	1.00	570	38	11	11	41.7	AGVPYKKPEVTQK	26.9	0.93	237	7	5	5	30.4	AGVPYKKPEVTQK	26.6
	Tb927.8.1340	60S ribosomal protein L7a, putative							AGVPYKKPEVTQKK	32.7							ASAAAPSPFVARPK	46.0
									AKASAAAPSPFVARPK	42.7							DFGIGRDVPYAR	38.3
									APLCVVSGLQEVTR Carbamidomethyl (C4)	37.7							LVLIANNVDPIELVLWMPTLCR Carbamidomethyl (C21)	19.9
									ASAAAPSPFVARPK	55.5							TATCVAFTDVNAEDQAALKNLTR Carbamidomethyl (C4)	79.5
									DFGIGRDVPYAR	39.8								
									KTATCVAFTDVNAEDQAALKNLTR Carbamidomethyl (C5)	56.9								
									LKVPPALNQFTK	24.8								
									LVLIANNVDPIELVLWMPTLCR Carbamidomethyl (C21)	12.3								
									RLKVPPALNQFTK	42.6								
									TATCVAFTDVNAEDQAALKNLTR Carbamidomethyl (C4)	67.9								
109	Tb927.10.6910	Sterol methyltransferase, putative	0.97	355	29	8	6	29.3	FTSCNVMGVNNNEYQINR Carbamidomethyl (C4)- Oxidation (M7)	43.9	0.96	342	24	7	4	24.2	GGELGIFTPSFFVK	45.5
	Tb927.10.6950	sterol 24-c-methyltransferase, putative							GGELGIFTPSFFVK	38.5							LAPAGTCKATEILEEGAVNLVKGGELGIFTPSFFVK Carbamidomethyl (C7)	37.3
									LAPAGTCKATEILEEGAVNLVK Carbamidomethyl (C7)	54.3							LAPAGTCKATEILEEGAVNLVKGGELGIFTPSFFVK Carbamidomethyl (C7)- Deamidated (N19)	26.8
									LAPAGTCKATEILEEGAVNLVKGGELGIFTPSFFVK Carbamidomethyl (C7)	26.1							RQDATSLTNEYYDIVTDFYEYGWGQNFHFAPR	49.6
									LAPAGTCKATEILEEGAVNLVKGGELGIFTPSFFVK Carbamidomethyl (C7)- Deamidated (N19)	43.4							RQDATSLTNEYYDIVTDFYEYGWGQNFHFAPR Deamidated (N9)	36.1
									RQDATSLTNEYYDIVTDFYEYGWGQNFHFAPR	19.2							SVPWYEPLQGSYTSLSGLR	26.9
									SVPWYEPLQGSYTSLSGLR	57.5							SVPWYEPLQGSYTSLSGLR Deamidated (Q9)	70.5
									SVPWYEPLQGSYTSLSGLR Deamidated (Q9)	19.4								
110	Tb927.9.9660	hypothetical protein, conserved	1.00	309	46	7	5	20.2	EGPPSPGKVVAYDTLLRPLQR	29.3	1.00	300	32	7	4	15.7	EGPPSPGKVVAYDTLLRPLQR	24.1
									GALGVDVESSLEPSQEEIIAQREDAFR	57.3							GALGVDVESSLEPSQEEIIAQREDAFR	44.3
									GALGVDVESSLEPSQEEIIAQREDAFRR	29.5							GALGVDVESSLEPSQEEIIAQREDAFR Deamidated (Q15)	61.1
									GKQSEEPVAQAEPVKR	39.3							GALGVDVESSLEPSQEEIIAQREDAFR Deamidated (Q21)	51.1
									GKQSEEPVAQAEPVKR Deamidated (Q3)	11.0							GALGVDVESSLEPSQEEIIAQREDAFRR	19.6
									GKQSEEPVAQAEPVKR Deamidated (Q3)- Deamidated (Q10)	8.4							GALGVDVESSLEPSQEEIIAQREDAFRR Deamidated (Q21)	19.2
									LAASAKEGPPSPGKVVAYDTLLRPLQR	44.0							LAASAKEGPPSPGK	7.7
111	Tb927.2.2510	voltage-dependent anion-selective channel	1.00	473	36	10	9	37.4	CGYTAAVRTNKLNTYTTSITAR Carbamidomethyl (C1)	32.9	0.98	343	23	7	5	25.2	DLLTKNYSSAQK	12.0
	Tb927.2.2520	voltage-dependent anion-selective channel							GNCDWTASCR Carbamidomethyl (C3)- Carbamidomethyl (C9)	41.9							GQPWTGVVGVEADCVLVK Carbamidomethyl (C14)	45.1
									GQPWTGVVGVEADCVLVK Carbamidomethyl (C14)	27.5							SLKPGVLITHS	36.2
									NYSSAQKWKLESK	31.7							SLVLYKDYHKDAK	26.5
									SLKPGVLITHS	38.1							TNKLNTYTTSITAR	47.6
									SLVLYKDYHKDAK	31.3							TNKLNTYTTSITAR Deamidated (N2)	41.8
									TNKLNTYTTSITAR	62.1							TNKLNTYTTSITAR Deamidated (N5)	28.4
									TNKLNTYTTSITAR Deamidated (N5)	28.5								
									TNKLNTYTTSITARVPK	37.3								
									VVVGAEVVCGR Carbamidomethyl (C9)	53.3								
112	Tb927.11.6460	hypothetical protein, conserved	1.00	241	14	6	5	10.8	ALAVNNIPVSDEEFDAITDAFAAPYTHGGSGEVTPR	59.1	1.00	305	8	7	6	14.1	ALAVNNIPVSDEEFDAITDAFAAPYTHGGSGEVTPR	70.5
									GAGSLVSYTNFLQALQAEEPPPELLTTLK	18.2							ALAVNNIPVSDEEFDAITDAFAAPYTHGGSGEVTPR Deamidated (N6)	90.0
									HTEADGGSANYLAQTR	5.5							FIADIGQVAPSAVPSVSTR	45.2
									TNFQQGLAR	29.6							GAGSLVSYTNFLQALQAEEPPPELLTTLK	16.4
									VNYVQFLADTNPR	41.2							LSHDVSSHQALLLPFFADFDRFHR	32.9
									VNYVQFLADTNPR Deamidated (N11)	43.0							TGFVTAPQFESALGQLR	32.7
																	TNFQQGLAR	14.2
113	Tb927.7.6670	hypothetical protein, conserved	1.00	443	29	11	11	6.6	AVLHVDAPADGR	20.1	1.00	87	3	3	3	2.3	EGIDVTGLRPEVQQALAR	13.8
									AVTVVPAETTSVGTQR	60.4							HVGGAEVTCEPLFASLEQLLRLPDDTESTLR Carbamidomethyl (C9)	35.2
									EGIDVTGLRPEVQQALAR	14.5							RVPLQQPPTTTALNLPLSGLR	37.6
									FVSQEVVEQQLK	29.0								
									HVGGAEVTCEPLFASLEQLLRLPDDTESTLR Carbamidomethyl (C9)	34.8								
									LLDSIQIIPLRQPHPR	30.7								
									LSLAAVAAAAELPSRK	28.5								
									LSLAAVAAAAELPSRKGAAAEFAR	29.0								
									QVLPEGTLQFIAEDPGEAVQDKLLAAPYTSR	32.8								
									RVPLQQPPTTTALNLPLSGLR	47.1								
									SLPSQGDSLPVVWAATGR	20.5								
114	Tb927.3.4630	UDP-glucose:glycoprotein glucosyltransferase, putative	1.00	430	21	9	7	8.0	AKTIDLCNNPLTK Carbamidomethyl (C7)	15.3	1.00	159	6	3	3	3.6	IAAVVAALRR	29.9
									IAAVVAALRR	33.3							TIDLCNNPLTK Carbamidomethyl (C5)	29.1
									IPKLENVRR	16.7							VVAPWTETPLLQEGCEAVASLTVPVGTQTHDDAPSGFYK Carbamidomethyl (C15)	77.6

									IQFQGESLSGGSGDTTAR	26.8								
									IQFQGESLSGGSGDTTAR Deamidated (Q2)	55.4								
									TGGCAAEGRPFALIGNK Carbamidomethyl (C4)	23.3								
									VVAPWTETPLLQEGCEAVASLTVPVGTQTHDDAPSGFYK Carbamidomethyl (C15)	86.4								
									VVAPWTETPLLQEGCEAVASLTVPVGTQTHDDAPSGFYK Deamidated (Q12)- Carbamidomethyl (C15)	70.6								
									VWLPQDAVTWFNDVENNAYLYRLPTTLR	36.7								
115	Tb927.8.1510	ATP-dependent DEAD/H RNA helicase, putative	0.98	40	4	1	1	6.5	FSDLVAPDTIHQAFIDLGYKSPTPIQSIAWPILLNSR	34.7	1.00	203	10	4	4	9.0	FSDLVAPDTIHQAFIDLGYK	40.8
																	FSDLVAPDTIHQAFIDLGYKSPTPIQSIAWPILLNSR	43.5
																	SPTPIQSIAWPILLNSR	46.6
																	VGEGPIALVLAPTR	51.9
116	Tb927.11.11520	glycosomal membrane protein, putative	1.00	557	45	12	11	28.9	AFSGVFK	28.1	0.91	44	3	1	1	6.0	LLEQTDGRDKILK	38.9
									AFSGVFKALGSLDTCQSR Carbamidomethyl (C15)	33.5								
									ALGSLDTCQSR Carbamidomethyl (C8)	36.3								
									ALGSLDTCQSR Carbamidomethyl (C8)- Deamidated (Q9)	59.5								
									FVKLLEQTDGRDK	23.1								
									ILKAFSGVFK	43.2								
									LLEQTDGRDK	34.5								
									LLEQTDGRDKILK	37.0								
									MAKWVGDVPK Oxidation (M1)	25.7								
									SIGDARCLLR Carbamidomethyl (C7)	29.4								
									SSAFGAVGK	24.7								
									SSAFGAVGKSIGDAR	67.9								
117	Tb927.3.2600	ATP-dependent DEAD/H RNA helicase, putative	1.00	323	18	7	7	6.3	ALAQSLEREVTLPTLSQK	27.0	1.00	234	8	5	4	4.0	AQAALPSAVLQNFIADISPFLLSALR	32.7
									ALINQAVADVCAR Carbamidomethyl (C11)	50.1							NLKDAASSIQSWLK	39.7
									KAHLPAASILTAIMPDESFTAQEEGYSAGAPR Oxidation (M14)	11.5							SLPELAQPQPVQLQEYPYLKHELVEHLAK	37.6
									LALTAASECPGGSR Carbamidomethyl (C9)	61.9							SVVLAGDHILLNPTQFR	81.5
									LKEQAVDTPAKAR	56.3							SVVLAGDHILLNPTQFR Deamidated (N12)	25.5
									SLPELAQPQPVQLQEYPYLKHELVEHLAK	22.7								
									SVVLAGDHILLNPTQFR	67.0								
118	Tb927.8.5200	hypothetical protein, conserved	1.00	198	11	6	6	4.9	GRPPSLLLANVLSK	19.9	1.00	142	6	3	3	3.8	GLANCPEQESVGDDFAVPVSLILSWDGPGSSGGSGSGDRR Carbamidomethyl (C5)	59.1
									KGSAALSSAIR	19.5							GRPPSLLLANVLSK	48.4
									LDTSAIVPYKVDSK	16.7							LDTSAIVPYKVDSK	13.9
									LQLETSDGTLAHSGSGGSR	37.2								
									TIAANPALQYLLK	18.5								
									YTLYQAGAHGAQLSNPR	29.7								
119	Tb927.9.5730	nucleosome assembly protein-like protein	1.00	280	17	6	5	18.7	AAIVCGTRDITDEEIAAAQASLAAETTSSGVR Carbamidomethyl (C5)	43.6	1.00	138	10	4	4	16.1	DRPALSHLQDIQLEHIDGDPHKGVR	32.1
									AAIVCGTRDITDEEIAAAQASLAAETTSSGVR Carbamidomethyl (C5)- Deamidated (Q19)	45.1							INFHFSPNEYFTNEVLSK	22.1
									GLLNEYKSVR	50.7							SVEETALALIKK	38.3
									INFHFSPNEYFTNEVLSK	28.4							VVSPQEDPGRLEK	18.3
									NVYALKGLLNEYKSVR	48.0								
									VVSPQEDPGRLEK	20.7								
120	Tb927.9.12730	chaperone protein DNAj, putative	1.00	433	29	10	7	27.3	FGPGMVQQTVSQCPR Oxidation (M5)- Carbamidomethyl (C13)	38.9	1.00	165	14	5	4	13.8	ILGVSQSASQSDIKK	48.5
									ILGVSQSASQSDIKK	61.6							SAEDIFAEFFKGGMGGFGGNR Oxidation (M14)	19.3
									IYDQYGSEAAANMNAGGGMGGFGGR Oxidation (M13)- Oxidation (M19)	41.9							SAGPPQVPPLEVTLR	37.0
									SAEDIFAEFFKGGMGGFGGNR Oxidation (M14)	30.7							SSVLQPNSVLR	8.6
									SAGPPQVPPLEVTLR	38.7							SSVLQPNSVLR Deamidated (N7)	27.0
									SAGPPQVPPLEVTLR Deamidated (Q6)	27.0								
									SSVLQPNSVLR	52.2								
									VAGEGMPNSSGGNGDLYIVTK Oxidation (M6)	14.2								
									VAGEGMPNSSGGNGDLYIVTK Oxidation (M6)- Deamidated (N13)	20.7								
									VAGEGMPNSSGGNGDLYIVTK Oxidation (M6)- Deamidated (N8)	39.0								
121	Tb927.8.3380	electron transfer protein, putative	1.00	229	14	6	5	28.9	AHQDPTLAFR	24.1	1.00	414	14	7	5	31.8	AHQDPTLAFR	34.9
									SGAQTDVMALLQLLDAVCESGNVTHLIR Deamidated (Q4)- Oxidation (M8)- Carbamidomethyl (C18)	18.4							ALIEETLQNYK	21.9
									SGAQTDVMALLQLLDAVCESGNVTHLIR Oxidation (M8)- Carbamidomethyl (C18)	16.1							SGAQTDVMALLQLLDAVCESGNVTHLIR Carbamidomethyl (C18)	80.9
									SRLDNIVER	37.8							SGAQTDVMALLQLLDAVCESGNVTHLIR Deamidated (Q12)- Carbamidomethyl (C18)	78.3
									TITKVIHGVPSDDEAQLAQLESK	37.3							SRLDNIVER	42.1
									VIHGVPSDDEAQLAQLESK	57.7							VIHGVPSDDEAQLAQLESK	62.3
																	VIHGVPSDDEAQLAQLESK Deamidated (Q15)	62.8
122	Tb927.11.11010	hypothetical protein, conserved	1.00	435	32	13	12	37.6	ANEKAYDSALLATNAEFAR	60.0	1.00	56	4	3	3	13.2	ANEKAYDSALLATNAEFAR	3.0
									ASNLKELEAK	19.7							HINLQILDEDAALKR	29.4
									ASNLKELEAKR	20.5							THDYDEPQELIR	9.0
									AYDSALLATNAEFAR	34.6								
									EKYEWQLSHLDGV	26.1								
									HINLQILDEDAALKR	46.7								
									KRNEEAAASAVLIR	11.9								
									LMQIENEENHSLFK Oxidation (M2)	42.6								
									LMQIENEENHSLFK Oxidation (M2)- Deamidated (N6)	10.0								
									PLPPVEDAPNSMAR Oxidation (M12)	18.1								
									SSVPLHTDTTRPLPGAAR	18.1								
									SSVPLHTDTTRPLPGAARR	21.8								
									THDYDEPQELIR	31.7								
123	Tb927.4.1500	RNA editing associated helicase 2	1.00	171	9	6	6	4.4	ASGVAWNKEASQR	8.2	0.00	0	0	0	0	0.0		
									GVQGFQALMDLIR Oxidation (M9)	35.2								
									IKNYLASVGR	18.5								
									SVAAAPDVGTSDPTAPQNTK	43.7								
									TGDETYDAALAEVESMPIDPDAKTVLQR Oxidation (M16)	38.5								
									TQFEQALYSPR	17.1								
124	Tb927.3.3310	60S ribosomal protein L13, putative	0.99	455	37	9	9	31.2	GFSLEELKAAGVKPR	46.4	0.41	32	4	1	1	7.3	SRYGTAAVGGLVTPAR	12.0
	Tb927.3.3320	60S ribosomal protein L13, putative							LGRGFSLEELKAAGVKPR	32.2								
									LVLFPLNR Deamidated (N7)	10.0								
									PKGNNAIPHVHQR	42.8								
									SRYGTAAVGGLVTPAR	30.1								
									SRYGTAAVGGLVTPAREAPR	52.2								
									VFFNQPAQK	31.9								
									YGTAAVGGLVTPAR	55.1								
									YGTAAVGGLVTPAREAPR	35.3								
125	Tb927.11.5450	malic enzyme, putative	1.00	186	12	5	4	13.1	ILPVMMDVGTDNEGLRADPQYLGLR Oxidation (M5)- Oxidation (M6)	19.7	1.00	287	20	5	5	18.4	EREHLGVVGLLPPAEETLDDHVKR	28.2
									IVFFGAGSAATGVATCIVDLVAAK Carbamidomethyl (C16)	25.1							FVKPTALIGLGAQGGVFTEDILK	29.7
									NIPQEVLQTAAVTLSK	52.6							IVFFGAGSAATGVATCIVDLVAAK Carbamidomethyl (C16)	39.1
									NIPQEVLQTAAVTLSK Deamidated (N1)	41.6							QVAAAVIEKLQEMGLAK	65.9
									YLEPEYYLSK	24.4							SYCEKPVIFALSNPSSK Carbamidomethyl (C3)	50.5
126	Tb927.2.450	retrotransposon hot spot protein 4 (RHS4), putative	0.00	109	6	4	4	4.7	AAQTAASAVEIK	20.4	0.96	189	8	4	4	6.4	AAQTAASAVEIK	35.4
									AAQTAASAVEIKGVYDSIYNAK	18.3							AAQTAASAVEIKGVYDSIYNAK	60.0
									AREEVVEGR	12.6							HIVIGTPGIGK	25.0
									HIVIGTPGIGK	14.2							NRDGAAPPTTPNIAVLLQVTK	30.1
									IDMVGPLPR Oxidation (M3)	54.0							VSSGTVNVDSYR	50.4
127	Tb927.10.13800	hypothetical protein, conserved	1.00	256	10	4	4	26.3	IQIDEVREKYTAAPAQTSGEAANADCSQ Carbamidomethyl (C26)	4.5	1.00	83	7	5	5	21.7	LGKQVGSNASQVAPR	4.7
									LGKQVGSNASQVAPR	58.6							SFAEAIKLGKQVGSNASQVAPR Deamidated (Q11)	9.1
									SFAEAIKLGKQVGSNASQVAPR	63.6							SSGKPNGREGPNR Deamidated (N6)- Deamidated (N12)	13.7
									VSSTTQPAAEAAVEKPADSGAPAVPDAEAETRRPK	97.4							VSSTTQPAAEAAVEKPADSGAPAVPDAEAETR	33.0
																	VSSTTQPAAEAAVEKPADSGAPAVPDAEAETRRPK	10.4
128	Tb927.10.14600	40S ribosomal protein S2, putative	0.97	229	17	6	6	24.8	FKAFNVVGDGNGHIGVGAR	41.6	0.00	0	0	0	0	0.0		

	Tb927.10.14710	40S ribosomal protein S2, putative							GAGIVAAPVPK	32.2								
									GAGIVAAPVPKK	41.9								
									GAGIVAAPVPKKILEFAGVEDVYTSSCGK Carbamidomethyl (C27)	8.2								
									LSIVPVRR	29.9								
									RGGRGGR	31.4								
									VGKEVSLAIR	27.9								
129	Tb927.9.3170	cytochrome oxidase subunit V	1.00	420	36	10	9	48.0	GAASLLESLYR	73.9	0.00	4	1	1	1	6.1	VFLPPHLGDPHR	4.4
									GAEIPDHVFQTPAVIERPEIFKDGNK Deamidated (N25)	29.3								
									GAEIPDHVFQTPAVIERPEIFKDGNKISN Deamidated (N25)	19.2								
									KPEVNDRIR	26.9								
									SLLETMKWEATPR	60.9								
									SLLETMKWEATPR Oxidation (M6)	41.2								
									SQYASTMDPRDADVLR Oxidation (M7)	40.3								
									SQYASTMDPRDADVLRR Oxidation (M7)	28.2								
									VFLPPHLGDPHR	17.1								
									VFLPPHLGDPHRLLK	23.0								
130	Tb927.10.520	hypothetical protein, conserved	1.00	494	49	10	7	21.0	AAIAKGDNAAAVEGR	65.1	1.00	209	14	4	2	8.6	AAIAKGDNAAAVEGR	67.8
									AAIAKGDNAAAVEGR Deamidated (N8)	82.8							AAIAKGDNAAAVEGR Deamidated (N8)	67.1
									AAIAKGDNAAAVEGRHTA	46.0							RLEFENGPLRDQVEAHTQR	21.9
									AAIAKGDNAAAVEGRHTA Deamidated (N8)	38.9							RLEFENGPLRDQVEAHTQR Deamidated (N6)	17.3
									LLAERAAIAKGDNAAAVEGR	13.5								
									MQGSWSVLK	23.3								
									RLEFENGPLRDQVEAHTQR	22.7								
									RLEFENGPLRDQVEAHTQR Deamidated (N6)	31.6								
									SMNIPTFPMLAR Oxidation (M2)- Oxidation (M9)	26.3								
									YGPTDFVEQSETFSPDYHKR	25.8								
131	Tb927.11.1090	calpain-like protein, putative,cytoskeleton assocociated protein, putative	1.00	248	31	8	7	2.2	ASGEVPIALTIVGSPDANVR	20.3	0.39	53	5	2	2	0.4	LIAEDREGNAAR	21.8
									IAELEAAMNEHSHELAK Oxidation (M8)	25.6							LIAEDREGNATR	22.7
									IAELEAAMNEHSHKLAK Oxidation (M8)	36.8								
									IAELEAAMNEHSHKLAK Oxidation (M8)- Deamidated (N9)	21.3								
									IAELEVAMNEHSHELAK Oxidation (M8)	35.9								
									LIAEDREGNAAR	25.1								
									LIAEDREGNATR	20.9								
									YLNPEPEGVPLELLPLNEDEVVSEKEDRLR	8.5								
132	Tb927.8.7530	3,2-trans-enoyl-CoA isomerase, mitochondrial precursor, putative	1.00	150	7	4	4	16.8	FLSVPQQSR	30.7	1.00	344	13	7	6	18.4	IGLNETKLGITTPPWVVPAYAYVLGSR	23.7
									IGLNETKLGITTPPWVVPAYAYVLGSR	29.4							LGITTPPWVVPAYAYVLGSR	36.5
									LGITTPPWVVPAYAYVLGSR	8.6							MLQLGDTPTADEALRVGLVDLVVDGEQQLR Deamidated (Q28)	42.9
									VGLVDLVVDGEQQLREAVLKEAER	54.4							VGLVDLVVDGEQQLR Deamidated (Q12)	62.7
																	VGLVDLVVDGEQQLREAVLK	35.7
																	VGLVDLVVDGEQQLREAVLKEAER	65.9
																	VGLVDLVVDGEQQLREAVLKEAER Deamidated (Q12)	47.3
133	Tb927.10.450	hypothetical protein, conserved	1.00	104	9	4	4	14.4	IVAGHEPEQTNALLQLLASCAALPSDKK Carbamidomethyl (C20)	11.5	0.99	41	2	1	1	4.8	IVAGHEPEQTNALLQLLASCAALPSDKK Carbamidomethyl (C20)	37.1
									MFVNPDALTR Oxidation (M1)	18.1								
									NIISNDNAIAGMVR Oxidation (M12)	35.3								
									VAEQQAARPTKDSDGTLQDGEAKGYLGQQALR	29.5								
134	Tb927.11.13090	elongation factor 1 gamma, putative	0.99	342	29	9	7	21.0	ITDYLCWEGPTIPKPVLEGR Carbamidomethyl (C6)	55.7	0.75	111	7	2	2	8.7	ITDYLCWEGPTIPKPVLEGR Carbamidomethyl (C6)	36.8
	Tb927.11.13190	elongation factor 1 gamma, putative							KKPNPLDELPPSPFVLDAFKR	34.4							LLVVAAFANVPVTVK	49.3
									KKPNPLDELPPSPFVLDAFKR Deamidated (N4)	16.8								
									LDKSGSFLYGR	36.2								
									LLVVAAFANVPVTVK	30.1								
									LLVVAAFANVPVTVK Deamidated (N9)	4.1								
									SQGATFGPVKAK	43.8								
									TVEVLKSQGATFGPVK	26.5								
									TVEVLKSQGATFGPVKAK	38.6								
135	Tb927.7.1730	60S ribosomal protein L7, putative	1.00	433	29	8	8	38.8	AAFKQQQTENFKK	34.0	0.86	244	17	7	6	29.8	AAFKQQQTENFKK	33.5
									AIAANKVAAAALK	55.4							AIAANKVAAAALK	37.2
									AIAANKVAAAALKK	76.1							AIAANKVAAAALKK	51.5
									AVEPYIAYGYPSLSTVR	58.4							AIAANKVAAAALKK Deamidated (N5)	24.6
									AVPAPESAIKR	25.1							AVEPYIAYGYPSLSTVR	20.6
									HFVEGGDYGNRDTLINR	29.2							AVPAPESAIKR	21.0
									PAKAVPAPESAIKR	37.1							HFVEGGDYGNRDTLINR	23.9
									TVTNGLWPFKLAPPAGGMR Deamidated (N4)- Oxidation (M18)	16.1								
136	Tb927.6.1520	aquaporin 3, putative	1.00	173	7	3	1	10.3	SANPQEKNENELVGDNADNEAHDAVDVNYWAPR	57.1	1.00	243	6	5	1	10.3	SANPQEKNENELVGDNADNEAHDAVDVNYWAPR Deamidated (N10)	45.2
									SANPQEKNENELVGDNADNEAHDAVDVNYWAPR Deamidated (N16)	52.3							SANPQEKNENELVGDNADNEAHDAVDVNYWAPR Deamidated (N16)- Deamidated (N19)	43.5
									SANPQEKNENELVGDNADNEAHDAVDVNYWAPR Deamidated (Q5)	50.0							SANPQEKNENELVGDNADNEAHDAVDVNYWAPR Deamidated (N19)	40.2
																	SANPQEKNENELVGDNADNEAHDAVDVNYWAPR Deamidated (N3)	59.1
																	SANPQEKNENELVGDNADNEAHDAVDVNYWAPR Deamidated (Q5)	54.7
137	Tb927.11.6250	hypothetical protein, conserved	1.00	190	20	5	4	23.1	AAPDSVQLR	15.4	1.00	237	9	3	2	16.4	SAQDPLFSGVSTVVVR	81.4
									SAQDPLFSGVSTVVVR	64.3							SAQDPLFSGVSTVVVR Deamidated (Q3)	52.5
									SAQDPLFSGVSTVVVR Deamidated (Q3)	35.6							SVALAVAQELRELGLSSEADKVEAAASK	49.0
									SVALAVAQELRELGLSSEADKVEAAASK	8.9								
									VAGGRVQTK	27.7								
138	Tb927.11.3590	40S ribosomal protein S4, putative	0.97	268	25	8	7	33.3	ATNIFVIGKDMSSVPVTLPK Deamidated (N3)	22.1	0.75	115	6	2	2	11.0	IGEIVSIERHPGAFDIAR	24.0
	Tb927.11.3600	40S ribosomal protein S4, putative							ATNIFVIGKDMSSVPVTLPK Oxidation (M11)	25.1							LKDASGHEFATR	38.3
									HPGAFDIAR	21.9								
									IGEIVSIERHPGAFDIAR	42.2								
									INVIQEREEK	8.4								
									LKDASGHEFATR	27.1								
									LTGVFAPRPR	44.9								
									VVNVYTGTGRIPVAVTHDGHR	30.5								
139	Tb927.7.3550	hypothetical protein, conserved	1.00	330	26	9	8	8.5	CGAFEGQTAKVPR Carbamidomethyl (C1)	29.6	0.27	33	2	2	2	2.6	AAEGKPSTSEAESSDVGAAANTR	24.1
									DGSGPRESLFLTVVR	34.0							LIVTVHHGK	9.2
									IVSSGRFELLNDSDALTVK	32.7								
									LIVTVHHGK	18.1								
									LMQINIIEER Oxidation (M2)	27.4								
									SQMLQLITPK Oxidation (M3)	30.1								
									TSNLQRVMEDEELNQPAIPLPPTTLDELRK Oxidation (M8)	23.2								
									VMEDEELNQPAIPLPPTTLDELRK	27.3								
									VMEDEELNQPAIPLPPTTLDELRK Oxidation (M2)	47.5								
140	Tb927.3.4760	dynamin, putative,vacuolar sortin protein 1, putative	1.00	346	17	7	7	14.7	CPLVLQLVQLPR Carbamidomethyl (C1)	50.0	1.00	210	8	5	4	8.0	CPLVLQLVQLPR Carbamidomethyl (C1)	46.5
									FFDFSEINEEIQNR	38.1							FFDFSEINEEIQNR	52.3
									LISVVNDLHDAFANVK	40.6							FFDFSEINEEIQNR Deamidated (N8)	38.8
									SATTAMMTALTK Oxidation (M6)- Oxidation (M7)	43.7							SSVLEAIVGKDFLPR	31.3
									SSVLEAIVGKDFLPR	51.4							TVLDSVREFALI	33.3
									TVGELLAEPPQIAQQR	39.2								
									TVLDSVREFALI	48.1								
141	Tb927.9.10310	mitochondrial carrier protein, putative,mitochondrial phosphate transporter, putative	1.00	219	22	4	4	23.0	ADPAAGFPFKSLVPLWSR	19.4	1.00	212	10	6	3	17.7	CIGGGVLSCGLTHTAVCPLDVVK Carbamidomethyl (C1)- Carbamidomethyl (C9)- Carbamidomethyl (C17)	36.7
									CIGGGVLSCGLTHTAVCPLDVVK Carbamidomethyl (C1)- Carbamidomethyl (C9)- Carbamidomethyl (C17)	39.3							GIGSGFKVLAAEDGFGAK	32.1
									SYGQIANEIGYGNLCTK Carbamidomethyl (C15)	60.4							YANPDIPHNNSYYLK	33.7
									YANPDIPHNNSYYLK	45.1							YANPDIPHNNSYYLK Deamidated (N10)	31.3
																	YANPDIPHNNSYYLK Deamidated (N3)	24.7
																	YANPDIPHNNSYYLK Deamidated (N3)- Deamidated (N9)	29.4

142	Tb927.9.10400	hypothetical protein, conserved	1.00	315	21	6	6	10.1	ESNYIEAADALKASNPSVEK	55.0	1.00	111	7	2	2	4.9	ESNYIEAADALK	49.3
									ESNYIEAADALKASNPSVEKAGR	47.0							LPKDELDAQVYR	51.1
									GLKFSALIAAPK	31.4								
									LPKDELDAQVYR	52.6								
									YLRESNYIEAADALK	25.9								
									YLRESNYIEAADALKASNPSVEK	36.5								
143	Tb927.10.14500	hypothetical protein, conserved	1.00	324	25	7	7	22.9	AAADAENFETLKR	42.6	0.00	20	2	2	2	12.2	TGKGPRPIPFDAPEEQVRVK	5.1
									ATLLKNQPTTTN	33.8							TVARDPANRDNAAALGAVLHR	15.1
									LLVLEHKSR	34.6								
									TGKGPRPIPFDAPEEQVR	31.5								
									TGKGPRPIPFDAPEEQVRVK	31.0								
									TIGTLNTYGPRPEGAGVVWCR Carbamidomethyl (C20)	26.6								
									VKAAADAENFETLKR	46.9								
144	Tb927.7.1300	protein disulfide isomerase, putative	1.00	251	19	5	5	18.0	AAAGAKDKVLIAK	41.6	1.00	90	6	2	2	6.1	AFVSYLNNQIK	15.8
									AFVSYLNNQIK	47.1							LLAQIQQAVSPK	61.8
									ALGEGAMHYIR Oxidation (M7)	58.7								
									IATNVLENGHEYISKEHER Deamidated (N8)	35.2								
									YKVEGYPTLVFFPK	39.6								
145	Tb927.10.3940	40S ribosomal protein S3A, putative	1.00	371	27	9	9	33.2	FTVQEVQGR	44.4	0.00	0	0	0	0	0.0		
									IAADFLR	28.8								
									IAADFLRGR	23.7								
									KEWYDVVAPK	45.2								
									LAKLDINDAVSLLTR	18.2								
									NFEVRQFAK	30.2								
									TICNKTQGTKIAADFLR Carbamidomethyl (C3)	30.0								
									VKFTVQEVQGR	30.0								
									VYEGNLADLNKTQNEDDAYRK	24.4								
146	Tb927.2.4710	RNA-binding protein, putative	1.00	257	23	6	5	17.0	FRDDYKVAVR	28.7	1.00	258	31	4	4	12.0	NFFAAVGEPVDIFIHER	66.8
									HLPDDATEQQLR	24.9							NMFQSAGTILDFFLEPR	52.5
									NFFAAVGEPVDIFIHER	53.5							VAGLADGTADDAVR	40.0
									NFFAAVGEPVDIFIHER Deamidated (N1)	35.5							VAGLADGTADDAVREFFTR	26.6
									NMFQSAGTILDFFLEPR Oxidation (M2)	34.3								
									VAGLADGTADDAVREFFTR	17.5								
147	Tb927.11.2060	60S acidic ribosomal subunit protein, putative	0.98	286	25	8	7	34.9	ATAFFQALNIATK	58.9	0.85	134	5	4	3	13.6	ATAFFQALNIATK	57.1
	Tb927.11.2050	60S acidic ribosomal subunit protein, putative							DLRGKGELVMGK Oxidation (M10)	21.4							ATAFFQALNIATK Deamidated (N9)	36.0
									LGGGEAAAPAAAAAAAAPAAAPAAAAAEEEEDDDDFGMGALF Oxidation (M38)	35.1							VGAIAPCDVIVPAGNTGMEPK Carbamidomethyl (C7)- Oxidation (M18)	5.6
									SQQVHGVR	15.7							VLFCLMDNVR Carbamidomethyl (C4)	31.6
									TAALEGKLGGGEAAAPAAAAAAAAPAAAPAAAAAEEEEDDDDFGMGALF Oxidation (M45)	31.0								
									VGAIAPCDVIVPAGNTGMEPK Carbamidomethyl (C7)- Oxidation (M18)	25.8								
									VLFCLMDNVR Carbamidomethyl (C4)	33.4								
									VLFCLMDNVR Carbamidomethyl (C4)- Oxidation (M6)	28.5								
148	Tb927.4.2450	thioredoxin, putative	1.00	381	16	7	5	14.8	LALPVRPLPASAHGLENFCSPDAR Carbamidomethyl (C19)	46.0	1.00	64	3	1	1	4.6	SAGAIQSQAISLINAPGIK	55.3
									SAGAIQSQAISLINAPGIK	80.0								
									SAGAIQSQAISLINAPGIK Deamidated (Q6)	74.4								
									SAGAIQSQAISLINAPGIK Deamidated (Q6)- Deamidated (N14)	7.1								
									SAGAIQSQAISLINAPGIKTVK	32.6								
									SRVPPIYAVLSLSPR	35.7								
									VPPIYAVLSLSPR	59.2								
149	Tb927.4.4210	ATP-dependent zinc metallopeptidase, putative,metallo-peptidase, Clan MA(E) Family M41	1.00	292	17	7	7	11.4	ALINSYAER	37.1	0.92	48	3	2	2	5.0	TGAGIGEKQEQEHTLNELLTQLDGFSSSR	9.3
									HLLENEVLTFDDVVR	9.6							VPTGALLLGPPGTGK	35.8
									MAYMFVSSFAPRPVYPAPGTAGTR Oxidation (M1)- Oxidation (M4)	45.4								
									SRVLSPFER	29.8								
									VAEKIFFNHLSTGASDDLNKVAR	43.7								
									VPTGALLLGPPGTGK	56.2								
									VPTGALLLGPPGTGKTLLAK	17.4								
150	Tb927.10.7500	fibrillarin	1.00	238	12	5	4	21.0	GGGGRGGGAGAKVVVEPHLLHPGVFISK	27.3	1.00	160	6	3	2	13.3	LKPLEQASLEPFERDHAVVVGVYR	41.0
									ILALNAQAFLK	37.2							LKPLEQASLEPFERDHAVVVGVYR Deamidated (Q6)	53.1
									LKPLEQASLEPFERDHAVVVGVYR	43.9							VVVEPHLLHPGVFISK	40.2
									LKPLEQASLEPFERDHAVVVGVYR Deamidated (Q6)	33.6								
									VVVEPHLLHPGVFISK	42.9								
151	Tb927.11.3490	hypothetical protein, conserved	1.00	287	17	6	6	9.2	EADQVELSSGTPIVR	40.8	1.00	141	6	2	2	4.4	NVLEQLEIVDDKAVDR	50.0
									GAKEADQVELSSGTPIVR	60.6							SIVEKSEQLAVDTATEVVK	36.5
									HGGAPTQPVDPDALVAR	26.5								
									NVLEQLEIVDDKAVDR	35.9								
									QQASQFCETLR Carbamidomethyl (C7)	21.0								
									SIDERVVNLQK	31.7								
152	Tb927.11.2670	Nucleoporin	1.00	271	13	5	5	12.5	AQLLEWLDANDPLYDR	15.4	1.00	125	3	3	2	5.7	AQLLEWLDANDPLYDR	50.1
									AQLLEWLDANDPLYDRSGAPPLTISEVLSTK	70.9							AQLLEWLDANDPLYDRSGAPPLTISEVLSTK	55.3
									IAEKQTFR	31.9							AQLLEWLDANDPLYDRSGAPPLTISEVLSTK Deamidated (N10)	20.0
									SSTAAGAAAAAAR	74.0								
									VVRDDGANINAFLLQK	40.6								
153	Tb927.10.3650	NADH-dependent fumarate reductase, putative	0.89	242	11	6	5	5.4	ATSGINGWGTR	44.4	1.00	200	8	5	5	4.6	ATSGINGWGTR Deamidated (N6)	32.7
									ATSGINGWGTR Deamidated (N6)	42.2							FNLPGALQR	42.4
									ELLSSNSGLCQEDEPTIINLKGLEHTIPYR Carbamidomethyl (C10)	28.2							FVNELDLR	25.5
									FVNELDLR	39.4							RFVNELDLR	29.3
									FVNELDLRSVVSK	33.7							SGDAIGWLTSLGVPLSVLSQLGGHSFKR	34.1
									VLRFNLPGALQR	20.6								
154	Tb927.5.1300	vacuolar proton translocating ATPase subunit A, putative	0.48	31	2	1	1	2.7	LGQLAAFQFIDLNSDVSAFQR Deamidated (Q3)- Deamidated (Q8)	24.6	1.00	361	18	7	5	12.1	GNSVLHTDEITTPFSEGEKER	42.7
																	ISEVNGASLYPYAESTER	50.4
																	ISEVNGASLYPYAESTER Deamidated (N5)	62.7
																	LGQLAAFQFIDLNSDVSAFQR	47.8
																	LGQLAAFQFIDLNSDVSAFQR Deamidated (Q8)	46.6
																	QSPGLNLLTGVIPK	39.6
																	YLHDEIEKAGLTCVSTEAIGR Carbamidomethyl (C13)	19.8
155	Tb927.3.4970	hypothetical protein, conserved	0.98	102	6	5	5	10.5	AAVNKGSVNAAAPGTIDRR	10.1	0.91	40	4	2	2	5.8	EIAPSYTPVVDEVARDDAAAALEQVELLEK	24.9
									EIAPSYTPVVDEVARDDAAAALEQVELLEKYVVR	20.2							ERSPTVMSKGQTGAGGSGPGPSGAVESDLLQK Oxidation (M7)	6.0
									ERSPTVMSKGQTGAGGSGPGPSGAVESDLLQK Oxidation (M7)	4.4								
									SIAKVQGGDLNVTAPK	36.1								
									VLALEKEVESR	30.6								
156	Tb927.8.4810	prohibitin 1	1.00	245	23	5	5	17.0	AAVVRAEGEAESAR	20.4	1.00	119	4	3	3	15.5	AAVVRAEGEAESAR	18.0
									AAVVRAEGEAESARLISEAIQR	19.7							ILGLDEIKVFNIR	61.4
									LISEAIQR	41.8							NVTFLPGGSNMLLHMK Deamidated (N10)	29.8
									NVTFLPGGSNMLLHMK Oxidation (M11)- Oxidation (M15)	21.5								
									SGGGLLELR	77.9								
157	Tb927.10.3990	DHH1	1.00	163	16	5	4	16.0	ELQMGIFEKGFERPSPVQEEAIPVALQGKDVLAR Deamidated (Q3)- Oxidation (M4)	13.8	1.00	237	14	6	4	20.7	GFERPSPVQEEAIPVALQGKDVLAR	49.9
									ELQMGIFEKGFERPSPVQEEAIPVALQGKDVLAR Oxidation (M4)	33.7							GFERPSPVQEEAIPVALQGKDVLAR Deamidated (Q18)	36.4
									GFERPSPVQEEAIPVALQGKDVLAR	43.4							GFERPSPVQEEAIPVALQGKDVLAR Deamidated (Q9)	12.5
									IIVLDEADKLLSQEFTSLMR	12.0							IIVLDEADKLLSQEFTSLMR	62.3
									NVTFEEYGLRR	25.7							NVTFEEYGLRR	11.2
																	TASFVIPVLEKVDTQLPHIQALLMVPTR	24.3

158	Tb927.11.8260	carbonic anhydrase-like protein	1.00	64	4	1	1	2.6	AAFNKLSQVLR	59.2	1.00	197	12	4	3	10.2	AAFNKLSQVLR<br>AAFNKLSQVLR Deamidated (N4)<br>FVIMTTPQLISK<br>QSYVTYSGSLTTPPCTEGVR Carbamidomethyl (C15)	56.8<br>56.0<br>39.5<br>26.6
159	Tb927.8.900	splicing factor TSR1	1.00	301	35	7	7	18.6	LEQVAPVSDVR<br>RGECAFVDVPSPAHAR Carbamidomethyl (C4)<br>RRGECAFVDVPSPAHAR Carbamidomethyl (C5)<br>SISDRELR<br>SKLEEAAPIIGFR<br>SKLEEAAPIIGFRR<br>TALFVGLGPSGR	41.9<br>14.8<br>20.3<br>27.6<br>51.3<br>19.4<br>57.2	1.00	127	7	3	3	11.9	LITEMDGKYIGEAR Oxidation (M5)<br>SKLEEAAPIIGFR<br>TALFVGLGPSGR	9.1<br>41.1<br>57.2
160	Tb927.9.2900	hypothetical protein, conserved	1.00	212	13	6	5	3.3	HLVETVPGIGR<br>IIASGGSQHAEAALLNVNR<br>LMAEMLPSVAELTEDRDDVVVEQAR Oxidation (M2)- Oxidation (M5)<br>MGADALSLLVGTVGGYQR<br>MGADALSLLVGTVGGYQR Oxidation (M1)<br>VILSSGAGAGETPSR	39.5<br>8.2<br>29.3<br>20.7<br>19.3<br>67.6	0.00	14	1	1	1	0.7	MGADALSLLVGTVGGYQR	14.1
161	Tb927.9.4210	fatty acyl CoA synthetase 3	1.00	259	22	8	8	12.5	AFDHAFQSR<br>ALAYRPVDRVEK<br>ALGKVAVPVPGSETSDCSPIYR Carbamidomethyl (C17)<br>ATASFQETAR<br>GDLAEYNPSLLIGVPR<br>RVIDERYTDIIAPLFVEEC Carbamidomethyl (C19)<br>VAVPVPGSETSDCSPIYR Carbamidomethyl (C13)<br>VIDERYTDIIAPLFVEEC Carbamidomethyl (C18)	39.5<br>20.6<br>26.6<br>13.5<br>17.5<br>18.1<br>46.2<br>33.5	1.00	214	13	6	5	11.5	ALAYRPVDRVEK<br>ATASFQETAR<br>IALSGGGPLSTATQDFVNVVLAR<br>IALSGGGPLSTATQDFVNVVLAR Deamidated (Q14)<br>VAVPVPGSETSDCSPIYR Carbamidomethyl (C13)<br>VIDERYTDIIAPLFVEEC Carbamidomethyl (C18)	20.5<br>25.1<br>53.6<br>33.6<br>30.1<br>11.7
162	Tb927.3.1120	GTP-binding nuclear protein rtb2, putative	1.00	211	18	4	3	31.8	KLANDPNLTLVEAPMLDPNVQPLTAEQLQALQEEAR Deamidated (N7)- Oxidation (M15)<br>KLANDPNLTLVEAPMLDPNVQPLTAEQLQALQEEAR Oxidation (M15)<br>SNYNFEKPFLWLAK<br>VCDNIPIVLVGNKVDCAER Carbamidomethyl (C2)- Carbamidomethyl (C16)	55.4<br>59.8<br>22.8<br>36.3	1.00	57	2	1	1	8.8	VCDNIPIVLVGNKVDCAER Carbamidomethyl (C2)- Carbamidomethyl (C16)	39.2
163	Tb927.5.3230	hypothetical protein, conserved	1.00	218	20	4	4	4.2	AALIEACNVSMDPEHLLLR Carbamidomethyl (C7)- Oxidation (M11)<br>ALTQCLSTYELIAPQVIDKNVPAILQIASR Carbamidomethyl (C5)<br>SLAAVAVASGELSDTAAKLR<br>VVLPQLLTSCHDVAHETRPVVER Carbamidomethyl (C10)	32.3<br>31.7<br>50.5<br>35.7	0.00	12	1	1	1	0.8	STSMALGFTVGPASSAR Oxidation (M4)	12.3
164	Tb927.11.1270	hypothetical protein, conserved	1.00	240	10	4	3	17.2	ATLPASQQAIYNY<br>LLSTGKIEEYLPVNIK<br>LLSTGKIEEYLPVNIKATLPASQQAIYNY<br>LLSTGKIEEYLPVNIKATLPASQQAIYNY Deamidated (N28)	44.7<br>64.5<br>55.0<br>62.3	0.00	0	0	0	0	0.0		
165	Tb927.2.3780	translation initiation factor IF-2, putative	1.00	104	10	5	5	9.2	AIAVFPVQLTILDDAIHNTDPIILPVK<br>AIAVFPVQLTILDDAIHNTDPIILPVKVK<br>AKEHVLESVTK<br>MAAAGFIVPDVEKVR<br>SPICCVLGHVDTGKTSLLDRIR Carbamidomethyl (C4)- Carbamidomethyl (C5)	17.5<br>35.3<br>12.7<br>6.3<br>18.6	1.00	268	21	7	6	13.0	AIAVFPVQLTILDDAIHNTDPIILPVK<br>AIAVFPVQLTILDDAIHNTDPIILPVKVK<br>AKEHVLESVTK<br>IAANELEYVIPGSHLLVVRPGDDKEAVAK<br>IAANELEYVIPGSHLLVVRPGDDKEAVAK Deamidated (N4)<br>LFKDELTEDDINLLATLIK<br>SPICCVLGHVDTGKTSLLDR Carbamidomethyl (C4)- Carbamidomethyl (C5)	32.0<br>38.3<br>30.9<br>33.0<br>35.9<br>41.7<br>15.3
166	Tb927.11.13500	par1	1.00	239	18	6	6	15.7	AATPFVHPLQVHGVK<br>AILEKYER<br>AVAALIELAETAIATAENYSGYVDSR<br>AVEGEETAVELAIRDIPVGRA<br>AVVGGDVLEVER<br>KAAELVQLTAK	39.2<br>40.0<br>35.1<br>20.1<br>32.4<br>35.6	1.00	97	8	3	2	7.9	AVAALIELAETAIATAENYSGYVDSR<br>AVAALIELAETAIATAENYSGYVDSR Deamidated (N18)<br>AVEGEETAVELAIRDIPVGRA	48.7<br>14.0<br>17.6
167	Tb927.11.4480	radial spoke protein RSP4/6, putative	1.00	280	14	6	6	20.2	HSFVDGESTEACNEPCPVPPSELSR Carbamidomethyl (C12)- Carbamidomethyl (C16)<br>LAENERLFERAPPEIQTTIEQPDPYTTITTTR<br>LFERAPPEIQTTIEQPDPYTTITTTR<br>LSFWVTSNPAAQWTLLPDVTPQQICAGRR Carbamidomethyl (C25)<br>PVNPEPQDLEAMFAR Oxidation (M12)<br>SLEVPVQPEPGFVGVNR	38.3<br>31.2<br>49.9<br>69.7<br>14.5<br>43.8	0.00	4	1	1	1	2.9	SLEVPVQPEPGFVGVNR Deamidated (Q7)	3.6
168	Tb927.5.1470	NADH-cytochrome b5 reductase, putative	1.00	210	10	6	6	32.3	IKDNSGSEITYPFVPITPNNTK<br>ILVAGPPELIKEIAGEK<br>ILVAGPPELIKEIAGEKIFNEGMAPEQGPLK Oxidation (M23)<br>LISAQYESPDTR<br>SVGMLASGTGIAPMYR Oxidation (M4)- Oxidation (M14)<br>WLGGIGEINEEMIR Oxidation (M12)	40.5<br>24.5<br>29.6<br>60.0<br>34.5<br>8.3	1.00	124	8	4	4	22.1	IKDNSGSEITYPFVPITPNNTK<br>ILVAGPPELIKEIAGEK<br>ILVAGPPELIKEIAGEKIFNEGMAPEQGPLK<br>LISAQYESPDTR	7.3<br>10.8<br>24.5<br>55.3
169	Tb927.8.1890	cytochrome c1, heme protein, mitochondrial precursor	1.00	185	6	4	3	28.7	AAQFANNGSEPPDLQHSVFGK Deamidated (N6)<br>AAQFANNGSEPPDLQHSVFGK Deamidated (N7)<br>QIYTEVFAPCHPLGR Carbamidomethyl (C10)<br>QLASQYEVVDSAPDAQGMLNPRPGKPTDTLPIPFPNQR Oxidation (M18)	56.4<br>25.2<br>36.2<br>55.9	1.00	167	7	3	1	8.1	AAQFANNGSEPPDLQHSVFGK Deamidated (N6)<br>AAQFANNGSEPPDLQHSVFGK Deamidated (N6)- Deamidated (N7)<br>AAQFANNGSEPPDLQHSVFGK Deamidated (N7)	66.0<br>36.9<br>46.5
170	Tb927.11.2410	hypothetical protein, conserved	1.00	221	14	5	4	23.0	FKVVPSST<br>LYNNAVALR<br>LYNNAVALR Deamidated (N3)<br>QLASLIPVFIGSALPK<br>YAACSAIVAQQMATELAK Carbamidomethyl (C4)- Oxidation (M12)	37.6<br>50.9<br>44.4<br>23.9<br>39.8	1.00	150	8	5	5	27.9	EAGRPIKIQQEK<br>FKVVPSST<br>KTLNAEIKQLASLIPVFIGSALPK Deamidated (N4)<br>TLNAEIKQLASLIPVFIGSALPK<br>YAACSAIVAQQMATELAK Carbamidomethyl (C4)	33.3<br>14.2<br>34.1<br>28.2<br>28.2
171	Tb927.11.14730	metalloprotease, putative,cell division protein FtsH homologue, putative	1.00	333	22	8	7	13.2	AVAGEANAPFLSCSGADFIEIFGGSGPK Carbamidomethyl (C13)<br>AVAGEANAPFLSCSGADFIEIFGGSGPKR Carbamidomethyl (C13)<br>EAAPCIVFIDEIDAIGSR Carbamidomethyl (C5)<br>EAAPCIVFIDEIDAIGSRNQSGR Carbamidomethyl (C5)<br>IITGDAECKPKIQR Carbamidomethyl (C8)<br>VASLTAEEEAPEVK<br>VRELFAQAR<br>VRELFAQAR Deamidated (Q7)	39.3<br>55.7<br>41.6<br>24.3<br>13.8<br>27.7<br>33.6<br>34.7	1.00	201	16	5	4	9.0	AVAGEANAPFLSCSGADFIEIFGGSGPK Carbamidomethyl (C13)<br>AVAGEANAPFLSCSGADFIEIFGGSGPKR Carbamidomethyl (C13)<br>AVAGEANAPFLSCSGADFIEIFGGSGPKR Deamidated (N7)- Carbamidomethyl (C13)<br>EAAPCIVFIDEIDAIGSR Carbamidomethyl (C5)<br>VASLTAEEEAPEVK	21.5<br>41.4<br>10.3<br>46.9<br>25.1
172	Tb927.6.4090	chaperonin HSP60, mitochondrial precursor, putative	1.00	295	17	5	3	7.6	NALAEGIVAGGGAALLHASK<br>NALAEGIVAGGGAALLHASK Deamidated (N1)<br>NVIIRQPSGEPK<br>VIDAVNAARNALAEGIVAGGGAALLHASK<br>VIDAVNAARNALAEGIVAGGGAALLHASK Deamidated (N10)	71.9<br>5.7<br>28.1<br>67.2<br>35.4	1.00	285	13	5	4	9.6	NALAEGIVAGGGAALLHASK<br>NVIIRQPSGEPK<br>TGILIVRNAAR Deamidated (N8)<br>VIDAVNAARNALAEGIVAGGGAALLHASK<br>VIDAVNAARNALAEGIVAGGGAALLHASK Deamidated (N10)	72.4<br>37.2<br>29.5<br>54.8<br>36.5
173	Tb927.3.3490	high mobility group protein, putative	1.00	150	9	4	4	15.9	AAGAAWKELGPEER<br>AAGAAWKELGPEERK<br>AKNPGMQNTAILQTLGK<br>HSDLSIVEMSK Oxidation (M9)	53.8<br>34.5<br>10.7<br>28.5	1.00	49	3	1	1	5.2	AAGAAWKELGPEER	35.8
174	Tb927.1.4310	hypothetical protein, conserved	1.00	334	18	7	6	7.3	DAPNAAQSPYNIR<br>IDLYSWSQAPGSIYTPR<br>LLLSSQKPPALEEALDEADYPTAFVDRYVYGDEWR<br>LLLSSQKPPALEEALDEADYPTAFVDRYVYGDEWR Deamidated (Q6)<br>NLEVTCIARPLPK Carbamidomethyl (C6)<br>QGIPPQALHALFAEESVER<br>SFVADAVQSEIDTLISDEMAAR Oxidation (M19)	64.0<br>30.9<br>41.4<br>42.3<br>21.0<br>20.5<br>19.8	1.00	116	2	2	1	2.1	LLLSSQKPPALEEALDEADYPTAFVDRYVYGDEWR<br>LLLSSQKPPALEEALDEADYPTAFVDRYVYGDEWR Deamidated (Q6)	67.6<br>48.2
175	Tb927.7.1790	Adenine phosphoribosyltransferase, putative	1.00	257	22	5	5	36.5	EAEEQLEFVNSYITR<br>GYIIGAPLAVALGIPFVTAR<br>RFPSSFVPEGDDLKYLPMSR Oxidation (M18)<br>SIRNDSIPPR	49.0<br>65.7<br>27.6<br>22.5	1.00	142	8	2	2	15.2	EAEEQLEFVNSYITR<br>GYIIGAPLAVALGIPFVTAR	68.7<br>54.4

									VLIVDDFIGTGSTMLAALR	43.1								
176	Tb927.11.11360	guanine nucleotide-binding protein beta subunit- like protein,activated protein kinase c receptor	0.98	300	17	6	6	24.5	FSPSLDAPVIVSGGWDNLVK	56.7	0.96	271	14	6	4	15.7	FSPSLDAPVIVSGGWDNLVK	47.6
	Tb927.11.11370	guanine nucleotide-binding protein beta subunit-like protein,activated protein kinase c receptor							FSPSLDAPVIVSGGWDNLVKVWDLATGR	34.1							FSPSLDAPVIVSGGWDNLVK Deamidated (N17)	38.4
									GAHTDWVSCVR Carbamidomethyl (C9)	41.0							FSPSLDAPVIVSGGWDNLVKVWDLATGR	29.6
									GWVTSLACPQTPETATK Carbamidomethyl (C8)	60.4							IFDLENKDIIVELAPEHQGSK	32.0
									IFDLENKDIIVELAPEHQGSK	29.1							IFDLENKDIIVELAPEHQGSKK	35.1
									IFDLENKDIIVELAPEHQGSKK	45.1							IFDLENKDIIVELAPEHQGSKK Deamidated (Q18)- Deamidated (N6)	31.6
177	Tb927.9.10560	hypothetical protein, conserved	1.00	218	11	3	2	14.3	GRHSAFSAAEEVASEEGLNEASSR	61.1	0.00	0	0	0	0	0.0		
									HSAFSAAEEVASEEGLNEASSR	71.1								
									HSAFSAAEEVASEEGLNEASSR Deamidated (N17)	33.2								
178	Tb927.3.3670	RNA-binding protein, putative	1.00	261	22	7	7	12.8	AKPTGKQKNTK Deamidated (N9)	23.6	0.00	0	0	0	0	0.0		
									ISAAQAAAPGSLPYSK	34.0								
									ISAAQAAAPGSLPYSKAPIR	33.4								
									RISAAQAAAPGSLPYSKAPIR	37.2								
									RKPAPAAAAKPNALPSNITGVTEK	19.4								
									SLRVEVPKPR	18.1								
									VVTTVTENGTELPK	39.7								
179	Tb927.11.12150	flagellar protein essential for flagellar pocket biogenesis	1.00	172	5	4	4	9.7	AFSLAGEASAADDKVR	48.3	0.99	69	3	2	2	7.2	HGFTVFNLNFSPATVDDLFEKGDSNRDGR	31.9
									AHNAADQAARDADIVKLR	28.9							LKEIELADQLKQR	30.1
									ALEKALDEAR	27.8								
									LKEIELADQLKQR	63.7								
180	Tb927.10.3260	Long-chain-fatty-acid--CoA ligase 5 (EC 6.2.1.3) (Long-chain acyl-CoA synthetase 5) (LACS 5), putative	1.00	149	11	4	4	10.2	LLSDEWTPENGVLTAAAK Deamidated (N10)	49.1	1.00	119	7	3	3	8.1	LLSDEWTPENGVLTAAAK Deamidated (N10)	40.8
									TITNTFSKPHGDLVEYRPFVLSAVPR	38.0							TITNTFSKPHGDLVEYRPFVLSAVPR	36.4
									VVAGTEREDASPVYR	24.7							YKELIEEIFRNDV	12.1
									YKELIEEIFRNDV	9.6								
181	Tb927.3.2180	hypothetical protein, conserved	1.00	133	3	3	2	35.3	SFSGLSGPDGHWIMLPK Oxidation (M14)	8.3	0.00	0	0	0	0	0.0		
									SSSPFQEPNTAIYDSYLAWTYFQPMDVHIEKLPAPEAK Deamidated (N9)- Oxidation (M25)	44.6								
									SSSPFQEPNTAIYDSYLAWTYFQPMDVHIEKLPAPEAK Oxidation (M25)	80.3								
182	Tb927.10.560	40S ribosomal proteins S11, putative	1.00	235	17	5	5	21.8	IGLGFATPK	61.5	0.49	25	2	1	1	6.9	TPTVDLTVQHEK	22.1
									KIGLGFATPK	22.3								
									TIRYNVLQVVSK	34.3								
									TPTVDLTVQHEK	28.9								
									TPTVDLTVQHEKAYQR	28.3								
183	Tb927.7.4770	cyclophilin-type peptidyl-prolyl cis-trans isomerase, putative	1.00	345	15	6	4	36.4	IVIGLFGKETPR	64.5	0.00	0	0	0	0	0.0		
									KVEGVKTGTGDKPTSPVR	32.6								
									TVENFKQLATGVNGFGYKDSTFHR	54.1								
									TVENFKQLATGVNGFGYKDSTFHR Deamidated (N13)	52.2								
									TVENFKQLATGVNGFGYKDSTFHR Deamidated (Q7)	70.9								
									VLEGMDVVR Oxidation (M5)	25.7								
184	Tb927.11.550	hypothetical protein SCD6.10	0.00	18	1	1	1	10.4	GSDIEELTVFEESHNAMMDPAVVTALPAR Oxidation (M17)- Oxidation (M18)	17.9	1.00	56	1	1	1	6.4	GQVEVPAAEQLFDQIVFR Deamidated (Q2)	56.2
185	Tb927.8.890	small GTP-binding protein Rab1, putative	1.00	182	6	4	1	15.4	VAAQGANSGATAGGRPLLTGNNRPATNSGGQK	35.6	0.00	0	0	0	0	0.0		
									VAAQGANSGATAGGRPLLTGNNRPATNSGGQK Deamidated (Q4)	49.8								
									VAAQGANSGATAGGRPLLTGNNRPATNSGGQK Deamidated (Q4)- Deamidated (N21)	32.2								
									VAAQGANSGATAGGRPLLTGNNRPATNSGGQK Deamidated (Q4)- Deamidated (N22)	36.3								
186	Tb927.7.2570	mRNA processing protein, putative	1.00	200	17	3	3	10.2	AFQLLVSDTAPDVVVSR	69.8	1.00	216	15	4	4	10.2	AFQLLVSDTAPDVVVSR	48.5
									AVGLTAFELAGR	48.6							AVGLTAFELAGR	71.3
									LAGLVAEDFVREYGCEAKL Carbamidomethyl (C15)	39.2							LAGLVAEDFVREYGCEAK Carbamidomethyl (C15)	36.8
																	LAGLVAEDFVREYGCEAKL Carbamidomethyl (C15)	26.5
187	Tb927.10.15180	nucleosome assembly protein, putative	1.00	61	4	1	1	3.3	LVQGDDGATVTR	33.1	0.00	24	2	2	2	4.6	TPITWKPGKDVTVR	8.6
																	TPITWKPGKDVTVRTEK	15.9
188	Tb927.11.1430	hypothetical protein, conserved	1.00	153	9	5	5	13.2	DFYSQSGLELDPALPLPFDAYTELQNRPK	11.1	1.00	188	14	4	4	11.2	DFYSQSGLELDPALPLPFDAYTELQNRPK	35.2
									FGITSYDPIPGGKNPPAAPIGCHRR Carbamidomethyl (C22)	36.0							FVLTVYPADDTISIFEPVIR	25.6
									HIVPLPDGSPITFANFR	16.9							HIVPLPDGSPITFANFR	28.3
									TIAASDPTNLTLLPEQVR	42.1							TIAASDPTNLTLLPEQVR	24.2
									VFNGTSTLPR Deamidated (N3)	38.6								
189	Tb927.9.9550	hypothetical protein, conserved	1.00	221	19	6	6	38.4	IDKESGFQPVVNQR	41.1	1.00	104	3	2	2	14.7	LLEEVQKDTSR	44.7
									IDKESGFQPVVNQRK	21.8							TPTSLSTAAVEKQAK	50.9
									LLEEVQKDTSRRPK	20.3								
									QQQQHQQQQTSAPKPMLVNEALER Oxidation (M16)	17.0								
									TPTSLSTAAVEK	38.0								
									TPTSLSTAAVEKQAK	27.7								
190	Tb927.4.5010	calreticulin, putative	0.97	265	23	5	4	11.4	FSSIDHWTASK	72.3	0.91	211	16	4	4	13.7	FSSIDHWTASK	67.1
	Tb927.8.7410	calreticulin, putative							FYADAEKSKGLR	47.1							FYALSTPLPTPITNEKK	29.6
									KIPNPAYKGPWAPR	49.2							KAEEDALAAFEK	22.7
									KIPNPAYKGPWAPR Deamidated (N4)	36.2							KIPNPAYKGPWAPR	40.5
									SKGPWAPR	27.5								
191	Tb927.7.3740	hypothetical protein, conserved	0.99	71	5	2	2	3.7	LVSVEGNVLNLDDRKADYSGLQR	19.4	0.00	11	1	1	1	1.8	TVGPGDGLDAVQSAR	10.6
									RLNQLQNR	34.7								
192	Tb927.10.9570	paraflagellar rod component, putative	1.00	248	11	7	6	9.2	AIRLEEVNDETQFR	36.6	1.00	171	11	5	4	4.9	GSIDLKKILLK	29.3
									GSIDLKKILLK	25.2							LADKTINLNSK	8.0
									LLTFFILDTHALR	27.1							LLTFFILDTHALR	56.8
									STDYAISQEQKVGIDAFKAYESSVR	28.8							TVQTIVSDIENRPASVTK	38.0
									STDYAISQEQKVGIDAFKAYESSVR Deamidated (Q10)	29.5							TVQTIVSDIENRPASVTK Deamidated (N11)	2.0
									STIAHECELNKEPSCLKR Carbamidomethyl (C7)- Carbamidomethyl (C15)	19.6								
									TVQTIVSDIENRPASVTK	50.5								
193	Tb927.8.1570	hypothetical protein, conserved	0.92	58	6	2	2	8.8	IVSTPSGDSVDVR	33.7	1.00	103	5	3	3	27.2	AAAVEAFENSMDALKQFQLVDFEDHLESVQLNYLDQTVSA	38.8
									KSGVHEPLLWR	19.2							GAYYNSGSSSFTDAPSLTFGR	32.7
																	IVSTPSGDSVDVR	22.9
194	Tb927.10.6180	hypothetical protein, conserved	0.80	32	3	1	1	3.6	SGATEGEGPSPFLGYFVR	25.8	1.00	349	30	5	5	17.4	EAEDVELLQHEINSGYGTNN	37.7
																	FGPGEHLYIASAR	42.1
																	FIGNVLNKESFPVRVEK	27.6
																	KVGLFVSPWSNEYALLSVR	58.1
																	SGATEGEGPSPFLGYFVR	34.8
195	Tb927.10.1390	hypoxanthine-guanine phosphoribosyltransferase, putative	1.00	118	5	2	2	13.3	SMKEMAQTLANAYK Oxidation (M2)- Oxidation (M5)	38.1	0.99	44	2	1	1	7.3	SYPMAESVVATESTIHR Oxidation (M4)	29.8
									SYPMAESVVATESTIHR Oxidation (M4)	64.5								
196	Tb927.11.12220	vacuolar transporter chaperone 4	1.00	138	10	5	5	10.1	IHAYDLKPVLR	32.3	1.00	329	27	8	8	15.5	AAFQCGLDQSIR Carbamidomethyl (C5)	21.1
									NLNTDTIEHIIAR	17.9							GDLSLKPALEK	23.7
									SAVYPDFREQGAYINYK	27.0							GVPFTLGSPISSVYYDNDKFLLYHR	18.1
									TTDRVIFGTGHAQTQTVGASEAR	19.1							HVAAEVFSGLIPWQTGKR	41.4
									YAHGIATLYGHTPFIK	20.3							IHAYDLKPVLR	38.6
																	NLNTDTIEHIIAR	41.0
																	SAVYPDFREQGAYINYK	26.5
																	YAHGIATLYGHTPFIK	24.9
197	Tb927.8.6660	paraflagellar rod component, putative	1.00	174	16	5	5	16.1	ITLDKSQISK	43.3	1.00	191	22	4	3	11.2	SLVLNAPFPVLQEQLIER	38.9

								SIDVSNNPLVTVCSASPIIR Carbamidomethyl (C13)	28.0							SLVLNAPFPVLQEQLIER Deamidated (Q14)	39.7
								SLVLNAPFPVLQEQLIER	42.4							TTIPVPLFLSAEFTTDLETLKPR	29.6
								TTIPVPLFLSAEFTTDLETLKPR	8.5							VSFSGCHVTDEFVSELAEILQDHQSIR Carbamidomethyl (C6)	35.5
								VSFSGCHVTDEFVSELAEILQDHQSIR Carbamidomethyl (C6)	23.8								
198	Tb927.8.4780	hypothetical protein, conserved	1.00	199	17	6	6	2.4	ACNPFLLYEDRK Carbamidomethyl (C2)	24.3	0.00	8	1	1	1	0.6	ALVTQLAEENATKTDAVHKQYPFLPK	8.4
								AIVEERKAEEESLAAAYPFLGR	17.0								
								ALVTQLAEENATKTDAVHKQYPFLPK	20.3								
								KAEEESLAAAYPFLGR	47.3								
								RVMGIPLGELPINDDAMFSQLGQR Oxidation (M3)- Oxidation (M17)	29.7								
								TSSDGESFAGGLPGAR	21.4								
199	Tb927.3.3460	hypothetical protein, conserved	1.00	233	10	5	4	14.9	GNPVGAIAEIDKILPEISDDALKR	65.3	0.36	33	6	2	2	8.5	GNPVGAIAEIDKILPEISDDALKR	7.3
								GNPVGAIAEIDKILPEISDDALKR Deamidated (N2)	63.2							TSVPHQPFYAGEDELRR	20.6
								IVDEAIKGIVEGYSTK	39.9								
								TGAKGLAGNASVLGK	26.8								
								TSVPHQPFYAGEDELRR	28.0								
200	Tb927.11.9080	hypothetical protein, conserved	1.00	199	9	4	3	3.5	LKLIGTPQK	22.9	0.00	7	1	1	1	1.6	STALQEQIAGATTVAPEPR	6.5
								STALQEQIAGATTVAPEPR	46.0								
								STALQEQIAGATTVAPEPR Deamidated (Q7)	2.3								
								VKPIDAAATALEVVR	75.3								
201	Tb927.11.14130	ribosomal protein L18, putative	0.96	294	17	8	6	39.7	AGAVSQAYNDLASR	81.2	0.00	0	0	0	0	0.0		
	Tb927.10.3840	60S ribosomal protein L18a, putative							ARYHNIEVLGVK	19.6								
								ETPSEKNPQPTVYKFEVFAPNFVVAK	29.4								
								ETPSEKNPQPTVYKFEVFAPNFVVAK Deamidated (N7)	29.9								
								LNVAQFHPHNLSFPLLQR	29.2								
								LNVAQFHPHNLSFPLLQR Deamidated (N10)	17.3								
								LNVAQFHPHNLSFPLLQRR	17.9								
								YHNIEVLGVK	41.6								
202	Tb927.10.4880	hypothetical protein, conserved	1.00	151	13	3	3	11.2	KSPFMSKLMDQALPVYDDVVTGSGR Oxidation (M5)- Oxidation (M9)	33.4	0.99	56	2	2	1	8.0	LMDQALPVYDDVVTGSGR Oxidation (M2)	44.2
								LMDQALPVYDDVVTGSGR Oxidation (M2)	77.6							LMDQALPVYDDVVTGSGR Oxidation (M2)- Deamidated (Q4)	11.6
								SPFMSKLMDQALPVYDDVVTGSGR Oxidation (M4)- Oxidation (M8)	25.6								
203	Tb927.9.11220	hypothetical protein, conserved	1.00	195	13	6	5	17.1	AVNQFYESEELAK	23.3	1.00	132	10	3	3	14.0	LAAFQPDIIEFFLANK	49.6
								AVNQFYESEELAKR	40.7							SVPAFSFLAIHVAK	34.2
								AVNQFYESEELAKR Deamidated (Q4)	38.8							VVDTTSIQEVLVFLQTVAPR	18.9
								SPFEVDWTAGEAR	6.7								
								SVPAFSFLAIHVAK	40.6								
								VVDTTSIQEVLVFLQTVAPR	7.4								
204	Tb927.8.3870	hypothetical protein, conserved	1.00	181	15	4	4	4.4	LYNNQETSVHTDEENEPLYK	10.9	1.00	91	2	2	2	1.6	PVTVEEVVLR	71.4
								PVTVEEVVLR	72.0							VEAVDSYRR	20.0
								SLSHYEGESEEILR	43.1								
								VEAVDSYRR	22.2								
205	Tb927.2.5160	chaperone protein DNAj, putative	1.00	148	8	3	3	15.5	ILGYPKQEEPAPEATEHTLAVTYVDLDREAR	38.3	0.00	0	0	0	0	0.0		
								SIGPGFVQQMQVACPR Oxidation (M10)- Carbamidomethyl (C14)	61.6								
								STNVIDPQKLWSVSR	20.3								
206	Tb927.10.240	peroxin 14, putative	1.00	177	14	7	7	23.2	AELSITLGKLR	16.8	1.00	136	8	4	3	12.8	EVPALPVPAIPVASESHVDAK	24.1
								EVPALPVPAIPVASESHVDAK	14.6							HTSLLYAPQAPPLPEAAAATR	34.1
								HTSLLYAPQAPPLPEAAAATR	34.0							HTSLLYAPQAPPLPEAAAATR Deamidated (Q9)	23.1
								LRGQVTAYSR	19.5							SLVHREVPALPVPAIPVASESHVDAK	39.8
								RHTSLLYAPQAPPLPEAAAATR	25.9								
								SLVHREVPALPVPAIPVASESHVDAK	6.6								
								TNEKQESQIKSLQEEVNR	30.8								
207	Tb927.10.8230	protein disulfide isomerase,bloodstream- specific protein 2 precursor	1.00	337	20	7	7	18.7	ANVGPAVTPASNAEEVTR	50.0	1.00	73	5	2	2	6.0	SLPVPEVETVDGKTTIVAK	24.2
								GYPTIILFRNGKEAEHYGGAR Deamidated (N10)	35.2							VSLKFFEAEPK	33.3
								LFPDEKPETIVVYR	28.8								
								SLPVPEVETVDGKTTIVAK	27.8								
								TKDDIIKYIK	55.1								
								VSLKFFEAEPK	43.6								
								VSLKFFEAEPKLFPDEKPETIVVYR	37.7								
208	Tb927.6.4210	aldehyde dehydrogenase, putative	1.00	254	15	5	4	15.6	AKHIADQLVTGMTNVNDFGINYLCQSLPFGGVK Oxidation (M12)- Carbamidomethyl (C24)	50.8	1.00	215	9	5	4	11.4	ASTTDRIPGVK	35.4
								ASTTDRIPGVK	31.4							FDNQPSTQPLTPR	32.2
								FDNQPSTQPLTPR	38.1							LLTILEKR	43.7
								SAAQTLTPVVLELGGKDPAIVCDDADLEHVVPIIMR Carbamidomethyl (C22)	54.0							SAAQTLTPVVLELGGKDPAIVCDDADLEHVVPIIMR Carbamidomethyl (C22)	58.3
								SAAQTLTPVVLELGGKDPAIVCDDADLEHVVPIIMR Carbamidomethyl (C22)- Oxidation (M35)	46.0							SAAQTLTPVVLELGGKDPAIVCDDADLEHVVPIIMR Carbamidomethyl (C22)- Oxidation (M35)	16.6
209	Tb927.6.3150	Hydin,flagellar component	1.00	218	19	8	8	3.2	ALLSVTYSPFLYTKEKDIGNLFIPLPSGEGR	8.7	0.00	0	0	0	0	0.0		
								ALQNVITLSPVSGTVR	36.0								
								GAVPVVASPLVEASR	24.5								
								GRLDLPESFAVSPSPVK	8.4								
								KAAVAKPTAHEVAPPPPADTIQMPLTFVGSSVTR Oxidation (M23)	24.9								
								KVVGAVAEPAYR	30.9								
								VIVVVGPPCSGK Carbamidomethyl (C9)	26.7								
								VIVVVGPPCSGKTTQTQK Carbamidomethyl (C9)	20.2								
210	Tb927.10.7090	alternative oxidase	0.75	40	2	2	2	7.3	RLQNSVNPFVVLK	30.2	1.00	262	17	8	5	15.5	LQNSVNPFVVLK	8.4
								TRTDFGSEGAK	9.7							LRPTKNDVPEVAR	32.5
																LSFLETVPVVPLR	25.9
																NATFRDLINVIR	28.2
																NATFRDLINVIR Deamidated (N1)	33.0
																NATFRDLINVIR Deamidated (N9)	33.1
																RLQNSVNPFVVLK	32.5
																RLQNSVNPFVVLK Deamidated (N4)	32.0
211	Tb927.11.1710	mitochondrial RNA binding protein 1,gBP21, MRP1	1.00	210	14	5	5	30.6	AAEGGEKIAATSSAEGGAR	47.3	0.00	0	0	0	0	0.0		
								KVDPNDLSPQFDADRR	21.5								
								LLLISQYPQLGPR	71.7								
								SVSDTSRYH	27.1								
								VAVDGKLLLISQYPQLGPR Deamidated (Q15)	1.1								
212	Tb927.3.1810	hypothetical protein, conserved	1.00	63	5	2	2	15.6	LNTVLQYIPNEWSPFTELGIPEEVRVR	37.5	1.00	178	5	4	2	17.5	LNTVLQYIPNEWSPFTELGIPEEVRVR	35.0
								SSSSPSSPEAPASSPLKPDDINAR	10.8							LNTVLQYIPNEWSPFTELGIPEEVRVR Deamidated (Q6)- Deamidated (N10)	34.2
																STEQEEAVNNLLEEVQALLSRPVPIGSLFR	44.0
																STEQEEAVNNLLEEVQALLSRPVPIGSLFR Deamidated (N10)	49.5
213	Tb927.9.9450	hypothetical protein, conserved,zinc finger protein family member, putative	1.00	190	33	4	3	4.6	AAKPALQVPQPR	41.0	0.77	64	5	3	3	4.3	AAKPALQVPQPR	27.5
								AKPNEAASPYPQQQTRPTSAR	23.8							AKPNEAASPYPQQQTRPTSAR	17.2
								AKPNEAASPYPQQQTRPTSAR Deamidated (N4)	19.4							RGEVQPTVEAK	12.8
								RGEVQPTVEAKLPR	45.7								
214	Tb927.11.8160	dynein heavy chain, putative	1.00	120	10	5	4	1.5	AVNTVISAAGLNKR	35.8	0.00	14	1	1	1	0.4	TWPLLIDPQGQANSWIR	13.8
								AVNTVISAAGLNKR Deamidated (N3)	20.0								
								AVNTVISAAGLNKRENPNEDEDLLLLR	9.2								
								LIAGLGGEKVR	18.9								
								TIKSDPSSVIPIGDFDFAERER	14.6								
215	Tb927.4.3590	translation elongation factor 1-beta, putative	1.00	124	8	4	4	31.4	DGLLWGDHKLVPIAFGVK	6.0	0.75	121	7	3	3	28.0	DGLLWGDHKLVPIAFGVK	28.7
								ITPPVAAPVAAPAAAPAAAPAATPAK	22.4							ITPPVAAPVAAPAAAPAAAPAATPAK	17.5

									LFLGGVKPSEEDVKAFNDLLGGDNTNVFR	40.5							LFLGGVKPSEEDVKAFNDLLGGDNTNVFR	55.1
									TAWGAPVKITPPVAAPVAAPAAAPAAAPAATPAKK	43.5								
216	Tb927.9.2450	electon transport protein SCO1/SCO2, putative	1.00	149	4	3	2	11.5	ILGLVGTQEEVESVAR	64.8	1.00	88	4	3	3	16.4	EAGASDDNGQAAINIK	5.1
									ILGLVGTQEEVESVAR Deamidated (Q8)	19.4							ILGLVGTQEEVESVAR	42.9
									VANVATMLDENVASVQKPSEI Oxidation (M7)	58.6							VANVATMLDENVASVQKPSEI Deamidated (Q16)	35.7
217	Tb927.3.2440	serine/threonine-protein kinase, putative,protein kinase, putative	1.00	168	6	4	2	9.4	LLVKDAAHR	32.4	0.00	0	0	0	0	0.0		
									SAAQSGNNSSKNPAQVVNTPAHSSQLNAR	36.1								
									SAAQSGNNSSKNPAQVVNTPAHSSQLNAR Deamidated (N8)	47.5								
									SAAQSGNNSSKNPAQVVNTPAHSSQLNAR Deamidated (Q15)	46.5								
218	Tb927.9.4190	fatty acyl CoA syntetase 1	1.00	131	7	4	4	9.6	GDLKEFKPVFIVGVPR	37.8	1.00	165	13	4	4	9.0	GDLKEFKPVFIVGVPR	35.5
									LGELYPSVGQLLKGVEGR	42.9							LGELYPSVGQLLK	31.5
									QKFGPLAHICGPAEGENCSPVYR Carbamidomethyl (C10)- Carbamidomethyl (C18)	28.3							TLTDDTARPR	19.5
									TLTDDTARPR	15.3							VVFGLPLGQGYGLTETCCNGSIQR Deamidated (Q9)- Carbamidomethyl (C17)- Carbamidomethyl (C18)	49.3
219	Tb927.4.4070	mevalonate kinase, putative	1.00	167	7	4	4	17.3	ALNELYSLNLSEEAVNR	66.1	1.00	203	9	4	3	16.4	ALNELYSLNLSEEAVNR	50.9
									HLNIDTSKDGLLVK	45.8							ALNELYSLNLSEEAVNR Deamidated (N9)	43.1
									LTVSCPELDAIATCCR Carbamidomethyl (C5)- Carbamidomethyl (C14)- Carbamidomethyl (C15)	11.0							HLNIDTSKDGLLVK	37.7
									YSVQPGGSKL	31.2							LGGPLVPSSGIGASASDVVSLSR	33.6
220	Tb927.10.2530	adenylate kinase, putative	1.00	223	14	6	5	32.0	GLDDVTSEPLIQRPDDRKEVVEK	23.2	0.00	0	0	0	0	0.0		
									MKVIFLGPPGCGKGTQSPFVAK Oxidation (M1)- Carbamidomethyl (C11)	20.9								
									SVMDVGGLVSDDIVFGIVKESIKQPR Oxidation (M3)	45.1								
									SVMDVGGLVSDDIVFGIVKESIKQPR Oxidation (M3)- Deamidated (Q24)	50.3								
									VIFLGPPGCGK Carbamidomethyl (C9)	20.2								
									VIFLGPPGCGKGTQSPFVAK Carbamidomethyl (C9)	21.6								
221	Tb927.5.500	hypothetical protein, conserved	1.00	239	12	5	5	7.1	ASLAECNAIVESTREPSGPVAGPDEAEAAKR Carbamidomethyl (C6)	67.0	0.32	42	2	2	2	2.2	NIVTSALDDEFRETNVGK	18.6
									NIVTSALDDEFRETNVGK	15.1							RVPAVVSVFDTLR	23.6
									RVPAVVSVFDTLR	28.5								
									VGLGTATPTDAEEGGEEKVPGPATLSPAPYEATR	28.5								
									VGLGTATPTDAEEGGEEKVPGPATLSPAPYEATRAER	54.9								
222	Tb927.2.3800	mRNA processing protein, putative	1.00	171	4	3	2	11.6	AFQLLHSETATDVGIASIDPSKLPGQSK	62.8	1.00	61	3	1	1	5.7	AFQLLHSETATDVGIASIDPSKLPGQSK	46.2
									NTVWGLWNEGNLFSLSVPELAFFLQEHCR Carbamidomethyl (C28)	59.3								
									NTVWGLWNEGNLFSLSVPELAFFLQEHCR Deamidated (Q25)- Carbamidomethyl (C28)	43.3								
223	Tb927.7.2170	hypothetical protein, conserved	1.00	177	12	5	5	2.6	ALFQQLVAR	49.7	0.00	0	0	0	0	0.0		
									ALLQMCLR Oxidation (M5)- Carbamidomethyl (C6)	28.9								
									LVDGVDCR Carbamidomethyl (C7)	15.5								
									NCIVEALESSSTR Carbamidomethyl (C2)	14.0								
									RALFQQLVAR	41.7								
224	Tb927.8.1990	peroxidoxin	1.00	188	12	4	4	16.4	ALFVIDDKGILR	57.9	0.25	22	1	1	1	5.3	ALFVIDDKGILR	21.5
									ALFVIDDKGILRHVTINDLPVGR	30.5								
									HVTINDLPVGR	39.6								
									TATVREAAPEFAGK	34.1								
225	Tb927.10.12840	mitochondrial carrier protein, putative,mitochondrial 2-oxoglutarate/malate carrier protein, putative	1.00	153	13	4	4	16.5	AALVGVAQVAVHDQVLSALR	26.5	1.00	233	23	4	3	16.5	AALVGVAQVAVHDQVLSALR	50.1
									GPFRGLSTGILR	28.2							AALVGVAQVAVHDQVLSALR Deamidated (Q8)	36.1
									LGCYPIFR Carbamidomethyl (C3)	30.2							VVREEGALAVYDSFAPYFFR	26.9
									YKSVTQTIGR	29.9							YKSVTQTIGR	23.6
226	Tb927.7.2390	hypothetical protein, conserved	1.00	114	3	3	2	4.1	AALVTAFDSISAPLTSNGSNSIPR	49.3	0.00	5	1	1	1	1.6	LPPVSAALKTHPFMK Oxidation (M14)	4.7
									AALVTAFDSISAPLTSNGSNSIPR Deamidated (N17)	46.5								
									LPPVSAALKTHPFMK Oxidation (M14)	17.7								
227	Tb927.4.1330	DNA topoisomerase IB, large subunit	1.00	262	30	5	5	7.9	AVATYFIDKLALR	59.6	1.00	101	6	3	3	4.2	AVATYFIDKLALR	51.2
									ILPEDVVLNIGK	28.3							VIPTALTGSASSTK	23.4
									ILPEDVVLNIGKEAPIPQAPAGHKWK	24.0							VIPTALTGSASSTKNK	17.1
									VIPTALTGSASSTK	48.1								
									VIPTALTGSASSTKNK	39.9								
228	Tb927.11.12230	heat shock protein HslVU, ATPase subunit HslU, putative,ATP-dependent hsl protease ATP-binding subunit hslU, putative	1.00	99	2	2	2	5.4	LVDETQISALAR	61.3	0.77	32	1	1	1	3.6	KIASILDSYIVGQAEGKR	32.0
									VTTKVNANAQNIGAR	38.0								
229	Tb927.8.3690	isocitrate dehydrogenase [NADP], mitochondrial precursor, putative	1.00	197	10	4	4	11.2	DASNDKVTEQAAEAIQR	38.7	0.00	10	1	1	1	3.0	VVPQWSEPIIVGR	10.1
									NILGGTVFREPIIVK	49.0								
									NILGGTVFREPIIVKNVPR	50.2								
									VVPQWSEPIIVGR	29.1								
230	Tb927.11.15040	chaperonin HSP60, mitochondrial precursor, putative,heat shock protein 60	1.00	229	22	5	5	10.8	HIEFENSFENLGANLVR	32.5	1.00	145	7	4	4	8.8	HIEFENSFENLGANLVR	26.7
									ITKDGVTVAK	27.8							ITKDGVTVAK	30.3
									KLLLLGVENLVK	56.3							KLLLLGVENLVK	67.0
									NVVLEMPYASPK Oxidation (M6)	34.4							SVSTGTNPMDLKR Oxidation (M9)	12.8
									SVSTGTNPMDLKR Oxidation (M9)	28.3								
231	Tb927.6.3050	aldehyde dehydrogenase family, putative	1.00	118	7	4	4	10.7	EKPLALYVFSNNKR	18.1	1.00	52	3	1	1	2.8	SLLNLVEENTDEFCK Carbamidomethyl (C14)	46.7
									SLLNLVEENTDEFCK Carbamidomethyl (C14)	47.0								
									VAEIVMAAAAK Oxidation (M6)	34.8								
									VLNSLLKPSAEVAGAVGR Deamidated (N3)	12.2								
232	Tb927.8.5120	cytochrome c	0.99	183	8	6	5	53.5	AAQCHTGTKGGSNGVGPNLYGIVGR Deamidated (Q3)- Carbamidomethyl (C4)	35.9	0.00	0	0	0	0	0.0		
									AAQCHTGTKGGSNGVGPNLYGIVGRK Carbamidomethyl (C4)- Deamidated (N13)	34.2								
									AAQCHTGTKGGSNGVGPNLYGIVGRK Deamidated (Q3)- Carbamidomethyl (C4)	33.3								
									ADLIAYLETLKD	22.5								
									MSFAGLKKPQER Oxidation (M1)	20.6								
									SGTVEGFTYSK	27.2								
233	Tb927.6.1500	alkyl-dihydroxyacetone phosphate synthase	1.00	145	12	5	5	12.9	HNVVVVPFGGGTNVTGGVEPNPFETRR	23.7	0.86	53	3	2	2	6.7	HNVVVVPFGGGTNVTGGVEPNPFETRR	31.9
									IKPTPGLTKEEAIK	20.5							IKPTPGLTKEEAIK	20.3
									LLPSPPSEKETPK	21.1								
									LLPSPPSEKETPKATQAR	20.8								
									QNREMMFDKMGIPGALQAHL Oxidation (M5)- Oxidation (M6)- Oxidation (M10)	26.6								
234	Tb927.2.5660	adenylate kinase, putative	1.00	113	5	3	2	18.1	GIPLQIFIAGPAGSGKR	49.1	0.00	12	1	1	1	11.5	NAQQAQAVEECGVIPQVFILLDLPEDLSFR Carbamidomethyl (C11)	11.7
									GIPLQIFIAGPAGSGKR Deamidated (Q5)	39.1								
									NAQQAQAVEECGVIPQVFILLDLPEDLSFR Carbamidomethyl (C11)	20.2								
235	Tb927.10.5770	valosin-containing protein homolog,Transitional endoplasmic reticulum ATPase, putative	1.00	184	9	4	4	9.7	AAAPCVLFFDELDSVAR Carbamidomethyl (C5)	42.5	1.00	53	4	1	1	2.2	AAAPCVLFFDELDSVAR Carbamidomethyl (C5)	40.2
									IVSQLLTLMDGLKGR	34.4								
									KSPLAPDVDLDQLAAATHGFSGADLAGICQR Carbamidomethyl (C29)	57.1								
									KYELFATSLQQSR	37.1								
236	Tb927.11.5290	mitochondrial carrier protein, putative	1.00	146	9	3	3	14.2	AFPANAACFAAR Carbamidomethyl (C8)	59.8	0.88	39	2	2	2	9.6	VQASPVPLTPVVAFR	33.5
									DKTKSTLNNLCGM Carbamidomethyl (C11)- Oxidation (M13)	6.2							VRQQTALSSGAR	5.7
									VQASPVPLTPVVAFR	54.4								
237	Tb927.9.5690	60S acidic ribosomal protein, putative	1.00	114	6	3	3	58.4	MSAVSVAAAPAAGGAAAPAAGGAAAGGAAAPAK Oxidation (M1)	27.0	0.00	13	2	1	1	29.2	MSAVSVAAAPAAGGAAAPAAGGAAAGGAAAPAK Oxidation (M1)	10.9
									SVNTLIAEGVAK Deamidated (N3)	31.2								
									YLAAYALASLAKPAPTADDVK	16.9								
238	Tb927.11.6140	40S ribosomal protein S15A, putative	1.00	124	6	2	2	16.9	NTGGKVLGFFY	53.9	0.00	0	0	0	0	0.0		
									TMMSVLANALR Oxidation (M2)- Oxidation (M3)	52.3								

239	Tb927.11.600	hypothetical protein, conserved	1.00	173	10	4	3	41.5	ATTPGQLEGVPAELQQLAEAFTK<br>QQALDSIKLMQQGVR Deamidated (Q2)- Oxidation (M10)<br>QQALDSIKLMQQGVR Oxidation (M10)<br>QQNVERQQALDSIKLMQQGVR Oxidation (M16)	25.7<br>44.2<br>28.9<br>45.0	0.00	4	1	1	1	21.7	ATTPGQLEGVPAELQQLAEAFTK	4.3
240	Tb927.2.5800	sedoheptulose-1,7-bisphosphatase	1.00	240	19	5	5	20.8	AGVPCDVVGIVETVAGACR Carbamidomethyl (C5)- Carbamidomethyl (C18)<br>AIAAGLRNDGVTAAK<br>KAGVPCDVVGIVETVAGACR Carbamidomethyl (C6)- Carbamidomethyl (C19)<br>LLFEAAPMAFLIHCAGGR Oxidation (M8)- Carbamidomethyl (C14)<br>VVSMEQTTPIALGCAR Oxidation (M4)- Carbamidomethyl (C14)	46.3<br>45.7<br>36.7<br>26.4<br>37.4	1.00	165	7	4	4	19.3	AGVPCDVVGIVETVAGACR Carbamidomethyl (C5)- Carbamidomethyl (C18)<br>AIAAGLRNDGVTAAK<br>QNLTSDSLITDTLR<br>VVSMEQTTPIALGCAR Oxidation (M4)- Carbamidomethyl (C14)	54.3<br>33.7<br>43.5<br>13.3
241	Tb927.4.2080	hypothetical protein, conserved	1.00	203	11	4	4	5.5	DKKLQELNDQLFALR<br>IQSLEVLNGNISR Deamidated (N8)<br>LLLQNESLQQR<br>VAIVAQNPGTR	12.9<br>60.8<br>38.3<br>58.6	1.00	197	8	5	5	8.6	DKKLQELNDQLFALR<br>GSNQHLQQALLKEDIAELEEKLQLAEER<br>IQSLEVLNGNISR Deamidated (N8)<br>LLLQNESLQQR Deamidated (Q4)<br>VAIVAQNPGTR	32.4<br>29.7<br>36.5<br>35.3<br>45.5
242	Tb927.10.8780	hypothetical protein, conserved	1.00	114	6	3	2	2.1	QNTLVPVLVHALDDNPLSGFDQLFSEKVVPR Deamidated (N2)<br>QNTLVPVLVHALDDNPLSGFDQLFSEKVVPR Deamidated (Q1)- Deamidated (N2)<br>VAGCVTHIEPFASK Carbamidomethyl (C4)	35.0<br>41.5<br>17.0	0.00	0	0	0	0	0.0		
243	Tb927.10.15750	hypothetical protein, conserved	1.00	184	13	4	3	2.4	LFEQLMDLARDEQQHR<br>LFEQLMDLARDEQQHR Oxidation (M6)<br>VALESTAVETTSR<br>VEVATWDSEQQLR	23.5<br>38.7<br>69.1<br>25.5	0.23	22	1	1	1	0.6	QNTEEEKKEAK Deamidated (N2)	21.8
244	Tb927.9.4500	heat shock protein, putative,HSP70-like protein	1.00	91	5	2	2	3.7	AQFEEACGELFNEAVR Carbamidomethyl (C7)<br>SGLLNDVESLLLHYK	63.8<br>11.2	0.99	56	3	2	2	4.1	FPQQSICGPSLLAGLIVSK Carbamidomethyl (C7)<br>SGLLNDVESLLLHYK	44.2<br>8.2
245	Tb927.9.4680	ATP-dependent DEAD box helicase, putative	1.00	102	11	3	3	10.9	GGDIIAQAQSGTGK<br>GIYSHGFEKPSSIQQR<br>LQSGTIVAVGTPGR	34.3<br>41.4<br>9.3	0.99	80	8	3	2	7.4	GIYSHGFEKPSSIQQR<br>GIYSHGFEKPSSIQQR Deamidated (Q15)<br>LQSGTIVAVGTPGR	30.2<br>34.8<br>6.1
246	Tb927.4.870	dynein heavy chain, putative	1.00	153	13	4	4	2.1	FVAEDEPLFKGLIGDLFPGLDPTRAPQENLSR<br>LLPTSSLLFEVGDLQYASPATVSR<br>QLAACQDEIQDLTAGLR Carbamidomethyl (C5)<br>SITELKSFAKPPASVVAVVR	25.5<br>40.1<br>47.1<br>18.4	0.97	42	2	1	1	0.5	LLPTSSLLFEVGDLQYASPATVSR	35.5
247	Tb927.10.5760	adenylate kinase, putative	1.00	199	14	5	5	27.4	GILLDGFPR<br>GLHILLFGAPGCGK Carbamidomethyl (C12)<br>GLHILLFGAPGCGKGTASEFLVR Carbamidomethyl (C12)<br>IYNLKSDPSPQEIMGR Oxidation (M14)<br>SVATKVDELLR	7.2<br>56.4<br>20.9<br>29.5<br>40.8	0.00	10	1	1	1	7.4	IYNLKSDPSPQEIMGR Oxidation (M14)	9.9
248	Tb927.10.13180	hypothetical protein, conserved	1.00	127	6	4	4	4.4	GAICAQLSK Carbamidomethyl (C4)<br>IYISELVEREPAAHVVR<br>SISVITITSHAPR<br>VHNVMDKDAIVAAAR Oxidation (M5)	17.5<br>27.5<br>64.6<br>15.9	0.00	21	1	1	1	1.4	IYISELVEREPAAHVVR	21.2
249	Tb927.11.14780	phosphomannose isomerase, putative	1.00	128	10	4	4	16.6	LIKLDCGVQHYAWGKEAAESYVAK Carbamidomethyl (C6)<br>WKDVPTLLR<br>YGTDGLER<br>YRAPEGSEWELQHYSPPREFQDFSLYR	33.4<br>30.7<br>20.2<br>15.4	0.91	58	3	2	2	12.5	LIKLDCGVQHYAWGKEAAESYVAK Carbamidomethyl (C6)<br>YRAPEGSEWELQHYSPPREFQDFSLYR	23.8<br>25.0
250	Tb927.2.2940	hypothetical protein, conserved	1.00	119	9	2	2	16.1	ILSEGAALELPVIADVLQCGPVVSLLRR Carbamidomethyl (C19)<br>LGLLLVQLGAAKENQIFGPR	25.5<br>53.0	1.00	148	9	4	2	16.1	ILSEGAALELPVIADVLQCGPVVSLLRR Carbamidomethyl (C19)<br>LGLLLVQLGAAKENQIFGPR<br>LGLLLVQLGAAKENQIFGPR Deamidated (N14)<br>LGLLLVQLGAAKENQIFGPR Deamidated (Q7)	30.9<br>56.8<br>41.0<br>3.2
251	Tb927.10.10360	microtubule-associated protein, putative	0.89	170	43	4	4	1.2	DVLREEEEVTGPR<br>HVDPSHFR<br>STTAESYAPIDPAAYK<br>STTAESYAPIDPAAYKR	26.2<br>21.6<br>36.6<br>31.1	1.00	163	17	4	4	1.9	DVLREEEEVTGPR<br>HVDESHFLTTTHEAYKPIDPSEYR<br>HVDPSHFR<br>STTAESYAPIDPAAYKR	38.4<br>41.9<br>19.2<br>30.8
252	Tb927.4.1360	hypothetical protein, conserved	1.00	190	15	5	5	27.3	GIPIRGGVPIVFPQFGNMGPLPPHGFAR Oxidation (M18)<br>ILVLPSSKM Oxidation (M9)<br>NDDGSSILVHLQGAHLTNWR<br>NKEGTELLYTSSK<br>VKLQPQFEWK	20.1<br>28.1<br>49.6<br>41.7<br>12.6	0.00	0	0	0	0	0.0		
253	Tb927.11.15150	1-acyl-sn-glycerol-3-phosphate acyltransferase, putative	1.00	98	8	3	3	10.7	AGLETVKGTVAPMMEAAR Oxidation (M13)- Oxidation (M14)<br>KAGLETVKGTVAPMMEAAR Oxidation (M14)- Oxidation (M15)<br>MLAVFPEGAR Oxidation (M1)	24.5<br>8.8<br>45.0	0.00	7	1	1	1	7.0	KAGLETVKGTVAPMMEAAR Oxidation (M14)- Oxidation (M15)	7.5
254	Tb927.10.8200	hypothetical protein, conserved	0.78	50	4	2	2	9.3	CAGLPIFCHTFQIPR Carbamidomethyl (C1)- Carbamidomethyl (C8)<br>VIDSNKLSVK	23.7<br>19.2	1.00	195	5	4	3	20.8	CAGLPIFCHTFQIPR Carbamidomethyl (C1)- Carbamidomethyl (C8)<br>EAGHSEVSDTLEDLFATAPQK<br>SFLTQLTNDPQGVTHENILR<br>SFLTQLTNDPQGVTHENILR Deamidated (N8)	25.8<br>74.6<br>58.7<br>29.9
255	Tb927.7.4900	5'-3' exonuclease XRNA, putative,exoribonuclease 1, putative	1.00	188	11	4	4	5.9	ACGAAPFGATGTVVR Carbamidomethyl (C2)<br>SIEEFIESCGIQSVPLVAAQDDAFPREHVR Carbamidomethyl (C9)<br>SVTTTAAPGSGTFSVPLPR<br>VFQKPVSIPHVLLDYASSR Deamidated (Q3)	40.2<br>47.9<br>30.3<br>27.8	1.00	111	3	3	3	3.2	ACGAAPFGATGTVVR Carbamidomethyl (C2)<br>SVTTTAAPGSGTFSVPLPR<br>TLLAAYETVQDR	11.1<br>19.1<br>80.7
256	Tb927.9.5960	succinate dehydrogenase, putative	1.00	221	17	5	5	33.5	AAAPKPPTVFTAK<br>AAAPKPPTVFTAKDNSK<br>PAAPLPGEVAK<br>TAFHVVTQRPGFATTPYTVSR<br>VGPPLEDAKVASKY	31.9<br>29.5<br>44.3<br>29.4<br>45.5	0.83	32	1	1	1	7.5	VGPPLEDAKVASKY	32.2
257	Tb927.11.9400	hypothetical protein, conserved	1.00	142	7	4	2	5.3	LAQLLISCNGDKLEELTGVRQELADELAGGTLLK Carbamidomethyl (C8)<br>LAQLLISCNGDKLEELTGVRQELADELAGGTLLK Carbamidomethyl (C8)- Deamidated (N9)<br>LAQLLISCNGDKLEELTGVRQELADELAGGTLLK Deamidated (Q3)- Deamidated (N9)- Carbamidomethyl (C8)<br>VLAIQPDGSGNSR	46.5<br>20.2<br>41.3<br>24.7	1.00	170	5	3	1	3.8	LAQLLISCNGDKLEELTGVRQELADELAGGTLLK Carbamidomethyl (C8)<br>LAQLLISCNGDKLEELTGVRQELADELAGGTLLK Carbamidomethyl (C8)- Deamidated (N9)<br>LAQLLISCNGDKLEELTGVRQELADELAGGTLLK Carbamidomethyl (C8)- Deamidated (Q21)- Deamidated (N9)	68.4<br>47.3<br>38.2
258	Tb927.7.210	proline dehydrogenase	0.79	32	2	1	1	3.1	TVVSEAVLALDPTPVQK	31.4	1.00	232	13	6	5	16.4	FKTVVSEAVLALDPTPVQK<br>IIIDKLPK<br>LASSQKYTEDEINAVLQVLDPNNEGKTNYYK<br>LASSQKYTEDEINAVLQVLDPNNEGKTNYYK Deamidated (Q17)- Deamidated (N22)<br>RAEAIFGPFLTYNTLVK<br>YVPYGPVKETVHYLGR	42.1<br>32.3<br>29.4<br>30.8<br>18.1<br>31.1
259	Tb927.11.8870	mitochondrial DEAD box protein,KREH1	1.00	109	10	3	2	6.0	GLDFPDVTCVVNLIAPK Carbamidomethyl (C9)<br>IVVLAPTRELVQQTAK<br>IVVLAPTRELVQQTAK Deamidated (Q12)	10.4<br>27.3<br>38.6	0.00	14	1	1	1	3.1	GLDFPDVTCVVNLIAPK Carbamidomethyl (C9)	14.1
260	Tb927.11.1980	hypothetical protein, conserved,zinc finger protein family member, putative	1.00	66	4	2	2	7.7	LVYDTVDEPLTGEDADPPSNWHEQLKPK<br>TQAPAASINPPR	31.6<br>23.3	1.00	115	4	2	2	9.1	LVYDTVDEPLTGEDADPPSNWHEQLKPK<br>NIPFEELEEEGNFKDAAVK	34.5<br>63.8
261	Tb927.4.4910	3,2-trans-enoyl-CoA isomerase, mitochondrial precursor, putative	1.00	117	9	4	4	12.8	FLSVPQQSR<br>IGLNETKLGITTPPWVIPAYAYVLGSR<br>LDTSDRGITTVALSR<br>LGITTPPWVIPAYAYVLGSR	30.7<br>31.0<br>23.2<br>13.4	0.87	35	4	1	1	5.0	LGITTPPWVIPAYAYVLGSR	14.2
262	Tb927.10.16150	ATP-dependent zinc metallopeptidase, putative,metallo-peptidase, Clan MA(E) Family M41	0.62	80	1	1	1	3.0	GCLLTGQPGTGKTLLAK Carbamidomethyl (C2)	80.5	1.00	99	4	2	2	3.1	KVAPSVVFLDEIDAVGTR	24.4

																	VAPSVVFLDEIDAVGTR	51.7
263	Tb927.11.8050	hypothetical protein, conserved	1.00	91	4	2	2	8.8	TPEFNVTQSVAPVIEAVERDYAALTSSER	29.0	1.00	158	8	5	3	12.2	AAASVLGYHEDEDQFFAK	16.6
									TVQPEPEGGFVGVPLLK	41.2							TPEFNVTQSVAPVIEAVERDYAALTSSER	41.1
																	TPEFNVTQSVAPVIEAVERDYAALTSSER Deamidated (N5)	44.1
																	TPEFNVTQSVAPVIEAVERDYAALTSSER Deamidated (Q8)	31.3
																	TVQPEPEGGFVGVPLLK	11.6
264	Tb927.10.2950	hypothetical protein, conserved	1.00	103	4	2	2	1.5	IGKSNEVTTAAGTAAGSR	62.7	0.00	7	2	1	1	1.0	IGKSNEVTTAAGTAAGSR	7.0
									VAGILTQIR	23.1								
265	Tb927.8.1550	paraflagellar rod component, putative	1.00	186	11	5	5	10.3	DMAIAEELLAAQQSYFSR	3.2	1.00	60	4	2	2	5.3	RGEVEPILQHVISYNR	14.4
									ILSSTVGIDLPR	80.2							RPYATTVPHSGYLSKPTLPPVEALR	36.5
									RPYATTVPHSGYLSKPTLPPVEALR	33.8								
									SVHVDRLQTLQR	12.3								
									TVGINHEEAFLEK	33.5								
266	Tb927.5.4420	nucleolar RNA helicase II, putative,nucleolar RNA helicase Gu, putative	1.00	135	9	3	3	7.6	GLDLPMVDLVIQCAPPTDIDAFIHR Oxidation (M6)- Carbamidomethyl (C13)	32.1	0.59	29	1	1	1	2.5	IKFEVLPAPTREEILK	28.8
									GSATVQMLGQR Oxidation (M7)	39.1								
									SLPTHVFCSILR Carbamidomethyl (C8)	34.8								
267	Tb927.10.770	hypothetical protein, conserved	1.00	202	8	4	3	5.2	INLGPEIEELFAELSSIR	55.8	1.00	117	5	2	1	2.9	INLGPEIEELFAELSSIR	60.1
									INLGPEIEELFAELSSIR Deamidated (N2)	57.9							INLGPEIEELFAELSSIR Deamidated (N2)	45.0
									ISGGAAGPTIDELKK	40.8								
									ISGGAAGPTIDELKKINLGPEIEELFAELSSIR	18.6								
268	Tb927.11.3360	hypothetical protein, conserved	1.00	99	3	2	2	1.8	AATVLQSAVQAYLTR	65.1	1.00	60	2	2	2	1.8	AATVLQSAVQAYLTR	54.8
									AATVLQSAVQAYLTRK Deamidated (Q6)	19.2							AATVLQSAVQAYLTRK Deamidated (Q6)	5.7
269	Tb927.6.4980	40S ribosomal protein S14	1.00	226	19	6	6	29.2	AGMKIGRIEDVTPVPTDSTR Oxidation (M3)	33.8	0.00	4	1	1	1	14.6	MRATGGVGTKSPGPGAQAALR Oxidation (M1)	3.6
									ATGGVGTKSPGPGAQAALR	51.8								
									IGRIEDVTPVPTDSTR	34.5								
									IGRIEDVTPVPTDSTRR	15.4								
									MRATGGVGTKSPGPGAQAALR Oxidation (M1)	25.5								
									SPGPGAQAALR	20.5								
270	Tb927.10.10280	microtubule-associated protein, putative	1.00	209	51	5	5	2.2	DVLREEEEATGPR	21.3	0.67	112	13	3	3	1.6	DVLREEEEVTGPR	38.4
									DVLREEEEVTGPR	26.2							HVDPSHFR	19.2
									HVDPSHFR	21.6							STTAESYAPIDPAAYKR	30.8
									STTAESYAPIDPAAYK	36.6								
									STTAESYAPIDPAAYKR	31.1								
271	Tb927.3.1840	3-oxo-5-alpha-steroid 4-dehydrogenase, putative	1.00	186	12	5	5	29.9	ALTEQGVKDGSEVVYKDLGPQVGYR	32.7	0.00	18	1	1	1	9.1	MKVTVISGSSSEVVELPSNAGLTDLKK Oxidation (M1)	18.0
									MKVTVISGSSSEVVELPSNAGLTDLKK Oxidation (M1)	23.6								
									NKKAMLPFLL Oxidation (M5)	25.6								
									NYIKTDPSVR	33.9								
									SGGDKNDKSAYITLDAK	48.1								
272	Tb927.11.4450	hypothetical protein, conserved	1.00	114	5	3	3	43.6	AISDAVAVAEILKNQGLIDVKK	34.0	0.00	0	0	0	0	0.0		
									LSGGKPEVTISALGK	11.1								
									SKDFDTIYAEQQKR	42.5								
273	Tb927.7.3370	hypothetical protein, conserved	1.00	102	7	4	3	8.7	ATRDEAFNVSDAVLSHQHQIR	9.4	0.00	0	0	0	0	0.0		
									ITELTTEIERLNEQEELIVKGGSVLTQLQQR	39.7								
									ITELTTEIERLNEQEELIVKGGSVLTQLQQR Deamidated (N12)	36.4								
									LNEQEELIVKGGSVLTQLQQR	10.0								
274	Tb927.8.4400	hypothetical protein, conserved	1.00	189	19	5	3	7.9	ILAEVVQHTDVLAEQVSLYKPEVVVPR	30.0	1.00	64	2	1	1	4.0	ILAEVVQHTDVLAEQVSLYKPEVVVPR	56.6
									ILAEVVQHTDVLAEQVSLYKPEVVVPR Deamidated (Q15)	16.3								
									ILAEVVQHTDVLAEQVSLYKPEVVVPR Deamidated (Q7)	42.3								
									TKIENVVTNLQLLGAK	35.9								
									TRLPSAAAGR	23.3								
275	Tb927.11.10760	kinesin-like protein, putative	1.00	144	4	4	4	9.6	AIEKEFESVHQKEGVTR	35.5	0.97	62	3	2	2	4.1	FTDEILAAFKR	25.6
									FTDEILAAFKR	71.7							TLTVLDATNEFLPR	32.0
									ILAFLNASPEER	8.5								
									VYSLYGNSVDGLEGGILPR	28.0								
276	Tb927.10.9780	ATP-dependent DEAD/H RNA helicase, putative,ATP- dependent RNA helicase, putative	1.00	87	6	2	2	7.1	FTHPTPVQEEVLPSVLSQNVDGR	35.2	0.69	30	1	1	1	4.1	ELALQISEAMQQLVQHAPQVIVGCVVGGMAPEKQQR Carbamidomethyl (C24)	30.0
									TLAFAIPILQHVLTNVLVNEVTQGPDGKIGNEETANAAK	26.2								
277	Tb927.11.16510	hypothetical protein, conserved	1.00	170	15	4	4	29.5	APPKASLPAR	27.0	0.00	0	0	0	0	0.0		
									HHYAGPVSIPGSEK	41.5								
									HHYAGPVSIPGSEKLITE	39.7								
									WASDKYAEEMVLLSR Oxidation (M10)	33.6								
278	Tb927.11.17000	hypothetical protein, conserved,leucine-rich repeat protein (LRRP), putative	1.00	134	11	4	3	4.9	EVPLTEEFEVIDTGVR	50.4	1.00	108	6	2	2	3.1	EVPLTEEFEVIDTGVR	44.1
									IVQVPHGDDGGETTR	25.0							IVQVPHGDDGGETTR	33.7
									IVQVPHGDDGGETTR Deamidated (Q3)	11.2								
									LVPDDESRNVLTEGVPVR	20.1								
279	Tb927.9.15360	40S ribosomal protein S6, putative	1.00	156	9	3	2	9.6	MKLNIAYPR Oxidation (M1)	41.0	0.00	0	0	0	0	0.0		
									NGTVKQFEVADEVLR	41.8								
									NGTVKQFEVADEVLR Deamidated (N1)	44.1								
280	Tb927.10.170	pseudouridine synthase, Cbf5p	1.00	136	11	4	4	9.6	RVILPCEYLLTNYKR Carbamidomethyl (C6)	37.5	0.81	30	1	1	1	3.5	RVILPCEYLLTNYKR Carbamidomethyl (C6)	30.4
									RWGLGPVAVK	37.7								
									VILPCEYLLTNYKR Carbamidomethyl (C5)	27.7								
									VVVKDSAVNAVCYGAK Carbamidomethyl (C12)	10.2								
281	Tb927.3.5570	syntaxin, putative	1.00	134	5	3	2	6.9	NAVGHLQQAR	44.1	1.00	77	2	2	1	3.9	NAVGHLQQAR	30.3
									NAVGHLQQAR Deamidated (N1)	51.1							NAVGHLQQAR Deamidated (N1)	46.3
									SCDVFYKK Carbamidomethyl (C2)	31.2								
282	Tb927.10.6630	ATP-dependent DEAD/H RNA helicase HEL64, putative	1.00	177	10	4	4	10.6	AGAKGDAFTLITKK	20.9	1.00	127	9	3	3	8.6	AGAKGDAFTLITKK	13.7
									EAQITPSVLKELIGILER	32.0							EAQITPSVLKELIGILER	53.6
									IKPVDWGNVSLVPGNWK	29.2							IKPVDWGNVSLVPGNWK	43.2
									LCLVATDVAAR Carbamidomethyl (C2)	65.3								
283	Tb927.8.2740	mitochondrial RNA binding protein	1.00	150	6	3	2	12.4	FLIFNVEQLEDPEVALR	62.2	1.00	127	4	2	1	5.0	FLIFNVEQLEDPEVALR	65.5
									TLDRSPVSIPTFLLSGEPMQHEGVK Oxidation (M19)	29.7							FLIFNVEQLEDPEVALR Deamidated (N5)	49.1
									TLDRSPVSIPTFLLSGEPMQHEGVK Oxidation (M19)- Deamidated (Q20)	27.9								
284	Tb927.10.2770	eukaryotic translation initiation factor 5, putative	1.00	197	13	4	4	10.7	SCRNPETQLSVER Carbamidomethyl (C2)	31.6	0.00	0	0	0	0	0.0		
									YFQSEFGAQR	21.5								
									YVTALATHFAK	57.7								
									YVTALATHFAKNPQAALR	39.1								
285	Tb927.7.3980	immunodominant antigen, putative,tc40 antigen-like	1.00	105	7	3	2	2.3	RSPAGAAKPASNVLAPTTGTK	37.7	0.52	39	2	2	1	2.3	RSPAGAAKPASNVLAPTTGTK	24.8
									RSPAGAAKPASNVLAPTTGTK Deamidated (N12)	20.3							RSPAGAAKPASNVLAPTTGTK Deamidated (N12)	14.3
									SPAGAAKPASNVLAPTTGTK	23.6								
286	Tb927.3.3590	U3 small nucleolar ribonucleoprotein protein MPP10, putative	1.00	83	3	2	2	5.1	FVEKLEQAR	35.2	0.00	3	1	1	1	3.7	LAQHLDALSNFHYTPKPVQEDLSAR	3.3
									LAQHLDALSNFHYTPKPVQEDLSAR	43.1								
287	Tb927.11.11540	DNA topoisomerase II, putative	1.00	155	13	4	4	3.4	FGSTCDLPASLIDCVLK Carbamidomethyl (C5)- Carbamidomethyl (C14)	18.6	0.94	36	2	1	1	0.8	LRVPLLSDAAR	32.5

								LRVPLLSDAAR<br>REIALIQCK Carbamidomethyl (C8)<br>VYNNGEGIPVQR Deamidated (N4)	47.5<br>17.4<br>29.9								
288	Tb927.7.1290	hypothetical protein, conserved	1.00	104	9	4	4	10.3	AVTAAGHER<br>QFSGGGADEIKQPAR<br>QFSGGGADEIKQPARR<br>RAVTAAGHER	7.6<br>38.4<br>25.7<br>11.3	0.59	28	1	1	1	6.2	QFSGGGADEIKQPAR	27.5
289	Tb927.11.2790	hypothetical protein, conserved	1.00	117	6	2	2	2.3	ALAEQASKAPAAAGR<br>KALAEQASKAPAAAGR	43.1<br>48.2	0.00	0	0	0	0	0.0		
290	Tb927.8.2000	cyclophilin, putative	1.00	102	7	2	2	8.3	IVMELYADTVPR Oxidation (M3)<br>VTEGIDVVKSIER	42.6<br>40.3	0.00	0	0	0	0	0.0		
291	Tb927.8.1860	pitrilysin-like metalloprotease,metallo-peptidase, Clan ME, Family M16C	1.00	75	3	2	2	2.3	IAREDVVELCR Carbamidomethyl (C10)<br>VACQLLANEYLHR Carbamidomethyl (C3)	33.5<br>29.9	0.98	68	2	2	2	2.8	AENNPLDFIDWLPHLR<br>VACQLLANEYLHR Carbamidomethyl (C3)	34.4<br>34.0
292	Tb927.11.6210	sterol 14-alpha-demethylase	1.00	147	9	4	3	10.8	EVYSFMVPVFGEGVAYAAPYPR<br>EVYSFMVPVFGEGVAYAAPYPR Oxidation (M6)<br>GDIIACSPLLSHHDEEAFPEPR Carbamidomethyl (C6)<br>VGSYVVPKGDIIACSPLLSHHDEEAFPEPR Carbamidomethyl (C14)	7.0<br>45.6<br>24.9<br>58.6	1.00	82	2	2	2	10.8	EVYSFMVPVFGEGVAYAAPYPR<br>VGSYVVPKGDIIACSPLLSHHDEEAFPEPR Carbamidomethyl (C14)	27.5<br>54.8
293	Tb927.6.4130	hypothetical protein, conserved	1.00	150	14	5	4	37.5	ALLSLFDMMNPLP Oxidation (M8)- Oxidation (M9)<br>AYVALPDGRMALVYPIIDTQLTPTR<br>AYVALPDGRMALVYPIIDTQLTPTR Oxidation (M10)<br>MALVYPIIDTQLTPTR Oxidation (M1)<br>RAYVALPDGRMALVYPIIDTQLTPTR Oxidation (M11)	26.0<br>20.8<br>17.5<br>42.9<br>18.0	0.00	0	0	0	0	0.0		
294	Tb927.9.13990	RNA-binding protein, putative	1.00	130	10	3	3	14.4	GSALVTMATREDALQAIR Oxidation (M7)<br>LFDDGPRPQLVQR<br>YTDLIADKTGRPK	19.7<br>33.1<br>45.3	0.99	90	4	3	3	17.7	GFGNANANMTSYNNSAGNFGRGGGYVNR Deamidated (N13)- Deamidated (N14)<br>LFDDGPRPQLVQR<br>YTDLIADKTGRPK	29.3<br>18.6<br>35.1
295	Tb927.6.2050	ribosome biogenesis regulatory protein (RRS1), putative	1.00	119	7	3	3	23.5	AQEEVKHLTAAAK<br>GKFDGSTTLLKLPQPVMQLPR Oxidation (M17)<br>HLATASNGKFDKVSLR Deamidated (N7)	37.8<br>22.8<br>21.8	0.00	0	0	0	0	0.0		
296	Tb927.4.410	CAF 40	1.00	113	4	2	1	10.8	NLLQLLVNIGDAGATKLHESLLAGGKPQGGDASA<br>NLLQLLVNIGDAGATKLHESLLAGGKPQGGDASA Deamidated (N1)	63.0<br>47.7	1.00	136	6	2	1	10.8	NLLQLLVNIGDAGATKLHESLLAGGKPQGGDASA<br>NLLQLLVNIGDAGATKLHESLLAGGKPQGGDASA Deamidated (N8)	47.9<br>64.3
297	Tb927.7.280	cyclophilin-type peptidyl-prolyl cis-trans isomerase, putative	1.00	120	7	3	3	24.0	AYTSPYPQNPK<br>NTNGSQFFICTVPCPWLDGKHVVFGQVLR Deamidated (N3)- Carbamidomethyl (C10)- Carbamidomethyl (C14)<br>TVIISDSGVLKEPA	40.3<br>41.6<br>19.0	0.00	0	0	0	0	0.0		
298	Tb927.10.6050	clathrin heavy chain	0.99	122	7	4	4	4.1	IAGSANLGGVDDLYR<br>ILIMELPTGSKTDLTVQRR Oxidation (M4)<br>SCNLMCLAWNNPSTGGK Carbamidomethyl (C2)- Carbamidomethyl (C6)- Oxidation (M5)<br>TCLRAPNNALRGPDILSR Carbamidomethyl (C2)	15.1<br>36.0<br>19.9<br>22.3	1.00	105	6	4	4	5.6	IAGSANLGGVDDLYR<br>LAAICAVPLVLQVESLQDVVNRYEAYGLYDELFAVLK Carbamidomethyl (C5)<br>LLVISGNLPEKFIEENDYYDPVVMGK<br>TCLRAPNNALRGPDILSR Carbamidomethyl (C2)	52.9<br>27.0<br>7.3<br>12.5
299	Tb927.9.14200	hypothetical protein, conserved	1.00	143	13	3	3	10.4	DGRDKEPEALIWR<br>EGFLEPSDDVVLEYAR<br>WDTTYPPILSR	18.7<br>67.1<br>34.9	0.98	43	2	1	1	4.2	EGFLEPSDDVVLEYAR	35.4
300	Tb927.3.5020	hypothetical protein, conserved	1.00	149	57	4	3	4.1	ALVTQLAPR<br>ALVTQLAPR Deamidated (Q5)<br>ASGDVTPLAVSGAEGSDQKFDLLDRLR<br>NIPGAGVFDPTASNVTSLESQR	16.9<br>12.6<br>35.9<br>23.3	0.00	29	10	2	1	0.6	ALVTQLAPR<br>ALVTQLAPR Deamidated (Q5)	9.2<br>9.2
301	Tb927.8.6390	lysophospholipase, putative,alpha/beta hydrolase, putative	1.00	107	8	3	3	17.1	IAPAGVAALSGYFAAAEKILPQLCNK Carbamidomethyl (C24)<br>TRETELITHGLK<br>YGPLLQIGNR	21.2<br>21.5<br>50.8	0.99	45	2	1	1	4.3	TRETELITHGLK	41.6
302	Tb927.11.6050	hypothetical protein, conserved	1.00	121	7	3	3	3.3	FNPLVFAISVETPKNVCVPR Deamidated (N2)- Carbamidomethyl (C17)<br>PSVPTANTSAPVASAPVDPELQAEALKR<br>REELAMLR	39.6<br>18.2<br>32.8	0.86	55	4	2	2	1.6	FNPLVFAISVETPKNVCVPR Deamidated (N2)- Carbamidomethyl (C17)<br>REELAMLR	9.4<br>37.4
303	Tb927.2.4550	FtsJ cell division protein, putative	1.00	69	5	2	1	1.8	AIVPDIGKLTTQELVR<br>AIVPDIGKLTTQELVR Deamidated (Q12)	45.2<br>7.8	0.99	95	5	3	3	5.6	AIVPDIGKLTTQELVR Deamidated (Q12)<br>VFSDVEEEKILTPSGALVVPR<br>VVLPVTAEEVEAQR	27.7<br>28.9<br>38.1
304	Tb927.9.15330	hypothetical protein, conserved	1.00	79	2	1	1	2.0	VVAMLTGGAAKDAVPSAK Oxidation (M4)	48.8	0.00	10	2	1	1	2.0	VVAMLTGGAAKDAVPSAK Oxidation (M4)	8.6
305	Tb927.11.5220	chaperone protein DNAj, putative	1.00	103	9	2	1	2.9	VLVAYNILSDDIKRSDYDQFGR<br>VLVAYNILSDDIKRSDYDQFGR Deamidated (N6)	27.5<br>36.1	0.64	40	1	1	1	1.8	VLVAYNILSDDIKR Deamidated (N6)	40.5
306	Tb927.11.3500	hypothetical protein, conserved	1.00	86	7	2	2	2.5	LLCAYVGPSHTLGLSPK Carbamidomethyl (C3)<br>RMVLALNR	38.0<br>31.0	1.00	62	4	2	2	4.0	GGFVDSIDKVAHEFIESHNLQPIR<br>LLCAYVGPSHTLGLSPK Carbamidomethyl (C3)	45.0<br>8.6
307	Tb927.6.1470	hypothetical protein, conserved	1.00	131	6	3	3	9.4	KLDDELLQR<br>RKLDDELLQR<br>TRLEGFGYGDR	51.1<br>29.0<br>34.5	1.00	174	9	4	3	9.4	KLDDELLQR<br>KLDDELLQR Deamidated (Q8)<br>RKLDDELLQR<br>TRLEGFGYGDR	48.3<br>28.8<br>34.3<br>34.5
308	Tb927.1.1700	hypothetical protein, conserved	1.00	120	6	3	2	8.5	LVGFLLPVPFAVNGQHEVLFK<br>LVGFLLPVPFAVNGQHEVLFK Deamidated (Q15)<br>SAQVELLHKQESGDTSNPQAR	33.9<br>35.7<br>26.9	0.85	101	7	4	2	8.5	LVGFLLPVPFAVNGQHEVLFK<br>LVGFLLPVPFAVNGQHEVLFK Deamidated (N13)<br>LVGFLLPVPFAVNGQHEVLFK Deamidated (Q15)<br>SAQVELLHKQESGDTSNPQAR	18.8<br>29.5<br>26.9<br>19.8
309	Tb927.2.5130	hypothetical protein, conserved	1.00	104	3	2	1	9.2	VVTLVEPTKWANLIEEYPDTLYSSESAKNVENVLSK<br>VVTLVEPTKWANLIEEYPDTLYSSESAKNVENVLSK Deamidated (N12)	46.4<br>41.7	0.00	15	1	1	1	7.2	VVTLVEPTKWANLIEEYPDTLYSSESAK	15.0
310	Tb927.8.2630	kinesin, putative	0.99	42	4	1	1	3.3	TLPQVPFRDSVLTWLLSDSIGGNAR	32.1	1.00	79	4	2	2	5.3	LAQKNATVANSNRQR Deamidated (Q3)<br>TLPQVPFRDSVLTWLLSDSIGGNAR	31.4<br>33.7
311	Tb927.11.6300	40S ribosomal protein S5, putative	1.00	123	4	3	3	15.3	NLKTLPECLADEIVNASK Carbamidomethyl (C8)<br>TLPECLADEIVNASK Carbamidomethyl (C5)<br>TPTYVPHSAGR	32.4<br>54.5<br>30.2	0.00	0	0	0	0	0.0		
312	Tb927.9.1750	hypothetical protein, conserved	1.00	148	12	4	4	2.3	HAASSSGSSPAPGGVKVPSLPINAPLSGR<br>IVLCASQAKPYFR Carbamidomethyl (C4)<br>LVSPPDAQSSGSPTGIASPPAHGDPLLGGAR<br>VYEVGPNAAANEGSLLIDKPLSGSETTYVAR	18.4<br>22.6<br>26.4<br>46.4	0.00	0	0	0	0	0.0		
313	Tb927.9.11000	small GTPase, putative,GTP-binding protein, putative	1.00	108	6	2	2	6.8	ATAEEGVALPQSIR<br>KATAEEGVALPQSIR	12.8<br>58.6	1.00	104	2	2	1	6.8	KATAEEGVALPQSIR<br>KATAEEGVALPQSIR Deamidated (Q12)	66.2<br>38.2
314	Tb927.9.11850	structural maintenance of chromosome 1, putative	1.00	183	8	4	3	4.2	LVAANDAQLSQYKQR<br>RLPPLTFLPLNSVNGSAVDDRLR<br>RLPPLTFLPLNSVNGSAVDDRLR Deamidated (N14)<br>TGELAKIELAKAAAAR	56.9<br>34.9<br>43.4<br>21.4	0.00	4	1	1	1	1.8	RLPPLTFLPLNSVNGSAVDDRLR Deamidated (N14)	3.8
315	Tb927.10.4760	hypothetical protein, conserved	1.00	190	12	4	4	15.7	KLPSEAFQQR<br>LLLEGK<br>SLSDELEYAQDKHR<br>SLSDELEYAQDKHRWVYGDAPR	58.5<br>31.4<br>43.9<br>30.4	0.00	8	1	1	1	9.1	SLSDELEYAQDKHRWVYGDAPR	7.9

316	Tb927.6.3890	replication factor C, subunit 2, putative	1.00	121	8	4	3	14.1	SLDEVKSQEEVVQALR<br>SLDEVKSQEEVVQALR Deamidated (Q8)<br>SSIDALDHVSGGDLR<br>TTAILAVAHELFGPDYIK	21.0<br>43.5<br>27.5<br>14.1	1.00	75	3	2	2	9.8	SLDEVKSQEEVVQALR Deamidated (Q8)<br>TTAILAVAHELFGPDYIK	19.1<br>46.2
317	Tb927.11.16480	enoyl-CoA hydratase/isomerase family protein, putative	1.00	88	6	2	2	8.0	ATDPAEVFSTDYVGAIR<br>THACGDFPEGVR Carbamidomethyl (C4)	50.7<br>22.4	0.73	55	5	3	3	16.0	ATDPAEVFSTDYVGAIR<br>TAISTMEKLSPTSLK Oxidation (M6)<br>VFTLKDTSTVEDVMGSLAADGGEFAR	22.2<br>1.8<br>23.3
318	Tb927.7.2190	hypothetical protein, conserved	1.00	56	4	1	1	8.3	RVEVGTKGDGYDPDYVSEEHLR	50.1	0.76	30	2	1	1	5.3	VRGGSTPTFHAGSR	21.1
319	Tb927.7.3430	cyclophilin-type peptidyl-prolyl cis-trans isomerase, putative	0.99	111	7	4	4	34.2	ALHTMPGKEVLDILSPRY Oxidation (M5)<br>AWITECGQSSGDKLEDTERALSGER Carbamidomethyl (C6)<br>VIPGFIVQGGDILLKDGR<br>VIPGFIVQGGDILLKDGRGNVSVFGFPFPDESFEGK	35.4<br>16.5<br>27.3<br>22.4	0.00	0	0	0	0	0.0		
320	Tb927.11.3770	hypothetical protein, conserved	1.00	122	4	3	2	12.2	LLNTNVRAPPLISAANGDTEVTEQ<br>LLNTNVRAPPLISAANGDTEVTEQ Deamidated (N16)<br>TVLLVRPR	54.8<br>39.0<br>19.4	0.00	0	0	0	0	0.0		
321	Tb927.7.3330	hypothetical protein, conserved	1.00	122	14	3	3	1.1	CAVVVDAANACR Carbamidomethyl (C1)- Carbamidomethyl (C11)<br>VVEFDVTHPSSIHPSEIAQR<br>VYRDCVSNVTFTPSGR Carbamidomethyl (C5)	28.3<br>34.1<br>26.9	0.90	41	2	2	2	1.0	ELDVQNEKLHEMEEQLAELRAENEELR Deamidated (Q5)<br>VYRDCVSNVTFTPSGR Carbamidomethyl (C5)	34.5<br>6.7
322	Tb927.11.7900	mitochondrial RNA binding protein,RBP16,RNA- binding protein of 16 kDa	1.00	142	7	5	3	51.8	GFGFIEDDADKKQHFVHFSALQTETGGFR<br>GFGFIEDDADKKQHFVHFSALQTETGGFR Deamidated (Q13)<br>GRNDNQQGGQHQSFSDDF<br>GRNDNQQGGQHQSFSDDF Deamidated (N3)<br>TRAENVTSPGGAKLPSGPRPPEGAGR	43.3<br>30.5<br>16.0<br>4.8<br>29.7	0.00	0	0	0	0	0.0		
323	Tb927.11.10240	hslVU complex proteolytic subunit, putative,hslVU complex proteolytic subunit, threonine peptidase, Clan T(1), family T1B	1.00	153	6	2	2	12.0	AAVELAKEWR<br>ALIDVDGYDAEKIAR	53.4<br>68.9	0.00	0	0	0	0	0.0		
324	Tb927.6.2640	importin alpha subunit, putative	0.00	22	1	1	1	5.1	DIALQHNAIPAFVNLISDPDQPLSIVR	21.8	1.00	51	5	1	1	5.1	DIALQHNAIPAFVNLISDPDQPLSIVR	43.0
325	Tb927.10.4000	methylglutaconyl-CoA hydratase, mitochondrial precursor, putative	1.00	156	12	3	3	24.9	APLVMGLSNALQLILTADVVPAERAQR Oxidation (M5)<br>FGLPETGLAIIPGAGGTYR<br>LGLVTELVPAGGASEAALAVAGR	36.0<br>23.7<br>42.0	1.00	96	8	2	2	15.2	FGLPETGLAIIPGAGGTYR<br>LGLVTELVPAGGASEAALAVAGR	12.9<br>56.6
326	Tb927.4.1540	hypothetical protein, conserved	1.00	114	8	2	2	4.7	IAPGATVSTILSQLSSETEKER<br>IAPGATVSTILSQLSSETEKERAK	74.7<br>18.9	1.00	80	6	1	1	4.3	IAPGATVSTILSQLSSETEKER	65.3
327	Tb927.6.4630	kinetoplastid-specific phospho-protein phosphatase, putative	0.00	7	1	1	1	5.2	NLLGDYSAVDPGSLAGSGGVSGR	6.6	1.00	210	9	3	3	17.6	EAQSVGGDVIELLGNHEIR<br>ITGLVDDRQHWIGGDSFFVQLGDIFDVGPDDISIVK<br>NLLGDYSAVDPGSLAGSGGVSGR	39.6<br>44.8<br>56.1
328	Tb927.11.5600	hypothetical protein, conserved	1.00	162	7	3	2	16.8	LLQEEASQGVR<br>LLQEEASQGVR Deamidated (Q3)<br>SNTIEGGKKLLQEEASQGVR	30.1<br>40.1<br>40.0	0.00	9	1	1	1	16.8	SNTIEGGKKLLQEEASQGVR	9.3
329	Tb927.10.5990	hypothetical protein, conserved	1.00	134	6	2	2	3.2	SIVLALQTLQEK<br>SIVLALQTLQEKIR	44.3<br>72.2	1.00	56	1	1	1	2.7	SIVLALQTLQEK	55.6
330	Tb927.2.3610	hypothetical protein, conserved	1.00	185	10	3	3	10.4	KLAALTDAGIQK<br>LAALTDAGIQK<br>SAKKLAALTDAGIQK	43.4<br>32.0<br>62.9	0.00	0	0	0	0	0.0		
331	Tb927.11.10150	hypothetical protein, conserved	0.97	41	1	1	1	1.4	LFNTLETAGTSVEPWAR	40.7	1.00	80	2	2	2	4.4	LFNTLETAGTSVEPWAR<br>TLTEIEQLPFQSTHNTSDESHITPYTLGSVAAGVQR	21.1<br>59.3
332	Tb927.7.640	hypothetical protein, conserved	1.00	130	15	2	2	5.1	VKEEVAQLAAEK<br>VKEEVAQLAAEKVSASR	22.8<br>61.3	1.00	115	5	3	2	5.1	VKEEVAQLAAEK<br>VKEEVAQLAAEKVSASR<br>VKEEVAQLAAEKVSASR Deamidated (Q7)	16.3<br>49.6<br>37.9
333	Tb927.7.5940	Protein Associated with Differentiation	1.00	68	4	1	1	6.1	IKEAEDNQTNAENVCAEPTCDDNADACLEEKAADSSK Carbamidomethyl (C15)- Carbamidomethyl (C20)- Carbamidomethyl (C27)	57.3	0.00	0	0	0	0	0.0		
334	Tb927.10.7380	hypothetical protein, conserved	1.00	172	7	3	2	15.8	ILPVAPVEGTPAHTAVEQLLAEVVQGCEALGASVADPVTATR Carbamidomethyl (C27)<br>ILPVAPVEGTPAHTAVEQLLAEVVQGCEALGASVADPVTATR Deamidated (Q18)- Carbamidomethyl (C27)<br>VLNERPLQNQYVR	63.8<br>56.5<br>25.6	1.00	100	7	1	1	12.0	ILPVAPVEGTPAHTAVEQLLAEVVQGCEALGASVADPVTATR Carbamidomethyl (C27)	60.4
335	Tb927.11.4680	hypothetical protein, conserved	1.00	122	6	3	3	17.1	KLGTVAAPR<br>KPPVYELPPGDATIDEFEFLPAAFEGTFDNEGSMLVGPR Oxidation (M34)<br>QMTAQTTLLER Oxidation (M2)	41.8<br>25.8<br>42.1	0.00	0	0	0	0	0.0		
336	Tb927.9.2320	hypothetical protein, conserved	1.00	89	3	2	2	10.9	NVVQSALNPNIQPIKYEAPLQGDITVER<br>TACLPDGAVPQR Carbamidomethyl (C3)	65.0<br>18.1	1.00	142	3	3	1	7.6	NVVQSALNPNIQPIKYEAPLQGDITVER<br>NVVQSALNPNIQPIKYEAPLQGDITVER Deamidated (N1)<br>NVVQSALNPNIQPIKYEAPLQGDITVER Deamidated (Q4)	46.8<br>39.8<br>55.4
337	Tb927.10.11520	hypothetical protein, conserved	1.00	134	5	3	3	27.4	ATQAFQSVGALR<br>ATQAFQSVGALRMQSNILHR Oxidation (M13)<br>CLDTEELYTLLR Carbamidomethyl (C1)	53.6<br>31.9<br>29.1	0.00	0	0	0	0	0.0		
338	Tb927.10.3040	hypothetical protein, conserved	1.00	171	18	4	4	20.0	APIESIIFKY<br>AVLDEYREAVVDGK<br>AVLDEYREAVVDGKVK<br>RAPIESIIFKY	40.9<br>38.7<br>26.2<br>34.8	0.00	0	0	0	0	0.0		
339	Tb927.10.14700	hypothetical protein, conserved	1.00	117	6	3	3	9.9	GGSSPSFLEIAR<br>SLRDSTTINR<br>VRQPDLGEHPLR	61.0<br>15.5<br>26.9	0.00	0	0	0	0	0.0		
340	Tb927.7.680	chaperone protein DNAj, putative	1.00	121	5	2	2	4.8	FAAQNVLQDVAITEEQRR<br>SGQPQSEVADALIDELSGR	33.2<br>68.5	1.00	117	5	2	2	4.8	FAAQNVLQDVAITEEQRR<br>SGQPQSEVADALIDELSGR	13.9<br>60.2
341	Tb927.5.4330	dihydrolipoamide branched chain transacylase, putative	1.00	112	8	4	4	12.3	GLVVPVVRDVQQK<br>SVAELVHEVNELVTLGR<br>VLATPAVREFAR<br>VLREDVLSYAGK	26.4<br>11.3<br>35.3<br>24.9	0.99	50	3	2	2	6.6	SVAELVHEVNELVTLGR<br>VLATPAVREFAR	27.7<br>2.1
342	Tb927.8.610	hypothetical protein, conserved	1.00	127	6	3	2	3.2	NEAQELRLR<br>VAAAGVTVTKLQEELQAARNEAQELR<br>VAAAGVTVTKLQEELQAARNEAQELR Deamidated (Q12)	29.7<br>44.7<br>30.7	0.00	13	1	1	1	3.0	VAAAGVTVTKLQEELQAARNEAQELR	12.9
343	Tb927.4.4700	hypothetical protein, conserved	0.96	63	3	2	2	7.4	HTFPAIGQPR<br>LLAEAAAAAR	35.9<br>25.1	0.00	0	0	0	0	0.0		
344	Tb927.10.12590	hypothetical protein, conserved	1.00	131	6	2	2	4.6	ATTGPLGTSAFAAEDLQR<br>TTALVAGQKTEELAAAMAAENHAIR Oxidation (M17)	57.0<br>40.6	0.00	0	0	0	0	0.0		
345	Tb927.7.3500	glutathione-S-transferase/glutaredoxin, putative	1.00	67	4	2	2	12.5	FHGGSKPDLADLDTHGVLQSVR<br>LYNEIIEATTIKPWLER	39.4<br>22.7	1.00	135	12	3	2	12.5	FHGGSKPDLADLDTHGVLQSVR<br>LYNEIIEATTIKPWLER<br>LYNEIIEATTIKPWLER Deamidated (N3)	20.0<br>47.0<br>30.4
346	Tb927.11.900	isocitrate dehydrogenase, putative	1.00	131	14	3	3	13.3	HAFGDQYSATDAVVKEPGTFEMR Oxidation (M22)<br>LVTTWKKPVVIGR<br>TILGGTVFREPIICSNVPR Carbamidomethyl (C14)	31.0<br>18.1<br>47.0	0.00	12	1	1	1	4.6	TILGGTVFREPIICSNVPR Carbamidomethyl (C14)	11.7

347	Tb927.5.2790	mitochondrial DNA polymerase beta-PAK	1.00	143	8	5	4	9.1	AKSPPAPPPAALPAPAPAAVPVTPK<br>QAAAAASAPVSPRPVKQPAGTR<br>QAAAAASAPVSPRPVKQPAGTR Deamidated (Q1)<br>SPAFVGTIEDAVDDEKDGEAAVGKNAK<br>SPPAPPPAALPAPAPAAVPVTPK	16.8<br>22.3<br>34.6<br>23.7<br>28.4	0.00	0	0	0	0	0.0		
348	Tb927.5.4470	hypothetical protein, conserved	1.00	121	6	3	2	3.6	AVGVYGGGCTPGTYLK Carbamidomethyl (C9)<br>LVLANAASQEVEQR<br>LVLANAASQEVEQR Deamidated (N5)	14.4<br>34.3<br>39.2	0.99	48	2	2	2	3.0	LVLANAASQEVEQR<br>VLNERVENLTK	46.6<br>1.8
349	Tb927.3.5430	hypothetical protein, conserved	1.00	142	9	3	3	15.9	LKPLYGIVSQR<br>SKSLIEAERR<br>SVSLGSREEQIAFWEGEIEKLR	43.6<br>33.3<br>46.1	0.00	0	0	0	0	0.0		
350	Tb927.10.4080	hypothetical protein, conserved	1.00	103	20	2	2	1.7	AAPAAPELQK<br>AAPAAPELQKAAPAAPELQK	29.4<br>52.2	0.41	35	2	1	1	0.9	AAPAAPELQK	29.6
351	Tb927.10.7810	hypothetical protein, conserved	1.00	95	6	2	2	6.5	LASDAAAEAIDTRFQSK<br>YVDDSEVFENPVNKADRIPANYQPLAAFK	33.5<br>40.1	0.00	0	0	0	0	0.0		
352	Tb927.11.10690	hypothetical protein, conserved	0.78	30	1	1	1	1.7	TLVDCRR Carbamidomethyl (C5)	29.6	0.99	73	6	2	2	10.1	AAVLEELIALHPEYAPLR<br>AFVLPFDSSPTPPSPSDLDSAAR	21.3<br>24.9
353	Tb927.10.5840	translation elongation factor 1-beta, putative	0.00	17	2	2	2	17.9	DGLLWGDHKLVPIAFGVK<br>LSAQPYVSGFTPSKEDAR	6.0<br>11.1	1.00	135	5	4	4	44.8	AAAGDDDDIDLFGEATEEELAALEAK<br>DGLLWGDHKLVPIAFGVK<br>LSAQPYVSGFTPSKEDAR<br>SSILFDIKPWDDTVDLDGLAQKLHAIKR	35.7<br>28.7<br>35.8<br>32.9
354	Tb927.3.750	hypothetical protein, conserved	1.00	149	6	3	2	10.2	VSPIQGTIDSPNDGFVTK<br>VSPIQGTIDSPNDGFVTKDVKIDINPNIGGAYAR<br>VSPIQGTIDSPNDGFVTKDVKIDINPNIGGAYAR Deamidated (Q5)	43.1<br>44.2<br>48.5	0.00	0	0	0	0	0.0		
355	Tb927.8.1270	hypothetical protein, conserved	1.00	90	6	3	3	7.5	AREVVETSPLQK<br>QGAIILPEEAAEVR<br>TAVATAVAAGGKSDGR	23.2<br>12.9<br>40.4	0.97	39	2	1	1	2.9	TAVATAVAAGGKSDGR	27.9
356	Tb927.4.1920	GPI transamidase, putative	1.00	70	3	2	1	2.6	LVVNPTPEVPTSGNSNTK<br>LVVNPTPEVPTSGNSNTK Deamidated (N16)	41.6<br>5.5	0.97	66	2	2	2	6.7	LVVNPTPEVPTSGNSNTK Deamidated (N16)<br>SGNDNQALGNVEVTPTAQHFISAVGYDR	31.7<br>33.8
357	Tb927.7.3050	hypothetical protein, conserved	0.00	18	1	1	1	2.3	VKDATAEAAGVTHIPAADFTPFCAFLR Carbamidomethyl (C23)	18.4	1.00	66	2	1	1	2.3	VKDATAEAAGVTHIPAADFTPFCAFLR Carbamidomethyl (C23)	43.3
358	Tb927.11.880	cyclophilin a,cyclophilin type peptidyl-prolyl cis-trans isomerase	1.00	70	3	2	2	18.6	ALCTGEKGFGYAGSGFHR Carbamidomethyl (C3)<br>SIYGEKFPDESFAGK	31.2<br>24.9	0.00	0	0	0	0	0.0		
359	Tb927.1.2100	calpain-like cysteine peptidase, putative,cysteine peptidase, Clan CA, family C2, putative	1.00	87	6	3	3	4.0	AVPLTEEYYAR<br>HTQEVNDLFKPR<br>VVQNSSADAYHPLSDKWTFYQAR	23.7<br>31.2<br>1.5	0.00	0	0	0	0	0.0		
360	Tb927.5.800	casein kinase I, isoform 2	1.00	89	4	3	3	15.4	IHDTLQEGRADQQQQQQQQQR<br>ISDRKQTTSVETLCR Carbamidomethyl (C14)<br>SFPAEFAAYLNYTR	35.1<br>12.5<br>39.5	0.00	0	0	0	0	0.0		
361	Tb927.10.8590	flagellum transition zone component	1.00	129	6	2	1	2.7	ESAGSIASSENKLSDVNVAVPPPRVSVPPAPVPR<br>ESAGSIASSENKLSDVNVAVPPPRVSVPPAPVPR Deamidated (N17)	82.6<br>34.9	0.00	0	0	0	0	0.0		
362	Tb927.10.7230	hypothetical protein, conserved	0.99	60	5	2	2	1.5	ALCHVDHDPTDLSEWAENPLVR Carbamidomethyl (C3)<br>ALLFVKPHANTPETR	23.9<br>15.2	0.00	11	1	1	1	0.9	ALCHVDHDPTDLSEWAENPLVR Carbamidomethyl (C3)- Deamidated (N18)	10.9
363	Tb927.11.9530	14-3-3-I protein	1.00	91	3	2	2	16.0	LLRDNLNLWTDEQPVN<br>QAYDEAVTELETLDDEAYRESNLIVR	29.0<br>57.5	1.00	128	5	3	2	16.0	LLRDNLNLWTDEQPVN<br>QAYDEAVTELETLDDEAYRESNLIVR<br>QAYDEAVTELETLDDEAYRESNLIVR Deamidated (Q1)	29.2<br>44.6<br>29.8
364	Tb927.4.3450	hypothetical protein, conserved	1.00	78	4	1	1	21.1	VLDETYTEWTEELDQAHVTDAITR	71.4	0.00	5	1	1	1	21.1	VLDETYTEWTEELDQAHVTDAITR	4.8
365	Tb927.11.2430	dynein heavy chain, putative	1.00	56	3	1	1	0.4	SGPIVVESQLLNVVQR	51.0	0.00	35	2	2	2	1.3	LLSDLANSSGDLLENTALIQALNDVKVQASSITEALEK Deamidated (N7)<br>SGPIVVESQLLNVVQR	31.8<br>3.5
366	Tb927.10.1660	hypothetical protein, conserved	0.99	63	5	2	2	11.5	DVAFGTLREILSSGNQR<br>TVLSVSPDERHGSVPFQ	14.1<br>31.2	0.95	66	4	2	2	9.5	DVAFGTLREILSSGNQR<br>RMIINEDPQER Oxidation (M2)	32.2<br>26.2
367	Tb927.11.1620	hypothetical protein, conserved	1.00	110	3	2	2	16.7	KFELLPTSAEAIEAR<br>NLSHVVNTIDSAIVETR	40.8<br>64.1	0.00	0	0	0	0	0.0		
368	Tb927.1.2670	flagellar protein PF16	1.00	89	5	2	2	3.5	ALKNIIQR<br>VLPTDIAAKR	44.2<br>28.5	1.00	49	1	1	1	2.3	SAAAWSLGQIGR	48.7
369	Tb927.11.9570	hypothetical protein, conserved	1.00	135	4	3	2	14.1	GLRDDEQYAVSAGLPIQSADVALAR<br>LSALQVQLLESNLCEIR Carbamidomethyl (C14)<br>LSALQVQLLESNLCEIR Deamidated (Q5)- Carbamidomethyl (C14)	27.4<br>56.3<br>45.5	0.44	66	2	2	1	5.7	LSALQVQLLESNLCEIR Deamidated (N12)- Carbamidomethyl (C14)<br>LSALQVQLLESNLCEIR Deamidated (Q5)- Carbamidomethyl (C14)	44.6<br>21.8
370	Tb927.10.14980	intraflagellar transport protein IFT52, putative	0.40	25	1	1	1	2.3	VAVEQQEELPRDFTK	25.4	1.00	73	5	2	2	6.3	LNQIDADEPDITDYHHVPDIASLSER<br>VAVEQQEELPRDFTK	31.1<br>17.4
371	Tb927.11.15550	NADH-cytochrome b5 reductase, putative	1.00	63	4	1	1	6.5	FKPLALGEVINLAEDVAIFR	46.8	1.00	114	7	1	1	6.5	FKPLALGEVINLAEDVAIFR	72.2
372	Tb927.3.4650	C-8 sterol isomerase, putative	0.99	68	7	3	2	6.8	MPGPCFALEYAR Oxidation (M1)- Carbamidomethyl (C5)<br>QYKMPGPCFALEYAR Deamidated (Q1)- Oxidation (M4)- Carbamidomethyl (C8)<br>QYKMPGPCFALEYAR Oxidation (M4)- Carbamidomethyl (C8)	33.0<br>3.3<br>16.0	0.00	0	0	0	0	0.0		
373	Tb927.7.2710	NADH-cytochrome b5 reductase, putative	1.00	88	4	1	1	6.3	IGILASDTGIAPVYQVAR	72.5	1.00	93	3	1	1	6.3	IGILASDTGIAPVYQVAR	78.4
374	Tb927.5.1710	ribonucleoprotein p18, mitochondrial precursor, putative	1.00	119	9	2	2	21.8	KYDLFGYEVDTNTAPWIEK<br>TEGKEHPGHLSQQAGLFDVKVE	60.6<br>27.5	0.00	0	0	0	0	0.0		
375	Tb927.3.5370	hypothetical protein, conserved	1.00	87	5	2	2	12.5	RVPEPSTPITEALTTPESVKK<br>YFVITKPEVDDDDLPLAK	19.7<br>57.8	0.26	24	1	1	1	6.7	RVPEPSTPITEALTTPESVKK	24.1
376	Tb927.4.470	snoRNP protein GAR1, putative	0.99	59	5	2	2	17.0	FNAFVYGANKAK<br>TVPGIQAESISAGDTIYISPTQISPLR	44.3<br>12.4	0.96	64	7	2	2	17.0	FNAFVYGANKAK<br>TVPGIQAESISAGDTIYISPTQISPLR	26.2<br>28.3
377	Tb927.11.2610	hypothetical protein, conserved	0.97	85	5	3	3	8.2	AGGDENLPHLDELHLER<br>IIRGEPLAGTR<br>VQRLEGFAQR	22.1<br>28.5<br>30.9	1.00	82	3	2	2	6.1	AGGDENLPHLDELHLER<br>IIRGEPLAGTR	32.4<br>43.2
378	Tb927.11.4880	hypothetical protein, conserved	1.00	138	8	4	4	17.9	AHVFVYDDRN<br>GVPIVDATKFVK<br>KNEGVGTASR<br>NYVVANAVENVLALPTK	34.8<br>26.4<br>31.0<br>30.5	0.00	22	1	1	1	6.2	NYVVANAVENVLALPTK	21.8
379	Tb927.3.2310	flagellar component,PACRGA	1.00	66	4	2	2	6.7	ARGDAIDFGQR<br>FAYKGTVQR	42.2<br>13.9	0.00	0	0	0	0	0.0		
380	Tb927.11.5420	hypothetical protein, conserved	0.96	75	2	2	2	9.7	ELEKMAAKNR Oxidation (M5)<br>NAAAALVDPALKDEEIDILETR	29.3<br>45.5	0.00	0	0	0	0	0.0		

381	Tb927.9.6870	RNA-binding protein, putative	1.00	64	6	2	2	18.9	ACEFVETFNGKEFR Carbamidomethyl (C2)<br>RPPGFAFITIAPSR	12.2<br>35.1	0.26	22	2	1	1	9.5	RPPGFAFITIAPSR	20.3
382	Tb927.10.6220	5'-3' exoribonuclease XRND, putative,5'-3' exoribonuclease 2, putative	1.00	112	5	3	3	7.2	DVQIVCPDAAIAEKTAHGGAIECNAAR Carbamidomethyl (C6)- Carbamidomethyl (C23)<br>HPVVQLLAVLPR<br>SSMKPIPTEVAAKFPIAGR Oxidation (M3)	9.7<br>57.3<br>39.8	1.00	58	2	1	1	1.5	HPVVQLLAVLPR	57.6
383	Tb927.7.1670	hypothetical protein, conserved	1.00	87	5	2	2	18.8	TKVQATQAVPLVLAAVMGEHLR Oxidation (M17)<br>TVTDGPSHADVVR	31.7<br>30.1	0.00	0	0	0	0	0.0		
384	Tb927.10.11760	pumilio/PUF RNA binding protein 6	0.99	81	8	3	2	2.7	IAHILNHAVEFAQDQEGSR<br>IAHILNHAVEFAQDQEGSR Deamidated (Q15)<br>IAHILNHAVEFAQDQEGSRFIQR	10.5<br>34.5<br>22.9	0.99	43	2	1	1	2.3	IAHILNHAVEFAQDQEGSR	34.8
385	Tb927.4.560	dynein heavy chain, putative	1.00	46	7	1	1	0.7	TEANTPILLITTTGADPSQELQAIAHQK	33.5	0.00	0	0	0	0	0.0		
386	Tb927.5.2850	radial spoke protein RSP2, putative	0.99	99	6	3	2	9.9	AVAETVLAQPSNPQEYIALYLLHVLQEEQNAAIAATR<br>AVAETVLAQPSNPQEYIALYLLHVLQEEQNAAIAATR Deamidated (Q9)<br>GTLPSSDAMEKLVR	36.4<br>31.3<br>29.9	1.00	126	5	2	1	7.1	AVAETVLAQPSNPQEYIALYLLHVLQEEQNAAIAATR<br>AVAETVLAQPSNPQEYIALYLLHVLQEEQNAAIAATR Deamidated (Q9)	44.4<br>54.0
387	Tb927.5.2780	mitochondrial DNA polymerase beta	1.00	119	7	2	2	11.6	AAATLFDREGIFTVEDLIEKADQIQLTEQQR<br>LKAIQELTQIHGFGPR	40.8<br>54.5	1.00	49	4	1	1	7.6	AAATLFDREGIFTVEDLIEKADQIQLTEQQR	38.9
388	Tb927.10.9920	hypothetical protein, conserved	1.00	138	8	4	3	12.4	GGLGGESSSPFPNLSAQLAGQKR<br>NNAGSGTKGGLGGESSSPFPNLSAQLAGQKR<br>NNAGSGTKGGLGGESSSPFPNLSAQLAGQKR Deamidated (N2)<br>RSTTVPPVLSVLPLPSDAAR	21.0<br>38.6<br>32.0<br>23.1	0.00	4	1	1	1	4.9	RSTTVPPVLSVLPLPSDAAR	4.4
389	Tb927.11.16830	Rab-like 5, small G protein	0.84	54	3	2	2	17.8	ALETSLDYQSDNFKEAFDALVEQIIASR<br>STVTNFLAGTR	22.5<br>24.5	1.00	64	2	1	1	12.8	ALETSLDYQSDNFKEAFDALVEQIIASR	50.0
390	Tb927.7.920	dynein heavy chain, putative	1.00	71	2	1	1	0.5	ILHLLTSSTGNILDNEPLVR	46.9	0.00	0	0	0	0	0.0		
391	Tb927.11.5180	hypothetical protein, conserved	1.00	90	9	2	2	8.4	CSACGAVIEHAVR Carbamidomethyl (C1)- Carbamidomethyl (C4)<br>LLRPIIER	51.6<br>27.0	0.00	0	0	0	0	0.0		
392	Tb927.10.15250	paraflagellar rod component, putative	1.00	52	2	1	1	2.7	LAEILDQPEVHER	39.7	0.99	45	2	1	1	2.7	LAEILDQPEVHER	41.3
393	Tb927.7.6660	chaperone protein DNAj, putative	1.00	93	7	2	2	12.1	GSGVPYGSVPADIESSR<br>KPREEDVLKNISGR	52.0<br>22.2	0.00	0	0	0	0	0.0		
394	Tb927.3.3330	heat shock protein 20, putative	1.00	99	4	1	1	10.6	VLPLTGAGSFSSFSR	86.5	0.00	0	0	0	0	0.0		
395	Tb927.1.3950	D-alanine aminotransferase	1.00	60	4	1	1	3.4	VLVVINPGNPTGGVLER	57.7	0.00	0	0	0	0	0.0		
396	Tb927.8.4230	hypothetical protein, conserved	0.99	48	2	1	1	1.6	TIQAAVIEEFVANKGNR	30.0	0.00	0	0	0	0	0.0		
397	Tb927.8.7040	hypothetical protein, conserved	1.00	128	15	3	3	24.3	ATEARVEEEAEQTLELVNTLIR<br>ATEARVEEEAEQTLELVNTLIRK<br>AVGKDEEKEEYSIPTAEILDAVR	22.2<br>25.0<br>47.2	0.00	0	0	0	0	0.0		
398	Tb927.5.550	vacuolar ATP synthase, putative	1.00	97	4	1	1	7.1	LFPSFGSLVDIHDDIAEAESVEQLRER	69.5	1.00	83	12	1	1	7.1	LFPSFGSLVDIHDDIAEAESVEQLRER	53.0
399	Tb927.2.4110	mitochondrial processing peptidase alpha subunit, putative,metallo-peptidase, Clan ME, Family M16	1.00	109	7	2	2	4.9	TVLAATGVADHK<br>TVLAATGVADHKSFAEEVEKLLR	20.6<br>62.2	0.00	0	0	0	0	0.0		
400	Tb927.9.10580	3-demethylubiquinone-9 3-methyltransferase, putative	0.99	68	3	2	2	9.0	GVSAAEISKFSR<br>VLRDITPQASLAYR	38.9<br>26.1	0.37	44	1	1	1	3.1	FIPPADLSR	43.6
401	Tb927.11.3290	hypothetical protein, conserved	0.99	58	3	2	2	2.1	LLQLEDVIKKTPR<br>QQQATQDKVDGVVEALAK Deamidated (Q2)- Deamidated (Q3)- Deamidated (Q6)	38.5<br>12.6	0.71	31	1	1	1	1.2	QQQATQDKVDGVVEALAK Deamidated (Q2)- Deamidated (Q3)- Deamidated (Q6)	30.9
402	Tb927.11.4000	hypothetical protein, conserved	1.00	98	5	2	2	11.2	AGSTTLAQNANAVSYLPASSLPDRAEGATLPTHAPR<br>LLPLVALAQLRDADYFLTTGPKPVSEIVK	68.5<br>17.0	0.00	0	0	0	0	0.0		
403	Tb927.4.590	hypothetical protein, conserved	1.00	55	7	2	1	1.6	FVGQVEGAALHPK<br>FVGQVEGAALHPK Deamidated (Q4)	34.2<br>7.7	1.00	102	10	3	2	5.6	FVGQVEGAALHPK<br>FVGQVEGAALHPK Deamidated (Q4)<br>GYLASSSPSVTPLWTVQLPFPIIAVATGEDALR	34.1<br>13.7<br>26.2
404	Tb927.4.600	hypothetical protein, conserved	0.99	56	5	2	2	7.6	FVLFEGDRSGGISGLSAQGPAVTR<br>SGGISGLSAQGPAVTR	17.0<br>13.8	0.00	14	1	1	1	7.6	FVLFEGDRSGGISGLSAQGPAVTR	14.3
405	Tb927.1.1580	cytochrome c oxidase assembly factor, putative,electron transport protein SCO1/2, putative	1.00	78	9	2	1	4.7	GLTGTSKEIEQAAR<br>GLTGTSKEIEQAAR Deamidated (Q11)	47.6<br>4.1	0.99	65	2	2	1	4.7	GLTGTSKEIEQAAR<br>GLTGTSKEIEQAAR Deamidated (Q11)	41.9<br>23.3
406	Tb927.6.2560	hypothetical protein, conserved	0.97	38	2	1	1	12.6	LTTLDEKEINPTVSASFSFLPVVTAAIPSSLSDIQLAPR	27.1	1.00	65	5	1	1	12.6	LTTLDEKEINPTVSASFSFLPVVTAAIPSSLSDIQLAPR	52.9
407	Tb927.11.4980	ATP-dependent DEAD/H RNA helicase, putative,ATP- dependent RNA helicase, putative	0.86	33	2	1	1	4.8	NAQNPVALILLPSEELCKQTVEVANALTR Carbamidomethyl (C17)	26.8	1.00	55	1	1	1	2.7	LTAAPILVSTAASLGK	54.6
408	Tb927.11.13510	hypothetical protein, conserved	0.98	63	5	2	2	21.7	SRIPTEYIR<br>VVQGTPNSVEWDPRNPPDTPFFTETTAVER	24.8<br>34.8	0.00	0	0	0	0	0.0		
409	Tb927.11.7780	hypothetical protein, conserved	0.99	87	7	3	3	9.6	LAENFSATSDPR<br>LSAERHGSTLVR<br>SGIGHAELAEEENLCR Carbamidomethyl (C15)	24.9<br>25.2<br>22.6	0.00	0	0	0	0	0.0		
410	Tb927.11.7290	pantothenate kinase subunit, putative	1.00	124	8	3	3	3.1	EGEVAGEDNLLSNWAK<br>LLNDDPFSTVGTLQFAK<br>TLPIAAMWTGTR Oxidation (M7)	32.0<br>48.8<br>32.0	0.00	0	0	0	0	0.0		
411	Tb927.9.9860	hypothetical protein, conserved	1.00	93	3	2	2	3.1	ISPVLGGSTVSQR<br>RVLANEIRR Deamidated (N5)	55.7<br>31.9	0.00	0	0	0	0	0.0		
412	Tb927.11.2510	hypothetical protein, conserved	0.98	81	5	2	2	2.4	RVVGPGVNQVER<br>SRNGLVHADALNEGAVAQR Deamidated (N3)	21.8<br>49.1	0.00	0	0	0	0	0.0		
413	Tb927.7.3630	TPR-repeat-containing chaperone protein DNAJ, putative	0.93	36	57	1	1	5.5	YNMILKILLLVGAAVGAECSGVADDDTTK Deamidated (N2)- Oxidation (M3)- Carbamidomethyl (C19)	20.5	1.00	83	92	2	2	12.3	AECALACGDNQAASEELKYVVQREPQNLDAVALGAR Carbamidomethyl (C3)- Carbamidomethyl (C7)<br>YNMILKILLLVGAAVGAECSGVADDDTTK Deamidated (N2)- Oxidation (M3)- Carbamidomethyl (C19)	49.5<br>20.9
414	Tb927.3.3450	ADP-ribosylation factor-like protein 3A, putative	1.00	70	7	2	1	9.0	ILILGLDNAGKTSILR<br>ILILGLDNAGKTSILR Deamidated (N8)	42.9<br>11.0	0.00	0	0	0	0	0.0		
415	Tb927.10.15000	hypothetical protein, conserved	1.00	51	7	1	1	5.0	ILANLATQGIKDVSSLTIIPK	34.8	0.00	21	2	1	1	5.0	ILANLATQGIKDVSSLTIIPK	13.6
416	Tb927.11.10140	hypothetical protein, conserved	1.00	79	4	1	1	6.7	AGSEPDESTFIVDPANLR	62.6	0.93	37	1	1	1	6.7	AGSEPDESTFIVDPANLR	36.9
417	Tb927.11.3710	hypothetical protein, conserved	1.00	120	7	3	3	7.6	SIAKPPLSK<br>SLILLDEAIKDRDAQLAVK<br>VLSSSTSQVGR	15.3<br>38.9<br>49.3	0.80	31	2	1	1	3.7	SLILLDEAIKDRDAQLAVK	28.7
418	Tb927.10.11300	paraflagellar rod component, putative	0.99	79	7	2	1	19.8	ILGPIKEQTIEKLNDVIPNATTTSR<br>ILGPIKEQTIEKLNDVIPNATTTSR Deamidated (N14)- Deamidated (N19)	32.5<br>36.2	0.00	0	0	0	0	0.0		
419	Tb927.7.4550	60S ribosomal protein-like	1.00	134	5	2	2	21.9	GLGGGGGGAAVAFPVAAAAGAAPSGASATEAEPPKTEK<br>GLGGGGGGAAVAFPVAAAAGAAPSGASATEAEPPKTEKK	58.8<br>52.2	0.00	0	0	0	0	0.0		

420	Tb927.11.5820	hypothetical protein, conserved	1.00	80	3	2	1	3.3	IAQILGSGDR<br>IAQILGSGDR Deamidated (Q3)	57.7<br>19.5	0.00	0	0	0	0	0.0		
421	Tb927.10.3500	RNA-binding protein, putative	1.00	67	6	2	2	3.1	SLYGSTVPIFPTR<br>VLPSVADFVPVIPK	11.3<br>34.8	1.00	51	5	1	1	1.6	VLPSVADFVPVIPK	37.9
422	Tb927.10.9810	hypothetical protein, conserved	0.99	65	6	2	2	8.6	AVENDPANLEFACR Carbamidomethyl (C13)<br>DHWCDRLDLLQR Carbamidomethyl (C4)	31.7<br>16.4	0.00	0	0	0	0	0.0		
423	Tb927.10.15170	hypothetical protein, conserved	1.00	103	3	3	3	5.2	KGQLHVINALSR<br>LLLRPPAEEGSPEVSQPQREENQ<br>RLLLRPPAEEGSPEVSQPQREENQ	16.4<br>47.6<br>38.7	0.00	0	0	0	0	0.0		
424	Tb927.9.2650	hypothetical protein, conserved	1.00	112	12	3	3	9.2	TTPGAASILDPAAYPDLPGCDVVRPLQSGK Carbamidomethyl (C20)<br>TTPGAASILDPAAYPDLPGCDVVRPLQSGKWLK Carbamidomethyl (C20)<br>VGQPHTAVAQFVVAPLVTNYRPR	24.0<br>30.3<br>23.5	0.00	12	1	1	1	3.8	VGQPHTAVAQFVVAPLVTNYRPR	11.9
425	Tb927.11.6440	hypothetical protein, conserved	1.00	64	7	1	1	2.5	VVFEAPASHQR	41.0	0.97	39	1	1	1	2.5	VVFEAPASHQR	39.4
426	Tb927.5.1520	heat shock protein HslVU, ATPase subunit HslU, putative	0.98	49	2	1	1	3.0	ALAGVSDGFREHLR	46.0	0.90	55	3	2	2	8.8	ALAGVSDGFREHLR<br>IKDAIPAVLQEELDKIVDTEDVSAEALR	21.8<br>25.4
427	Tb927.4.4690	hypothetical protein, conserved	1.00	74	4	2	2	10.4	GIVDRQEQFMQLR Oxidation (M10)<br>GIVNRPLNQTNIDILR	26.3<br>40.2	1.00	96	2	2	1	5.8	GIVNRPLNQTNIDILR<br>GIVNRPLNQTNIDILR Deamidated (N11)	48.4<br>47.4
428	Tb927.10.13720	RNA-binding protein, putative	1.00	75	15	2	2	8.2	GVYAGGPLGRPAPYTRPPANPQPPEETLK<br>GVYAGGPLGRPAPYTRPPANPQPPEETLKLR	29.1<br>20.9	0.94	85	6	3	2	8.2	GVYAGGPLGRPAPYTRPPANPQPPEETLK<br>GVYAGGPLGRPAPYTRPPANPQPPEETLK Deamidated (Q22)<br>GVYAGGPLGRPAPYTRPPANPQPPEETLKLR	24.6<br>29.9<br>19.9
429	Tb927.3.2660	hypothetical protein, conserved	0.97	75	4	2	2	5.9	ASSVKQQPLAR<br>GTPPIYVLATAEGVTSSNAPGIYRPSFVHSLR	30.9<br>25.7	1.00	75	1	1	1	4.4	GTPPIYVLATAEGVTSSNAPGIYRPSFVHSLR	75.4
430	Tb927.7.320	hypothetical protein, conserved	1.00	87	3	2	2	13.9	ATYSFPLKQPR<br>SWGTEGINAPLQQK	31.1<br>39.6	0.00	0	0	0	0	0.0		
431	Tb927.9.9060	p21 antigen protein, putative	1.00	53	1	1	1	12.6	NQLLQTDDQKTPIAALDTLIKLER	52.8	0.00	0	0	0	0	0.0		
432	Tb927.11.10660	hypothetical protein, conserved	0.99	41	2	1	1	0.7	AQCLQGIESALAVLGTTEAEMPLASAIQR Carbamidomethyl (C3)- Oxidation (M21)	34.0	0.00	0	0	0	0	0.0		
433	Tb927.11.14090	hypothetical protein, conserved	0.97	45	4	1	1	2.5	KVPFAGGTVSAK	30.7	0.00	17	1	1	1	2.5	KVPFAGGTVSAK	16.7
434	Tb927.9.8740	RNA-binding protein	1.00	114	4	2	1	4.9	GGFDAQKQPQQLQIGR<br>GGFDAQKQPQQLQIGR Deamidated (Q11)	53.5<br>49.6	0.00	0	0	0	0	0.0		
435	Tb927.7.7500	thymine-7-hydroxylase, putative	0.00	14	2	1	1	5.0	TWGFFHVVGHPISPER	11.4	1.00	122	2	2	2	15.0	EGSRHPLVCGEHTDYGIITLLYQDSVGGLQLR Carbamidomethyl (C9)<br>TWGFFHVVGHPISPER	79.6<br>41.9
436	Tb927.9.8160	chaperone protein DNAj, putative	1.00	75	10	1	1	4.7	LALILHPDKTDGTTTEQFTR	37.8	1.00	63	11	1	1	4.7	LALILHPDKTDGTTTEQFTR	38.6
437	Tb927.2.100	retrotransposon hot spot protein 1 (RHS1), putative	0.99	102	9	3	3	5.6	GRLDAAVIAAK<br>NQIPQSVLQVLQEPR<br>RPRDENVPPPPAAAQPPQIR	26.1<br>17.9<br>31.5	0.00	0	0	0	0	0.0		
438	Tb927.11.10780	hypothetical protein, conserved	1.00	63	3	1	1	5.4	MGPSEYDELENIVER Oxidation (M1)	40.9	0.00	0	0	0	0	0.0		
439	Tb927.2.2970	mitochondrial carrier protein, putative	0.00	22	2	1	1	7.7	ATAAGIAGVMEIGLFHPFDTVAKR Oxidation (M10)	11.4	0.99	44	1	1	1	5.5	VQNKDPGSTLTGLVVFR	44.4
440	Tb927.10.4050	serine palmitoyltransferase, putative	1.00	85	6	1	1	4.7	GAVVANKAVTEAESCDEAYNKQGSVR Carbamidomethyl (C15)	68.7	0.00	0	0	0	0	0.0		
441	Tb04.24M18.150	hypothetical protein, conserved	0.99	57	5	2	2	29.6	DLRDAEHFLAVLPIIDGEDHAHTESAFSQLPK<br>VLIVPATTPAIKDSAR	11.8<br>33.8	0.00	0	0	0	0	0.0		
442	Tb927.6.4990	ATP synthase, epsilon chain, putative	1.00	58	6	1	1	7.7	ISVIEAVIAALKHH	36.5	0.00	0	0	0	0	0.0		
443	Tb927.10.9830	hypothetical protein, conserved	1.00	50	3	1	1	7.6	AANIEQGVAFVK	46.9	0.00	0	0	0	0	0.0		
444	Tb927.10.10140	paraflagellar rod component, putative	0.00	23	3	2	1	8.8	AAESEHVAAILQFLADEDEFRTETEKNNR<br>AAESEHVAAILQFLADEDEFRTETEKNNR Deamidated (N28)	8.0<br>11.6	0.99	113	3	3	1	8.8	AAESEHVAAILQFLADEDEFRTETEKNNR<br>AAESEHVAAILQFLADEDEFRTETEKNNR Deamidated (N28)<br>AAESEHVAAILQFLADEDEFRTETEKNNR Deamidated (Q12)	33.0<br>45.4<br>34.4
445	Tb927.6.1570	2-hydroxy-3-oxopropionate reductase, putative	1.00	64	3	1	1	3.3	AGFPLVVFNR	47.9	0.00	0	0	0	0	0.0		
446	Tb927.8.6240	hypothetical protein, conserved	0.70	37	6	2	2	7.1	IVRPAESFKPR<br>YKQEPLPK	18.2<br>8.1	0.96	38	3	1	1	4.1	IVRPAESFKPR	27.3
447	Tb927.11.1670	cysteine desulfurase	0.99	44	1	1	1	3.2	ALGIDAENAHTSIR	44.3	0.00	6	1	1	1	3.2	ALGIDAENAHTSIR	6.5
448	Tb927.10.1100	60S ribosomal protein L9, putative	1.00	62	2	1	1	5.3	FLDGIYVQTK	55.0	0.00	0	0	0	0	0.0		
449	Tb927.3.2880	hypothetical protein, conserved	1.00	65	5	2	2	15.4	WTGCSVEQLEELSR Carbamidomethyl (C4)<br>WTGCSVEQLEELSRVV Carbamidomethyl (C4)	11.6<br>36.8	0.00	12	1	1	1	15.4	WTGCSVEQLEELSRVV Carbamidomethyl (C4)	12.1
450	Tb927.11.14700	hypothetical protein, conserved	0.94	37	1	1	1	2.6	SAAQVTPIVLHSGR	36.9	0.96	35	1	1	1	5.1	SQDVSTVAADGESTVPDHGPLRPTGLWK	35.5
451	Tb927.10.7350	hypothetical protein, conserved	0.98	55	5	2	2	2.2	TITQDMGAGLLGR Oxidation (M6)<br>VLSAAAAPALHSGVADHNTMSLPLKK Oxidation (M20)	30.3<br>11.6	0.00	0	0	0	0	0.0		
452	Tb927.2.270	retrotransposon hot spot protein (RHS, pseudogene), putative,retrotransposon hot spot protein 3 (RHS3), frameshift	1.00	71	4	2	2	6.0	EFVGPVAATKLDR<br>RPRDENVPPPPAAAAAQPPQIR	26.9<br>42.2	0.00	0	0	0	0	0.0		
453	Tb927.10.11390	60S ribosomal protein L6, putative	0.00	13	1	1	1	9.4	VDLTGVDTSAITPEIFKR	12.7	1.00	68	2	2	2	21.9	GTVSDERAQLQNAIDTALIQAIKK<br>VDLTGVDTSAITPEIFKR	11.0<br>57.1
454	Tb927.8.3290	DNA polymerase zeta catalytic subunit, putative	0.98	65	8	2	1	0.8	GGSGLLGNQSARGAK Deamidated (Q9)<br>GGSGLLGNQSARGAK Deamidated (Q9)- Deamidated (N8)	24.3<br>23.8	0.00	15	1	1	1	0.8	GGSGLLGNQSARGAK Deamidated (Q9)	14.8
455	Tb927.10.15360	hypothetical protein, conserved	1.00	58	4	1	1	0.7	NAVVNNAPIQQR	50.9	0.00	0	0	0	0	0.0		
456	Tb927.10.6640	COP-coated vesicle membrane protein erv25 precursor, putative,ER--golgi transport protein erv25 precursor, putative	0.00	16	1	1	1	11.1	FLLQNTQPICFVEEVGEDTRFLTGVYTR Carbamidomethyl (C10)	15.7	1.00	103	4	2	1	11.1	FLLQNTQPICFVEEVGEDTRFLTGVYTR Carbamidomethyl (C10)<br>FLLQNTQPICFVEEVGEDTRFLTGVYTR Deamidated (N5)- Carbamidomethyl (C10)	43.4<br>35.1
457	Tb927.10.4040	3-keto-dihydrosphingosine reductase	1.00	62	3	1	1	5.8	FQVISPPDVYTPGFEQENIR	53.6	0.96	56	2	2	1	5.8	FQVISPPDVYTPGFEQENIR<br>FQVISPPDVYTPGFEQENIR Deamidated (N18)	15.8<br>40.4
458	Tb927.10.4130	NADH-ubiquinone oxidoreductase complex I subunit, putative,NDUFA5/B13 subunit, putative	1.00	53	2	1	1	3.4	FAVDKDLQR	41.6	0.00	0	0	0	0	0.0		
459	Tb927.3.1410	cytochrome oxidase subunit VII	1.00	71	11	2	2	11.5	PRPFGVWAPATTLAEYR<br>PRPFGVWAPATTLAEYRAR	19.8<br>21.0	0.00	0	0	0	0	0.0		
460	Tb927.11.4650	hypothetical protein, conserved	1.00	60	3	2	1	2.2	SNYDGTSLPAWPAPGKKPTYPAALSELRLPQPR<br>SNYDGTSLPAWPAPGKKPTYPAALSELRLPQPR Deamidated (N2)	38.5<br>9.7	0.00	15	1	1	1	2.2	SNYDGTSLPAWPAPGKKPTYPAALSELRLPQPR	14.7
461	Tb927.11.6230	pretranslocation protein, alpha subunit, putative,SEC61-like (pretranslocation process) protein, putative	1.00	100	4	2	1	3.3	MSPILAFLPEVATPLR<br>MSPILAFLPEVATPLR Oxidation (M1)	40.6<br>44.1	0.37	24	1	1	1	3.3	MSPILAFLPEVATPLR	24.1
462	Tb927.8.2020	agmatinase, putative	1.00	56	3	1	1	4.5	LGPVALFGYGAGMPR Oxidation (M13)	51.5	0.00	0	0	0	0	0.0		

463	Tb927.2.5270	dynein heavy chain, putative	1.00	47	3	1	1	0.6	STLLAQDGGASAVNPDAEVEKDGKR	30.6	0.00	0	0	0	0	0.0		
464	Tb927.10.10010	60S acidic ribosomal protein, putative	1.00	99	7	2	2	9.3	RATIVPLTK<br>TGQVATCPLLLR Carbamidomethyl (C7)	26.7<br>57.4	0.94	37	1	1	1	5.3	TGQVATCPLLLR Carbamidomethyl (C7)	36.6
465	Tb927.4.3890	ATP-dependent RNA helicase, putative	1.00	68	5	2	2	3.0	LAALFPEKQVK<br>SSAGRPPAAETTSTKPEVPKPR	18.7<br>32.0	0.00	0	0	0	0	0.0		
466	Tb927.11.10090	hypothetical protein, conserved	0.99	67	4	2	2	5.3	LHIAPQIIPAGR<br>NGRGEGDAVR	34.4<br>29.2	0.00	0	0	0	0	0.0		
467	Tb927.4.3070	hypothetical protein, conserved	0.99	71	6	2	2	8.3	AATAVVIGMNR Oxidation (M9)<br>VATILLPGDLQHTSTTEEDKAQQYVR	40.0<br>18.5	0.00	20	1	1	1	5.8	VATILLPGDLQHTSTTEEDKAQQYVR	19.9
468	Tb927.7.6350	NADH-ubiquinone oxidoreductase, mitochondrial, putative	0.99	46	1	1	1	4.4	SAIIPLLHLAQR	46.0	0.00	0	0	0	0	0.0		
469	Tb927.8.4930	hypothetical protein, conserved	1.00	63	4	1	1	5.3	VTALPTSHAHPSGASR	45.4	0.98	43	2	1	1	5.3	VTALPTSHAHPSGASR	38.8
470	Tb927.1.90	retrotransposon hot spot protein (RHS, pseudogene), putative	0.93	46	2	2	2	6.3	HIVIGTPGIGK<br>RPRDENVPPPPPAAAAAAQPPQIR<br>VRDVLLEDALLQR	14.2<br>37.5<br>8.4	1.00	49	2	1	1	2.2	HIVIGTPGIGK<br>VRDVLLEDALLQR	25.0<br>48.7
471	Tb927.9.6920	hypothetical protein, conserved	0.97	36	2	1	1	7.2	EGVKPFPDAPLGGAAVEPHALPPKPR	36.2	0.00	8	1	1	1	7.5	REGVKPFPDAPLGGAAVEPHALPPKPR	7.9
472	Tb927.6.4320	hypothetical protein, conserved	0.97	38	1	1	1	2.5	AAILENAASR	37.6	0.00	0	0	0	0	0.0		
473	Tb927.7.6260	hypothetical protein, conserved	0.00	14	1	1	1	4.4	AAYGLWVVANEIIR	14.4	0.98	42	1	1	1	4.4	AAYGLWVVANEIIR Deamidated (N10)	42.2
474	Tb927.11.4920	hypothetical protein, conserved	0.99	63	5	2	2	12.9	KAVTTADAPLPSCTFNTTVSR Carbamidomethyl (C13)<br>SRFPGPIPVWTSTK	15.9<br>34.6	0.00	0	0	0	0	0.0		
475	Tb927.11.15850	hypothetical protein, conserved	1.00	56	1	1	1	4.4	TFQPELLGQAWSVPNKPLLFWGTGVSAVQQALR	56.4	0.00	0	0	0	0	0.0		
476	Tb927.10.510	hypothetical protein, conserved	0.99	54	5	2	2	11.4	DGGLPVTLEVPVDGR<br>LTAEVVLIPAFVYITHSDAGILR	34.0<br>7.2	1.00	83	3	2	2	11.4	DGGLPVTLEVPVDGR<br>LTAEVVLIPAFVYITHSDAGILR	46.1<br>36.6
477	Tb927.9.10520	hypothetical protein, conserved	0.98	64	6	2	2	9.9	ALAVAESRNEVR<br>TQDYIQGFHQHLK	18.8<br>29.8	0.91	35	3	1	1	4.7	ALAVAESRNEVR	29.4
478	Tb927.10.15710	mitochondrial carrier protein, putative	1.00	71	4	1	1	5.6	HAPLVDVVAGGLGSAAAK	35.1	0.00	0	0	0	0	0.0		
479	Tb927.4.3840	nucleolar protein, putative	1.00	74	3	2	2	4.6	NTGVIFANDVSEPR<br>TKSLNANLQR	50.2<br>21.4	0.00	0	0	0	0	0.0		
480	Tb927.11.13890	hypothetical protein, conserved	1.00	65	3	1	1	5.6	IAPGVTVEETDVQSR	54.9	0.00	0	0	0	0	0.0		
481	Tb927.7.5790	protein disulfide isomerase, putative	1.00	50	2	1	1	7.4	GYPTLLLFAR	30.8	0.00	0	0	0	0	0.0		
482	Tb927.7.7260	kinesin, putative	0.99	73	17	2	1	1.1	DALRLQLDPPQ Deamidated (Q6)<br>DALRLQLDPPQ Deamidated (Q6)- Deamidated (Q11)	30.0<br>25.6	0.92	56	9	2	1	1.1	DALRLQLDPPQ Deamidated (Q6)<br>DALRLQLDPPQ Deamidated (Q6)- Deamidated (Q11)	17.5<br>29.7
483	Tb927.4.1280	hypothetical protein, conserved	0.96	40	2	1	1	4.5	AIIEATASETHPAAAQLR	24.7	0.00	0	0	0	0	0.0		
484	Tb927.3.2080	hypothetical protein, conserved	0.99	67	5	2	2	5.8	AGRDPTELGL<br>ELAEKAGRDPTELGL	32.5<br>24.6	0.00	0	0	0	0	0.0		
485	Tb927.11.7520	hypothetical protein, conserved	0.99	46	2	1	1	3.3	EILDTVTLLQSR	33.2	0.00	0	0	0	0	0.0		
486	Tb927.5.3800	glutamine hydrolysing (not ammonia-dependent) carbomoyl phosphate synthase, putative	0.97	39	2	1	1	0.5	ILVIDMGVK Oxidation (M6)	32.0	0.00	0	0	0	0	0.0		
487	Tb927.11.13310	hypothetical protein, conserved	1.00	56	2	1	1	6.0	SVVNSGSGGGAPASPHTR	30.6	0.00	10	1	1	1	6.0	SVVNSGSGGGAPASPHTR	10.4
488	Tb927.7.7210	hypothetical protein, conserved	0.00	9	1	1	1	2.7	ACGPLTDELADALVTDFWK Carbamidomethyl (C2)	8.7	1.00	84	4	1	1	2.7	ACGPLTDELADALVTDFWK Carbamidomethyl (C2)	34.6
489	Tb927.11.16750	hypothetical protein, conserved	1.00	74	9	2	2	15.4	LGQQAMTPVAQTGYKPMPGQSS Oxidation (M6)- Oxidation (M17)<br>QGGAPLGVTDVNLR	26.2<br>22.7	0.00	17	1	1	1	6.0	QGGAPLGVTDVNLR	17.2
490	Tb927.10.830	adenylate kinase, putative	1.00	84	3	1	1	5.0	AVTADVLKTVESR	67.9	0.00	0	0	0	0	0.0		
491	Tb927.7.6990	hypothetical protein, conserved	0.97	64	6	2	2	9.0	ELCEGPLDLKAGER Carbamidomethyl (C3)<br>KGPPVAHSLPVYK	28.4<br>27.7	0.00	0	0	0	0	0.0		
492	Tb927.11.5250	hypothetical protein, conserved	1.00	82	5	2	2	1.5	RGDVGWELLR	41.4	0.00	0	0	0	0	0.0		
493	Tb927.10.8980	hypothetical protein, conserved	0.97	39	5	1	1	7.1	TPLPVGNAASAR	28.8	0.00	0	0	0	0	0.0		
494	Tb927.11.12040	hypothetical protein, conserved	0.96	37	4	1	1	13.2	TGRLPMPDDPLFNPIHMANFR Oxidation (M6)- Oxidation (M17)	31.3	0.00	0	0	0	0	0.0		
495	Tb927.11.540	ABC transporter, putative	0.70	30	1	1	1	4.1	SPGILILDEATSALDSESEIVVHEAVSR	30.0	1.00	80	4	1	1	4.1	SPGILILDEATSALDSESEIVVHEAVSR	60.9
496	Tb927.11.6430	hypothetical protein, conserved	0.98	65	4	2	2	2.9	AVSPVGVTGSSINGAGVSVPLPR<br>LAPLSASTLRPAAK	32.7<br>17.6	0.00	11	2	1	1	1.1	LAPLSASTLRPAAK	9.1
497	Tb927.1.3450	hypothetical protein, conserved	1.00	79	2	1	1	1.6	ALVALQGLCNDAR Carbamidomethyl (C9)	73.9	0.00	0	0	0	0	0.0		
498	Tb927.8.6640	hypothetical protein, conserved	1.00	78	6	1	1	3.0	STAASTAQGLLNPLTAAK	54.3	0.00	0	0	0	0	0.0		
499	Tb927.11.610	hypothetical protein, conserved	1.00	75	2	2	2	10.7	FLPGYAGHIPTYCSK Carbamidomethyl (C13)<br>FSPESPHEVAPALPPVLPLTQRR	36.1<br>39.2	0.00	0	0	0	0	0.0		
500	Tb927.8.1870	Golgi/lysosome glycoprotein 1	1.00	44	4	1	1	5.0	FVSDTEDCDASAAGGVPETLALEPEDRFGPK Carbamidomethyl (C8)	37.9	0.00	9	1	1	1	5.0	FVSDTEDCDASAAGGVPETLALEPEDRFGPK Carbamidomethyl (C8)	9.2
501	Tb927.8.6050	hypothetical protein, conserved	1.00	77	4	1	1	11.1	AVISPQEKPLTSSSSGEALGGSGNEVKR	54.5	0.00	0	0	0	0	0.0		
502	Tb927.6.2870	hypothetical protein, conserved	0.99	67	3	2	1	2.8	GFINAEQTTDASR<br>GFINAEQTTDASR Deamidated (N4)	18.6<br>34.0	0.97	41	3	1	1	2.8	GFINAEQTTDASR	29.2
503	Tb927.6.630	hypothetical protein, conserved	0.96	38	1	1	1	3.6	TGAEALFFALDPASNPGEEKLPLIGR	37.6	1.00	66	3	1	1	3.6	TGAEALFFALDPASNPGEEKLPLIGR	44.3
504	Tb927.7.4290	hypothetical protein, conserved	1.00	52	4	1	1	4.0	AAAQAAAEAAKR	42.4	0.00	0	0	0	0	0.0		
505	Tb927.5.4040	hypothetical protein, conserved	0.80	29	4	1	1	2.3	RLVEPQLRPQAATDALLAR	26.8	0.99	38	4	1	1	2.3	RLVEPQLRPQAATDALLAR	20.4
506	Tb927.10.12960	ras-related protein rab-5,small GTPase, putative	1.00	76	2	1	1	8.7	LLLEHGLGANSGPGSLSGPR	40.5	1.00	72	2	1	1	8.7	LLLEHGLGANSGPGSLSGPR	55.7
507	Tb927.10.2550	malate dehydrogenase-related	1.00	69	7	1	1	6.2	AAPHAIVGIASGPVNSLVPLAR	57.6	1.00	61	1	1	1	6.2	AAPHAIVGIASGPVNSLVPLAR	60.8
508	Tb927.3.1820	hypothetical protein, conserved	0.29	24	1	1	1	7.8	ANPSDGVLYVAATPLAYQK	24.2	1.00	59	4	1	1	7.8	ANPSDGVLYVAATPLAYQK	32.6
509	Tb927.6.3670	paraflagellar rod component, putative	1.00	50	3	1	1	0.6	AVPIAAHPAFDTLPAPFLR	42.3	0.00	16	1	1	1	0.6	AVPIAAHPAFDTLPAPFLR	15.7
510	Tb927.5.1680	hypothetical protein, conserved	1.00	72	9	1	1	1.3	AAAAAAAAEVESGIAGVEETLRR	43.0	0.00	0	0	0	0	0.0		
511	Tb927.8.4450	RNA-binding protein, putative	0.98	43	1	1	1	4.7	STVVSSSSVATVPASNVSGK	42.9	0.00	0	0	0	0	0.0		

512	Tb927.6.4080	hypothetical protein, conserved	0.99	44	1	1	1	6.8	TILGPQGGFAGEER	44.0	0.00	0	0	0	0	0.0		
513	Tb927.6.1870	eukaryotic translation initiation factor 4e, putative	1.00	71	3	1	1	3.0	AATNAAALAVKEK	51.7	0.00	0	0	0	0	0.0		
514	Tb927.11.6660	hypothetical protein, conserved	1.00	64	4	1	1	2.3	VAAASVGGATPVTSFQTPTER	51.7	0.00	0	0	0	0	0.0		
515	Tb927.9.14420	cyclophilin-like protein, putative	1.00	51	3	1	1	3.7	RLPLTLIEEVPLPR	37.9	0.00	9	1	1	1	3.7	RLPLTLIEEVPLPR	9.2
516	Tb927.10.9820	mitochondrial intermediate peptidase, putative,metallo-peptidase, Clan MA(E) Family M3	0.98	43	2	1	1	2.8	SACVTDVEEIPTLSFLSSK Carbamidomethyl (C3)	41.1	0.00	17	1	1	1	2.8	SACVTDVEEIPTLSFLSSK Carbamidomethyl (C3)	16.5
517	Tb927.11.10960	hypothetical protein, conserved	1.00	67	3	1	1	3.4	SGGLGEGELGSSPHHLSLEQYR	41.4	0.00	0	0	0	0	0.0		
518	Tb927.11.8800	hypothetical protein, conserved	1.00	57	2	1	1	5.1	TIVLIGKPEDVVYR	55.3	0.00	0	0	0	0	0.0		
519	Tb927.5.2530	hypothetical protein, conserved	0.98	39	2	1	1	1.1	PPPTSVASTAR	24.4	0.99	44	3	1	1	1.1	PPPTSVASTAR	33.1
520	Tb927.5.3390	ADG1, pseudogene	1.00	65	4	1	1	9.3	RLELQNVEQTR	58.9	0.00	0	0	0	0	0.0		
521	Tb927.7.5680	deoxyribose-phosphate aldolase, putative	1.00	64	2	1	1	5.0	QGFTGKLNDALLER	43.9	0.00	0	0	0	0	0.0		
522	Tb927.10.6610	chaperone protein DNAj, putative	1.00	58	2	1	1	4.4	NVVQQQQESLAR	54.7	0.00	0	0	0	0	0.0		
523	Tb927.11.9560	oxidoreductase, putative	1.00	63	1	1	1	2.9	LVVCLGSIESPALLLR Carbamidomethyl (C4)	63.2	0.00	0	0	0	0	0.0		
524	Tb927.10.3640	hypothetical protein, conserved	1.00	62	2	1	1	3.9	YATGGKPITAK	56.6	0.76	41	2	1	1	3.9	YATGGKPITAK	36.4
525	Tb927.6.4070	hypothetical protein, conserved	1.00	56	2	1	1	7.7	DYPQVFQGSPSTSYPIATAH	50.8	0.00	0	0	0	0	0.0		
526	Tb927.11.7100	hypothetical protein, conserved	1.00	48	2	1	1	4.9	VLSSSHIGAR	40.6	0.00	0	0	0	0	0.0		
527	Tb927.11.1680	vesicular-fusion protein SEC18, putative	1.00	44	2	1	1	1.0	AAVSHALLR	25.3	0.53	31	1	1	1	1.0	AAVSHALLR	30.6
528	Tb927.8.5860	50S ribosomal protein L17, putative	1.00	60	1	1	1	3.7	ILADHVVELAK	59.9	0.00	0	0	0	0	0.0		
529	Tb927.10.14770	protein kinase, putative	0.99	47	2	1	1	2.2	LANSSLPVSHTSTR	30.5	0.00	0	0	0	0	0.0		
530	Tb927.7.4460	hypothetical protein, conserved	1.00	60	4	1	1	2.0	SQTIPGAADTPIVAAAR	42.2	0.00	0	0	0	0	0.0		
531	Tb927.2.2160	paraflagellar rod component, putative	0.59	28	2	1	1	5.2	SSSTYVPDAVFENLIER	23.4	0.99	47	1	1	1	5.2	SSSTYVPDAVFENLIER	46.6
532	Tb927.10.360	hypothetical protein, conserved	1.00	56	2	1	1	5.0	VVPDVAGQIATFEAR	44.2	0.00	0	0	0	0	0.0		
533	Tb927.7.1090	hypothetical protein, conserved	1.00	56	2	1	1	1.8	FATSATNQGGLVKPSGPPSSVAAAAQR	55.6	0.00	0	0	0	0	0.0		
534	Tb927.11.8030	hypothetical protein, conserved	1.00	56	1	1	1	2.6	ILDYSHVGLHR	55.7	0.00	0	0	0	0	0.0		
535	Tb927.8.3050	hypothetical protein, conserved	1.00	55	2	1	1	2.1	VGLGTGAQQLAR	35.6	0.00	0	0	0	0	0.0		
536	Tb927.11.10080	hypothetical protein, conserved	1.00	55	1	1	1	13.2	FRVQPLPSLHANSLADDTPLVTTTR	55.1	0.00	0	0	0	0	0.0		
537	Tb927.11.10210	hypothetical protein, conserved	0.99	45	2	1	1	1.0	LISLASSAGR	40.0	0.00	0	0	0	0	0.0		
538	Tb927.6.5090	hypothetical protein, conserved	1.00	54	2	1	1	1.7	AALGTEGTQVLSPR	44.1	0.00	0	0	0	0	0.0		
539	Tb927.11.460	hypothetical protein, conserved,predicted WD40 repeat protein	0.99	41	5	1	1	1.0	SLVVANKPR	31.7	0.00	0	0	0	0	0.0		
540	Tb927.11.11250	cytosolic malate dehydrogenase	0.99	47	1	1	1	6.7	VAVTGAAGQIGYSLLPLIAAGR	47.0	0.26	24	1	1	1	6.7	VAVTGAAGQIGYSLLPLIAAGR	23.5
541	Tb927.6.1550	hypothetical protein, conserved,leucine-rich repeat protein (LRRP), putative	0.99	45	4	1	1	2.6	ALGAHPTLR	33.9	0.00	0	0	0	0	0.0		
542	Tb927.7.540	chaperone protein DNAj, putative	1.00	46	2	1	1	5.3	AAALQNHPDHAPKEAEAQNAAQER	45.2	0.00	0	0	0	0	0.0		
543	Tb927.7.5320	hypothetical protein, conserved	1.00	53	2	1	1	2.0	EAPLVLVSTVAEKLR	47.4	0.00	0	0	0	0	0.0		
544	Tb927.10.14720	peroxin 13	1.00	53	4	1	1	2.6	KGLVPGNFLR	41.4	0.00	0	0	0	0	0.0		
545	Tb927.4.1600	hypothetical protein, conserved	1.00	52	1	1	1	5.1	VILLVGMPGSGK Oxidation (M7)	51.6	0.00	0	0	0	0	0.0		
546	Tb927.9.3370	thioredoxin	0.98	44	1	1	1	10.3	VLQLPTFIIAR	44.0	0.00	0	0	0	0	0.0		
547	Tb927.10.10800	hypothetical protein, conserved	1.00	46	2	1	1	7.7	GVCNVYHLSQVSSHVGSLLGQTFPTAVPLTSPTPV Carbamidomethyl (C3)	34.8	0.44	27	1	1	1	7.7	GVCNVYHLSQVSSHVGSLLGQTFPTAVPLTSPTPV Carbamidomethyl (C3)	26.9
548	Tb927.7.3810	hypothetical protein, conserved	1.00	49	3	1	1	3.7	ALGFAADSKPAAAR	38.8	0.00	0	0	0	0	0.0		
549	Tb927.10.7700	ABC transporter, putative	1.00	48	2	1	1	2.1	VILHNLTGTALPSR	32.6	0.00	0	0	0	0	0.0		
550	Tb927.8.8120	hypothetical protein, conserved	0.99	48	1	1	1	6.3	GGGPSAVCEQENLCTAR Carbamidomethyl (C8)- Carbamidomethyl (C14)	47.6	0.49	27	1	1	1	6.3	GGGPSAVCEQENLCTAR Carbamidomethyl (C8)- Carbamidomethyl (C14)	26.9
551	Tb927.7.2670	hypothetical protein, conserved,zinc finger protein family member, putative	0.99	44	2	1	1	2.2	FVQGHPGVPR	40.0	0.00	0	0	0	0	0.0		
552	Tb927.9.10070	hypothetical protein, conserved	1.00	48	1	1	1	4.4	CVAALEEPVVFFKN Carbamidomethyl (C1)	48.3	0.00	0	0	0	0	0.0		
553	Tb927.10.15950	TFIID-like protein, putative	1.00	48	1	1	1	5.3	AAGASGVYVDAAPK	48.0	0.00	0	0	0	0	0.0		
554	Tb927.5.1160	hypothetical protein, conserved	1.00	48	4	1	1	9.2	NFLEDRGLTCPGCQEKADFVR Carbamidomethyl (C10)- Carbamidomethyl (C13)	42.5	0.00	0	0	0	0	0.0		
555	Tb927.6.2720	calcium-binding protein, putative	1.00	48	1	1	1	3.1	VSGEAIDALKR	48.0	0.00	0	0	0	0	0.0		
556	Tb927.11.15240	small GTPase, putative,ras-related protein rab-2a, putative	1.00	46	1	1	1	14.6	TAAMIYDNVESGVLDAGAVSGKQGTGPGVAR Oxidation (M4)	46.3	0.00	0	0	0	0	0.0		
557	Tb927.10.600	hypothetical protein, conserved	0.00	0	0	0	0	0.0			1.00	90	3	2	2	7.2	APSHGALTLDDVPHQEAVR<br>FAGEANEESTLDYLLENFGR	29.6<br>46.7
558	Tb927.9.15060	rRNA processing protein, putative	0.00	0	0	0	0	0.0			0.99	64	2	2	1	13.9	MALTSTKPLPSDLNADDDPKREEAFIQQALLSVVR<br>MALTSTKPLPSDLNADDDPKREEAFIQQALLSVVR Oxidation (M1)	29.1<br>35.0
559	Tb927.9.5040	cAMP-specific phosphodiesterase	0.00	0	0	0	0	0.0			1.00	136	5	3	3	8.3	EIRFPCGQGIAGTVAASGVGENIQDAYQDPR Carbamidomethyl (C6)<br>GQIGFIDFVAAPFFQK Deamidated (Q15)<br>TYYHNPNDDVLADPVHDTAALIDETTAVGK	52.7<br>30.0<br>35.0
560	Tb927.8.4500	eukaryotic translation initation factor 4 gamma, putative	0.00	0	0	0	0	0.0			1.00	108	4	2	2	5.1	IQFLLLNTLETIDNNWVPR<br>YSPPLLSGTVVDESDDILR	36.6<br>37.2
561	Tb927.9.5280	hypothetical protein, conserved	0.00	0	0	0	0	0.0			1.00	73	4	1	1	18.3	LSPATESPGSGTAISDGEPSTDVEGETTVDDEIAQLEAELAALEREGGGK	42.8
562	Tb927.2.4090	hypothetical protein, conserved	0.00	0	0	0	0	0.0			1.00	45	3	1	1	14.1	KFSFANFQVPVQFVDISSFVGPEDQVYNVEPVREFATS	40.0
563	Tb927.8.4890	endoplasmic reticulum oxidoreductin, putative,pol-associated gene 1	0.00	0	0	0	0	0.0			1.00	59	1	1	1	7.7	GFEDEIKPIDPDRDATYVDLLQNPEANTGYSGPK	58.6
564	Tb927.10.790	vesicle-associated membrane protein, putative,synaptobrevin, putative	0.00	0	0	0	0	0.0			1.00	56	1	1	1	6.7	KAVDEVKEIALDNVER	56.0
565	Tb927.11.3240	T-complex protein 1, zeta subunit, putative	0.00	0	0	0	0	0.0			1.00	113	5	3	3	5.7	LDTGSTVDPLAAGILDNVLVK<br>LDTGSTVDPLAAGILDNVLVKR<br>YIQDGLHPR	39.8<br>35.6<br>34.7
566	Tb927.11.10170	hypothetical protein, conserved	0.00	0	0	0	0	0.0			1.00	49	5	1	1	12.2	RGPSGLGNNVNELIDAWKEFDVSPLR	28.7

567	Tb927.10.10880	ATP-binding cassette protein, putative,ABC transporter, putative,ATPase, putative	0.00	0	0	0	0	0.0	0.98	66	2	2	2	6.9	ELPVQSNLELLLVEQEQEFHESEVSAVQAVLQSHKK Deamidated (Q28)	35.2
															GGVLVVTHDAR	30.9
568	Tb927.11.2570	hypothetical protein, conserved	0.00	0	0	0	0	0.0	1.00	93	1	1	1	6.1	LVAAAAASAAAAAAAAASESQQKEQEYVGVSAVGK	93.3
569	Tb927.8.3150	T-complex protein 1, gamma subunit, putative	0.00	0	0	0	0	0.0	1.00	76	5	1	1	5.4	AQDEEVGDGTTSVIILTGEILSLSKPLLER	48.2
570	Tb927.11.11160	sodium/sulphate symporter, putative	0.00	0	0	0	0	0.0	1.00	54	2	1	1	2.2	LIGDSQKQEIEEDTPR	52.9
571	Tb927.10.14030	hypothetical protein, conserved	0.00	0	0	0	0	0.0	1.00	68	1	1	1	3.3	SGLLELAGLVEGKPK	68.2
572	Tb927.11.2120	hypothetical protein, conserved	0.00	0	0	0	0	0.0	1.00	58	2	1	1	2.8	VSDVDASLFGPSGVLGDPAVSK	45.5
573	Tb927.11.14960	pumilio/PUF RNA binding protein 7, putative	0.00	0	0	0	0	0.0	0.99	46	5	1	1	2.3	FLPYLGQLALHEFGQR	41.2
574	Tb927.6.4970	serine/arginine-rich protein specific kinase SRPK, putative,protein kinase, putative	0.00	0	0	0	0	0.0	1.00	55	2	1	1	2.8	KAEKVEGLAVKPGTTVAQAR	39.2
575	Tb927.10.14390	hypothetical protein, conserved	0.00	0	0	0	0	0.0	1.00	52	3	1	1	4.7	GGADKVTIASLPITEKEALLTYLQSK	37.9
576	Tb927.9.9940	PACRGB,flagellar component	0.00	0	0	0	0	0.0	1.00	52	1	1	1	9.0	RQNISDLVNETLHLLEQYGGEDAYINIK	51.9
577	Tb927.10.4430	pumillo RNA binding protein PUF1	0.00	0	0	0	0	0.0	1.00	51	2	1	1	4.9	LLTDSFANYVIQTAIVTSTEEKQFTQLR	30.0
578	Tb927.6.5210	variant surface glycoprotein (VSG, pseudogene), putative,variant surface glycoprotein, frameshift	0.00	0	0	0	0	0.0	0.99	51	1	1	1	2.5	AKMAAIGAAAAAK	51.0
579	Tb927.8.1240	electron transfer flavoprotein-ubiquinone oxidoreductase, putative	0.00	0	0	0	0	0.0	1.00	49	1	1	1	5.7	TLVEGGLVSLPQLHFPGGVLVGDCAGFLNLPK Carbamidomethyl (C24)	49.0
580	Tb927.10.12260	cytosolic nonspecific dipeptidase, putative,peptidase (M20/M25/M40 family), putative	0.00	0	0	0	0	0.0	0.99	48	2	1	1	4.4	AALTVVGDNLPDTATAGNVNR	28.1
