# Supplementary material for: Proteomic Analysis of the Acidocalcisome, an Organelle Conserved from Bacteria to Human Cells
Source: PLoS Pathog. 2014 Dec 11;10(12):e1004555. doi: 10.1371/journal.ppat.1004555 (PMC4263762; doi:10.1371/journal.ppat.1004555)
Supplement: S4 Table — Predicted subcellular locations for high-confidence protein groups (protein probability: 1% false discovery rate, p≥0.95,) identified in the T. brucei acidocalcisome fractions (ACCS1 and ACCS2) from our prediction servers using non-plant based algorithms. Individual protein predictions are based upon agreement between at least two analysis packages. Consensus for identifications with multiple protein hits (see Methods for explanation of protein grouping based on degenerate peptide fingerprints) is given when prediction for a compartment agrees among two of more members of the group. S, secreted. C, cytosol. M, mitochondrion. N, nucleus. PM, plasma membrane. G, Golgi complex. ER, endoplasmic reticulum. P, peroxisome. L, lysosome. CYKS, cytoskeleton. Threshold probabilities and confidences used to screen our predictions with poor reliability: targetP (RC = 1). pTarget ≥80%, SLP-LOCAL ≥2, WoLfPsort ≥∼80%. WoLFPSORT thresholds for each subcellular location were derived from empirical prediction confidence statistics (wolfpsort.org/empiricalConfidenceByNumNeighbors/index.html, updated August 15, 2007). (PDF) [file ppat.1004555.s014.pdf]

Table S4. Predicted subcellular locations for high-confidence protein groups (protein probability: 1% false discovery rate, p ≥ 0.95,) identified in the T. brucei acidocalcisome fractions (ACCS1 and ACCS2) from our prediction servers using non-plant based algorithms. Individual protein predictions are based upon agreement between at least two analysis packages. Consensus for identifications with multiple protein hits (see Methods for explanation of protein grouping based on degenerate peptide fingerprints) is given when prediction for a compartment agrees among two of more members of the group. S, secreted. C, cytosol. M, mitochondrion. N, nucleus. PM, plasma membrane. G, Golgi complex. ER, endoplasmic reticulum. P, peroxisome. L, lysosome. CYKS, cytoskeleton. Threshold probabilities and confidences used to screen our predictions with poor reliability: targetP (RC = 1). pTarget ≥ 80%, SLP-LOCAL ≥ 2, WoLfPsort ≥ ~80%. WoLFPSORT thresholds for each subcellular location were derived from empirical prediction confidence statistics (wolfpsort.org/empiricalConfidenceByNumNeighbors/index.html, updated August 15, 2007).

				TargetP		pTARGET		SLP-LOCAL		WoLF-PSORT	
Protein Group	GeneID	Description	Group Consensus	Location	Reliability	Location	Confidence	Location	Reliability	Location	Individual Consensus
1	Tb927.6.3740	heat shock 70 kDa protein, mitochondrial precursor, putative	M			M	81.4			M	M
	Tb927.6.3750	heat shock 70 kDa protein, mitochondrial precursor, putative				M	81.4			M	M
	Tb927.6.3800	heat shock 70 kDa protein, mitochondrial precursor, putative				M	81.4			M	M
2	Tb927.11.6280	pyruvate phosphate dikinase	C			C	100	N C	4		C
3	Tb927.1.2330	beta tubulin	C			C	87.6	N C	3		C
	Tb927.1.2350	beta tubulin				C	87.6	N C	3		C
	Tb927.1.2370	beta tubulin				C	87.6	N C	3		C
	Tb927.1.2390	beta tubulin				C	87.6	N C	3		C
4	Tb927.10.6400	chaperonin HSP60, mitochondrial precursor	M			M	93.9	M	2		M
	Tb927.10.6510	chaperonin HSP60, mitochondrial precursor				M	93.9	M	2		M
5	Tb927.3.1380	ATP synthase beta chain, mitochondrial precursor,ATP synthase F1, beta subunit	M			M	93.9	M	3	M	M
6	Tb927.2.4210	glycosomal phosphoenolpyruvate carboxykinase				P	81.4				
7	Tb927.11.7460	glucose-regulated protein 78, putative,luminal binding protein 1 (BiP), putative	S	S	1	M	81.4	S	2		S
	Tb927.11.7510	glucose-regulated protein 78, putative,luminal binding protein 1 (BiP), putative		S	1	M	81.4	S	2		S
8	Tb927.10.5620	fructose-bisphosphate aldolase, glycosomal				P	81.4				
9	Tb927.1.2340	alpha tubulin	C			C	81.4	N C	2		C
	Tb927.1.2360	alpha tubulin				C	81.4	N C	2		C
	Tb927.1.2380	alpha tubulin				C	81.4	N C	2		C
	Tb927.1.2400	alpha tubulin				C	81.4	N C	2		C
10	Tb927.6.4280	glyceraldehyde 3-phosphate dehydrogenase, glycosomal				M	93.9				
	Tb927.6.4300	glyceraldehyde 3-phosphate dehydrogenase, glycosomal				M	93.9				
11	Tb927.8.3530	glycerol-3-phosphate dehydrogenase [NAD], glycosomal									
12	Tb927.11.11330	heat shock protein 70	C			C	87.6	N C	3		C
13	Tb927.11.9980	2-oxoglutarate dehydrogenase E1 component, putative				P	81.4				
14	Tb927.10.15410	glycosomal malate dehydrogenase				M	87.6				
15	Tb927.9.12570	glycerol kinase, glycosomal						N C	2		
16	Tb927.5.930	NADH-dependent fumarate reductase				P	100	N C	2		
17	Tb927.11.3980	mitochondrial processing peptidase alpha subunit, putative,metallo-peptidase, Clan ME, Family M16				P	87.6			M	
18	Tb927.2.4230	NUP-1 protein, putative	N			N	100	N C	3		N
19	Tb927.3.2230	succinyl-CoA synthetase alpha subunit, putative	M			M	100	M	2		M
20	Tb927.5.1060	mitochondrial processing peptidase, beta subunit, putative,metallo-peptidase, Clan ME, Family M16				G	81.4			M	
21	Tb927.11.5520	triosephosphate isomerase	C			C	87.6	N C	2		C
22	Tb927.4.2070	antigenic protein, putative	C			C	87.6	N C	4		C
23	Tb927.11.11680	2-oxoglutarate dehydrogenase E2 component, putative	M			M	87.6			M	M
24	Tb927.10.8030	hypothetical protein, conserved	M					M	2	M	M
25	Tb927.10.7410	succinyl-CoA ligase [GDP-forming] beta-chain, putative-with=GeneDB:LmjF36.2950	M			M	87.6			M	M
26	Tb927.9.5900	glutamate dehydrogenase								M	
27	Tb927.5.2930	hypothetical protein, conserved				G	81.4			N	
28	Tb927.5.2080	guanosine monophosphate reductase, putative	C			C	100	N C	3		C
29	Tb927.9.5320	nucleolar RNA binding protein, putative				P	81.4				
30	Tb927.8.2770	inositol 1,4,5-trisphosphate receptor	C			C	81.4	N C	3	PM	C
31	Tb927.10.3210	delta-1-pyrroline-5-carboxylate dehydrogenase, putative				P	87.6				
32	Tb927.11.1020	ribokinase, putative	C			C	87.6	N C	2		C
33	Tb927.11.2690	succinyl-coA:3-ketoacid-coenzyme A transferase, mitochondrial precursor, putative				P	87.6				
34	Tb927.11.1450	2-oxoglutarate dehydrogenase E1 component, putative	M	M	1	P	81.4	M	2		M
35	Tb927.10.14820	mitochondrial carrier protein, putative,ADP/ATP translocase 1, putative						N C	3		
	Tb927.10.14830	mitochondrial carrier protein, putative,ADP/ATP translocase 1, putative						N C	3		
	Tb927.10.14840	mitochondrial carrier protein, putative,ADP/ATP translocase 1, putative						N C	3		
36	Tb927.10.14550	ATP-dependent DEAD/H RNA helicase, putative								N	
37	Tb927.10.2090	elongation factor 1-alpha,EF-1-alpha	C			C	100				
	Tb927.10.2100	elongation factor 1-alpha,EF-1-alpha				C	93.9	N C	2	C	C
	Tb927.10.2110	elongation factor 1-alpha				C	93.9	N C	2	C	C
38	Tb927.7.7420	ATP synthase alpha chain, mitochondrial precursor,ATP synthase F1, alpha subunit	M			M	100	M	3	M	M
	Tb927.7.7430	ATP synthase alpha chain, mitochondrial precursor,ATP synthase F1, alpha subunit				M	100	M	3	M	M
39	Tb927.10.2020	hexokinase	C			C	93.9	N C	3		C
40	Tb927.10.1510	NOT1				P	93.9	N C	2		
41	Tb927.2.1560	cyclophilin-type peptidyl-prolyl cis-trans isomerase, putative									
	Tb927.2.1680	cyclophilin-type peptidyl-prolyl cis-trans isomerase, putative									
42	Tb927.3.3580	lipophosphoglycan biosynthetic protein, putative	S	S	1	M	87.6	S	3		S
43	Tb927.3.1790	pyruvate dehydrogenase E1 beta subunit, putative	M			M	87.6	M	3	M	M
44	Tb927.10.7570	dihydrolipoamide acetyltransferase E2 subunit, putative	M			M	93.9			M	M
45	Tb927.5.3400	calcium-translocating P-type ATPase,calcium pump				P	100	N C	3		
46	Tb927.8.3750	nucleolar protein, putative				C	87.6				
47	Tb927.1.4100	cytochrome oxidase subunit IV	M			N	81.4	M	3	M	M

48	Tb927.8.2540	3-ketoacyl-CoA thiolase, putative	M			M	93.9			M	M
49	Tb927.8.5640	hypothetical protein, conserved	M	M	1					M	M
50	Tb927.10.1170	intraflagellar transport protein IFT172, putative				C	81.4				
51	Tb927.10.12700	pyruvate dehydrogenase E1 alpha subunit, putative	M					M	2	M	M
52	Tb927.10.16120	inosine-5'-monophosphate dehydrogenase,IMP dehydrogenase				C	87.6				
53	Tb927.9.11580	Gim5A protein,glycosomal membrane protein	PM			PM	81.4	N C	3		
	Tb927.9.11600	Gim5B protein,glycosomal membrane protein				PM	81.4	N C	3	PM	PM
54	Tb927.10.2010	hexokinase	C			C	87.6	N C	3		C
55	Tb927.8.1740	hypothetical protein, conserved				P	81.4			M	
56	Tb927.8.6110	hypothetical protein, conserved	M			M	87.6			M	M
57	Tb927.10.2560	mitochondrial malate dehydrogenase				P	87.6	M	2		
58	Tb927.10.4560	elongation factor 2	C			C	100	N C	4		C
	Tb927.10.4570	elongation factor 2				C	100	N C	4		C
59	Tb927.6.2230	hypothetical protein, conserved	N			N	87.6	N C	3		N
60	Tb927.10.8940	hypothetical protein, conserved	N			N	93.9	N C	2		N
61	Tb927.10.7020	acid phosphatase, putative		S	1	G	87.6				
62	Tb927.6.4440	hypothetical protein, conserved	N			N	93.9	N C	3		N
63	Tb927.8.6060	2-amino-3-ketobutyrate coenzyme A ligase, putative,glycine acetyltransferase, putative	M			M	93.9	M	2	M	M
64	Tb927.6.2790	L-threonine 3-dehydrogenase, putative				M	93.9				
65	Tb927.4.1300	hypothetical protein, conserved				P	87.6	N C	2		
66	Tb927.10.4310	prohibitin 2, putative				M	87.6	N C	2		
67	Tb927.9.14160	rieske iron-sulfur protein, mitochondrial precursor				G	93.9	M	2		
68	Tb927.1.120	retrotransposon hot spot protein 4 (RHS4), putative	C			C	81.4	N C	5		C
69	Tb927.10.2350	pyruvate dehydrogenase complex E3 binding protein, putative				M	81.4				
70	Tb927.10.180	ATP synthase F1 subunit gamma protein, putative						N C	3		
71	Tb927.3.3900	carnitine O-palmitoyltransferase II, putative				G	100			M	
72	Tb927.2.3030	ATP-dependent Clp protease subunit, heat shock protein 78 (HSP78), putative,serine peptidase, putative	M			M	87.6			M	M
73	Tb927.3.3270	ATP-dependent phosphofructokinase				P	93.9				
74	Tb927.10.2240	hypothetical protein, conserved									
75	Tb927.11.16730	dihydrolipoyl dehydrogenase				C	87.6				
76	Tb927.9.12510	ATP-dependent DEAD/H RNA helicase, putative	N			N	100	N C	3	N	N
77	Tb927.3.5050	60S ribosomal protein L4	N			N	93.9	N C	2		N
78	Tb927.4.4380	vacuolar-type proton translocating pyrophosphatase 1, putative	S	S	1			S	2	PM	S
	Tb927.8.7980	vacuolar-type proton translocating pyrophosphatase 1,V-type H( )-translocating pyrophosphatase		S	1			S	2	PM	S
79	Tb927.9.5150	ribosomal protein S6, putative,NHP2/RS6-like protein	C			C	93.9	N C	4	C	C
80	Tb927.4.3950	cytoskeleton-associated protein CAP5.5, putative,cysteine peptidase, Clan CA, family C2, putative						N C	3		
81	Tb927.3.3130	hypothetical protein, conserved									
82	Tb927.9.6100	TFIIF-stimulated CTD phosphatase, putative				M	100				
83	Tb927.8.6170	transketolase, putative				P	100	N C	2		
84	Tb927.3.930	dynein heavy chain, putative				P	81.4	N C	2		
85	Tb927.10.14750	fibrillarin, putative	N			N	93.9	N C	2	N	N
86	Tb927.9.6090	TFIIF-stimulated CTD phosphatase, putative				M	100				
87	Tb927.11.2650	heat shock protein 84, putative	M	M	1	M	93.9	M	3	M	M
88	Tb927.11.13180	hypothetical protein, conserved				M	100	N C	2		
89	Tb927.9.10770	Polyadenylate-binding protein 2 (Poly(A)-binding protein 2) (Poly(A)-binding protein II) (PABII) (Polyadenylate-binding nuclear protein 1) (Nuclear poly(A)-binding protein 1) (PABP2), putative				P	81.4	N C	2		
90	Tb927.8.6580	succinate dehydrogenase flavoprotein, putative	M			P	81.4	M	2	M	M
91	Tb927.11.7380	glycerol-3-phosphate dehydrogenase (FAD-dependent), mitochondrial				M	81.4				
92	Tb927.9.2470	nucleolar protein	C			C	87.6	N C	4		C
93	Tb927.8.1160	vacuolar-type Ca2 -ATPase, putative				M	87.6	N C	3	PM	
94	Tb927.7.2700	NADH-cytochrome b5 reductase, putative				P	87.6				
95	Tb927.10.470	choline dehydrogenase, putative				P	81.4				
96	Tb927.8.1180	vacuolar-type Ca2 -ATPase 1				P	87.6	N C	4	PM	
97	Tb927.11.13280	mitochondrial RNA binding protein 2				G	87.6			M	
98	Tb927.5.1210	short-chain dehydrogenase, putative	S	S	1	ER	93.9	S	2		S
99	Tb927.6.2170	co-chaperone GrpE, putative	M	M	1	M	81.4	M	4	M	M
100	Tb927.7.6360	histone H2A variant				P	93.9	N C	3		
101	Tb927.10.12500	P-type H -ATPase, putative						N C	5	PM	
102	Tb927.5.3810	orotidine-5-phosphate decarboxylase/orotate phosphoribosyltransferase, putative,OMPDCase-OPRTase, putative				G	87.6				
103	Tb927.10.13510	zinc metallopeptidase, putative				M	87.6	N C	3		
104	Tb927.5.520	stomatin-like protein, putative				L	87.6	S	2		
105	Tb927.11.3250	dynein heavy chain, putative	C			C	93.9	N C	6		C
106	Tb927.10.11310	intraflagellar transport protein IFT55/IFT57, putative	C			C	100	N C	6		C
107	Tb927.8.3060	cytosolic leucyl aminopeptidase, putative,metallo-peptidase, Clan MF, Family M17				P	81.4				
108	Tb927.8.1330	60S ribosomal protein L7a, putative	N			N	87.6	N C	3	N	N
	Tb927.8.1340	60S ribosomal protein L7a, putative				N	87.6	N C	3	N	N
109	Tb927.10.6910	Sterol methyltransferase, putative									
	Tb927.10.6950	sterol 24-c-methyltransferase, putative									
110	Tb927.9.9660	hypothetical protein, conserved						N C	6		
111	Tb927.2.2510	voltage-dependent anion-selective channel						N C	3		
	Tb927.2.2520	voltage-dependent anion-selective channel						N C	3		
112	Tb927.11.6460	hypothetical protein, conserved				P	81.4				
113	Tb927.7.6670	hypothetical protein, conserved				G	81.4			PM	

114	Tb927.3.4630	UDP-glucose:glycoprotein glucosyltransferase, putative				M	81.4				
115	Tb927.8.1510	ATP-dependent DEAD/H RNA helicase, putative	N			P	87.6	N C	3	N	N
116	Tb927.11.11520	glycosomal membrane protein, putative						N C	2		
117	Tb927.3.2600	ATP-dependent DEAD/H RNA helicase, putative				P	93.9	N C	6		
118	Tb927.8.5200	hypothetical protein, conserved									
119	Tb927.9.5730	nucleosome assembly protein-like protein	N			N	93.9	N C	6		N
120	Tb927.9.12730	chaperone protein DNAj, putative	M	M	1			M	2		M
121	Tb927.8.3380	electron transfer protein, putative				P	93.9				
122	Tb927.11.11010	hypothetical protein, conserved	N			N	93.9	N C	5		N
123	Tb927.4.1500	RNA editing associated helicase 2									
124	Tb927.3.3310	60S ribosomal protein L13, putative	N			N	87.6	N C	4		N
	Tb927.3.3320	60S ribosomal protein L13, putative				N	87.6	N C	4		N
125	Tb927.11.5450	malic enzyme, putative	M			M	87.6	M	2		M
126	Tb927.2.450	retrotransposon hot spot protein 4 (RHS4), putative						N C	5		
127	Tb927.10.13800	hypothetical protein, conserved	N			N	100	N C	4		N
128	Tb927.10.14600	40S ribosomal protein S2, putative				M	93.9	N C	2		
	Tb927.10.14710	40S ribosomal protein S2, putative				M	93.9	N C	2		
129	Tb927.9.3170	cytochrome oxidase subunit V	M			M	81.4	M	2		M
130	Tb927.10.520	hypothetical protein, conserved				G	81.4				
131	Tb927.11.1090	calpain-like protein, putative,cytoskeleton assocociated protein, putative	N			N	93.9	N C	7		N
132	Tb927.8.7530	3,2-trans-enoyl-CoA isomerase, mitochondrial precursor, putative				G	93.9	M	2		
133	Tb927.10.450	hypothetical protein, conserved	C			C	93.9	N C	4		C
134	Tb927.11.13090	elongation factor 1 gamma, putative	C			G	87.6	S	2		
	Tb927.11.13190	elongation factor 1 gamma, putative				C	81.4	N C	3		C
135	Tb927.7.1730	60S ribosomal protein L7, putative				P	87.6			C	
136	Tb927.6.1520	aquaporin 3, putative						N C	5	PM	
137	Tb927.11.6250	hypothetical protein, conserved				P	87.6	M	2		
138	Tb927.11.3590	40S ribosomal protein S4, putative				M	93.9				
	Tb927.11.3600	40S ribosomal protein S4, putative				M	93.9				
139	Tb927.7.3550	hypothetical protein, conserved	N			N	100	N C	4	N	N
140	Tb927.3.4760	dynamin, putative,vacuolar sortin protein 1, putative				P	93.9	N C	3		
141	Tb927.9.10310	mitochondrial carrier protein, putative,mitochondrial phosphate transporter, putative						N C	2		
142	Tb927.9.10400	hypothetical protein, conserved	N			N	93.9	N C	5		N
143	Tb927.10.14500	hypothetical protein, conserved	N			N	87.6	N C	2		N
144	Tb927.7.1300	protein disulfide isomerase, putative									
145	Tb927.10.3940	40S ribosomal protein S3A, putative				P	93.9	N C	4		
146	Tb927.2.4710	RNA-binding protein, putative	N			N	87.6	N C	3		N
147	Tb927.11.2060	60S acidic ribosomal subunit protein, putative	C			P	87.6	N C	3	C	C
	Tb927.11.2050	60S acidic ribosomal subunit protein, putative				P	87.6	N C	3	C	C
148	Tb927.4.2450	thioredoxin, putative	S	S	1	ER	93.9	S	2	S	S
149	Tb927.4.4210	ATP-dependent zinc metallopeptidase, putative,metallo-peptidase, Clan MA(E) Family M41									
150	Tb927.10.7500	fibrillarin	N			M	93.9	N C	2	N	N
151	Tb927.11.3490	hypothetical protein, conserved	C			C	81.4	N C	2		C
152	Tb927.11.2670	Nucleoporin				P	93.9			M	
153	Tb927.10.3650	NADH-dependent fumarate reductase, putative									
154	Tb927.5.1300	vacuolar proton translocating ATPase subunit A, putative				ER	87.6	N C	2	PM	
155	Tb927.3.4970	hypothetical protein, conserved				P	87.6	N C	5		
156	Tb927.8.4810	prohibitin 1	M			M	93.9			M	M
157	Tb927.10.3990	DHH1	N C			C	100	N C	7	N	N C
158	Tb927.11.8260	carbonic anhydrase-like protein	S	S	1	G	87.6	S	2		S
159	Tb927.8.900	splicing factor TSR1	N			N	93.9	N C	6	N	N
160	Tb927.9.2900	hypothetical protein, conserved						N C	3	PM	
161	Tb927.9.4210	fatty acyl CoA synthetase 3	C			C	93.9	N C	4		C
162	Tb927.3.1120	GTP-binding nuclear protein rtb2, putative				P	87.6				
163	Tb927.5.3230	hypothetical protein, conserved	N			N	87.6	N C	2	PM	N
164	Tb927.11.1270	hypothetical protein, conserved				G	81.4	N C	4		
165	Tb927.2.3780	translation initiation factor IF-2, putative				M	93.9	N C	4		
166	Tb927.11.13500	par1	C			C	87.6	N C	3		C
167	Tb927.11.4480	radial spoke protein RSP4/6, putative	N			N	81.4	N C	3		N
168	Tb927.5.1470	NADH-cytochrome b5 reductase, putative				M	100				
169	Tb927.8.1890	cytochrome c1, heme protein, mitochondrial precursor				G	81.4	N C	4		
170	Tb927.11.2410	hypothetical protein, conserved	C			C	93.9	N C	6	C	C
171	Tb927.11.14730	metalloprotease, putative,cell division protein FtsH homologue, putative	C			C	87.6	N C	4		C
172	Tb927.6.4090	chaperonin HSP60, mitochondrial precursor, putative				P	81.4				
173	Tb927.3.3490	high mobility group protein, putative	N			N	93.9	N C	6		N
174	Tb927.1.4310	hypothetical protein, conserved	N			N	87.6	N C	6		N
175	Tb927.7.1790	Adenine phosphoribosyltransferase, putative				P	87.6	N C	3		
176	Tb927.11.11360	guanine nucleotide-binding protein beta subunit- like protein,activated protein kinase c receptor				L	100				
	Tb927.11.11370	guanine nucleotide-binding protein beta subunit-like protein,activated protein kinase c receptor				L	100				
177	Tb927.9.10560	hypothetical protein, conserved								PM	
178	Tb927.3.3670	RNA-binding protein, putative	N			N	93.9			N	N
179	Tb927.11.12150	flagellar protein essential for flagellar pocket biogenesis				P	81.4				

180	Tb927.10.3260	Long-chain-fatty-acid--CoA ligase 5 (EC 6.2.1.3) (Long-chain acyl-CoA synthetase 5) (LACS 5), putative	C			C	93.9	N C	2		C
181	Tb927.3.2180	hypothetical protein, conserved				M	81.4				
182	Tb927.10.560	40S ribosomal proteins S11, putative	N C			N	93.9	N C	3	C	N C
183	Tb927.7.4770	cyclophilin-type peptidyl-prolyl cis-trans isomerase, putative	C			C	81.4	N C	3		C
184	Tb927.11.550	hypothetical protein SCD6.10								M	
185	Tb927.8.890	small GTP-binding protein Rab1, putative									
186	Tb927.7.2570	mRNA processing protein, putative	M	M	1	G	93.9	M	2	M	M
187	Tb927.10.15180	nucleosome assembly protein, putative	C			C	93.9	N C	6		C
188	Tb927.11.1430	hypothetical protein, conserved	C			C	81.4	N C	3		C
189	Tb927.9.9550	hypothetical protein, conserved	N			N	100	N C	6	N	N
190	Tb927.4.5010	calreticulin, putative		S	1						
	Tb927.8.7410	calreticulin, putative		S	1						
191	Tb927.7.3740	hypothetical protein, conserved	N			N	93.9	N C	5		N
192	Tb927.10.9570	paraflagellar rod component, putative	C			C	93.9	N C	3		C
193	Tb927.8.1570	hypothetical protein, conserved						N C	3		
194	Tb927.10.6180	hypothetical protein, conserved	S	S	1	PM	81.4	S	4		S
195	Tb927.10.1390	hypoxanthine-guanine phosphoribosyltransferase, putative	C			P	93.9	N C	3	C	C
196	Tb927.11.12220	vacuolar transporter chaperone 4						N C	3	PM	
197	Tb927.8.6660	paraflagellar rod component, putative	N			N	93.9	N C	4		N
198	Tb927.8.4780	hypothetical protein, conserved	N			N	100	N C	3		N
199	Tb927.3.3460	hypothetical protein, conserved	M			M	81.4	M	2	M	M
200	Tb927.11.9080	hypothetical protein, conserved	C			C	93.9	N C	6		C
201	Tb927.11.14130	ribosomal protein L18, putative	N			N	81.4	N C	2		N
	Tb927.10.3840	60S ribosomal protein L18a, putative				N	81.4	N C	2		N
202	Tb927.10.4880	hypothetical protein, conserved				G	93.9				
203	Tb927.9.11220	hypothetical protein, conserved				P	81.4	N C	2		
204	Tb927.8.3870	hypothetical protein, conserved	N			N	87.6	N C	4	N	N
205	Tb927.2.5160	chaperone protein DNAj, putative	C			C	100	N C	6		C
206	Tb927.10.240	peroxin 14, putative	N			N	100	N C	5		N
207	Tb927.10.8230	protein disulfide isomerase,bloodstream- specific protein 2 precursor				ER	93.9				
208	Tb927.6.4210	aldehyde dehydrogenase, putative									
209	Tb927.6.3150	Hydin,flagellar component				P	93.9	N C	2		
210	Tb927.10.7090	alternative oxidase	M			M	100	M	3	M	M
211	Tb927.11.1710	mitochondrial RNA binding protein 1,gBP21, MRP1								M	
212	Tb927.3.1810	hypothetical protein, conserved						N C	2		
213	Tb927.9.9450	hypothetical protein, conserved,zinc finger protein family member, putative	N			N	100	N C	7		N
214	Tb927.11.8160	dynein heavy chain, putative	C			C	87.6	N C	4		C
215	Tb927.4.3590	translation elongation factor 1-beta, putative				C	87.6				
216	Tb927.9.2450	electon transport protein SCO1/SCO2, putative	C			C	81.4	N C	5		C
217	Tb927.3.2440	serine/threonine-protein kinase, putative,protein kinase, putative	N			P	81.4	N C	6	N	N
218	Tb927.9.4190	fatty acyl CoA syntetase 1				G	93.9				
219	Tb927.4.4070	mevalonate kinase, putative	C			C	100	N C	3		C
220	Tb927.10.2530	adenylate kinase, putative	C			C	100	N C	2	C	C
221	Tb927.5.500	hypothetical protein, conserved				N	87.6				
222	Tb927.2.3800	mRNA processing protein, putative	M	M	1	M	93.9	M	2		M
223	Tb927.7.2170	hypothetical protein, conserved	N			N	87.6	N C	2	N	N
224	Tb927.8.1990	peroxidoxin	M	M	1	M	81.4	M	3	M	M
225	Tb927.10.12840	mitochondrial carrier protein, putative,mitochondrial 2-oxoglutarate/malate carrier protein, putative						N C	2		
226	Tb927.7.2390	hypothetical protein, conserved	N			N	100	N C	3		N
227	Tb927.4.1330	DNA topoisomerase IB, large subunit	C			C	100	N C	6		C
228	Tb927.11.12230	heat shock protein HslVU, ATPase subunit HslU, putative,ATP-dependent hsl protease ATP-binding subunit hslU, putative				M	87.6				
229	Tb927.8.3690	isocitrate dehydrogenase [NADP], mitochondrial precursor, putative	M	M	1	M	87.6	M	2	M	M
230	Tb927.11.15040	chaperonin HSP60, mitochondrial precursor, putative,heat shock protein 60				M	87.6				
231	Tb927.6.3050	aldehyde dehydrogenase family, putative	C			C	93.9	N C	4		C
232	Tb927.8.5120	cytochrome c				P	93.9	N C	3		
233	Tb927.6.1500	alkyl-dihydroxyacetone phosphate synthase	C			C	81.4	N C	5		C
234	Tb927.2.5660	adenylate kinase, putative	C			C	93.9	N C	2		C
235	Tb927.10.5770	valosin-containing protein homolog,Transitional endoplasmic reticulum ATPase, putative	C			C	93.9	N C	4		C
236	Tb927.11.5290	mitochondrial carrier protein, putative				PM	93.9				
237	Tb927.9.5690	60S acidic ribosomal protein, putative				P	87.6				
238	Tb927.11.6140	40S ribosomal protein S15A, putative				P	81.4				
239	Tb927.11.600	hypothetical protein, conserved				M	81.4				
240	Tb927.2.5800	sedoheptulose-1,7-bisphosphatase				P	81.4				
241	Tb927.4.2080	hypothetical protein, conserved	N			N	93.9	N C	6		N
242	Tb927.10.8780	hypothetical protein, conserved	C			C	93.9	N C	4		C
243	Tb927.10.15750	hypothetical protein, conserved	N			N	87.6	N C	2		N
244	Tb927.9.4500	heat shock protein, putative,HSP70-like protein	S	S	1			S	5		S
245	Tb927.9.4680	ATP-dependent DEAD box helicase, putative	N C			C	93.9	N C	7	N	N C
246	Tb927.4.870	dynein heavy chain, putative				G	93.9				
247	Tb927.10.5760	adenylate kinase, putative				M	87.6	N C	3		
248	Tb927.10.13180	hypothetical protein, conserved						N C	4		
249	Tb927.11.14780	phosphomannose isomerase, putative	C			C	93.9	N C	3		C

250	Tb927.2.2940	hypothetical protein, conserved	N			N	87.6	N C	4		N
251	Tb927.10.10360	microtubule-associated protein, putative	N			N	100	N C	8		N
252	Tb927.4.1360	hypothetical protein, conserved	C			M	93.9	N C	2	C	C
253	Tb927.11.15150	1-acyl-sn-glycerol-3-phosphate acyltransferase, putative	S	S	1	ER	100	S	4		S
254	Tb927.10.8200	hypothetical protein, conserved	N			N	81.4	N C	3		N
255	Tb927.7.4900	5'-3' exonuclease XRNA, putative,exoribonuclease 1, putative				G	87.6	N C	3		
256	Tb927.9.5960	succinate dehydrogenase, putative				S	87.6				
257	Tb927.11.9400	hypothetical protein, conserved	C			C	100	N C	5		C
258	Tb927.7.210	proline dehydrogenase	M			M	87.6	M	2	M	M
259	Tb927.11.8870	mitochondrial DEAD box protein,KREH1	M	M	1	G	87.6	M	3		M
260	Tb927.11.1980	hypothetical protein, conserved,zinc finger protein family member, putative				P	87.6	N C	3		
261	Tb927.4.4910	3,2-trans-enoyl-CoA isomerase, mitochondrial precursor, putative				G	93.9				
262	Tb927.10.16150	ATP-dependent zinc metallopeptidase, putative,metallo-peptidase, Clan MA(E) Family M41				P	87.6	N C	3		
263	Tb927.11.8050	hypothetical protein, conserved	N			N	100	N C	7	N	N
264	Tb927.10.2950	hypothetical protein, conserved				G	81.4				
265	Tb927.8.1550	paraflagellar rod component, putative	N			N	93.9	N C	3		N
266	Tb927.5.4420	nucleolar RNA helicase II, putative,nucleolar RNA helicase Gu, putative	C			C	93.9	N C	5		C
267	Tb927.10.770	hypothetical protein, conserved	M	M	1	M	81.4			M	M
268	Tb927.11.3360	hypothetical protein, conserved						N C	2		
269	Tb927.6.4980	40S ribosomal protein S14	N			N	87.6	N C	5		N
270	Tb927.10.10280	microtubule-associated protein, putative	N			N	100	N C	8		N
271	Tb927.3.1840	3-oxo-5-alpha-steroid 4-dehydrogenase, putative				P	87.6	N C	4		
272	Tb927.11.4450	hypothetical protein, conserved				P	87.6	N C	3		
273	Tb927.7.3370	hypothetical protein, conserved	N			N	93.9	N C	2	N	N
274	Tb927.8.4400	hypothetical protein, conserved				N	93.9				
275	Tb927.11.10760	kinesin-like protein, putative				P	93.9	N C	2		
276	Tb927.10.9780	ATP-dependent DEAD/H RNA helicase, putative,ATP- dependent RNA helicase, putative	N			N	100	N C	2	N	N
277	Tb927.11.16510	hypothetical protein, conserved				M	87.6				
278	Tb927.11.17000	hypothetical protein, conserved,leucine-rich repeat protein (LRRP), putative				P	87.6	N C	2		
279	Tb927.9.15360	40S ribosomal protein S6, putative				P	100	N C	5		
280	Tb927.10.170	pseudouridine synthase, Cbf5p	C			P	87.6	N C	4	C	C
281	Tb927.3.5570	syntaxin, putative	C			C	87.6	N C	3		C
282	Tb927.10.6630	ATP-dependent DEAD/H RNA helicase HEL64, putative	N			N	81.4	N C	5	N	N
283	Tb927.8.2740	mitochondrial RNA binding protein				P	81.4				
284	Tb927.10.2770	eukaryotic translation initiation factor 5, putative	C			C	100	N C	5		C
285	Tb927.7.3980	immunodominant antigen, putative,tc40 antigen-like	N			N	87.6	N C	5		N
286	Tb927.3.3590	U3 small nucleolar ribonucleoprotein protein MPP10, putative	N			N	87.6	N C	4		N
287	Tb927.11.11540	DNA topoisomerase II, putative	C			C	100	N C	5		C
288	Tb927.7.1290	hypothetical protein, conserved	S	S	1			S	3		S
289	Tb927.11.2790	hypothetical protein, conserved	N			N	93.9	N C	3		N
290	Tb927.8.2000	cyclophilin, putative									
291	Tb927.8.1860	pitrilysin-like metalloprotease,metallo-peptidase, Clan ME, Family M16C								M	
292	Tb927.11.6210	sterol 14-alpha-demethylase	S	S	1			S	4	ER	S
293	Tb927.6.4130	hypothetical protein, conserved	M			M	100			M	M
294	Tb927.9.13990	RNA-binding protein, putative	N			N	87.6	N C	7	S	N
295	Tb927.6.2050	ribosome biogenesis regulatory protein (RRS1), putative						N C	2		
296	Tb927.4.410	CAF 40						N C	3		
297	Tb927.7.280	cyclophilin-type peptidyl-prolyl cis-trans isomerase, putative				PM	87.6				
298	Tb927.10.6050	clathrin heavy chain				P	81.4	N C	2		
299	Tb927.9.14200	hypothetical protein, conserved				G	87.6	M	2		
300	Tb927.3.5020	hypothetical protein, conserved				P	87.6	N C	3		
301	Tb927.8.6390	lysophospholipase, putative,alpha/beta hydrolase, putative				P	81.4	N C	4		
302	Tb927.11.6050	hypothetical protein, conserved	N			N	87.6	N C	3	PM	N
303	Tb927.2.4550	FtsJ cell division protein, putative	N			N	93.9	N C	3		N
304	Tb927.9.15330	hypothetical protein, conserved	N			N	93.9	N C	2		N
305	Tb927.11.5220	chaperone protein DNAj, putative	S	S	1			S	3	S	S
306	Tb927.11.3500	hypothetical protein, conserved						N C	4		
307	Tb927.6.1470	hypothetical protein, conserved	N			N	93.9	N C	5	N	N
308	Tb927.1.1700	hypothetical protein, conserved	N			N	100	N C	4		N
309	Tb927.2.5130	hypothetical protein, conserved	C			C	87.6	N C	4		C
310	Tb927.8.2630	kinesin, putative	N			N	100	N C	4		N
311	Tb927.11.6300	40S ribosomal protein S5, putative	C			C	87.6			C	C
312	Tb927.9.1750	hypothetical protein, conserved	C			C	81.4	N C	3		C
313	Tb927.9.11000	small GTPase, putative,GTP-binding protein, putative				G	93.9	N C	2		
314	Tb927.9.11850	structural maintenance of chromosome 1, putative	N			N	93.9	N C	2		N
315	Tb927.10.4760	hypothetical protein, conserved								S	
316	Tb927.6.3890	replication factor C, subunit 2, putative	C			C	93.9	N C	5		C
317	Tb927.11.16480	enoyl-CoA hydratase/isomerase family protein, putative									
318	Tb927.7.2190	hypothetical protein, conserved		S	1						
319	Tb927.7.3430	cyclophilin-type peptidyl-prolyl cis-trans isomerase, putative						S	2		
320	Tb927.11.3770	hypothetical protein, conserved				P	81.4				
321	Tb927.7.3330	hypothetical protein, conserved	N			N	93.9	N C	6		N

322	Tb927.11.7900	mitochondrial RNA binding protein,RBP16,RNA- binding protein of 16 kDa									M	
323	Tb927.11.10240	hslVU complex proteolytic subunit, putative,hslVU complex proteolytic subunit, threonine peptidase, Clan T(1), family T1B	M			M	87.6				M	M
324	Tb927.6.2640	importin alpha subunit, putative				P	87.6	N C	4			
325	Tb927.10.4000	methylglutaconyl-CoA hydratase, mitochondrial precursor, putative										
326	Tb927.4.1540	hypothetical protein, conserved				G	93.9	N C	2			
327	Tb927.6.4630	kinetoplastid-specific phospho-protein phosphatase, putative				L	87.6					
328	Tb927.11.5600	hypothetical protein, conserved						N C	2			
329	Tb927.10.5990	hypothetical protein, conserved	N			N	93.9	N C	2		N	N
330	Tb927.2.3610	hypothetical protein, conserved				P	81.4				S	
331	Tb927.11.10150	hypothetical protein, conserved				G	87.6					
332	Tb927.7.640	hypothetical protein, conserved				G	81.4				PM	
333	Tb927.7.5940	Protein Associated with Differentiation	PM			PM	81.4	N C	3		PM	PM
334	Tb927.10.7380	hypothetical protein, conserved	M	M	1	L	87.6	M	2		PM	M
335	Tb927.11.4680	hypothetical protein, conserved				M	87.6	N C	3			
336	Tb927.9.2320	hypothetical protein, conserved				ER	87.6	S	3			
337	Tb927.10.11520	hypothetical protein, conserved	N			N	87.6	N C	3		S	N
338	Tb927.10.3040	hypothetical protein, conserved				M	81.4	N C	2			
339	Tb927.10.14700	hypothetical protein, conserved	N			N	100	N C	5			N
340	Tb927.7.680	chaperone protein DNAj, putative				G	87.6					
341	Tb927.5.4330	dihydrolipoamide branched chain transacylase, putative				M	81.4					
342	Tb927.8.610	hypothetical protein, conserved	C			C	87.6	N C	3			C
343	Tb927.4.4700	hypothetical protein, conserved	N			N	93.9	N C	4			N
344	Tb927.10.12590	hypothetical protein, conserved	N			N	100	N C	7			N
345	Tb927.7.3500	glutathione-S-transferase/glutaredoxin, putative										
346	Tb927.11.900	isocitrate dehydrogenase, putative				P	87.6	N C	3			
347	Tb927.5.2790	mitochondrial DNA polymerase beta-PAK	M	M	1	M	81.4	M	2		M	M
348	Tb927.5.4470	hypothetical protein, conserved	N			N	93.9	N C	4			N
349	Tb927.3.5430	hypothetical protein, conserved	S	S	1	ER	100	S	3			S
350	Tb927.10.4080	hypothetical protein, conserved						N C	2			
351	Tb927.10.7810	hypothetical protein, conserved	N			N	93.9	M	2		N	N
352	Tb927.11.10690	hypothetical protein, conserved	M			M	93.9	M	2			M
353	Tb927.10.5840	translation elongation factor 1-beta, putative	C			C	87.6	N C	2			C
354	Tb927.3.750	hypothetical protein, conserved				M	81.4					
355	Tb927.8.1270	hypothetical protein, conserved										
356	Tb927.4.1920	GPI transamidase, putative				L	93.9	S	2			
357	Tb927.7.3050	hypothetical protein, conserved				G	81.4					
358	Tb927.11.880	cyclophilin a,cyclophilin type peptidyl-prolyl cis-trans isomerase				P	87.6	N C	3			
359	Tb927.1.2100	calpain-like cysteine peptidase, putative,cysteine peptidase, Clan CA, family C2, putative	N			N	100	N C	4			N
360	Tb927.5.800	casein kinase I, isoform 2	C			C	81.4	N C	2			C
361	Tb927.10.8590	flagellum transition zone component	C			C	93.9	N C	4			C
362	Tb927.10.7230	hypothetical protein, conserved	C			C	87.6	N C	3			C
363	Tb927.11.9530	14-3-3-I protein	C			C	93.9	N C	3			C
364	Tb927.4.3450	hypothetical protein, conserved	M			M	87.6				M	M
365	Tb927.11.2430	dynein heavy chain, putative				M	87.6	N C	3			
366	Tb927.10.1660	hypothetical protein, conserved				G	81.4					
367	Tb927.11.1620	hypothetical protein, conserved				P	87.6	N C	2			
368	Tb927.1.2670	flagellar protein PF16	C			C	100	N C	4			C
369	Tb927.11.9570	hypothetical protein, conserved	N			N	81.4	N C	2		PM	N
370	Tb927.10.14980	intraflagellar transport protein IFT52, putative	C			C	93.9	N C	5			C
371	Tb927.11.15550	NADH-cytochrome b5 reductase, putative				M	100					
372	Tb927.3.4650	C-8 sterol isomerase, putative	S	S	1	ER	93.9	S	3			S
373	Tb927.7.2710	NADH-cytochrome b5 reductase, putative				M	81.4					
374	Tb927.5.1710	ribonucleoprotein p18, mitochondrial precursor, putative				S	81.4					
375	Tb927.3.5370	hypothetical protein, conserved	N			N	87.6	N C	7			N
376	Tb927.4.470	snoRNP protein GAR1, putative	N			N	87.6	N C	2			N
377	Tb927.11.2610	hypothetical protein, conserved	N			N	87.6	N C	2			N
378	Tb927.11.4880	hypothetical protein, conserved	C			C	100	N C	4			C
379	Tb927.3.2310	flagellar component,PACRGA				P	93.9	N C	3			
380	Tb927.11.5420	hypothetical protein, conserved				P	93.9	N C	2			
381	Tb927.9.6870	RNA-binding protein, putative	N C			C	93.9	N C	4		N	N C
382	Tb927.10.6220	5'-3' exoribonuclease XRND, putative,5'-3' exoribonuclease 2, putative										
383	Tb927.7.1670	hypothetical protein, conserved	C			C	93.9	N C	4			C
384	Tb927.10.11760	pumilio/PUF RNA binding protein 6	N			N	93.9	N C	4			N
385	Tb927.4.560	dynein heavy chain, putative	C			C	93.9	N C	4			C
386	Tb927.5.2850	radial spoke protein RSP2, putative						N C	2			
387	Tb927.5.2780	mitochondrial DNA polymerase beta	M			M	87.6	M	2			M
388	Tb927.10.9920	hypothetical protein, conserved	N			N	81.4	N C	6			N
389	Tb927.11.16830	Rab-like 5, small G protein	C			P	93.9	N C	4		C	C
390	Tb927.7.920	dynein heavy chain, putative	C			C	87.6	N C	5			C
391	Tb927.11.5180	hypothetical protein, conserved	S	S	1						S	S
392	Tb927.10.15250	paraflagellar rod component, putative	N			N	87.6	N C	4			N
393	Tb927.7.6660	chaperone protein DNAj, putative	M			M	87.6	M	2			M

394	Tb927.3.3330	heat shock protein 20, putative						N C	4		
395	Tb927.1.3950	D-alanine aminotransferase	C			C	93.9	N C	3		C
396	Tb927.8.4230	hypothetical protein, conserved									
397	Tb927.8.7040	hypothetical protein, conserved		M	1	G	87.6				
398	Tb927.5.550	vacuolar ATP synthase, putative	C			C	87.6	N C	3		C
399	Tb927.2.4110	mitochondrial processing peptidase alpha subunit, putative,metallo-peptidase, Clan ME, Family M16				G	87.6	N C	2	M	
400	Tb927.9.10580	3-demethylubiquinone-9 3-methyltransferase, putative				C	81.4	M	2		
401	Tb927.11.3290	hypothetical protein, conserved				N	81.4				
402	Tb927.11.4000	hypothetical protein, conserved	N			N	93.9	N C	6		N
403	Tb927.4.590	hypothetical protein, conserved	S	S	1			S	2		S
404	Tb927.4.600	hypothetical protein, conserved									
405	Tb927.1.1580	cytochrome c oxidase assembly factor, putative,electron transport protein SCO1/2, putative									
406	Tb927.6.2560	hypothetical protein, conserved	S	S	1	M	100	S	2		S
407	Tb927.11.4980	ATP-dependent DEAD/H RNA helicase, putative,ATP- dependent RNA helicase, putative				P	93.9	N C	4		
408	Tb927.11.13510	hypothetical protein, conserved				PM	81.4	N C	3		
409	Tb927.11.7780	hypothetical protein, conserved	C			C	81.4	N C	2		C
410	Tb927.11.7290	pantothenate kinase subunit, putative						N C	3	PM	
411	Tb927.9.9860	hypothetical protein, conserved				M	81.4				
412	Tb927.11.2510	hypothetical protein, conserved				M	100				
413	Tb927.7.3630	TPR-repeat-containing chaperone protein DNAJ, putative						N C	3		
414	Tb927.3.3450	ADP-ribosylation factor-like protein 3A, putative				G	81.4				
415	Tb927.10.15000	hypothetical protein, conserved	C			C	87.6	N C	3		C
416	Tb927.11.10140	hypothetical protein, conserved				M	81.4				
417	Tb927.11.3710	hypothetical protein, conserved	N			N	87.6	N C	3		N
418	Tb927.10.11300	paraflagellar rod component, putative				G	81.4				
419	Tb927.7.4550	60S ribosomal protein-like						M	2		
420	Tb927.11.5820	hypothetical protein, conserved						M	2	PM	
421	Tb927.10.3500	RNA-binding protein, putative	N			N	100	N C	3		N
422	Tb927.10.9810	hypothetical protein, conserved	N			N	87.6	N C	5		N
423	Tb927.10.15170	hypothetical protein, conserved	N			P	93.9	N C	4	N	N
424	Tb927.9.2650	hypothetical protein, conserved				P	87.6	N C	5		
425	Tb927.11.6440	hypothetical protein, conserved	N			N	100	N C	7		N
426	Tb927.5.1520	heat shock protein HslVU, ATPase subunit HslU, putative	M			M	93.9	M	3	M	M
427	Tb927.4.4690	hypothetical protein, conserved	N			N	93.9	N C	4		N
428	Tb927.10.13720	RNA-binding protein, putative	C			C	87.6	N C	2		C
429	Tb927.3.2660	hypothetical protein, conserved	N			N	100	N C	3		N
430	Tb927.7.320	hypothetical protein, conserved	N			N	81.4	N C	4	N	N
431	Tb927.9.9060	p21 antigen protein, putative				P	100				
432	Tb927.11.10660	hypothetical protein, conserved	N			N	87.6	N C	6	PM	N
433	Tb927.11.14090	hypothetical protein, conserved	N			N	87.6	N C	2		N
434	Tb927.9.8740	RNA-binding protein	N			N	87.6	N C	4		N
435	Tb927.7.7500	thymine-7-hydroxylase, putative	C			C	81.4	N C	3		C
436	Tb927.9.8160	chaperone protein DNAj, putative	PM			PM	93.9	N C	3	PM	PM
437	Tb927.2.100	retrotransposon hot spot protein 1 (RHS1), putative				P	93.9	N C	2		
438	Tb927.11.10780	hypothetical protein, conserved				PM	81.4	N C	5		
439	Tb927.2.2970	mitochondrial carrier protein, putative				M	100				
440	Tb927.10.4050	serine palmitoyltransferase, putative				P	87.6	N C	3		
441	Tb04.24M18.150	hypothetical protein, conserved				P	81.4				
442	Tb927.6.4990	ATP synthase, epsilon chain, putative									
443	Tb927.10.9830	hypothetical protein, conserved	M			P	81.4	M	2	M	M
444	Tb927.10.10140	paraflagellar rod component, putative	C			C	93.9	N C	5		C
445	Tb927.6.1570	2-hydroxy-3-oxopropionate reductase, putative				M	87.6				
446	Tb927.8.6240	hypothetical protein, conserved						N C	3		
447	Tb927.11.1670	cysteine desulfurase									
448	Tb927.10.1100	60S ribosomal protein L9, putative	C			P	87.6	N C	4	C	C
449	Tb927.3.2880	hypothetical protein, conserved				G	87.6				
450	Tb927.11.14700	hypothetical protein, conserved				G	81.4				
451	Tb927.10.7350	hypothetical protein, conserved				PM	87.6	N C	4		
452	Tb927.2.270	retrotransposon hot spot protein (RHS, pseudogene), putative,retrotransposon hot spot protein 3 (RHS3), frameshift				M	100				
453	Tb927.10.11390	60S ribosomal protein L6, putative	N			N	93.9	N C	2		N
454	Tb927.8.3290	DNA polymerase zeta catalytic subunit, putative				PM	81.4	N C	2		
455	Tb927.10.15360	hypothetical protein, conserved	N			N	81.4	N C	2	PM	N
456	Tb927.10.6640	COP-coated vesicle membrane protein erv25 precursor, putative,ER--golgi transport protein erv25 precursor, putative	S	S	1	M	93.9	S	2		S
457	Tb927.10.4040	3-keto-dihydrosphingosine reductase	S	S	1			S	4		S
458	Tb927.10.4130	NADH-ubiquinone oxidoreductase complex I subunit, putative,NDUFA5/B13 subunit, putative				G	87.6				
459	Tb927.3.1410	cytochrome oxidase subunit VII				L	93.9	M	2		
460	Tb927.11.4650	hypothetical protein, conserved	M	M	1	G	87.6	M	2		M
461	Tb927.11.6230	pretranslocation protein, alpha subunit, putative,SEC61-like (pretranslocation process) protein, putative	S PM	S	1	PM	87.6	S	2	PM	S PM
462	Tb927.8.2020	agmatinase, putative				M	87.6				
463	Tb927.2.5270	dynein heavy chain, putative	C			C	93.9	N C	6		C
464	Tb927.10.10010	60S acidic ribosomal protein, putative				P	87.6	N C	3		
465	Tb927.4.3890	ATP-dependent RNA helicase, putative	C			C	93.9	N C	4		C

466	Tb927.11.10090	hypothetical protein, conserved				N	87.6				
467	Tb927.4.3070	hypothetical protein, conserved				M	100				
468	Tb927.7.6350	NADH-ubiquinone oxidoreductase, mitochondrial, putative				PM	81.4				
469	Tb927.8.4930	hypothetical protein, conserved	M			M	87.6	M	2		M
470	Tb927.1.90	retrotransposon hot spot protein (RHS, pseudogene), putative				M	100	N C	3		
471	Tb927.9.6920	hypothetical protein, conserved	N			N	93.9	N C	4		N
472	Tb927.6.4320	hypothetical protein, conserved	S	S	1	ER	93.9	S	2		S
473	Tb927.7.6260	hypothetical protein, conserved	N			N	87.6	N C	5		N
474	Tb927.11.4920	hypothetical protein, conserved				P	87.6	N C	4		
475	Tb927.11.15850	hypothetical protein, conserved	M	M	1	M	87.6	M	2		M
476	Tb927.10.510	hypothetical protein, conserved	PM			PM	93.9	N C	2	PM	PM
477	Tb927.9.10520	hypothetical protein, conserved	M			G	93.9	M	2	M	M
478	Tb927.10.15710	mitochondrial carrier protein, putative				P	87.6				
479	Tb927.4.3840	nucleolar protein, putative	C			C	100	N C	6		C
480	Tb927.11.13890	hypothetical protein, conserved				G	87.6	N C	2	M	
481	Tb927.7.5790	protein disulfide isomerase, putative	S	S	1	ER	100	S	3	S	S
482	Tb927.7.7260	kinesin, putative				N	81.4				
483	Tb927.4.1280	hypothetical protein, conserved				N	87.6				
484	Tb927.3.2080	hypothetical protein, conserved				G	81.4				
485	Tb927.11.7520	hypothetical protein, conserved						N C	5		
486	Tb927.5.3800	glutamine hydrolysing (not ammonia-dependent) carbomoyl phosphate synthase, putative	C			C	93.9	N C	2		C
487	Tb927.11.13310	hypothetical protein, conserved	N			N	87.6	N C	2		N
488	Tb927.7.7210	hypothetical protein, conserved	C			C	93.9	N C	3		C
489	Tb927.11.16750	hypothetical protein, conserved				P	81.4	N C	4		
490	Tb927.10.830	adenylate kinase, putative	C			C	87.6	N C	5		C
491	Tb927.7.6990	hypothetical protein, conserved				N	81.4	M	2		
492	Tb927.11.5250	hypothetical protein, conserved				P	81.4	N C	2	PM	
493	Tb927.10.8980	hypothetical protein, conserved	M			M	93.9	M	2		M
494	Tb927.11.12040	hypothetical protein, conserved	S	S	1	S	81.4	S	2	S	S
495	Tb927.11.540	ABC transporter, putative				M	93.9	S	2		
496	Tb927.11.6430	hypothetical protein, conserved	N			N	100	N C	5		N
497	Tb927.1.3450	hypothetical protein, conserved	N			N	87.6	N C	2		N
498	Tb927.8.6640	hypothetical protein, conserved				G	81.4	N C	2		
499	Tb927.11.610	hypothetical protein, conserved				M	81.4				
500	Tb927.8.1870	Golgi/lysosome glycoprotein 1				L	81.4				
501	Tb927.8.6050	hypothetical protein, conserved				N	87.6				
502	Tb927.6.2870	hypothetical protein, conserved				G	87.6				
503	Tb927.6.630	hypothetical protein, conserved		S	1						
504	Tb927.7.4290	hypothetical protein, conserved	C			C	93.9	N C	3		C
505	Tb927.5.4040	hypothetical protein, conserved				G	81.4				
506	Tb927.10.12960	ras-related protein rab-5,small GTPase, putative	N			N	87.6	N C	2		N
507	Tb927.10.2550	malate dehydrogenase-related		M	1	G	87.6				
508	Tb927.3.1820	hypothetical protein, conserved	N			N	100	N C	3		N
509	Tb927.6.3670	paraflagellar rod component, putative	N			PM	81.4	N C	4	N	N
510	Tb927.5.1680	hypothetical protein, conserved	N			N	87.6	N C	2		N
511	Tb927.8.4450	RNA-binding protein, putative	N C			C	93.9	N C	4	N	N C
512	Tb927.6.4080	hypothetical protein, conserved				G	93.9				
513	Tb927.6.1870	eukaryotic translation initiation factor 4e, putative	N			N	87.6	N C	3		N
514	Tb927.11.6660	hypothetical protein, conserved	M	M	1			M	2		M
515	Tb927.9.14420	cyclophilin-like protein, putative				L	87.6				
516	Tb927.10.9820	mitochondrial intermediate peptidase, putative,metallo-peptidase, Clan MA(E) Family M3	M			G	81.4	M	2	M	M
517	Tb927.11.10960	hypothetical protein, conserved								M	
518	Tb927.11.8800	hypothetical protein, conserved				PM	81.4	M	2		
519	Tb927.5.2530	hypothetical protein, conserved				P	87.6	N C	4		
520	Tb927.5.3390	ADG1, pseudogene	S			S	81.4	S	2		S
521	Tb927.7.5680	deoxyribose-phosphate aldolase, putative				P	81.4	N C	2		
522	Tb927.10.6610	chaperone protein DNAj, putative	N			N	87.6	N C	6		N
523	Tb927.11.9560	oxidoreductase, putative				L	87.6				
524	Tb927.10.3640	hypothetical protein, conserved				G	87.6				
525	Tb927.6.4070	hypothetical protein, conserved									
526	Tb927.11.7100	hypothetical protein, conserved				P	81.4	N C	3		
527	Tb927.11.1680	vesicular-fusion protein SEC18, putative				G	87.6				
528	Tb927.8.5860	50S ribosomal protein L17, putative	M			M	93.9			M	M
529	Tb927.10.14770	protein kinase, putative	N			N	93.9	N C	6		N
530	Tb927.7.4460	hypothetical protein, conserved				PM	93.9				
531	Tb927.2.2160	paraflagellar rod component, putative	N			N	81.4	N C	5		N
532	Tb927.10.360	hypothetical protein, conserved	C			C	93.9	N C	4		C
533	Tb927.7.1090	hypothetical protein, conserved	N			N	87.6	N C	6	N	N
534	Tb927.11.8030	hypothetical protein, conserved	N			N	93.9	N C	5		N
535	Tb927.8.3050	hypothetical protein, conserved	S	S	1	ER	87.6	S	2		S
536	Tb927.11.10080	hypothetical protein, conserved	M			M	87.6	M	2		M
537	Tb927.11.10210	hypothetical protein, conserved	N			N	100	N C	4	N	N

538	Tb927.6.5090	hypothetical protein, conserved	PM			PM	81.4	N C	5	PM	PM
539	Tb927.11.460	hypothetical protein, conserved,predicted WD40 repeat protein						N C	2		
540	Tb927.11.11250	cytosolic malate dehydrogenase						N C	2		
541	Tb927.6.1550	hypothetical protein, conserved,leucine-rich repeat protein (LRRP), putative				PM	87.6	N C	3		
542	Tb927.7.540	chaperone protein DNAj, putative						N C	2		
543	Tb927.7.5320	hypothetical protein, conserved	C			C	81.4	N C	3		C
544	Tb927.10.14720	peroxin 13	N			N	100	N C	3		N
545	Tb927.4.1600	hypothetical protein, conserved				G	81.4				
546	Tb927.9.3370	thioredoxin	C			C	100	N C	2		C
547	Tb927.10.10800	hypothetical protein, conserved	S	S	1	ER	81.4	S	2	PM	S
548	Tb927.7.3810	hypothetical protein, conserved				G	81.4			M	
549	Tb927.10.7700	ABC transporter, putative						N C	4	PM	
550	Tb927.8.8120	hypothetical protein, conserved	S	S	1			S	4	S	S
551	Tb927.7.2670	hypothetical protein, conserved,zinc finger protein family member, putative	N			N	81.4	N C	3		N
552	Tb927.9.10070	hypothetical protein, conserved				M	87.6			S	
553	Tb927.10.15950	TFIID-like protein, putative	C			C	87.6	N C	8		C
554	Tb927.5.1160	hypothetical protein, conserved	S	S	1			S	3	S	S
555	Tb927.6.2720	calcium-binding protein, putative						N C	5		
556	Tb927.11.15240	small GTPase, putative,ras-related protein rab-2a, putative	C			C	87.6	N C	2	C	C
557	Tb927.10.600	hypothetical protein, conserved				G	81.4	M	2		
558	Tb927.9.15060	rRNA processing protein, putative	N			N	93.9	N C	6	N	N
559	Tb927.9.5040	cAMP-specific phosphodiesterase	C			C	93.9	N C	5		C
560	Tb927.8.4500	eukaryotic translation initation factor 4 gamma, putative				P	81.4	N C	3		
561	Tb927.9.5280	hypothetical protein, conserved				G	93.9	M	2		
562	Tb927.2.4090	hypothetical protein, conserved	S	S	1			S	3	S	S
563	Tb927.8.4890	endoplasmic reticulum oxidoreductin, putative,pol-associated gene 1	S	S	1	L	87.6	S	4		S
564	Tb927.10.790	vesicle-associated membrane protein, putative,synaptobrevin, putative				M	81.4				
565	Tb927.11.3240	T-complex protein 1, zeta subunit, putative				M	93.9	N C	2		
566	Tb927.11.10170	hypothetical protein, conserved				G	87.6				
567	Tb927.10.10880	ATP-binding cassette protein, putative,ABC transporter, putative,ATPase, putative				P	93.9	N C	5		
568	Tb927.11.2570	hypothetical protein, conserved									
569	Tb927.8.3150	T-complex protein 1, gamma subunit, putative	C			C	93.9	N C	2		C
570	Tb927.11.11160	sodium/sulphate symporter, putative						N C	4	PM	
571	Tb927.10.14030	hypothetical protein, conserved	S	S	1			S	4	S	S
572	Tb927.11.2120	hypothetical protein, conserved				G	81.4	N C	4		
573	Tb927.11.14960	pumilio/PUF RNA binding protein 7, putative				P	81.4				
574	Tb927.6.4970	serine/arginine-rich protein specific kinase SRPK, putative,protein kinase, putative	N			N	100	N C	3	N	N
575	Tb927.10.14390	hypothetical protein, conserved						N C	2		
576	Tb927.9.9940	PACRGB,flagellar component				P	100	N C	2		
577	Tb927.10.4430	pumillo RNA binding protein PUF1	C			C	100	N C	4		C
578	Tb927.6.5210	variant surface glycoprotein (VSG, pseudogene), putative,variant surface glycoprotein, frameshift				N	81.4				
579	Tb927.8.1240	electron transfer flavoprotein-ubiquinone oxidoreductase, putative									
580	Tb927.10.12260	cytosolic nonspecific dipeptidase, putative,peptidase (M20/M25/M40 family), putative				P	81.4	N C	4		
