# Supplementary material for: Proteomic Analysis of the Acidocalcisome, an Organelle Conserved from Bacteria to Human Cells
Source: PLoS Pathog. 2014 Dec 11;10(12):e1004555. doi: 10.1371/journal.ppat.1004555 (PMC4263762; doi:10.1371/journal.ppat.1004555)
Supplement: S5 Table — Subunits of the vacuolar H+-ATPase present in the genome of T. brucei . (PDF) [file ppat.1004555.s015.pdf]

Table S5. Subunits of the vacuolar H^+-ATPase present in the genome of *T. brucei*

TriTrypDB Gene ID	Gene Product	Length (aa)	MW (kDa)	Description
Tb927.4.1080	A (three catalytic sites)	610	67.8	V-type ATPase, A subunit, putative
Tb927.11.11690	B (three non-catalytic sites)	495	55.6	vacuolar ATP synthase subunit b, putative
Tb927.10.14040	C (assembly)	380	43.4	vacuolar ATP synthase subunit c, putative
Tb927.10.3760	D (central stalk)	283	31.3	vacuolar ATP synthase subunit d, putative
Tb927.11.9420	E (peripheral stalk)	216	24.8	ATP synthase, putative
Tb927.1.3820	F (central stalk)	133	14.2	ATP synthase subunit, putative
Tb927.8.2310	G (peripheral stalk)	109	12.8	(H)-ATPase G subunit, putative
Tb927.10.730	H (stabilizer)	468	52.9	ATP synthase, putative
Tb927.5.1300	*a* (proton-translocating)	783	89.6	vacuolar proton translocating ATPase subunit A, putative
Tb927.10.200	c1 (16-kDa proteolipid ring)	191	19.4	vacuolar ATP synthase, putative
Tb927.11.7480	c2 (16-kDa proteolipid ring)	252	26.3	vacuolar type h ATPase subunit, putative
Tb927.6.5050	c3 (22-kDa proteolipid ring)	224	23.1	V-type ATPase, C subunit, putative
Tb927.5.550	d (proton-transporting)	383	42.9	vacuolar ATP synthase, putative
Tb927.11.8130	e (vacuolar acidification)	68	7.7	hypothetical protein, conserved
