# Supplementary material for: Proteomic Analysis of the Acidocalcisome, an Organelle Conserved from Bacteria to Human Cells
Source: PLoS Pathog. 2014 Dec 11;10(12):e1004555. doi: 10.1371/journal.ppat.1004555 (PMC4263762; doi:10.1371/journal.ppat.1004555)
Supplement: S6 Table — Primers used to generate probes for blotting and constructs for antibody production or RNAi (the underlined nucleotides indicate the primer extension sequences for ligation independent cloning or the introduced HindIII and BamHI sites). (PDF) [file ppat.1004555.s016.pdf]

Table S6

Primers used to generate probes for blotting and constructs for antibody production or RNAi (the underlined nucleotides indicate the primer extension sequences for ligation independent cloning or the introduced *Hin*dIII and *Bam*HI sites).

Name	Sequences
TbIP3BD-F	5′-GACGACGACAAGATGGGAAATGTTACCGTGATATGCC-3′
TbIP3BD-R	5′-GAGGAGAAGCCCGGTTTATCCTGCCTTGAGCTCCGGCGCTTGAC-3′
TbTubb-F	5′-ATGCGCGAAAATCGTCTGCGTTCAGG-3′
TbTubb-R	5′-AGTGCAGACGCGGGAATGGGACAAG-3′
TbVAa-IF	5′-CGGGATCCGAGTACGAGGGGGAACTTCGTG-3′
TbVAa-IR	5′-CCCAAGCTTATTTTAACATGTTCGTGGAG-3′
TbVAd-IF	5′-CGGGATCCAATACGCCAGTTTGGTCCAG-3′
TbVAd-IR	5′-CCCAAGCTTCCGGCTGTCATCAAGCAGTTC-3′
TbVIT-IF	5′-CGGGATCCACAAAGAGGTCCACAACCCCTCTG-3′
TbVIT-IR	5′-CCCAAGCTTAGCTGCGGAACGTAACATGCTTAC-3′
TbZnT-IF	5′-CGGGATCCTGGAAATGTGTACAAGTATCGAC-3′
TbZnT-IR	5′-CCCAAGCTTTCGGAGGCAATCGACCACACGTG-3′
