# Supplementary material for: Proteomic Analysis of the Acidocalcisome, an Organelle Conserved from Bacteria to Human Cells
Source: PLoS Pathog. 2014 Dec 11;10(12):e1004555. doi: 10.1371/journal.ppat.1004555 (PMC4263762; doi:10.1371/journal.ppat.1004555)
Supplement: S7 Table — Common features of confirmed acidocalcisome proteins identified by the ELM server. (PDF) [file ppat.1004555.s017.pdf]

Table S7. Common features of acidocalcisome proteins identified by the ELM server

Protein	Signal peptide and other characteristics	CK1 phosphorylation site (MOD_CK1_1)	CK2 phosphorylation site (MOD_CK2_1)	GSK3 phosphorylation recognition site (MOD_GSK3_1)	Phosphothreonine motif binding a subset of FHA domains that show a preference for a large aliphatic amino acid at the pT+3 position (LIG_FHA_1)	NEK2 phosphorylation motif with preferred Phe, Leu or Met in the -3 position to compensate for less favorable residues in the +1 and +2 position (MOD_NEK2_1)	Tyrosine-based sort[illegible] signal responsibl[illegible] for the interactio[illegible] with μ sub[illegible] of adaptor protein complex (TRG_END[illegible] TIC_2)
VP1<br>Tb927.4.4380	Yes (1-24)<br>14 TMD<br>826 aa	SITSGIK (63-69)<br>SGGSDDD (228-234)<br>SCETGAA (503-509)	LFGSFGE (364-370)<br>IAASSAE (378-384)	YVISKRVS (125-132)<br>AVYTNSRT (213-220)<br>AASSAELS (379-386)<br>AELSADFT (383-390)<br>SCETGAAT (503-510)<br>VAISASNT (742-749)	SITSGIK (63-69)<br>HLTDEVM (115-121)<br>EITDALD (588-594)	LMQTYF (277-282)<br>LGGSSV (301-306)<br>LFGSFG (364-369)	YRPV (4944-4[illegible]<br>YGPI (566-569[illegible]
Vtc4<br>Tb927.11.12220	No<br>2 TMD<br>793 aa	*257-263<br>*303-309<br>*367-373<br>*405-411	NPATPDE (40-46)<br>DYLSEEE (81-87)<br>HALSIDE (167-173)<br>RASTKPE (503-509)	MKASDYLS (77-84)<br>DYLSEEET (81-88)<br>TNMSMVFS (90-97)<br>MVFSMVGS (94-101)<br>QTQTVGAS (539-546)<br>HMNSVAPT (572-579)<br>VAPTMSPT (576-583)<br>GVESLCFT (626-633)<br>DPKTLLTS (664-671)<br>PMLTGSDT (785-792)	AITYELL (118-124)<br>LNTDTIE (132-138)<br>GHTPFIK (483-489)<br>DPTIGIA (514-520)<br>RTTDRVI (526-532)<br>AQTQTVG (538-544)<br>PRTDCLR (552-558)	LYNSLL (47-52)<br>MVFSMV (94-99)<br>FMLSKV (176-181)<br>LCFTST (630-635)<br>FTSTGG (632-637)<br>FGNSMS (695-700)	YPDF (12-15)<br>YNSL (48-51)<br>YELL (121-124[illegible]<br>YAVL (147-150[illegible]<br>YHAL (166-169[illegible]<br>YEHV (182-18[illegible]<br>YQRV (560-56[illegible]<br>YTAF (614-617[illegible]<br>YGPM (783-78[illegible]
Vtc1<br>Tb927.7.3900	No<br>3 TMD<br>180 aa	*96-102		RNTSGSVS (42-49)<br>YYRTASSS (165-172)	*141-147	*75-80<br>*84-89<br>*91-96<br>*114-119<br>*149-154	YEDL (33-36)

PMC1<br>Tb927.8.1180	No<br>8 TMD<br>1106 aa	SDGSVAV (31-37)<br>SSVTGEN (244-250)<br>SPLTTVQ (833-839)<br>SDASSLL (1049-1055)	TVVSTAE (266-272)	KDTSNYGT (5-12)<br>AFLSTGLT (22-29)<br>HKVTVDVT (204-211)<br>LSGTVVST (263-270)<br>FSLTVVGT (818-825)<br>EGKSSPLT (829-836)<br>GKSSPLTT (830-837)<br>ALATEQPT (854-861)<br>QRETDGTS (1042-1049)<br>TDGTSDAS (1045-1052)<br>DGTSDASS (1046-1053)	KVTVDVT (205-211)<br>DVTEIVV (209-215)<br>QFTVNLV (810-816)<br>LTTVQLL (835-841)<br>GSTRQLY (1094-1100)	FLSTGL (23-28)<br>LSGTVV (263-268)<br>LMESRL (291-296)<br>LAYSQS (378-383)<br>LQFTVN (809-814)<br>FSLTVV (818-823)<br>LMDTLA (846-851)<br>LYDTLR (1099-1104)	YGTL (10-13)<br>YREL (940-943<br>YDTL (1100-11
IP3R<br>Tb927.8.2770	No<br>5 TMD<br>3099 aa	SAGTRMA (216-222)<br>SSKSQGS (347-353)<br>SQGSDSH (350-356)<br>SLDSDPC (362-368)<br>SVVSQRS (471-477)<br>SQRSCCN (474-480)<br>SEGSETL (549-555)<br>SMCSVGT (866-872)<br>SVGTHGV (869-875)<br>SDRSRVC (988-994)<br>SELTQAV (1049-1055)<br>SAVTTQL (1251-1257)<br>SGSTKDV (1258-1264)<br>STQTQNE (1373-1379)<br>SSSSAPE (1422-1428)<br>SETSRVP (1469-1475)<br>SYGTSYT (1678-1684)<br>SFGSITL (2012-2018)<br>SVITDIF (2050-2056)<br>SFETDYA (2060-2066)<br>SLYSSLK (2188-2194)<br>STGTGIT (2328-2334)<br>SNSTDNK (2341-2347)<br>SAITHNG (2764-2770)<br>SWNSSTL (2848-2854)<br>SSQSSSL (3063-3069)	ADGTFSE (399-405)<br>AVDTILE (457-463)<br>FLWSQCE (582-588)<br>QTETDGE (611-617)<br>TSPTLME (630-636)<br>CQRTLAE (679-685)<br>VRNSSLE (836-842)<br>VLDSGGE (898-904)<br>YVQSELE (940-946)<br>HQVSATE (1094-1100)<br>YTWTRLE (1129-1135)<br>FVTSPTE (1207-1213)<br>IRGSVEE (1354-1360)<br>STQTQNE (1373-1379)<br>VTGTTNE (1380-1386)<br>SSSSAPE (1422-1428)<br>VHITMLE (1490-1496)<br>LSQSNAE (1520-1526)<br>LMQSRTE (1757-1763)<br>VGATFHE (1808-1814)<br>LLLSCRE (1987-1993)<br>VQCTNAE (2147-2153)<br>DCCTLNE (2351-2357)<br>LNETDSE (2355-2361)<br>CLLSLIE (2389-2395)<br>AYDSILE (2426-2432)	SQGSDSHS (350-357)<br>DSHSAGAT (354-361)<br>AGATSLDS (358-365)<br>SLDSDPCS (362-369)<br>VEGSAVDT (453-460)<br>GDGSPEGS (464-471)<br>YLCTSEGS (5454-552)<br>SEGSETLS (549-556)<br>GSETLSLS (551-558)<br>LSLSWKPS (555-558)<br>FLWSQCET (582-589)<br>QVLTTEGT (598-605)<br>RYPSAEGS (622-629)<br>AEGSTSPT (626-633)<br>PKGSNVGT (660-667)<br>NVGTGYAS (664-671)<br>DSLTRLFS (824-831)<br>SVGTHGVT (869-876)<br>HQVSATET (1094-1101)<br>KGPSDYQS (1239-1246)<br>YQSTVAGS (1244-1251)<br>VAGSAVTT (1248-1255)<br>SAVTTQLS (1251-1258)<br>RICTVRRS (1312-1319)<br>RRRSVGLS (1462-1469)<br>GPSSPAFS (1502-1509)	RQTHSVM (323-329)<br>NLTGDIV (480-486)<br>SETLSLS (552-558)<br>VGTHGVT (870-876)<br>ELTQAVK (1050-1056)<br>EGTLHVI (1057-1063)<br>SNTHFVR (1065-1071)<br>TETACLV (1099-1105)<br>AVTTQLS (1252-1258)<br>GSTKDVA (1259-1265)<br>SETSRVP (1469-1475)<br>DPTPHLT (1562-1568)<br>RFTACIV (1640-1646)<br>PVTHLLE (1668-1674)<br>SYTPLVE (1682-1688)<br>SRTETVL (1760-1766)<br>VQTGNLV (1852-1858)<br>IATADVP (1919-1925<br>AATKCLD (1945-1951)<br>GATRAIA (2082-2088)<br>EFTRMLL (2173-2179)<br>LLTNALT (2178-2184)<br>ALTQCLL (2385-2391)<br>QHTNKLC (2472-2478)<br>CFTFILW (2825-2831)<br>RSTCFIC (2899-2905)	MALSAV (221-226)<br>LATSVA (285-290)<br>LLKTRQ (319-324)<br>LQCSCT (412-417)<br>LQRTLF (511-516)<br>LSLSWK (555-560)<br>FLWSQC (582-587)<br>LHLTRS (852-857)<br>LTRSGR (854-859)<br>LLCSMC (863-868)<br>LLGSSK (1027-1032)<br>LGSSKL (1028-1033)<br>LSGSTK (1257-1262)<br>LSETSR (1468-1473)<br>FTLSQS (1518-1523)<br>LSQSNA (1520-1525)<br>FNLSSN (1539-1544)<br>LRESAR (1572-1577)<br>FTKTTV (1601-1606)<br>LGRSAL (1662-1667)<br>LEITLS (1673-1678)<br>LWESNS (1695-1700)<br>LLLSRQ (1745-1750)<br>LMQSRT (1757-1762)<br>MCMSVA (1815-1820)<br>LSGTML (1863-1868)	YVAV (450-453<br>YNHI (645-648<br>YASL (669-672<br>YLQL (860-863<br>YSFV (895-898<br>YCSL (1191-11<br>YNDL (1398-14<br>YPFI (1498-15<br>YIDI (1531-153<br>YDAF (1621-16<br>YTPL (1683-16<br>YRRL (1776-17<br>YRDV (1804-18<br>YRRL (1843-18<br>YKKI (1882-18<br>YSAV (2100-21<br>YSSL (2190-21<br>YDNL (2249-22<br>YDSI (2427-24<br>YRLL (2726-27<br>YLYF(2933-29

		SQSSSLR (3064-3070)<br>SAASVAE (3077-3083)<br>SKGTWLR (3087-3093)	RGLSAKE (2707-2713)<br>QFGSNGE (2798-2804)<br>NEFTGQE (2946-2952)<br>EADSDDE (2990-2996)<br>SAASVAE (3077-3083)<br>LRHSEPE (3092-3098)	YVFTLSQS (1516-1523)<br>LSQSNAES (1520-1527)<br>IVLSILAT (1552-1559)<br>KDPTPHLT (1561-1568)<br>CLPSPGVT (1626-1633)<br>ITLSYGTS (1675-1682)<br>LMQSRTET (1757-1764)<br>LSGTMLTT (1863-1870)<br>LLISGAAT (1940-1947)<br>QLITMEDS (1967-1974)<br>KHVSAENS (2042-2049)<br>AENSSVIT (2046-2053)<br>SVITDIFT (2050-2057)<br>MLLTNALT (2177-2184)<br>NALTTPPS (2181-2188)<br>TPPSLYSS (2185-2192)<br>VVISKLLS (2309-2316)<br>PDVTGTPS (2321-2328)<br>DCCTLNET (2351-2358)<br>DEVSWNSS (2845-2852)<br>NEFTGQES (2946-2953)<br>INRSSQSS (3060-3067)<br>NRSSQSSS (3061-3068)<br>SAASVAET (3077-3084)<br>ETSTSKGT (3083-3090)	ERTRGLA (3048-3054)	MLTTRR (1867-1872)<br>LAETGP (1902-1907)<br>LLISGA (1940-1945)<br>LLLSCR (1987-1992)<br>FVSTTL (200-2005)<br>LLQTTF (2073-2078)<br>LQTTFS (2074-2079)<br>LGATRA (2081-2086)<br>MLLTNA (2177-2182)<br>LYSSLK (2189-2194)<br>FVCTLF (2222-2227)<br>MLQSAF (2279-2284)<br>FLKTVR (2451-2456)<br>LQHTNK (2471-2476)<br>FNETLQ (2698-2703)<br>LLVTRF (2717-2722)<br>LRSTCF (2898-2903)<br>LASSVV (2997-3002)	
AP-3, β3<br>Tb927.11.10650	No<br>No TMD<br>918 aa	SLASERA (11-17)<br>SGGSIVS (23-29)<br>SIVSRAR (26-32)<br>SLVSGDA (33-39)<br>SDSSDEA (276-282)<br>SSETTSS (306-312)<br>SETTSSS (307-313)<br>SSSSSSW (311-317)<br>SSSSSWD (312-318)<br>SSSSWDR (313-319)<br>SLNSAVV (352-358)<br>SIISKLA (433-439)<br>SVCTSQV (479-485)<br>SFKTEGS (575-581)	AALSFVE (213-219)<br>ISDSSDE (275-281)<br>DGESSSE (302-308)<br>IVLTHAE (402-408)<br>AEHTHYE (667-673)<br>GNSTSDE (779-785)<br>EQRTPAE (793-799)<br>TVPTETE *48-854)	EFISSGGS (19-26)<br>SIVSRARS (26-33)<br>VRKTAAIS (170-177)<br>DGESSSET (302-309)<br>GESSSETT (303-310)<br>ESSSETTS (304-311)<br>SSETTSSS (306-313)<br>SETTSSSS (307-314)<br>ETTSSSSS (308-315)<br>TTSSSSSS (309-316)<br>ERHSVVLS (390-397)<br>VVLSTIHT (394-401)<br>TIHTIVLT (398-405)<br>SIISKLAT (433-440)	LDTEPLV (164-170)<br>RKTAAIS (171-177)<br>AATAVIC (360-366)<br>ACTLPLL (376-382)<br>IHTIVLT (399-405)<br>VCTSQVM (480-486)<br>RQTARLV (520-526)<br>KTTASVE (893-899)	FISSGG (20-25)<br>FFVTHY (104-109)<br>LSISAF (119-124)<br>MDHSMH (129-134)<br>LLDSVK (342-347)<br>LLMSLN (349-354)<br>LKLSII (430-435)<br>LVKSFK (572-577)<br>LDGSGA (598-603)	YFFV (103-106<br>YSIL (195-198)<br>YRNL (232-235<br>YRLL (527-530<br>YARL (630-633<br>YHPL (688-691

		SDNSSDS (737-743)<br>SSDSGTC (740-746)<br>SGSSDAS (749-755)<br>SDASTSV (752-758)<br>SVHSSYT (757-763)<br>SSYTGDD (760-766)<br>SDASSKT (806-812)<br>SSKTNIT (809-815)<br>SGGTPGA (870-876)<br>SSTTAGN (877-883)		VRLSSVCT (475-482)<br>RLSSVCTS (476-483)<br>GNSSGRQT (515-522)<br>GADSGDDS (715-722)<br>DDSSMMYS (730-737)<br>MMYSDNSS (734-741)<br>DNSSDSGT (738-745)<br>DSGTCRGS (742-749)<br>GSGSSDAS (748-755)<br>SGSSDAST (749-756)<br>DASTSVHS (753-760)<br>ASTSVHSS (754-761)<br>GYSSTDGS (767-774)<br>IRPSDASS (803-810)<br>SGGTPGAS (870-877)<br>DTPTREGS (907-914)			
AP-3, δ<br>Tb927.5.3610	No<br>1 TMD<br>1127 aa	SNESEAE (27-33)<br>SEVTSTV (44-50)<br>STVSSPD (135-141)<br>SMLSTIQ (232-238)<br>SNWTLIK (239-245)<br>SDPSRSS (472-478)<br>SRSSQWE (475-481)<br>SNGTAKV (576-582)<br>SAVSAAP (733-739)<br>SATTEGT (815-821)<br>SSSSMRN (879-885)<br>SDRSIFV (943-949)<br>SFYSVLQ (1067-1073)<br>SRKSAAN (1074-1080)<br>SEHTSLA (1105-1111)	FQNTLAE (13-19)<br>KLRSSNE (23-29)<br>SNESEAE (27-33)<br>KLDSSSE (190-196)<br>VLATVIE (395-401)<br>SRSSQWE (475-481)<br>DSLTFEE (556-562)<br>LSPSDSE (671-677)<br>LPPSLTE (971-977)<br>VDTTSVE (1010-1016)<br>EHTSLAE (1106-1112)	KLRSSNES (23-30)<br>DIKSEVTS (41-48)<br>EVTSTVQS (45-52)<br>TVQSVKVT (49-56)<br>YCISTVSS (132-139)<br>YPESLRPT (176-183)<br>PFFSMLST (229-236)<br>SMLSTIQS (232-239)<br>VQTTGAKS (272-279)<br>NGMSKTPS (290-297)<br>MSKTPSLT (292-299)<br>EVLSGLVT (360-367)<br>VAQTDDYS (402-409)<br>NELSQLLS (458-465)<br>LLLSERIS (506-513)<br>LRASILQT (563-570)<br>ILQTEAIT (567-574)<br>VGATGSIS (583-590)<br>AGLSPSDS (669-676)<br>QSATTEGT (814-821)<br>VEISNLAS (872-879)<br>SDRSIFVS (943-950)<br>KGTTTLRT (954-961)	EVTSTVQ (45-51)<br>KVTAVLK (54-60)<br>PKTEVLP (103-109)<br>LLTALLK (110-116)<br>SKTPSLT (293-299)<br>SLTKIVA (297-303)<br>SNTNLLK (465-471)<br>RRTCELL (501-507)<br>LQTEAIT (568-574)<br>NGTAKVV (577-583)<br>GATGSIS (584-590)<br>DVTRSLA (792-798)<br>RVTAFVL (851-857)<br>QITMAVE (867-873)<br>YTTERVR (897-903)<br>KGTTTLR (954-960)<br>LRTKMLL (959-965)<br>SLTEPLL (974-980)<br>KPTESLL (90-996)<br>VDTTSVE (1010-1016)<br>VLTCFVA (1031-1037)	FQNTLA (13-18)<br>LSSTNQ (119-124)<br>MLSTIQ (233-238)<br>FVSTIN (371-376)<br>LDISLV (422-427)<br>LSNTNL (464-469)<br>LRASIL (563-568)<br>FRHSVH (606-611)<br>LQQSAT (812-817)<br>LRVTAF (850-855)<br>FVLSCR (855-860)<br>LASSSS (877-882)<br>MKGTTT (953-958)<br>MLLTFS (963-968)<br>LTFSGS (965-970)<br>FSGSLP (67-972)<br>LLFSLL (979-984)<br>FAKSHV (1005-1010)<br>FMQTIM (1017-1022)<br>LLMTAT (1121-1126)	YKRI (89-92)<br>YRIF (170-173)<br>YPRL (184-187<br>YECI (282-285<br>YLGL (319-322<br>YSYV (408-411<br>YVKI (417-420)<br>YDAI (693-696<br>YGRL (809-81<br>YLRV (849-852<br>YRYF (1002-10<br>YSVL (1069-10

				MLLTFSGS (963-970)<br>FSGSLPPS (967-974)<br>LLFSLLYT (979-986)<br>FAKSHVDT (1005-012)<br>LSESPVLT (1026-1033)<br>SFYSVLQS (1067-1074)<br>AVKSEHTS (1102-1109)			
VSP<br>Tb927.11.7060	No<br>No TMD<br>414 aa	SDRTSPQ (11-17)<br>SLGTNIS (32-38)<br>SMVSADF (153-159)	DLETGRE (189-195)<br>LVRTKPE (211-217)	DSRSDRTS (8-15)<br>SLGTNIST (32-39)<br>NISTGRGS (36-43)<br>KIMSRLFS (79-86)<br>HSRTKCFS (146-153)<br>SMVSADFS (153-160)	NNTHGVD (2-8)	MNNTHG (1-6)<br>FSASLG (29-34)<br>FDVTHL (88-93)<br>LVRTKP (211-216)	YRVL (181-184[illegible]
V-H^+-ATPase,<br>Subunit *a*<br>Tb927.5.1300	No<br>6 TMDP<br>783 aa	SDVSAFQ (47-53)<br>SNRTQEH (123-129)<br>SVFSTMN (281-287)<br>SGSTAVA (292-298)<br>SVDTFRS (447-453)<br>SSPSGVT (472-478)<br>SGVTPAR (475-481)<br>SQLSEVF (698-704)	QYQSLLE (115-121)<br>TPFSEGE (183-189)<br>TLNSQLE (239-245)<br>IRASLQE (310-316)<br>QVLTIVE (324-330)<br>EEISTKE (330-336)<br>EAPSLLE (573-579)	LWRSEDMT (9-16)<br>QRETAHDS (23-30)<br>AGLTCVST (80-87)<br>YRITRGNS (167-174)<br>RGNSVLHT (171-178)<br>VLHTDEIT (175-182)<br>DEITTPFS (179-186)<br>TLNSQLET (239-246)<br>QLETMKHT (243-250)<br>MKHTLQQS (247-254)<br>VEKSVFST (278-285)<br>KRPTCFFT (337-344)<br>CFFTNKFT (341-348)<br>CFQSIVDS (350-357)<br>SGVTPARS (475-482)<br>QRTSEAPS (569-576)<br>EAPSLLET (573-580)<br>GCVSNTAS (679-686)<br>WALSLAHS (691-698)	VSTEAIG (85-91)<br>LLTGVIP (149-155)<br>LHTDEIT (176-182)<br>FATPRLW (203-209)<br>ESTERLQ (228-234)<br>RQTVAVE (273-279)<br>PGTVNVP (588-594)	LRLTMQ (18-23)<br>LEESNR (120-125)<br>LWESLK (208-213)<br>MRDTLN (236-241)<br>MKHTLQ (247-252)<br>LQQSLL (251-256)<br>LLTSIS (261-266)<br>LKFSGS (289-294)<br>FSGSTA (291-296)<br>FYNSVK(500-505)<br>LLETMT (577-582)<br>LAHSQL (695-700)	YQSL (116-119[illegible]<br>YKEV (363-366[illegible]<br>YNSV (501-504[illegible]<br>YLRL (687-690[illegible]
V-H^+-ATPase,<br>Subunit *d*<br>Tb927.5.550	No<br>No TMD<br>383 aa	SFGSLVD (245-251)<br>STSSSSA (286-292)<br>SSSSAKG (288-294)<br>SGQTGAS (309-315)	AGASSLE (317-323)	ADLTVLRS (217-224)<br>VLRSTVNT (221-228)<br>LDDSRYNS (279-286)<br>RYNSTSSS (283-290)<br>YNSTSSSS (384-291)	GATAEVM<br>(203-209)<br>VNTMGVP (226-232)<br>EVTVALY (328-334)	LRSTVN (222-227)<br>LDDSRY (279-284)<br>LEVSNL (356-361)	YASL (32-35)<br>YGNF (53-56)<br>YEHM (102-10[illegible]<br>YEQF (191-194[illegible]<br>YGVF (344-347[illegible]

				NAVSGAAS (302-309)			YVSI (379-382)
Pho91<br>Tb927.11.11160	No<br>9 TMD<br>728 aa	SPLTGVE (32-38)<br>SHPSAHN (274-280)<br>SVLSFHD (452-458)	SPLTGVE (32-38)<br>KLNSPRE (99-105)<br>AFESSTE (110-116)<br>LGRTHLE (356-362)<br>IRPTLWE (408-414)	FIHSSPLT (28-35)<br>FESSTENS (111-118)<br>KNITINQT (241-248)<br>VASTTVAS (324-331)<br>GMLSPIAS (437-444)<br>IADSLHDT (591-598)<br>PPYTTLVS (603-610)	PLTGVEF (33-39)<br>FMTDVDA (221-227)<br>GRTHLEV (357-363)<br>RPTLWEF (409-415)	FIHSSP (28-33)<br>LGRTHL (356-361)<br>LHDTLV (595-600)	YKRL (22-25)<br>YTTL (605-608<br>YVVF (721-724
Vacuolar iron transporter<br>Tb927.3.800	No<br>3 TMD<br>281 aa	SNSSVAS (78-84)<br>SVASVLI (81-87)<br>SGVSMLR (242-248)	IEMSRME (30-36)	GSNSSVAS (77-84)<br>SGVSMLRS (242-249)	FETGDIE (25-31)<br>DATTIVN (150-156)<br>MMTEELG (169-175)	FGFSNV (88-93)<br>MLRSAA (246-251)	YKSI (15-18)<br>YKEV (42-45)
Zinc transporter<br>Tb927.8.7460	No<br>5 TMD<br>471 aa	SLNSTEV (48-54)<br>SIMSLRA (106-112)<br>SRTSCGK (114-120)<br>SIASDYA (410-416)	TEATHME (202-208)<br>EEDSLCE (261-267)<br>HGHSGSE (282-288)<br>HSGSEGE (284-290)	PCSSGPHS (13-20)<br>DQQSLNST (45-52)<br>GNISQRPT (187-194)<br>EMCTSIDS (208-215)<br>FGGSHHHS (247-254)<br>HDHSHSHS (292-299)<br>INYSELLS (385-392)<br>ELLSALRS (389-396)<br>LCHSLCAS (459-466)	HTTIQVD (445-451)	MLATTA (167-172)<br>LATTAQ (168-173)<br>FGGSHH (247-252)<br>LMESTP (377-382)<br>FGITHT (441-446)<br>LCHSLC (459-464)<br>LCASAQ (463-468)	YYGF<br>YSEL<br>YAAL
Acid phosphatase<br>Tb927.10.7020	Yes<br>0 TMD<br>435 aa		NDYSDTE (304-310)<br>YSDTEQE (306-312)<br>PEKSVGE (357-363)<br>QLCTLKE (395-401)<br>CNFTITE (420-426)	PVSDMDT (161-168)		LVLTKL (48-53)<br>FNRSRH (116-121)<br>LNTSLS (190-195)<br>FLKTVA (227-232)<br>LNRSTS (292-297)<br>FGFTFN (330-335)	YWSM (68-71)<br>YRDF (108-11)<br>YAFL (225-228)<br>YTSV (279-282)<br>YRIL (286-289)<br>YVRL (371-374)
Polyamine Transporter 1<br>Tb927.9.10340	Yes<br>11 TMD<br>492	SNVTDER (4-40)<br>SAFSKSL (151-157)	WNFSGFE (227-233)<br>FEGSIVE (472-47*)	VDNSLYPT (131-138)<br>TITTVPQS (208-215)<br>WNFSGFES (227-234)<br>QLTTTSRS (321-328)		MHMSNV (1-6)<br>LGISAF (148-153)<br>FSKSLV (153-158)<br>LTTTSR (322-327)<br>MCASLK (439-444)<br>FEGSIV (472-477)	YPTI (136-139)<br>YESL (488-491)

* excluded ELMs falling inside SMART/PFAM domains and/or scoring poorly with the structural filter.
